# A Novel PCR-Based Approach for Accurate Identification of *Vibrio parahaemolyticus*

**DOI:** 10.3389/fmicb.2016.00044

**Published:** 2016-01-28

**Authors:** Ruichao Li, Jiachi Chiou, Edward Wai-Chi Chan, Sheng Chen

**Affiliations:** ^1^Shenzhen Key lab for Food Biological Safety Control, Food Safety and Technology Research Center, Hong Kong PolyU Shen Zhen Research InstituteShenzhen, China; ^2^State Key Laboratory of Chirosciences, Department of Applied Biology and Chemical Technology, The Hong Kong Polytechnic UniversityHung Hom, Hong Kong

**Keywords:** *Vibrio parahaemolyticus*, *bla*_CARB-17_, molecular detection, PCR

## Abstract

A PCR-based assay was developed for more accurate identification of *Vibrio parahaemolyticus* through targeting the *bla*_CARB-17_ like element, an intrinsic β-lactamase gene that may also be regarded as a novel species-specific genetic marker of this organism. Homologous analysis showed that *bla*_CARB-17_ like genes were more conservative than the *tlh, toxR* and *atpA* genes, the genetic markers commonly used as detection targets in identification of *V. parahaemolyticus*. Our data showed that this *bla*_CARB-17_-specific PCR-based detection approach consistently achieved 100% specificity, whereas PCR targeting the *tlh* and *atpA* genes occasionally produced false positive results. Furthermore, a positive result of this test is consistently associated with an intrinsic ampicillin resistance phenotype of the test organism, presumably conferred by the products of *bla*_CARB-17_ like genes. We envision that combined analysis of the unique genetic and phenotypic characteristics conferred by *bla*_CARB-17_ shall further enhance the detection specificity of this novel yet easy-to-use detection approach to a level superior to the conventional methods used in *V. parahaemolyticus* detection and identification.

## Introduction

*Vibrio* sp. are gram-negative and halophilic bacteria that inhabit the estuarine and marine environment and some species can cause gastrointestinal diseases in human (Austin, 2010; Scallan et al., 2011; Letchumanan et al., 2014). Infections caused by the pathogenetic *Vibrio* sp. are often due to consumption of raw or undercooked seafood, with *V. parahaemolyticus* being one of the most important foodborne pathogens worldwide (Su and Liu, 2007; Gonzalez-Escalona et al., 2011; Letchumanan et al., 2015). Although infections caused by *V. parahaemolyticus* are always self-limiting, they can be life-threatening in patients who suffer from liver dysfunction or suppressed immunity (Ottaviani et al., 2012).

Identification of *V. parahaemolyticus* is conventionally conducted by performing biochemical tests upon isolation of the organisms from selective agar plates (Di Pinto et al., 2011). However, identification of *V. parahaemolyticus* by phenotypic approaches has some drawbacks such as being labor-intensive, time-consuming and not very effective in terms of detection specificity (Di Pinto et al., 2011; Izumiya et al., 2011). To cope with the problems caused by conventional microbiological culture method, some rapid detection techniques based on genus or species-specific genotypic features have been developed recently (Bej et al., 1999; Ward and Bej, 2006; Bauer and Rorvik, 2007; Neogi et al., 2010; Izumiya et al., 2011; Liu et al., 2012; Vinothkumar et al., 2013). Many of the targeting genes used in these approaches are phylogenetic markers or those involved in virulence (*tlh, toxR, atpA* etc.), yet some of which are not highly species-specific as different *Vibrio* species may share similar sequences, thus reducing the accuracy and specificity of such detection methods. *V. parahaemolyticus* is a member of the *V. harveyi* group, which comprises *V. alginolyticus, V. harveyi*, and *V. campbellii* etc. These species exhibited a high degree of genetic relatedness in phylogenetic analysis (Thompson et al., 2007). However, in our routine identification of *V. parahaemolyticus*, we noticed that PCR targeting *tlh* often could not differentiate organisms in the *V. harveyi* group, especially *V. parahaemolyticus* and *V. alginolyticus*. This phenomena is in agreement with previous findings that *tlh* was distributed among *V. alginolyticus* (Xie et al., 2005), and that similar virulence-related genes in *V. parahaemolyticus* also existed in other *Vibrionaceae* species(Klein et al., 2014). However, no investigation has been done to compare the specificity of these genetic markers in detecting *V. parahamolyticus*.

Our laboratory recently identified a β-lactamase that contributed to intrinsic ampicillin resistance in *V. parahaemolyticus* (Chiou et al., 2015). The gene encoding this enzyme is an intrinsic gene in *V. parahaemolyticus* and is more conserved in this species compared to other gene markers. It bears all the hallmarks of a unique marker suitable for *V. parahaemolyticus* detection and identification. In recent years, species-specific β-lactamase genes are being explored as targets for development of combined genetic and phenotypic bacteria identification approaches. An excellent example being the PCR detection method targeting the intrinsic β-lactamase gene, *bla*_OXA-51_ like gene, which can be applied for *Acinetobacter baumannii* detection (Turton et al., 2006). In this study, we attempted to develop a reliable and simple PCR assay targeting *bla*_CARB-17_ for detection and identification of *V. parahaemolyticus.*

## Materials and Methods

### Bacterial Strains

A total of 120 *V. parahaemolyticus* strains and 109 non- *V. parahaemolyticus* strains were included in this study. All strains were identified using 16S rRNA sequencing, API 20E test strips and Vitek 2 Compact system (bioMerieux, Inc.). Genomic DNA extraction was conducted by the boiling method as previously described (Pathmanathan et al., 2003). Briefly, 1 ml of overnight culture was centrifuged and the pellet was suspended in 400 μl of ddH_2_O. The bacterial suspension was boiled for 5 min and centrifuged at 11,000 *g* for 6 min. The supernatant was used as DNA template for PCR assay.

### Phylogenetic Analysis of Different Genetic Markers within the *Vibrio* sp.

Homology analysis of different *atpA, tlh, toxR* and *bla*_CARB-17_ variants was performed with the DNAMAN software (Lynnon Biosoft Corporation, USA^1^) by quick alignment method and default parameters were used. Sequences available at NCBI nucleotide collection (nr) and whole genome shotgun(wgs) databases were employed to retrieve *atpA, tlh, toxR* and *bla*_CARB-17_-like sequences. The four genes were almost identical among the strains from the same species, therefore only one representative sequence per specie was used to build the homology tree. The sequences used to construct the homology trees were displayed in Supplementary Materials.

### Development of a PCR Method Targeting *bla*_CARB–17_ Like Genes in *V. parahaemolyticus*

In order to design specific primers for *bla*_CARB-17_ gene detection in *V. parahaemolyticus*, the conserved regions of this gene in the *V. parahaemolyticus* genome was screened for selection of target regions, followed by development of a PCR-based mismatch amplification mutation assay. Upon sequence alignment, two regions (550–565, 834–852) that corresponded to the location of *bla*_CARB-17_ (KJ934265) were selected for primer design. Eventually, two degenerate primers (**Table 1**), named CARB-VP-F (ACYTTGATGGAAGATA) and CARB-VP-R (YTAACTTTCTTTGTAGTGM) respectively, were generated. Primer-Blast was used to check primer pair specificity^2^. The result showed that this primer set did not exhibit significant sequence homology to other DNA fragments in the NCBI nr database.

**Table 1:** Primers used in comparison of detection specificity of different *V. parahaemolyticus* detection methods.

Primer names	Primer sequences	Product length	Target genes	References
CARB-F	ACC(T)TTGATGGAAGATA	303 bp	*bla*_CARB-17_	This study
CARB-R	T(C)TAACTTTCTTTGTAGTGC(A)			
TLH-F	AAAGCGGATTATGCAGAAGCACTG	450 bp	*tlh*	Dickinson et al., 2013
TLF-R	GCTACTTTCTAGCATTTTCTCTGC			
atpA-VP-F	TACTAGGCCGCGTAGTA	794 bp	*atpA*	Izumiya et al., 2011
atpA-VP-R	CGCTGGACGTACACCT			
toxR-VP-F	GTCTTCTGACGCAATCGTTG	350 bp	*toxR*	Kim et al., 1999
toxR-VP-R	ATACGAGTGGTTGCTGTCATG			

PCR reactions with the designed primers were optimized by testing different annealing temperatures, primer concentrations and extension times. Each reaction mixture (20 μl) contained 10 μl of Premix Ex Taq^TM^ (TaKaRa, Japan), 0.5 μl of DNA template, 1 μl of forward and reverse primers(10 pM) respectively, and 7.5 μl of nuclease-free water. The PCR amplification program was as follows: 95°C for 5 min, followed by 30 cycles of 95°C for 30 s, 50°C for 30 s, and 72°C for 30 s, and final elongation at 72°C for 5 min. The annealing temperature 50°C was obtained through the comparison of three different temperatures, 45, 50, and 55°C. The PCR products were differentiated on 1.5% agarose and visualized by the Gel Doc System (Biorad). The specificity of the PCR method was tested with 120 *V. parahaemolyticus* strains and 109 non- *V. parahaemolyticus* strains (**Table 2**).

**Table 2:** Results of the specificity of PCR methods targeting different genes in *Vibrio parahaemolyticus* and non- *Vibrio parahaemolyticus* strains.

Species	Source	No. of strains	Positive rate (No. of positive strains)			
			*bla*_CARB-17_	*tlh*	*atpA*	*toxR*
*Vibrio parahaemolyticus*	Food, Clinical	120	100%	100%	100%	100%
*Vibrio cholerae*	Food	26	0	0	89% (23)	0
*Vibrio vulnificus*	Food, Clinical	4	0	0	0	0
*Vibrio alginolyticus*	Food	35	0	20% (7)	2.8% (1)	0
*Vibrio metschnikovii*	Food	1	0	0	0	0
*Vibrio fluvialis* ATCC33809	ATCC	1	0	0	0	0
*Vibrio harveyi* ATCC33842	ATCC	1	0	0	0	0
*Vibrio mimicus* ATCC 33653	ATCC	1	0	0	0	0
*Vibrio campbellii* ATCC 33865	ATCC	1	0	0	0	0
*Vibrio natriegens* ATCC 14048	Food	1	0	0	0	0
*Aeromonas* sp.	Food	7	0	0	0	0
*Escherichia coli*	Food, clinical	10	0	0	0	0
*Salmonella* sp.	Food, clinical	10	0	0	0	0
*Enterobacter* sp.	Clinical	2	0	0	0	0
*Pseudomonas aeruginosa* PAO1	Clinical	1	0	0	0	0
*Citrobacter freundii*	Clinical	2	0	0	0	0
*Klebsiella pneumonia*	Clinical	1	0	0	0	0
*Proteus mirabilis*	Food	2	0	0	0	0
*Myroides odoratimimus*	Food	2	0	0	0	0
*Staphylococcus aureus*	Food	1	0	0	0	0

### Comparison of *bla*_CARB_ Detection with Other Reported PCR Detection Methods

Other reported PCR detection methods targeting *tlh, atpA* and *toxR* genes were included in this study to compare the specificity between these methods (Kim et al., 1999; Izumiya et al., 2011; Dickinson et al., 2013). The primers used were listed in **Table 1**. PCR reactions were conducted according to those conditions previously reported.

## Result and Discussion

**FIGURE 1:**
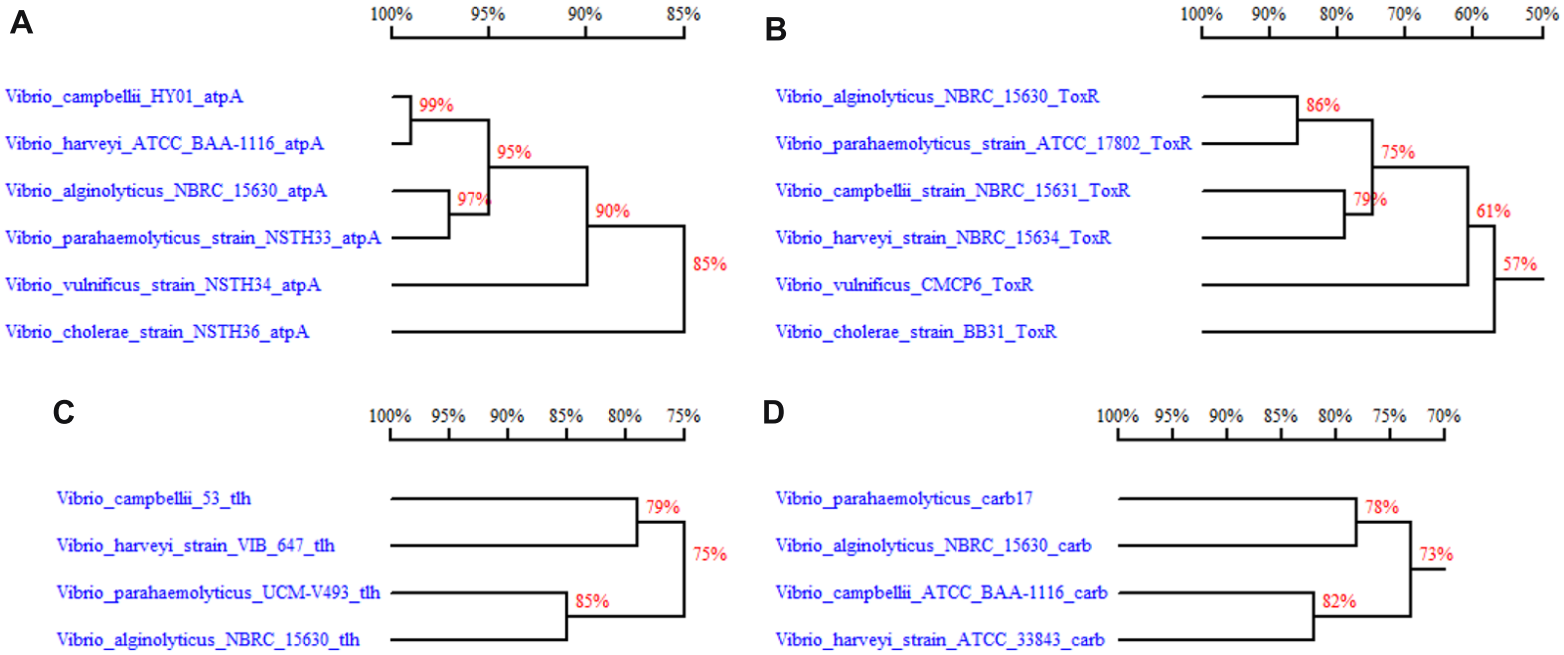
**Comparison of sequence homology comparison between related *Vibrio* sp. for four different genes used as *Vibrio parahaemolyticus* detection targets. (A)** *atpA* gene homology comparison between *Vibrio* sp. (NCBI accession no. AAWP01000035, CP006605.1, BATK01000022.1, KF886608.1, KF886609, KF886611); **(B)** *toxR* gene homology comparison between *Vibrio* sp.; **(C)** *tlh* gene homology comparison between species in the *V. harveyi* group (AB271112, DQ224369.1, CP007005.1, CP006719.1); **(D)** *bla*_CARB-17_ gene homology comparison between species in the *V. harveyi* group (KJ934265.1, CP006719.1, JPTG01000491, CP009468). Note: Some accession numbers represent the genome sequences, which harbor the analyzed genes. For the accurate gene sequences, refer to the Supplementary Sequences.

With thorough bioinformatics analysis of the putative β-lactamase gene, we identified 32 CARB-like variants among the 293 available *V. parahaemolyticus* whole genome sequences in NCBI WGS database as of October 1, 2014. Apart from *V. parahaemolyticus*, CARB-like genes were found to distribute among several other *Vibrio* sp., such as *V. alginolyticus, V. harveyi, V. campbellii, V. jasicida, V. natriegens, V. owensii*, and *V. rotiferianus* after conducting the BLAST with *bla*_CARB-17_ gene in nucleotide collection (nr) database. Upon phylogenetic analysis, we found that the *bla*_CARB-17_ like genes in *V. parahaemolyticus* exhibited the lowest degree of similarity (78% homology) with that in *V. alginolyticus* (**Figure 1**). In order to compare the uniqueness of this gene with other genetic markers used to detect *V. parahaemolyticus*, we selected *tlh, atpA* and *toxR* genes within the *Vibrio* sp. and compared their genetic relatedness. The results showed that the degree of homology between *V. parahaemolyticus* and *V. alginolyticus* were respectively 85, 97, and 86% for the *tlh, atpA* and *toxR* genes; these values were higher than that of the *bla*_CARB-17_ gene, indicating that the *bla*_CARB-17_ like gene is the most divergent among these genes in *Vibrio* sp., therefore offering the highest specificity for detection.

The PCR assay designed in this study for detection of the *bla*_CARB-17_ like gene in *V. parahaemolyticus* yielded an amplified fragment of 303bp (Supplementary Figure 1). The optimal annealing temperature was determined to be 50°C after optimization (Supplementary Figure 2). The specificity of the developed PCR in this study and other published methods (Izumiya et al., 2011; Dickinson et al., 2013) were verified in parallel with different strains (**Table 2**), with results showing that PCR method based on *bla*_CARB-17_ yielded 100% specificity for *V. parahaemolyticus*, while the methods based on detecting *atpA* and *tlh* were less specific and occasionally produced false positive result. This will undoubtedly reduce the accuracy of *V. parahaemolyticus* identification and may result in incorrect clinical diagnosis. The PCR method based on *toxR* gene detection displayed similar specificity as that targeting to *bla*_CARB-17_ in this study (Kim et al., 1999). Primers targeting the *atpA* gene exhibited very high false positive rate (89%) for *V. cholerae* and 2.8% false positive rate for *V. alginolyticus*, whereas primers targeting the *tlh* gene yield 20% false positive rate for *V. alginolyticus* (**Table 2**). This indicates that the choice of these two targets is not rigorous enough in terms of detection specificity. Some of the PCR results have been displayed in Supplementary Figure 3. The different *tlh* gene variants in *V. alginolyticus* and *V. parahaemolyticus* were detrimental to the specificity of the primers. All the *tlh* genes in WGS database from *V. alginolyticus* and *V. parahaemolyticus* were included in Supplementary Sequences. In contrast, the use of *bla*_CARB-17_ specific primers did not result in any false positive detection for all the bacteria tested, and consistently maintained 100% detection specificity for *V. parahaemolyticus*. Although many molecular detection methods have been developed to identify *V. parahaemolyticus* rapidly, some do not have a satisfactory level of specificity, hindering extensive application in routine laboratory tests (Xie et al., 2005; Klein et al., 2014). In this work, we showed that the *bla*_CARB-17_ gene is a *V. harveyi* clade (including *V. parahaemolyticus*) specific gene that can be used as a novel target for identification of *V. parahaemolyticus* by using degenerated primers. Combined with other specific target genes in other *Vibrio* sp., this novel target gene may be used to design multiplex-PCR approaches to detect food contamination by *V. parahaemolyticus* rapidly. With the increasing amount data of genome sequences, more species-specific genetic markers could be mined *in silico* through bioinformatics techniques, and relieve the laborious works required for specificity testing.

## Conclusion

In this report, we used the available genome sequences in NCBI to identify a resistance gene known as *bla*_CARB-17_ like gene, which is intrinsic to *V. parahaemolyticus*. Based on the DNA sequences, a set of degenerated primers were designed to detect this major foodborne pathogen. The *bla*_CARB-17_ gene can be used as a novel *V. parahaemolyticus* detection marker, or used in combination with other markers to detect different *Vibrio* sp. simultaneously and rapidly. The specificity of *bla*_CARB-17_ gene together with its ampicillin resistance phenotype offer this detection method higher accuracy and specificity than other previously reported methods and will be of great benefit for food safety and clinical diagnosis.

## Author Contributions

RL designed and conducted the experiments and wrote the manuscript, JC initiated the project, EC designed the experiment and edited the manuscript, SC designed the experiment, supervised the project and edited the manuscript.

## Conflict of Interest Statement

The authors declare that the research was conducted in the absence of any commercial or financial relationships that could be construed as a potential conflict of interest.

## Footnotes

1 www.lynon.com

2 http://www.ncbi.nlm.nih.gov/tools/primer-blast
