# Supplementary material for: A Novel PCR-Based Approach for Accurate Identification of Vibrio parahaemolyticus
Source: Front Microbiol. 2016 Jan 28;7:44. doi: 10.3389/fmicb.2016.00044 (PMC4729947; doi:10.3389/fmicb.2016.00044)
Supplement: Supplementary file 1 [file Data_Sheet_1.PDF]

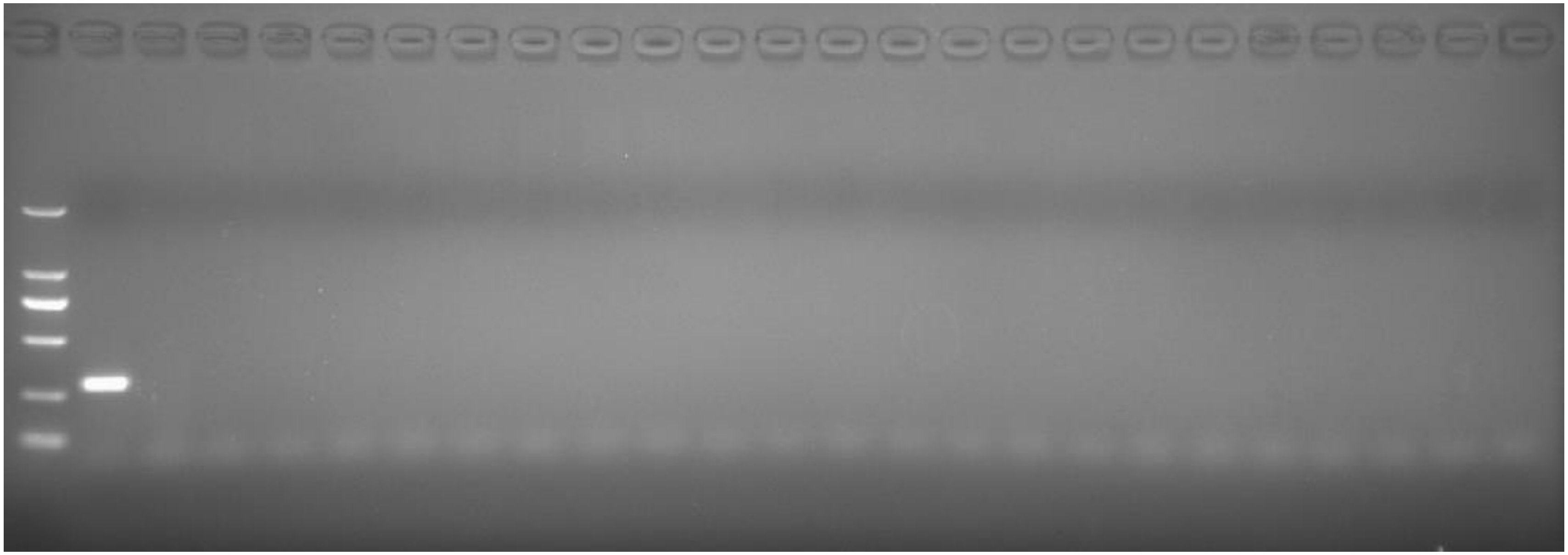

Supplementary Figure 1. Agarose gel picture of PCR with designed *bla*$_{CARB}$ primers. M stands for DL2000 DNA marker, lane 25 is negative controls (no template). Other lanes are the samples of genomic DNA templates of different strains. Lane 1, *V. parahaemolyticus* 17802; land 2, *Vibrio cholera* VC1; lane 3, *Vibrio vulnificus* ATCC27562; lane 4, *Vibrio alginolyticus* ATCC33787; lane 5, Vibrio metschnikovii V18; lane 6, *Vibrio fluvialis* ATCC33809; lane 7, *Vibrio harveyi* ATCC33842; lane 8, *Vibrio mimicus* ATCC 33653; lane 9, *Vibrio campbellii* ATCC 33865; land 10, *Vibrio natriegens* ATCC 14048; land 11, *Aeromonas caviae* A24; lane 12, *Escherichia coli* ATCC25922; lane 13, *Pseudomonas aeruginosa* PAO1; lane 14, *Salmonella typhimurium* LT2; lane 15, *Enterobacter spp*. E1; lane 16, *Citrobacter freundii* C1; lane 17, *Klebsiella pneumonia* 51; lane 18, *Proteus mirabilis* HD4; lane 19, *Myroides odoratimimus* MO12-4; lane 20, *Staphylococcus aureus* ATCC25923; lane 21, *Salmonella enteritidis* SE1; lane 22, *Salmonella derby* SD1; lane 23, *Aeromonas hydrophila* A10; lane 24, negative control.

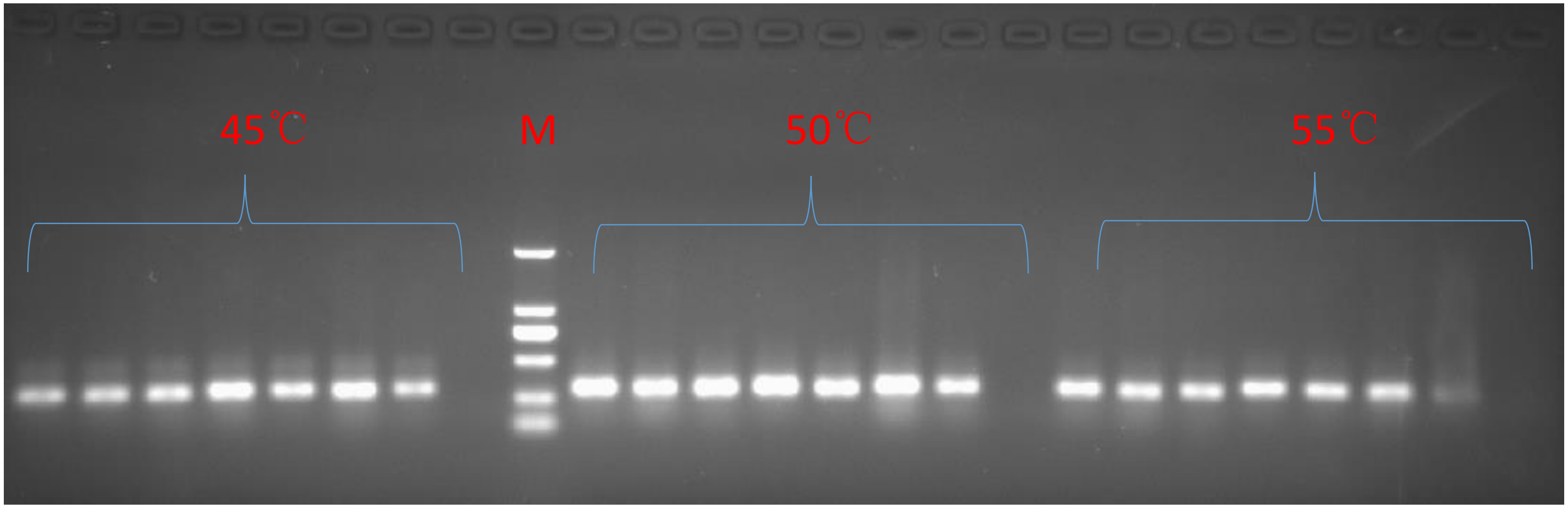

Supplementary Figure 2. Optimization of PCR condition for *bla*$_{\mathrm{CARB\text{-}17}}$ like gene amplification. M stands for DL2000 DNA marker; seven *V. parahaemolyticus* isolates were used as positive strains, the eighth lane stands for negative control.

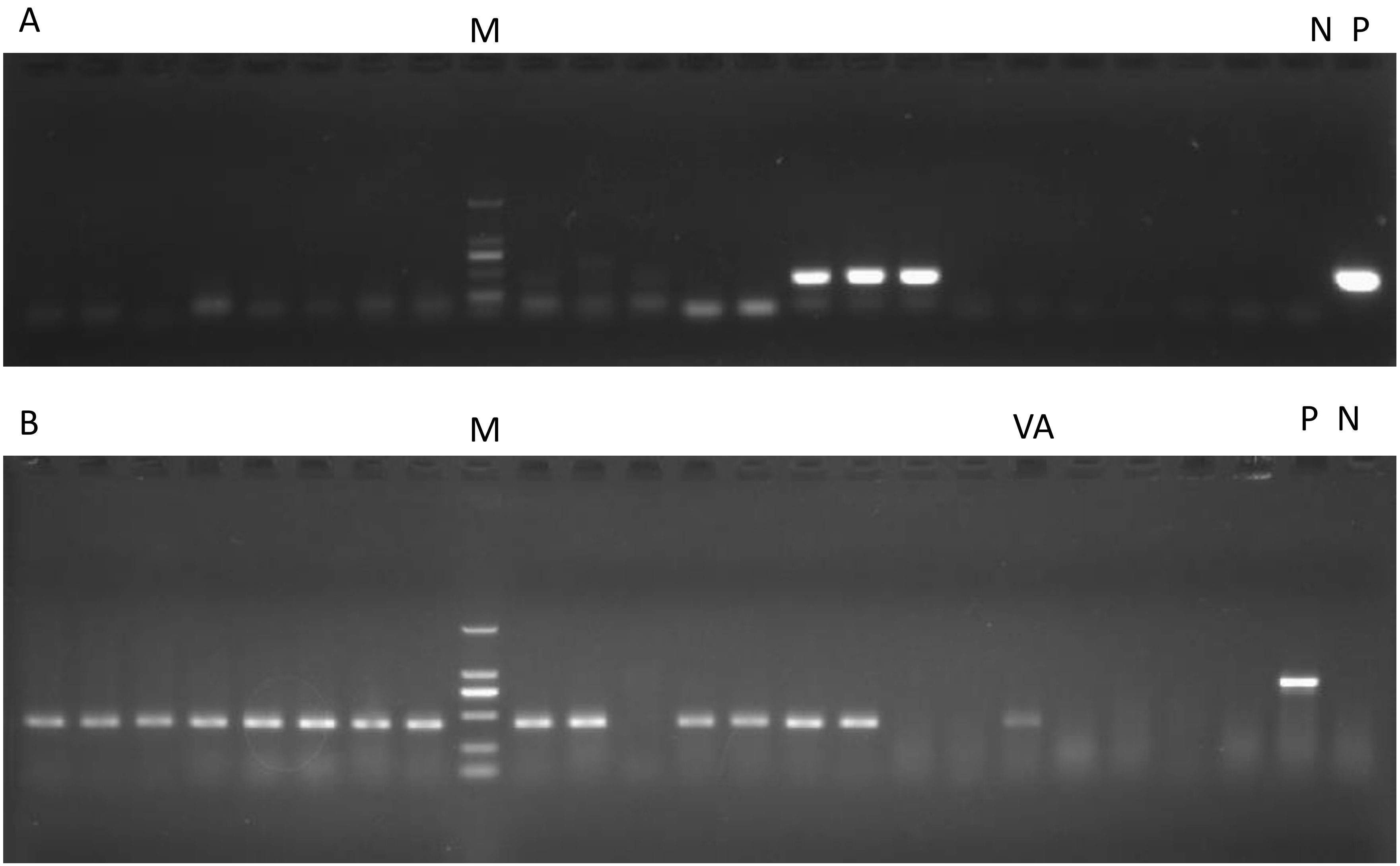
A
M
N P
B
M
VA
P N

Supplementary Figure 3. PCR result of *tlh* and *atpA* amplification using primers shown in table 1. (A) PCR result of *tlh* amplification with primers in Table 1. The DNA marker (lane M) is DL2000 (Takara,Japan). P represents the positive control of *V. parahaemolyticus* which has a 450bp PCR product. N represents the negative control of water as the template. Other samples are the *V. alginolyticus* DNA templates. Three of the 22 templates are positive in this gel. (B) PCR result of *atpA* amplification with primers in Table 1. The DNA marker ((lane M)) is DL2000 (Takara,Japan). P represents the positive control of *V. parahaemolyticus* which has a 794bp PCR product. N represents the negative control of water as the template. VA represents the *V. alginolyticus* template which has a non-specific product about 480bp. Other 21 templates are *V. cholera* templates 14 of which have non-specific product about 480bp in this gel.
