# Supplementary material for: A Novel PCR-Based Approach for Accurate Identification of Vibrio parahaemolyticus
Source: Front Microbiol. 2016 Jan 28;7:44. doi: 10.3389/fmicb.2016.00044 (PMC4729947; doi:10.3389/fmicb.2016.00044)
Supplement: Supplementary file 2 [file Data_Sheet_2.PDF]

6 atpA genes from Vibrio spp.

> Vibrio campbellii HY01 atpA, AAWP01000035
ATGCAACTTAATTCCACGGAAATTAGCGATCTGATCAAACAACGTATCGAATCTTTCGAAGTTGTTAGTGAAGCTC
GCAACGAGGGTACTATCGTATCGGTAAGCGATGGTATCATCCGCATCCACGGCCTAGCGGACGTGATGCAAGGTG
AAATGATTGAATTACCGGGTGGCCGTTATGCACTAGCACTTAACCTTGAGCGTGACTCGGTTGGTGCGGTAGTAAT
GGGCCCATATGCTGACCTTAAGGAAGGCATGAAAGTTACAGGTACTGGCCGCATTCTTGAAGTGCCAGTTGGTCCA
GAACTTCTAGGTCGCGTAGTGAACACACTAGGTGAGCCTATCGATGGTAAAGGTCCAATCGAAGCTAAATTGACTT
CGCCTGTAGAAGTAATCGCACCAGGTGTAATCGACCGTAAATCGGTAGACCAACCTGTGCAAACTGGTTACAAAT
CAGTTGACTCAATGATCCCAATCGGTCGTGGTCAGCGTGAGCTTGTAATCGGTGACCGTCAAACTGGTAAAACAGC
AATGGCGATCGATGCGATTATCAACCAGAAAGACTCTGGTATTTTCTCAATCTACGTAGCAATCGGTCAGAAAGCA
TCGACTATCGCTAACGTAGTTCGCAAACTAGAAGAGCACGGCGCGCTAGCTAACACTATCGTTGTTGTTGCATCTG
CTTCTGAATCTGCAGCGCTACAATACCTAGCGCCATACGCAGGTTGTGCGATGGGTGAATACTTCCGCGATCGCGG
TGAAGACGCACTGATTGTTTACGATGATCTATCTAAGCAAGCGGTAGCTTACCGTCAGATCTCTCTACTACTAAAA
CGTCCACCAGGCCGTGAGGCATTCCCAGGTGACGTATTCTACTTACACTCGCGTCTACTAGAGCGTGCAGCTCGTG
TAAACGAAGAGTACGTAGAGCGTTTCACTAACGGTGAAGTGAAAGGTAAGACTGGTTCTTTGACTGCTCTTCCTAT
CATCGAAACTCAAGCAGGTGACGTTTCAGCATTCGTACCGACTAACGTAATCTCGATTACCGATGGTCAGATCTTC
CTACAAACTGAGCTATTCAACGCGGGTGTACGCCCAGCCGTTGACCCAGGTATCTCAGTATCTCGTGTAGGTGGTT
CAGCTCAGACGAAAATCATCAAGAAGCTATCTGGCGGTATCCGTACTGCACTAGCTCAGTACCGTGAACTAGCGG
CATTTGCACAGTTCTCGTCTGACCTTGATGAAGCGACGAAGAAACAGCTAGACCACGGTCAAAAAGTTACAGAAC
TAATGAAGCAGAAGCAGTACGCTCCAATGTCTGTATTTGACCAAGCACTAGTAATCTTCGCGGCAGAGCGCGGCTA
TTTAGCAGATGTTGAACTAAACAAACTGCTAGATTTCGAAGCGGCTCTACTATCGTACGCTCGCGGTCAATACGCT
GAATTTGCAGCTGAGATCGACAAGACGGGTGCATACAACGATGAAGTTGAAGCTCAGTTGAAGAAACTGACTGAC
GACTTCGTAGCAACCCAAACTTGGTAA

> Vibrio harveyi ATCC BAA-1116 atpA, CP006605.1
ATGCAACTTAATTCCACGGAAATTAGCGATCTAATCAAACAGCGTATCGAATCTTTCGACGTTGTTAGTG
AAGCTCGCAACGAGGGTACTATCGTATCGGTGAGCGATGGTATCATCCGCATCCACGGCCTAGCGGACGT
GATGCAAGGTGAAATGATTGAATTACCGGGTGGCCGTTATGCACTAGCACTTAACCTTGAGCGTGACTCG
GTTGGTGCGGTAGTAATGGGCCCATATGCTAACCTTAAGGAAGGCATGAAAGTTACAGGTACTGGCCGCA
TTCTTGAAGTGCCAGTTGGTCCAGAACTTCTAGGTCGCGTAGTGAACACACTAGGTGAGCCTATTGATGG
TAAAGGTCCAATCGAAGCTAAATTGACTTCGCCTGTAGAAGTAATCGCACCAGGTGTAATCGCCCGTAAA
TCGGTAGACCAACCTGTGCAAACTGGCTACAAATCAGTTGACTCAATGATCCCAATCGGTCGTGGTCAGC
GTGAGCTTGTAATCGGTGACCGTCAAACTGGTAAAACAGCAATGGCGATCGATGCGATTATCAACCAGAA
AGACTCTGGTATTTTCTCAATCTACGTAGCAATCGGTCAGAAAGCATCGACAATCGCTAACGTAGTTCGC
AAACTAGAAGAGCACGGCGCACTAGCTAACACTATCGTTGTTGTTGCATCTGCTTCTGAATCTGCAGCGT
TACAATACCTGGCGCCATACGCAGGTTGTGCGATGGGTGAATACTTCCGCGATCGCGGTGAAGACGCACT
GATTGTTTACGATGATCTATCTAAGCAAGCGGTAGCTTACCGTCAGATCTCTCTACTACTAAAACGTCCA
CCAGGCCGTGAGGCATTCCCAGGTGACGTATTCTACTTACACTCGCGTCTACTAGAGCGTGCAGCTCGTG
TAAACGAAGAGTACGTAGAGCGTTTCACTAACGGTGAAGTGAAAGGTAAGACTGGTTCTTTGACTGCTCT
TCCTATCATCGAAACTCAAGCAGGTGACGTTTCAGCATTCGTACCGACTAACGTAATCTCGATTACCGAT
GGTCAGATCTTCCTACAAACTGAGCTATTCAACGCGGGTGTACGCCCAGCCGTTGACCCAGGTATCTCAG
TATCTCGTGTAGGTGGTTCAGCTCAGACGAAAATCATCAAGAAGCTATCTGGCGGTATCCGTACTGCACT
AGCTCAGTACCGTGAACTAGCGGCATTTGCACAGTTCTCGTCTGACCTTGATGAAGCGACGAAGAAACAG
CTAGACCACGGTCAAAAAGTTACAGAACTAATGAAGCAGAAGCAGTACGCTCCAATGTCTGTATTTGACC
AAGCACTAGTAATCTTCGCGGCAGAGCGCGGCTATTTAGCAGATGTTGAACTAAACAAACTGCTAGATTT
CGAAGCGGCTCTACTATCGTACGCTCGCGGTCAATACGCTGAATTTGCAGCTGAGATCGACAAGACGGGT
GCATACAACGATGAAGTTGAAGCTCAGTTGAAGAAACTGACTGACGACTTCGTAGCAACCCAAACTTGGTAA

> Vibrio alginolyticus NBRC 15630 atpA, BATK01000022.1
ATGCAACTTAATTCCACGGAAATTAGCGATCTGATCAAACAACGTATCGAATCTTTCGAA

GTTGTTAGTGAAGCTCGCAACGAGGGTACTATCGTATCGGTAAGCGATGGTATCATCCGC
ATCCACGGCCTAGCGGACGTGATGCAAGGTGAAATGATTGAATTACCGGGTGGTCGTTAT
GCACTGGCACTTAACCTTGAGCGTGACTCGGTTGGTGCGGTTGTAATGGGCCCATATGCT
GACCTTAAGGAAGGCATGAAAGTTACAGGTACTGGCCGCATTCTTGAAGTGCCAGTTGGT
CCAGAACTACTAGGCCGCGTAGTAAATACGCTGGGTGAGCCTATCGATGGTAAAGGTCCA
ATCGAAGCGAAAATGACTTCGCCTGTAGAAGTGATCGCACCAGGTGTAATCGACCGTAAA
TCGGTAGACCAACCTGTGCAAACTGGTTATAAGTCAGTTGACTCAATGATCCCAATCGGT
CGTGGTCAGCGTGAGCTTATCATCGGTGACCGTCAGATTGGTAAAACAGCACTGGCGATC
GACGCGATCATCAACCAGAAAGACTCTGGTATTTTCTCTATCTACGTAGCAATCGGTCAG
AAAGCATCGACTATCGCTAACGTTGTTCGCAAACTAGAAGAGCACGGCGCACTACAAAAT
ACTATCGTTGTAGTTGCATCAGCTTCTGAATCTGCAGCGCTTCAATACCTAGCGCCTTAC
GCAGGTTGTGCGATGGGTGAATACTTCCGCGATCGCGGTGAAGACGCACTGATTGTTTAC
GATGATCTATCTAAGCAAGCGGTAGCTTACCGTCAGATCTCTCTACTACTTAAACGTCCA
CCAGGCCGTGAGGCATTCCCAGGTGACGTATTCTACTTACACTCGCGTCTACTAGAGCGT
GCAGCTCGTGTAAACGAAGAGTACGTAGAGCGTTTCACTAACGGTGAAGTGAAAGGTAAG
ACTGGTTCTTTGACTGCTCTTCCTATCATCGAAACTCAAGCAGGTGACGTTTCAGCATTC
GTACCGACTAACGTAATCTCGATTACCGATGGTCAGATCTTCCTACAAACTGAGCTATTC
AACGCTGGTGTTCGTCCTGCTGTTGACCCTGGTATCTCAGTATCTCGTGTAGGTGGTTCA
GCTCAGACGAAAATCATCAAGAAACTATCAGGTGGTATCCGTACAGCACTAGCTGCATAC
CGTGAACTAGCGGCATTTGCTCAGTTCTCTTCTGACCTTGATGACGCAACGAAGAAACAG
CTAGACCATGGTCAAAAAGTTACAGAACTAATGAAGCAGAAGCAGTACGCTCCAATGTCT
GTATTTGACCAAGCTCTAACTATCTTCGCGGCAGAGCGCGGTTACCTTGATGATGTAGAA
CTTAACAAAGTTCTAGATTTCGAGGCGGCTCTACTATCGTACGCTCGCGGTCAATACGCT
GAACTAGCAGCTGAGATCGACAAGTCTGGCGCTTACAACGATGAAATCGAAGCTCAGTTG
AAGAAACTGACTGACGACTTCAAAGCAACCCAAACTTGGTAA

> Vibrio parahaemolyticus strain NSTH33 atpA, KF886608.1
ATGCAACTTAATTCCACGGAAATTAGCGATCTGATCAAACAGCGTATCGAATCTTTCGAAGTTGTTAGCG
AAGCTCGCAACGAGGGTACTATCGTATCGGTAAGCGATGGTATCATCCGCATCCACGGCCTAGCGGACGT
GATGCAAGGTGAAATGATTGAATTACCGGGTGGCCGTTATGCACTGGCACTTAACCTTGAGCGTGACTCG
GTTGGTGCGGTAGTAATGGGCCCATATGCTGACCTTAAGGAAGGCATGAAAGTTACAGGTACTGGCCGCA
TTCTTGAAGTGCCAGTTGGTCCAGAACTACTAGGCCGCGTAGTAAACACGCTGGGTGAGCCTATCGATGG
TAAAGGTCCAATCGAAGCGAAATTGACTTCACCTGTAGAAGTTATCGCACCAGGTGTAATCGACCGTAAA
TCGGTAGACCAACCTGTACAAACTGGCTACAAATCAGTTGACTCAATGATCCCTATCGGTCGTGGTCAGC
GTGAGCTTGTAATCGGTGACCGTCAGACTGGTAAAACAGCAATGGCGATCGATGCGATCATCAACCAGAA
AAACTCTGGTATTTTCTCAATCTACGTAGCAATCGGTCAGAAAGCATCGACTATCGCTAACGTAGTTCGC
AAACTAGAAGAGCACGGCGCGCTAGCAAACACTATCGTTGTTGTTGCATCTGCTTCTGAATCTGCAGCGC
TACAATACCTAGCGCCATATGCAGGTTGTGCGATGGGCGAATACTTCCGCGATCGCGGCGAAGACGCACT
GATTGTTTACGATGATCTATCTAAACAAGCGGTAGCTTACCGTCAGATCTCTCTACTACTAAAACGTCCA
CCAGGCCGTGAGGCATTCCCAGGTGACGTATTCTACTTACACTCACGTCTACTAGAGCGTGCAGCTCGTG
TAAACGAAGAGTACGTAGAGCGTTTCACTAACGGTGAAGTGAAAGGTAAGACTGGTTCTTTGACTGCTCT
TCCTATCATCGAAACTCAAGCAGGTGACGTTTCAGCATTCGTACCGACTAACGTAATCTCGATTACCGAT
GGTCAGATCTTCCTACAAACTGAGCTATTCAACGCAGGTGTACGTCCAGCGGTTGACCCAGGTATCTCAG
TATCTCGTGTAGGTGGTTCAGCTCAGACGAAAATCATCAAGAAACTATCAGGTGGTATTCGTACAGCACT
AGCTGCATACCGCGAACTAGCAGCATTTGCTCAGTTCTCTTCTGATCTTGATGAAGCAACGAAGAAACAG
CTAGACCATGGTCAAAAAGTTACAGAACTAATGAAGCAGAAGCAGTACGCTCCAATGTCTGTATTTGACC
AAGCACTAGTTATCTTCGCGGCAGAGCGCGGTTACCTTGACGATGTAGAATTGAACAAAGTTCTAGATTT
CGAGGCGGCTCTACTATCGTACGCTCGCGGTCAATACGCTGAATTAGCAGCTGAGATCGACAAGTCTGGT
GCTTACAACGATGAAATCGAAGCTCAGTTGAAGAAACTGACTGACGACTTCAAAGCAACCCAAACTTGGT
AA

> Vibrio vulnificus strain NSTH34 atpA, KF886609
ATGCAACTTAATTCCACGGAAATTAGCGATCTAATTAAACAGCGTATTGAATCTTTCAACGTTGTTAGTG
AAGCTCGCAATGAAGGTACTATCGTATCGGTAAGCGACGGTATCATTCGCATTCACGGCCTAGCGGACGT
GATGCAAGGTGAAATGATTGAATTACCGGGTGGCCGTTATGCACTAGCACTTAACCTTGAGCGTGACTCG
GTTGGTGCGGTAGTAATGGGTCCATATGCTGACCTTAAGGAAGGCATGAAAGTAACAGGTACTGGTCGTA
TTCTTGGAGTGCCAGTTGGTCCTGAGCTATTGGGTCGTGTAGTGAACACACTAGGTGAGCCAATTGACGG
TAAAGGTCCAATTGAAGCGAAATTAACGTCACCTGTTGAAGTGATTGCACCAGGTGTAATCGACCGTCAG
TCGGTTGATCAACCTGTACAAACTGGTTATAAGTCAGTTGACTCGATGATCCCTATCGGTCGTGGTCAGC
GTGAACTTATCATCGGTGACCGTCAGACTGGTAAAACAGCGATGGCGATCGATGCGATCATCAACCAGAA
AAACTCTGGTATTTTCTCTATCTACGTAGCGATTGGTCAGAAAGCTTCTACTATCGCCAACGTAGTGCGT
AAACTGGAAGAGCACGGTGCTCTGAAGAACACAATTGTGGTTGTGGCTTCAGCATCTGAATCTGCTGCAC
TGCAATACCTAGCGCCATACGCAGGTTGTGCAATGGGTGAATACTTCCGTGATCGCGGCGAAGACGCACT
GATTGTTTATGATGATCTATCAAAGCAAGCGGTCGCTTACCGTCAGATCTCTCTATTGCTAAAACGCCCA
CCAGGCCGTGAAGCATTCCCAGGTGACGTATTCTACCTTCACTCTCGTCTACTAGAGCGTGCAGCTCGTG
TAAACGCAGAGTATGTAGAGCGTTTCACTAACGGTGAAGTGAAAGGTAAGACAGGTTCTTTAACCGCTCT
TCCTATCATCGAAACTCAGGCTGGTGATGTTTCTGCATTCGTACCAACCAACGTAATCTCGATCACCGAT
GGTCAGATCTTCCTACAAACTGAGCTATTCAACGCGGGTGTACGTCCAGCGGTTGACCCAGGTATCTCAG
TATCTCGTGTAGGTGGTTCTGCACAAACGAAAATCATCAAGAAGTTGTCTGGTGGTATCCGTACAGCACT
AGCGGCTTACCGTGAACTTGCAGCGTTTGCTCAGTTCTCTTCGGATCTTGATGAAGCGACTAAGAGACAG
TTGAACCACGGTCAAAAAGTAACTGAACTGATGAAGCAGAAGCAGTACGCTCCGATGTCAGTGTTTGACC
AGGCTCTAACTATCTTTGCGGCAGAGCGTGGCTACCTAAGCGATATCGAACTTTCTAAAGTTCTTGATTT
CGAAGCAGCTCTACTATCGTACGCTCGCGGTCAATACGCTGAATTAGCAGCTGAGATCGACAAGACGGGT
GCTTACAACGATGAAATCGAAGCTCAGTTGAAGAAACTGACTGACGACTTCAAAGCAACCCAAACTTGGT
AA

> Vibrio cholerae strain NSTH36 atpA, KF886611
ATGCAACTTAATTCCACGGAAATTAGCGATCTGATCAAACAACGTATTGAATCTTTCAACGTTGTTAGTG
AAGCTCGCAACGAAGGTACTATCGTATCGGTAAGCGACGGTATCATTCGTATTCACGGCCTTGCAGATGT
TATGCAAGGTGAAATGATTGAATTACCGGGCAACCGTTATGCACTGGCACTGAACCTTGAGCGTGACTCG
GTCGGTGCGGTAGTAATGGGTCCATACGCGGATCTGCGTGAAGGCATGAAAGTAACAGGTACTGGCCGTA
TTTTGGAAGTTCCTGTTGGTCCTGAGCTGCTAGGTCGTGTAGTTAACACTCTGGGTGAGCCGATTGATGG
TAAAGGCCCAATCGGTGCAAAACAAACTTCACCAGTTGAAGTGATCGCACCGGGTGTTATCGATCGTAAG
TCGGTTGATCAACCTGTTCAAACTGGTTACAAATCAGTGGACTCGATGATTCCAATCGGCCGTGGCCAGC
GTGAGCTGATCATCGGTGACCGTCAAACAGGTAAAACTGCGTTGGCGATTGATGCGATCATCAACCAGAA
AAACTCTGGTATTTACTCTATCTATGTTGCTATCGGCCAGAAAGCGTCAACCATTGCGAACGTAGTTCGT
AAATTGGAAGAGCACGGCGCACTGAAAAACACCATTGTTGTGGTGGCTTCAGCGTCTGAATCTGCTGCAC
TGCAATACCTCGCGCCTTACTCAGGCTGTGCGATGGGTGAATACTTCCGCGATCGCGGTGAAGATGCGCT
GATTGTATACGATGACCTGTCTAAACAGGCTGTTGCTTACCGTCAAATCTCACTGCTTCTGCGTCGTCCA
CCAGGCCGTGAAGCTTTCCCTGGCGACGTGTTCTACCTCCACTCTCGTCTACTAGAGCGTGCCGCTCGTG
TAAACGAAGAGTACGTAGAGCGTTTCACCAAGGGTGAAGTGAAAGGTAAGACCGGTTCTCTAACCGCTCT
GCCAATCATCGAAACTCAAGCTGGTGACGTATCGGCATTCGTACCGACCAACGTAATTTCGATCACTGAT
GGTCAGATCTTCCTACAAACTGAACTGTTCAACGCGGGCGTACGTCCAGCGGTTGACCCAGGTATCTCAG
TTTCTCGTGTAGGTGGTGCAGCGCAAACCAAGATCGTTAAGAAGCTCTCTGGTGGTATCCGTACTGCTCT
GGCTGCTTACCGTGAATTGGCGGCGTTTGCTCAGTTCTCTTCGGATCTAGATGAAGCGACCAAGCGTCAG
CTGACTCACGGTCAGAAAGTGACTGAACTGATGAAGCAGAAACAGTATGCACCAATGTCTGTCTTTGATC
AGGCTCTGGTGATCTTTGCTGCGGAACGTGGCTATCTGACAGACGTAGAACTGAACAAGGTTCTCGACTT
CGAAGCTGCTCTACTATCGTATGCTCGTGCTCATTACGCTGAATTGGCAGCGCAAATCGACAAGACGGGT
GCTTACAACGATGAGATCGAAGCTCAGCTGAAGAAGCTGGTTGACGATTTCAAAGCAACCCAAACTTGGT
AA

4 tlh genes from Vibrio harveyi group

> Vibrio campbellii 53 tlh, AB271112
ATGAATAAGACCATTACGTTACTTAGTGCATTATTACTACCATTAAATCTTGCTCACGCAGCCGACTCAT
CAGAGCCTTCTTACCAGCTAAATGCCTCAGAAGTGAGAAGCGCACAACAAAAGCAAACATACACCTACGT
ACGATGCTGGTATCGAACTAGTTATTCACATGATGACCCAGAAACCGACTGGGAGTGGGCAGAAAATCCA
GATGGCAGTTATTTCACTATCGAAGGCTATTGGTGGAACGCACTCTCGTTTAAAAACATGTTCTATACCA
ATACATCACAAAGCGTGATCAAGCAGCGCTGTGAACAAACACTCGATCTTGCGAACGAAAATGCGGACAT
TACCTACTTCGCTGCCGATAACCGTTGGTCATATAACCACTCGATTTGGAGTAATGATCCTGTGATGCAG
CCAGATCAAATCAACAAGGTCGTGGCATTGGGTGACAGCTTGTCGGATACCGGGAACATCTTTAACGCAT
CACAATGGCGATTCCCGAATCCAAACAGCTGGTTCTTAGGGCATTTCTCTAACGGGTTTGTATGGACAGA
ATACATTGCTCAAGCGAAGAATTTACCGCTATACAACTGGGCGGTGGGTGGCGCTGCTGGCGAAAATCAA
TACATCGCGCTAACGGGCGTTGGAGAACAAGTCTCTTCTTACCTTGCTTATATGCAGCTGGCGAAAAACT
ACAACCCTGCCAATACCCTGTTCACCCTTGAGTTTGGATTGAATGACTTCATGAACTACAACCGCAGTGT
TCCAGAAGTAAAAGCAGATTATTCGGAGGCCTTGATAAAACTGAGTGATGCTGGCGCAAGAAACTTCTTA
CTCATGACCCTACCTGACGCGACCTATGCGCCTCAGTTCAAATATTCTAGCCAAGAAGAAATCGACACAA
TCCGTGCGAAAATCCTTGAGATGAACGAATTTATCAAAGCGCAGGCAGCTTACTACACTGCACAGGGGCT
CAACATTACGTTGCACGACACCCATGCACTCTTTGCTAGCTTGACAGCTAACCCTGAACAACATGGCTTC
AGTAACGCAACGGAAGCATGCCAAGACATCAATCGCTCGTCTGCTGCTGATTACTTGTACAAACATTCGC
TGCGTTCTGAATGTGCGTCCGTTGGGTCAGATAAATTTGTATTCTGGGATGTTACCCACCCGACCACAGC
AACACATCGCTACGTGGCTGAGGATATGCTAGAAACCACTGACCAACTTTCGAATCATCCATTCTAA

> Vibrio harveyi strain VIB 647 tlh, DQ224369.1
ATGAATAAAACTATTACGTTACTTAGTGCATTATTACTACCACTAAGTTTTGCTCACGCTGCCGAGCCAA
CATTGTCTCCAGAGATGGTCAGCGCCTCTCAAGTAAGAAGCGCGCAAGCGAAACAAACTTACACTTATGT
CCGCTGCTGGTACCGCACCAGTTATTCAAAAGATGAACCTGCGACCAATTGGGAATGGGCAGAAAATCCA
GACGGCAGTTACTTCACGCTTGATGGCTACTGGTGGAGTTCGGTTTCTTTCAAGAACATGTTCTACACAG
ACACACCGCAAAGTGTTATCAAGCAACGTTGTGAGCAAACTCTGGACCTAGCAAATGAAAACGCTGACAT
CACCTTCTTTGCAGCCGATAACCGTTTCTCCTACAACCATACTATCTGGAGCAACGACCCTGTCATGCAG
CCAGACCAAATCAACAAGGTCGTAGCATTGGGTGACAGCTTGTCTGATACAGGCAACATCTTTAATGCAT
CACAATGGCGATTCCCGAATCCAAATAGCTGGTTCTTGGGACACTTCTCAAACGGTTTTGTGTGGACTGA
GTACATTGCTCAAGCGAAAAACTTACCGCTATACAACTGGGCTGTAGGCGGCGCGGCAGGCGAAAACCAA
TACATCGCTCTGACTGGTGTAGGTGAGCAAGTTTCCTCTTACTTGGCATATGCGAAATTAGCGAAAAACT
ACAAGCCTGCTAATACCCTGTTTACCCTTGAGTTTGGTCTAAATGACTTCATGAACTACAACCGTAGCGT
GCCAGAAGTGAAATCAGACTACGCGGAAGCCTTGATTAAACTGACCGATGCAGGTGCGAAGAACTTGTTG
TTGATGACACTACCAGATGCAACACGTGCACCACAGTTTACCTACTCGACTCAAGAAGAAATCAACAAGA
TCCGCGCGAAGATCGTGGAAATGAATGAGTTCATCAAAGCACAAGCGGCGTATTACACTGCACAAGGCTA
CAACGTTACCTTGTACGATACGCATGCACTGTTTGAAAGCTTAACAGCAAATCCAGAGCAACACGGTTTT
GTAAACGCGAGCCAAGCTTGCCAAGACATCAACCGCTCTTCATCGGTGGATTACCTATACCATCATGCAT
TGCGTTCTGAGTGTGCGTCTTCTGGCTCTGATAAGTTTGTATTCTGGGACGTAACGCACCCGACCACAGC
AACACACCACTACGTGGCAGAAAAAATGCTAGAAAGTACGAATCAATTGTCAAACCATCCTTTCTAA

> Vibrio parahaemolyticus UCM-V493 tlh, CP007005.1
ATGAAAAAAACAATCACACTATTAACTGCATTACTCCCGCTTGCTTCTGCAGTTGCCGAA
GAGCCAACCTTATCACCAGAAATGGTTTCAGCGTCTGAAGTGATCAGCACGCAAGAAAAC
CAAACCTATACCTATGTTCGCTGTTGGTATCGCACCAGCTACTCGAAAGATGATCCGGCG
ACCGATTGGGAATGGGCAAAAAACGAAGATGGTAGCTACTTCACCATTGACGGCTACTGG
TGGAGCTCCGTTTCATTTAAAAACATGTTCTACACCAACACGTCGCAAAACGTTATCCGT
CAGCGTTGTGAAGCAACATTAGATTTGGCGAACGAGAACGCAGACATTACGTTCTTCGCC
GCTGACAATCGCTTCTCATACAACCACACGATCTGGAGCAACGACGCAGCAATGCAGCCA
GATCAAATCAACAAAGTGGTTGCACTCGGTGACAGCTTGTCTGATACAGGCAACATCTTT
AACGCATCACAATGGCGCTTCCCTAACCCGAACAGCTGGTTCTTAGGTCACTTCTCCAAC
GGTTTTGTGTGGACAGAATACATTGCCAAAGCGAAGAACCTTCCGCTCTACAACTGGGCA

GTTGGCGGCGCGGCTGGTGAGAACCAATACATCGCGCTAACAGGGGTTGGTGATCAAGTT
TCTTCGTACTTAACCTACGCAAAACTGGCGAAGAACTACAAACCAGCAAACACCTTGTTT
ACGCTTGAGTTTGGTTTGAATGACTTCATGAACTACAACCGTGGCGTTCCAGAAGTGAAA
GCAGATTATGCAGAAGCACTGATTCGTTTGACGGACGCAGGTGCGAAGAACTTCATGTTG
ATGACACTGCCAGACGCGACGAAAGCGCCTCAGTTTAAGTACTCAACACAAGAAGAGATC
GACAAAATTCGTGCGAAAGTGCTTGAGATGAACGAGTTCATCAAGGCACAAGCGATGTAC
TACAAAGCGCAAGGTTACAACATCACGTTGTTTGATACTCACGCCTTGTTCGAGACGCTA
ACTTCTGCGCCAGAAGAGCACGGTTTCGTGAACGCGAGTGATCCTTGTTTGGACATCAAC
CGCTCATCGTCTGTCGATTACATGTACACCCACGCATTGCGCTCTGAGTGTGCGGCGTCT
GGTGCTGAGAAATTTGTGTTCTGGGATGTCACGCACCCAACAACAGCAACTCACCGCTAT
GTTGCAGAGAAAATGCTAGAAAGTAGCAACAACTTAGCCGAGTACCGCTTCTAA

>Vibrio alginolyticus NBRC 15630 tlh, CP006719.1
ATGATGAAAAAAACAATCACACTATTAACTGCATTACTTCCGCTTGCCTCCGCCATTGCA
GAGGAGCCAACTTTATCACCGGCAATGGTTTCTGCGGCTGAAGTGGTTAGCGCGCAAGAA
AATCAAACTTATACGTACGTTCGATGCTGGTATCGTACGAGCCACTCTAAGGACGATGCG
GCTACCGATTGGAAATGGGCAAAAAACCAAGATGGTAGTGACTTCACTATCGATGGTTAT
TGGTGGAGCTCTGTCTCATTTAAAAACATGTTCTACACCAATACCTCACAAAACGTAATT
CGCCAGCGTTGTGAAGAAACACTAGATCTAGCGAACGAGAACGCAGACATTACATTTTTT
GCTGCTGACAATCGTTATTCTTATAACCACACGATCTGGAGCAATGATGCTGCAATGCAA
CCAGATCAAATCAACAAGGTAGTGGCTTTGGGCGATAGCTTGTCTGATACAGGCAACATC
TTTAACGCGTCCCAATGGCGCTTCCCTAACCCGAATAGCTGGTTCTTGGGTCACTTCTCA
AACGGTTTTGTTTGGACGGAATACGTTGCTAAAGCCAAAAACTTACCGCTATACAACTGG
GCAGTTGGTGGCGCAGCAGGTGAAAACCAATACATCGCGCTAACTGGTGTCGGCGAACAA
GTTTCTTCTTACTTAACCTACACAAAGCTGGCGAAGAACTACAACCCTGCTAACACATTG
TTTACGTTAGAGTTTGGTTTAAATGATTTCATGAACTACAACCGTAGCGTGCCGGAAGTA
AAAGCGGATTACGCTGAAGCTCTGATTCGTTTAACAGACGCTGGCGCGAAGAACTTCATG
TTGATGACACTGCCTGATGCAACCAAAGCACCACAGTTCAAATACTCAACACAGGAAGAA
ATTGAAACGATTCGTGCGAAAGTATTGAAGATGAACGAGTTCATCAAAGCACAAGCGATG
TACTACAAAGCCCAAGGCTACAACATCGCATTGTTTGATACACACGCACTGTTTGAGAAG
TTAACCTCGGCACCAGAAGAACACGGTTTCGTAAATGCAAGCGACCCTTGCTTAGACATC
AACCGTTCATCCTCTGTTGACTACATGTACACTCACTCATTACGTTCTGAATGTGCAGCA
TCTGGCGCAGATAAGTTCGTGTTCTGGGATGTGACTCACCCAACCACAGCAACGCATCGT
TATGTGGCTGAAAAAATGTTAGAAAGCAGCAATAACTTAGAAGAGTTTCGCTTTTAA

4 carb genes from Vibrio harveyi group

> Vibrio parahaemolyticus CARB17, KJ934265.1
ATGAAAAAGTTATTCCTGTTGGTTGGGCTGATGGTTTGCTCAACTGTTAGTTACGCCTCCAAATTAAACG
AAGACATCTCCCTCATCGAGAAACAAACATCTGGGCGAATTGGAGTGTCAGTCTGGGATACACAAACGGA
CGAGCGTTGGGATTATCGCGGAGACGAACGCTTCCCATTAATGAGCACATTCAAAACGTTAGCGTGTGCC
ACCATGCTAAGCGACATGGACAGCGGCAAACTCAACAAAAATGCCACAGCGAGAATCGATGAACGCAATA
TTGTGGTTTGGTCTCCGGTGATGGATAAACTGACTGGACAAAGCACACGTATCGAACACGCTTGTGAAGC
CGCCATGTTGATGAGCGACAACACCGCCGCGAACTTAGTGCTAAATGAAATTGGTGGTCCTAAAGCGGTC
ACACTGTTTTTGCGCTCTATTGGCGACAAAGCAACGCGACTTGACCGATTGGAACCCCGTTTGAATGAAG
CAAAACCGGGCGACAAGCGAGATACCACAACGCCTAACGCCATGGTAAACACCCTACATACCTTGATGGA
AGATAACGCCCTATCTTACGAGTCACGCACACAGCTGAAAATCTGGATGCAAGACAACAAAGTATCGGAT
TCGCTCATGCGCTCTGTTCTGCCAAAAGGCTGGTCGATTGCAGACCGCTCTGGCGCAGGTAACTACGGTT
CACGCGGCATTAGCGCGATGATCTGGAAAGACAACTACAAGCCAGTTTACATCAGTATTTACGTCACAGA
CACCGACCTTTCGCTTCAAGCTCGCGATCAACTGATCGCGCAAATCAGCCAACTGATTTTAGAGCACTAC
AAAGAAAGTTAA

>Vibrio alginolyticus NBRC 15630 carb, CP006719.1

ATGAAAAAGTTATTTTTGTTGGCTGGGCTAATGGTTTGCTCAACCCTAAGTTACGCGTCC
CAACTGAATGAGGACATTTCTCTCCTCGAGCAACAAACCTCAAGCAGAATTGGGGTGTCA
GTTTGGGATACCCAAGCAGACGAGCGTTGGGATTACCGTGGCGATGAACGCTTCCCACTC
ATGAGCACATTCAAAACCCTAGCTTGTGCCAAAATGCTAAGTGATATGGACAGCGGTAAG
CTAAGTAAAAATGCGACCGCAAAAGTCGATGAGCGCAGTATCGTTGTATGGTCTCCAGTG
ATGGATAAGCTCGCAGGCCAAAACACACGCATAGAGCACGCGTGTGAAGCCGCTATGTTA
ATGAGTGATAATACTGCAGCCAACTTGGTATTAAATGAAATTGGCGGCCCTAAAGCCGTA
ACAATGTTTCTGCGAACAATTGGAGATAAAGCAACGCGCCTAGACAGAATAGAACCTCGC
TTGAACGAAGCCACACCAGGCGACAGCCGCGATACAACCACACCTAACGCCATACTAAAC
ACTCTGCGAACCTTGATCGAAGGCGAAACGTTGTCTTATGAGTCTCGTGTACAATTAAAA
ATCTGGATGCAAGACAACAAAGTTTCAGACTCGTTAATGCGTTCTGTATTACCAACAGGT
TGGTCTATTGCAGACCGTTCTGGTGCGGGTGGTCATGGTTCGCGTGGCATTAACGCGATT
ATATGGAAGGAAAATCATCGACCTGTTTACATTAGTATTTATGTCACAGAAACCGAGCTC
TCACTTCAAGCTAGGGACCAGCTCGTTGCGCAGATAAGTCAGTTGATTTTACAAAAGTAC
AAAGACAATTAA

>Vibrio campbellii ATCC BAA-1116 carb, JPTG01000491
ATGAAAAAGCTATTTCTGCTACTTGGATTGTTAGCCTGTTCGAGTGCAACGTACGCCGCCAAACTTAATGAAGACA
TCTCCGCGATTGAAGAGCATATTTCGGGTCGAATCGGAGTGTCTGTCTGGGATACTCAAACCGATGAACATTGGGA
CTATAGAGGCGACGAGCGCTTCCCGATGATGAGCACATTCAAAACGCTTGCATGTGCAACGATGCTTAGTGACATG
GATAATGAAAAGCTCGATAAAAACGCCACTGCCAAAGTAGAAGAGCGCAATATGGTGGTTTGGTCACCAGTAATG
GATCGTATGGCAGGTCAAACAACGCGTATTGAACATGCTTGTGAAGCCGCAATGCTGATGAGCGACAATACCGCA
GCCAACATTGTTTTGCGTAGCATTGGGGGTCCACGCGGTGTCACTACGTTCTTGCGTTCTATCGGTGATAAAGCAA
CGCGTCTTGACCGTTTTGAACCAAGACTGAACGAGGCGAACCCGGGCGACAAGCGAGACACCACAACACCTAATG
CCATGGTAAATACGCTGCATACGTTGTTAGAAGGTGATGCGCTATCCTACGAATCACGCATTCAATTAAAAATCTG
GATGCAGGATAACAAAGTCTCAGATTCTTTGATGCGCTCTGTGTTACCAAAGGGTTGGTCGATTGCCGATCGTTCT
GGTGCAGGGGGCTTTGGATCACGCGGCATCACGGCAATGATCTGGAAAGAGAATCATAAACCGGTCTACATCAGC
ATTTACATTACTGAAACCGATTTATCCTTGCAGGCGCGCGATCAGGTTATCGCTCAAGTGAGCCAGTTAATACTGG
ATGAGTACAATACTATTTAA

> Vibrio harveyi strain ATCC 33843, CP009468
ATGAAAAAGCTATTTCTGCTGCTCGGATTGTTAGCCTGTTCAAGTGCAACTTACGCCGCCAAAATCAATG
AAGACCTCGCTGCAATAGAAGAACATATCTCAGGTCGCATCGGTGTTTCGGTTTGGGATACACAAACCGA
TGAGCATTGGGATTATCGTGGCGATGAGCGCTTTCCCATGATGAGCACCTTCAAAACACTCGCATGTGCG
ACCATGCTCAATGACATGGATAATGAAAAGCTCGATAAGAACGCGACCGCTAAAGTAGAAGAACGCAACA
TGGTGGTGTGGTCTCCGGTGATGGACCGCATGGCAGGTCAAACGACTCGCATTGAGCATGCTTGTGAAGC
CGCGATGCTGATGAGTGACAACACCGCAGCCAACATTGTTTTGCGCAGTGTGGGTGGACCTCGTGGTGTG
ACAATGTTCTTGCGTACCATTGGAGACAAAGCAACCCGACTTGACCGTTTTGAGCCGAGATTGAATGAAG
CGAAACCGGGCGACAAGCAAGACACGACAACACCTAACGCCATGGTAAACACCCTGCACACCCTACTAGA
AGGCGACGCGCTATCGTATGAATCTCGCATTCAATTAAAGATCTGGATGCAAGACAACAAGGTATCTGAC
TCTCTAATGCGTTCTGTGTTGCCTAAAGGCTGGTCAATTGCAGATCGTTCTGGCGCAGGCGGTTTTGGCT
CACGTGGTATCACCGCGATGATCTGGAAAGAAAATCACAAGCCTGTTTACATCAGTATCTATATCACCGG
CACCGATCTGTCACTGCAAGCTCGTGACCAAGTGATTGCTCAGATTAGCCAACTGGTGTTGGACGAATAC
AAAAATATTTAA

7 ToxR genes from Vibrio spp.
>Vibrio alginolyticus NBRC 15630 ToxR, CP006718.1
ATGACTAACATTGGCACCAAATTTCTACTTGCTCAAAGGTTCACCTTTGATCCAAATAGT
AATTCGCTCGCTGACCAACAAAACGGCAACGACGTTGTACGATTAGGAAGCAACGAAAGC

CGTATACTTCTGATGTTGGCAGAGAGACCAAACGAAGTTTTAACCCGTAACGAGCTTCAC
GAGTTTGTTTGGCGTGAGCAAGGTTTTGAGGTGGATGACTCAAGCCTGACTCAAGCGATT
TCTACTCTGCGTAAGATGTTGAAGGATTCAACAAAATCTCCAGAGTTTGTTAAAACGGTA
CCTAAACGTGGCTACCAACTCATTTGTTCAGTGGAACGCTTGAGCCCATTTTCTACTGAC
TCTAACACTGACGTTGAAGAAACCGCTTCTGAACACGAAGCGCCAGCAGTGGAGTTAGAA
GCGAGCGATACACCACCAACAGAGATAGTGACCGATACTACTGCTGATCTTGAGCCTCAA
GTAGAGCCGACTAAAACTCAGCCGAAGCCAGCATCTAACACGATTAACTGGCTACCGCGT
GTCATTATCTTTTTGTCTCTGCTGCTTCCTGTTTGCGTATTGTTATTCACGAACCCTGCG
GAATCCCAATTCCGTCAGATTGGTGAGTATCAAAATGTACCAGTGATGACACCTGTAAAT
CACCCTCAAATCAACAACTGGTTGCCTTCAATAGAGCAATGCATTGAGCGCTACGTGAAG
CACCATGCAGAAGACTCGTTGCCAGTTGAAGTGATTGCCACTGGTGGACAAAATAACCAG
CTGATTCTGAACTACATTCATGACAGTAACCACTCTTACGAGAACGTAACACTGCGTATT
TTCGCAGGTCAAAACGATCCAACAGACATCTGCAAATAA

> Vibrio campbellii strain NBRC 15631 ToxR, HQ318823.1
ATGACTAACATCGGCACCAAATTTCTGCTTGCTCAAAGATTCGTCTTTGACCCAAATAGTAATTCGCTCG
CTGACCAACAAAATGGCAACGAAGTTGTACGATTAGGCAGCAACGAAAGCCGTATTCTCCTCATGTTGGC
AGAGAGACCTAACGAAGTGATAACACGTAACGAACTGCATGAGTTCGTTTGGCGTGAACAAGGCTTTGAG
GTGGATGACTCAAGCTTAACTCAAGCGATCTCTACACTGCGTAAAATGTTGAAGGATTCAACAAAATCAC
CAGAATTCGTTAAAACCGTGCCAAAACGCGGCTACCAACTTATTTGCTCTGTTGAGCGTTTAAGCCCGCT
TTCTTCTGACTCTATCCCTGATATTGATGATCAAGAAGAAGAGAACGAAGCGCCAGTGGTTGATTTAGAG
CAATTTGCCGAAGCTACCACAGAAACGCAAGCAGACATCGCTCCAGAAGCCGCCGCTCCAGTAGAAAAGC
CGAAGCCAACACCAGCGCAGAAGAACGCGAACTGGCTATTCCGTATTTTGGTTGTCGTTGCTTTGCTGCT
GCCAGTTTGTGTACTGATGTTGACTAAGCCAGCAGAATCTCAATTCCGTCAAATTGGTGAATACCATAAT
GTGCCAGTAATGACGCCAATTAACCACCCTCAATTAAATAATTGGTTGCCTTCCATTGAGCAATGTATCC
AACGTTATGTCGAACACCACGCCGCGGAATCTTCACCAGTCGAAGTCATCGCGACAGGTGGTCAAAACAA
CCAACTGATTCTGAACTATATCCACGATAGCGATCATTCATACGAGAATGTCACGCTGCGTATTTTCGCT
GGTCGTAATGACCCAACCGACATCTGCAACTAA

> Vibrio harveyi strain NBRC 15634 ToxR, DQ403146.1
ATGACTAATATCGGCACCAAATTTCTGCTTGCTCAAAGATTTGTCTTTGACCCAAATAGTAATTCGCTCG
CTGACCAACAAAATGGCAACGAAGTTGTACGATTAGGAAGCAACGAAAGCCGTATTCTACTCATGTTGGC
AGAGAGACCCAATGAAGTGTTAACGCGTAACGAGCTTCACGAGTTCGTATGGCGGGAGCAGGGTTTTGAG
GTGGATGACTCAAGCCTTACTCAAACGATTTCCACTCTACGTAAAATGTTGAAAGACTCTACGAAGTCGC
CTGAATTTGTAAAAACGGTGCCAAAGCGCGGTTACCAACTGATTTGTTCAGTTGAACGTTTAAGCCCACT
ATCTTCTGAAGCAGCACTCACCGATGTTGATGCTCAAGAGGAAGAAAATGAAGCTCCAGTCGTTGATCTA
GAGCAATTCGCAGAGCCCACTGCTGAGACAAAAGCAGAAACAGCCGTCGAACAAGCACCGACAGCTCAAC
CATTAAAATCTGCACCTGCACAAAAGAACACAAACTGGTTATTCAGAATCATCGTGTTAGTTGCCCTGCT
ACTTCCTGTTGGCGTGTTAATGCTAACCAACCCTGCAGAATCACAATTTCGTCAAATTGGTGAATATCAC
AACGTGCCAGTGATGACACCGATCAACCACCCTCAATTAAACAATTGGCTGCCGTCCATTGAGCAATGCA
TCCGAACGGTATGTGAACACCGCGCTGCTGAGTCTTCCCAAGTCGAAGTCATTCACAGTGGTGGGCAAAA
CAATCAGTTGATTCTGAACTACATTCCCGACAGTGATCACTCATATGAGAATGTAACACTGGGGATTTTC
CTGGGGCGGAACGCTCCCACTGACATTTGCATTAGGGGCTGA

>Vibrio parahaemolyticus strain ATCC 17802 ToxR, AY527396.1
ATGACTAACATCGGCACCAAATTTCTACTTGCTCAAAGGTTTACCTTTGATCCAAATAGTAATTCGCTCG
CTGACCAACAAAGCGGCAACGAAGTTGTACGATTAGGAAGCAACGAAAGCCGTATACTCCTGATGTTGGC
GGAGAGACCAAACGAAGTTTTAACCCGTAACGAGCTTCACGAGTTTGTTTGGCGTGAGCAAGGTTTTGAG
GTGGATGACTCAAGCCTGACTCAAGCGATTTCTACTCTGCGTAAGATGTTGAAGGATTCAACCAAATCTC
CAGAGTTTGTTAAAACCGTTCCAAAACGAGGCTATCAACTCATTTGTACTGTTGAACGCCTAAGCCCGCT

TTCTTCAGACTCAAGCTCAATTGAAGTTGAAGAACCTGCTTCTGATAACAATGACGCCTCTGCTCATGAG
GTAGAAACGATCGTAGAGCCGCCTTTAGCGACGCCTTCTGACGCAATCGTTGAACCAGAAGCGCCAGTAG
TACCTGAAAAAGCACCTGTGGCTTCTGCTGTGAATCCTTGGATTCCACGCGTTATTTTATTTTTGGCACT
ATTACTACCGATTTGCGTACTGCTGTTTACAAACCCTGCGGAATCTCAGTTCCGTCAGATTGGTGAGTAT
CAGAACGTACCAGTGATGACACCTGTAAATCACCCGCAAATCAACAACTGGTTGCCTTCTATTGAGCAGT
GCATTGAACGCTACGTTAAGCACCATGCAGAAGACTCGTTACCAGTGGAAGTAATTGCCACTGGCGGACA
AAATAACCAGCTGATTTTGAACTACATTCATGACAGCAACCACTCGTATGAGAACGTGACATTGCGTATT
TTCGCAGGTCAAAATGATCCAACAGACATCTGCAAATAA

>Vibrio cholerae strain BB31 ToxR, KF498634.1
ATGAGTCATATTGGTACTAAATTCATTCTTGCTGAAAAATTTACCTTTGATCCCCTAAGCAATACTCTGA
TTGACAAAGAAGATAGTGAAGAGATCATTCGATTAGGCAGCAACGAAAGTCGAATTCTTTGGCTGCTGGC
CCAACGTCCAAACGAGGTGATTTCTCGCAATGATTTGCATGACTTTGTTTGGCGAGAGCAAGGTTTTGAA
GTCGATGATTCCAGCTTAACCCAAGCCATTTCGACTCTGCGCAAAATGCTCAAAGATTCGACAAAGTCCC
CACAATACGTCAAAACGGTTCCGAAACGCGGTTACCAATTGATCGCCCGAGTCGAAACGGTTGAAGAAGA
GATGGCTCGCGAAAGCGAAGCTGCTCATGACATCTCTCAACCGGAATCTGTCAATGAATACGCAGAATCA
AGCAGTGTGCCTTCATCAGCCACTGTAGTGAACACACCGCAGCCAGCCAATGTCGTGGCGAATAAATCGG
CTCCAAACTTGGGGAATCGACTGTTTATTCTGATAGCGGTCTTACTCCCCCTCGCAGTATTGCTGCTCAC
TAACCCTAGCCAATCCAGCTTTAAACCCCTAACGGTTGTCGATGGCGTAGCCGTCAATATGCCTAATAAC
CACCCTGATCTTTCAAATTGGCTACCGTCAATCGAACTGTGCGTTAAAAAATACAATGAAAAGCATACTG
GTGGGCTCAAGCCGATAGAAGTCATTGCCACGGGTGGACAAAATAACCAGTTAACGTTGAATTACATTCA
CAGCCCTGAAGTTTCGGGGGAAAACATAACCTTACGCATCGTTGCTAACCCCAAAGATGCCATCAATGGG
TGTGAGTAA

>Vibrio vulnificus CMCP6 ToxR, AE016795.3
ATGAGTAATATCGGCACTAAGTTTGTAATCGCACAGCGATTTGTTTTCGATCCTAACAGCAACACATTAC
TTGATCAGGCTGTCAACAATGAAGTGACTCGATTAGGTAGCAATGAAAGTCGAATTTTACTTCTGTTATC
AGAAAAACCTAACGAGGTATTAACTCGTAACGAGTTGCATGAGTTTGTTTGGCGCGAACAAGGTTTCGAA
GTCGATGATTCCAGCCTAACTCAAGCGATTTCTACCTTACGCAAAATGCTCAAAGACTCTACAAAGTCCC
CTGAGTTTGTGAAAACGGTTCCAAAACGTGGTTATCAGTTGATCTGTTCGGTTGAGCGCATTAACCCGCT
CCTGTCAGATTCAACCAACAACGTGAATGACGCAGCTTCTGAAGCATTAGATCAAGAAGAATTAGAAAAC
GAAATCAGTACTGACGCGGTTCAAACATCCTCGTCAGAAATTGGTAGAGATGTCGCGCATAATGCTGGCA
CGTCAACAAAGATGGCCGCGTCACAAAAAAATTGGCTAATTAAAGGGCTATTCTTGTTAGCCGCACTGCT
GCCACTTTGCGTTGTCTTGCTCACCAATCCGTCGGAGTCTAAGTTCCGTTTACTGGAAAATGTCAATGGT
GTGGAAGTGCTCACTCCACTGAATCATCCACCATTACAAGCTTGGATGCCTTCTATTAGGCAGTGTGTTA
ATAAGTATGCAGAAACGCATACTGGCGATTCTGCTCCAGTAAAAGTGATCGCGACAGGTGGCCAGGGTAA
TCAGTTGATCCTCAATTACATCCACACACTTCCACATTCAAACGAGAACGTGACGCTGAGAATTTTCTCA
GAACAAAACGATCTTGGCTCTATCTGTAAATAA

All the 355 tlh genes from 355 whole genome sequences of Vibrio parahaemolyticus out of 366 items (the other tlh genes in the remaining 11 WGS have been truncated and are not included in the following list):

>O3:K6 substr. RIMD 2210633
ATGATGAAAAAAACAATCACACTATTAACTGCATTACTCCCGCTTGCTTCTGCAGTTGCCGAAGAGCCAACCTTAT
CACCAGAAATGGTTTCAGCGTCTGAAGTGATCAGCACGCAAGAAAACCAAACCTATACCTATGTTCGCTGTTGGTA
TCGCACCAGCTACTCGAAAGATGATCCAGCGACCGATTGGGAATGGGCAAAAAACGAAGATGGTAGCTACTTCAC
CATTGACGGCTACTGGTGGAGCTCCGTTTCATTTAAAAACATGTTCTACACCAACACGTCGCAAAACGTTATCCGT
CAGCGTTGTGAAGCAACATTAGATTTGGCGAACGAGAACGCAGACATTACGTTCTTCGCCGCTGACAATCGCTTCT
CATACAACCACACGATCTGGAGCAACGACGCAGCAATGCAGCCAGATCAAATCAACAAAGTGGTTGCACTCGGTG

ACAGCTTGTCTGATACAGGCAACATCTTTAACGCATCACAATGGCGCTTCCCTAACCCGAACAGCTGGTTCTTAGG
TCACTTCTCCAACGGTTTTGTTTGGACAGAATACATTGCCAAAGCGAAGAACCTTCCGCTCTACAACTGGGCAGTT
GGCGGCGCGGCTGGTGAGAACCAATACATCGCGCTAACAGGGGTTGGTGAGCAAGTTTCTTCGTACTTAACCTACG
CAAAACTGGCGAAGAACTACAAACCAGCAAACACCTTGTTTACGCTTGAGTTTGGTTTGAATGACTTCATGAACTA
CAACCGTGGCGTTCCAGAAGTGAAAGCGGATTATGCAGAAGCACTGATTCGTTTGACGGACGCAGGTGCGAAGAA
CTTCATGTTGATGACACTGCCAGATGCGACGAAAGCGCCTCAGTTTAAGTACTCAACACAAGAAGAGATCGACAA
AATTCGTGCGAAAGTGCTTGAGATGAACGAGTTCATCAAGGCACAAGCGATGTACTACAAAGCGCAAGGTTACAA
CATCACGTTGTTTGATACTCACGCCTTGTTCGAGACGCTAACTTCTGCGCCAGAAGAGCACGGTTTCGTGAACGCG
AGCGATCCTTGTTTGGACATCAACCGCTCATCGTCTGTCGATTACATGTACACCCACGCATTGCGCTCTGAGTGTGC
AGCGTCTGGTGCTGAGAAGTTTGTGTTCTGGGATGTCACGCATCCAACAACAGCAACTCACCGCTATGTTGCAGAG
AAAATGCTAGAAAGTAGCAACAACTTAGCCGAGTACCGTTTCTAA

>BB22OP
ATGATGAAAAAAACAATCACACTATTAACTGCATTACTCCCGCTTGCTTCTGCAGTTGCCGAA
GAGCCAACCTTATCACCAGAAATGGTTTCAGCGTCTGAAGTGATCAGCACGCAAGAAAAC
CAAACCTATACCTATGTTCGCTGTTGGTATCGCACCAGCTACTCGAAAGATGATCCGGCG
ACCGATTGGGAATGGGCAAAAAACGAAGATGGTAGCTACTTCACCATTGACGGCTACTGG
TGGAGCTCCGTTTCATTTAAAAACATGTTCTACACCAACACGTCGCAAAACGTTATCCGT
CAGCGTTGTGAAGCCACATTAGATTTGGCGAACGAGAACGCAGACATTACGTTCTTCGCC
GCTGACAATCGCTTCTCATACAACCACACGATCTGGAGCAACGACGCAGCAATGCAGCCA
GATCAAATCAACAAAGTGGTTGCACTCGGTGACAGCTTGTCTGATACAGGCAACATCTTT
AACGCATCACAATGGCGCTTCCCTAACCCGAACAGCTGGTTCTTAGGTCACTTCTCCAAC
GGTTTTGTGTGGACAGAATACATTGCCAAAGCGAAGAACCTTCCGCTCTACAACTGGGCA
GTTGGCGGCGCGGCTGGTGAGAACCAATACATCGCGCTAACAGGGGTTGGTGAGCAAGTT
TCTTCGTACTTAACCTACGCAAAACTGGCGAAGAACTACAAACCAGCAAACACCTTGTTT
ACGCTTGAGTTTGGTTTGAATGACTTCATGAACTACAACCGTGGCGTTCCAGAAGTGAAA
GCAGATTATGCAGAAGCACTGATTCGTTTGACGGACGCAGGTGCGAAGAACTTCATGTTG
ATGACACTGCCAGACGCGACGAAAGCGCCTCAGTTTAAGTACTCAACACAAGAAGAGATC
GACAAAATTCGTGCGAAAGTGCTTGAGATGAACGAGTTCATCAAGGCTCAAGCGATGTAC
TACAAAGCGCAAGGTTACAACATCACGTTGTTTGATACTCACGCCTTGTTCGAGACGCTA
ACTTCTGCGCCAGAAGAGCACGGTTTCGTGAACGCGAGCGATCCTTGTTTGGACATCAAC
CGCTCATCGTCTGTCGACTACATGTACACCCACGCATTGCGCTCTGAGTGTGCGGCGTCT
GGTGCTGAGAAGTTTGTGTTCTGGGATGTCACGCACCCAACAACAGCAACTCACCGCTAT
GTTGCAGAGAAAATGCTAGAAAGTAGCAACAACTTAGCCGAGTACCGTTTCTAA

>FDA_R31
ATGATGAAAAAAACAATCACACTATTAACTGCATTACTCCCGCTTGCTTCTGCAGTTGCCGAAGAGCCAACCTTAT
CACCAGAAATGGTTTCAGCGTCTGAAGTGATCAGCACGCAAGAAAACCAAACCTATACCTATGTTCGCTGTTGGTA
TCGCACCAGCTACTCGAAAGATGATCCGGCGACCGATTGGGAATGGGCAAAAAACGAAGATGGTAGCTACTTCAC
CATTGACGGCTACTGGTGGAGCTCCGTTTCATTTAAAAACATGTTCTACACCAACACGTCGCAAAACGTTATCCGT
CAGCGTTGTGAAGCAACATTAGATTTGGCGAACGAGAACGCAGACATTACGTTCTTCGCCGCTGACAATCGCTTCT
CATACAACCACACGATCTGGAGCAACGACGCAGCAATGCAGCCAGATCAAATCAACAAAGTGGTTGCACTCGGTG
ACAGCTTGTCTGATACAGGCAACATCTTTAACGCATCACAATGGCGCTTCCCTAACCCGAACAGCTGGTTCTTAGG
TCACTTCTCCAACGGTTTTGTGTGGACAGAATACATTGCCAAAGCGAAGAACCTTCCGCTCTACAACTGGGCAGTT
GGCGGTGCGGCTGGTGAGAACCAATACATCGCGCTAACAGGGGTTGGTGATCAAGTTTCTTCGTACTTAACCTACG
CAAAACTGGCGAAGAACTACAAACCAGCAAACACCTTGTTTACGCTTGAGTTTGGTTTGAATGACTTCATGAACTA
CAACCGTGGCGTTCCAGAAGTGAAAGCGGATTATGCAGAAGCACTGATTCGTTTGACGGACGCAGGTGCGAAGAA
CTTCATGTTGATGACACTGCCAGATGCGACGAAAGCGCCTCAGTTTAAGTACTCAACACAAGAAGAGATCGACAA
AATTCGTGCGAAAGTGCTTGAGATGAACGAGTTCATCAAGGCACAAGCGATGTACTACAAAGCGCAAGGTTACAA
CATCACGTTGTTTGATACTCACGCCTTGTTCGAGACGCTAACTTCTGCGCCAGAAGAGCACGGTTTCGTGAACGCG
AGCGATCCTTGTTTGGACATCAACCGCTCATCGTCTGTCGATTACATGTACACCCACGCATTGCGCTCTGAGTGTGC

AGCGTCTGGTGCTGAGAAGTTTGTGTTCTGGGATGTCACGCACCCAACAACAGCAACTCACCGCTATGTTGCAGAG
AAAATGCTAGAAAGTAGCAACAACTTAGAAGAGTTTCGCTTTTAA

>CDC_K4557
ATGATGAAAAAAACAATCACACTATTAACTGCATTACTCCCGCTTGCTTCTGCAGTTGCCGAAGAGCCAACCTTAT
CACCAGAAATGGTTTCAGCGTCTGAAGTGATCAGCACGCAAGAAAACCAAACCTATACCTATGTTCGCTGTTGGTA
TCGCACCAGCTACTCGAAAGATGATCCGGCGACCGATTGGGAATGGGCAAAAAACGAAGATGGTAGCTACTTCAC
CATTGACGGCTACTGGTGGAGCTCCGTTTCATTTAAAAACATGTTCTACACCAACACGTCGCAAAACGTTATCCGT
CAGCGTTGTGAAGCAACATTAGATTTGGCGAACGAGAACGCAGACATTACGTTCTTCGCCGCTGACAATCGCTTCT
CATACAACCACACGATCTGGAGCAACGACGCAGCAATGCAGCCAGATCAAATCAACAAAGTGGTTGCACTCGGTG
ACAGCTTGTCTGATACAGGCAACATCTTTAACGCATCACAATGGCGCTTCCCTAACCCGAACAGCTGGTTCTTAGG
TCACTTCTCCAACGGTTTTGTGTGGACAGAATACATTGCCAAAGCGAAGAACCTTCCGCTCTACAACTGGGCAGTT
GGCGGTGCGGCTGGTGAGAACCAATACATCGCGCTAACAGGGGTTGGTGATCAAGTTTCTTCGTACTTAACCTACG
CAAAACTGGCGAAGAACTACAAACCAGCAAACACCTTGTTTACGCTTGAGTTTGGTTTGAATGACTTCATGAACTA
CAACCGTGGCGTTCCAGAAGTGAAAGCGGATTATGCAGAAGCACTGATTCGTTTGACGGACGCAGGTGCGAAGAA
CTTCATGTTGATGACACTGCCAGATGCGACGAAAGCGCCTCAGTTTAAGTACTCAACACAAGAAGAGATCGACAA
AATTCGTGCGAAAGTGCTTGAGATGAACGAGTTCATCAAGGCACAAGCGATGTACTACAAAGCGCAAGGTTACAA
CATCACGTTGTTTGATACTCACGCCTTGTTCGAGACGCTAACTTCTGCGCCAGAAGAGCACGGTTTCGTGAACGCG
AGCGATCCTTGTTTGGACATCAACCGCTCATCGTCTGTCGATTACATGTACACCCACGCATTGCGCTCTGAGTGTGC
AGCGTCTGGTGCTGAGAAGTTTGTGTTCTGGGATGTCACGCACCCAACAACAGCAACTCACCGCTATGTTGCAGAG
AAAATGCTAGAAAGTAGCAACAACTTAGAAGAGTTTCGCTTTTAA

>UCM-V493
ATGATGAAAAAAACAATCACACTATTAACTGCATTACTCCCGCTTGCTTCTGCAGTTGCCGAAGAGCCAACCTTAT
CACCAGAAATGGTTTCAGCGTCTGAAGTGATCAGCACGCAAGAAAACCAAACCTATACCTATGTTCGCTGTTGGTA
TCGCACCAGCTACTCGAAAGATGATCCGGCGACCGATTGGGAATGGGCAAAAAACGAAGATGGTAGCTACTTCAC
CATTGACGGCTACTGGTGGAGCTCCGTTTCATTTAAAAACATGTTCTACACCAACACGTCGCAAAACGTTATCCGT
CAGCGTTGTGAAGCAACATTAGATTTGGCGAACGAGAACGCAGACATTACGTTCTTCGCCGCTGACAATCGCTTCT
CATACAACCACACGATCTGGAGCAACGACGCAGCAATGCAGCCAGATCAAATCAACAAAGTGGTTGCACTCGGTG
ACAGCTTGTCTGATACAGGCAACATCTTTAACGCATCACAATGGCGCTTCCCTAACCCGAACAGCTGGTTCTTAGG
TCACTTCTCCAACGGTTTTGTGTGGACAGAATACATTGCCAAAGCGAAGAACCTTCCGCTCTACAACTGGGCAGTT
GGCGGCGCGGCTGGTGAGAACCAATACATCGCGCTAACAGGGGTTGGTGATCAAGTTTCTTCGTACTTAACCTACG
CAAAACTGGCGAAGAACTACAAACCAGCAAACACCTTGTTTACGCTTGAGTTTGGTTTGAATGACTTCATGAACTA
CAACCGTGGCGTTCCAGAAGTGAAAGCAGATTATGCAGAAGCACTGATTCGTTTGACGGACGCAGGTGCGAAGAA
CTTCATGTTGATGACACTGCCAGACGCGACGAAAGCGCCTCAGTTTAAGTACTCAACACAAGAAGAGATCGACAA
AATTCGTGCGAAAGTGCTTGAGATGAACGAGTTCATCAAGGCACAAGCGATGTACTACAAAGCGCAAGGTTACAA
CATCACGTTGTTTGATACTCACGCCTTGTTCGAGACGCTAACTTCTGCGCCAGAAGAGCACGGTTTCGTGAACGCG
AGTGATCCTTGTTTGGACATCAACCGCTCATCGTCTGTCGATTACATGTACACCCACGCATTGCGCTCTGAGTGTGC
GGCGTCTGGTGCTGAGAAATTTGTGTTCTGGGATGTCACGCACCCAACAACAGCAACTCACCGCTATGTTGCAGAG
AAAATGCTAGAAAGTAGCAACAACTTAGCCGAGTACCGCTTCTAA

>AQ3810
ATGATGAAAAAAACAATCACACTATTAACTGCATTACTCCCGCTTGCTTCTGCAGTTGCCGAAGAGC
CAACCTTATCACCAGAAATGGTTTCAGCGTCTGAAGTGATCAGCACGCAAGAAAACCAAACCTATACCTA
TGTTCGCTGTTGGTATCGCACCAGCTACTCGAAAGATGATCCAGCGACCGATTGGGAATGGGCAAAAAAC
GAAGATGGTAGCTACTTCACCATTGACGGCTACTGGTGGAGCTCCGTTTCATTTAAAAACATGTTCTACA
CCAACACGTCGCAAAACGTTATCCGTCAGCGTTGTGAAGCAACATTAGATTTGGCGAACGAGAACGCAGA
CATTACGTTCTTCGCCGCTGACAATCGCTTCTCATACAACCACACGATCTGGAGCAACGACGCAGCAATG
CAGCCAGATCAAATCAACAAAGTGGTTGCACTCGGTGACAGCTTGTCTGATACAGGCAACATCTTTAACG
CATCACAATGGCGCTTCCCTAACCCGAACAGCTGGTTCTTAGGTCACTTCTCCAACGGTTTTGTTTGGAC
AGAATACATTGCCAAAGCGAAGAACCTTCCGCTCTACAACTGGGCAGTTGGCGGCGCGGCTGGTGAGAAC

CAATACATCGCGCTAACAGGGGTTGGTGAGCAAGTTTCTTCGTACTTAACCTACGCAAAACTGGCGAAGA
ACTACAAACCAGCAAACACCTTGTTTACGCTTGAGTTTGGTTTGAATGACTTCATGAACTACAACCGTGG
CGTTCCAGAAGTGAAAGCGGATTATGCAGAAGCACTGATTCGTTTGACGGACGCAGGTGCGAAGAACTTC
ATGTTGATGACACTGCCAGATGCGACGAAAGCGCCTCAGTTTAAGTACTCAACACAAGAAGAGATCGACA
AAATTCGTGCGAAAGTGCTTGAGATGAACGAGTTCATCAAGGCACAAGCGATGTACTACAAAGCGCAAGG
TTACAACATCACGTTGTTTGATACTCACGCCTTGTTCGAGACGCTAACTTCTGCGCCAGAAGAGCACGGT
TTCGTGAACGCGAGCGATCCTTGTTTGGACATCAACCGCTCATCGTCTGTCGATTACATGTACACCCACG
CATTGCGCTCTGAGTGTGCAGCGTCTGGTGCTGAGAAGTTTGTGTTCTGGGATGTCACGCATCCAACAAC
AGCAACTCACCGCTATGTTGCAGAGAAAATGCTAGAAAGTAGCAACAACTTAGCCGAGTACCGTTTCTAA

>v110
ATGATGAAAAAAACAATCACACTATTAACTGCATTACTCCCGCTTGCTTCTGCAGTTGCCGAAGAGCCAA
CCTTATCACCAGAAATGGTTTCAGCGTCTGAAGTGATCAGCACGCAAGAAAACCAAACCTATACCTATGT
TCGCTGTTGGTATCGCACCAGCTACTCGAAAGATGACCCGGCGACCGATTGGGAATGGGCAAAAAACGAA
GATGGTAGCTACTTCACCATTGACGGCTACTGGTGGAGCTCCGTTTCATTTAAAAACATGTTCTACACCA
ACACGTCGCAAAACGTTATCCGTCAGCGTTGTGAAGCAACATTAGATTTGGCGAACGAGAACGCAGACAT
TACGTTCTTCGCCGCTGACAATCGCTTCTCATACAACCACACGATCTGGAGCAACGACGCAGCAATGCAG
CCAGATCAAATCAACAAAGTGGTTGCACTCGGTGACAGCTTGTCTGATACAGGCAACATCTTTAACGCAT
CACAATGGCGCTTCCCTAACCCGAACAGCTGGTTCTTAGGTCACTTCTCCAACGGTTTTGTGTGGACTGA
ATACATTGCCAAAGCGAAGAACCTTCCGCTCTACAACTGGGCAGTTGGCGGCGCGGCTGGTGAGAACCAA
TACATCGCGCTAACAGGGGTTGGTGATCAAGTTTCTTCGTACTTAACCTACGCAAAACTGGCGAAGAACT
ACAAACCAGCAAACACCTTGTTTACGCTTGAGTTTGGTTTGAATGACTTCATGAACTACAACCGTGGCGT
TCCAGAAGTGAAAGCGGATTATGCAGAAGCACTGATTCGTTTGACGGACGCAGGTGCGAAGAACTTCATG
TTGATGACACTGCCAGACGCGACGAAAGCGCCTCAGTTTAAGTACTCAACACAAGAAGAGATCGACAAAA
TTCGTGCGAAAGTGCTTGAGATGAACGAGTTCATCAAGGCACAAGCGATGTACTACAAAGCGCAAGGTTA
CAACATCACGTTGTTTGATACTCACGCCTTGTTCGAGACGCTAACTTCTGCGCCAGAAGAGCACGGTTTC
GTGAACGCGAGTGATCCTTGTTTGGACATCAACCGCTCATCGTCTGTCGATTACATGTACACCCACGCAT
TGCGCTCTGAGTGTGCGGCGTCCGGTGCTGAGAAATTTGTGTTCTGGGATGTCACGCACCCAACAACAGC
AACTCACCGCTATGTTGCAGAGAAAATGCTAGAAAGTAGCAACAACTTAGCCGAGTACCGTTTCTAA

>10296
ATGATGAAAAAAACAATCACACTATTAACTGCATTACTCCCGCTTGCTTCTGCAGTTGCCGAAGAGCCAACCTTAT
CACCAGAAATGGTTTCAGCGTCTGAAGTGATCAGCACGCAAGAAAACCAAACCTATACCTATGTTCGCTGTTGGTA
TCGCACCAGCTACTCGAAAGATGATCCGGCGACCGATTGGGAATGGGCAAAAAACGAAGATGGTAGCTACTTCAC
CATTGACGGCTACTGGTGGAGCTCCGTTTCACTTAAAAACATGTTCTACACCAACACGTCGCAAAACGTTATCCGT
CAGCGTTGTGAAGCAACATTAGATTTGGCGAACGAGAACGCAGACATTACGTTCTTCGCCGCTGACAATCGCTTCT
CATACAACCACACGATCTGGAGCAACGACGCAGCAATGCAGCCAGATCAAATCAACAAAGTGGTTGCACTCGGTG
ACAGCTTGTCTGATACAGGCAACATCTTTAACGCATCACAATGGCGCTTCCCTAACCCGAACAGCTGGTTCTTAGG
TCACTTCTCCAACGGTTTTGTGTGGACAGAATACATTGCCAAAGCGAAGAACCTTCCGCTCTACAACTGGGCAGTT
GGCGGCGCGGCTGGTGAGAACCAATACATCGCGCTAACAGGGGTTGGTGATCAAGTTTCTTCGTACTTAACCTACG
CAAAACTGGCGAAGAACTACAAACCAGCAAACACCTTGTTTACGCTTGAGTTTGGTTTGAATGACTTCATGAACTA
CAACCGTGGCGTTCCAGAAGTGAAAGCAGATTATGCAGAAGCACTGATTCGTTTGACGGACGCAGGTGCGAAGAA
CTTCATGTTGATGACACTGCCAGACGCGACGAAAGCGCCTCAGTTTAAGTACTCAACACAAGAAGAGATCGACAA
AATTCGTGCGAAAGTGCTTGAGATGAACGAGTTCATCAAGGCACAAGCGATGTACTACAAAGCGCAAGGTTACAA
CATCACGTTGTTTGATACTCACGCCTTGTTCGAGACGCTAACTTCTGCGCCAGAAGAGCACGGTTTCGTGAACGCG
AGCGATCCTTGTTTGGACATCAACCGCTCATCGTCTGTCGATTACATGTACACCCACGCATTGCGCTCTGAGTGTGC
AGCGTCTGGTGCTGAGAAGTTTGTGTTCTGGGATGTCACGCATCCAACAACAGCAACTCACCGCTATGTTGCAGAG
AAAATGCTAGAAAGTAGCAACAACTTAGCCGAGTACCGTTTCTAA

>12310
ATGATGAAAAAAACAATCACACTATTAACTGCATTACTCCCGCTTGCTTCTGCAGTTGCCGAAGAGCCAACCTTAT
CACCAGAAATGGTTTCAGCGTCTGAAGTGATCAGCACGCAAGAAAACCAAACCTATACCTATGTTCGCTGTTGGTA
TCGCACCAGCTACTCGAAAGATGATCCGGCGACCGATTGGGAATGGGCAAAAAACGAAGATGGTAGCTACTTCAC
CATTGACGGCTACTGGTGGAGCTCCGTTTCACTTAAAAACATGTTCTACACCAACACGTCGCAAAACGTTATCCGT
CAGCGTTGTGAAGCAACATTAGATTTGGCGAACGAGAACGCAGACATTACGTTCTTCGCCGCTGACAATCGCTTCT
CATACAACCACACGATCTGGAGCAACGACGCAGCAATGCAGCCAGATCAAATCAACAAAGTGGTTGCACTCGGTG
ACAGCTTGTCTGATACAGGCAACATCTTTAACGCATCACAATGGCGCTTCCCTAACCCGAACAGCTGGTTCTTAGG
TCACTTCTCCAACGGTTTTGTGTGGACAGAATACATTGCCAAAGCGAAGAACCTTCCGCTCTACAACTGGGCAGTT
GGCGGCGCGGCTGGTGAGAACCAATACATCGCGCTAACAGGGGTTGGTGATCAAGTTTCTTCGTACTTAACCTACG
CAAAACTGGCGAAGAACTACAAACCAGCAAACACCTTGTTTACGCTTGAGTTTGGTTTGAATGACTTCATGAACTA
CAACCGTGGCGTTCCAGAAGTGAAAGCAGATTATGCAGAAGCACTGATTCGTTTGACGGACGCAGGTGCGAAGAA
CTTCATGTTGATGACACTGCCAGACGCGACGAAAGCGCCTCAGTTTAAGTACTCAACACAAGAAGAGATCGACAA
AATTCGTGCGAAAGTGCTTGAGATGAACGAGTTCATCAAGGCACAAGCGATGTACTACAAAGCGCAAGGTTACAA
CATCACGTTGTTTGATACTCACGCCTTGTTCGAGACGCTAACTTCTGCGCCAGAAGAGCACGGTTTCGTGAACGCG
AGCGATCCTTGTTTGGACATCAACCGCTCATCGTCTGTCGATTACATGTACACCCACGCATTGCGCTCTGAGTGTGC
AGCGTCTGGTGCTGAGAAGTTTGTGTTCTGGGATGTCACGCATCCAACAACAGCAACTCACCGCTATGTTGCAGAG
AAAATGCTAGAAAGTAGCAACAACTTAGCCGAGTACCGTTTCTAA

>EKP-008
ATGATGAAAAAAACAATCACACTATTAACTGCATTACTCCCGCTTGCTTCTGCAGTTGCCGAAGAGCCAA
CCTTATCACCAGAAATGGTTTCAGCGTCTGAAGTGATCAGCACGCAAGAAAACCAAACCTATACCTATGT
TCGCTGTTGGTATCGCACCAGCTACTCGAAAGATGATCCGGCGACCGATTGGGAATGGGCAAAAAACGAA
GATGGTAGCTACTTCACCATTGACGGCTACTGGTGGAGCTCCGTTTCATTTAAAAACATGTTCTACACCA
ACACGTCGCAAAACGTTATCCGTCAGCGTTGTGAAGCAACATTAGATTTGGCGAACGAGAACGCAGACAT
TACGTTCTTCGCCGCTGACAATCGCTTCTCATACAACCACACGATCTGGAGCAACGACGCAGCAATGCAG
CCAGATCAAATCAACAAAGTGGTTGCACTCGGTGACAGCTTGTCTGATACAGGCAACATCTTTAACGCAT
CACAATGGCGCTTCCCTAACCCGAACAGCTGGTTCTTAGGTCACTTCTCCAACGGTTTTGTGTGGACAGA
ATACATTGCCAAAGCGAAGAACCTTCCGCTCTACAACTGGGCAGTTGGCGGCGCGGCTGGTGAGAACCAA
TACATCGCGCTAACAGGGGTTGGTGATCAAGTTTCTTCGTACTTAACCTACGCAAAACTGGCGAAGAACT
ACAAACCAGCAAACACCTTGTTTACGCTTGAGTTTGGTTTGAATGACTTCATGAACTACAACCGTGGCGT
TCCAGAAGTGAAAGCAGATTATGCAGAAGCACTGATTCGTTTGACGGACGCAGGTGCGAAGAACTTCATG
TTGATGACACTGCCAGACGCGACGAAAGCGCCTCAGTTTAAGTACTCAACACAAGAAGAGATCGACAAAA
TTCGTGCGAAAGTGCTTGAGATGAACGAGTTCATCAAGGCACAAGCGATGTACTACAAAGCGCAAGGTTA
CAACATCACGTTGTTTGATACTCACGCCTTGTTCGAGACGCTAACTTCTGCGCCAGAAGAGCACGGTTTC
GTGAACGCGAGTGATCCTTGTTTGGACATCAACCGCTCATCGTCTATCGATTACATGTACACCCACGCAT
TGCGCTCTGAGTGTGCGGCGTCTGGTGCTGAGAAGTTTGTGTTCTGGGATGTCACGCACCCAACAACAGC
AACTCACCGCTATGTTGCAGAGAAAATGCTAGAAAGTAGCAACAACTTAGCCGAGTACCGTTTCTAA

>970107
ATGATGAAAAAAACAATCACACTATTAACTGCATTACTCCCGCTTGCTTCTGCAGTTGCCGAAGAGCCAA
CCTTATCACCAGAAATGGTTTCAGCGTCTGAAGTGATCAGCACGCAAGAAAACCAAACCTATACCTATGT
TCGCTGTTGGTATCGCACCAGCTACTCGAAAGATGATCCGGCGACCGATTGGGAATGGGCAAAAAACGAA
GATGGTAGCTACTTCACCATTGACGGCTACTGGTGGAGCTCCGTTTCATTTAAAAACATGTTCTACACCA
ACACGTCGCAAAACGTTATCCGTCAGCGTTGTGAAGCAACATTAGATTTGGCGAACGAGAACGCAGACAT
TACGTTCTTCGCCGCTGACAATCGCTTCTCATACAACCACACGATCTGGAGCAACGACGCAGCAATGCAG
CCAGATCAAATCAACAAAGTGGTTGCACTCGGTGACAGCTTGTCTGATACAGGCAACATCTTTAACGCAT
CACAATGGCGCTTCCCTAACCCGAACAGCTGGTTCTTAGGTCACTTCTCCAACGGTTTTGTGTGGACAGA
ATACATTGCCAAAGCGAAGAACCTTCCGCTCTACAACTGGGCAGTTGGCGGCGCGGCTGGTGAGAACCAA
TACATCGCGCTAACAGGGGTTGGTGATCAAGTTTCTTCGTACTTAACCTACGCAAAACTGGCGAAGAACT

ACAAACCAGCAAACACCTTGTTTACGCTTGAGTTTGGTTTGAATGACTTCATGAACTACAACCGTGGCGT
TCCAGAAGTGAAAGCGGATTATGCAGAAGCACTGATTCGTTTGACGGACGCAGGTGCGAAGAACTTCATG
TTGATGACACTGCCAGACGCGACGAAAGCGCCTCAGTTTAAGTACTCAACACAAGAAGAGATCGACAAAA
TTCGTGCGAAAGTGCTTGAGATGAACGAGTTCATCAAGGCACAAGCGATGTACTACAAAGCGCAAGGTTA
CAACATCACGTTGTTTGATACTCACGCCTTGTTCGAGACGCTAACTTCTGCGCCAGAAGAGCACGGTTTC
GTGAACGCGAGCGATCCTTGTTTGGACATCAACCGCTCATCGTCTGTCGATTACATGTACACCCACGCAT
TGCGCTCTGAGTGTGCAGCGTCTGGTGCTGAGAAGTTTGTGTTCTGGGATGTCACGCATCCAACAACAGC
AACTCACCGCTATGTTGCAGAGAAAATGCTAGAAAGTAGCAACAACTTAGCCGAGTACCGTTTCTAA

>B-265
ATGATGAAAAAAACAATCACACTATTAACTGCATTACTCCCGCTTGCTTCTGCAGTTGCCGAAGAGCCAACCTTAT
CACCAGAAATGGTTTCAGCGTCTGAAGTGATCAGCACGCAAGAAAACCAAACCTATACCTATGTTCGCTGTTGGTA
TCGCACCAGCTACTCGAAAGATGATCCGGCGACCGATTGGGAATGGGCAAAAAACGAAGATGGTAGCTACTTCAC
CATTGACGGCTACTGGTGGAGCTCCGTTTCATTTAAAAACATGTTCTACACCAACACGTCGCAAAACGTTATCCGT
CAGCGTTGTGAAGCAACATTAGATTTGGCGAACGAGAACGCAGACATTACGTTCTTCGCCGCTGACAATCGCTTCT
CATACAACCACACGATCTGGAGCAACGACGCAGCAATGCAGCCAGATCAAATCAACAAAGTGGTTGCTCTCGGTG
ACAGCTTGTCTGATACAGGCAACATCTTTAACGCATCACAATGGCGCTTCCCTAACCCGAACAGCTGGTTCTTAGG
TCACTTCTCCAACGGTTTTGTGTGGACAGAATACATTGCCAAAGCGAAGAACCTTCCGCTCTACAACTGGGCAGTT
GGCGGTGCGGCTGGTGAGAACCAATACATCGCGCTAACAGGGGTTGGTGAGCAAGTTTCTTCGTACTTAACCTACG
CAAAACTGGCGAAGAACTACAAACCAGCAAACACCTTGTTTACGCTTGAGTTTGGTTTGAATGACTTCATGAACTA
CAACCGTGGAGTTCCAGAAGTGAAAGCGGATTATGCAGAAGCACTGATTCGTTTGACGGACGCAGGTGCGAAGAA
CTTCATGTTGATGACACTGCCAGACGCGACGAAAGCGCCTCAGTTTAAGTACTCAACACAAGAAGAGATCGACAA
AATTCGTGCGAAAGTGCTTGAGATGAACGAGTTCATCAAGGCACAAGCGATGTACTACAAAGCGCAAGGTTACAA
CATCACGTTGTTTGATACTCACGCCTTGTTCGAGACGCTAACTTCTGCGCCAGAAGAGCACGGTTTCGTGAACGCG
AGTGATCCTTGTTTGGACATCAACCGCTCATCGTCTGTCGATTACATGTACACCCACGCATTGCGCTCTGAGTGTGC
GGCGTCCGGTGCTGAGAAATTTGTGTTCTGGGATGTCACGCACCCAACAACAGCAACTCACCGCTATGTTGCAGAG
AAAATGCTAGAAAGTAGCAACAACTTAGCCGAGTACCGTTTCTAA

>605
ATGATGAAAAAAACAATCACACTATTAACTGCATTACTCCCGCTTGCTTCTGCAGTTGCCGAAGAGCCAA
CCTTATCACCAGAAATGGTTTCAGCGTCTGAAGTGATCAGCACGCAAGAAAACCAAACCTATACCTATGT
TCGCTGTTGGTATCGCACCAGCTACTCGAAAGATGATCCGGCGACCGATTGGGAATGGGCAAAAAACGAA
GATGGTAGCTACTTCACCATTGACGGCTACTGGTGGAGCTCCGTTTCATTTAAAAACATGTTCTACACCA
ACACGTCGCAAAACGTTATCCGTCAGCGTTGTGAAGCAACATTAGATTTGGCGAACGAGAACGCAGACAT
TACGTTCTTCGCCGCTGACAATCGCTTCTCATACAACCACACGATCTGGAGCAACGACGCAGCAATGCAG
CCAGATCAAATCAACAAAGTGGTTGCTCTCGGTGACAGCTTGTCTGATACAGGCAACATCTTTAACGCAT
CACAATGGCGCTTCCCTAACCCGAACAGCTGGTTCTTAGGTCACTTCTCCAACGGTTTTGTGTGGACAGA
ATACATTGCCAAAGCGAAGAACCTTCCGCTCTACAACTGGGCAGTTGGCGGCGCGGCTGGTGAGAACCAA
TACATCGCGCTAACAGGGTTGGTGAGCAAGTTTCTTCGTACTTAACCTACGCAAAACTGGCGAAGAACTA
CAAACCAGCAAACACCTTGTTTACGCTTGAGTTTGGTTTGAATGACTTCATGAACTACAACCGTGGCGTT
CCAGAAGTGAAAGCGGATTATGCAGAAGCACTGATTCGTTTGACGGACGCAGGTGCGAAGAACTTCATGT
TGATGACACTGCCAGACGCGACGAAAGCGCCTCAGTTTAAGTACTCAACACAAGAAGAGAGCGACAAAAT
TCGTGCGAAAGTGCTTGAGATGAACGAGTTCATCAAGGCACAAGCGATGTACTACAAAGCGCAAGGTTAC
AACATCACGTTGTTTGATACTCACGCCTTGTTCGAGACGCTAACTTCTGCGCCAGAAGAGCACGGTTTCG
TGAACGCGAGTGATCCTTGTTTGGACATCAACCGCTCATCGTCTGTCGATTACATGTACACCCACGCATT
GCGCTCTGAGTGTGCAGCGTCTGGTGCTGAGAAGTTTGTGTTCTGGGATGTCACGCATCCAACAACAGCA
ACTCACCGCTATGTTGCAGAGAAAATGCTAGAAAGTAGCAACAACTTAGCAGAGTACCGTTTCTAA

>50
ATGATGAAAAAAACAATCACACTATTAACTGCATTACTCCCGCTTGCTTCTGCAGTTGCCGAAGAGCCAACCTTAT
CACCAGAAATGGTTTCAGCGTCTGAAGTGATCAGCACGCAAGAAAACCAAACCTATACCTATGTTCGCTGTTGGTA

TCGCACCAGCTACTCGAAAGATGATCCGGCGACCGATTGGGAATGGGCAAAAAACGAAGATGGTAGCTACTTCAC
CATTGACGGCTACTGGTGGAGCTCCGTTTCATTTAAAAACATGTTCTACACCAACACGTCGCAAAACGTTATCCGT
CAGCGTTGTGAAGCAACATTAGATTTGGCGAACGAGAACGCAGACATTACGTTCTTCGCCGCTGACAATCGCTTCT
CATACAACCACACGATCTGGAGCAACGACGCAGCAATGCAGCCAGATCAAATCAACAAAGTGGTTGCTCTCGGTG
ACAGCTTGTCTGATACAGGCAACATCTTTAACGCATCACAATGGCGCTTCCCTAACCCGAACAGCTGGTTCTTAGG
TCACTTCTCCAACGGTTTTGTGTGGACAGAATACATTGCCAAAGCGAAGAACCTTCCGCTCTACAACTGGGCAGTT
GGCGGCGCGGCTGGTGAGAACCAATACATCGCGCTAACAGGGGTTGGTGATCAAGTTTCTTCGTACTTAACCTACG
CAAAACTGGCGAAGAACTACAAACCAGCAAACACCTTGTTTACGCTTGAGTTTGGTTTGAATGACTTCATGAACTA
CAACCGTGGCGTTCCAGAAGTGAAAGCAGATTATGCAGAAGCACTGATTCGTTTGACGGACGCAGGTGCGAAGAA
CTTCATGTTGATGACACTGCCAGACGCGACAAAGGCGCCTCAGTTTAAGTACTCAACACAAGAAGAGATCGACAA
AATTCGTGCGAAAGTGCTTGAGATGAACGAGTTCATCAAGGCACAAGCGATGTACTACAAAGCGCAAGGTTACAA
CATCACGTTGTTTGATACTCACGCCTTGTTCGAGACGCTAACTTCTGCGCCAGAAGAGCACGGTTTCGTGAACGCG
AGTGATCCTTGTTTGGACATCAACCGCTCATCGTCTGTCGATTACATGTACACCCACGCATTGCGCTCTGAGTGTGC
AGCGTCTGGTGCTGAGAAGTTTGTGTTCTGGGATGTCACGCATCCAACAACAGCAACTCACCGCTATGTTGCAGAG
AAAATGCTAGAAAGTAGCAACAACTTAGCAGAGTACCGTTTCTAA

>3256
ATGATGAAAAAAACAATCACACTATTAACTGCATTACTCCCGCTTGCTTCTGCAGTTGCCGAAGAGCCAA
CCTTATCACCAGAAATGGTTTCAGCGTCTGAAGTGATCAGCACGCAAGAAAACCAAACCTATACCTATGT
TCGCTGTTGGTATCGCACCAGCTACTCGAAAGATGATCCGGCGACCGATTGGGAATGGGCAAAAAACGAA
GATGGTAGCTACTTCACCATTGACGGCTACTGGTGGAGCTCCGTTTCACTTAAAAACATGTTCTACACCA
ACACGTCGCAAAACGTTATCCGTCAGCGTTGTGAAGCAACATTAGATTTGGCGAACGAGAACGCAGACAT
TACGTTCTTCGCCGCTGACAATCGCTTCTCATACAACCACACGATCTGGAGCAACGACGCAGCAATGCAG
CCAGATCAAATCAACAAAGTGGTTGCACTCGGTGACAGCTTGTCTGATACAGGCAACATCTTTAACGCAT
CACAATGGCGCTTCCCTAACCCGAACAGCTGGTTCTTAGGTCACTTCTCCAACGGTTTTGTGTGGACAGA
ATACATTGCCAAAGCGAAGAACCTTCCGCTCTACAACTGGGCAGTTGGCGGCGCGGCTGGTGAGAACCAA
TACATCGCGCTAACAGGGGTTGGTGATCAAGTTTCTTCGTACTTAACCTACGCAAAACTGGCGAAGAACT
ACAAACCAGCAAACACCTTGTTTACGCTTGAGTTTGGTTTGAATGACTTCATGAACTACAACCGTGGCGT
TCCAGAAGTGAAAGCAGATTATGCAGAAGCACTGATTCGTTTGACGGACGCAGGTGCGAAGAACTTCATG
TTGATGACACTGCCAGACGCGACGAAAGCGCCTCAGTTTAAGTACTCAACACAAGAAGAGATCGACAAAA
TTCGTGCGAAAGTGCTTGAGATGAACGAGTTCATCAAGGCACAAGCGATGTACTACAAAGCGCAAGGTTA
CAACATCACGTTGTTTGATACTCACGCCTTGTTCGAGACGCTAACTTCTGCGCCAGAAGAGCACGGTTTC
GTGAACGCGAGCGATCCTTGTTTGGACATCAACCGCTCATCGTCTGTCGATTACATGTACACCCACGCAT
TGCGCTCTGAGTGTGCAGCGTCTGGTGCTGAGAAGTTTGTGTTCTGGGATGTCACGCATCCAACAACAGC
AACTCACCGCTATGTTGCAGAGAAAATGCTAGAAAGTAGCAACAACTTAGCCGAGTACCGTTTCTAA

>IDH02640
ATGATGAAAAAAACAATCACACTATTAACTGCATTACTCCCGCTTGCTTCTGCAGTTGCCGAAGAGCCAACCTTAT
CACCAGAAATGGTTTCAGCGTCTGAAGTGATCAGCACGCAAGAAAACCAAACCTATACCTATGTTCGCTGTTGGTA
TCGCACCAGCTACTCGAAAGATGATCCAGCGACCGATTGGGAATGGGCAAAAAACGAAGATGGTAGCTACTTCAC
CATTGACGGCTACTGGTGGAGCTCCGTTTCATTTAAAAACATGTTCTACACCAACACGTCGCAAAACGTTATCCGT
CAGCGTTGTGAAGCAACATTAGATTTGGCGAACGAGAACGCAGACATTACGTTCTTCGCCGCTGACAATCGCTTCT
CATACAACCACACGATCTGGAGCAACGACGCAGCAATGCAGCCAGATCAAATCAACAAAGTGGTTGCACTCGGTG
ACAGCTTGTCTGATACAGGCAACATCTTTAACGCATCACAATGGCGCTTCCCTAACCCGAACAGCTGGTTCTTAGG
TCACTTCTCCAACGGTTTTGTTTGGACAGAATACATTGCCAAAGCGAAGAACCTTCCGCTCTACAACTGGGCAGTT
GGCGGCGCGGCTGGTGAGAACCAATACATCGCGCTAACAGGGGTTGGTGAGCAAGTTTCTTCGTACTTAACCTACG
CAAAACTGGCGAAGAACTACAAACCAGCAAACACCTTGTTTACGCTTGAGTTTGGTTTGAATGACTTCATGAACTA
CAACCGTGGCGTTCCAGAAGTGAAAGCGGATTATGCAGAAGCACTGATTCGTTTGACGGACGCAGGTGCGAAGAA
CTTCATGTTGATGACACTGCCAGATGCGACGAAAGCGCCTCAGTTTAAGTACTCAACACAAGAAGAGATCGACAA
AATTCGTGCGAAAGTGCTTGAGATGAACGAGTTCATCAAGGCACAAGCGATGTACTACAAAGCGCAAGGTTACAA

CATCACGTTGTTTGATACTCACGCCTTGTTCGAGACGCTAACTTCTGCGCCAGAAGAGCACGGTTTCGTGAACGCG
AGCGATCCTTGTTTGGACATCAACCGCTCATCGTCTGTCGATTACATGTACACCCACGCATTGCGCTCTGAGTGTGC
AGCGTCTGGTGCTGAGAAGTTTGTGTTCTGGGATGTCACGCATCCAACAACAGCAACTCACCGCTATGTTGCAGAG
AAAATGCTAGAAAGTAGCAACAACTTAGCCGAGTACCGTTTCTAA

>IDH02189
ATGATGAAAAAAACAATCACACTATTAACTGCATTACTCCCGCTTGCTTCTGCAGTTGCCGAAGAGCCAA
CCTTATCACCAGAAATGGTTTCAGCGTCTGAAGTGATCAGCACGCAAGAAAACCAAACCTATACCTATGT
TCGCTGTTGGTATCGCACCAGCTACTCGAAAGATGATCCAGCGACCGATTGGGAATGGGCAAAAAACGAA
GATGGTAGCTACTTCACCATTGACGGCTACTGGTGGAGCTCCGTTTCATTTAAAAACATGTTCTACACCA
ACACGTCGCAAAACGTTATCCGTCAGCGTTGTGAAGCAACATTAGATTTGGCGAACGAGAACGCAGACAT
TACGTTCTTCGCCGCTGACAATCGCTTCTCATACAACCACACGATCTGGAGCAACGACGCAGCAATGCAG
CCAGATCAAATCAACAAAGTGGTTGCACTCGGTGACAGCTTGTCTGATACAGGCAACATCTTTAACGCAT
CACAATGGCGCTTCCCTAACCCGAACAGCTGGTTCTTAGGTCACTTCTCCAACGGTTTTGTTTGGACAGA
ATACATTGCCAAAGCGAAGAACCTTCCGCTCTACAACTGGGCAGTTGGCGGCGCGGCTGGTGAGAACCAA
TACATCGCGCTAACAGGGGTTGGTGAGCAAGTTTCTTCGTACTTAACCTACGCAAAACTGGCGAAGAACT
ACAAACCAGCAAACACCTTGTTTACGCTTGAGTTTGGTTTGAATGACTTCATGAACTACAACCGTGGCGT
TCCAGAAGTGAAAGCGGATTATGCAGAAGCACTGATTCGTTTGACGGACGCAGGTGCGAAGAACTTCATG
TTGATGACACTGCCAGATGCGACGAAAGCGCCTCAGTTTAAGTACTCAACACAAGAAGAGATCGACAAAA
TTCGTGCGAAAGTGCTTGAGATGAACGAGTTCATCAAGGCACAAGCGATGTACTACAAAGCGCAAGGTTA
CAACATCACGTTGTTTGATACTCACGCCTTGTTCGAGACGCTAACTTCTGCGCCAGAAGAGCACGGTTTC
GTGAACGCGAGCGATCCTTGTTTGGACATCAACCGCTCATCGTCTGTCGATTACATGTACACCCACGCAT
TGCGCTCTGAGTGTGCAGCGTCTGGTGCTGAGAAGTTTGTGTTCTGGGATGTCACGCATCCAACAACAGC
AACTCACCGCTATGTTGCAGAGAAAATGCTAGAAAGTAGCAACAACTTAGCCGAGTACCGTTTCTAA

>SBR10290
ATGATGAAAAAAACAATCACACTATTAACTGCATTACTCCCGCTTGCTTCTGCAGTTGCCGAAGAGCCAA
CCTTATCACCAGAAATGGTTTCAGCGTCTGAAGTGATCAGCACGCAAGAAAACCAAACCTATACCTATGT
TCGCTGTTGGTATCGCACCAGCTACTCGAAAGATGATCCGGCGACCGATTGGGAATGGGCAAAAAACGAA
GATGGTAGCTACTTCACCATTGACGGCTACTGGTGGAGCTCCGTTTCACTTAAAAACATGTTCTACACCA
ACACGTCGCAAAACGTTATCCGTCAGCGTTGTGAAGCAACATTAGATTTGGCGAACGAGAACGCAGACAT
TACGTTCTTCGCCGCTGACAATCGCTTCTCATACAACCACACGATCTGGAGCAACGACGCAGCAATGCAG
CCAGATCAAATCAACAAAGTGGTTGCACTCGGTGACAGCTTGTCTGATACAGGCAACATCTTTAACGCAT
CACAATGGCGCTTCCCTAACCCGAACAGCTGGTTCTTAGGTCACTTCTCCAACGGTTTTGTGTGGACAGA
ATACATTGCCAAAGCGAAGAACCTTCCGCTCTACAACTGGGCAGTTGGCGGCGCGGCTGGTGAGAACCAA
TACATCGCGCTAACAGGGGTTGGTGATCAAGTTTCTTCGTACTTAACCTACGCAAAACTGGCGAAGAACT
ACAAACCAGCAAACACCTTGTTTACGCTTGAGTTTGGTTTGAATGACTTCATGAACTACAACCGTGGCGT
TCCAGAAGTGAAAGCAGATTATGCAGAAGCACTGATTCGTTTGACGGACGCAGGTGCGAAGAACTTCATG
TTGATGACACTGCCAGACGCGACGAAAGCGCCTCAGTTTAAGTACTCAACACAAGAAGAGATCGACAAAA
TTCGTGCGAAAGTGCTTGAGATGAACGAGTTCATCAAGGCACAAGCGATGTACTACAAAGCGCAAGGTTA
CAACATCACGTTGTTTGATACTCACGCCTTGTTCGAGACGCTAACTTCTGCGCCAGAAGAGCACGGTTTC
GTGAACGCGAGCGATCCTTGTTTGGACATCAACCGCTCATCGTCTGTCGATTACATGTACACCCACGCAT
TGCGCTCTGAGTGTGCAGCGTCTGGTGCTGAGAAGTTTGTGTTCTGGGATGTCACGCATCCAACAACAGC
AACTCACCGCTATGTTGCAGAGAAAATGCTAGAAAGTAGCAACAACTTAGCCGAGTACCGTTTCTAA

>Peru-288
ATGATGAAAAAAACAATCACACTATTAACTGCATTACTCCCGCTTGCTTCTGCAGTTGCCGAAGAGCCAA
CCTTATCACCAGAAATGGTTTCAGCGTCTGAAGTGATCAGCACGCAAGAAAACCAAACCTATACCTATGT
TCGCTGTTGGTATCGCACCAGCTACTCGAAAGATGATCCAGCGACCGATTGGGAATGGGCAAAAAACGAA

GATGGTAGCTACTTCACCATTGACGGCTACTGGTGGAGCTCCGTTTCATTTAAAAACATGTTCTACACCA
ACACGTCGCAAAACGTTATCCGTCAGCGTTGTGAAGCAACATTAGATTTGGCGAACGAGAACGCAGACAT
TACGTTCTTCGCCGCTGACAATCGCTTCTCATACAACCACACGATCTGGAGCAACGACGCAGCAATGCAG
CCAGATCAAATCAACAAAGTGGTTGCACTCGGTGACAGCTTGTCTGATACAGGCAACATCTTTAACGCAT
CACAATGGCGCTTCCCTAACCCGAACAGCTGGTTCTTAGGTCACTTCTCCAACGGTTTTGTTTGGACAGA
ATACATTGCCAAAGCGAAGAACCTTCCGCTCTACAACTGGGCAGTTGGCGGCGCGGCTGGTGAGAACCAA
TACATCGCGCTAACAGGGGTTGGTGAGCAAGTTTCTTCGTACTTAACCTACGCAAAACTGGCGAAGAACT
ACAAACCAGCAAACACCTTGTTTACGCTTGAGTTTGGTTTGAATGACTTCATGAACTACAACCGTGGCGT
TCCAGAAGTGAAAGCGGATTATGCAGAAGCACTGATTCGTTTGACGGACGCAGGTGCGAAGAACTTCATG
TTGATGACACTGCCAGATGCGACGAAAGCGCCTCAGTTTAAGTACTCAACACAAGAAGAGATCGACAAAA
TTCGTGCGAAAGTGCTTGAGATGAACGAGTTCATCAAGGCACAAGCGATGTACTACAAAGCGCAAGGTTA
CAACATCACGTTGTTTGATACTCACGCCTTGTTCGAGACGCTAACTTCTGCGCCAGAAGAGCACGGTTTC
GTGAACGCGAGCGATCCTTGTTTGGACATCAACCGCTCATCGTCTGTCGATTACATGTACACCCACGCAT
TGCGCTCTGAGTGTGCAGCGTCTGGTGCTGAGAAGTTTGTGTTCTGGGATGTCACGCATCCAACAACAGC
AACTCACCGCTATGTTGCAGAGAAAATGCTAGAAAGTAGCAACAACTTAGCCGAGTACCGTTTCTAA

>861
ATGATGAAAAAAACAATCACACTATTAACTGCATTACTCCCGCTTGCTTCTGCAGTTGCCGAAGAGCCAACCTTAT
CACCAGAAATGGTTTCAGCGTCTGAAGTGATCAGCACGCAAGAAAACCAAACCTATACCTATGTTCGCTGTTGGTA
TCGCACCAGCTACTCGAAAGATGATCCAGCGACCGATTGGGAATGGGCAAAAAACGAAGATGGTAGCTACTTCAC
CATTGACGGCTACTGGTGGAGCTCCGTTTCATTTAAAAACATGTTCTACACCAACACGTCGCAAAACGTTATCCGT
CAGCGTTGTGAAGCAACATTAGATTTGGCGAACGAGAACGCAGACATTACGTTCTTCGCCGCTGACAATCGCTTCT
CATACAACCACACGATCTGGAGCAACGACGCAGCAATGCAGCCAGATCAAATCAACAAAGTGGTTGCACTCGGTG
ACAGCTTGTCTGATACAGGCAACATCTTTAACGCATCACAATGGCGCTTCCCTAACCCGAACAGCTGGTTCTTAGG
TCACTTCTCCAACGGTTTTGTTTGGACAGAATACATTGCCAAAGCGAAGAACCTTCCGCTCTACAACTGGGCAGTT
GGCGGCGCGGCTGGTGAGAACCAATACATCGCGCTAACAGGGGTTGGTGAGCAAGTTTCTTCGTACTTAACCTACG
CAAAACTGGCGAAGAACTACAAACCAGCAAACACCTTGTTTACGCTTGAGTTTGGTTTGAATGACTTCATGAACTA
CAACCGTGGCGTTCCAGAAGTGAAAGCGGATTATGCAGAAGCACTGATTCGTTTGACGGACGCAGGTGCGAAGAA
CTTCATGTTGATGACACTGCCAGATGCGACGAAAGCGCCTCAGTTTAAGTACTCAACACAAGAAGAGATCGACAA
AATTCGTGCGAAAGTGCTTGAGATGAACGAGTTCATCAAGGCACAAGCGATGTACTACAAAGCGCAAGGTTACAA
CATCACGTTGTTTGATACTCACGCCTTGTTCGAGACGCTAACTTCTGCGCCAGAAGAGCACGGTTTCGTGAACGCG
AGCGATCCTTGTTTGGACATCAACCGCTCATCGTCTGTCGATTACATGTACACCCACGCATTGCGCTCTGAGTGTGC
AGCGTCTGGTGCTGAGAAGTTTGTGTTCTGGGATGTCACGCATCCAACAACAGCAACTCACCGCTATGTTGCAGAG
AAAATGCTAGAAAGTAGCAACAACTTAGCCGAGTACCGTTTCTAA

>EKP-026
ATGATGAAAAAAACAATCACACTATTAACTGCATTACTCCCGCTTGCTTCTGCAGTTGCCGAAGAGCCAA
CCTTATCACCAGAAATGGTTTCAGCGTCTGAAGTGATCAGCACGCAAGAAAACCAAACCTATACCTATGT
TCGCTGTTGGTATCGCACCAGCTACTCGAAAGATGATCCAGCGACCGATTGGGAATGGGCAAAAAACGAA
GATGGTAGCTACTTCACCATTGACGGCTACTGGTGGAGCTCCGTTTCATTTAAAAACATGTTCTACACCA
ACACGTCGCAAAACGTTATCCGTCAGCGTTGTGAAGCAACATTAGATTTGGCGAACGAGAACGCAGACAT
TACGTTCTTCGCCGCTGACAATCGCTTCTCATACAACCACACGATCTGGAGCAACGACGCAGCAATGCAG
CCAGATCAAATCAACAAAGTGGTTGCACTCGGTGACAGCTTGTCTGATACAGGCAACATCTTTAACGCAT
CACAATGGCGCTTCCCTAACCCGAACAGCTGGTTCTTAGGTCACTTCTCCAACGGTTTTGTTTGGACAGA
ATACATTGCCAAAGCGAAGAACCTTCCGCTCTACAACTGGGCAGTTGGCGGCGCGGCTGGTGAGAACCAA
TACATCGCGCTAACAGGGGTTGGTGAGCAAGTTTCTTCGTACTTAACCTACGCAAAACTGGCGAAGAACT
ACAAACCAGCAAACACCTTGTTTACGCTTGAGTTTGGTTTGAATGACTTCATGAACTACAACCGTGGCGT
TCCAGAAGTGAAAGCGGATTATGCAGAAGCACTGATTCGTTTGACGGACGCAGGTGCGAAGAACTTCATG
TTGATGACACTGCCAGATGCGACGAAAGCGCCTCAGTTTAAGTACTCAACACAAGAAGAGATCGACAAAA
TTCGTGCGAAAGTGCTTGAGATGAACGAGTTCATCAAGGCACAAGCGATGTACTACAAAGCGCAAGGTTA
CAACATCACGTTGTTTGATACTCACGCCTTGTTCGAGACGCTAACTTCTGCGCCAGAAGAGCACGGTTTC

GTGAACGCGAGCGATCCTTGTTTGGACATCAACCGCTCATCGTCTGTCGATTACATGTACACCCACGCAT
TGCGCTCTGAGTGTGCAGCGTCTGGTGCTGAGAAGTTTGTGTTCTGGGATGTCACGCATCCAACAACAGC
AACTCACCGCTATGTTGCAGAGAAAATGCTAGAAAGTAGCAACAACTTAGCCGAGTACCGTTTCTAA

>V14/01
ATGATGAAAAAAACAATCACACTATTAACTGCATTACTCCCGCTTGCTTCTGCAGTTGCCGAAGAGCCAA
CCTTATCACCAGAAATGGTTTCAGCGTCTGAAGTGATCAGCACGCAAGAAAACCAAACCTATACCTATGT
TCGCTGTTGGTATCGCACCAGCTACTCGAAAGATGATCCAGCGACCGATTGGGAATGGGCAAAAAACGAA
GATGGTAGCTACTTCACCATTGACGGCTACTGGTGGAGCTCCGTTTCATTTAAAAACATGTTCTACACCA
ACACGTCGCAAAACGTTATCCGTCAGCGTTGTGAAGCAACATTAGATTTGGCGAACGAGAACGCAGACAT
TACGTTCTTCGCCGCTGACAATCGCTTCTCATACAACCACACGATCTGGAGCAACGACGCAGCAATGCAG
CCAGATCAAATCAACAAAGTGGTTGCACTCGGTGACAGCTTGTCTGATACAGGCAACATCTTTAACGCAT
CACAATGGCGCTTCCCTAACCCGAACAGCTGGTTCTTAGGTCACTTCTCCAACGGTTTTGTTTGGACAGA
ATACATTGCCAAAGCGAAGAACCTTCCGCTCTACAACTGGGCAGTTGGCGGCGCGGCTGGTGAGAACCAA
TACATCGCGCTAACAGGGGTTGGTGAGCAAGTTTCTTCGTACTTAACCTACGCAAAACTGGCGAAGAACT
ACAAACCAGCAAACACCTTGTTTACGCTTGAGTTTGGTTTGAATGACTTCATGAACTACAACCGTGGCGT
TCCAGAAGTGAAAGCGGATTATGCAGAAGCACTGATTCGTTTGACGGACGCAGGTGCGAAGAACTTCATG
TTGATGACACTGCCAGATGCGACGAAAGCGCCTCAGTTTAAGTACTCAACACAAGAAGAGATCGACAAAA
TTCGTGCGAAAGTGCTTGAGATGAACGAGTTCATCAAGGCACAAGCGATGTACTACAAAGCGCAAGGTTA
CAACATCACGTTGTTTGATACTCACGCCTTGTTCGAGACGCTAACTTCTGCGCCAGAAGAGCACGGTTTC
GTGAACGCGAGCGATCCTTGTTTGGACATCAACCGCTCATCGTCTGTCGATTACATGTACACCCACGCAT
TGCGCTCTGAGTGTGCAGCGTCTGGTGCTGAGAAGTTTGTGTTCTGGGATGTCACGCATCCAACAACAGC
AACTCACCGCTATGTTGCAGAGAAAATGCTAGAAAGTAGCAACAACTTAGCCGAGTACCGTTTCTAA

>V-223/04
ATGATGAAAAAAACAATCACACTATTAACTGCATTACTCCCGCTTGCTTCTGCAGTTGCCGAAGAGCCAA
CCTTATCACCAGAAATGGTTTCAGCGTCTGAAGTGATCAGCACGCAAGAAAACCAAACCTATACCTATGT
TCGCTGTTGGTATCGCACCAGCTACTCGAAAGATGATCCAGCGACCGATTGGGAATGGGCAAAAAACGAA
GATGGTAGCTACTTCACCATTGACGGCTACTGGTGGAGCTCCGTTTCATTTAAAAACATGTTCTACACCA
ACACGTCGCAAAACGTTATCCGTCAGCGTTGTGAAGCAACATTAGATTTGGCGAACGAGAACGCAGACAT
TACGTTCTTCGCCGCTGACAATCGCTTCTCATACAACCACACGATCTGGAGCAACGACGCAGCAATGCAG
CCAGATCAAATCAACAAAGTGGTTGCACTCGGTGACAGCTTGTCTGATACAGGCAACATCTTTAACGCAT
CACAATGGCGCTTCCCTAACCCGAACAGCTGGTTCTTAGGTCACTTCTCCAACGGTTTTGTTTGGACAGA
ATACATTGCCAAAGCGAAGAACCTTCCGCTCTACAACTGGGCAGTTGGCGGCGCGGCTGGTGAGAACCAA
TACATCGCGCTAACAGGGGTTGGTGAGCAAGTTTCTTCGTACTTAACCTACGCAAAACTGGCGAAGAACT
ACAAACCAGCAAACACCTTGTTTACGCTTGAGTTTGGTTTGAATGACTTCATGAACTACAACCGTGGCGT
TCCAGAAGTGAAAGCGGATTATGCAGAAGCACTGATTCGTTTGACGGACGCAGGTGCGAAGAACTTCATG
TTGATGACACTGCCAGATGCGACGAAAGCGCCTCAGTTTAAGTACTCAACACAAGAAGAGATCGACAAAA
TTCGTGCGAAAGTGCTTGAGATGAACGAGTTCATCAAGGCACAAGCGATGTACTACAAAGCGCAAGGTTA
CAACATCACGTTGTTTGATACTCACGCCTTGTTCGAGACGCTAACTTCTGCGCCAGAAGAGCACGGTTTC
GTGAACGCGAGCGATCCTTGTTTGGACATCAACCGCTCATCGTCTGTCGATTACATGTACACCCACGCAT
TGCGCTCTGAGTGTGCAGCGTCTGGTGCTGAGAAGTTTGTGTTCTGGGATGTCACGCATCCAACAACAGC
AACTCACCGCTATGTTGCAGAGAAAATGCTAGAAAGTAGCAACAACTTAGCCGAGTACCGTTTCTAA

>VP2007-007
ATGATGAAAAAAACAATCACACTATTAACTGCATTACTCCCGCTTGCTTCTGCAGTTGCCGAAGAGCCAA
CCTTATCACCAGAAATGGTTTCAGCGTCTGAAGTGATCAGCACGCAAGAAAACCAAACCTATACCTATGT
TCGCTGTTGGTATCGCACCAGCTACTCGAAAGATGATCCGGCGACCGATTGGGAATGGGCAAAAAACGAA
GATGGTAGCTACTTCACCATTGACGGCTACTGGTGGAGCTCCGTTTCATTTAAAAACATGTTCTACACCA
ACACGTCGCAAAACGTTATCCGTCAGCGTTGTGAAGCAACATTAGATTTGGCGAACGAGAACGCAGATAT
TACGTTCTTCGCCGCTGACAATCGCTTCTCATACAACCACACGATCTGGAGCAACGACGCAGCAATGCAG

CCAGATCAAATCAACAAAGTGGTTGCACTCGGTGACAGCTTGTCTGATACAGGCAACATCTTTAACGCAT
CACAATGGCGCTTCCCTAACCCGAACAGCTGGTTCTTAGGTCACTTCTCCAACGGTTTTGTGTGGACAGA
ATACATTGCCAAAGCGAAGAACCTTCCGCTCTACAACTGGGCAGTTGGCGGCGCGGCTGGTGAGAACCAA
TACATCGCGCTAACAGGGGTTGGTGATCAAGTTTCTTCGTACTTAACCTACGCAAAACAGGCGAAGAACT
ACAAACCAGCAAACACCTTGTTTACGCTTGAGTTTGGTTTGAATGACTTCATGAACTACAACCGTGGCGT
TCCAGAAGTGAAAGCGGATTATGCAGAAGCACTGATTCGTTTGACGGACGCAGGTGCGAAGAACTTCATG
TTGATGACACTGCCAGACGCGACGAAAGCGCCTCAGTTTAAGTACTCAACACAAGAAGAGATCGACAAAA
TTCGTGCGAAAGTGCTTGAGATGAACGAGTTCATCAAGGCACAAGCGATGTACTACAAAGCGCAAGGTTA
CAACATCACGTTGTTTGATACTCACGCCTTGTTCGAGACGCTAACTTCTGCGCCAGAAGAGCACGGTTTC
GTGAACGCGAGCGATCCTTGTTTGGACATCAACCGCTCATCGTCTGTCGATTACATGTACACCCACGCAT
TGCGCTCTGAGTGTGCAGCGTCTGGTGCTGAGAAGTTTGTGTTCTGGGATGTGACTCACCCAACTACAGC
CACGCATCGTTATGTTGCTGAAAAAATGTTGGAAAGCAGCAATAACTTAGAAGAGTTTCGCTTTTAA

>EKP-021
ATGATGAAAAAAACAATCACACTATTAACTGCATTACTCCCGCTTGCTTCTGCAGTTGCCGAAGAGCCAA
CCTTATCACCAGAAATGGTTTCAGCGTCTGAAGTGATCAGCACGCAAGAAAACCAAACCTATACCTATGT
TCGCTGTTGGTATCGCACCAGCTACTCGAAAGATGATCCAGCGACCGATTGGGAATGGGCAAAAAACGAA
GATGGTAGCTACTTCACCATTGACGGCTACTGGTGGAGCTCCGTTTCATTTAAAAACATGTTCTACACCA
ACACGTCGCAAAACGTTATCCGTCAGCGTTGTGAAGCAACATTAGATTTGGCGAACGAGAACGCAGACAT
TACGTTCTTCGCCGCTGACAATCGCTTCTCATACAACCACACGATCTGGAGCAACGACGCAGCAATGCAG
CCAGATCAAATCAACAAAGTGGTTGCACTCGGTGACAGCTTGTCTGATACAGGCAACATCTTTAACGCAT
CACAATGGCGCTTCCCTAACCCGAACAGCTGGTTCTTAGGTCACTTCTCCAACGGTTTTGTTTGGACAGA
ATACATTGCCAAAGCGAAGAACCTTCCGCTCTACAACTGGGCAGTTGGCGGCGCGGCTGGTGAGAACCAA
TACATCGCGCTAACAGGGGTTGGTGAGCAAGTTTCTTCGTACTTAACCTACGCAAAACTGGCGAAGAACT
ACAAACCAGCAAACACCTTGTTTACGCTTGAGTTTGGTTTGAATGACTTCATGAACTACAACCGTGGCGT
TCCAGAAGTGAAAGCGGATTATGCAGAAGCACTGATTCGTTTGACGGACGCAGGTGCGAAGAACTTCATG
TTGATGACACTGCCAGATGCGACGAAAGCGCCTCAGTTTAAGTACTCAACACAAGAAGAGATCGACAAAA
TTCGTGCGAAAGTGCTTGAGATGAACGAGTTCATCAAGGCACAAGCGATGTACTACAAAGCGCAAGGTTA
CAACATCACGTTGTTTGATACTCACGCCTTGTTCGAGACGCTAACTTCTGCGCCAGAAGAGCACGGTTTC
GTGAACGCGAGCGATCCTTGTTTGGACATCAACCGCTCATCGTCTGTCGATTACATGTACACCCACGCAT
TGCGCTCTGAGTGTGCAGCGTCTGGTGCTGAGAAGTTTGTGTTCTGGGATGTCACGCATCCAACAACAGC
AACTCACCGCTATGTTGCAGAGAAAATGCTAGAAAGTAGCAACAACTTAGCCGAGTACCGTTTCTAA

>AQ3810-2
ATGATGAAAAAAACAATCACACTATTAACTGCATTACTCCCGCTTGCTTCTGCAGTTGCCGAAGAGCCAACCTTAT
CACCAGAAATGGTTTCAGCGTCTGAAGTGATCAGCACGCAAGAAAACCAAACCTATACCTATGTTCGCTGTTGGTA
TCGCACCAGCTACTCGAAAGATGATCCAGCGACCGATTGGGAATGGGCAAAAAACGAAGATGGTAGCTACTTCAC
CATTGACGGCTACTGGTGGAGCTCCGTTTCATTTAAAAACATGTTCTACACCAACACGTCGCAAAACGTTATCCGT
CAGCGTTGTGAAGCAACATTAGATTTGGCGAACGAGAACGCAGACATTACGTTCTTCGCCGCTGACAATCGCTTCT
CATACAACCACACGATCTGGAGCAACGACGCAGCAATGCAGCCAGATCAAATCAACAAAGTGGTTGCACTCGGTG
ACAGCTTGTCTGATACAGGCAACATCTTTAACGCATCACAATGGCGCTTCCCTAACCCGAACAGCTGGTTCTTAGG
TCACTTCTCCAACGGTTTTGTTTGGACAGAATACATTGCCAAAGCGAAGAACCTTCCGCTCTACAACTGGGCAGTT
GGCGGCGCGGCTGGTGAGAACCAATACATCGCGCTAACAGGGGTTGGTGAGCAAGTTTCTTCGTACTTAACCTACG
CAAAACTGGCGAAGAACTACAAACCAGCAAACACCTTGTTTACGCTTGAGTTTGGTTTGAATGACTTCATGAACTA
CAACCGTGGCGTTCCAGAAGTGAAAGCGGATTATGCAGAAGCACTGATTCGTTTGACGGACGCAGGTGCGAAGAA
CTTCATGTTGATGACACTGCCAGATGCGACGAAAGCGCCTCAGTTTAAGTACTCAACACAAGAAGAGATCGACAA
AATTCGTGCGAAAGTGCTTGAGATGAACGAGTTCATCAAGGCACAAGCGATGTACTACAAAGCGCAAGGTTACAA
CATCACGTTGTTTGATACTCACGCCTTGTTCGAGACGCTAACTTCTGCGCCAGAAGAGCACGGTTTCGTGAACGCG
AGCGATCCTTGTTTGGACATCAACCGCTCATCGTCTGTCGATTACATGTACACCCACGCATTGCGCTCTGAGTGTGC

AGCGTCTGGTGCTGAGAAGTTTGTGTTCTGGGATGTCACGCATCCAACAACAGCAACTCACCGCTATGTTGCAGAG
AAAATGCTAGAAAGTAGCAACAACTTAGCCGAGTACCGTTTCTAA

>VP-48
ATGATGAAAAAAACAATCACACTATTAACTGCATTACTCCCGCTTGCTTCTGCAGTTGCCGAAGAGCCAACCTTAT
CACCAGAAATGGTTTCAGCGTCTGAAGTGATCAGCACGCAAGAAAACCAAACCTATACCTATGTTCGCTGTTGGTA
TCGCACCAGCTACTCGAAAGATGATCCAGCGACCGATTGGGAATGGGCAAAAAACGAAGATGGTAGCTACTTCAC
CATTGACGGCTACTGGTGGAGCTCCGTTTCATTTAAAAACATGTTCTACACCAACACGTCGCAAAACGTTATCCGT
CAGCGTTGTGAAGCAACATTAGATTTGGCGAACGAGAACGCAGACATTACGTTCTTCGCCGCTGACAATCGCTTCT
CATACAACCACACGATCTGGAGCAACGACGCAGCAATGCAGCCAGATCAAATCAACAAAGTGGTTGCACTCGGTG
ACAGCTTGTCTGATACAGGCAACATCTTTAACGCATCACAATGGCGCTTCCCTAACCCGAACAGCTGGTTCTTAGG
TCACTTCTCCAACGGTTTTGTTTGGACAGAATACATTGCCAAAGCGAAGAACCTTCCGCTCTACAACTGGGCAGTT
GGCGGCGCGGCTGGTGAGAACCAATACATCGCGCTAACAGGGGTTGGTGAGCAAGTTTCTTCGTACTTAACCTACG
CAAAACTGGCGAAGAACTACAAACCAGCAAACACCTTGTTTACGCTTGAGTTTGGTTTGAATGACTTCATGAACTA
CAACCGTGGCGTTCCAGAAGTGAAAGCGGATTATGCAGAAGCACTGATTCGTTTGACGGACGCAGGTGCGAAGAA
CTTCATGTTGATGACACTGCCAGATGCGACGAAAGCGCCTCAGTTTAAGTACTCAACACAAGAAGAGATCGACAA
AATTCGTGCGAAAGTGCTTGAGATGAACGAGTTCATCAAGGCACAAGCGATGTACTACAAAGCGCAAGGTTACAA
CATCACGTTGTTTGATACTCACGCCTTGTTCGAGACGCTAACTTCTGCGCCAGAAGAGCACGGTTTCGTGAACGCG
AGCGATCCTTGTTTGGACATCAACCGCTCATCGTCTGTCGATTACATGTACACCCACGCATTGCGCTCTGAGTGTGC
AGCGTCTGGTGCTGAGAAGTTTGTGTTCTGGGATGTCACGCATCCAACAACAGCAACTCACCGCTATGTTGCAGAG
AAAATGCTAGAAAGTAGCAACAACTTAGCCGAGTACCGTTTCTAA

>VPCR-2009
ATGATGAAAAAAACAATCACACTATTAACTGCATTACTCCCGCTTGCTTCTGCAGTTGCCGAAGAGCCAACCTTAT
CACCAGAAATGGTTTCAGCGTCTGAAGTGATCAGCACGCAAGAAAACCAAACCTATACCTATGTTCGCTGTTGGTA
TCGCACCAGCTACTCGAAAGATGATCCGGCGACCGATTGGGAATGGGCAAAAAACGAAGATGGTAGCTACTTCAC
CATTGACGGCTACTGGTGGAGCTCCGTTTCATTTAAAAACATGTTCTACACCAACACCTCGCAAAACGTTATCCGT
CAGCGTTGTGAAGCCACATTAGATTTGGCGAACGAGAACGCAGACATTACGTTCTTCGCCGCTGACAATCGCTTCT
CATACAACCACACGATCTGGAGCAACGACGCAGCAATGCAGCCAGATCAAATCAACAAAGTGGTTGCACTCGGTG
ACAGCTTGTCTGATACAGGCAACATCTTTAACGCATCACAATGGCGCTTCCCTAACCCGAATAGCTGGTTCTTAGG
TCACTTCTCCAACGGTTTTGTGTGGACAGAATACATTGCCAAAGCGAAGAACCTTCCACTCTACAACTGGGCAGTT
GGCGGCGCGGCTGGTGAGAACCAATACATCGCGCTAACAGGGGTTGGTGATCAAGTTTCTTCGTACTTAACCTACG
CAAAACTGGCGAAGAACTACAAACCAGCAAACACCTTGTTTACGCTTGAGTTTGGTTTGAATGACTTCATGAACTA
CAACCGTGGCGTTCCAGAAGTGAAAGCAGATTATGCAGAAGCACTGATTCGTTTGACGGACGCAGGTGCGAAGAA
CTTCATGTTGATGACACTGCCAGACGCGACGAAAGCGCCTCAGTTTAAGTACTCAACACAAGAAGAGATCGACAA
AATTCGTGCGAAAGTGCTTGAGATGAACGAGTTCATCAAGGCACAAGCGATGTACTACAAAGCGCAAGGTTACAA
CATCACGTTGTTTGATACTCACGCCTTGTTCGAGACGCTAACTTCTGCGCCAGAAGAGCACGGTTTCGTGAACGCG
AGCGATCCTTGTTTGGACATCAACCGCTCATCGTCTGTCGACTACATGTACACCCACGCATTGCGCTCTGAGTGTGC
GGCGTCCGGTGCTGAGAAATTTGTGTTCTGGGATGTCACGCACCCAACAACAGCAACTCACCGCTATGTTGCAGAG
AAAATGCTAGAAAGTAGCAACAACTTAGCCGAGTACCGTTTCTAA

>VPTS-2009
ATGATGAAAAAAACAATCACACTATTAACTGCATTACTCCCGCTTGCTTCTGCAGTTGCCGAAGAGCCAACCTTAT
CACCAGAAATGGTTTCAGCGTCTGAAGTGATCAGCACGCAAGAAAACCAAACCTATACCTATGTTCGCTGTTGGTA
TCGCACCAGCTACTCGAAAGATGATCCGGCGACCGATTGGGAATGGGCAAAAAACGAAGATGGTAGCTACTTCAC
CATTGACGGCTACTGGTGGAGCTCCGTCTCATTTAAAAACATGTTCTACACCAACACGTCGCAAAACGTTATCCGT
CAGCGTTGTGAAGCAACATTAGATTTGGCGAACGAGAACGCAGACATTACGTTCTTCGCCGCTGACAATCGCTTCT
CATACAACCACACGATCTGGAGCAACGACGCAGCAATGCAGCCAGATCAAATCAACAAAGTGGTTGCACTCGGTG
ACAGCTTGTCTGATACAGGCAACATCTTTAACGCATCACAATGGCGCTTCCCTAACCCGAACAGCTGGTTCTTAGG
TCACTTCTCCAACGGTTTTGTGTGGACAGAATACATTGCCAAAGCGAAGAACCTTCCGCTCTACAACTGGGCAGTT
GGCGGCGCGGCTGGTGAGAACCAATACATCGCGCTAACAGGGGTTGGTGATCAAGTTTCTTCGTACTTAACCTACG

CAAAACTGGCGAAGAACTACAAACCAGCAAACACATTGTTTACGCTTGAGTTTGGTTTGAATGACTTCATGAACTA
CAACCGTGGCGTTCCAGAAGTGAAAGCGGATTATGCAGAAGCACTGATTCGTTTGACGGACGCAGGTGCGAAGAA
CTTCATGTTGATGACACTGCCAGACGCGACGAAAGCGCCTCAGTTTAAGTACTCAACACAAGAAGAGATCGACAA
AATTCGTGCGAAAGTGCTTGAGATGAACGAGTTCATCAAGGCACAAGCGATGTACTACAAAGCGCAAGGTTACAA
CATCACGTTGTTTGATACTCACGCCTTGTTCGAGACGCTAACTTCTGCGCCAGAAGAGCACGGTTTCGTGAACGCG
AGTGATCCTTGTTTGGACATCAACCGCTCATCGTCTGTCGATTACATGTACACCCACGCATTGCGCTCTGAGTGTGC
GGCGTCTGGTGCTGAGAAATTTGTGTTCTGGGATGTCACGCACCCGACAACAGCAACTCACCGCTATGTTGCAGAG
AAAATGCTAGAAAGTAGCAACAACTTAGCCGAGTACCGTTTCTAA

>VPTS-2010
ATGATGAAAAAAACAATCACACTATTAACTGCATTACTCCCGCTTGCTTCTGCAGTTGCCGAAGAGCCAA
CCTTATCACCAGAAATGGTTTCAGCGTCTGAAGTGATCAGCACGCAAGAAAACCAAACCTATACCTATGT
TCGCTGTTGGTATCGCACCAGCTACTCGAAAGATGATCCGGCGACCGATTGGGAATGGGCAAAAAACGAA
GATGGTAGCTACTTCACCATTGACGGCTACTGGTGGAGCTCCGTTTCATTTAAAAACATGTTCTACACCA
ACACGTCGCAAAACGTTATCCGTCAGCGTTGTGAAGCAACATTAGATTTGGCGAACGAGAACGCAGACAT
TACGTTCTTCGCCGCTGACAATCGCTTCTCATACAACCACACGATCTGGAGCAACGACGCAGCAATGCAG
CCAGATCAAATCAACAAAGTGGTTGCACTCGGTGACAGCTTGTCTGATACAGGCAACATCTTTAACGCAT
CACAATGGCGCTTCCCTAACCCGAACAGCTGGTTCTTAGGTCACTTCTCCAACGGTTTTGTGTGGACAGA
ATACATTGCCAAAGCGAAGAACCTTCCGCTCTACAACTGGGCAGTTGGCGGCGCGGCTGGTGAGAACCAA
TACATCGCGCTAACAGGGGTTGGTGATCAAGTTTCTTCGTACTTAACCTACGCAAAACTGGCGAAGAACT
ACAAACCAGCAAACACCTTGTTTACGCTTGAGTTTGGTTTGAATGACTTCATGAACTACAACCGTGGCGT
TCCAGAAGTGAAAGCAGATTATGCAGAAGCACTGATTCGTTTGACGGACGCAGGTGCGAAGAACTTCATG
TTGATGACACTGCCAGACGCGACGAAAGCGCCTCAGTTTAAGTACTCAACACAAGAAGAGATCGACAAAA
TTCGTGCGAAAGTGCTTGAGATGAACGAGTTCATCAAGGCACAAGCGATGTACTACAAAGCGCAAGGTTA
CAACATCACGTTGTTTGATACTCACGCCTTGTTCGAGACGCTAACTTCTGCGCCCGAAGAGCACGGTTTC
GTGAACGCGAGTGATCCTTGTTTGGACATCAACCGCTCATCGTCTGTCGACTACATGTACACCCACGCAT
TGCGCTCTGAGTGTGCGGCGTCTGGTGCTGAGAAGTTTGTGTTCTGGGATGTCACGCACCCAACAACAGC
AACTCACCGCTATGTTGCAGAGAAAATGCTAGAAAGTAGCAACAACTTAGCCGAGTACCGTTTCTAA

>VPTS-2010_2
ATGATGAAAAAAACAATCACACTATTAACTGCATTACTCCCGCTTGCTTCTGCAGTTGCCGAAGAGCCAACCTTAT
CACCAGAAATGGTTTCAGCGTCTGAAGTGATCAGCACGCAAGAAAACCAAACCTATACCTATGTTCGCTGTTGGTA
TCGCACCAGCTACTCGAAAGATGATCCGGCGACCGATTGGGAATGGGCAAAAAACGAAGATGGTAGCTACTTCAC
CATTGACGGCTACTGGTGGAGCTCCGTTTCATTTAAAAACATGTTCTACACCAACACGTCGCAAAACGTTATCCGT
CAGCGTTGTGAAGCAACATTAGATTTGGCGAACGAGAACGCAGACATTACGTTCTTCGCCGCTGACAATCGCTTCT
CATACAACCACACGATCTGGAGCAACGACGCAGCAATGCAGCCAGATCAAATCAACAAAGTGGTTGCACTCGGTG
ACAGCTTGTCTGATACAGGCAACATCTTTAACGCATCACAATGGCGCTTCCCTAACCCGAACAGCTGGTTCTTAGG
TCACTTCTCCAACGGTTTTGTTTGGACAGAATACATTGCCAAAGCGAAGAACCTTCCGCTCTACAACTGGGCAGTT
GGCGGCGCGGCTGGTGAGAACCAATACATCGCGCTAACAGGGGTTGGTGATCAAGTTTCTTCGTACTTAACCTACG
CAAAACTGGCGAAGAACTACAAACCAGCAAACACCTTGTTTACGCTTGAGTTTGGTTTGAATGACTTCATGAACTA
CAACCGTGGCGTTCCAGAAGTGAAAGCGGATTATGCAGAAGCACTGATTCGTTTGACGGACGCAGGTGCGAAGAA
CTTCATGTTGATGACACTGCCAGATGCGACGAAAGCGCCTCAGTTTAAGTACTCAACACAAGAAGAGATCGACAA
AATTCGTGCGAAAGTGCTTGAGATGAACGAGTTCATCAAGGCACAAGCGATGTACTACAAAGCGCAAGGTTACAA
CATCACGTTGTTTGATACTCACGCCTTGTTCGAGACGCTAACTTCTGCGCCAGAAGAGCACGGTTTCGTGAACGCG
AGCGATCCTTGTTTGGACATCAACCGCTCATCGTCTGTCGATTACATGTACACCCACGCATTGCGCTCTGAGTGTGC
GGCGTCTGGTGCTGAGAAGTTTGTGTTCTGGGATGTCACGCACCCAACAACAGCAACTCACCGCTATGTTGCAGAG
AAAATGCTAGAAAGTAGCAACAACTTAGCCGAGTACCGTTTCTAA

>T12739
ATGATGAAAAAAACAATCACACTATTAACTGCATTACTCCCGCTTGCTTCTGCAGTTGCCGAAGAGCCAA
CCTTATCACCAGAAATGGTTTCAGCGTCTGAAGTGATCAGCACGCAAGAAAACCAAACCTATACCTATGT

TCGCTGTTGGTATCGCACCAGCTACTCGAAAGATGACCCGGCGACCGATTGGGAATGGGCAAAAAACGAA
GATGGTAGCTACTTCACCATTGACGGCTACTGGTGGAGCTCCGTTTCATTTAAAAACATGTTCTACACCA
ACACGTCGCAAAACGTTATCCGTCAGCGTTGTGAAGCAACATTAGATTTGGCGAACGAGAACGCAGACAT
TACGTTCTTCGCCGCTGACAATCGCTTCTCATACAACCACACGATCTGGAGCAACGACGCAGCAATGCAG
CCAGATCAAATCAACAAAGTGGTTGCACTCGGTGACAGCTTGTCTGATACAGGCAACATCTTTAACGCAT
CACAATGGCGCTTCCCTAACCCGAACAGCTGGTTCTTAGGTCACTTCTCCAACGGTTTTGTTTGGACAGA
ATACATTGCCAAAGCGAAGAACCTTCCGCTCTACAACTGGGCAGTTGGCGGCGCGGCTGGTGAGAACCAA
TACATCGCGCTAACAGGGGTTGGTGAGCAAGTTTCTTCGTACTTAACCTACGCAAAACTGGCGAAGAACT
ACAAACCAGCAAACACCTTGTTTACGCTTGAGTTTGGTTTGAATGACTTCATGAACTACAACCGTGGCGT
TCCAGAAGTGAAAGCGGATTATGCAGAAGCACTGATTCGTTTGACGGACGCAGGTGCGAAGAACTTCATG
TTGATGACACTGCCAGATGCGACGAAAGCGCCTCAGTTTAAGTACTCAACACAAGAAGAGATCGACAAAA
TTCGTGCGAAAGTGCTTGAGATGAACGAGTTCATCAAGGCACAAGCGATGTACTACAAAGCGCAAGGTTA
CAACATCACGTTGTTTGATACTCACGCCTTGTTCGAGACGCTAACTTCTGCGCCAGAAGAGCACGGTTTC
GTGAACGCGAGCGATCCTTGTTTGGACATCAACCGCTCATCGTCTGTCGATTACATGTACACCCACGCAT
TGCGCTCTGAGTGTGCAGCGTCTGGTGCTGAGAAGTTTGTGTTCTGGGATGTCACGCATCCAACAACAGC
AACTCACCGCTATGTTGCAGAGAAAATGCTAGAAAGTAGCAACAACTTAGCCGAGTACCGTTTCTAA

>T9109
ATGATGAAAAAAACAATCACACTATTAACTGCATTACTCCCGCTTGCTTCTGCAGTTGCCGAAGAGCCAA
CCTTATCACCAGAAATGGTTTCAGCGTCTGAAGTGATCAGCACGCAAGAAAACCAAACCTATACCTATGT
TCGCTGTTGGTATCGCACCAGCTACTCGAAAGATGACCCGGCGACCGATTGGGAATGGGCAAAAAACGAA
GATGGTAGCTACTTCACCATTGACGGCTACTGGTGGAGCTCCGTTTCATTTAAAAACATGTTCTACACCA
ACACGTCGCAAAACGTTATCCGTCAGCGTTGTGAAGCAACATTAGATTTGGCGAACGAGAACGCAGACAT
TACGTTCTTCGCCGCTGACAATCGCTTCTCATACAACCACACGATCTGGAGCAACGACGCAGCAATGCAG
CCAGATCAAATCAACAAAGTGGTTGCACTCGGTGACAGCTTGTCTGATACAGGCAACATCTTTAACGCAT
CACAATGGCGCTTCCCTAACCCGAACAGCTGGTTCTTAGGTCACTTCTCCAACGGTTTTGTTTGGACAGA
ATACATTGCCAAAGCGAAGAACCTTCCGCTCTACAACTGGGCAGTTGGCGGCGCGGCTGGTGAGAACCAA
TACATCGCGCTAACAGGGGTTGGTGAGCAAGTTTCTTCGTACTTAACCTACGCAAAACTGGCGAAGAACT
ACAAACCAGCAAACACCTTGTTTACGCTTGAGTTTGGTTTGAATGACTTCATGAACTACAACCGTGGCGT
TCCAGAAGTGAAAGCGGATTATGCAGAAGCACTGATTCGTTTGACGGACGCAGGTGCGAAGAACTTCATG
TTGATGACACTGCCAGATGCGACGAAAGCGCCTCAGTTTAAGTACTCAACACAAGAAGAGATCGACAAAA
TTCGTGCGAAAGTGCTTGAGATGAACGAGTTCATCAAGGCACAAGCGATGTACTACAAAGCGCAAGGTTA
CAACATCACGTTGTTTGATACTCACGCCTTGTTCGAGACGCTAACTTCTGCGCCAGAAGAGCACGGTTTC
GTGAACGCGAGCGATCCTTGTTTGGACATCAACCGCTCATCGTCTGTCGATTACATGTACACCCACGCAT
TGCGCTCTGAGTGTGCAGCGTCTGGTGCTGAGAAGTTTGTGTTCTGGGATGTCACGCATCCAACAACAGC
AACTCACCGCTATGTTGCAGAGAAAATGCTAGAAAGTAGCAACAACTTAGCCGAGTACCGTTTCTAA

>4.2548
ATGATGAAAAAAACAATCACACTATTAACTGCATTACTCCCGCTTGCTTCTGCAGTTGCCGAAGAGCCAACCTTAT
CACCAGAAATGGTTTCAGCGTCTGAAGTGATCAGCACGCAAGAAAACCAAACCTATACCTATGTTCGCTGTTGGTA
TCGCACCAGCTACTCGAAAGATGATCCGGCGACCGATTGGGAATGGGCAAAAAACGAAGATGGTAGCTACTTCAC
CATTGACGGCTACTGGTGGAGCTCCGTTTCATTTAAAAACATGTTCTACACCAACACGTCGCAAAACGTTATCCGT
CAGCGTTGTGAAGCAACATTAGATTTGGCGAACGAGAACGCAGACATTACGTTCTTCGCCGCTGACAATCGCTTCT
CATACAACCACACGATCTGGAGCAACGACGCAGCAATGCAGCCAGATCAAATCAACAAAGTGGTTGCACTCGGTG
ACAGCTTGTCTGATACAGGCAACATCTTTAACGCATCACAATGGCGCTTCCCTAACCCGAATAGCTGGTTCTTAGG
TCACTTCTCCAACGGTTTTGTGTGGACAGAATACATTGCCAAAGCGAAGAACCTTCCACTCTACAACTGGGCAGTT
GGCGGCGCGGCTGGTGAGAACCAATACATCGCGCTAACAGGGGTTGGTGATCAAGTTTCTTCGTACTTAACCTACG
CAAAACTGGCGAAGAACTACAAACCAGCAAACACCTTGTTTACGCTTGAGTTTGGTTTGAATGACTTCATGAACTA
CAACCGTGGCGTTCCAGAAGTGAAAGCAGATTATGCAGAAGCACTGATTCGTTTGACGGACGCAGGTGCGAAGAA
CTTCATGTTGATGACACTGCCAGACGCGACGAAAGCGCCTCAGTTTAAGTACTCAACACAAGAAGAGATCGACAA
AATTCGTGCGAAAGTGCTTGAGATGAACGAGTTCATCAAGGCACAAGCGATGTACTACAAAGCGCAAGGTTACAA

CATAACGTTGTTTGATACTCACGCCTTGTTCGAGACGCTAACTTCTGCGCCCGAAGAGCACGGTTTCGTGAACGCG
AGCGATCCTTGTTTGGACATCAACCGCTCATCGTCTGTCGATTACATGTACACCCACGCATTGCGCTCTGAGTGTGC
AGCGTCTGGTGCTGAGAAGTTTGTGTTCTGGGATGTCACGCACCCAACAACAGCAACTCACCGCTATGTTGCAGAG
AAAATGCTAGAAAGTAGCAACAACTTAGCCGAGTACCGTTTCTAA

>9.5357
ATGATGAAAAAAACAATCACACTATTAACTGCATTACTCCCGCTTGCTTCTGCAGTTGCCGAAGAGCCAA
CCTTATCACCAGAAATGGTTTCAGCGTCTGAAGTGATCAGCACGCAAGAAAACCAAACCTATACCTATGT
TCGCTGTTGGTATCGCACCAGCTACTCGAAAGATGATCCGGCGACCGATTGGGAATGGGCAAAAAACGAA
GATGGTAGCTACTTCACCATTGACGGCTACTGGTGGAGCTCCGTTTCATTTAAAAACATGTTCTACACCA
ACACGTCGCAAAACGTTATCCGTCAGCGTTGTGAAGCAACATTAGATTTGGCGAACGAGAACGCAGACAT
TACGTTCTTCGCCGCTGACAATCGCTTCTCATACAACCACACGATCTGGAGCAACGACGCAGCAATGCAG
CCAGATCAAATCAACAAAGTGGTTGCACTCGGTGACAGCTTGTCTGATACAGGCAACATCTTTAACGCAT
CACAATGGCGCTTCCCTAACCCGAACAGCTGGTTCTTAGGTCACTTCTCCAACGGTTTTGTGTGGACAGA
ATACATTGCCAAAGCGAAGAACCTTCCGCTCTACAACTGGGCAGTTGGCGGCGCGGCTGGTGAGAACCAA
TACATCGCGCTAACAGGGGTTGGTGATCAAGTTTCTTCGTACTTAACTTACGCAAAACTGGCGAAGAACT
ACAAACCAGCAAACACCTTGTTTACGCTTGAGTTTGGTTTGAATGACTTCATGAACTACAACCGTGGCGT
TCCAGAAGTGAAAGCGGATTATGCAGAAGCACTGATTCGTTTGACGGACGCAGGTGCGAAGAACTTCATG
TTGATGACACTGCCAGACGCGACGAAAGCGCCTCAGTTTAAGTACTCAACACAAGAAGAGATCGACAAAA
TTCGTGCGAAAGTGCTTGAGATGAACGAGTTCATCAAGGCACAAGCGATGTACTACAAAGCGCAAGGTTA
CAACATCACGTTGTTTGATACTCACGCCTTGTTCGAGACGCTAACTTCTGCGCCAGAAGAGCACGGTTTC
GTGAACGCGAGTGATCCTTGTTTGGACATCAACCGCTCATCGTCTGTCGATTACATGTACACCCACGCAT
TGCGCTCTGAGTGTGCGGCGTCCGGTGCTGAGAAATTTGTGTTCTGGGATGTCACGCACCCAACAACAGC
AACTCACCGCTATGTTGCAGAGAAAATGCTAGAAAGTAGCAACAACTTAGCCGAGTACCGTTTCTAA

>08-0278
ATGATGAAAAAAACAATCACACTATTAACTGCATTACTCCCGCTTGCTTCTGCAGTTGCCGAAGAGCCAACCTTAT
CACCAGAAATGGTTTCAGCGTCTGAAGTGATCAGCACGCAAGAAAACCAAACCTATACCTATGTTCGCTGTTGGTA
TCGCACCAGCTACTCGAAAGATGATCCGGCGACCGATTGGGAATGGGCAAAAAACGAAGATGGTAGCTACTTCAC
CATTGACGGCTACTGGTGGAGCTCCGTTTCATTTAAAAACATGTTCTACACCAACACGTCGCAAAACGTTATCCGT
CAGCGTTGTGAAGCAACATTAGATTTGGCGAACGAGAACGCAGACATTACGTTCTTCGCCGCTGACAATCGCTTCT
CATACAACCACACGATCTGGAGCAACGACGCAGCAATGCAGCCAGATCAAATCAACAAAGTGGTTGCACTCGGTG
ACAGCTTGTCTGATACAGGCAACATCTTTAACGCATCACAATGGCGCTTCCCTAACCCGAACAGCTGGTTCTTAGG
TCACTTCTCCAACGGTTTTGTGTGGACAGAATACATTGCCAAAGCGAAGAACCTTCCGCTCTACAACTGGGCAGTT
GGCGGCGCGGCTGGTGAGAACCAATACATCGCGCTAACAGGGGTTGGTGATCAAGTTTCTTCGTACTTAACCTACG
CAAAACTGGCGAAGAACTACAAACCAGCAAACACCTTGTTTACGCTTGAGTTTGGTTTGAATGACTTCATGAACTA
CAACCGTGGCGTTCCAGAAGTGAAAGCAGATTATGCAGAAGCACTGATTCGTTTGACGGACGCAGGTGCGAAGAA
CTTCATGTTGATGACACTGCCAGACGCGACGAAAGCGCCTCAGTTTAAGTACTCAACACAAGAAGAGATCGACAA
AATTCGTGCGAAAGTGCTTGAGATGAACGAGTTCATCAAGGCACAAGCGATGTACTACAAAGCGCAAGGTTACAA
CATCACGTTGTTTGATACTCACGCCTTGTTCGAGACGCTAACTTCTGCGCCAGAAGAGCACGGTTTCGTGAACGCG
AGTGATCCTTGTTTGGACATCAACCGCTCATCGTCTGTCGATTACATGTACACCCACGCATTGCGCTCTGAGTGTGC
GGCGTCTGGTGCTGAGAAGTTTGTGTTCTGGGATGTCACGCACCCAACAACAGCAACTCACCGCTATGTTGCAGAG
AAAATGCTAGAAAGTAGCAACAACTTAGCCGAGTACCGTTTCTAA

>09-3217
ATGATGAAAAAAACAATCACACTATTAACTGCATTACTCCCGCTTGCTTCTGCAGTTGCCGAAGAGCCAA
CCTTATCACCAGAAATGGTTTCAGCGTCTGAAGTGATCAGCACGCAAGAAAACCAAACCTATACCTATGT
TCGCTGTTGGTATCGCACCAGCTACTCGAAAGATGATCCGGCGACCGATTGGGAATGGGCAAAAAACGAA
GATGGTAGCTACTTCACCATTGACGGCTACTGGTGGAGCTCCGTTTCATTTAAAAACATGTTCTACACCA
ACACGTCGCAAAACGTTATCCGTCAGCGTTGTGAAGCAACATTAGATTTGGCGAACGAGAACGCAGACAT
TACGTTCTTCGCCGCTGACAATCGCTTCTCATACAACCACACGATCTGGAGCAACGACGCAGCAATGCAG

CCAGATCAAATCAACAAAGTGGTTGCACTCGGTGACAGCTTGTCTGATACAGGCAACATCTTTAACGCAT
CACAATGGCGCTTCCCTAACCCGAACAGCTGGTTCTTAGGTCACTTCTCCAACGGTTTTGTGTGGACAGA
ATACATTGCCAAAGCGAAGAACCTTCCGCTCTACAACTGGGCAGTTGGCGGTGCGGCTGGTGAGAACCAA
TACATCGCGCTAACAGGGGTTGGTGATCAAGTTTCTTCGTACTTAACCTACGCAAAACTGGCGAAGAACT
ACAAACCAGCAAACACCTTGTTTACGCTTGAGTTTGGTTTGAATGACTTCATGAACTACAACCGTGGCGT
TCCAGAAGTGAAAGCGGATTATGCAGAAGCACTGATTCGTTTGACGGACGCAGGTGCGAAGAACTTCATG
TTGATGACACTGCCAGATGCGACGAAAGCGCCTCAGTTTAAGTACTCAACACAAGAAGAGATCGACAAAA
TTCGTGCGAAAGTGCTTGAGATGAACGAGTTCATCAAGGCACAAGCGATGTACTACAAAGCGCAAGGTTA
CAACATCACGTTGTTTGATACTCACGCCTTGTTCGAGACGCTAACTTCTGCGCCAGAAGAGCACGGTTTC
GTGAACGCGAGCGATCCTTGTTTGGACATCAACCGCTCATCGTCTGTCGATTACATGTACACCCACGCAT
TGCGCTCTGAGTGTGCAGCGTCTGGTGCTGAGAAGTTTGTGTTCTGGGATGTCACGCACCCAACAACAGC
AACTCACCGCTATGTTGCAGAGAAAATGCTAGAAAGTAGCAACAACTTAGAAGAGTTTCGCTTTTAA

>08-7626
ATGATGAAAAAAACAATCACACTATTAACTGCATTACTCCCGCTTGCTTCTGCAGTTGCCGAAGAGCCAA
CCTTATCACCAGAAATGGTTTCAGCGTCTGAAGTGATCAGCACGCAAGAAAACCAAACCTATACCTATGT
TCGCTGTTGGTATCGCACCAGCTACTCGAAAGATGATCCGGCGACCGATTGGGAATGGGCAAAAAACGAA
GATGGTAGCTACTTCACCATTGACGGCTACTGGTGGAGCTCCGTTTCATTTAAAAACATGTTCTACACCA
ACACGTCGCAAAACGTTATCCGTCAGCGTTGTGAAGCCACATTAGATTTGGCGAACGAGAACGCAGACAT
TACGTTCTTCGCCGCTGACAATCGCTTCTCATACAACCACACGATCTGGAGCAACGACGCAGCAATGCAG
CCAGATCAAATCAACAAAGTGGTTGCACTCGGTGACAGCTTGTCTGATACAGGCAACATCTTTAACGCAT
CACAATGGCGCTTCCCTAACCCGAACAGCTGGTTCTTAGGTCACTTCTCCAACGGTTTTGTGTGGACAGA
ATACATTGCCAAAGCGAAGAACCTTCCGCTCTACAACTGGGCAGTTGGCGGCGCGGCTGGTGAGAACCAA
TACATCGCGCTAACAGGGGTTGGTGATCAAGTTTCTTCGTACTTAACCTACGCAAAACTGGCGAAGAACT
ACAAACCAGCAAACACCTTGTTTACGCTTGAGTTTGGTTTGAATGACTTCATGAACTACAACCGTGGCGT
TCCAGAAGTGAAAGCAGATTATGCAGAAGCACTGATTCGTTTGACGGACGCAGGTGCGAAGAACTTCATG
TTGATGACACTGCCAGACGCGACGAAAGCGCCTCAGTTTAAGTACTCAACACAAGAAGAGATCGACAAAA
TTCGTGCGAAAGTGCTTGAGATGAACGAGTTCATCAAGGCACAAGCGATGTACTACAAAGCGCAAGGTTA
CAACATCACGTTGTTTGATACTCACGCCTTGTTCGAGACGCTAACTTCTGCGCCCGAAAAGCACGGTTTC
GTGAACGCGAGTGATCCTTGTTTGGACATCAACCGCTCATCGTCTGTCGATTACATGTACACCCACGCAT
TGCGCTCTGAGTGTGCGGCGTCTGGTGCTGAGAAGTTTGTGTTCTGGGATGTCACGCACCCAACAACAGC
AACTCACCGCTATGTTGCAGAGAAAATGCTAGAAAGTAGCAACAACTTAGCCGAGTACCGTTTCTAA

>04-2551
ATGATGAAAAAAACAATCACACTATTAACTGCATTACTCCCGCTTGCTTCTGCAGTTGCCGAAGAGCCAA
CCTTATCACCAGAAATGGTTTCAGCGTCTGAAGTGATCAGCACGCAAGAAAACCAAACCTATACCTATGT
TCGCTGTTGGTATCGCACCAGCTACTCGAAAGATGATCCAGCGACCGATTGGGAATGGGCAAAAAACGAA
GATGGTAGCTACTTCACCATTGACGGCTACTGGTGGAGCTCCGTTTCATTTAAAAACATGTTCTACACCA
ACACGTCGCAAAACGTTATCCGTCAGCGTTGTGAAGCAACATTAGATTTGGCGAACGAGAACGCAGACAT
TACGTTCTTCGCCGCTGACAATCGCTTCTCATACAACCACACGATCTGGAGCAACGACGCAGCAATGCAG
CCAGATCAAATCAACAAAGTGGTTGCACTCGGTGACAGCTTGTCTGATACAGGCAACATCTTTAACGCAT
CACAATGGCGCTTCCCTAACCCGAACAGCTGGTTCTTAGGTCACTTCTCCAACGGTTTTGTTTGGACAGA
ATACATTGCCAAAGCGAAGAACCTTCCGCTCTACAACTGGGCAGTTGGCGGCGCGGCTGGTGAGAACCAA
TACATCGCGCTAACAGGGGTTGGTGAGCAAGTTTCTTCGTACTTAACCTACGCAAAACTGGCGAAGAACT
ACAAACCAGCAAACACCTTGTTTACGCTTGAGTTTGGTTTGAATGACTTCATGAACTACAACCGTGGCGT
TCCAGAAGTGAAAGCGGATTATGCAGAAGCACTGATTCGTTTGACGGACGCAGGTGCGAAGAACTTCATG
TTGATGACACTGCCAGATGCGACGAAAGCGCCTCAGTTTAAGTACTCAACACAAGAAGAGATCGACAAAA
TTCGTGCGAAAGTGCTTGAGATGAACGAGTTCATCAAGGCACAAGCGATGTACTACAAAGCGCAAGGTTA

CAACATCACGTTGTTTGATACTCACGCCTTGTTCGAGACGCTAACTTCTGCGCCAGAAGAGCACGGTTTC
GTGAACGCGAGCGATCCTTGTTTGGACATCAACCGCTCATCGTCTGTCGATTACATGTACACCCACGCAT
TGCGCTCTGAGTGTGCAGCGTCTGGTGCTGAGAAGTTTGTGTTCTGGGATGTCACGCATCCAACAACAGC
AACTCACCGCTATGTTGCAGAGAAAATGCTAGAAAGTAGCAACAACTTAGCCGAGTACCGTTTCTAA

>10-4251
ATGATGAAAAAAACAATCACACTATTAACTGCATTACTCCCGCTTGCTTCTGCAGTTGCCGAAGAGCCAA
CCTTATCACCAGAAATGGTTTCAGCGTCTGAAGTGATCAGCACGCAAGAAAACCAAACCTATACCTATGT
TCGCTGTTGGTATCGCACCAGCTACTCGAAAGATGATCCAGCGACCGATTGGGAATGGGCAAAAAACGAA
GATGGTAGCTACTTCACCATTGACGGCTACTGGTGGAGCTCCGTTTCATTTAAAAACATGTTCTACACCA
ACACGTCGCAAAACGTTATCCGTCAGCGTTGTGAAGCAACATTAGATTTGGCGAACGAGAACGCAGACAT
TACGTTCTTCGCCGCTGACAATCGCTTCTCATACAACCACACGATCTGGAGCAACGACGCAGCAATGCAG
CCAGATCAAATCAACAAAGTGGTTGCACTCGGTGACAGCTTGTCTGATACAGGCAACATCTTTAACGCAT
CACAATGGCGCTTCCCTAACCCGAACAGCTGGTTCTTAGGTCACTTCTCCAACGGTTTTGTTTGGACAGA
ATACATTGCCAAAGCGAAGAACCTTCCGCTCTACAACTGGGCAGTTGGCGGCGCGGCTGGTGAGAACCAA
TACATCGCGCTAACAGGGGTTGGTGAGCAAGTTTCTTCGTACTTAACCTACGCAAAACTGGCGAAGAACT
ACAAACCAGCAAACACCTTGTTTACGCTTGAGTTTGGTTTGAATGACTTCATGAACTACAACCGTGGCGT
TCCAGAAGTGAAAGCGGATTATGCAGAAGCACTGATTCGTTTGACGGACGCAGGTGCGAAGAACTTCATG
TTGATGACACTGCCAGATGCGACGAAAGCGCCTCAGTTTAAGTACTCAACACAAGAAGAGATCGACAAAA
TTCGTGCGAAAGTGCTTGAGATGAACGAGTTCATCAAGGCACAAGCGATGTACTACAAAGCGCAAGGTTA
CAACATCACGTTGTTTGATACTCACGCCTTGTTCGAGACGCTAACTTCTGCGCCAGAAGAGCACGGTTTC
GTGAACGCGAGCGATCCTTGTTTGGACATCAACCGCTCATCGTCTGTCGATTACATGTACACCCACGCAT
TGCGCTCTGAGTGTGCAGCGTCTGGTGCTGAGAAGTTTGTGTTCTGGGATGTCACGCATCCAACAACAGC
AACTCACCGCTATGTTGCAGAGAAAATGCTAGAAAGTAGCAACAACTTAGCCGAGTACCGTTTCTAA

>09-4663
ATGATGAAAAAAACAATCACACTATTAACTGCATTACTCCCGCTTGCTTCTGCAGTTGCCGAAGAGCCAACCTTAT
CACCAGAAATGGTTTCAGCGTCTGAAGTGATCAGCACGCAAGAAAACCAAACCTATACCTATGTTCGCTGTTGGTA
TCGCACCAGCTACTCGAAAGATGATCCGGCGACCGATTGGGAATGGGCAAAAAACGAAGATGGTAGCTACTTCAC
CATTGACGGCTACTGGTGGAGCTCCGTTTCATTTAAAAACATGTTCTACACCAACACGTCGCAAAACGTTATCCGT
CAGCGTTGTGAAGCCACATTAGATTTGGCGAACGAGAACGCAGACATTACGTTCTTCGCCGCTGACAATCGCTTCT
CATACAACCACACGATCTGGAGCAACGACGCAGCAATGCAGCCAGATCAAATCAACAAAGTGGTTGCACTCGGTG
ACAGCTTGTCTGATACAGGCAACATCTTTAACGCATCACAATGGCGCTTCCCTAACCCGAACAGCTGGTTCTTAGG
TCACTTCTCCAACGGTTTTGTGTGGACAGAATACATTGCCAAAGCGAAGAACCTTCCGCTCTACAACTGGGCAGTT
GGCGGCGCGGCTGGTGAGAACCAATACATCGCGCTAACAGGGGTTGGTGATCAAGTTTCTTCGTACTTAACCTACG
CAAAACTGGCGAAGAACTACAAACCAGCAAACACCTTGTTTACGCTTGAGTTTGGTTTGAATGACTTCATGAACTA
CAACCGTGGCGTTCCAGAAGTGAAAGCAGATTATGCAGAAGCACTGATTCGTTTGACGGACGCAGGTGCGAAGAA
CTTCATGTTGATGACACTGCCAGACGCGACGAAAGCGCCTCAGTTTAAGTACTCAACACAAGAAGAGATCGACAA
AATTCGTGCGAAAGTGCTTGAGATGAACGAGTTCATCAAGGCACAAGCGATGTACTACAAAGCGCAAGGTTACAA
CATCACGTTGTTTGATACTCACGCCTTGTTCGAGACGCTAACTTCTGCGCCCGAAAAGCACGGTTTCGTGAACGCG
AGTGATCCTTGTTTGGACATCAACCGCTCATCGTCTGTCGATTACATGTACACCCACGCATTGCGCTCTGAGTGTGC
GGCGTCTGGTGCTGAGAAGTTTGTGTTCTGGGATGTCACGCACCCAACAACAGCAACTCACCGCTATGTTGCAGAG
AAAATGCTAGAAAGTAGCAACAACTTAGCCGAGTACCGTTTCTAA

>10-4244
ATGATGAAAAAAACAATCACACTATTAACTGCATTACTCCCGCTTGCTTCTGCAGTTGCCGAAGAGCCAACCTTAT
CACCAGAAATGGTTTCAGCGTCTGAAGTGATCAGCACGCAAGAAAACCAAACCTATACCTATGTTCGCTGTTGGTA
TCGCACCAGCTACTCGAAAGATGATCCGGCGACCGATTGGGAATGGGCAAAAAACGAAGATGGTAGCTACTTCAC

CATTGACGGCTACTGGTGGAGCTCCGTTTCATTTAAAAACATGTTCTACACCAACACGTCGCAAAACGTTATCCGT
CAGCGTTGTGAAGCAACATTAGATTTGGCGAACGAGAACGCAGACATTACGTTCTTCGCCGCTGACAATCGCTTCT
CATACAACCACACGATCTGGAGCAACGACGCAGCAATGCAGCCAGATCAAATCAACAAAGTGGTTGCACTCGGTG
ACAGCTTGTCTGATACAGGCAACATCTTTAACGCATCACAATGGCGCTTCCCTAACCCGAACAGCTGGTTCTTAGG
TCACTTCTCCAACGGTTTTGTGTGGACAGAATACATTGCCAAAGCGAAGAACCTTCCGCTCTACAACTGGGCAGTT
GGCGGCGCGGCTGGTGAGAACCAATACATCGCGCTAACAGGGGTTGGTGATCAAGTTTCTTCGTACTTAACCTACG
CAAAACTGGCGAAGAACTACAAACCAGCAAACACCTTGTTTACGCTTGAGTTTGGTTTGAATGACTTCATGAACTA
CAACCGTGGCGTTCCAGAAGTGAAAGCGGATTATGCAGAAGCACTGATTCGTTTGACGGACGCAGGTGCGAAGAA
CTTCATGTTGATGACACTGCCAGACGCGACGAAAGCGCCTCAGTTTAAGTACTCAACACAAGAAGAGATCGACAA
AATTCGTGCGAAAGTGCTTGAGATGAACGAGTTCATCAAGGCACAAGCGATGTACTACAAAGCGCAAGGTTACAA
CATCACGTTGTTTGATACTCACGCCTTGTTCGAGACGCTAACTTCTGCGCCAGAAGAGCACGGTTTCGTGAACGCG
AGCGATCCTTGTTTGGACATCAACCGCTCATCGTCTGTCGATTACATGTACACCCACGCATTGCGCTCTGAGTGTGC
AGCGTCTGGTGCTGAGAAGTTTGTGTTCTGGGATGTCACGCATCCAACAACTGCAACTCACCGCTATGTTGCAGAG
AAAATGCTAGAAAGTAGCAACAACTTAGCCGAGTACCGTTTCTAA

>10-4287
ATGATGAAAAAAACAATCACACTATTAACTGCATTACTCCCGCTTGCTTCTGCAGTTGCCGAAGAGCCAA
CCTTATCACCAGAAATGGTTTCAGCGTCTGAAGTGATCAGCACGCAAGAAAACCAAACCTATACCTATGT
TCGCTGTTGGTATCGCACCAGCTACTCGAAAGATGATCCGGCGACCGATTGGGAATGGGCAAAAAACGAA
GATGGTAGCTACTTCACCATTGACGGCTACTGGTGGAGCTCCGTTTCATTTAAAAACATGTTCTACACCA
ACACGTCGCAAAACGTTATCCGTCAGCGTTGTGAAGCAACATTAGATTTGGCGAACGAGAACGCAGACAT
TACGTTCTTCGCCGCTGACAATCGCTTCTCATACAACCACACGATCTGGAGCAACGACGCAGCAATGCAG
CCAGATCAAATCAACAAAGTGGTTGCACTCGGTGACAGCTTGTCTGATACAGGCAACATATTTAACGCAT
CACAATGGCGCTTCCCTAACCCGAACAGCTGGTTCTTAGGTCACTTCTCCAACGGTTTTGTGTGGACAGA
ATACATTGCCAAAGCGAAGAACCTTCCGCTCTACAACTGGGCAGTTGGCGGCGCGGCTGGTGAGAACCAA
TACATCGCGCTAACAGGGGTTGGTGATCAAGTTTCTTCGTACTTAACCTACGCAAAACTGGCGAAGAACT
ACAAACCAGCAAACACCTTGTTTACGCTTGAGTTTGGTTTGAATGACTTCATGAACTACAACCGTGGCGT
TCCAGAAGTGAAAGCGGATTATGCAGAAGCACTGATTCGTTTGACGGACGCAGGTGCGAAGAACTTCATG
TTGATGACACTGCCAGATGCGACGAAAGCGCCTCAGTTTAAGTACTCAACACAAGAAGAGATCGACAAAA
TTCGTGCGAAAGTGCTTGAGATGAACGAGTTCATCAAGGCACAAGCGATGTACTACAAAGCGCAAGGTTA
CAACATCACGTTGTTTGATACTCACGCCTTGTTCGAGACGCTAACTTCTGCGCCAGAAGAGCACGGTTTC
GTGAACGCGAGTGATCCTTGTTTGGACATCAACCGCTCATCGTCTGTCGATTACATGTACACCCACGCAT
TGCGCTCTGAGTGTGCAGCATCTGGTGCTGAGAAGTTTGTGTTCTGGGATGTCACGCATCCAACAACAGC
AACTCACCGCTATGTTGCAGAGAAAATGCTAGAAAGTAGCAACAACTTAGCAGAGTACCGTTTCTAA

>10-4255
ATGATGAAAAAAACAATCACACTATTAACTGCATTACTCCCGCTTGCTTCTGCAGTTGCCGAAGAGCCAACCTTAT
CACCAGAAATGGTTTCAGCGTCTGAAGTGATCAGCACGCAAGAAAACCAAACCTATACCTATGTTCGCTGTTGGTA
TCGCACCAGCTACTCGAAAGATGATCCGGCGACCGATTGGGAATGGGCAAAAAACGAAGATGGTAGCTACTTCAC
CATTGACGGCTACTGGTGGAGCTCCGTTTCATTTAAAAACATGTTCTACACCAACACGTCGCAAAACGTTATCCGT
CAGCGTTGTGAAGCAACATTAGATTTGGCGAACGAGAACGCAGACATTACGTTCTTCGCCGCTGACAATCGCTTCT
CATACAACCACACGATCTGGAGCAACGACGCAGCAATGCAGCCAGATCAAATCAACAAAGTGGTTGCACTCGGTG
ACAGCTTGTCTGATACAGGCAACATCTTTAACGCATCACAATGGCGCTTCCCTAACCCGAACAGCTGGTTCTTAGG
TCACTTCTCCAACGGTTTTGTGTGGACAGAATACATTGCCAAAGCGAAGAACCTTCCGCTCTACAACTGGGCAGTT
GGCGGTGCGGCTGGTGAGAACCAATACATCGCGCTAACAGGGGTTGGTGATCAAGTTTCTTCGTACTTAACCTACG
CAAAACTGGCGAAGAACTACAAACCAGCAAACACCTTGTTTACGCTTGAGTTTGGTTTGAATGACTTCATGAACTA
CAACCGTGGCGTTCCAGAAGTGAAAGCGGATTATGCAGAAGCACTGATTCGTTTGACGGACGCAGGTGCGAAGAA
CTTCATGTTGATGACACTGCCAGATGCGACGAAAGCGCCTCAGTTTAAGTACTCAACACAAGAAGAGATCGACAA
AATTCGTGCGAAAGTGCTTGAGATGAACGAGTTCATCAAGGCACAAGCGATGTACTACAAAGCGCAAGGTTACAA

CATCACGTTGTTTGATACTCACGCCTTGTTCGAGACGCTAACTTCTGCGCCAGAAGAGCACGGTTTCGTGAACGCG
AGCGATCCTTGTTTGGACATCAACCGCTCATCGTCTGTCGATTACATGTACACCCACGCATTGCGCTCTGAGTGTGC
AGCGTCTGGTGCTGAGAAGTTTGTGTTCTGGGATGTCACGCACCCAACAACAGCAACTCACCGCTATGTTGCAGAG
AAAATGCTAGAAAGTAGCAACAACTTAGAAGAGTTTCGCTTTTAA

>Peru-466
ATGATGAAAAAAACAATCACACTATTAACTGCATTACTCCCGCTTGCTTCTGCAGTTGCCGAAGAGCCAA
CCTTATCACCAGAAATGGTTTCAGCGTCTGAAGTGATCAGCACGCAAGAAAACCAAACCTATACCTATGT
TCGCTGTTGGTATCGCACCAGCTACTCGAAAGATGATCCAGCGACCGATTGGGAATGGGCAAAAAACGAA
GATGGTAGCTACTTCACCATTGACGGCTACTGGTGGAGCTCCGTTTCATTTAAAAACATGTTCTACACCA
ACACGTCGCAAAACGTTATCCGTCAGCGTTGTGAAGCAACATTAGATTTGGCGAACGAGAACGCAGACAT
TACGTTCTTCGCCGCTGACAATCGCTTCTCATACAACCACACGATCTGGAGCAACGACGCAGCAATGCAG
CCAGATCAAATCAACAAAGTGGTTGCACTCGGTGACAGCTTGTCTGATACAGGCAACATCTTTAACGCAT
CACAATGGCGCTTCCCTAACCCGAACAGCTGGTTCTTAGGTCACTTCTCCAACGGTTTTGTTTGGACAGA
ATACATTGCCAAAGCGAAGAACCTTCCGCTCTACAACTGGGCAGTTGGCGGCGCGGCTGGTGAGAACCAA
TACATCGCGCTAACAGGGGTTGGTGAGCAAGTTTCTTCGTACTTAACCTACGCAAAACTGGCGAAGAACT
ACAAACCAGCAAACACCTTGTTTACGCTTGAGTTTGGTTTGAATGACTTCATGAACTACAACCGTGGCGT
TCCAGAAGTGAAAGCGGATTATGCAGAAGCACTGATTCGTTTGACGGACGCAGGTGCGAAGAACTTCATG
TTGATGACACTGCCAGATGCGACGAAAGCGCCTCAGTTTAAGTACTCAACACAAGAAGAGATCGACAAAA
TTCGTGCGAAAGTGCTTGAGATGAACGAGTTCATCAAGGCACAAGCGATGTACTACAAAGCGCAAGGTTA
CAACATCACGTTGTTTGATACTCACGCCTTGTTCGAGACGCTAACTTCTGCGCCAGAAGAGCACGGTTTC
GTGAACGCGAGCGATCCTTGTTTGGACATCAACCGCTCATCGTCTGTCGATTACATGTACACCCACGCAT
TGCGCTCTGAGTGTGCAGCGTCTGGTGCTGAGAAGTTTGTGTTCTGGGATGTCACGCATCCAACAACAGC
AACTCACCGCTATGTTGCAGAGAAAATGCTAGAAAGTAGCAACAACTTAGCCGAGTACCGTTTCTAA

>AQ4037
ATGATGAAAAAAACAATCACACTATTAACTGCATTACTCCCGCTTGCTTCTGCAGTTGCCGAAGAGCCAACCTTAT
CACCAGAAATGGTTTCAGCGTCTGAAGTGATCAGCACGCAAGAAAACCAAACCTATACCTATGTTCGCTGTTGGTA
TCGCACCAGCTACTCGAAAGATGATCCAGCGACCGATTGGGAATGGGCAAAAAACGAAGATGGTAGCTACTTCAC
CATTGACGGCTACTGGTGGAGCTCCGTTTCATTTAAAAACATGTTCTACACCAACACGTCGCAAAACGTTATCCGT
CAGCGTTGTGAAGCAACATTAGATTTGGCGAACGAGAACGCAGACATTACGTTCTTCGCCGCTGACAATCGCTTCT
CATACAACCACACGATCTGGAGCAACGACGCAGCAATGCAGCCAGATCAAATCAACAAAGTGGTTGCACTCGGTG
ACAGCTTGTCTGATACAGGCAACATCTTTAACGCATCACAATGGCGCTTCCCTAACCCGAACAGCTGGTTCTTAGG
TCACTTCTCCAACGGTTTTGTTTGGACAGAATACATTGCCAAAGCGAAGAACCTTCCGCTCTACAACTGGGCAGTT
GGCGGCGCGGCTGGTGAGAACCAATACATCGCGCTAACAGGGGTTGGTGAGCAAGTTTCTTCGTACTTAACCTACG
CAAAACTGGCAAAGAACTACAAACCAGCAAACACCTTGTTTACGCTTGAGTTTGGTTTGAATGACTTCATGAACTA
CAACCGTGGCGTTCCAGAAGTGAAAGCGGATTATGCAGAAGCACTGATTCGTTTGACGGACGCAGGTGCGAAGAA
CTTCATGTTGATGACACTGCCAGATGCGACGAAAGCGCCTCAGTTTAAGTACTCAACACAAGAAGAGATCGACAA
AATTCGTGCGAAAGTGCTTGAGATGAACGAGTTCATCAAGGCACAAGCGATGTACTACAAAGCGCAAGGTTACAA
CATCACGTTGTTTGATACTCACGCCTTGTTCGAGACGCTAACTTCTGCGCCAGAAGAGCACGGTTTCGTGAACGCG
AGCGATCCTTGTTTGGACATCAACCGCTCATCGTCTGTCGATTACATGTACACCCACGCATTGCGCTCTGAGTGTGC
AGCGTCTGGTGCTGAGAAGTTTGTGTTCTGGGATGTCACGCATCCAACAACAGCAACTCACCGCTATGTTGCAGAG
AAAATGCTAGAAAGTAGCAACAACTTAGCCGAGTACCGTTTCTAA

>AN-5034
ATGATGAAAAAAACAATCACACTATTAACTGCATTACTCCCGCTTGCTTCTGCAGTTGCCGAAGAGCCAACCTTAT
CACCAGAAATGGTTTCAGCGTCTGAAGTGATCAGCACGCAAGAAAACCAAACCTATACCTATGTTCGCTGTTGGTA
TCGCACCAGCTACTCGAAAGATGATCCAGCGACCGATTGGGAATGGGCAAAAAACGAAGATGGTAGCTACTTCAC
CATTGACGGCTACTGGTGGAGCTCCGTTTCATTTAAAAACATGTTCTACACCAACACGTCGCAAAACGTTATCCGT
CAGCGTTGTGAAGCAACATTAGATTTGGCGAACGAGAACGCAGACATTACGTTCTTCGCCGCTGACAATCGCTTCT
CATACAACCACACGATCTGGAGCAACGACGCAGCAATGCAGCCAGATCAAATCAACAAAGTGGTTGCACTCGGTG

ACAGCTTGTCTGATACAGGCAACATCTTTAACGCATCACAATGGCGCTTCCCTAACCCGAACAGCTGGTTCTTAGG
TCACTTCTCCAACGGTTTTGTTTGGACAGAATACATTGCCAAAGCGAAGAACCTTCCGCTCTACAACTGGGCAGTT
GGCGGCGCGGCTGGTGAGAACCAATACATCGCGCTAACAGGGGTTGGTGAGCAAGTTTCTTCGTACTTAACCTACG
CAAAACTGGCGAAGAACTACAAACCAGCAAACACCTTGTTTACGCTTGAGTTTGGTTTGAATGACTTCATGAACTA
CAACCGTGGCGTTCCAGAAGTGAAAGCGGATTATGCAGAAGCACTGATTCGTTTGACGGACGCAGGTGCGAAGAA
CTTCATGTTGATGACACTGCCAGATGCGACGAAAGCGCCTCAGTTTAAGTACTCAACACAAGAAGAGATCGACAA
AATTCGTGCGAAAGTGCTTGAGATGAACGAGTTCATCAAGGCACAAGCGATGTACTACAAAGCGCAAGGTTACAA
CATCACGTTGTTTGATACTCACGCCTTGTTCGAGACGCTAACTTCTGCGCCAGAAGAGCACGGTTTCGTGAACGCG
AGCGATCCTTGTTTGGACATCAACCGCTCATCGTCTGTCGATTACATGTACACCCACGCATTGCGCTCTGAGTGTGC
AGCGTCTGGTGCTGAGAAGTTTGTGTTCTGGGATGTCACGCATCCAACAACAGCAACTCACCGCTATGTTGCAGAG
AAAATGCTAGAAAGTAGCAACAACTTAGCCGAGTACCGTTTCTAA

>K5030
ATGATGAAAAAAACAATCACACTATTAACTGCATTACTCCCGCTTGCTTCTGCAGTTGCCGAAGAGCCAACCTTAT
CACCAGAAATGGTTTCAGCGTCTGAAGTGATCAGTACGCAAGAAAACCAAACCTATACCTATGTTCGCTGTTGGTA
TCGCACCAGCTACTCGAAAGATGATCCAGCGACCGATTGGGAATGGGCAAAAAACGAAGATGGTAGCTACTTCAC
CATTGACGGCTACTGGTGGAGCTCCGTTTCATTTAAAAACATGTTCTACACCAACACGTCGCAAAACGTTATCCGT
CAGCGTTGTGAAGCAACATTAGATTTGGCGAACGAGAACGCAGACATTACGTTCTTCGCCGCTGACAATCGCTTCT
CATACAACCACACGATCTGGAGCAACGACGCAGCAATGCAGCCAGATCAAATCAACAAAGTGGTTGCACTCGGTG
ACAGCTTGTCTGATACAGGCAACATCTTTAACGCATCACAATGGCGCTTCCCTAACCCGAACAGCTGGTTCTTAGG
TCACTTCTCCAACGGTTTTGTTTGGACAGAATACATTGCCAAAGCGAAGAACCTTCCGCTCTACAACTGGGCAGTT
GGCGGCGCGGCTGGTGAGAACCAATACATCGCGCTAACAGGGGTTGGTGAGCAAGTTTCTTCGTACTTAACCTACG
CAAAACTGGCGAAGAACTACAAACCAGCAAACACCTTGTTTACGCTTGAGTTTGGTTTGAATGACTTCATGAACTA
CAACCGTGGCGTTCCAGAAGTGAAAGCGGATTATGCAGAAGCACTGATTCGTTTGACGGACGCAGGTGCGAAGAA
CTTCATGTTGATGACACTGCCAGATGCGACGAAAGCGCCTCAGTTTAAGTACTCAACACAAGAAGAGATCGACAA
AATTCGTGCGAAAGTGCTTGAGATGAACGAGTTCATCAAGGCACAAGCGATGTACTACAAAGCGCAAGGTTACAA
CATCACGTTGTTTGATACTCACGCCTTGTTCGAGACGCTAACTTCTGCGCCAGAAGAGCACGGTTTCGTGAACGCG
AGCGATCCTTGTTTGGACATCAACCGCTCATCGTCTGTCGATTACATGTACACCCACGCATTGCGCTCTGAGTGTGC
AGCGTCTGGTGCTGAGAAGTTTGTGTTCTGGGATGTCACGCATCCAACAACAGCAACTCACCGCTATGTTGCAGAG
AAAATGCTAGAAAGTAGCAACAACTTAGCCGAGTACCGTTTCTAA

>10329
ATGATGAAAAAAACAATCACACTATTAACTGCATTACTCCCGCTTGCTTCTGCAGTTGCCGAAGAGCCAACCTTAT
CACCAGAAATGGTTTCAGCGTCTGAAGTGATCAGCACGCAAGAAAACCAAACCTATACCTATGTTCGCTGTTGGTA
TCGCACCAGCTACTCGAAAGATGATCCGGCGACCGATTGGGAATGGGCAAAAAACGAAGATGGTAGCTACTTCAC
CATTGACGGCTACTGGTGGAGCTCCGTTTCATTTAAAAACATGTTCTACACCAACACGTCGCAAAACGTTATCCGT
CAGCGTTGTGAAGCAACATTAGATTTGGCGAACGAGAACGCAGACATTACGTTCTTCGCCGCTGACAATCGCTTCT
CATACAACCACACGATCTGGAGCAACGACGCAGCAATGCAGCCAGATCAAATCAACAAAGTGGTTGCACTCGGTG
ACAGCTTGTCTGATACAGGCAACATCTTTAACGCATCACAATGGCGCTTCCCTAACCCGAACAGCTGGTTCTTAGG
TCACTTCTCCAACGGTTTTGTGTGGACAGAATACATTGCCAAAGCGAAGAACCTTCCGCTCTACAACTGGGCAGTT
GGCGGCGCGGCTGGTGAGAACCAATACATCGCGCTAACAGGGGTTGGTGATCAAGTTTCTTCGTACTTAACCTACG
CAAAACTGGCGAAGAACTACAAACCAGCAAACACCTTGTTTACGCTTGAGTTTGGTTTGAATGACTTCATGAACTA
CAACCGTGGCGTTCCAGAAGTGAAAGCGGATTATGCAGAAGCACTGATTCGTTTGACGGACGCAGGTGCGAAGAA
CTTCATGTTGATGACACTGCCAGACGCGACGAAAGCGCCTCAGTTTAAGTACTCAACACAAGAAGAGATCGACAA
AATTCGTGCGAAAGTGCTTGAGATGAACGAGTTCATCAAGGCACAAGCGATGTACTACAAAGCGCAAGGTTACAA
CATCACGTTGTTTGATACTCACGCCTTGTTCGAGACGCTAACTTCTGCGCCAGAAGAGCACGGTTTCGTGAACGCG
AGCGATCCTTGTTTGGACATCAACCGCTCATCGTCTGTCGATTACATGTACACCCACGCATTGCGCTCTGAGTGTGC
AGCGTCTGGTGCTGAGAAGTTTGTGTTCTGGGATGTCACGCATCCAACAACTGCAACTCACCGCTATGTTGCAGAG
AAAATGCTAGAAAGTAGCAACAACTTAGCCGAGTACCGTTTCTAA

>SNUVpS-1

ATGATGAAAAAAACAATCACACTATTAACTGCATTACTCCCGCTTGCTTCTGCAGTTGCCGAAGAGCCAA
CCTTATCACCAGAAATGGTTTCAGCGTCTGAAGTGATCAGCACGCAAGAAAACCAAACCTATACCTATGT
TCGCTGTTGGTATCGCACCAGCTACTCGAAAGATGATCCGGCGACCGATTGGGAATGGGCAAAAAACGAA
GATGGTAGCTACTTCACCATTGACGGCTACTGGTGGAGCTCCGTTTCATTTAAAAACATGTTCTACACCA
ACACGTCGCAAAACGTTATCCGTCAGCGTTGTGAAGCAACATTAGATTTGGCGAACGAGAACGCAGACAT
TACGTTCTTCGCCGCTGACAATCGCTTCTCATACAACCACACGATCTGGAGCAACGACGCAGCAATGCAG
CCAGATCAAATCAACAAAGTGGTTGCACTCGGTGACAGCTTGTCTGATACAGGCAACATCTTTAACGCAT
CACAATGGCGCTTCCCTAACCCGAACAGCTGGTTCTTAGGTCACTTCTCCAACGGTTTTGTGTGGACAGA
ATACATTGCCAAAGCGAAGAACCTTCCGCTCTACAACTGGGCAGTTGGCGGCGCGGCTGGTGAGAACCAA
TACATCGCGCTAACAGGGGTTGGTGATCAAGTTTCTTCGTACTTAACCTACGCAAAACTGGCGAAGAACT
ACAAACCAGCAAACACCTTGTTTACGCTTGAGTTTGGTTTGAATGACTTCATGAACTACAACCGTGGCGT
TCCAGAAGTGAAAGCGGATTATGCAGAAGCACTGATTCGTTTGACGGACGCAGGTGCGAAGAACTTCATG
TTGATGACACTGCCAGACGCGACGAAAGCGCCTCAGTTTAAGTACTCAACACAAGAAGAGATCGACAAAA
TTCGTGCGAAAGTGCTTGAGATGAACGAGTTCATCAAGGCACAAGCGATGTACTACAAAGCGCAAGGTTA
CAACATCACGTTGTTTGATACTCACGCCTTGTTCGAGACGCTAACTTCTGCGCCAGAAGAGCACGGTTTC
GTGAACGCGAGCGATCCTTGTTTGGACATCAACCGCTCATCGTCTGTCGATTACATGTACACCCACGCAT
TGCGCTCTGAGTGTGCAGCGTCTGGTGCTGAGAAGTTTGTGTTCTGGGATGTCACGCATCCAACAACAGC
AACTCACCGCTATGTTGCAGAGAAAATGCTAGAAAGTAGCAACAACTTAGCCGAGTACCGTTTCTAA

>PCV08-7
ATGATGAAAAAAACAATCACACTATTAACTGCATTACTCCCGCTTGCTTCTGCAGTTGCCGAAGAGCCAACCTTAT
CACCAGAAATGGTTTCAGCGTCTGAAGTGATCAGCACGCAAGAAAACCAAACCTATACCTATGTTCGCTGTTGGTA
TCGCACCAGCTACTCGAAAGATGATCCGGCGACCGATTGGGAATGGGCAAAAAACGAAGATGGTAGCTACTTCAC
CATTGACGGCTACTGGTGGAGCTCCGTTTCACTTAAAAACATGTTCTACACCAACACGTCGCAAAACGTTATCCGT
CAGCGTTGTGAAGCAACATTAGATTTGGCGAACGAGAACGCAGACATTACGTTCTTCGCCGCTGACAATCGCTTCT
CATACAACCACACGATCTGGAGCAACGACGCAGCAATGCAGCCAGATCAAATCAACAAAGTGGTTGCACTCGGTG
ACAGCTTGTCTGATACAGGCAACATCTTTAACGCATCACAATGGCGCTTCCCTAACCCGAACAGCTGGTTCTTAGG
TCACTTCTCCAACGGTTTTGTGTGGACAGAATACATTGCCAAAGCGAAGAACCTTCCGCTCTACAACTGGGCAGTT
GGCGGCGCGGCTGGTGAGAACCAATACATCGCGCTAACAGGGGTTGGTGATCAAGTTTCTTCGTACTTAACCTACG
CAAAACTGGCGAAGAACTACAAACCAGCAAACACCTTGTTTACGCTTGAGTTTGGTTTGAATGACTTCATGAACTA
CAACCGTGGCGTTCCAGAAGTGAAAGCAGATTATGCAGAAGCACTGATTCGTTTGACGGACGCAGGTGCGAAGAA
CTTCATGTTGATGACACTGCCAGACGCGACGAAAGCGCCTCAGTTTAAGTACTCAACACAAGAAGAGATCGACAA
AATTCGTGCGAAAGTGCTTGAGATGAACGAGTTCATCAAGGCACAAGCGATGTACTACAAAGCGCAAGGTTACAA
CATCACGTTGTTTGATACTCACGCCTTGTTCGAGACGCTAACTTCTGCGCCAGAAGAGCACGGTTTCGTGAACGCG
AGCGATCCTTGTTTGGACATCAACCGCTCATCGTCTGTCGATTACATGTACACCCACGCATTGCGCTCTGAGTGTGC
AGCGTCTGGTGCTGAGAAGTTTGTGTTCTGGGATGTCACGCATCCAACAACAGCAACTCACCGCTATGTTGCAGAG
AAAATGCTAGAAAGTAGCAACAACTTAGCCGAGTACCGTTTCTAA

>VP-NY4
ATGATGAAAAAAACAATCACACTATTAACTGCATTACTCCCGCTTGCTTCTGCAGTTGCCGAAGAGCCAACCTTAT
CACCAGAAATGGTTTCAGCGTCTGAAGTGATCAGCACGCAAGAAAACCAAACCTATACCTATGTTCGCTGTTGGTA
TCGCACCAGCTACTCGAAAGATGATCCAGCGACCGATTGGGAATGGGCAAAAAACGAAGATGGTAGCTACTTCAC
CATTGACGGCTACTGGTGGAGCTCCGTTTCATTTAAAAACATGTTCTACACCAACACGTCGCAAAACGTTATCCGT
CAGCGTTGTGAAGCAACATTAGATTTGGCGAACGAGAACGCAGACATTACGTTCTTCGCCGCTGACAATCGCTTCT
CATACAACCACACGATCTGGAGCAACGACGCAGCAATGCAGCCAGATCAAATCAACAAAGTGGTTGCACTCGGTG
ACAGCTTGTCTGATACAGGCAACATCTTTAACGCATCACAATGGCGCTTCCCTAACCCGAACAGCTGGTTCTTAGG
TCACTTCTCCAACGGTTTTGTTTGGACAGAATACATTGCCAAAGCGAAGAACCTTCCGCTCTACAACTGGGCAGTT
GGCGGCGCGGCTGGTGAGAACCAATACATCGCGCTAACAGGGGTTGGTGAGCAAGTTTCTTCGTACTTAACCTACG
CAAAACTGGCGAAGAACTACAAACCAGCAAACACCTTGTTTACGCTTGAGTTTGGTTTGAATGACTTCATGAACTA
CAACCGTGGCGTTCCAGAAGTGAAAGCGGATTATGCAGAAGCACTGATTCGTTTGACGGACGCAGGTGCGAAGAA
CTTCATGTTGATGACACTGCCAGATGCGACGAAAGCGCCTCAGTTTAAGTACTCAACACAAGAAGAGATCGACAA

AATTCGTGCGAAAGTGCTTGAGATGAACGAGTTCATCAAGGCACAAGCGATGTACTACAAAGCGCAAGGTTACAA
CATCACGTTGTTTGATACTCACGCCTTGTTCGAGACGCTAACTTCTGCGCCAGAAGAGCACGGTTTCGTGAACGCG
AGCGATCCTTGTTTGGACATCAACCGCTCATCGTCTGTCGATTACATGTACACCCACGCATTGCGCTCTGAGTGTGC
AGCGTCTGGTGCTGAGAAGTTTGTGTTCTGGGATGTCACGCATCCAACAACAGCAACTCACCGCTATGTTGCAGAG
AAAATGCTAGAAAGTAGCAACAACTTAGCCGAGTACCGTTTCTAA

>VP2007-095
ATGATGAAAAAAACAATCACACTATTAACTGCATTACTCCCGCTTGCTTCTGCAGTTGCCGAAGAGCCAA
CCTTATCACCAGAAATGGTTTCAGCGTCTGAAGTGATCAGCACGCAAGAAAACCAAACCTATACCTATGT
TCGCTGTTGGTATCGCACCAGCTACTCGAAAGATGATCCGGCGACCGATTGGGAATGGGCAAAAAACGAA
GATGGTAGCTACTTCACCATTGACGGCTACTGGTGGAGCTCCGTTTCATTTAAAAACATGTTCTACACCA
ACACGTCGCAAAACGTTATCCGTCAGCGTTGTGAAGCAACATTAGATTTGGCGAACGAGAACGCAGACAT
TACGTTCTTCGCCGCTGACAATCGCTTCTCATACAACCACACGATCTGGAGCAACGACGCAGCAATGCAG
CCAGATCAAATCAACAAAGTGGTTGCACTCGGTGACAGCTTGTCTGATACAGGCAACATCTTTAACGCAT
CACAATGGCGCTTCCCTAACCCGAACAGCTGGTTCTTAGGTCACTTCTCCAACGGTTTTGTGTGGACAGA
ATACATTGCCAAAGCGAAGAACCTTCCGCTCTACAACTGGGCAGTTGGCGGCGCGGCTGGTGAGAACCAA
TACATCGCGCTAACAGGGGTTGGTGATCAAGTTTCTTCGTACTTAACCTACGCAAAACTGGCGAAGAACT
ACAAACCAGCAAACACCTTGTTTACGCTTGAGTTTGGTTTGAATGACTTCATGAACTACAACCGTGGCGT
TCCAGAAGTGAAAGCGGATTATGCAGAAGCACTGATTCGTTTGACGGACGCAGGTGCGAAGAACTTCATG
TTGATGACACTGCCAGACGCGACGAAAGCGCCTCAGTTTAAGTACTCAACACAAGAAGAGATCGACAAAA
TTCGTGCGAAAGTGCTTGAGATGAACGAGTTCATCAAGGCACAAGCGATGTACTACAAAGCGCAAGGTTA
CAACATCACGTTGTTTGATACTCACGCCTTGTTCGAGACGCTAACTTCTGCGCCAGAAGAGCACGGTTTC
GTGAACGCGAGCGATCCTTGTTTGGACATCAACCGCTCATCGTCTGTCGATTACATGTACACCCACGCAT
TGCGCTCTGAGTGTGCAGCGTCTGGTGCTGAGAAGTTTGTGTTCTGGGATGTCACGCATCCAACAACAGC
AACTCACCGCTATGTTGCAGAGAAAATGCTAGAAAGTAGCAACAACTTAGCAGAGTACCGTTTCTAA

>VP232
ATGATGAAAAAAACAATCACACTATTAACTGCATTACTCCCGCTTGCTTCTGCAGTTGCCGAAGAGCCAA
CCTTATCACCAGAAATGGTTTCAGCGTCTGAAGTGATCAGCACGCAAGAAAACCAAACCTATACCTATGT
TCGCTGTTGGTATCGCACCAGCTACTCGAAAGATGATCCAGCGACCGATTGGGAATGGGCAAAAAACGAA
GATGGTAGCTACTTCACCATTGACGGCTACTGGTGGAGCTCCGTTTCATTTAAAAACATGTTCTACACCA
ACACGTCGCAAAACGTTATCCGTCAGCGTTGTGAAGCAACATTAGATTTGGCGAACGAGAACGCAGACAT
TACGTTCTTCGCCGCTGACAATCGCTTCTCATACAACCACACGATCTGGAGCAACGACGCAGCAATGCAG
CCAGATCAAATCAACAAAGTGGTTGCACTCGGTGACAGCTTGTCTGATACAGGCAACATCTTTAACGCAT
CACAATGGCGCTTCCCTAACCCGAACAGCTGGTTCTTAGGTCACTTCTCCAACGGTTTTGTTTGGACAGA
ATACATTGCCAAAGCGAAGAACCTTCCGCTCTACAACTGGGCAGTTGGCGGCGCGGCTGGTGAGAACCAA
TACATCGCGCTAACAGGGGTTGGTGAGCAAGTTTCTTCGTACTTAACCTACGCAAAACTGGCGAAGAACT
ACAAACCAGCAAACACCTTGTTTACGCTTGAGTTTGGTTTGAATGACTTCATGAACTACAACCGTGGCGT
TCCAGAAGTGAAAGCGGATTATGCAGAAGCACTGATTCGTTTGACGGACGCAGGTGCGAAGAACTTCATG
TTGATGACACTGCCAGATGCGACGAAAGCGCCTCAGTTTAAGTACTCAACACAAGAAGAGATCGACAAAA
TTCGTGCGAAAGTGCTTGAGATGAACGAGTTCATCAAGGCACAAGCGATGTACTACAAAGCGCAAGGTTA
CAACATCACGTTGTTTGATACTCACGCCTTGTTCGAGACGCTAACTTCTGCGCCAGAAGAGCACGGTTTC
GTGAACGCGAGCGATCCTTGTTTGGACATCAACCGCTCATCGTCTGTCGATTACATGTACACCCACGCAT
TGCGCTCTGAGTGTGCAGCGTCTGGTGCTGAGAAGTTTGTGTTCTGGGATGTCACGCATCCAACAACAGC
AACTCACCGCTATGTTGCAGAGAAAATGCTAGAAAGTAGCAACAACTTAGCCGAGTACCGTTTCTAA

>10290
ATGATGAAAAAAACAATCACACTATTAACTGCATTACTCCCGCTTGCTTCTGCAGTTGCCGAAGAGCCAA
CCTTATCACCAGAAATGGTTTCAGCGTCTGAAGTGATCAGCACGCAAGAAAACCAAACCTATACCTATGT
TCGCTGTTGGTATCGCACCAGCTACTCGAAAGATGATCCGGCGACCGATTGGGAATGGGCAAAAAACGAA
GATGGTAGCTACTTCACCATTGACGGCTACTGGTGGAGCTCCGTTTCACTTAAAAACATGTTCTACACCA

ACACGTCGCAAAACGTTATCCGTCAGCGTTGTGAAGCAACATTAGATTTGGCGAACGAGAACGCAGACAT
TACGTTCTTCGCCGCTGACAATCGCTTCTCATACAACCACACGATCTGGAGCAACGACGCAGCAATGCAG
CCAGATCAAATCAACAAAGTGGTTGCACTCGGTGACAGCTTGTCTGATACAGGCAACATCTTTAACGCAT
CACAATGGCGCTTCCCTAACCCGAACAGCTGGTTCTTAGGTCACTTCTCCAACGGTTTTGTGTGGACAGA
ATACATTGCCAAAGCGAAGAACCTTCCGCTCTACAACTGGGCAGTTGGCGGCGCGGCTGGTGAGAACCAA
TACATCGCGCTAACAGGGGTTGGTGATCAAGTTTCTTCGTACTTAACCTACGCAAAACTGGCGAAGAACT
ACAAACCAGCAAACACCTTGTTTACGCTTGAGTTTGGTTTGAATGACTTCATGAACTACAACCGTGGCGT
TCCAGAAGTGAAAGCAGATTATGCAGAAGCACTGATTCGTTTGACGGACGCAGGTGCGAAGAACTTCATG
TTGATGACACTGCCAGACGCGACGAAAGCGCCTCAGTTTAAGTACTCAACACAAGAAGAGATCGACAAAA
TTCGTGCGAAAGTGCTTGAGATGAACGAGTTCATCAAGGCACAAGCGATGTACTACAAAGCGCAAGGTTA
CAACATCACGTTGTTTGATACTCACGCCTTGTTCGAGACGCTAACTTCTGCGCCAGAAGAGCACGGTTTC
GTGAACGCGAGCGATCCTTGTTTGGACATCAACCGCTCATCGTCTGTCGATTACATGTACACCCACGCAT
TGCGCTCTGAGTGTGCAGCGTCTGGTGCTGAGAAGTTTGTGTTCTGGGATGTCACGCATCCAACAACAGC
AACTCACCGCTATGTTGCAGAGAAAATGCTAGAAAGTAGCAACAACTTAGCCGAGTACCGTTTCTAA

>VP250
ATGATGAAAAAAACAATCACACTATTAACTGCATTACTCCCGCTTGCTTCTGCAGTTGCCGAAGAGCCAA
CCTTATCACCAGAAATGGTTTCAGCGTCTGAAGTGATCAGCACGCAAGAAAACCAAACCTATACCTATGT
TCGCTGTTGGTATCGCACCAGCTACTCGAAAGATGATCCAGCGACCGATTGGGAATGGGCAAAAAACGAA
GATGGTAGCTACTTCACCATTGACGGCTACTGGTGGAGCTCCGTTTCATTTAAAAACATGTTCTACACCA
ACACGTCGCAAAACGTTATCCGTCAGCGTTGTGAAGCAACATTAGATTTGGCGAACGAGAACGCAGACAT
TACGTTCTTCGCCGCTGACAATCGCTTCTCATACAACCACACGATCTGGAGCAACGACGCAGCAATGCAG
CCAGATCAAATCAACAAAGTGGTTGCACTCGGTGACAGCTTGTCTGATACAGGCAACATCTTTAACGCAT
CACAATGGCGCTTCCCTAACCCGAACAGCTGGTTCTTAGGTCACTTCTCCAACGGTTTTGTTTGGACAGA
ATACATTGCCAAAGCGAAGAACCTTCCGCTCTACAACTGGGCAGTTGGCGGCGCGGCTGGTGAGAACCAA
TACATCGCGCTAACAGGGGTTGGTGAGCAAGTTTCTTCGTACTTAACCTACGCAAAACTGGCGAAGAACT
ACAAACCAGCAAACACCTTGTTTACGCTTGAGTTTGGTTTGAATGACTTCATGAACTACAACCGTGGCGT
TCCAGAAGTGAAAGCGGATTATGCAGAAGCACTGATTCGTTTGACGGACGCAGGTGCGAAGAACTTCATG
TTGATGACACTGCCAGATGCGACGAAAGCGCCTCAGTTTAAGTACTCAACACAAGAAGAGATCGACAAAA
TTCGTGCGAAAGTGCTTGAGATGAACGAGTTCATCAAGGCACAAGCGATGTACTACAAAGCGCAAGGTTA
CAACATCACGTTGTTTGATACTCACGCCTTGTTCGAGACGCTAACTTCTGCGCCAGAAGAGCACGGTTTC
GTGAACGCGAGCGATCCTTGTTTGGACATCAACCGCTCATCGTCTGTCGATTACATGTACACCCACGCAT
TGCGCTCTGAGTGTGCAGCGTCTGGTGCTGAGAAGTTTGTGTTCTGGGATGTCACGCATCCAACAACAGC
AACTCACCGCTATGTTGCAGAGAAAATGCTAGAAAGTAGCAACAACTTAGCCGAGTACCGTTTCTAA

>3259
ATGATGAAAAAAACAATCACACTATTAACTGCATTACTCCCGCTTGCTTCTGCAGTTGCCGAAGAGCCAACCTTAT
CACCAGAAATGGTTTCAGCGTCTGAAGTGATCAGCACGCAAGAAAACCAAACCTATACCTATGTTCGCTGTTGGTA
TCGCACCAGCTACTCGAAAGATGATCCGGCGACCGATTGGGAATGGGCAAAAAACGAAGATGGTAGCTACTTCAC
CATTGACGGCTACTGGTGGAGCTCCGTTTCATTTAAAAACATGTTCTACACCAACACGTCGCAAAACGTTATCCGT
CAGCGTTGTGAAGCAACATTAGATTTGGCGAACGAGAACGCAGACATTACGTTCTTCGCCGCTGACAATCGCTTCT
CATACAACCACACGATCTGGAGCAACGACGCAGCAATGCAGCCAGATCAAATCAACAAAGTGGTTGCACTCGGTG
ACAGCTTGTCTGATACAGGCAACATCTTTAACGCATCACAATGGCGCTTCCCTAACCCGAACAGCTGGTTCTTAGG
TCACTTCTCCAACGGTTTTGTGTGGACAGAATACATTGCCAAAGCGAAGAACCTTCCGCTCTACAACTGGGCAGTT
GGCGGCGCGGCTGGTGAGAACCAATACATCGCGCTAACAGGGGTTGGTGATCAAGTTTCTTCGTACTTAACCTACG
CAAAACTGGCGAAGAACTACAAACCAGCAAACACCTTGTTTACGCTTGAGTTTGGTTTGAATGACTTCATGAACTA
CAACCGTGGCGTTCCAGAAGTGAAAGCAGATTATGCAGAAGCACTGATTCGTTTGACGGACGCAGGTGCGAAGAA
CTTCATGTTGATGACACTGCCAGACGCGACGAAAGCGCCTCAGTTTAAGTACTCAACACAAGAAGAGATCGACAA
AATTCGTGCGAAAGTGCTTGAGATGAACGAGTTCATCAAGGCACAAGCGATGTACTACAAAGCGCAAGGTTACAA
CATCACGTTGTTTGATACTCACGCCTTGTTCGAGACGCTAACTTCTGCGCCAGAAGAGCACGGTTTCGTGAACGCG
AGTGATCCTTGTTTGGACATCAACCGCTCATCGTCTATCGATTACATGTACACCCACGCATTGCGCTCTGAGTGTGC

GGCGTCTGGTGCTGAGAAGTTTGTGTTCTGGGATGTCACGCACCCAACAACAGCAACTCACCGCTATGTTGCAGAG
AAAATGCTAGAAAGTAGCAACAACTTAGCCGAGTACCGTTTCTAA

>NIHCB0603
ATGATGAAAAAAACAATCACACTATTAACTGCATTACTCCCGCTTGCTTCTGCAGTTGCCGAAGAGCCAA
CCTTATCACCAGAAATGGTTTCAGCGTCTGAAGTGATCAGTACGCAAGAAAACCAAACCTATACCTATGT
TCGCTGTTGGTATCGCACCAGCTACTCGAAAGATGATCCAGCGACCGATTGGGAATGGGCAAAAAACGAA
GATGGTAGCTACTTCACCATTGACGGCTACTGGTGGAGCTCCGTTTCATTTAAAAACATGTTCTACACCA
ACACGTCGCAAAACGTTATCCGTCAGCGTTGTGAAGCAACATTAGATTTGGCGAACGAGAACGCAGACAT
TACGTTCTTCGCCGCTGACAATCGCTTCTCATACAACCACACGATCTGGAGCAACGACGCAGCAATGCAG
CCAGATCAAATCAACAAAGTGGTTGCACTCGGTGACAGCTTGTCTGATACAGGCAACATCTTTAACGCAT
CACAATGGCGCTTCCCTAACCCGAACAGCTGGTTCTTAGGTCACTTCTCCAACGGTTTTGTTTGGACAGA
ATACATTGCCAAAGCGAAGAACCTTCCGCTCTACAACTGGGCAGTTGGCGGCGCGGCTGGTGAGAACCAA
TACATCGCGCTAACAGGGGTTGGTGAGCAAGTTTCTTCGTACTTAACCTACGCAAAACTGGCGAAGAACT
ACAAACCAGCAAACACCTTGTTTACGCTTGAGTTTGGTTTGAATGACTTCATGAACTACAACCGTGGCGT
TCCAGAAGTGAAAGCGGATTATGCAGAAGCACTGATTCGTTTGACGGACGCAGGTGCGAAGAACTTCATG
TTGATGACACTGCCAGATGCGACGAAAGCGCCTCAGTTTAAGTACTCAACACAAGAAGAGATCGACAAAA
TTCGTGCGAAAGTGCTTGAGATGAACGAGTTCATCAAGGCACAAGCGATGTACTACAAAGCGCAAGGTTA
CAACATCACGTTGTTTGATACTCACGCCTTGTTCGAGACGCTAACTTCTGCGCCAGAAGAGCACGGTTTC
GTGAACGCGAGCGATCCTTGTTTGGACATCAACCGCTCATCGTCTGTCGATTACATGTACACCCACGCAT
TGCGCTCTGAGTGTGCAGCGTCTGGTGCTGAGAAGTTTGTGTTCTGGGATGTCACGCATCCAACAACAGC
AACTCACCGCTATGTTGCAGAGAAAATGCTAGAAAGTAGCAACAACTTAGCCGAGTACCGTTTCTAA

>949
ATGATGAAAAAAACAATCACACTATTAACTGCATTACTCCCGCTTGCTTCTGCAGTTGCCGAAGAGCCAA
CCTTATCACCAGAAATGGTTTCAGCGTCTGAAGTGATCAGCACGCAAGAAAACCAAACCTATACCTATGT
TCGCTGTTGGTATCGCACCAGCTACTCGAAAGATGATCCAGCGACCGATTGGGAATGGGCAAAAAACGAA
GATGGTAGCTACTTCACCATTGACGGCTACTGGTGGAGCTCCGTTTCATTTAAAAACATGTTCTACACCA
ACACGTCGCAAAACGTTATCCGTCAGCGTTGTGAAGCAACATTAGATTTGGCGAACGAGAACGCAGACAT
TACGTTCTTCGCCGCTGACAATCGCTTCTCATACAACCACACGATCTGGAGCAACGACGCAGCAATGCAG
CCAGATCAAATCAACAAAGTGGTTGCACTCGGTGACAGCTTGTCTGATACAGGCAACATCTTTAACGCAT
CACAATGGCGCTTCCCTAACCCGAACAGCTGGTTCTTAGGTCACTTCTCCAACGGTTTTGTTTGGACAGA
ATACATTGCCAAAGCGAAGAACCTTCCGCTCTACAACTGGGCAGTTGGCGGCGCGGCTGGTGAGAACCAA
TACATCGCGCTAACAGGGGTTGGTGAGCAAGTTTCTTCGTACTTAACCTACGCAAAACTGGCGAAGAACT
ACAAACCAGCAAACACCTTGTTTACGCTTGAGTTTGGTTTGAATGACTTCATGAACTACAACCGTGGCGT
TCCAGAAGTGAAAGCGGATTATGCAGAAGCACTGATTCGTTTGACGGACGCAGGTGCGAAGAACTTCATG
TTGATGACACTGCCAGATGCGACGAAAGCGCCTCAGTTTAAGTACTCAACACAAGAAGAGATCGACAAAA
TTCGTGCGAAAGTGCTTGAGATGAACGAGTTCATCAAGGCACAAGCGATGTACTACAAAGCGCAAGGTTA
CAACATCACGTTGTTTGATACTCACGCCTTGTTCGAGACGCTAACTTCTGCGCCAGAAGAGCACGGTTTC
GTGAACGCGAGCGATCCTTGTTTGGACATCAACCGCTCATCGTCTGTCGATTACATGTACACCCACGCAT
TGCGCTCTGAGTGTGCAGCGTCTGGTGCTGAGAAGTTTGTGTTCTGGGATGTCACGCATCCAACAACAGC
AACTCACCGCTATGTTGCAGAGAAAATGCTAGAAAGTAGCAACAACTTAGCCGAGTACCGTTTCTAA

>VPCR-2010
ATGATGAAAAAAACAATCACACTATTAACTGCATTACTCCCGCTTGCTTCTGCAGTTGCCGAAGAGCCAACCTTAT
CACCAGAAATGGTTTCAGCGTCTGAAGTGATCAGCACGCAAGAAAACCAAACCTATACCTATGTTCGCTGTTGGTA
TCGCACCAGCTACTCGAAAGATGATCCGGCGACCGATTGGGAATGGGCAAAAAACGAAGATGGTAGCTACTTCAC
CATTGACGGCTACTGGTGGAGCTCCGTTTCATTTAAAAACATGTTCTACACCAACACGTCGCAAAACGTTATCCGT
CAGCGTTGTGAAGCAACATTAGATTTGGCGAACGAGAACGCAGACATTACGTTCTTCGCCGCTGACAATCGCTTCT
CATACAACCACACGATCTGGAGCAACGACGCAGCAATGCAGCCAGATCAAATCAACAAAGTGGTTGCACTCGGTG
ACAGCTTGTCTGATACAGGCAACATCTTTAACGCATCACAATGGCGCTTCCCTAACCCGAACAGCTGGTTCTTAGG

TCACTTCTCCAACGGTTTTGTGTGGACAGAATACATTGCCAAAGCGAAGAACCTTCCGCTCTACAACTGGGCAGTT
GGCGGCGCGGCTGGTGAGAACCAATACATCGCGCTAACAGGGGTTGGTGATCAAGTTTCTTCGTACTTAACCTACG
CAAAACTGGCGAAGAACTACAAACCAGCAAACACCTTGTTTACGCTTGAGTTTGGTTTGAATGACTTCATGAACTA
CAACCGTGGCGTTCCAGAAGTGAAAGCGGATTATGCAGAAGCACTGATTCGTTTGACGGACGCAGGTGCGAAGAA
CTTCATGTTGATGACACTGCCAGACGCGACGAAAGCGCCTCAGTTTAAGTACTCAACACAAGAAGAGATCGACAA
AATTCGTGCGAAAGTGCTTGAGATGAACGAGTTCATCAAGGCACAAGCGATGTACTACAAAGCGCAAGGTTACAA
CATCACGTTGTTTGATACTCACGCCTTGTTCGAGACGCTAACTTCTGCGCCCGAAGAGCACGGTTTCGTGAACGCG
AGTGATCCTTGTTTGGACATCAACCGCTCATCGTCTGTCGATTACATGTACACCCACGCATTGCGCTCTGAGTGTGC
GGCGTCTGGTGCTGAGAAGTTTGTGTTCTGGGATGTCACGCACCCAACAACAGCAACTCACCGCTATGTTGCAGAG
AAAATGCTAGAAAGTAGCAACAACTTAGCCGAGTACCGTTTCTAA

>NIHCB0757
ATGATGAAAAAAACAATCACACTATTAACTGCATTACTCCCGCTTGCTTCTGCAGTTGCCGAAGAGCCAACCTTAT
CACCAGAAATGGTTTCAGCGTCTGAAGTGTTCAGCACGCAAGAAAACCAAACCTATACCTATGTTCGCTGTTGGTA
TCGCACCAGCTACTCGAAAGATGACCCGGCGACCGATTGGGAATGGGCAAAAAACGAAGATGGTAGCTACTTCAC
CATTGACGGCTACTGGTGGAGCTCCGTTTCATTTAAAAACATGTTCTACACCAACACGTCGCAAAACGTTATCCGT
CAGCGTTGTGAAGCAACATTAGATTTGGCGAACGAGAACGCAGACATTACGTTCTTCGCCGCTGACAATCGCTTCT
CATACAACCACACGATCTGGAGCAACGACGCAGCAATGCAGCCAGATCAAATCAACAAAGTGGTTGCACTCGGTG
ACAGCTTGTCTGATACAGGCAACATCTTTAACGCATCACAATGGCGCTTCCCTAACCCGAACAGCTGGTTCTTAGG
TCACTTCTCCAACGGTTTTGTGTGGACAGAATACATTGCCAAAGCGAAGAACCTTCCGCTCTACAACTGGGCAGTT
GGCGGCGCGGCTGGTGAGAACCAATACATCGCGCTAACAGGGGTTGGTGATCAAGTTTCTTCGTACTTAACCTACG
CAAAACTGGCGAAGAACTACAAACCAGCAAACACCTTGTTTACGCTTGAGTTTGGTTTGAATGACTTCATGAACTA
CAACCGTGGCGTTCCAGAAGTGAAAGCGGATTATGCAGAAGCACTGATTCGTTTGACGGACGCAGGTGCGAAGAA
CTTCATGTTGATGACACTGCCAGACGCGACGAAAGCGCCTCAGTTTAAGTACTCAACACAAGAAGAGATCGACAA
AATTCGTGCGAAAGTGCTTGAGATGAACGAGTTCATCAAGGCACAAGCGATGTACTACAAAGCGCAAGGTTACAA
CATCACGTTGTTTGATACTCACGCCTTGTTCGAGACGCTAACTTCTGCGCCAGAAGAGCACGGTTTCGTGAACGCG
AGCGATCCTTGTTTGGACATCAACCGCTCATCGTCTGTCGATTACATGTACACCCACGCATTGCGCTCTGAGTGTGC
GGCGTCCGGTGCTGAGAAATTTGTGTTCTGGGATGTCACGCACCCAACAACAGCAACTCACCGCTATGTTGCAGAG
AAAATGCTAGAAAGTAGCAACAACTTAGCCGAGTACCGTTTCTAA

>S174
ATGATGAAAAAAACAATCACACTATTAACTGCATTACTCCCGCTTGCTTCTGCAGTTGCCGAAGAGCCAA
CCTTATCACCAGAAATGGTTTCAGCGTCTGAAGTGATCAGCACGCAAGAAAACCAAACCTATACCTATGT
TCGCTGTTGGTATCGCACCAGCTACTCGAAAGATGATCCGGCGACCGATTGGGAATGGGCAAAAAACGAA
GATGGTAGCTACTTCACCATTGACGGCTACTGGTGGAGCTCCGTTTCATTTAAAAACATGTTCTACACCA
ACACGTCGCAAAACGTTATCCGTCAGCGTTGTGAAGCAACATTAGATTTGGCGAACGAGAACGCAGACAT
TACGTTCTTCGCCGCTGACAATCGCTTCTCATACAACCACACGATCTGGAGCAACGACGCAGCAATGCAG
CCAGATCAAATCAACAAAGTGGTTGCACTCGGTGACAGCTTGTCTGATACAGGCAACATCTTTAACGCAT
CACAATGGCGCTTCCCTAACCCGAACAGCTGGTTCTTAGGTCACTTCTCCAACGGTTTTGTGTGGACAGA
ATACATTGCCAAAGCGAAGAACCTTCCGCTCTACAACTGGGCAGTTGGCGGCGCGGCTGGTGAGAACCAA
TACATCGCGCTAACAGGGGTTGGTGATCAAGTTTCTTCGTACTTAACCTACGCAAAACTGGCGAAGAACT
ACAAACCAGCAAACACCTTGTTTACGCTTGAGTTTGGTTTGAATGACTTCATGAACTACAACCGTGGCGT
TCCAGAAGTGAAAGCGGATTATGCAGAAGCACTGATTCGTTTGACGGACGCAGGTGCGAAGAACTTCATG
TTGATGACACTGCCAGACGCGACGAAAGCGCCTCAGTTTAAGTACTCAACACAAGAAGAGATCGACAAAA
TTCGTGCGAAAGTGCTTGAGATGAACGAGTTCATCAAGGCACAAGCGATGTACTACAAAGCGCAAGGTTA
CAACATCACGTTGTTTGATACTCACGCCTTGTTCGAGACGCTAACTTCTGCGCCAGAAGAGCACGGTTTC
GTGAACGCGAGTGATCCTTGTTTGGACATCAACCGCTCATCGTCTGTCGATTACATGTACACCCACGCAT
TGCGCTCTGAGTGTGCGGCGTCCGGTGCTGAGAAATTTGTGTTCTGGGATGTCACGCACCCAACAACAGC
AACTCACCGCTATGTTGCAGAGAAAATGCTAGAAAGTAGCAACAACTTAGCCGAGTACCGTTTCTAA

>S173

ATGATGAAAAAAACAATCACACTATTAACTGCATTACTCCCGCTTGCTTCTGCAGTTGCCGAAGAGCCAA
CCTTATCACCAGAAATGGTTTCAGCGTCTGAAGTGATCAGCACGCAAGAAAACCAAACCTATACCTATGT
TCGCTGTTGGTATCGCACCAGCTACTCGAAAGATGATCCGGCGACCGATTGGGAATGGGCAAAAAACGAA
GATGGTAGCTACTTCACCATTGACGGCTACTGGTGGAGCTCCGTTTCATTTAAAAACATGTTCTACACCA
ACACGTCGCAAAACGTTATCCGTCAGCGTTGTGAAGCAACATTAGATTTGGCGAACGAGAACGCAGACAT
TACGTTCTTCGCCGCTGACAATCGCTTCTCATACAACCATACGATCTGGAGCAACGACGCAGCAATGCAG
CCAGATCAAATCAACAAAGTGGTTGCACTCGGTGACAGCTTGTCTGATACAGGCAACATCTTTAACGCAT
CACAATGGCGCTTCCCTAACCCGAACAGCTGGTTCTTAGGTCACTTCTCCAACGGTTTTGTGTGGACAGA
ATACATTGCCAAAGCGAAGAACCTTCCGCTCTACAACTGGGCAGTTGGCGGCGCGGCTGGTGAGAACCAA
TACATCGCGCTAACAGGGGTTGGTGATCAAGTTTCTTCGTACTTAACCTACGCAAAACTGGCGAAGAACT
ACAAACCAGCAAACACCTTGTTTACGCTTGAGTTTGGTTTGAATGACTTCATGAACTACAACCGTGGCGT
TCCAGAAGTGAAAGCGGATTATGCAGAAGCACTGATTCGTTTGACGGACGCAGGTGCGAAGAACTTCATG
TTGATGACACTGCCAGATGCGACGAAAGCGCCTCAGTTTAAGTACTCAACACAAGAAGAGATCGACAAAA
TTCGTGCGAAAGTGCTTGAGATGAACGAGTTCATCAAGGCACAAGCGATGTACTACAAAGCGCAAGGTTA
CAACATCACGTTGTTTGATACTCACGCCTTGTTCGAGACGCTAACTTCTGCGCCAGAAGAGCACGGTTTC
GTGAACGCGAGCGATCCTTGTTTGGACATCAACCGCTCATCGTCTGTCGATTACATGTACACCCACGCAT
TGCGCTCTGAGTGTGCAGCGTCTGGTGCTGAGAAGTTTGTGTTCTGGGATGTCACGCATCCAACAACAGC
AACTCACCGCTATGTTGCAGAGAAAATGCTAGAAAGTAGCAACAACTTAGCCGAGTACCGTTTCTAA

>S172
ATGATGAAAAAAACAATCACACTATTAACTGCATTACTCCCGCTTGCTTCTGCAGTTGCCGAAGAGCCAA
CCTTATCACCAGAAATGGTTTCAGCGTCTGAAGTGATCAGCACGCAAGAAAACCAAACCTATACCTATGT
TCGCTGTTGGTATCGCACCAGCTACTCGAAAGATGATCCGGCGACCGATTGGGAATGGGCAAAAAACGAA
GATGGTAGCTACTTCACCATTGACGGCTACTGGTGGAGCTCCGTTTCATTTAAAAACATGTTCTACACCA
ACACGTCGCAAAACGTTATCCGTCAGCGTTGTGAAGCAACATTAGATTTGGCGAACGAGAACGCAGACAT
TACGTTCTTCGCCGCTGACAATCGCTTCTCATACAACCACACGATCTGGAGCAACGACGCAGCAATGCAG
CCAGATCAAATCAACAAAGTGGTTGCACTCGGTGACAGCTTGTCTGATACAGGCAACATCTTTAACGCAT
CACAATGGCGCTTCCCTAACCCGAACAGCTGGTTCTTAGGTCACTTCTCCAACGGTTTTGTGTGGACAGA
ATACATTGCCAAAGCGAAGAACCTTCCGCTCTACAACTGGGCAGTTGGCGGCGCGGCTGGTGAGAACCAA
TACATCGCGCTAACAGGGGTTGGTGATCAAGTTTCTTCGTACTTAACCTACGCAAAACTGGCGAAGAACT
ACAAACCAGCAAACACCTTGTTTACGCTTGAGTTTGGTTTGAATGACTTCATGAACTACAACCGTGGCGT
TCCAGAAGTGAAAGCAGATTATGCAGAAGCACTGATTCGTTTGACGGACGCAGGTGCAAAGAACTTCATG
TTGATGACACTGCCAGACGCGACGAAAGCGCCTCAGTTTAAGTACTCAACACAAGAAGAGATCGACAAAA
TTCGTGCGAAAGTGCTTGAGATGAACGAGTTCATCAAGGCACAAGCGATGTACTACAAAGCGCAAGGTTA
CAACATCACGTTGTTTGATACTCACGCCTTGTTCGAGACGCTAACTTCTGCGCCAGAAGAGCACGGTTTC
GTGAACGCGAGCGATCCTTGTTTGGACATCAACCGCTCATCGTCTGTCGATTACATGTACACCCACGCAT
TGCGCTCTGAGTGTGCGGCGTCTGGTGCTGAGAAGTTTGTGTTCTGGGATGTCACGCATCCAACAACAGC
AACTCACCGCTATGTTGCAGAGAAAATGCTAGAAAGTAGCAACAACTTAGCCGAGTACCGTTTCTAA

>S171
ATGATGAAAAAAACAATCACACTATTAACTGCATTACTCCCGCTTGCTTCTGCAGTTGCCGAAGAGCCAA
CCTTATCACCAGAAATGGTTTCAGCGTCTGAAGTGATCAGCACGCAAGAAAACCAAACCTATACCTATGT
TCGCTGTTGGTATCGCACCAGCTACTCGAAAGATGATCCGGCGACCGATTGGGAATGGGCAAAAAACGAA
GATGGTAGCTACTTCACCATTGACGGCTACTGGTGGAGCTCCGTTTCATTTAAAAACATGTTCTACACCA
ACACGTCGCAAAACGTTATCCGTCAGCGTTGTGAAGCAACATTAGATTTGGCGAACGAGAACGCAGACAT
TACGTTCTTCGCCGCTGACAATCGCTTCTCATACAACCACACGATCTGGAGCAACGACGCAGCAATGCAG
CCAGATCAAATCAACAAAGTGGTTGCACTCGGTGACAGCTTGTCTGATACAGGCAACATCTTTAACGCAT
CACAATGGCGCTTCCCTAACCCGAACAGCTGGTTCTTAGGTCACTTCTCCAACGGTTTTGTGTGGACAGA
ATACATTGCCAAAGCGAAGAACCTTCCGCTCTACAACTGGGCAGTTGGCGGCGCGGCTGGTGAGAACCAA
TACATCGCGCTAACAGGGGTTGGTGATCAAGTTTCTTCGTACTTAACCTACGCAAAACTGGCGAAGAACT
ACAAACCAGCAAACACCTTGTTTACGCTTGAGTTTGGTTTGAATGACTTCATGAACTACAACCGTGGCGT

TCCAGAAGTGAAAGCGGATTATGCAGAAGCACTGATTCGTTTGACGGACGCAGGTGCGAAGAACTTCATG
TTGATGACACTGCCAGACGCGACGAAAGCGCCTCAGTTTAAGTACTCAACACAAGAAGAGATCGACAAAA
TTCGTGCGAAAGTGCTTGAGATGAACGAGTTCATCAAGGCACAAGCGATGTACTACAAAGCGCAAGGTTA
CAACATCACGTTGTTTGATACTCACGCCTTGTTCGAGACGCTAACTTCTGCGCCCGAAGAGCACGGTTTC
GTGAACGCGAGTGATCCTTGTTTGGACATCAACCGCTCATCGTCTGTCGATTACATGTACACCCACGCAT
TGCGCTCTGAGTGTGCAGCGTCTGGTGCTGAGAAGTTTGTGTTCTGGGATGTCACGCATCCAACAACAGC
AACTCACCGCTATGTTGCAGAGAAAATGCTAGAAAGTAGCAACAACTTAGCCGAGTACCGTTTCTAA

>S170
ATGATGAAAAAAACAATCACACTATTAACTGCATTACTCCCGCTTGCTTCTGCAGTTGCCGAAGAGCCAA
CCTTATCACCAGAAATGGTTTCAGCGTCTGAAGTGATCAGCACGCAAGAAAACCAAACCTATACCTATGT
TCGCTGTTGGTATCGCACCAGCTACTCGAAAGATGATCCGGCGACCGATTGGGAATGGGCAAAAAACGAA
GATGGTAGCTACTTCACCATTGACGGCTACTGGTGGAGCTCCGTTTCATTTAAAAACATGTTCTACACCA
ACACGTCGCAAAACGTTATCCGTCAGCGTTGTGAAGCAACATTAGATTTGGCGAACGAGAACGCAGACAT
TACGTTCTTCGCCGCTGACAATCGCTTCTCATACAACCACACGATCTGGAGCAACGACGCAGCAATGCAG
CCAGATCAAATCAACAAAGTGGTTGCTCTCGGTGACAGCTTGTCTGATACAGGCAACATCTTTAACGCAT
CACAATGGCGCTTCCCTAACCCGAACAGCTGGTTCTTAGGTCACTTCTCCAACGGTTTTGTGTGGACAGA
ATACATTGCCAAAGCGAAGAACCTTCCGCTCTACAACTGGGCAGTTGGCGGCGCGGCTGGTGAGAACCAA
TACATCGCGCTAACAGGGGTTGGTGATCAAGTTTCTTCGTACTTAACCTACGCAAAACTGGCGAAGAACT
ACAAACCAGCAAACACCTTGTTTACGCTTGAGTTTGGTTTGAATGACTTCATGAACTACAACCGTGGCGT
TCCAGAAGTGAAAGCAGATTATGCAGAAGCACTGATTCGTTTGACGGACGCAGGTGCGAAGAACTTCATG
TTGATGACACTGCCAGACGCGACAAAGGCGCCTCAGTTTAAGTACTCAACACAAGAAGAGATCGACAAAA
TTCGTGCGAAAGTGCTTGAGATGAACGAGTTCATCAAGGCACAAGCGATGTACTACAAAGCGCAAGGTTA
CAACATCACGTTGTTTGATACTCACGCCTTGTTCGAGACGCTAACTTCTGCGCCAGAAGAGCACGGTTTC
GTGAACGCGAGTGATCCTTGTTTGGACATCAACCGCTCATCGTCTGTCGATTACATGTACACCCACGCAT
TGCGCTCTGAGTGTGCAGCGTCTGGTGCTGAGAAGTTTGTGTTCTGGGATGTCACGCATCCAACAACAGC
AACTCACCGCTATGTTGCAGAGAAAATGCTAGAAAGTAGCAACAACTTAGCAGAGTACCGTTTCTAA

>S168
ATGATGAAAAAAACAATCACACTATTAACTGCATTACTCCCGCTTGCTTCTGCAGTTGCCGAAGAGCCAA
CCTTATCACCAGAAATGGTTTCAGCGTCTGAAGTGTTCAGCACGCAAGAAAACCAAACCTATACCTATGT
TCGCTGTTGGTATCGCACCAGCTACTCGAAAGATGACCCGGCGACCGATTGGGAATGGGCAAAAAACGAA
GATGGTAGCTACTTCACCATTGACGGCTACTGGTGGAGCTCCGTTTCATTTAAAAACATGTTCTACACCA
ACACGTCGCAAAACGTTATCCGTCAGCGTTGTGAAGCAACATTAGATTTGGCGAACGAGAACGCAGACAT
TACGTTCTTCGCCGCTGACAATCGCTTCTCATACAACCACACGATCTGGAGCAACGACGCAGCAATGCAG
CCAGATCAAATCAACAAAGTGGTTGCACTCGGTGACAGCTTGTCTGATACAGGCAACATCTTTAACGCAT
CACAATGGCGCTTCCCTAACCCGAACAGCTGGTTCTTAGGTCACTTCTCCAACGGTTTTGTGTGGACAGA
ATACATTGCCAAAGCGAAGAACCTTCCGCTCTACAACTGGGCAGTTGGCGGCGCGGCTGGTGAGAACCAA
TACATCGCGCTAACAGGGGTTGGTGATCAAGTTTCTTCGTACTTAACCTACGCAAAACTGGCGAAGAACT
ACAAACCAGCAAACACCTTGTTTACGCTTGAGTTTGGTTTGAATGACTTCATGAACTACAACCGTGGCGT
TCCAGAAGTGAAAGCGGATTATGCAGAAGCACTGATTCGTTTGACGGACGCAGGTGCGAAGAACTTCATG
TTGATGACACTGCCAGACGCGACGAAAGCGCCTCAGTTTAAGTACTCAACACAAGAAGAGATCGACAAAA
TTCGTGCGAAAGTGCTTGAGATGAACGAGTTCATCAAGGCACAAGCGATGTACTACAAAGCGCAAGGTTA
CAACATCACGTTGTTTGATACTCACGCCTTGTTCGAGACGCTAACTTCTGCGCCAGAAGAGCACGGTTTC
GTGAACGCGAGCGATCCTTGTTTGGACATCAACCGCTCATCGTCTGTCGACTACATGTACACCCACGCAT
TGCGCTCTGAGTGTGCGGCGTCTGGTGCTGAGAAGTTTGTATTCTGGGATGTCACGCACCCAACAACAGC
AACTCACCGCTATGTTGCAGAGAAAATGCTAGAAAGTAGCAACAACTTAGCCGAGTACCGTTTCTAA

>S167
ATGATGAAAAAAACAATCACACTATTAACTGCATTACTCCCGCTTGCTTCTGCAGTTGCCGAAGAGCCAACCTTAT
CACCAGAAATGGTTTCACCGTCTGAAGTGATCAGCACGCAAAAAAACCAAACCTATACCTATGTTCGCTGTTGGTA

TCGCACCAGCTACTCGAAAGATGATCCGGCGACCGATTGGGAATGGGCAAAAAACGAAGATGGTAGCTACTTCAC
CATTGACGGCTACTGGTGGAGCTCCGTTTCATTTAAAAACATGTTCTACACCAACACGTCGCAAAACGTTATCCGT
CAGCGTTGTGAAGCAACATTAGATTTGGCGAACGAGAACGCAGACATTACGTTCTTCGCCGCTGACAATCGCTTCT
CATACAACCACACGATCTGGAGCAACGACGCAGCAATGCAGCCAGATCAAATCAACAAAGTGGTTGCACTCGGTG
ACAGCTTGTCTGATACCGGCAACATCTTTAACGCATCACAATGGCGCTTCCCTAACCCGAACAGCTGGTTCTTAGG
TCACTTCTCCAACGGTTTTGTGTGGACAGAATACATTGCCAAAGCGAAGAACCTTCCGCTCTACAACTGGGCAGTT
GGCGGCGCGGCTGGTGAGAACCAATACATCGCGCTAACAGGGGTTGGTGATCAAGTTTCTTCGTACTTAACCTACG
CAAAACTGGCGAAGAACTACAAACCAGCAAACACCTTGTTTACGCTTGAGTTTGGTTTGAATGACTTCATGAACTA
CAACCGTGGCGTTCCTGAAGTGAAAGCGGATTATGCAGAAGCACTGATTCGTTTGACGGACGCAGGTGCGAAGAA
CTTCATGTTGATGACACTGCCAGACGCGACGAAAGCGCCTCAGTTTAAGTACTCAACACAAGAAGAGATCGACAA
AATTCGTGCGAAAGTGCTTGAGATGAACGAGTTCATCAAGGCACAAGCGATGTACTACAAAGCGCAAGGTTACAA
CATCACGTTGTTTGATACTCACGCCTTGTTCGAGACGCTAACTTCTGCGCCAGAAGAGCACGGTTTCGTGAACGCG
AGCGATCCTTGTTTGGACATCAACCGCTCATCGTCTGTCGATTACATGTACACCCACGCATTGCGCTCTGAGTGTGC
AGCGTCTGGTGCTGAGAAGTTTGTGTTCTGGGATGTCACGCATCCAACAACAGCAACTCACCGCTATGTTGCAGAG
AAAATGCTAGAAAGTAGCAACAACTTAGCCGAGTACCGTTTCTAA

>S166
ATGATGAAAAAAACAATCACACTATTAACTGCATTACTCCCGCTTGCTTCTGCAGTTGCCGAAGAGCCAA
CCTTATCACCAGAAATGGTTTCAGCGTCTGAAGTGATCAGCACGCAAGAAAACCAAACCTATACCTATGT
TCGCTGTTGGTATCGCACCAGCTACTCGAAAGATGATCCGGCGACCGATTGGGAATGGGCAAAAAACGAA
GATGGTAGCTACTTCACCATTGACGGCTACTGGTGGAGCTCCGTTTCATTTAAAAACATGTTCTACACCA
ACACGTCGCAAAACGTTATCCGTCAGCGTTGTGAAGCAACATTAGATTTGGCGAACGAGAACGCAGACAT
TACGTTCTTCGCCGCTGACAATCGCTTCTCATACAACCACACGATCTGGAGCAACGACGCAGCAATGCAG
CCAGATCAAATCAACAAAGTGGTTGCACTCGGTGACAGCTTGTCTGATACAGGCAACATCTTTAACGCAT
CACAATGGCGCTTCCCTAACCCGAACAGCTGGTTCTTAGGTCACTTCTCCAACGGTTTTGTGTGGACAGA
ATACATTGCCAAAGCGAAGAACCTTCCGCTCTACAACTGGGCAGTTGGCGGCGCGGCTGGTGAGAACCAA
TACATCGCGCTAACAGGGGTTGGTGATCAAGTTTCTTCGTACTTAACCTACGCAAAACTGGCGAAGAACT
ACAAACCAGCAAACACCTTGTTTACGCTTGAGTTTGGTTTGAATGACTTCATGAACTACAACCGTGGCGT
TCCAGAAGTGAAAGCAGATTATGCAGAAGCACTGATTCGTTTGACGGACGCAGGTGCGAAGAACTTCATG
TTGATGACACTGCCAGACGCGACGAAAGCGCCTCAGTTTAAGTACTCAACACAAGAAGAGATCGACAAAA
TTCGTGCGAAAGTGCTTGAGATGAACGAGTTCATCAAGGCACAAGCGATGTACTACAAAGCGCAAGGTTA
CAACATCACGTTGTTTGATACTCACGCCTTGTTCGAGACGCTAACTTCTGCGCCCGAAGAGCACGGTTTC
GTGAACGCGAGTGATCCTTGTTTGGACATCAACCGCTCATCGTCTGTCGATTACATGTACACCCACGCAT
TGCGCTCTGAGTGTGCGGCGTCTGGTGCTGAGAAGTTTGTGTTCTGGGATGTCACGCACCCAACAACAGC
AACTCACCGCTATGTTGCAGAGAAAATGCTAGAAAGTAGCAACAACTTAGCCGAGTACCGTTTCTAA

>S165
ATGATGAAAAAAACAATCACACTATTAACTGCATTACTCCCGCTTGCTTCTGCAGTTGCCGAAGAGCCAA
CCTTATCACCAGAAATGGTTTCAGCGTCTGAAGTGATCAGCACGCAAGAAAACCAAACCTATACCTATGT
TCGCTGTTGGTATCGCACCAGCTACTCGAAAGATGATCCGGCGACCGATTGGGAATGGGCAAAAAACGAA
GATGGTAGCTACTTCACCATTGACGGCTACTGGTGGAGCTCCGTTTCATTTAAAAACATGTTCTACACCA
ACACGTCGCAAAACGTTATCCGTCAGCGTTGTGAAGCAACATTAGATTTGGCGAACGAGAACGCAGACAT
TACGTTCTTCGCCGCTGACAATCGCTTCTCATACAACCACACGATCTGGAGCAACGACGCAGCAATGCAG
CCAGATCAAATCAACAAAGTGGTTGCACTCGGTGACAGCTTGTCTGATACAGGCAACATCTTTAACGCAT
CACAATGGCGCTTCCCTAACCCGAACAGCTGGTTCTTAGGTCACTTCTCCAACGGTTTTGTGTGGACAGA
ATACATTGCCAAAGCGAAGAACCTTCCGCTCTACAACTGGGCAGTTGGCGGCGCGGCTGGTGAGAACCAA
TACATCGCGCTAACAGGGGTCGGTGATCAAGTTTCTTCGTACTTAACCTACGCAAAACTGGCGAAGAACT
ACAAACCAGCAAACACCTTGTTTACGCTTGAGTTTGGTTTGAATGACTTCATGAACTACAACCGTGGCGT
TCCAGAAGTGAAAGCGGATTATGCAGAAGCACTGATTCGTTTGACGGACGCAGGTGCGAAGAACTTCATG
TTGATGACACTGCCAGACGCGACGAAAGCGCCTCAGTTTAAGTACTCAACACAAGAAGAGATCGACAAAA
TTCGTGCGAAAGTGCTTGAGATGAACGAGTTCATCAAGGCACAAGCGATGTACTACAAAGCGCAAGGTTA

CAACATCACGTTGTTTGATACTCACGCCTTGTTCGAGACGCTAACTTCTGCGCCCGAAGAGCACGGTTTC
GTGAACGCGAGTGATCCTTGTTTGGACATCAACCGCTCATCGTCTGTCGATTACATGTACACCCACGCAT
TGCGCTCTGAGTGTGCGGCGTCTGGTGCTGAGAAGTTTGTGTTCTGGGATGTCACGCACCCAACAACAGC
AACTCACCGCTATGTTGCAGAGAAAATGCTAGAAAGTAGCAACAACTTAGCCGAGTACCGTTTCTAA

>S164
ATGATGAAAAAAACAATCACACTATTAACTGCATTACTCCCGCTTGCTTCTGCAGTTGCCGAAGAGCCAACCTTAT
CACCAGAAATGGTTTCAGCGTCTGAAGTGATCAGCACGCAAGAAAACCAAACCTATACCTATGTTCGCTGTTGGTA
TCGCACCAGCTACTCGAAAGATGATCCGGCGACCGATTGGGAATGGGCAAAAAACGAAGATGGTAGCTACTTCAC
CATTGACGGCTACTGGTGGAGCTCCGTTTCATTTAAAAACATGTTCTACACCAACACGTCGCAAAACGTTATCCGT
CAGCGTTGTGAAGCAACATTAGATTTGGCGAACGAGAACGCAGACATTACGTTCTTCGCCGCTGACAATCGCTTCT
CATACAACCACACGATCTGGAGCAACGACGCAGCAATGCAGCCAGATCAAATCAACAAAGTGGTTGCACTCGGTG
ACAGCTTGTCTGATACAGGCAACATCTTTAACGCATCACAATGGCGCTTCCCTAACCCGAACAGCTGGTTCTTAGG
TCACTTCTCCAACGGTTTTGTGTGGACAGAATACATTGCCAAAGCGAAGAACCTTCCGCTCTACAACTGGGCAGTT
GGCGGCGCGGCTGGTGAGAACCAATACATCGCGCTAACAGGGGTTGGTGATCAAGTTTCTTCGTACTTAACCTACG
CAAAACTGGCGAAGAACTACAAACCAGCAAACACCTTGTTTACGCTTGAGTTTGGTTTGAATGACTTCATGAACTA
CAACCGTGGCGTTCCAGAAGTGAAAGCGGATTATGCAGAAGCACTGATTCGTTTGACGGACGCAGGTGCGAAGAA
CTTCATGTTGATGACACTGCCAGACGCGACGAAAGCGCCTCAGTTTAAGTACTCAACACAAGAAGAGATCGACAA
AATTCGTGCGAAAGTGCTTGAGATGAACGAGTTCATCAAGGCACAAGCGATGTACTACAAAGCGCAAGGTTACAA
CATCACGTTGTTTGATACTCACGCCTTGTTCGAGACGCTAACTTCTGCGCCCGAAGAGCACGGTTTCGTGAACGCG
AGTGATCCTTGTTTGGACATCAACCGCTCATCGTCTGTCGATTACATGTACACCCACGCATTGCGCTCTGAGTGTGC
GGCGTCTGGTGCTGAGAAGTTTGTGTTCTGGGATGTCACGCACCCAACAACAGCAACTCACCGCTATGTTGCAGAG
AAAATGCTAGAAAGTAGCAACAACTTAGCCGAGTACCGTTTCTAA

>S163
ATGATGAAAAAAACAATCACACTATTAACTGCATTACTCCCGCTTGCTTCTGCAGTTGCCGAAGAGCCAA
CCTTATCACCAGAAATGGTTTCAGCGTCTGAAGTGATCAGCACGCAAGAAAACCAAACCTATACCTATGT
TCGCTGTTGGTATCGCACCAGCTACTCGAAAGATGATCCGGCGACCGATTGGGAATGGGCAAAAAACGAA
GATGGTAGCTACTTCACCATTGACGGCTACTGGTGGAGCTCCGTTTCATTTAAAAACATGTTCTACACCA
ACACGTCGCAAAACGTTATCCGTCAGCGTTGTGAAGCAACATTAGATTTGGCGAACGAGAACGCAGACAT
TACGTTCTTCGCCGCTGACAATCGCTTCTCATACAACCACACGATCTGGAGCAACGACGCAGCAATGCAG
CCAGATCAAATCAACAAAGTGGTTGCACTCGGTGACAGCTTGTCTGATACAGGCAACATCTTTAACGCAT
CACAATGGCGCTTCCCTAACCCGAACAGCTGGTTCTTAGGTCACTTCTCCAACGGTTTTGTGTGGACAGA
ATACATTGCCAAAGCGAAGAACCTTCCGCTCTACAACTGGGCAGTTGGCGGCGCGGCTGGTGAGAACCAA
TACATCGCGCTAACAGGGGTTGGTGATCAAGTTTCTTCGTACTTAACCTACGCAAAACTGGCGAAGAACT
ACAAACCAGCAAACACCTTGTTTACGCTTGAGTTTGGTTTGAATGACTTCATGAACTACAACCGTGGCGT
TCCAGAAGTGAAAGCAGATTATGCAGAAGCACTGATTCGTTTGACGGACGCAGGTGCGAAGAACTTCATG
TTGATGACACTGCCAGACGCGACAAAAGCGCCTCAGTTTAAGTACTCAACACAAGAAGAGATCGACAAAA
TTCGTGCGAAAGTGCTTGAGATGAACGAGTTCATCAAGGCACAAGCGATGTACTACAAAGCGCAAGGTTA
CAACATCACGTTGTTTGATACTCACGCCTTGTTCGAGACGCTAACTTCTGCGCCCGAAGAGCACGGTTTC
GTGAACGCGAGTGATCCTTGTTTGGACATCAACCGCTCATCGTCTGTCGATTACATGTACACCCATGCAT
TGCGCTCTGAGTGTGCGGCGTCTGGTGCTGAGAAGTTTGTGTTCTGGGATGTCACGCACCCAACAACAGC
AACTCACCGCTATGTTGCAGAGAAAATGCTAGAAAGTAGCAACAACTTAGCCGAGTACCGTTTCTAA

>S162
ATGATGAAAAAAACAATCACACTATTAACTGCATTACTCCCGCTTGCTTCTGCAGTTGCCGAAGAGCCAACCTTAT
CACCAGAAATGGTTTCAGCGTCTGAAGTGATCAGCACGCAAGAAAACCAAACCTATACCTATGTTCGCTGTTGGTA
TCGCACCAGCTACTCGAAAGATGATCCGGCGACCGATTGGGAATGGGCAAAAAACGAAGATGGTAGCTACTTCAC
CATTGACGGCTACTGGTGGAGCTCCGTTTCATTTAAAAACATGTTCTACACCAACACGTCGCAAAACGTTATCCGT
CAGCGTTGTGAAGCAACATTAGATTTGGCGAACGAGAACGCAGACATTACGTTCTTCGCCGCTGACAATCGCTTCT
CATACAACCACACGATCTGGAGCAACGACGCAGCAATGCAGCCAGATCAAATCAACAAAGTGGTTGCACTCGGTG

ACAGCTTGTCTGATACAGGCAACATCTTTAACGCATCACAATGGCGCTTCCCTAACCCGAACAGCTGGTTCTTAGG
TCACTTCTCCAACGGTTTTGTGTGGACAGAATACATTGCCAAAGCGAAGAACCTTCCGCTCTACAACTGGGCAGTT
GGCGGCGCGGCTGGTGAGAACCAATACATCGCGCTAACAGGGGTTGGTGATCAAGTTTCTTCGTACTTAACCTACG
CAAAACTGGCGAAGAACTACAAACCAGCAAACACCTTGTTTACGCTTGAGTTTGGTTTGAATGACTTCATGAACTA
CAACCGTGGCGTTCCAGAAGTGAAAGCGGATTATGCAGAAGCACTGATTCGTTTGACGGACGCAGGTGCGAAGAA
CTTCATGTTGATGACACTGCCAGACGCGACGAAAGCGCCTCAGTTTAAGTACTCAACACAAGAAGAGATCGACAA
AATTCGTGCGAAAGTGCTTGAGATGAACGAGTTCATCAAGGCACAAGCGATGTACTACAAAGCGCAAGGTTACAA
CATCACGTTGTTTGATACTCACGCCTTGTTCGAGACGCTAACTTCTGCGCCAGAAGAGCACGGTTTCGTGAACGCG
AGCGATCCTTGTTTGGACATCAACCGCTCATCGTCTGTCGATTACATGTACACCCACGCATTGCGTTCTGAGTGTGC
AGCGTCTGGTGCTGAGAAGTTTGTGTTCTGGGATGTCACGCATCCAACAACAGCAACTCACCGCTATGTTGCAGAG
AAAATGCTAGAAAGTAGCAACAACTTAGCCGAGTACCGTTTCTAA

>S161
ATGATGAAAAAAACAATCACACTATTAACTGCATTACTCCCGCTTGCTTCTGCAGTTGCCGAAGAGCCAA
CCTTATCACCAGAAATGGTTTCAGCGTCTGAAGTGATCAGCACGCAAGAAAACCAAACCTATACCTATGT
TCGCTGTTGGTATCGCACCAGCTACTCGAAAGATGATCCGGCGACCGATTGGGAATGGGCAAAAAACGAA
GATGGTAGCTACTTCACCATTGACGGCTACTGGTGGAGCTCCGTTTCATTTAAAAACATGTTCTACACCA
ACACGTCGCAAAACGTTATCCGTCAGCGTTGTGAAGCAACATTAGATTTGGCGAACGAGAACGCAGACAT
TACGTTCTTCGCCGCTGACAATCGCTTCTCATACAACCACACGATCTGGAGCAACGACGCAGCAATGCAG
CCAGATCAAATCAACAAAGTGGTTGCTCTCGGTGACAGCTTGTCTGATACAGGCAACATCTTTAACGCAT
CACAATGGCGCTTCCCTAACCCGAACAGCTGGTTCTTAGGTCACTTCTCCAACGGTTTTGTGTGGACAGA
ATACATTGCCAAAGCGAAGAACCTTCCGCTCTACAACTGGGCAGTTGGCGGCGCGGCTGGTGAGAACCAA
TACATCGCGCTAACAGGGGTTGGTGATCAAGTTTCTTCGTACTTAACCTACGCAAAACTGGCGAAGAACT
ACAAACCAGCAAACACCTTGTTTACGCTTGAGTTTGGTTTGAATGACTTCATGAACTACAACCGTGGCGT
TCCAGAAGTGAAAGCAGATTATGCAGAAGCACTGATTCGTTTGACGGACGCAGGTGCGAAGAACTTCATG
TTGATGACACTGCCAGACGCGACAAAGGCGCCTCAGTTTAAGTACTCAACACAAGAAGAGATCGACAAAA
TTCGTGCGAAAGTGCTTGAGATGAACGAGTTCATCAAGGCACAAGCGATGTACTACAAAGCGCAAGGTTA
CAACATCACGTTGTTTGATACTCACGCCTTGTTCGAGACGCTAACTTCTGCGCCAGAAGAGCACGGTTTC
GTGAACGCGAGTGATCCTTGTTTGGACATCAACCGCTCATCGTCTGTCGATTACATGTACACCCACGCAT
TGCGCTCTGAGTGTGCAGCGTCTGGTGCTGAGAAGTTTGTGTTCTGGGATGTCACGCATCCAACAACAGC
AACTCACCGCTATGTTGCAGAGAAAATGCTAGAAAGTAGCAACAACTTAGCAGAGTACCGTTTCTAA

>S160
ATGATGAAAAAAACAATCACACTATTAACTGCATTACTCCCGCTTGCTTCTGCAGTTGCCGAAGAGCCAA
CCTTATCACCAGAAATGGTTTCAGCGTCTGAAGTGATCAGCACGCAAGAAAACCAAACCTATACCTATGT
TCGCTGTTGGTATCGCACCAGCTACTCGAAAGATGATCCGGCGACCGATTGGGAATGGGCAAAAAACGAA
GATGGTAGCTACTTCACCATTGACGGCTACTGGTGGAGCTCCGTTTCATTTAAAAACATGTTCTACACCA
ACACGTCGCAAAACGTTATCCGTCAGCGTTGTGAAGCAACATTAGATTTGGCGAACGAGAACGCAGACAT
TACGTTCTTCGCCGCTGACAATCGCTTCTCATACAACCACACGATCTGGAGCAACGACGCAGCAATGCAG
CCAGATCAAATCAACAAAGTGGTTGCACTCGGTGACAGCTTGTCTGATACAGGCAACATCTTTAACGCAT
CACAATGGCGCTTCCCTAACCCGAACAGCTGGTTCTTAGGTCACTTCTCCAACGGTTTTGTGTGGACAGA
ATACATTGCCAAAGCGAAGAACCTTCCGCTCTACAACTGGGCAGTTGGCGGCGCGGCTGGTGAGAACCAA
TACATCGCGCTAACAGGGGTTGGTGATCAAGTTTCTTCGTACTTAACCTACGCAAAACTGGCGAAGAACT
ACAAACCAGCAAACACCTTGTTTACGCTTGAGTTTGGTTTGAATGACTTCATGAACTACAACCGTGGCGT
TCCAGAAGTGAAAGCAGATTATGCAGAAGCACTGATTCGTTTGACGGACGCAGGTGCGAAGAACTTCATG
TTGATGACACTGCCAGACGCGACGAAAGCGCCTCAGTTTAAGTACTCAACACAAGAAGAGATCGACAAAA
TTCGTGCGAAAGTGCTTGAGATGAACGAGTTCATCAAGGCACAAGCGATGTACTACAAAGCGCAAGGTTA
CAACATCACGTTGTTTGATACTCACGCCTTGTTCGAGACGCTAACTTCTGCGCCAGAAGAGCACGGTTTC
GTGAACGCGAGTGATCCTTGTTTGGACATCAACCGCTCATCGTCTGTCGATTACATGTACACCCACGCAT
TGCGCTCTGAGTGTGCGGCGTCTGGTGCTGAGAAATTTGTGTTCTGGGATGTCACGCACCCAACAACAGC
AACTCACCGCTATGTTGCAGAGAAAATGCTAGAAAGTAGCAACAACTTAGCCGAGTACCGTTTCTAA

>S159
ATGATGAAAAAAACAATCACACTATTAACTGCATTACTCCCGCTTGCTTCTGCAGTTGCCGAAGAGCCAACCTTAT
CACCAGAAATGGTTTCAGCGTCTGAAGTGATCAGCACGCAAGAAAACCAAACCTATACCTATGTTCGCTGTTGGTA
TCGCACCAGCTACTCGAAAGATGATCCGGCGACCGATTGGGAATGGGCAAAAAACGAAGATGGTAGCTACTTCAC
CATTGACGGCTACTGGTGGAGCTCCGTTTCATTTAAAAACATGTTCTACACCAACACGTCGCAAAACGTTATCCGT
CAGCGTTGTGAAGCAACATTAGATTTGGCGAACGAGAACGCAGACATTACGTTCTTCGCCGCTGACAATCGCTTCT
CATACAACCACACGATCTGGAGCAACGACGCAGCAATGCAGCCAGATCAAATCAACAAAGTGGTTGCACTCGGTG
ACAGCTTGTCTGATACAGGCAACATCTTTAACGCATCACAATGGCGCTTCCCTAACCCGAACAGCTGGTTCTTAGG
TCACTTCTCCAACGGTTTTGTGTGGACAGAATACATTGCCAAAGCGAAGAACCTTCCGCTCTACAACTGGGCAGTT
GGCGGCGCGGCTGGTGAGAACCAATATATCGCGCTAACAGGGGTTGGTGATCAAGTTTCTTCGTACTTAACCTACG
CAAAACTGGCGAAGAACTACAAACCAGCAAACACCTTGTTTACGCTTGAGTTTGGTTTGAATGACTTCATGAACTA
CAACCGTGGCGTTCCAGAAGTGAAAGCAGATTATGCAGAAGCACTGATTCGTTTGACGGACGCAGGTGCGAAGAA
CTTCATGTTGATGACACTGCCAGACGCGACGAAAGCGCCTCAGTTTAAGTACTCAACACAAGAAGAGATCGACAA
AATTCGTGCGAAAGTGCTTGAGATGAACGAGTTCATCAAGGCACAAGCGATGTACTACAAAGCGCAAGGTTACAA
CATCACGTTGTTTGATACTCACGCCTTGTTCGAGACGCTAACTTCTGCGCCCGAAGAGCACGGTTTCGTGAACGCG
AGTGATCCTTGTTTGGACATCAACCGCTCATCGTCTATCGATTACATGTACACCCACGCATTGCGCTCTGAGTGTGC
GGCGTCTGGTGCTGAGAAGTTTGTGTTCTGGGATGTCACGCACCCAACAACAGCAACTCACCGCTATGTTGCAGAG
AAAATGCTAGAAAGTAGCAACAACTTAGCCGAGTACCGTTTCTAA

>S158
ATGATGAAAAAAACAATCACACTATTAACTGCATTACTCCCGCTTGCTTCTGCAGTTGCCGAAGAGCCAACCTTAT
CACCAGAAATGGTTTCAGCGTCTGAAGTGATCAGCACGCAAGAAAACCAAACCTATACCTATGTTCGCTGTTGGTA
TCGCACCAGCTACTCGAAAGATGATCCGGCGACCGATTGGGAATGGGCAAAAAACGAAGATGGTAGCTACTTCAC
CATTGACGGCTACTGGTGGAGCTCCGTTTCATTTAAAAACATGTTCTACACCAACACGTCGCAAAACGTTATCCGT
CAGCGTTGTGAAGCAACATTAGATTTGGCGAACGAGAACGCAGACATTACGTTCTTCGCCGCTGACAATCGCTTCT
CATACAACCACACGATCTGGAGCAACGACGCAGCAATGCAGCCAGATCAAATCAACAAAGTGGTTGCTCTCGGTG
ACAGCTTGTCTGATACAGGCAACATCTTTAACGCATCACAATGGCGCTTCCCTAACCCGAACAGCTGGTTCTTAGG
TCACTTCTCCAACGGTTTTGTGTGGACAGAATACATTGCCAAAGCGAAGAACCTTCCGCTCTACAACTGGGCAGTT
GGCGGCGCGGCTGGTGAGAACCAATACATCGCGCTAACAGGGGTTGGTGATCAAGTTTCTTCGTACTTAACCTACG
CAAAACTGGCGAAGAACTACAAACCAGCAAACACCTTGTTTACGCTTGAGTTTGGTTTGAATGACTTCATGAACTA
CAACCGTGGCGTTCCAGAAGTGAAAGCAGATTATGCAGAAGCACTGATTCGTTTGACGGACGCAGGTGCGAAGAA
CTTCATGTTGATGACACTGCCAGACGCGACAAAGGCGCCTCAGTTTAAGTACTCAACACAAGAAGAGATCGACAA
AATTCGTGCGAAAGTGCTTGAGATGAACGAGTTCATCAAGGCACAAGCGATGTACTACAAAGCGCAAGGTTACAA
CATCACGTTGTTTGATACTCACGCCTTGTTCGAGACGCTAACTTCTGCGCCAGAAGAGCACGGTTTCGTGAACGCG
AGTGATCCTTGTTTGGACATCAACCGCTCATCGTCTGTCGATTACATGTACACCCACGCATTGCGCTCTGAGTGTGC
AGCGTCTGGTGCTGAGAAGTTTGTGTTCTGGGATGTCACGCATCCAACAACAGCAACTCACCGCTATGTTGCAGAG
AAAATGCTAGAAAGTAGCAACAACTTAGCAGAGTACCGTTTCTAA

>S157
ATGATGAAAAAAACAATCACACTATTAACTGCATTACTCCCGCTTGCTTCTGCAGTTGCCGAAGAGCCAACCTTAT
CACCAGAAATGGTTTCAGCGTCTGAAGTGATCAGCACGCAAGAAAACCAAACCTATACCTATGTTCGCTGTTGGTA
TCGCACCAGCTACTCGAAAGATGATCCGGCGACCGATTGGGAATGGGCAAAAAACGAAGATGGTAGCTACTTCAC
CATTGACGGCTACTGGTGGAGCTCCGTTTCATTTAAAAACATGTTCTACACCAACACGTCGCAAAACGTTATCCGT
CAGCGTTGTGAAGCAACATTAGATTTGGCGAACGAGAACGCAGACATTACGTTCTTCGCCGCTGACAATCGCTTCT
CATACAACCACACGATCTGGAGCAACGACGCAGCAATGCAGCCAGATCAAATCAACAAAGTGGTTGCACTCGGTG
ACAGCTTGTCTGATACAGGCAACATCTTTAACGCATCACAATGGCGCTTCCCTAACCCGAACAGCTGGTTCTTAGG
TCACTTCTCCAACGGTTTTGTGTGGACAGAATACATTGCCAAAGCGAAGAACCTTCCGCTCTACAACTGGGCAGTT
GGCGGCGCGGCTGGTGAGAACCAATATATCGCGCTAACAGGGGTTGGTGATCAAGTTTCTTCGTACTTAACCTACG
CAAAACTGGCGAAGAACTACAAACCAGCAAACACCTTGTTTACGCTTGAGTTTGGTTTGAATGACTTCATGAACTA
CAACCGTGGCGTTCCAGAAGTGAAAGCAGATTATGCAGAAGCACTGATTCGTTTGACGGACGCAGGTGCGAAGAA

CTTCATGTTGATGACACTGCCAGACGCGACGAAAGCGCCTCAGTTTAAGTACTCAACACAAGAAGAGATCGACAA
AATTCGTGCGAAAGTGCTTGAGATGAACGAGTTCATCAAGGCACAAGCGATGTACTACAAAGCGCAAGGTTACAA
CATCACGTTGTTTGATACTCACGCCTTGTTCGAGACGCTAACTTCTGCGCCCGAAGAGCACGGTTTCGTGAACGCG
AGTGATCCTTGTTTGGACATCAACCGCTCATCGTCTATCGATTACATGTACACCCACGCATTGCGCTCTGAGTGTGC
GGCGTCTGGTGCTGAGAAGTTTGTGTTCTGGGATGTCACGCACCCAACAACAGCAACTCACCGCTATGTTGCAGAG
AAAATGCTAGAAAGTAGCAACAACTTAGCCGAGTACCGTTTCTAA

>S156
ATGATGAAAAAAACAATCACACTATTAACTGCATTACTCCCGCTTGCTTCTGCAGTTGCCGAAGAGCCAACCTTAT
CACCAGAAATGGTTTCAGCGTCTGAAGTGATCAGCACGCAAGAAAACCAAACCTATACCTATGTTCGCTGTTGGTA
TCGCACCAGCTACTCGAAAGATGATCCGGCGACCGATTGGGAATGGGCAAAAAACGAAGATGGTAGCTACTTCAC
CATTGACGGCTACTGGTGGAGCTCCGTTTCATTTAAAAACATGTTCTACACCAACACGTCGCAAAACGTTATCCGT
CAGCGTTGTGAAGCAACATTAGATTTGGCGAACGAGAACGCAGACATTACGTTCTTCGCCGCTGACAATCGCTTCT
CATACAACCACACGATCTGGAGCAACGACGCAGCAATGCAGCCAGATCAAATCAACAAAGTGGTTGCTCTCGGTG
ACAGCTTGTCTGATACAGGCAACATCTTTAACGCATCACAATGGCGCTTCCCTAACCCGAACAGCTGGTTCTTAGG
TCACTTCTCCAACGGTTTTGTGTGGACAGAATACATTGCCAAAGCGAAGAACCTTCCGCTCTACAACTGGGCAGTT
GGCGGCGCGGCTGGTGAGAACCAATACATCGCGCTAACAGGGGTTGGTGATCAAGTTTCTTCGTACTTAACCTACG
CAAAACTGGCGAAGAACTACAAACCAGCAAACACCTTGTTTACGCTTGAGTTTGGTTTGAATGACTTCATGAACTA
CAACCGTGGCGTTCCAGAAGTGAAAGCAGATTATGCAGAAGCACTGATTCGTTTGACGGACGCAGGTGCGAAGAA
CTTCATGTTGATGACACTGCCAGACGCGACAAAGGCGCCTCAGTTTAAGTACTCAACACAAGAAGAGATCGACAA
AATTCGTGCGAAAGTGCTTGAGATGAACGAGTTCATCAAGGCACAAGCGATGTACTACAAAGCGCAAGGTTACAA
CATCACGTTGTTTGATACTCACGCCTTGTTCGAGACGCTAACTTCTGCGCCAGAAGAGCACGGTTTCGTGAACGCG
AGTGATCCTTGTTTGGACATCAACCGCTCATCGTCTGTCGATTACATGTACACCCACGCATTGCGCTCTGAGTGTGC
AGCGTCTGGTGCTGAGAAGTTTGTGTTCTGGGATGTCACGCATCCAACAACAGCAACTCACCGCTATGTTGCAGAG
AAAATGCTAGAAAGTAGCAACAACTTAGCAGAGTACCGTTTCTAA

>S155
ATGATGAAAAAAACAATCACACTATTAACTGCATTACTCCCGCTTGCTTCTGCAGTTGCCGAAGAGCCAACCTTAT
CACCAGAAATGGTTTCAGCGTCTGAAGTGATCAGCACGCAAGAAAACCAAACCTATACCTATGTTCGCTGTTGGTA
TCGCACCAGCTACTCGAAAGATGATCCGGCGACCGATTGGGAATGGGCAAAAAACGAAGATGGTAGCTACTTCAC
CATTGACGGCTACTGGTGGAGCTCCGTTTCATTTAAAAACATGTTCTACACCAACACGTCGCAAAACGTTATCCGT
CAGCGTTGTGAAGCAACATTAGATTTGGCGAACGAGAACGCAGACATTACGTTCTTCGCCGCTGACAATCGCTTCT
CATACAACCACACGATCTGGAGCAACGACGCAGCAATGCAGCCAGATCAAATCAACAAAGTGGTTGCTCTCGGTG
ACAGCTTGTCTGATACAGGCAACATCTTTAACGCATCACAATGGCGCTTCCCTAACCCGAACAGCTGGTTCTTAGG
TCACTTCTCCAACGGTTTTGTGTGGACAGAATACATTGCCAAAGCGAAGAACCTTCCGCTCTACAACTGGGCAGTT
GGCGGCGCGGCTGGTGAGAACCAATACATCGCGCTAACAGGGGTTGGTGATCAAGTTTCTTCGTACTTAACCTACG
CAAAACTGGCGAAGAACTACAAACCAGCAAACACCTTGTTTACGCTTGAGTTTGGTTTGAATGACTTCATGAACTA
CAACCGTGGCGTTCCAGAAGTGAAAGCAGATTATGCAGAAGCACTGATTCGTTTGACGGACGCAGGTGCGAAGAA
CTTCATGTTGATGACACTGCCAGACGCGACAAAGGCGCCTCAGTTTAAGTACTCAACACAAGAAGAGATCGACAA
AATTCGTGCGAAAGTGCTTGAGATGAACGAGTTCATCAAGGCACAAGCGATGTACTACAAAGCGCAAGGTTACAA
CATCACGTTGTTTGATACTCACGCCTTGTTCGAGACGCTAACTTCTGCGCCAGAAGAGCACGGTTTCGTGAACGCG
AGTGATCCTTGTTTGGACATCAACCGCTCATCGTCTGTCGATTACATGTACACCCACGCATTGCGCTCTGAGTGTGC
AGCGTCTGGTGCTGAGAAGTTTGTGTTCTGGGATGTCACGCATCCAACAACAGCAACTCACCGCTATGTTGCAGAG
AAAATGCTAGAAAGTAGCAACAACTTAGCAGAGTACCGTTTCTAA

>S154
ATGATGAAAAAAACAATCACACTATTAACTGCATTACTCCCGCTTGCTTCTGCAGTTGCCGAAGAGCCAA
CCTTATCACCAGAAATGGTTTCAGCGTCTGAAGTGATCAGCACGCAAGAAAACCAAACCTATACCTATGT
TCGCTGTTGGTATCGCACCAGCTACTCGAAAGATGATCCGGCGACCGATTGGGAATGGGCAAAAAACGAA
GATGGTAGCTACTTCACCATTGACGGCTACTGGTGGAGCTCCGTTTCATTTAAAAACATGTTCTACACCA
ACACGTCGCAAAACGTTATCCGTCAGCGTTGTGAAGCAACATTAGATTTGGCGAACGAGAACGCAGACAT

TACGTTCTTCGCCGCTGACAATCGCTTCTCATACAACCACACGATCTGGAGCAACGACGCAGCAATGCAG
CCAGATCAAATCAACAAAGTGGTTGCTCTCGGTGACAGCTTGTCTGATACAGGCAACATCTTTAACGCAT
CACAATGGCGCTTCCCTAACCCGAACAGCTGGTTCTTAGGTCACTTCTCCAACGGTTTTGTGTGGACAGA
ATACATTGCCAAAGCGAAGAACCTTCCGCTCTACAACTGGGCAGTTGGCGGCGCGGCTGGTGAGAACCAA
TACATCGCGCTAACAGGGGTTGGTGATCAAGTTTCTTCGTACTTAACCTACGCAAAACTGGCGAAGAACT
ACAAACCAGCAAACACCTTGTTTACGCTTGAGTTTGGTTTGAATGACTTCATGAACTACAACCGTGGCGT
TCCAGAAGTGAAAGCAGATTATGCAGAAGCACTGATTCGTTTGACGGACGCAGGTGCGAAGAACTTCATG
TTGATGACACTGCCAGACGCGACAAAGGCGCCTCAGTTTAAGTACTCAACACAAGAAGAGATCGACAAAA
TTCGTGCGAAAGTGCTTGAGATGAACGAGTTCATCAAGGCACAAGCGATGTACTACAAAGCGCAAGGTTA
CAACATCACGTTGTTTGATACTCACGCCTTGTTCGAGACGCTAACTTCTGCGCCAGAAGAGCACGGTTTC
GTGAACGCGAGTGATCCTTGTTTGGACATCAACCGCTCATCGTCTGTCGATTACATGTACACCCACGCAT
TGCGCTCTGAGTGTGCAGCGTCTGGTGCTGAGAAGTTTGTGTTCTGGGATGTCACGCATCCAACAACAGC
AACTCACCGCTATGTTGCAGAGAAAATGCTAGAAAGTAGCAACAACTTAGCAGAGTACCGTTTCTAA

>S153
ATGATGAAAAAAACAATCACACTATTAACTGCATTACTCCCGCTTGCTTCTGCAGTTGCCGAAGAGCCAACCTTAT
CACCAGAAATGGTTTCAGCGTCTGAAGTGATCAGCACGCAAGAAAACCAAACCTATACCTATGTTCGCTGTTGGTA
TCGCACCAGCTACTCGAAAGATGATCCGGCGACCGATTGGGAATGGGCAAAAAACGAAGATGGTAGCTACTTCAC
CATTGACGGCTACTGGTGGAGCTCCGTTTCATTTAAAAACATGTTCTACACCAACACGTCGCAAAACGTTATCCGT
CAGCGTTGTGAAGCAACATTAGATTTGGCGAACGAGAACGCAGACATTACGTTCTTCGCCGCTGACAATCGCTTCT
CATACAACCACACGATCTGGAGCAACGACGCAGCAATGCAGCCAGATCAAATCAACAAAGTGGTTGCTCTCGGTG
ACAGCTTGTCTGATACAGGCAACATCTTTAACGCATCACAATGGCGCTTCCCTAACCCGAACAGCTGGTTCTTAGG
TCACTTCTCCAACGGTTTTGTGTGGACAGAATACATTGCCAAAGCGAAGAACCTTCCGCTCTACAACTGGGCAGTT
GGCGGCGCGGCTGGTGAGAACCAATACATCGCGCTAACAGGGGTTGGTGATCAAGTTTCTTCGTACTTAACCTACG
CAAAACTGGCGAAGAACTACAAACCAGCAAACACCTTGTTTACGCTTGAGTTTGGTTTGAATGACTTCATGAACTA
CAACCGTGGCGTTCCAGAAGTGAAAGCAGATTATGCAGAAGCACTGATTCGTTTGACGGACGCAGGTGCGAAGAA
CTTCATGTTGATGACACTGCCAGACGCGACAAAGGCGCCTCAGTTTAAGTACTCAACACAAGAAGAGATCGACAA
AATTCGTGCGAAAGTGCTTGAGATGAACGAGTTCATCAAGGCACAAGCGATGTACTACAAAGCGCAAGGTTACAA
CATCACGTTGTTTGATACTCACGCCTTGTTCGAGACGCTAACTTCTGCGCCAGAAGAGCACGGTTTCGTGAACGCG
AGTGATCCTTGTTTGGACATCAACCGCTCATCGTCTGTCGATTACATGTACACCCACGCATTGCGCTCTGAGTGTGC
AGCGTCTGGTGCTGAGAAGTTTGTGTTCTGGGATGTCACGCATCCAACAACAGCAACTCACCGCTATGTTGCAGAG
AAAATGCTAGAAAGTAGCAACAACTTAGCAGAGTACCGTTTCTAA

>S152
ATGATGAAAAAAACAATCACACTATTAACTGCATTACTCCCGCTTGCTTCTGCAGTTGCCGAAGAGCCAA
CCTTATCACCAGAAATGGTTTCAGCGTCTGAAGTGATCAGCACGCAAGAAAACCAAACCTATACCTATGT
TCGCTGTTGGTATCGCACCAGCTACTCGAAAGATGATCCGGCGACCGATTGGGAATGGGCAAAAAACGAA
GATGGTAGCTACTTCACCATTGACGGCTACTGGTGGAGCTCCGTTTCATTTAAAAACATGTTCTACACCA
ACACGTCGCAAAACGTTATCCGTCAGCGTTGTGAAGCAACATTAGATTTGGCGAACGAGAACGCAGACAT
TACGTTCTTCGCCGCTGACAATCGCTTCTCATACAACCACACGATCTGGAGCAACGACGCAGCAATGCAG
CCAGATCAAATCAACAAAGTGGTTGCACTCGGTGACAGCTTGTCTGATACAGGCAACATCTTTAACGCAT
CACAATGGCGCTTCCCTAACCCGAACAGCTGGTTCTTAGGTCACTTCTCCAACGGTTTTGTGTGGACAGA
ATACATTGCCAAAGCGAAGAACCTTCCGCTCTACAACTGGGCAGTTGGCGGTGCGGCTGGTGAGAACCAA
TACATCGCGCTAACAGGGGTTGGTGATCAAGTTTCTTCGTACTTAACCTACGCAAAACTGGCGAAGAACT
ACAAACCAGCAAACACCTTGTTTACGCTTGAGTTTGGTTTGAATGACTTCATGAACTACAACCGTGGCGT
TCCAGAAGTGAAAGCGGATTATGCAGAAGCACTGATTCGTTTGACGGACGCAGGTGCGAAGAACTTCATG
TTGATGACACTGCCAGACGCGACGAAAGCGCCTCAGTTTAAGTACTCAACACAAGAAGAGATCGACAAGA
TTCGTGCGAAAGTGCTTGAGATGAACGAGTTCATCAAGGCACAAGCGATGTACTACAAAGCGCAAGGTTA
CAACATCACGTTGTTTGATACTCACGCCTTGTTCGAGACGCTAACTTCTGCGCCAGAAGAGCACGGTTTC
GTGAACGCGAGCGATCCTTGTTTGGACATCAACCGCTCATCGTCTGTCGATTACATGTACACCCACGCAT
TGCGCTCTGAGTGTGCAGCGTCTGGTGCTGAGAAGTTTGTGTTCTGGGATGTCACGCATCCAACAACAGC

AACTCACCGCTATGTTGCAGAGAAAATGCTAGAAAGTAGCAACAACTTAGCAGAGTACCGTTTCTAA

>S151
ATGATGAAAAAAACAATCACACTATTAACTGCATTACTCCCGCTTGCTTCTGCAGTTGCCGAAGAGCCAA
CCTTATCACCAGAAATGGTTTCAGCGTCTGAAGTGATCAGCACGCAAGAAAACCAAACCTATACCTATGT
TCGCTGTTGGTATCGCACCAGCTACTCGAAAGATGATCCGGCGACCGATTGGGAATGGGCAAAAAACGAA
GATGGTAGCTACTTCACCATTGACGGCTACTGGTGGAGCTCCGTTTCATTTAAAAACATGTTCTACACCA
ACACGTCGCAAAACGTTATCCGTCAGCGTTGTGAAGCAACATTAGATTTGGCGAACGAGAACGCAGACAT
TACGTTCTTCGCCGCTGACAATCGCTTCTCATACAACCACACGATCTGGAGCAACGACGCAGCAATGCAG
CCAGATCAAATCAACAAAGTGGTTGCTCTCGGTGACAGCTTGTCTGATACAGGCAACATCTTTAACGCAT
CACAATGGCGCTTCCCTAACCCGAACAGCTGGTTCTTAGGTCACTTCTCCAACGGTTTTGTGTGGACAGA
ATACATTGCCAAAGCGAAGAACCTTCCGCTCTACAACTGGGCAGTTGGCGGCGCGGCTGGTGAGAACCAA
TACATCGCGCTAACAGGGGTTGGTGATCAAGTTTCTTCGTACTTAACCTACGCAAAACTGGCGAAGAACT
ACAAACCAGCAAACACCTTGTTTACGCTTGAGTTTGGTTTGAATGACTTCATGAACTACAACCGTGGCGT
TCCAGAAGTGAAAGCAGATTATGCAGAAGCACTGATTCGTTTGACGGACGCAGGTGCGAAGAACTTCATG
TTGATGACACTGCCAGACGCGACAAAGGCGCCTCAGTTTAAGTACTCAACACAAGAAGAGATCGACAAAA
TTCGTGCGAAAGTGCTTGAGATGAACGAGTTCATCAAGGCACAAGCGATGTACTACAAAGCGCAAGGTTA
CAACATCACGTTGTTTGATACTCACGCCTTGTTCGAGACGCTAACTTCTGCGCCAGAAGAGCACGGTTTC
GTGAACGCGAGTGATCCTTGTTTGGACATCAACCGCTCATCGTCTGTCGATTACATGTACACCCACGCAT
TGCGCTCTGAGTGTGCAGCGTCTGGTGCTGAGAAGTTTGTGTTCTGGGATGTCACGCATCCAACAACAGC
AACTCACCGCTATGTTGCAGAGAAAATGCTAGAAAGTAGCAACAACTTAGCAGAGTACCGTTTCTAA

>S150
ATGATGAAAAAAACAATCACACTATTAACTGCATTACTCCCGCTTGCTTCTGCAGTTGCCGAAGAGCCAACCTTAT
CACCAGAAATGGTTTCAGCGTCTGAAGTGATCAGCACGCAAGAAAACCAAACCTATACCTATGTTCGCTGTTGGTA
TCGCACCAGCTACTCGAAAGATGATCCGGCGACCGATTGGGAATGGGCAAAAAACGAAGATGGTAGCTACTTCAC
CATTGACGGCTACTGGTGGAGCTCCGTTTCATTTAAAAACATGTTCTACACCAACACGTCGCAAAACGTTATCCGT
CAGCGTTGTGAAGCAACATTAGATTTGGCGAACGAGAACGCAGACATTACGTTCTTCGCCGCTGACAATCGCTTCT
CATACAACCACACGATCTGGAGCAACGACGCAGCAATGCAGCCAGATCAAATCAACAAAGTGGTTGCTCTCGGTG
ACAGCTTGTCTGATACAGGCAACATCTTTAACGCATCACAATGGCGCTTCCCTAACCCGAACAGCTGGTTCTTAGG
TCACTTCTCCAACGGTTTTGTGTGGACAGAATACATTGCCAAAGCGAAGAACCTTCCGCTCTACAACTGGGCAGTT
GGCGGCGCGGCTGGTGAGAACCAATACATCGCGCTAACAGGGGTTGGTGATCAAGTTTCTTCGTACTTAACCTACG
CAAAACTGGCGAAGAACTACAAACCAGCAAACACCTTGTTTACGCTTGAGTTTGGTTTGAATGACTTCATGAACTA
CAACCGTGGCGTTCCAGAAGTGAAAGCAGATTATGCAGAAGCACTGATTCGTTTGACGGACGCAGGTGCGAAGAA
CTTCATGTTGATGACACTGCCAGACGCGACAAAGGCGCCTCAGTTTAAGTACTCAACACAAGAAGAGATCGACAA
AATTCGTGCGAAAGTGCTTGAGATGAACGAGTTCATCAAGGCACAAGCGATGTACTACAAAGCGCAAGGTTACAA
CATCACGTTGTTTGATACTCACGCCTTGTTCGAGACGCTAACTTCTGCGCCAGAAGAGCACGGTTTCGTGAACGCG
AGTGATCCTTGTTTGGACATCAACCGCTCATCGTCTGTCGATTACATGTACACCCACGCATTGCGCTCTGAGTGTGC
AGCGTCTGGTGCTGAGAAGTTTGTGTTCTGGGATGTCACGCATCCAACAACAGCAACTCACCGCTATGTTGCAGAG
AAAATGCTAGAAAGTAGCAACAACTTAGCAGAGTACCGTTTCTAA

>S148
ATGATGAAAAAAACAATCACACTATTAACTGCATTACTCCCGCTTGCTTCTGCAGTTGCCGAAGAGCCAA
CCTTATCACCAGAAATGGTTTCAGCGTCTGAAGTGATCAGCACGCAAGAAAACCAAACCTATACCTATGT
TCGCTGTTGGTATCGCACCAGCTACTCGAAAGATGATCCGGCGACCGATTGGGAATGGGCAAAAAACGAA
GATGGTAGCTACTTCACCATTGACGGCTACTGGTGGAGCTCCGTTTCATTTAAAAACATGTTCTACACCA
ACACGTCGCAAAACGTTATCCGTCAGCGTTGTGAAGCAACATTAGATTTGGCGAACGAGAACGCAGACAT
TACGTTCTTCGCCGCTGACAATCGCTTCTCATACAACCACACGATCTGGAGCAACGACGCAGCAATGCAG
CCAGATCAAATCAACAAAGTGGTTGCACTCGGTGACAGCTTGTCTGATACAGGCAACATCTTTAACGCAT
CACAATGGCGCTTCCCTAACCCGAACAGCTGGTTCTTAGGTCACTTCTCCAACGGTTTTGTGTGGACAGA
ATACATTGCCAAAGCGAAGAACCTTCCGCTCTACAACTGGGCAGTTGGCGGCGCGGCTGGTGAGAACCAA

TACATCGCGCTAACAGGGGTTGGTGATCAAGTTTCTTCGTACTTAACCTACGCAAAACTGGCGAAGAACT
ACAAACCAGCAAACACCTTGTTTACGCTTGAGTTTGGTTTGAATGACTTCATGAACTACAACCGTGGCGT
TCCAGAAGTGAAAGCGGATTATGCAGAAGCACTGATTCGTTTGACGGACGCAGGTGCGAAGAACTTCATG
TTGATGACACTGCCAGACGCGACGAAAGCGCCTCAGTTTAAGTACTCAACACAAGAAGAGATCGACAAAA
TTCGTGCGAAAGTGCTTGAGATGAACGAGTTCATCAAGGCACAAGCGATGTACTACAAAGCGCAAGGTTA
CAACATCACGTTGTTTGATACTCACGCCTTGTTCGAGACGCTAACTTCTGCGCCAGAAGAGCACGGTTTC
GTGAACGCGAGCGATCCTTGTTTGGACATCAACCGCTCATCGTCTGTCGATTACATGTACACCCACGCAT
TGCGTTCTGAGTGTGCAGCGTCTGGTGCTGAGAAGTTTGTGTTCTGGGATGTCACGCATCCAACAACAGC
AACTCACCGCTATGTTGCAGAGAAAATGCTAGAAAGTAGTAACAACTTAGCCGAGTACCGTTTCTAA

>S147
ATGATGAAAAAAACAATCACACTATTAACTGCATTACTCCCGCTTGCTTCTGCAGTTGCCGAAGAGCCAA
CCTTATCACCAGAAATGGTTTCAGCGTCTGAAGTGATCAGCACGCAAGAAAACCAAACCTATACCTATGT
TCGCTGTTGGTATCGCACCAGCTACTCGAAAGATGATCCAGCGACCGATTGGGAATGGGCAAAAAACGAA
GATGGTAGCTACTTCACCATTGACGGCTACTGGTGGAGCTCCGTTTCATTTAAAAACATGTTCTACACCA
ACACGTCGCAAAACGTTATCCGTCAGCGTTGTGAAGCAACATTAGATTTGGCGAACGAGAACGCAGACAT
TACGTTCTTCGCCGCTGACAATCGCTTCTCATACAACCACACGATCTGGAGCAACGACGCAGCAATGCAG
CCAGATCAAATCAACAAAGTGGTTGCACTCGGTGACAGCTTGTCTGATACAGGCAACATCTTTAACGCAT
CACAATGGCGCTTCCCTAACCCGAACAGCTGGTTCTTAGGTCACTTCTCCAACGGTTTTGTGTGGACAGA
ATACATTGCCAAAGCGAAGAACCTTCCGCTCTACAACTGGGCAGTTGGCGGCGCGGCTGGTGAGAACCAA
TACATCGCGCTAACAGGGGTTGGTGACCAAGTTTCTTCGTACTTAACCTACGCAAAACTGGCGAAGAACT
ACAAACCAGCAAACACCTTGTTTACGCTTGAGTTTGGTTTGAATGACTTCATGAACTACAACCGTGGCGT
TCCAGAAGTGAAAGCAGATTATGCAGAAGCACTGATTCGTTTGACGGACGCAGGTGCGAAGAACTTCATG
TTGATGACACTGCCAGACGCGACGAAAGCGCCTCAGTTTAAGTACTCAACACAAGAAGAGATCGACAAAA
TTCGTGCGAAAGTGCTTGAGATGAACGAGTTCATCAAGGCACAAGCGATGTACTACAAAGCGCAAGGTTA
CAACATCACGTTGTTTGATACTCACGCCTTGTTCGAGACGCTAACTTCTGCGCCCGAAGAGCACGGTTTC
GTGAACGCGAGCGATCCTTGTTTGGACATCAACCGCTCATCGTCTGTCGATTACATGTACACCCACGCAT
TGCGCTCTGAGTGTGCAGCGTCTGGTGCTGAGAAGTTTGTGTTCTGGGATGTCACGCACCCAACAACAGC
AACTCACCGCTATGTTGCAGAGAAAATGCTAGAAAGTAGCAACAACTTAGCAGAGTACCGTTTCTAA

>S146
ATGATGAAAAAAACAATCACACTATTAACTGCATTACTCCCGCTTGCTTCTGCAGTTGCCGAAGAGCCAA
CCTTATCACCAGAAATGGTTTCAGCGTCTGAAGTGATCAGCACGCAAGAAAACCAAACCTATACCTATGT
TCGCTGTTGGTATCGCACCAGCTACTCGAAAGATGATCCGGCGACCGATTGGGAATGGGCAAAAAACGAA
GATGGTAGCTACTTCACCATTGACGGCTACTGGTGGAGCTCCGTTTCATTTAAAAACATGTTCTACACCA
ACACGTCGCAAAACGTTATCCGTCAGCGTTGTGAAGCAACATTAGATTTGGCGAACGAGAACGCAGACAT
TACGTTCTTCGCCGCTGACAATCGCTTCTCATACAACCACACGATCTGGAGCAACGACGCAGCAATGCAG
CCAGATCAAATCAACAAAGTGGTTGCACTCGGTGACAGCTTGTCTGATACAGGCAACATCTTTAACGCAT
CACAATGGCGCTTCCCTAACCCGAACAGCTGGTTCTTAGGTCACTTCTCCAACGGTTTTGTGTGGACAGA
ATACATTGCCAAAGCGAAGAACCTTCCGCTCTACAACTGGGCAGTTGGCGGCGCGGCTGGTGAGAACCAA
TACATCGCGCTAACAGGGGTTGGTGATCAAGTTTCTTCGTACTTAACCTACGCAAAACTGGCGAAGAACT
ACAAACCAGCAAACACCTTGTTTACGCTTGAGTTTGGTTTGAATGACTTCATGAACTACAACCGTGGCGT
TCCAGAAGTGAAAGCAGATTATGCAGAAGCACTGATTCGTTTGACGGACGCAGGTGCGAAGAACTTCATG
TTGATGACATTGCCAGACGCGACGAAAGCGCCTCAGTTTAAGTACTCAACACAAGAAGAGATCGACAAAA
TTCGTGCGAAAGTGCTTGAGATGAACGAGTTCATCAAGGCACAAGCGATGTACTACAAAGCGCAAGGTTA
CAACATCACGTTGTTTGATACTCACGCCTTGTTCGAGACGCTAACTTCTGCGCCCGAAGAGCACGGTTTC
GTGAACGCGAGTGATCCTTGTTTGGACATCAACCGCTCATCGTCTGTCGATTACATGTACACCCACGCAT
TGCGCTCTGAGTGTGCGGCGTCTGGTGCTGAGAAGTTTGTGTTCTGGGATGTCACGCATCCAACAACAGC
AACTCACCGCTATGTTGCAGAGAAAATGCTAGAAAGTAGCAACAACTTAGCCGAGTACCGTTTCTAA

>S145

ATGATGAAAAAAACAATCACACTATTAACTGCATTACTCCCGCTTGCTTCTGCAGTTGCCGAAGAGCCAACCTTAT
CACCAGAAATGGTTTCAGCGTCTGAAGTGATCAGCACGCAAGAAAACCAAACCTATACCTATGTTCGCTGTTGGTA
TCGCACCAGCTACTCGAAAGATGATCCGGCGACCGATTGGGAATGGGCAAAAAACGAAGATGGTAGCTACTTCAC
CATTGACGGCTACTGGTGGAGCTCCGTTTCATTTAAAAACATGTTCTACACCAACACGTCGCAAAACGTTATCCGT
CAGCGTTGTGAAGCAACATTAGATTTGGCGAACGAGAACGCAGACATTACGTTCTTCGCCGCTGACAATCGCTTCT
CATACAACCACACGATCTGGAGCAACGACGCAGCAATGCAGCCAGATCAAATCAACAAAGTGGTTGCACTCGGTG
ACAGCTTGTCTGATACAGGCAACATCTTTAACGCATCACAATGGCGCTTCCCTAACCCGAACAGCTGGTTCTTAGG
TCACTTCTCCAACGGTTTTGTGTGGACAGAATACATTGCCAAAGCGAAGAACCTTCCGCTCTACAACTGGGCAGTT
GGCGGCGCGGCTGGTGAGAACCAATACATCGCGCTAACAGGAGTTGGTGATCAAGTTTCTTCGTACTTAACCTACG
CAAAACTGGCGAAGAACTACAAACCAGCAAACACCTTGTTTACGCTTGAGTTTGGTTTGAATGACTTCATGAACTA
CAACCGTGGCGTTCCAGAAGTGAAAGCGGATTATGCAGAAGCACTGATTCGTTTGACGGACGCAGGTGCGAAGAA
CTTCATGTTGATGACACTGCCAGATGCGACGAAAGCGCCTCAGTTTAAGTACTCAACACAAGAAGAGATCGACAA
AATTCGTGCGAAAGTGCTTGAGATAAACGAGTTCATCAAGGCACAAGCGATGTACTACAAAGCGCAAGGTTACAA
CATCACGTTGTTTGATACTCACGCCTTGTTCGAGACGCTAACTTCTGCGCCAGAAGAGCACGGTTTCGTGAACGCG
AGTGATCCTTGTTTGGACATCAACCGCTCATCGTCTGTCGATTACATGTACACCCACGCATTGCGCTCTGAGTGTGC
AGCGTCTGGTGCTGAGAAGTTTGTGTTCTGGGATGTCACGCATCCAACAACAGCAACTCACCGCTATGTTGCAGAG
AAAATGCTAGAAAGTAGCAACAACTTAGCAGAGTACCGTTTCTAA

>S144
ATGATGAAAAAAACAATCACACTATTAACTGCATTACTCCCGCTTGCTTCTGCAGTTGCCGAAGAGCCAACCTTAT
CACCAGAAATGGTTTCAGCGTCTGAAGTGATCAGCACGCAAGAAAACCAAACCTATACCTATGTTCGCTGTTGGTA
TCGCACCAGCTACTCGAAAGATGATCCGGCGACCGATTGGGAATGGGCAAAAAACGAAGATGGTAGCTACTTCAC
CATTGACGGCTACTGGTGGAGCTCCGTTTCATTTAAAAACATGTTCTACACCAACACGTCGCAAAACGTTATCCGT
CAGCGTTGTGAAGCAACATTAGATTTGGCGAACGAGAACGCAGACATTACGTTCTTCGCCGCTGACAATCGCTTCT
CATACAACCACACGATCTGGAGCAACGACGCAGCAATGCAGCCAGATCAAATCAACAAAGTGGTTGCTCTCGGTG
ACAGCTTGTCTGATACAGGCAACATCTTTAACGCATCACAATGGCGCTTCCCTAACCCGAACAGCTGGTTCTTAGG
TCACTTCTCCAACGGTTTTGTGTGGACAGAATACATTGCCAAAGCGAAGAACCTTCCGCTCTACAACTGGGCAGTT
GGCGGCGCGGCTGGTGAGAACCAATACATCGCGCTAACAGGGGTTGGTGATCAAGTTTCTTCGTACTTAACCTACG
CAAAACTGGCGAAGAACTACAAACCAGCAAACACCTTGTTTACGCTTGAGTTTGGTTTGAATGACTTCATGAACTA
CAACCGTGGCGTTCCAGAAGTGAAAGCAGATTATGCAGAAGCACTGATTCGTTTGACGGACGCAGGTGCGAAGAA
CTTCATGTTGATGACACTGCCAGACGCGACAAAGGCGCCTCAGTTTAAGTACTCAACACAAGAAGAGATCGACAA
AATTCGTGCGAAAGTGCTTGAGATGAACGAGTTCATCAAGGCACAAGCGATGTACTACAAAGCGCAAGGTTACAA
CATCACGTTGTTTGATACTCACGCCTTGTTCGAGACGCTAACTTCTGCGCCAGAAGAGCACGGTTTCGTGAACGCG
AGTGATCCTTGTTTGGACATCAACCGCTCATCGTCTGTCGATTACATGTACACCCACGCATTGCGCTCTGAGTGTGC
AGCGTCTGGTGCTGAGAAGTTTGTGTTCTGGGATGTCACGCATCCAACAACAGCAACTCACCGCTATGTTGCAGAG
AAAATGCTAGAAAGTAGCAACAACTTAGCAGAGTACCGTTTCTAA

>S143
ATGATGAAAAAAACAATCACACTATTAACTGCATTACTCCCGCTTGCTTCTGCAGTTGCCGAAGAGCCAA
CCTTATCACCAGAAATGGTTTCAGCGTCTGAAGTGATCAGCACGCAAGAAAACCAAACCTATACCTATGT
TCGCTGTTGGTATCGCACCAGCTACTCGAAAGATGATCCGGCGACCGATTGGGAATGGGCAAAAAACGAA
GATGGTAGCTACTTCACCATTGACGGCTACTGGTGGAGCTCCGTTTCATTTAAAAACATGTTCTACACCA
ACACGTCGCAAAACGTTATCCGTCAGCGTTGTGAAGCAACATTAGATTTGGCGAACGAGAACGCAGACAT
TACGTTCTTCGCCGCTGACAATCGCTTCTCATACAACCACACGATCTGGAGCAACGACGCAGCAATGCAG
CCAGATCAAATCAACAAAGTGGTTGCTCTCGGTGACAGCTTGTCTGATACAGGCAACATCTTTAACGCAT
CACAATGGCGCTTCCCTAACCCGAACAGCTGGTTCTTAGGTCACTTCTCCAACGGTTTTGTGTGGACAGA
ATACATTGCCAAAGCGAAGAACCTTCCGCTCTACAACTGGGCAGTTGGCGGCGCGGCTGGTGAGAACCAA
TACATCGCGCTAACAGGGGTTGGTGATCAAGTTTCTTCGTACTTAACCTACGCAAAACTGGCGAAGAACT
ACAAACCAGCAAACACCTTGTTTACGCTTGAGTTTGGTTTGAATGACTTCATGAACTACAACCGTGGCGT
TCCAGAAGTGAAAGCAGATTATGCAGAAGCACTGATTCGTTTGACGGACGCAGGTGCGAAGAACTTCATG

TTGATGACACTGCCAGACGCGACAAAGGCGCCTCAGTTTAAGTACTCAACACAAGAAGAGATCGACAAAA
TTCGTGCGAAAGTGCTTGAGATGAACGAGTTCATCAAGGCACAAGCGATGTACTACAAAGCGCAAGGTTA
CAACATCACGTTGTTTGATACTCACGCCTTGTTCGAGACGCTAACTTCTGCGCCAGAAGAGCACGGTTTC
GTGAACGCGAGTGATCCTTGTTTGGACATCAACCGCTCATCGTCTGTCGATTACATGTACACCCACGCAT
TGCGCTCTGAGTGTGCAGCGTCTGGTGCTGAGAAGTTTGTGTTCTGGGATGTCACGCATCCAACAACAGC
AACTCACCGCTATGTTGCAGAGAAAATGCTAGAAAGTAGCAACAACTTAGCAGAGTACCGTTTCTAA

>S142
ATGATGAAAAAAACAATCACACTATTAACTGCATTACTCCCGCTTGCTTCTGCAGTTGCCGAAGAGCCAACCTTAT
CACCAGAAATGGTTTCAGCGTCTGAAGTGATCAGCACGCAAGAAAACCAAACCTATACCTATGTTCGCTGTTGGTA
TCGCACCAGCTACTCGAAAGATGATCCGGCGACCGATTGGGAATGGGCAAAAAACGAAGATGGTAGCTACTTCAC
CATTGACGGCTACTGGTGGAGCTCCGTTTCATTTAAAAACATGTTCTACACCAACACGTCGCAAAACGTTATCCGT
CAGCGTTGTGAAGCAACATTAGATTTGGCGAACGAGAACGCAGACATTACGTTCTTCGCCGCTGACAATCGCTTCT
CATACAACCACACGATCTGGAGCAACGACGCAGCAATGCAGCCAGATCAAATCAACAAAGTGGTTGCACTCGGTG
ACAGCTTGTCTGATACAGGCAACATCTTTAACGCATCACAATGGCGCTTCCCTAACCCGAACAGCTGGTTCTTAGG
TCACTTCTCCAACGGTTTTGTGTGGACAGAATACATTGCCAAAGCGAAGAACCTTCCGCTCTACAACTGGGCAGTT
GGCGGCGCGGCTGGTGAGAACCAATACATCGCGCTAACAGGGGTTGGTGATCAAGTTTCTTCGTACTTAACCTACG
CAAAACTGGCGAAGAACTACAAACCAGCAAACACCTTGTTTACGCTTGAGTTTGGTTTGAATGACTTCATGAACTA
CAACCGTGGCGTTCCAGAAGTGAAAGCGGATTATGCAGAAGCACTGATTCGTTTGACGGACGCAGGTGCGAAGAA
CTTCATGTTGATGACACTGCCAGACGCGACGAAAGCGCCTCAGTTTAAGTACTCAACACAAGAAGAGATCGACAA
AATTCGTGCAAAAGTGCTTGAGATGAACGAGTTCATCAAGGCACAAGCGATGTACTACAAAGCGCAAGGTTACAA
CATCACGTTGTTTGATACTCACGCCTTGTTCGAGACGCTAACTTCTGCGCCCGAAGAGCACGGTTTCGTGAACGCG
AGTGATCCTTGTTTGGACATCAACCGCTCATCGTCTGTCGATTACATGTACACCCACGCATTGCGCTCTGAGTGTGC
AGCGTCTGGTGCTGAGAAGTTTGTGTTCTGGGATGTCACGCATCCAACAACAGCAACTCACCGCTATGTTGCAGAG
AAAATGCTAGAAAGTAGCAACAACTTAGCAGAGTACCGTTTCTAA

>S141
ATGATGAAAAAAACAATCACACTATTAACTGCATTACTCCCGCTTGCTTCTGCAGTTGCCGAAGAGCCAA
CCTTATCACCAGAAATGGTTTCAGCGTCTGAAGTGATCAGCACGCAAGAAAACCAAACCTATACCTATGT
TCGCTGTTGGTATCGCACCAGCTACTCGAAAGATGATCCGGCGACCGATTGGGAATGGGCAAAAAACGAA
GATGGTAGCTACTTCACCATTGACGGCTACTGGTGGAGCTCCGTTTCATTTAAAAACATGTTCTACACCA
ACACGTCGCAAAACGTTATCCGTCAGCGTTGTGAAGCAACATTAGATTTGGCGAACGAGAACGCAGACAT
TACGTTCTTCGCCGCTGACAATCGCTTCTCATACAACCACACGATCTGGAGCAACGACGCAGCAATGCAG
CCAGATCAAATCAACAAAGTGGTTGCACTCGGTGACAGCTTGTCTGATACAGGCAACATCTTTAACGCAT
CACAATGGCGCTTCCCTAACCCGAACAGCTGGTTCTTAGGTCACTTCTCCAACGGTTTTGTGTGGACAGA
ATACATTGCCAAAGCGAAGAACCTTCCGCTCTACAACTGGGCAGTTGGCGGCGCGGCTGGTGAGAACCAA
TACATCGCGCTAACAGGGGTTGGTGATCAAGTTTCTTCGTACTTAACCTACGCAAAACTGGCGAAGAACT
ACAAACCAGCAAACACCTTGTTTACGCTTGAGTTTGGTTTGAATGACTTCATGAACTACAACCGTGGCGT
TCCAGAAGTGAAAGCAGATTATGCAGAAGCACTGATTCGTTTGACGGACGCAGGTGCGAAGAACTTCATG
TTGATGACACTGCCAGACGCGACGAAAGCGCCTCAGTTTAAGTACTCAACACAAGAAGAGATCGACAAAA
TTCGTGCGAAAGTGCTTGAGATGAACGAGTTCATCAAGGCACAAGCGATGTACTACAAAGCGCAAGGTTA
CAACATCACGTTGTTTGATACTCACGCCTTGTTCGAGACGCTAACTTCTGCGCCAAAAGAGCACGGTTTC
GTGAACGCGAGCGATCCTTGTTTGGACATCAACCGCTCATCGTCTGTCGATTACATGTACACCCACGCAT
TGCGCTCTGAGTGTGCAGCGTCTGGTGCTGAGAAGTTTGTGTTCTGGGATGTCACGCATCCAACAACAGC
AACTCACCGCTATGTTGCAGAGAAAATGCTAGAAAGTAGCAACAACTTAGCCGAGTACCGTTTCTAA

>S140
ATGATGAAAAAAACAATCACACTATTAACTGCATTACTCCCGCTTGCTTCTGCAGTTGCCGAAGAGCCAACCTTAT
CACCAGAAATGGTTTCAGCGTCTGAAGTGATCAGCACGCAAGAAAACCAAACCTATACCTATGTTCGCTGTTGGTA
TCGCACCAGCTACTCGAAAGATGATCCGGCGACCGATTGGGAATGGGCAAAAAACGAAGATGGTAGCTACTTCAC
CATTGACGGCTACTGGTGGAGCTCCGTTTCATTTAAAAACATGTTCTACACCAACACGTCGCAAAACGTTATCCGT

CAGCGTTGTGAAGCAACATTAGATTTGGCGAACGAGAACGCAGACATTACGTTCTTCGCCGCTGACAATCGCTTCT
CATACAACCACACGATCTGGAGCAACGACGCAGCAATGCAGCCAGATCAAATCAACAAAGTGGTTGCACTCGGTG
ACAGCTTGTCTGATACAGGCAACATCTTTAACGCATCACAATGGCGCTTCCCTAACCCGAACAGCTGGTTCTTAGG
TCACTTCTCCAACGGTTTTGTGTGGACAGAATACATTGCCAAAGCGAAGAACCTTCCGCTCTACAACTGGGCAGTT
GGCGGTGCGGCTGGTGAGAACCAATACATCGCGCTAACAGGGGTTGGTGAGCAAGTTTCTTCGTACTTAACCTACG
CAAAACTGGCGAAGAACTACAAACCAGCAAACACCTTGTTTACGCTTGAGTTTGGTTTGAATGACTTCATGAACTA
CAACCGTGGCGTTCCAGAAGTGAAAGCAGATTATGCAGAAGCACTGATTCGTTTGACGGACGCAGGTGCGAAGAA
CTTCATGTTGATGACACTGCCAGACGCGACGAAAGCGCCTCAGTTTAAGTACTCAACACAAGAAGAGATCGACAA
AATTCGTGCGAAAGTGCTTGAGATGAACGAGTTCATCAAGGCACAAGCGATGTACTACAAAGCGCAAGGTTACAA
CATCACGTTGTTTGATACTCACGCCTTGTTCGAGACGCTAACTTCTGCGCCAGAAGAGCACGGTTTCGTGAACGCG
AGCGATCCTTGTTTGGACATCAACCGCTCATCGTCTGTCGATTACATGTACACCCACGCATTGCGCTCTGAGTGTGC
AGCGTCTGGTGCTGAGAAATTTGTGTTCTGGGATGTCACGCACCCAACAACAGCAACTCACCGCTATGTTGCAGAG
AAAATGCTAGAAAGTAGCAACAACTTAGCCGAGTACCGTTTCTAA

>S139
ATGATGAAAAAAACAATCACACTATTAACTGCATTACTCCCGCTTGCTTCTGCAGTTGCCGAAGAGCCAA
CCTTATCACCAGAAATGGTTTCAGCGTCTGAAGTGATCAGCACGCAAGAAAACCAAACCTATACCTATGT
TCGCTGTTGGTATCGCACCAGCTACTCGAAAGATGATCCGGCGACCGATTGGGAATGGGCAAAAAACGAA
GATGGTAGCTACTTCACCATTGACGGCTACTGGTGGAGCTCCGTTTCATTTAAAAACATGTTCTACACCA
ACACGTCGCAAAACGTTATCCGTCAGCGTTGTGAAGCCACATTAGATTTGGCGAACGAGAACGCAGACAT
TACGTTCTTCGCCGCTGACAATCGCTTCTCATACAACCACACGATCTGGAGCAACGACGCAGCAATGCAG
CCAGATCAAATCAACAAAGTGGTTGCACTCGGTGACAGCTTGTCTGATACAGGCAACATCTTTAACGCAT
CACAATGGCGCTTCCCTAACCCGAACAGCTGGTTCTTAGGTCACTTCTCCAACGGTTTTGTGTGGACAGA
ATACATTGCCAAAGCGAAGAACCTTCCGCTCTACAACTGGGCAGTTGGCGGCGCGGCTGGTGAGAACCAA
TACATCGCGCTAACAGGGGTTGGTGATCAAGTTTCTTCGTACTTAACCTACGCAAAACTGGCGAAGAACT
ACAAACCAGCAAACACCTTGTTTACGCTTGAGTTTGGTTTGAATGACTTCATGAACTACAACCGTGGCGT
TCCAGAAGTGAAAGCGGATTATGCAGAAGCACTGATTCGTTTGACGGACGCAGGTGCGAAGAACTTCATG
TTGATGACACTGCCAGACGCGACGAAAGCGCCTCAGTTTAAGTACTCAACACAAGAAGAGATCGACAAAA
TTCGTGCGAAAGTGCTTGAGATGAACGAGTTCATCAAGGCACAAGCGATGTACTACAAAGCGCAAGGTTA
CAACATCACGTTGTTTGATACTCACGCCTTGTTCGAGACGCTAACTTCTGCGCCCGAAGAGCACGGTTTC
GTGAACGCGAGTGATCCTTGTTTGGACATCAACCGCTCATCGTCTGTCGATTACATGTACACCCACGCAT
TGCGCTCTGAGTGTGCGGCGTCTGGTGCTGAGAAGTTTGTGTTCTGGGATGTCACGCACCCAACAACAGC
AACTCACCGCTATGTTGCAGAGAAAATGCTAGAAAGTAGCAACAACTTAGCCGAGTACCGTTTCTAA

>S138
ATGATGAAAAAAACAATCACACTATTAACTGCATTACTCCCGCTTGCTTCTGCAGTTGCCGAAGAGCCAA
CCTTATCACCAGAAATGGTTTCAGCGTCTGAAGTGATCAGCACGCAAGAAAACCAAACCTATACCTATGT
TCGCTGTTGGTATCGCACCAGCTACTCGAAAGATGATCCAGCGACCGATTGGGAATGGGCAAAAAACGAA
GATGGTAGCTACTTCACCATTGACGGCTACTGGTGGAGCTCCGTTTCATTTAAAAACATGTTCTACACCA
ACACGTCGCAAAACGTTATCCGTCAGCGTTGTGAAGCAACATTAGATTTGGCGAACGAGAACGCAGACAT
TACGTTCTTCGCCGCTGACAATCGCTTCTCATACAACCACACGATCTGGAGCAACGACGCAGCAATGCAG
CCAGATCAAATCAACAAAGTGGTTGCACTCGGTGACAGCTTGTCTGATACAGGCAACATCTTTAACGCAT
CACAATGGCGCTTCCCTAACCCGAACAGCTGGTTCTTAGGTCACTTCTCCAACGGTTTTGTTTGGACAGA
ATACATTGCCAAAGCGAAGAACCTTCCGCTCTACAACTGGGCAGTTGGCGGCGCGGCTGGTGAGAACCAA
TACATCGCGCTAACAGGGGTTGGTGAGCAAGTTTCTTCGTACTTAACCTACGCAAAACTGGCGAAGAACT
ACAAACCAGCAAACACCTTGTTTACGCTTGAGTTTGGTTTGAATGACTTCATGAACTACAACCGTGGCGT
TCCAGAAGTGAAAGCGGATTATGCAGAAGCACTGATTCGTTTGACGGACGCAGGTGCGAAGAACTTCATG
TTGATGACACTGCCAGATGCGACGAAAGCGCCTCAGTTTAAGTACTCAACACAAGAAGAGATCGACAAAA
TTCGTGCGAAAGTGCTTGAGATGAACGAGTTCATCAAGGCACAAGCGATGTACTACAAAGCGCAAGGTTA
CAACATCACGTTGTTTGATACTCACGCCTTGTTCGAGACGCTAACTTCTGCGCCAGAAGAGCACGGTTTC

GTGAACGCGAGCGATCCTTGTTTGGACATCAACCGCTCATCGTCTGTCGATTACATGTACACCCACGCAT
TGCGCTCTGAGTGTGCAGCGTCTGGTGCTGAGAAGTTTGTGTTCTGGGATGTCACGCATCCAACAACAGC
AACTCACCGCTATGTTGCAGAGAAAATGCTAGAAAGTAGCAACAACTTAGCCGAGTACCGTTTCTAA

>S137
ATGATGAAAAAAACAATCACACTATTAACTGCATTACTCCCGCTTGCTTCTGCAGTTGCCGAAGAGCCAA
CCTTATCACCAGAAATGGTTTCAGCGTCTGAAGTGATCAGCACGCAAGAAAACCAAACCTATACCTATGT
TCGCTGTTGGTATCGCACCAGCTACTCGAAAGATGATCCAGCGACCGATTGGGAATGGGCAAAAAACGAA
GATGGTAGCTACTTCACCATTGACGGCTACTGGTGGAGCTCCGTTTCATTTAAAAACATGTTCTACACCA
ACACGTCGCAAAACGTTATCCGTCAGCGTTGTGAAGCAACATTAGATTTGGCGAACGAGAACGCAGACAT
TACGTTCTTCGCCGCTGACAATCGCTTCTCATACAACCACACGATCTGGAGCAACGACGCAGCAATGCAG
CCAGATCAAATCAACAAAGTGGTTGCACTCGGTGACAGCTTGTCTGATACAGGCAACATCTTTAACGCAT
CACAATGGCGCTTCCCTAACCCGAACAGCTGGTTCTTAGGTCACTTCTCCAACGGTTTTGTTTGGACAGA
ATACATTGCCAAAGCGAAGAACCTTCCGCTCTACAACTGGGCAGTTGGCGGCGCGGCTGGTGAGAACCAA
TACATCGCGCTAACAGGGGTTGGTGAGCAAGTTTCTTCGTACTTAACCTACGCAAAACTGGCGAAGAACT
ACAAACCAGCAAACACCTTGTTTACGCTTGAGTTTGGTTTGAATGACTTCATGAACTACAACCGTGGCGT
TCCAGAAGTGAAAGCGGATTATGCAGAAGCACTGATTCGTTTGACGGACGCAGGTGCGAAGAACTTCATG
TTGATGACACTGCCAGATGCGACGAAAGCGCCTCAGTTTAAGTACTCAACACAAGAAGAGATCGACAAAA
TTCGTGCGAAAGTGCTTGAGATGAACGAGTTCATCAAGGCACAAGCGATGTACTACAAAGCGCAAGGTTA
CAACATCACGTTGTTTGATACTCACGCCTTGTTCGAGACGCTAACTTCTGCGCCAGAAGAGCACGGTTTC
GTGAACGCGAGCGATCCTTGTTTGGACATCAACCGCTCATCGTCTGTCGATTACATGTACACCCACGCAT
TGCGCTCTGAGTGTGCAGCGTCTGGTGCTGAGAAGTTTGTGTTCTGGGATGTCACGCATCCAACAACAGC
AACTCACCGCTATGTTGCAGAGAAAATGCTAGAAAGTAGCAACAACTTAGCCGAGTACCGTTTCTAA

>S136
ATGATGAAAAAAACAATCACACTATTAACTGCATTACTCCCGCTTGCTTCTGCAGTTGCCGAAGAGCCAACCTTAT
CACCAGAAATGGTTTCAGCGTCTGAAGTGATCAGCACGCAAGAAAACCAAACCTATACCTATGTTCGCTGTTGGTA
TCGCACCAGCTACTCGAAAGATGATCCAGCGACCGATTGGGAATGGGCAAAAAACGAAGATGGTAGCTACTTCAC
CATTGACGGCTACTGGTGGAGCTCCGTTTCATTTAAAAACATGTTCTACACCAACACGTCGCAAAACGTTATCCGT
CAGCGTTGTGAAGCAACATTAGATTTGGCGAACGAGAACGCAGACATTACGTTCTTCGCCGCTGACAATCGCTTCT
CATACAACCACACGATCTGGAGCAACGACGCAGCAATGCAGCCAGATCAAATCAACAAAGTGGTTGCACTCGGTG
ACAGCTTGTCTGATACAGGCAACATCTTTAACGCATCACAATGGCGCTTCCCTAACCCGAACAGCTGGTTCTTAGG
TCACTTCTCCAACGGTTTTGTTTGGACAGAATACATTGCCAAAGCGAAGAACCTTCCGCTCTACAACTGGGCAGTT
GGCGGCGCGGCTGGTGAGAACCAATACATCGCGCTAACAGGGGTTGGTGAGCAAGTTTCTTCGTACTTAACCTACG
CAAAACTGGCGAAGAACTACAAACCAGCAAACACCTTGTTTACGCTTGAGTTTGGTTTGAATGACTTCATGAACTA
CAACCGTGGCGTTCCAGAAGTGAAAGCGGATTATGCAGAAGCACTGATTCGTTTGACGGACGCAGGTGCGAAGAA
CTTCATGTTGATGACACTGCCAGATGCGACGAAAGCGCCTCAGTTTAAGTACTCAACACAAGAAGAGATCGACAA
AATTCGTGCGAAAGTGCTTGAGATGAACGAGTTCATCAAGGCACAAGCGATGTACTACAAAGCGCAAGGTTACAA
CATCACGTTGTTTGATACTCACGCCTTGTTCGAGACGCTAACTTCTGCGCCAGAAGAGCACGGTTTCGTGAACGCG
AGCGATCCTTGTTTGGACATCAACCGCTCATCGTCTGTCGATTACATGTACACCCACGCATTGCGCTCTGAGTGTGC
AGCGTCTGGTGCTGAGAAGTTTGTGTTCTGGGATGTCACGCATCCAACAACAGCAACTCACCGCTATGTTGCAGAG
AAAATGCTAGAAAGTAGCAACAACTTAGCCGAGTACCGTTTCTAA

>S135
ATGATGAAAAAAACAATCACACTATTAACTGCATTACTCCCGCTTGCTTCTGCAGTTGCCGAAGAGCCAA
CCTTATCACCAGAAATGGTTTCAGCGTCTGAAGTGATCAGCACGCAAGAAAACCAAACCTATACCTATGT
TCGCTGTTGGTATCGCACCAGCTACTCGAAAGATGATCCAGCGACCGATTGGGAATGGGCAAAAAACGAA
GATGGTAGCTACTTCACCATTGACGGCTACTGGTGGAGCTCCGTTTCATTTAAAAACATGTTCTACACCA
ACACGTCGCAAAACGTTATCCGTCAGCGTTGTGAAGCAACATTAGATTTGGCGAACGAGAACGCAGACAT
TACGTTCTTCGCCGCTGACAATCGCTTCTCATACAACCACACGATCTGGAGCAACGACGCAGCAATGCAG
CCAGATCAAATCAACAAAGTGGTTGCACTCGGTGACAGCTTGTCTGATACAGGCAACATCTTTAACGCAT

CACAATGGCGCTTCCCTAACCCGAACAGCTGGTTCTTAGGTCACTTCTCCAACGGTTTTGTTTGGACAGA
ATACATTGCCAAAGCGAAGAACCTTCCGCTCTACAACTGGGCAGTTGGCGGCGCGGCTGGTGAGAACCAA
TACATCGCGCTAACAGGGGTTGGTGAGCAAGTTTCTTCGTACTTAACCTACGCAAAACTGGCGAAGAACT
ACAAACCAGCAAACACCTTGTTTACGCTTGAGTTTGGTTTGAATGACTTCATGAACTACAACCGTGGCGT
TCCAGAAGTGAAAGCGGATTATGCAGAAGCACTGATTCGTTTGACGGACGCAGGTGCGAAGAACTTCATG
TTGATGACACTGCCAGATGCGACGAAAGCGCCTCAGTTTAAGTACTCAACACAAGAAGAGATCGACAAAA
TTCGTGCGAAAGTGCTTGAGATGAACGAGTTCATCAAGGCACAAGCGATGTACTACAAAGCGCAAGGTTA
CAACATCACGTTGTTTGATACTCACGCCTTGTTCGAGACGCTAACTTCTGCGCCAGAAGAGCACGGTTTC
GTGAACGCGAGCGATCCTTGTTTGGACATCAACCGCTCATCGTCTGTCGATTACATGTACACCCACGCAT
TGCGCTCTGAGTGTGCAGCGTCTGGTGCTGAGAAGTTTGTGTTCTGGGATGTCACGCATCCAACAACAGC
AACTCACCGCTATGTTGCAGAGAAAATGCTAGAAAGTAGCAACAACTTAGCCGAGTACCGTTTCTAA

>S134
ATGATGAAAAAAACAATCACACTATTAACTGCATTACTCCCGCTTGCTTCTGCAGTTGCCGAAGAGCCAACCTTAT
CACCAGAAATGGTTTCAGCGTCTGAAGTGATCAGCACGCAAGAAAACCAAACCTATACCTATGTTCGCTGTTGGTA
TCGCACCAGCTACTCGAAAGATGATCCGGCGACCGATTGGGAATGGGCAAAAAACGAAGATGGTAGCTACTTCAC
CATTGACGGCTACTGGTGGAGCTCCGTTTCATTTAAAAACATGTTCTACACCAACACGTCGCAAAACGTTATCCGT
CAGCGTTGTGAAGCAACATTAGATTTGGCGAACGAGAACGCAGACATTACGTTCTTCGCCGCTGACAATCGCTTCT
CATACAACCACACGATCTGGAGCAACGACGCAGCAATGCAGCCAGATCAAATCAACAAAGTGGTTGCACTCGGTG
ACAGCTTGTCTGATACAGGCAACATCTTTAACGCATCACAATGGCGCTTCCCTAACCCGAACAGCTGGTTCTTAGG
TCACTTCTCCAACGGTTTTGTGTGGACAGAATACATTGCCAAAGCGAAGAACCTTCCGCTCTACAACTGGGCAGTT
GGCGGCGCGGCTGGTGAGAACCAATACATCGCACTAACAGGGGTTGGTGATCAAGTTTCTTCGTACTTAACCTACG
CAAAACTGGCGAAGAACTACAAACCAGCAAACACCTTGTTTACGCTTGAGTTTGGTTTGAATGACTTCATGAACTA
CAACCGTGGCGTTCCAGAAGTGAAAGCAGATTATGCAGAAGCACTGATTCGTTTGACGGACGCAGGTGCGAAGAA
CTTCATGTTGATGACACTGCCAGATGCGACGAAAGCGCCTCAGTTTAAGTACTCAACACAAGAAGAGATCGACAA
AATTCGTGCGAAAGTGCTTGAGATGAACGAGTTCATCAAGGCACAAGCGATGTACTACAAAGCGCAAGGTTACAA
CATCACGTTGTTTGATACTCACGCCTTGTTCGAGACGCTAACTTCTGCGCCAGAAGAGCACGGTTTCGTGAACGCG
AGTGATCCTTGTTTGGACATCAACCGCTCATCGTCTGTCGATTACATGTACACCCACGCATTGCGCTCTGAGTGTGC
AGCGTCTGGTGCTGAGAAGTTTGTGTTCTGGGATGTCACGCACCCAACAACAGCAACTCACCGCTATGTTGCAGAG
AAAATGCTAGAAAGTAGCAACAACTTAGCAGAGTACCGTTTCTAA

>S133
ATGATGAAAAAAACAATCACACTATTAACTGCATTACTCCCGCTTGCTTCTGCAGTTGCCGAAGAGCCAA
CCTTATCACCAGAAATGGTTTCAGCGTCTGAAGTGATCAGCACGCAAGAAAACCAAACCTATACCTATGT
TCGCTGTTGGTATCGCACCAGCTACTCGAAAGATGATCCAGCGACCGATTGGGAATGGGCAAAAAACGAA
GATGGTAGCTACTTCACCATTGACGGCTACTGGTGGAGCTCCGTTTCATTTAAAAACATGTTCTACACCA
ACACGTCGCAAAACGTTATCCGTCAGCGTTGTGAAGCAACATTAGATTTGGCGAACGAGAACGCAGACAT
TACGTTCTTCGCCGCTGACAATCGCTTCTCATACAACCACACGATCTGGAGCAACGACGCAGCAATGCAG
CCAGATCAAATCAACAAAGTGGTTGCACTCGGTGACAGCTTGTCTGATACAGGCAACATCTTTAACGCAT
CACAATGGCGCTTCCCTAACCCGAACAGCTGGTTCTTAGGTCACTTCTCCAACGGTTTTGTTTGGACAGA
ATACATTGCCAAAGCGAAGAACCTTCCGCTCTACAACTGGGCAGTTGGCGGCGCGGCTGGTGAGAACCAA
TACATCGCGCTAACAGGGGTTGGTGAGCAAGTTTCTTCGTACTTAACCTACGCAAAACTGGCGAAGAACT
ACAAACCAGCAAACACCTTGTTTACGCTTGAGTTTGGTTTGAATGACTTCATGAACTACAACCGTGGCGT
TCCAGAAGTGAAAGCGGATTATGCAGAAGCACTGATTCGTTTGACGGACGCAGGTGCGAAGAACTTCATG
TTGATGACACTGCCAGATGCGACGAAAGCGCCTCAGTTTAAGTACTCAACACAAGAAGAGATCGACAAAA
TTCGTGCGAAAGTGCTTGAGATGAACGAGTTCATCAAGGCACAAGCGATGTACTACAAAGCGCAAGGTTA
CAACATCACGTTGTTTGATACTCACGCCTTGTTCGAGACGCTAACTTCTGCGCCAGAAGAGCACGGTTTC
GTGAACGCGAGCGATCCTTGTTTGGACATCAACCGCTCATCGTCTGTCGATTACATGTACACCCACGCAT
TGCGCTCTGAGTGTGCAGCGTCTGGTGCTGAGAAGTTTGTGTTCTGGGATGTCACGCATCCAACAACAGC
AACTCACCGCTATGTTGCAGAGAAAATGCTAGAAAGTAGCAACAACTTAGCCGAGTACCGTTTCTAA

>S132
ATGATGAAAAAAACAATCACACTATTAACTGCATTACTCCCGCTTGCTTCTGCAGTTGCCGAAGAGCCAA
CCTTATCACCAGAAATGGTTTCAGCGTCTGAAGTGATCAGCACGCAAGAAAACCAAACCTATACCTATGT
TCGCTGTTGGTATCGCACCAGCTACTCGAAAGATGATCCGGCGACCGATTGGGAATGGGCAAAAAACGAA
GATGGTAGCTACTTCACCATTGACGGCTACTGGTGGAGCTCCGTTTCATTTAAAAACATGTTCTACACCA
ACACGTCGCAAAACGTTATCCGTCAGCGTTGTGAAGCCACATTAGATTTGGCGAACGAGAACGCAGACAT
TACGTTCTTCGCCGCTGACAATCGCTTCTCATACAACCACACGATCTGGAGCAACGACGCAGCAATGCAG
CCAGATCAAATCAACAAAGTGGTTGCACTCGGTGACAGCTTGTCTGATACAGGCAACATCTTTAACGCAT
CACAATGGCGCTTCCCTAACCCGAACAGCTGGTTCTTAGGTCACTTCTCCAACGGTTTTGTGTGGACAGA
ATACATTGCCAAAGCGAAGAACCTTCCGCTCTACAACTGGGCAGTTGGCGGCGCGGCTGGTGAGAACCAA
TACATCGCGCTAACAGGGGTTGGTGATCAAGTTTCTTCGTACTTAACCTACGCAAAACTGGCGAAGAACT
ACAAACCAGCAAACACCTTGTTTACGCTTGAGTTTGGTTTGAATGACTTCATGAACTACAACCGTGGCGT
TCCAGAAGTGAAAGCAGATTATGCAGAAGCACTGATTCGTTTGACGGACGCAGGTGCAAAGAACTTCATG
TTGATGACACTGCCAGACGCGACGAAAGCGCCTCAGTTTAAGTACTCAACACAAGAAGAGATCGACAAAA
TTCGTGCGAAAGTGCTTGAGATGAACGAGTTCATCAAGGCACAAGCGATGTACTACAAAGCGCAAGGTTA
CAACATCACGTTGTTTGATACTCACGCCTTGTTCGAGACGCTAACTTCTGCGCCAGAAGAGCACGGTTTC
GTGAACGCGAGCGATCCTTGTTTGGACATCAACCGTTCATCGTCTGTCGATTACATGTACACCCACGCAT
TGCGCTCTGAGTGTGCGGCGTCTGGTGCTGAGAAGTTTGTGTTCTGGGATGTCACGCATCCAACAACAGC
AACTCACCGCTATGTTGCAGAGAAAATGCTAGAAAGTAGCAACAACTTAGCCGAGTACCGTTTCTAA

>S131
ATGATGAAAAAAACAATCACACTATTAACTGCATTACTCCCGCTTGCTTCTGCAGTTGCCGAAGAGCCAA
CCTTATCACCAGAAATGGTTTCAGCGTCTGAAGTGATCAGCACGCAAGAAAACCAAACCTATACCTATGT
TCGCTGTTGGTATCGCACCAGCTACTCGAAAGATGATCCAGCGACCGATTGGGAATGGGCAAAAAACGAA
GATGGTAGCTACTTCACCATTGACGGCTACTGGTGGAGCTCCGTTTCATTTAAAAACATGTTCTACACCA
ACACGTCGCAAAACGTTATCCGTCAGCGTTGTGAAGCAACATTAGATTTGGCGAACGAGAACGCAGACAT
TACGTTCTTCGCCGCTGACAATCGCTTCTCATACAACCACACGATCTGGAGCAACGACGCAGCAATGCAG
CCAGATCAAATCAACAAAGTGGTTGCACTCGGTGACAGCTTGTCTGATACAGGCAACATCTTTAACGCAT
CACAATGGCGCTTCCCTAACCCGAACAGCTGGTTCTTAGGTCACTTCTCCAACGGTTTTGTTTGGACAGA
ATACATTGCCAAAGCGAAGAACCTTCCGCTCTACAACTGGGCAGTTGGCGGCGCGGCTGGTGAGAACCAA
TACATCGCGCTAACAGGGGTTGGTGAGCAAGTTTCTTCGTACTTAACCTACGCAAAACTGGCGAAGAACT
ACAAACCAGCAAACACCTTGTTTACGCTTGAGTTTGGTTTGAATGACTTCATGAACTACAACCGTGGCGT
TCCAGAAGTGAAAGCGGATTATGCAGAAGCACTGATTCGTTTGACGGACGCAGGTGCGAAGAACTTCATG
TTGATGACACTGCCAGATGCGACGAAAGCGCCTCAGTTTAAGTACTCAACACAAGAAGAGATCGACAAAA
TTCGTGCGAAAGTGCTTGAGATGAACGAGTTCATCAAGGCACAAGCGATGTACTACAAAGCGCAAGGTTA
CAACATCACGTTGTTTGATACTCACGCCTTGTTCGAGACGCTAACTTCTGCGCCAGAAGAGCACGGTTTC
GTGAACGCGAGCGATCCTTGTTTGGACATCAACCGCTCATCGTCTGTCGATTACATGTACACCCACGCAT
TGCGCTCTGAGTGTGCAGCGTCTGGTGCTGAGAAGTTTGTGTTCTGGGATGTCACGCATCCAACAACAGC
AACTCACCGCTATGTTGCAGAGAAAATGCTAGAAAGTAGCAACAACTTAGCCGAGTACCGTTTCTAA

>S130
ATGATGAAAAAAACAATCACACTATTAACTGCATTACTCCCGCTTGCTTCTGCAGTTGCCGAAGAGCCAA
CCTTATCACCAGAAATGGTTTCAGCGTCTGAAGTGATCAGCACGCAAGAAAACCAAACCTATACCTATGT
TCGCTGTTGGTATCGCACCAGCTACTCGAAAGATGATCCAGCGACCGATTGGGAATGGGCAAAAAACGAA
GATGGTAGCTACTTCACCATTGACGGCTACTGGTGGAGCTCCGTTTCATTTAAAAACATGTTCTACACCA
ACACGTCGCAAAACGTTATCCGTCAGCGTTGTGAAGCAACATTAGATTTGGCGAACGAGAACGCAGACAT
TACGTTCTTCGCCGCTGACAATCGCTTCTCATACAACCACACGATCTGGAGCAACGACGCAGCAATGCAG
CCAGATCAAATCAACAAAGTGGTTGCACTCGGTGACAGCTTGTCTGATACAGGCAACATCTTTAACGCAT
CACAATGGCGCTTCCCTAACCCGAACAGCTGGTTCTTAGGTCACTTCTCCAACGGTTTTGTTTGGACAGA
ATACATTGCCAAAGCGAAGAACCTTCCGCTCTACAACTGGGCAGTTGGCGGCGCGGCTGGTGAGAACCAA
TACATCGCGCTAACAGGGGTTGGTGAGCAAGTTTCTTCGTACTTAACCTACGCAAAACTGGCGAAGAACT

ACAAACCAGCAAACACCTTGTTTACGCTTGAGTTTGGTTTGAATGACTTCATGAACTACAACCGTGGCGT
TCCAGAAGTGAAAGCGGATTATGCAGAAGCACTGATTCGTTTGACGGACGCAGGTGCGAAGAACTTCATG
TTGATGACACTGCCAGATGCGACGAAAGCGCCTCAGTTTAAGTACTCAACACAAGAAGAGATCGACAAAA
TTCGTGCGAAAGTGCTTGAGATGAACGAGTTCATCAAGGCACAAGCGATGTACTACAAAGCGCAAGGTTA
CAACATCACGTTGTTTGATACTCACGCCTTGTTCGAGACGCTAACTTCTGCGCCAGAAGAGCACGGTTTC
GTGAACGCGAGCGATCCTTGTTTGGACATCAACCGCTCATCGTCTGTCGATTACATGTACACCCACGCAT
TGCGCTCTGAGTGTGCAGCGTCTGGTGCTGAGAAGTTTGTGTTCTGGGATGTCACGCATCCAACAACAGC
AACTCACCGCTATGTTGCAGAGAAAATGCTAGAAAGTAGCAACAACTTAGCCGAGTACCGTTTCTAA

>S129
ATGATGAAAAAAACAATCACACTATTAACTGCATTACTCCCGCTTGCTTCTGCAGTTGCCGAAGAGCCAA
CCTTATCACCAGAAATGGTTTCAGCGTCTGAAGTGATCAGCACGCAAGAAAACCAAACCTATACCTATGT
TCGCTGTTGGTATCGCACCAGCTACTCGAAAGATGATCCGGCGACCGGTTGGGAATGGGCAAAAAACGAA
GATGGTAGCTACTTCACCATTGACGGCTACTGGTGGAGCTCCGTTTCATTTAAAAACATGTTCTACACCA
ACACGTCGCAAAACGTTATCCGTCAGCGTTGTGAAGCAACATTAGATTTGGCGAACGAGAACGCAGACAT
TACGTTCTTCGCCGCTGACAATCGCTTCTCATACAACCACACGATCTGGAGCAACGACGCAGCAATGCAG
CCAGATCAAATCAACAAAGTGGTTGCACTCGGTGACAGCTTGTCTGATACAGGCAACATCTTTAACGCAT
CACAATGGCGCTTCCCTAACCCGAACAGCTGGTTCTTAGGTCACTTCTCGAACGGTTTTGTGTGGACAGA
ATACATTGCCAAAGCGAAGAACCTTCCGCTCTACAACTGGGCAGTTGGCGGCGCGGCTGGTGAGAACCAA
TACATCGCGCTAACAGGGGTTGGTGATCAAGTTTCTTCGTACTTAACCTACGCAAAACTGGCGAAGAACT
ACAAACCAGCAAACACCTTGTTTACGCTTGAGTTTGGTTTGAATGACTTCATGAACTACAACCGTGGCGT
TCCAGAAGTGAAAGCGGATTATGCAGAAGCACTGATTCGTTTGACGGACGCAGGTGCGAAGAACTTCATG
TTGATGACACTGCCAGACGCGACGAAAGCGCCTCAGTTTAAGTACTCAACACAAGAAGAGATCGACAAAA
TTCGTGCGAAAGTGCTTGAGATGAACGAGTTCATCAAGGCACAAGCGATGTACTACAAAGCGCAAGGTTA
CAACATCACGTTGTTTGATACTCACGCCTTGTTCGAGACGCTAACTTCTGCGCCAGAAGAGCACGGTTTC
GTGAACGCGAGCGATCCTTGTTTGGACATCAACCGCTCATCGTCTGTCGACTACATGTACACCCACGCAT
TGCGCTCTGAGTGTGCGGCGTCTGGTGCTGAGAAGTTTGTATTCTGGGATGTCACGCACCCAACAACAGC
AACTCACCGCTATGTTGCAGAGAAAATGCTAGAAAGTAGCAACAACTTAGCCGAGTACCGTTTCTAA

>S128
ATGATGAAAAAAACAATCACACTATTAACTGCATTACTCCCACTTGCTTCTGCAGTTGCCGAAGAGCCAACCTTAT
CACCAGAAATGGTTTCAGCGTCTGAAGTGATCAGCACGCAAGAAAACCAAACCTATACCTATGTTCGCTGTTGGTA
TCGCACCAGCTACTCGAAAGATGATCCGGCGACCGATTGGGAATGGGCAAAAAACGAAGATGGTAGCTACTTCAC
CATTGACGGCTACTGGTGGAGCTCCGTTTCATTTAAAAACATGTTCTATACCAACACGTCGCAAAACGTTATCCGT
CAGCGTTGTGAAGCAACATTAGATTTGGCGAACGAGAACGCAGACATTACGTTCTTCGCCGCTGACAATCGCTTCT
CATACAACCACACGATCTGGAGCAACGACGCAGCAATGCAGCCAGATCAAATCAACAAAGTGGTTGCACTCGGTG
ACAGCTTGTCTGATACAGGCAACATCTTTAACGCATCACAATGGCGCTTCCCTAACCCGAACAGCTGGTTCTTAGG
TCACTTCTCCAACGGTTTTGTGTGGACAGAATACATTGCCAAAGCGAAGAACCTTCCGCTCTACAACTGGGCAGTT
GGCGGCGCGGCTGGTGAGAACCAATACATCGCGCTAACAGGGGTTGGTGATCAAGTTTCTTCGTACTTAACCTACG
CAAAACTGGCGAAGAACTACAAACCAGCAAACACCTTGTTTACGCTTGAGTTTGGTTTGAATGACTTCATGAACTA
CAACCGTGGCGTTCCAGAAGTGAAAGCGGATTATGCAGAAGCACTGATTCGTTTGACGGACGCAGGTGCGAAGAA
CTTCATGTTGATGACACTGCCAGACGCGACGAAAGCGCCTCAGTTTAAGTACTCAACACAAGAAGAGATCGACAA
AATTCGTGCGAAAGTGCTTGAGATGAACGAGTTCATCAAGGCACAAGCGATGTACTACAAAGCGCAAGGTTACAA
CATCACGTTGTTTGATACTCACGCCTTGTTCGAGACGCTAACTTCTGCGCCAGAAGAGCACGGTTTCGTGAACGCG
AGCGATCCTTGTTTGGACATCAACCGCTCATCGTCTGTCGACTACATGTACACCCACGCATTGCGCTCTGAGTGTGC
GGCGTCTGGTGCTGAGAAGTTTGTATTCTGGGATGTCACGCACCCAACAACAGCAACTCACCGCATGTTGCAGAGA
AAATGCTAGAAAGTAGCAACAACTTAGCCGAGTACCGTTTCTAA

>S126
ATGATGAAAAAAACAATCACACTATTAACTGCATTACTCCCGCTTGCTTCTGCAGTTGCCGAAGAGCCAACCTTAT
CACCAGAAATGGTTTCAGCGTCTGAAGTGATCAGCACGCAAGAAAACCAAACCTATACCTATGTTCGCTGTTGGTA

TCGCACCAGCTACTCGAAAGATGATCCAGCGACCGATTGGGAATGGGCAAAAAACGAAGATGGTAGCTACTTCAC
CATTGACGGCTACTGGTGGAGCTCCGTTTCATTTAAAAACATGTTCTACACCAACACGTCGCAAAACGTTATCCGT
CAGCGTTGTGAAGCAACATTAGATTTGGCGAACGAGAACGCAGACATTACGTTCTTCGCCGCTGACAATCGCTTCT
CATACAACCACACGATCTGGAGCAACGACGCAGCAATGCAGCCAGATCAAATCAACAAAGTGGTTGCACTCGGTG
ACAGCTTGTCTGATACAGGCAACATCTTTAACGCATCACAATGGCGCTTCCCTAACCCGAACAGCTGGTTCTTAGG
TCACTTCTCCAACGGTTTTGTTTGGACAGAATACATTGCCAAAGCGAAGAACCTTCCGCTCTACAACTGGGCAGTT
GGCGGCGCGGCTGGTGAGAACCAATACATCGCGCTAACAGGGGTTGGTGAGCAAGTTTCTTCGTACTTAACCTACG
CAAAACTGGCGAAGAACTACAAACCAGCAAACACCTTGTTTACGCTTGAGTTTGGTTTGAATGACTTCATGAACTA
CAACCGTGGCGTTCCAGAAGTGAAAGCGGATTATGCAGAAGCACTGATTCGTTTGACGGACGCAGGTGCGAAGAA
CTTCATGTTGATGACACTGCCAGATGCGACGAAAGCGCCTCAGTTTAAGTACTCAACACAAGAAGAGATCGACAA
AATTCGTGCGAAAGTGCTTGAGATGAACGAGTTCATCAAGGCACAAGCGATGTACTACAAAGCGCAAGGTTACAA
CATCACGTTGTTTGATACTCACGCCTTGTTCGAGACGCTAACTTCTGCGCCAGAAGAGCACGGTTTCGTGAACGCG
AGCGATCCTTGTTTGGACATCAACCGCTCATCGTCTGTCGATTACATGTACACCCACGCATTGCGCTCTGAGTGTGC
AGCGTCTGGTGCTGAGAAGTTTGTGTTCTGGGATGTCACGCATCCAACAACAGCAACTCACCGCTATGTTGCAGAG
AAAATGCTAGAAAGTAGCAACAACTTAGCCGAGTACCGTTTCTAA

>S125
ATGATGAAAAAAACAATCACACTATTAACTGCATTACTCCCGCTTGCTTCTGCAGTTGCCGAAGAGCCAACCTTAT
CACCAGAAATGGTTTCAGCGTCTGAAGTGATCAGCACGCAAGAAAACCAAACCTATACCTATGTTCGCTGTTGGTA
TCGCACCAGCTACTCGAAAGATGATCCGGCGACCGATTGGGAATGGGCAAAAAACGAAGATGGTAGCTACTTCAC
CATTGACGGCTACTGGTGGAGCTCCGTTTCATTTAAAAACATGTTCTACACCAACACGTCGCAAAACGTTATCCGT
CAGCGTTGTGAAGCAACATTAGATTTGGCGAACGAGAACGCAGACATTACGTTCTTCGCCGCTGACAATCGCTTCT
CATACAACCACACGATCTGGAGCAACGACGCAGCAATGCAGCCAGATCAAATCAACAAAGTGGTTGCACTCGGTG
ACAGCTTGTCTGATACAGGCAACATCTTTAACGCATCACAATGGCGCTTCCCTAACCCGAACAGCTGGTTCTTAGG
TCACTTCTCCAACGGTTTTGTGTGGACAGAATACATTGCCAAAGCGAAGAACCTTCCGCTCTACAACTGGGCAGTT
GGCGGCGCGGCTGGTGAGAACCAATACATCGCGCTAACAGGGGTTGGTGATCAAGTTTCTTCGTACTTAACCTACG
CAAAACTGGCGAAGAACTACAAACCAGCAAACACCTTGTTTACGCTTGAGTTTGGTTTGAATGACTTCATGAACTA
CAACCGTGGCGTTCCAGAAGTGAAAGCGGATTATGCAGAAGCACTGATTCGTTTGACGGACGCAGGTGCGAAGAA
CTTCATGTTGATGACACTGCCAGACGCGACGAAAGCGCCTCAGTTTAAGTACTCAACACAAGAAGAGATCGACAA
AATTCGTGCGAAAGTGCTTGAGATGAACGAGTTCATCAAGGCACAAGCGATGTACTACAAAGCGCAAGGTTACAA
CATCACGTTGTTTGATACTCACGCCTTGTTCGAGACGCTAACTTCTGCGCCAGAAGAGCACGGTTTCGTGAACGCG
AGCGATCCTTGTTTGGACATCAACCGCTCATCGTCTGTCGATTACATGTACACCCACGCATTGCGCTCTGAGTGTGC
AGCGTCTGGTGCTGAGAAGTTTGTGTTCTGGGATGTCACGCATCCAACAACAGCAACTCACCGCTATGTTGCAGAG
AAAATGCTAGAAAGTAGCAACAACTTAGCCGAGTACCGTTTCTAA

>S124
ATGATGAAAAAAACAATCACACTATTAACTGCATTACTCCCGCTTGCTTCTGCAGTTGCCGAAGAGCCAA
CCTTATCACCAGAAATGGTTTCAGCGTCTGAAGTGATCAGCACGCAAGAAAACCAAACCTATACCTATGT
TCGCTGTTGGTATCGCACCAGCTACTCGAAAGATGATCCGGCGACCGATTGGGAATGGGCAAAAAACGAA
GATGGTAGCTACTTCACCATTGACGGCTACTGGTGGAGCTCCGTTTCATTTAAAAACATGTTCTACACCA
ACACGTCGCAAAACGTTATCCGTCAGCGTTGTGAAGCAACATTAGATTTGGCGAACGAGAACGCAGACAT
TACGTTCTTCGCCGCTGACAATCGCTTCTCATACAACCACACGATCTGGAGCAACGACGCAGCAATGCAG
CCAGATCAAATCAACAAAGTGGTTGCACTCGGTGACAGCTTGTCTGATACAGGCAACATCTTTAACGCAT
CACAATGGCGCTTCCCCAACCCGAACAGCTGGTTCTTAGGTCACTTCTCCAACGGTTTTGTTTGGACAGA
ATACATTGCCAAAGCGAAGAACCTTCCGCTCTACAACTGGGCAGTTGGCGGCGCGGCTGGTGAGAACCAA
TACATCGCGCTAACAGGGGTTGGTGAGCAAGTTTCTTCGTACTTAACCTACGCAAAACTGGCGAAGAACT
ACAAACCAGCAAACACCTTGTTTACGCTTGAGTTTGGTTTGAATGACTTCATGAACTACAACCGTGGCGT
TCCAGAAGTGAAAGCAGATTATGCAGAAGCACTGATTCGTTTGACGGACGCAGGTGCGAAGAACTTCATG
TTGATGACACTGCCAGACGCGACGAAAGCGCCTCAGTTTAAGTACTCAACACAAGAAGAGATCGACAAAA
TTCGTGCGAAAGTGCTTGAGATGAACGAGTTCATCAAGGCACAAGCGATGTACTACAAAGCGCAAGGTTA
CAACATCACGTTGTTTGATACTCACGCCTTGTTCGAGACGCTAACTTCTGCGCCAGAAGAGCACGGTTTC

GTGAACGCGAGCGATCCTTGTTTGGACATCAACCGCTCATCGTCTGTCGATTACATGTACACCCACGCAT
TGCGCTCTGAGTGTGCAGCGTCTGGTGCTGAGAAGTTTGTGTTCTGGGATGTCACGCATCCAACAACAGC
AACTCACCGCTATGTTGCAGAGAAAATGCTAGAAAGTAGCAACAACTTAGCCGAGTACCGTTTCTAA

>S123
ATGATGAAAAAAACAATCACACTATTAACTGCATTACTCCCGCTTGCTTCTGCAGTTGCCGAAGAGCCAACCTTAT
CACCAGAAATGGTTTCAGCGTCTGAAGTGATCAGCACGCAAGAAAACCAAACCTATACCTATGTTCGCTGTTGGTA
TCGCACCAGCTACTCGAAAGATGATCCGGCGACCGATTGGGAATGGGCAAAAAACGAAGATGGTAGCTACTTCAC
CATTGACGGCTACTGGTGGAGCTCCGTTTCATTTAAAAACATGTTCTACACCAACACGTCGCAAAACGTTATCCGT
CAGCGTTGTGAAGCAACATTAGATTTGGCGAACGAGAACGCAGACATTACGTTCTTCGCCGCTGACAATCGCTTCT
CATACAACCACACGATCTGGAGCAACGACGCAGCAATGCAGCCAGATCAAATCAACAAAGTGGTTGCACTCGGTG
ACAGCTTGTCTGATACAGGCAACATCTTTAACGCATCACAATGGCGCTTCCCTAACCCGAACAGCTGGTTCTTAGG
TCACTTCTCCAACGGTTTTGTGTGGACAGAATACATTGCCAAAGCGAAGAACCTTCCGCTCTACAACTGGGCAGTT
GGCGGCGCGGCTGGTGAGAACCAATACATCGCGCTAACAGGGGTTGGTGATCAAGTTTCTTCGTACTTAACCTACG
CAAAACTGGCGAAGAACTACAAACCAGCAAACACCTTGTTTACGCTTGAGTTTGGTTTGAATGACTTCATGAACTA
CAACCGTGGCGTTCCAGAAGTGAAAGCGGATTATGCAGAAGCACTGATTCGTTTGACGGACGCAGGTGCGAAGAA
CTTCATGTTGATGACACTGCCAGACGCGACGAAAGCGCCTCAGTTTAAGTACTCAACACAAGAAGAGATCGACAA
AATTCGTGCGAAAGTGCTTGAGATGAACGAGTTCATCAAGGCACAAGCGATGTACTACAAAGCGCAAGGTTACAA
CATCACGTTGTTTGATACTCACGCCTTGTTCGAGACGCTAACTTCTGCGCCAGAAGAGCACGGTTTCGTGAACGCG
AGCGATCCTTGTTTGGACATCAACCGCTCATCGTCTGTCGATTACATGTACACCCACGCATTGCGCTCTGAGTGTGC
AGCGTCTGGTGCTGAGAAGTTTGTGTTCTGGGATGTCACGCATCCAACAACAGCAACTCACCGCTATGTTGCAGAG
AAAATGCTAGGAAGTAGCAACAACTTAGCAGAGTACCGTTTCTAA

>S122
ATGATGAAAAAAACAATCACACTATTAACTGCATTACTCCCGCTTGCTTCTGCAGTTGCCGAAGAGCCAACCTTAT
CACCAGAAATGGTTTCAGCGTCTGAAGTGATCAGCACGCAAGAAAACCAAACCTATACCTATGTTCGCTGTTGGTA
TCGCACCAGCTACTCGAAAGATGATCCGGCGACCGATTGGGAATGGGCAAAAAACGAAGATGGTAGCTACTTCAC
CATTGACGGCTACTGGTGGAGCTCCGTTTCATTTAAAAACATGTTCTACACCAACACGTCGCAAAACGTTATCCGT
CAGCGTTGTGAAGCAACATTAGATTTGGCGAACGAGAACGCAGACATTACGTTCTTCGCCGCTGACAATCGCTTCT
CATACAACCACACGATCTGGAGCAACGACGCAGCAATGCAGCCAGATCAAATCAACAAAGTGGTTGCACTCGGTG
ACAGCTTGTCTGATACAGGCAACATCTTTAACGCATCACAATGGCGCTTCCCTAACCCGAACAGCTGGTTCTTAGG
TCACTTCTCCAACGGTTTTGTGTGGACAGAATACATTGCCAAAGCGAAGAACCTTCCGCTCTACAACTGGGCAGTT
GGCGGCGCGGCTGGTGAGAACCAATACATCGCGCTAACAGGGGTTGGTGATCAAGTTTCTTCGTACTTAACCTACG
CAAAACTGGCGAAGAACTACAAACCAGCAAACACCTTGTTTACGCTTGAGTTTGGTTTGAATGACTTCATGAACTA
CAACCGTGGCGTTCCAGAAGTGAAAGCGGATTATGCAGAAGCACTGATTCGTTTGACGGACGCAGGTGCGAAGAA
CTTCATGTTGATGACACTGCCAGACGCGACGAAAGCGCCTCAGTTTAAGTACTCAACACAAGAAGAGATCGACAA
AATTCGTGCGAAAGTGCTTGAGATGAACGAGTTCATCAAGGCACAAGCGATGTACTACAAAGCGCAAGGTTACAA
CATCACGTTGTTTGATACTCACGCCTTGTTCGAGACGCTAACTTCTGCGCCAGAAGAGCACGGTTTCGTGAACGCG
AGCGATCCTTGTTTGGACATCAACCGCTCATCGTCTGTCGATTACATGTACACCCACGCATTGCGCTCTGAGTGTGC
AGCGTCTGGTGCTGAGAAGTTTGTGTTCTGGGATGTCACGCATCCAACAACAGCAACTCACCGCTATGTTGCAGAG
AAAATGCTAGGAAGTAGCAACAACTTAGCAGAGTACCGTTTCTAA

>S121
ATGATGAAAAAAACAATCACACTATTAACTGCATTACTCCCGCTTGCTTCTGCAGTTGCCGAAGAGCCAA
CCTTATCACCAGAAATGGTTTCAGCGTCTGAAGTGATCAGCACGCAAGAAAACCAAACCTATACCTATGT
TCGCTGTTGGTATCGCACCAGCTACTCGAAAGATGATCCGGCGACCGATTGGGAATGGGCAAAAAACGAA
GATGGTAGCTACTTCACCATTGACGGCTACTGGTGGAGCTCCGTTTCATTTAAAAACATGTTCTACACCA
ACACGTCGCAAAACGTTATCCGTCAGCGTTGTGAAGCAACATTAGATTTGGCGAACGAGAACGCAGACAT
TACGTTCTTCGCCGCTGACAATCGCTTCTCATACAACCACACGATCTGGAGCAACGACGCAGCAATGCAG
CCAGATCAAATCAACAAAGTGGTTGCACTCGGTGACAGCTTGTCTGATACAGGCAACATCTTTAACGCAT
CACAATGGCGCTTCCCTAACCCGAACAGCTGGTTCTTAGGTCACTTCTCCAACGGTTTTGTGTGGACAGA

ATACATTGCCAAAGCGAAGAACCTTCCGCTCTACAACTGGGCAGTTGGCGGCGCGGCTGGTGAGAACCAA
TACATCGCGCTAACAGGGGTTGGTGATCAAGTTTCTTCGTACTTAACCTACGCAAAACTGGCGAAGAACT
ACAAACCAGCAAACACCTTGTTTACGCTTGAGTTTGGTTTGAATGACTTCATGAACTACAACCGTGGCGT
TCCAGAAGTGAAAGCGGATTATGCAGAAGCACTGATTCGTTTGACGGACGCAGGTGCGAAGAACTTCATG
TTGATGACACTGCCAGATGCGACGAAAGCGCCTCAGTTTAAGTACTCAACACAAGAAGAGATCGACAAAA
TTCGTGCGAAAGTGCTTGAGATGAACGAGTTCATCAAGGCACAAGCGATGTACTACAAAGCGCAAGGTTA
CAACATCACGTTGTTTGATACTCACGCCTTGTTCGAGACGCTAACTTCTGCGCCAGAAGAGCACGGTTTC
GTGAACGCGAGCGATCCTTGTTTGGACATCAACCGCTCATCGTCTGTCGATTACATGTACACCCACGCAT
TGCGCTCTGAGTGTGCAGCGTCTGGTGCTGAGAAGTTTGTGTTCTGGGATGTCACGCATCCAACAACAGC
AACTCACCGCTATGTTGCAGAGAAAATGCTAGAAAGTAGCAACAACTTAGCAGAGTACCGTTTCTAA

>S120
ATGATGAAAAAAACAATCACACTATTAACTGCATTACTCCCGCTTGCTTCTGCAGTTGCCGAAGAGCCAACCTTAT
CACCAGAAATGGTTTCAGCGTCTGAAGTGATCAGCACGCAAGAAAACCAAACCTATACCTATGTTCGCTGTTGGTA
TCGCACCAGCTACTCGAAAGATGATCCGGCGACCGATTGGGAATGGGCAAAAAACGAAGATGGTAGCTACTTCAC
CATTGACGGCTACTGGTGGAGCTCCGTCTCATTTAAAAACATGTTCTACACCAACACGTCGCAAAACGTTATCCGT
CAGCGTTGTGAAGCAACATTAGATTTGGCGAACGAGAACGCAGACATTACGTTCTTCGCCGCTGACAATCGCTTCT
CATACAACCACACGATCTGGAGCAACGACGCAGCAATGCAGCCAGATCAAATCAACAAAGTGGTTGCACTCGGTG
ACAGCTTGTCTGATACAGGCAACATCTTTAACGCATCACAATGGCGCTTCCCTAACCCGAACAGCTGGTTCTTAGG
TCACTTCTCCAACGGTTTTGTGTGGACAGAATACATTGCCAAAGCGAAGAACCTTCCGCTCTACAACTGGGCAGTT
GGCGGCGCGGCTGGTGAGAACCAATACATCGCGCTAACAGGGGTTGGTGATCAAGTTTCTTCGTACTTAACCTACG
CAAAACTGGCGAAGAACTACAAACCAGCAAACACATTGTTTACGCTTGAGTTTGGTTTGAATGACTTCATGAACTA
CAACCGTGGCGTTCCAGAAGTGAAAGCGGATTATGCAGAAGCACTGATTCGTTTGACGGACGCAGGTGCGAAGAA
CTTCATGTTGATGACACTGCCAGACGCGACGAAAGCGCCTCAGTTTAAGTACTCAACACAAGAAGAGATCGACAA
AATTCGTGCGAAAGTGCTTGAGATGAACGAGTTCATCAAGGCACAAGCGATGTACTACAAAGCGCAAGGTTACAA
CATCACGTTGTTTGATACTCACGCCTTGTTCGAGACGCTAACTTCTGCGCCAGAAGAGCACGGTTTCGTGAACGCG
AGTGATCCTTGTTTGGACATCAACCGCTCATCGTCTGTCGATTACATGTACACCCACGCATTGCGCTCTGAGTGTGC
GGCGTCTGGTGCTGAGAAATTTGTGTTCTGGGATGTCACGCACCCGACAACAGCAACTCACCGCTATGTTGCAGAG
AAAATGCTAGAAAGTAGCAACAACTTAGCCGAGTACCGTTTCTAA

>S119
ATGATGAAAAAAACAATCACACTATTAACTGCATTACTCCCGCTTGCTTCTGCAGTTGCCGAAGAGCCAACCTTAT
CACCAGAAATGGTTTCAGCGTCGGAAGTGATCAGCACGCAAGAAAACCAAACCTATACCTATGTTCGCTGTTGGTA
TCGCACCAGCTACTCGAAAGATGATCCGGCGACCGATTGGGAATGGGCAAAAAACGAAGATGGTAGCTACTTCAC
CATTGACGGCTACTGGTGGAGCTCCGTTTCATTTAAAAACATGTTCTACACCAACACGTCGCAAAACGTTATCCGT
CAGCGTTGTGAAGCAACATTAGATTTGGCGAACGAGAACGCAGACATTACGTTCTTCGCCGCTGACAATCGCTTCT
CATACAACCACACGATCTGGAGCAACGACGCAGCAATGCAGCCAGATCAAATCAACAAAGTGGTTGCACTCGGTG
ACAGCTTGTCTGATACAGGCAACATCTTTAACGCATCACAATGGCGCTTCCCTAACCCGAACAGCTGGTTCTTAGG
TCACTTCTCCAACGGTTTTGTGTGGACAGAATACATTGCCAAAGCGAAGAACCTTCCGCTCTACAACTGGGCAGTT
GGCGGCGCGGCTGGTGAGAACCAATACATCGCGCTAACAGGGGTTGGTGATCAAGTTTCTTCGTACTTAACCTACG
CAAAACTGGCGAAGAACTACAAACCAGCAAACACCTTGTTTACGCTTGAGTTTGGTTTGAATGACTTCATGAACTA
CAACCGTGGCGTTCCAGAAGTGAAAGCAGATTATGCAGAAGCACTGATTCGTTTGACGGACGCAGGTGCGAAGAA
CTTCATGTTGATGACACTGCCAGACGCGACGAAAGCGCCTCAGTTTAAGTACTCAACACAAGAAGAGATCGACAA
AATTCGTGCGAAAGTGCTTGAGATGAACGAGTTCATCAAGGCACAAGCGATGTACTACAAAGCGCAAGGTTACAA
CATCACGTTGTTTGATACTCACGCCTTGTTCGAGACGCTAACTTCTGCGCCCGAAGAACACGGTTTCGTGAACGCG
AGTGATCCTTGTTTGGACATCAACCGCTCATCGTCTGTCGATTACATGTACACCCACGCATTGCGCTCTGAGTGTGC
GGCGTCTGGTGCTGAGAAATTTGTGTTCTGGGATGTCACGCACCCAACAACAGCAACTCACCGCTATGTTGCAGAG
AAAATGCTAGAAAGTAGCAACAACTTAGCCGAGTACCGTTTCTAA

>S118

ATGATGAAAAAAACAATCACACTATTAACTGCATTACTCCCGCTTGCTTCTGCAGTTGCCGAAGAGCCAACCTTAT
CACCAGAAATGGTTTCAGCGTCTGAAGTGATCAGCACGCAAGAAAACCAAACCTATACCTATGTTCGCTGTTGGTA
TCGCACCAGCTACTCGAAAGATGATCCGGCGACCGATTGGGAATGGGCAAAAAACGAAGATGGTAGCTACTTCAC
CATTGACGGCTACTGGTGGAGCTCCGTTTCATTTAAAAACATGTTCTACACCAACACGTCGCAAAACGTTATCCGT
CAGCGTTGTGAAGCAACATTAGATTTGGCGAACGAGAACGCAGACATTACGTTCTTCGCCGCTGACAATCGCTTCT
CATACAACCACACGATCTGGAGCAACGACGCAGTAATGCAGCCAGATCAAATCAACAAAGTGGTTGCACTCGGTG
ACAGCTTGTCTGATACAGGCAACATCTTTAACGCATCACAATGGCGCTTCCCTAACCCGAACAGCTGGTTCTTAGG
TCACTTCTCCAACGGTTTTGTTTGGACAGAATACATTGCCAAAGCGAAGAACCTTCCGCTCTACAACTGGGCAGTT
GGCGGCGCGGCTGGTGAGAACCAATACATCGCGCTAACAGGGGTTGGTGATCAAGTTTCTTCGTACTTAACCTACG
CAAAACTGGCGAAGAACTACAAACCAGCAAACACCTTGTTTACGCTTGAGTTTGGTTTGAATGACTTCATGAACTA
CAACCGTGGCGTTCCAGAAGTGAAAGCAGATTATGCAGAAGCACTGATTCGTTTGACGGACGCAGGTGCGAAGAA
CTTCATGTTGATGACACTGCCAGACGCGACGAAAGCGCCTCAGTTTAAGTACTCAACACAAGAAGAGATCGACAA
AATTCGTGCGAAAGTGCTTGAGATGAACGAGTTCATCAAGGCACAAGCGATGTACTACAAAGCGCAAGGTTACAA
CATCACGTTGTTTGATACTCACGCCTTGTTCGAGACGCTAACTTCTGCGCCAGAAGAGCACGGTTTCGTGAACGCG
AGTGATCCTTGTTTGGACATCAACCGCTCATCGTCTGTCGATTACATGTACACCCACGCATTGCGCTCTGAGTGTGC
GGCGTCTGGTGCTGAGAAGTTTGTGTTCTGGGATGTCACGCACCCAACAACAGCAACTCACCGCTATGTTGCAGAG
AAAATGCTAGAAAGTAGCAACAACTTAGCCGAGTACCGTTTCTAA

>S117
ATGATGAAAAAAACAATCACACTATTAACTGCATTACTCCCGCTTGCTTCTGCAGTTGCCGAAGAGCCAACCTTAT
CACCAGAAATGGTTTCAGCGTCTGAAGTGATCAGCACGCAAGAAAACCAAACCTATACCTATGTTCGCTGTTGGTA
TCGCACCAGCTACTCGAAAGATGATCCGGCGACCGATTGGGAATGGGCAAAAAACGAAGATGGTAGCTACTTCAC
CATTGACGGCTACTGGTGGAGCTCCGTTTCATTTAAAAACATGTTCTACACCAACACGTCGCAAAACGTTATCCGT
CAGCGTTGTGAAGCAACATTAGATTTGGCGAACGAGAACGCAGACATTACGTTCTTCGCCGCTGACAATCGCTTCT
CATACAACCACACGATCTGGAGCAACGACGCAGCAATGCAGCCAGATCAAATCAACAAAGTGGTTGCACTCGGTG
ACAGCTTGTCTGATACAGGCAACATCTTTAACGCATCACAATGGCGCTTCCCTAACCCGAACAGCTGGTTCTTAGG
TCACTTCTCCAACGGTTTTGTGTGGACAGAATACATTGCCAAAGCGAAGAACCTTCCGCTCTACAACTGGGCAGTT
GGCGGCGCGGCTGGTGAGAACCAATACATCGCGCTAACAGGGGTTGGTGATCAAGTTTCTTCGTACTTAACCTACG
CAAAACTGGCGAAGAACTACAAACCAGCAAACACCTTGTTTACGCTTGAGTTTGGTTTGAATGACTTCATGAACTA
CAACCGTGGCGTTCCAGAAGTGAAAGCGGATTATGCAGAAGCACTGATTCGTTTGACGGACGCAGGTGCGAAGAA
CTTCATGTTGATGACACTGCCAGACGCGACGAAAGCGCCTCAGTTTAAGTACTCAACACAAGAAGAGATCGACAA
AATTCGTGCGAAAGTGCTTGAGATGAACGAGTTCATCAAGGCACAAGCGATGTACTACAAAGCGCAAGGTTACAA
CATCACGTTGTTTGATACTCACGCCTTGTTCGAGACGCTAACTTCTGCGCCAGAAGAGCACGGTTTCGTGAACGCG
AGCGATCCTTGTTTGGACATCAACCGCTCATCGTCTGTCGATTACATGTACACCCACGCATTGCGCTCTGAGTGTGC
AGCGTCTGGTGCTGAGAAGTTTGTGTTCTGGGATGTCACGCATCCAACAACAGCAACTCACCGCTATGTTGCAGAG
AAAATGCTAGAAAGTAGCAACAACTTAGCCGAGTACCGTTTCTAA

>S116
ATGATGAAAAAAACAATCACACTATTAACTGCATTACTCCCGCTTGCTTCTGCAGTTGCCGAAGAGCCAA
CCTTATCACCAGAAATGGTTTCAGCGTCTGAAGTGATCAGCACGCAAGAAAACCAAACCTATACCTATGT
TCGCTGTTGGTATCGCACCAGCTACTCGAAAGATGATCCGGCGACCGATTGGGAATGGGCAAAAAACGAA
GATGGTAGCTACTTCACCATTGACGGCTACTGGTGGAGCTCCGTTTCATTTAAAAACATGTTCTACACCA
ACACGTCGCAAAACGTTATCCGTCAGCGTTGTGAAGCAACATTAGATTTGGCGAACGAGAACGCAGACAT
TACGTTCTTCGCCGCTGACAATCGCTTCTCATACAACCACACGATCTGGAGCAACGACGCAGCAATGCAG
CCAGATCAAATCAACAAAGTGGTTGCACTCGGTGACAGCTTGTCTGATACAGGCAACATCTTTAACGCAT
CACAATGGCGCTTCCCTAACCCGAACAGCTGGTTCTTAGGTCACTTCTCCAACGGTTTTGTGTGGACAGA
ATACATTGCCAAAGCGAAGAACCTTCCGCTCTACAACTGGGCAGTTGGCGGCGCGGCTGGTGAGAACCAA
TACATCGCGCTAACAGGGGTTGGTGATCAAGTTTCTTCGTACTTAACCTACGCAAAACTGGCGAAGAACT
ACAAACCAGCAAACACCTTGTTTACGCTTGAGTTTGGTTTGAATGACTTCATGAACTACAACCGTGGCGT
TCCAGAAGTGAAAGCGGATTATGCAGAAGCACTGATTCGTTTGACGGACGCAGGTGCGAAGAACTTCATG
TTGATGACACTGCCAGATGCGACGAAAGCGCCTCAGTTTAAGTACTCAACACAAGAAGAGATCGACAAAA

TTCGTGCGAAAGTGCTTGAGATGAACGAGTTCATCAAGGCACAAGCGATGTACTACAAAGCGCAAGGTTA
CAACATCACGTTGTTTGATACTCACGCCTTGTTCGAGACGCTAACTTCTGCGCCAGAAGAGCACGGTTTC
GTGAACGCGAGCGATCCTTGTTTGGACATCAACCGCTCATCGTCTGTCGATTACATGTACACCCACGCAT
TGCGCTCTGAGTGTGCAGCGTCTGGTGCTGAGAAGTTTGTGTTCTGGGATGTCACGCATCCAACAACAGC
AACTCACCGCTATGTTGCAGAGAAAATGCTAGAAAGTAGCAACAACTTAGCAGAGTACCGTTTCTAA

>S115
ATGATGAAAAAAACAATCACACTATTAACTGCATTACTCCCGCTTGCTTCTGCAGTTGCCGAAGAGCCAA
CCTTATCACCAGAAATGGTTTCAGCGTCTGAAGTGATCAGCACGCAAGAAAACCAAACCTATACCTATGT
TCGCTGTTGGTATCGCACCAGCTATTCGAAAGATGATCCGGCGACCGATTGGGAATGGGCAAAAAACGAA
GATGGTAGCTACTTCACCATTGACGGCTACTGGTGGAGCTCCGTTTCATTTAAAAACATGTTCTACACCA
ACACGTCGCAAAACGTTATCCGTCAGCGTTGTGAAGCAACATTAGATTTGGCGAACGAGAACGCAGACAT
TACGTTCTTCGCCGCTGACAATCGCTTCTCATACAACCACACGATCTGGAGCAACGACGCAGCAATGCAG
CCAGATCAAATCAACAAAGTGGTTGCACTCGGTGACAGCTTGTCTGATACAGGCAACATCTTTAACGCAT
CACAATGGCGCTTCCCTAACCCGAACAGCTGGTTCTTAGGTCACTTCTCCAACGGTTTTGTGTGGACAGA
ATACATTGCCAAAGCGAAGAACCTTCCGCTCTACAACTGGGCAGTTGGCGGCGCGGCTGGTGAGAACCAA
TACATCGCGCTAACAGGGGTTGGTGAGCAAGTTTCTTCGTACTTAACCTACGCAAAACTGGCGAAGAACT
ACAAACCAGCAAACACCTTGTTTACGCTTGAGTTTGGTTTGAATGACTTCATGAACTACAACCGTGGCGT
TCCAGAAGTGAAAGCAGATTATGCAGAAGCACTGATTCGTTTGACGGACGCAGGTGCGAAGAACTTCATG
TTGATGACACTGCCAGACGCGACGAAAGCGCCTCAGTTTAAGTACTCAACACAAGAAGAGATCGACAAAA
TTCGTGCGAAAGTGCTTGAGATGAACGAGTTCATCAAGGCACAAGCGATGTACTACAAAGCGCAAGGTTA
CAACATCACGTTGTTTGATACTCACGCCTTGTTCGAGACGCTAACTTCTGCGCCCGAAGAGCACGGTTTC
GTGAACGCGAGTGATCCTTGTTTGGACATCAACCGCTCATCGTCTGTCGATTACATGTACACCCACGCAT
TGCGCTCTGAGTGTGCAGCGTCTGGTGCTGAGAAGTTTGTGTTCTGGGATGTCACGCATCCAACAACAGC
AACTCACCGCTATGTTGCAGAGAAAATGCTAGAAAGTAGCAACAACTTAGCTGAGTACCGTTTCTAA

>S114
ATGATGAAAAAAACAATCACACTATTAACTGCATTACTCCCGCTTGCTTCTGCAGTTGCCGAAGAGCCAACCTTAT
CACCAGAAATGGTTTCAGCGTCTGAAGTGATCAGCACGCAAGAAAACCAAACCTATACCTATGTTCGCTGTTGGTA
TCGCACCAGCTACTCGAAAGATGATCCGGCGACCGATTGGGAATGGGCAAAAAACGAAGATGGTAGCTACTTCAC
CATTGACGGCTACTGGTGGAGCTCCGTTTCATTTAAAAACATGTTCTACACCAACACGTCGCAAAACGTTATCCGT
CAGCGTTGTGAAGCAACATTAGATTTGGCGAACGAGAACGCAGACATTACGTTCTTCGCCGCTGACAATCGCTTCT
CATACAACCACACGATCTGGAGCAACGACGCAGCAATGCAGCCAGATCAAATCAACAAAGTGGTTGCACTCGGTG
ACAGCTTGTCTGATACAGGCAACATATTTAACGCATCACAATGGCGCTTCCCTAACCCGAACAGCTGGTTCTTAGG
TCACTTCTCCAACGGTTTTGTGTGGACAGAATACATTGCCAAAGCGAAGAACCTTCCGCTCTACAACTGGGCAGTT
GGCGGCGCGGCTGGTGAGAACCAATACATCGCGCTAACAGGGGTTGGTGATCAAGTTTCTTCGTACTTAACCTACG
CAAAACTGGCGAAGAACTACAAACCAGCAAACACCTTGTTTACGCTTGAGTTTGGTTTGAATGACTTCATGAACTA
CAACCGTGGCGTTCCAGAAGTGAAAGCGGATTATGCAGAAGCACTGATTCGTTTGACGGACGCAGGTGCGAAGAA
CTTCATGTTGATGACACTGCCAGATGCGACGAAAGCGCCTCAGTTTAAGTACTCAACACAAGAAGAGATCGACAA
AATTCGTGCGAAAGTGCTTGAGATGAACGAGTTCATCAAGGCACAAGCGATGTACTACAAAGCGCAAGGTTACAA
CATCACGTTGTTTGATACTCACGCCTTGTTCGAGACGCTAACTTCTGCGCCAGAAGAGCACGGTTTCGTGAACGCG
AGTGATCCTTGTTTGGACATCAACCGCTCATCGTCTGTCGATTACATGTACACCCACGCATTGCGCTCTGAGTGTGC
AGCATCTGGTGCTGAGAAGTTTGTGTTCTGGGATGTCACGCATCCAACAACAGCAACTCACCGCTATGTTGCAGAG
AAAATGCTAGAAAGTAGCAACAACTTAGCAGAGTACCGTTTCTAA

>S113
ATGATGAAAAAAACAATCACACTATTAACTGCATTACTCCCGCTTGCTTCTGCAGTTGCCGAAGAGCCAA
CCTTATCACCAGAAATGGTTTCAGCGTCTGAAGTGATCAGCACGCAAGAAAACCAAACCTATACCTATGT
TCGCTGTTGGTATCGCACCAGCTACTCGAAAGATGATCCGGCGACCGATTGGGAATGGGCAAAAAACGAA
GATGGTAGCTACTTCACCATTGACGGCTACTGGTGGAGCTCCGTTTCATTTAAAAACATGTTCTACACCA
ACACGTCGCAAAACGTTATCCGTCAGCGTTGTGAAGCAACATTAGATTTGGCGAACGAGAACGCAGACAT

TACGTTCTTCGCCGCTGACAATCGCTTCTCATACAACCACACGATCTGGAGCAACGACGCAGCAATGCAG
CCAGATCAAATCAACAAAGTGGTTGCACTCGGTGACAGCTTGTCTGATACAGGCAACATATTTAACGCAT
CACAATGGCGCTTCCCTAACCCGAACAGCTGGTTCTTAGGTCACTTCTCCAACGGTTTTGTGTGGACAGA
ATACATTGCCAAAGCGAAGAACCTTCCGCTCTACAACTGGGCAGTTGGCGGCGCGGCTGGTGAGAACCAA
TACATCGCGCTAACAGGGGTTGGTGATCAAGTTTCTTCGTACTTAACCTACGCAAAACTGGCGAAGAACT
ACAAACCAGCAAACACCTTGTTTACGCTTGAGTTTGGTTTGAATGACTTCATGAACTACAACCGTGGCGT
TCCAGAAGTGAAAGCGGATTATGCAGAAGCACTGATTCGTTTGACGGACGCAGGTGCGAAGAACTTCATG
TTGATGACACTGCCAGATGCGACGAAAGCGCCTCAGTTTAAGTACTCAACACAAGAAGAGATCGACAAAA
TTCGTGCGAAAGTGCTTGAGATGAACGAGTTCATCAAGGCACAAGCGATGTACTACAAAGCGCAAGGTTA
CAACATCACGTTGTTTGATACTCACGCCTTGTTCGAGACGCTAACTTCTGCGCCAGAAGAGCACGGTTTC
GTGAACGCGAGTGATCCTTGTTTGGACATCAACCGCTCATCGTCTGTCGATTACATGTACACCCACGCAT
TGCGCTCTGAGTGTGCAGCATCTGGTGCTGAGAAGTTTGTGTTCTGGGATGTCACGCATCCAACAACAGC
AACTCACCGCTATGTTGCAGAGAAAATGCTAGAAAGTAGCAACAACTTAGCAGAGTACCGTTTCTAA

>S112
ATGATGAAAAAAACAATCACACTATTAACTGCATTACTCCCGCTTGCTTCTGCAGTTGCCGAAGAGCCAACCTTAT
CACCAGAAATGGTTTCAGCGTCTGAAGTGATCAGCACGCAAGAAAACCAAACCTATACCTATGTTCGCTGTTGGTA
TCGCACCAGCTACTCGAAAGATGATCCGGCGACCGATTGGGAATGGGCAAAAAACGAAGATGGTAGCTACTTCAC
CATTGACGGCTACTGGTGGAGCTCCGTTTCATTTAAAAACATGTTCTACACCAACACGTCGCAAAACGTTATCCGT
CAGCGTTGTGAAGCAACATTAGATTTGGCGAACGAGAACGCAGACATTACGTTCTTCGCCGCTGACAATCGCTTCT
CATACAACCACACGATCTGGAGCAACGACGCAGCAATGCAGCCAGATCAAATCAACAAAGTGGTTGCACTCGGTG
ACAGCTTGTCTGATACAGGCAACATATTTAACGCATCACAATGGCGCTTCCCTAACCCGAACAGCTGGTTCTTAGG
TCACTTCTCCAACGGTTTTGTGTGGACAGAATACATTGCCAAAGCGAAGAACCTTCCGCTCTACAACTGGGCAGTT
GGCGGCGCGGCTGGTGAGAACCAATACATCGCGCTAACAGGGGTTGGTGATCAAGTTTCTTCGTACTTAACCTACG
CAAAACTGGCGAAGAACTACAAACCAGCAAACACCTTGTTTACGCTTGAGTTTGGTTTGAATGACTTCATGAACTA
CAACCGTGGCGTTCCAGAAGTGAAAGCGGATTATGCAGAAGCACTGATTCGTTTGACGGACGCAGGTGCGAAGAA
CTTCATGTTGATGACACTGCCAGATGCGACGAAAGCGCCTCAGTTTAAGTACTCAACACAAGAAGAGATCGACAA
AATTCGTGCGAAAGTGCTTGAGATGAACGAGTTCATCAAGGCACAAGCGATGTACTACAAAGCGCAAGGTTACAA
CATCACGTTGTTTGATACTCACGCCTTGTTCGAGACGCTAACTTCTGCGCCAGAAGAGCACGGTTTCGTGAACGCG
AGTGATCCTTGTTTGGACATCAACCGCTCATCGTCTGTCGATTACATGTACACCCACGCATTGCGCTCTGAGTGTGC
AGCATCTGGTGCTGAGAAGTTTGTGTTCTGGGATGTCACGCATCCAACAACAGCAACTCACCGCTATGTTGCAGAG
AAAATGCTAGAAAGTAGCAACAACTTAGCAGAGTACCGTTTCTAA

>S111
ATGATGAAAAAAACAATCACACTATTAACTGCATTACTCCCGCTTGCTTCTGCAGTTGCCGAAGAGCCAACCTTAT
CACCAGAAATGGTTTCAGCGTCTGAAGTGATCAGCACGCAAGAAAACCAAACCTATACCTATGTTCGCTGTTGGTA
TCGCACCAGCTACTCGAAAGATGATCCGGCGACCGATTGGGAATGGGCAAAAAACGAAGATGGTAGCTACTTCAC
CATTGACGGCTACTGGTGGAGCTCCGTTTCATTTAAAAACATGTTCTACACCAACACGTCGCAAAACGTTATCCGT
CAGCGTTGTGAAGCAACATTAGATTTGGCGAACGAGAACGCAGACATTACGTTCTTCGCCGCTGACAATCGCTTCT
CATACAACCACACGATCTGGAGCAACGACGCAGCAATGCAGCCAGATCAAATCAACAAAGTGGTTGCACTCGGTG
ACAGCTTGTCTGATACAGGCAACATATTTAACGCATCACAATGGCGCTTCCCTAACCCGAACAGCTGGTTCTTAGG
TCACTTCTCCAACGGTTTTGTGTGGACAGAATACATTGCCAAAGCGAAGAACCTTCCGCTCTACAACTGGGCAGTT
GGCGGCGCGGCTGGTGAGAACCAATACATCGCGCTAACAGGGGTTGGTGATCAAGTTTCTTCGTACTTAACCTACG
CAAAACTGGCGAAGAACTACAAACCAGCAAACACCTTGTTTACGCTTGAGTTTGGTTTGAATGACTTCATGAACTA
CAACCGTGGCGTTCCAGAAGTGAAAGCGGATTATGCAGAAGCACTGATTCGTTTGACGGACGCAGGTGCGAAGAA
CTTCATGTTGATGACACTGCCAGATGCGACGAAAGCGCCTCAGTTTAAGTACTCAACACAAGAAGAGATCGACAA
AATTCGTGCGAAAGTGCTTGAGATGAACGAGTTCATCAAGGCACAAGCGATGTACTACAAAGCGCAAGGTTACAA
CATCACGTTGTTTGATACTCACGCCTTGTTCGAGACGCTAACTTCTGCGCCAGAAGAGCACGGTTTCGTGAACGCG
AGTGATCCTTGTTTGGACATCAACCGCTCATCGTCTGTCGATTACATGTACACCCACGCATTGCGCTCTGAGTGTGC
AGCATCTGGTGCTGAGAAGTTTGTGTTCTGGGATGTCACGCATCCAACAACAGCAACTCACCGCTATGTTGCAGAG
AAAATGCTAGAAAGTAGCAACAACTTAGCAGAGTACCGTTTCTAA

>S110
ATGATGAAAAAAACAATCACACTATTAACTGCATTACTCCCGCTTGCTTCTGCAGTTGCCGAAGAGCCAA
CCTTATCACCAGAAATGGTTTCAGCGTCTGAAGTGATCAGCACGCAAGAAAACCAAACCTATACCTATGT
TCGCTGTTGGTATCGCACCAGCTACTCGAAAGATGATCCGGCGACCGATTGGGAATGGGCAAAAAACGAA
GATGGTAGCTACTTCACCATTGACGGCTACTGGTGGAGCTCCGTTTCATTTAAAAACATGTTCTACACCA
ACACGTCGCAAAACGTTATCCGTCAGCGTTGTGAAGCAACATTAGATTTGGCGAACGAGAACGCAGACAT
TACGTTCTTCGCCGCTGACAATCGCTTCTCATACAACCACACGATCTGGAGCAACGACGCAGCAATGCAG
CCAGATCAAATCAACAAAGTGGTTGCACTCGGTGACAGCTTGTCTGATACAGGCAACATATTTAACGCAT
CACAATGGCGCTTCCCTAACCCGAACAGCTGGTTCTTAGGTCACTTCTCCAACGGTTTTGTGTGGACAGA
ATACATTGCCAAAGCGAAGAACCTTCCGCTCTACAACTGGGCAGTTGGCGGCGCGGCTGGTGAGAACCAA
TACATCGCGCTAACAGGGGTTGGTGATCAAGTTTCTTCGTACTTAACCTACGCAAAACTGGCGAAGAACT
ACAAACCAGCAAACACCTTGTTTACGCTTGAGTTTGGTTTGAATGACTTCATGAACTACAACCGTGGCGT
TCCAGAAGTGAAAGCGGATTATGCAGAAGCACTGATTCGTTTGACGGACGCAGGTGCGAAGAACTTCATG
TTGATGACACTGCCAGATGCGACGAAAGCGCCTCAGTTTAAGTACTCAACACAAGAAGAGATCGACAAAA
TTCGTGCGAAAGTGCTTGAGATGAACGAGTTCATCAAGGCACAAGCGATGTACTACAAAGCGCAAGGTTA
CAACATCACGTTGTTTGATACTCACGCCTTGTTCGAGACGCTAACTTCTGCGCCAGAAGAGCACGGTTTC
GTGAACGCGAGTGATCCTTGTTTGGACATCAACCGCTCATCGTCTGTCGATTACATGTACACCCACGCAT
TGCGCTCTGAGTGTGCAGCATCTGGTGCTGAGAAGTTTGTGTTCTGGGATGTCACGCATCCAACAACAGC
AACTCACCGCTATGTTGCAGAGAAAATGCTAGAAAGTAGCAACAACTTAGCAGAGTACCGTTTCTAA

>S109
ATGATGAAAAAAACAATCACACTATTAACTGCATTACTCCCGCTTGCTTCTGCAGTTGCCGAAGAGCCAA
CCTTATCACCAGAAATGGTTTCAGCGTCTGAAGTGATCAGCACGCAAGAAAACCAAACCTATACCTATGT
TCGCTGTTGGTATCGCACCAGCTACTCGAAAGATGATCCGGCGACCGATTGGGAATGGGCAAAAAACGAA
GATGGTAGCTACTTCACCATTGACGGCTACTGGTGGAGCTCCGTTTCATTTAAAAACATGTTCTACACCA
ACACGTCGCAAAACGTTATCCGTCAGCGTTGTGAAGCAACATTAGATTTGGCGAACGAGAACGCAGACAT
TACGTTCTTCGCCGCTGACAATCGCTTCTCATACAACCACACGATCTGGAGCAACGACGCAGCAATGCAG
CCAGATCAAATCAACAAAGTGGTTGCACTCGGTGACAGCTTGTCTGATACAGGCAACATCTTTAACGCAT
CACAATGGCGCTTCCCTAACCCGAACAGCTGGTTCTTAGGTCACTTCTCCAACGGTTTTGTGTGGACAGA
ATACATTGCCAAAGCGAAGAACCTTCCGCTCTACAACTGGGCAGTTGGCGGCGCGGCTGGTGAGAACCAA
TACATCGCGCTAACAGGGGTTGGTGAGCAAGTTTCTTCGTACTTAACCTACGCAAAACTGGCGAAGAACT
ACAAACCAGCAAACACCTTGTTTACGCTTGAGTTTGGTTTGAATGACTTCATGAACTACAACCGTGGCGT
TCCAGAAGTGAAAGCGGATTATGCAGAAGCACTGATTCGTTTGACGGACGCAGGTGCGAAGAACTTCATG
TTGATGACACTGCCAGATGCGACGAAAGCGCCTCAGTTTAAGTACTCAACACAAGAAGAGATCGACAAAA
TTCGTGCGAAAGTGCTTGAGATGAACGAGTTCATCAAGGCACAAGCGATGTACTACAAAGCGCAAGGTTA
CAACATCACGTTGTTTGATACTCACGCCTTGTTCGAGACGCTAACTTCTGCGCCAGAAGAGCACGGTTTC
GTGAACGCGAGCGATCCTTGTTTGGACATCAACCGCTCATCGTCTGTCGATTACATGTACACCCACGCAT
TGCGCTCTGAGTGTGCAGCGTCTGGTGCTGAGAAGTTTGTGTTCTGGGATGTCACGCATCCAACAACAGC
AACTCACCGCTATGTTGCAGAGAAAATGCTAGAAAGTAGCAACAACTTAGCCGAGTACCGTTTCTAA

>S108
ATGATGAAAAAAACAATCACACTATTAACTGCATTACTCCCGCTTGCTTCTGCAGTTGCCGAAGAGCCAACCTTAT
CACCAGAAATGGTTTCAGCGTCTGAAGTGATCAGCACGCAAGAAAACCAAACCTATACCTATGTTCGCTGTTGGTA
TCGCACCAGCTACTCGAAAGATGATCCGGCGACCGATTGGGAATGGGCAAAAAACGAAGATGGTAGCTACTTCAC
CATTGACGGCTACTGGTGGAGCTCCGTTTCATTTAAAAACATGTTCTACACCAACACGTCGCAAAACGTTATCCGT
CAGCGTTGTGAAGCAACATTAGATTTGGCGAACGAGAACGCAGACATTACGTTCTTCGCCGCTGACAATCGCTTCT
CATACAACCACACGATCTGGAGCAACGACGCAGCAATGCAGCCAGATCAAATCAACAAAGTGGTTGCACTCGGTG
ACAGCTTGTCTGATACAGGCAACATCTTTAACGCATCACAATGGCGCTTCCCCAACCCGAACAGCTGGTTCTTAGG
TCACTTCTCCAACGGTTTTGTTTGGACAGAATACATTGCCAAAGCGAAGAACCTTCCGCTCTACAACTGGGCAGTT
GGCGGCGCGGCTGGTGAGAACCAATACATCGCGCTAACAGGGGTTGGTGAGCAAGTTTCTTCGTACTTAACCTACG

CAAAACTGGCGAAGAACTACAAACCAGCAAACACCTTGTTTACGCTTGAGTTTGGTTTGAATGACTTCATGAACTA
CAACCGTGGCGTTCCAGAAGTGAAAGCAGATTATGCAGAAGCACTGATTCGTTTGACGGACGCAGGTGCGAAGAA
CTTCATGTTGATGACACTGCCAGACGCGACGAAAGCGCCTCAGTTTAAGTACTCAACACAAGAAGAGATCGACAA
AATTCGTGCGAAAGTGCTTGAGATGAACGAGTTCATCAAGGCACAAGCGATGTACTACAAAGCGCAAGGTTACAA
CATCACGTTGTTTGATACTCACGCCTTGTTCGAGACGCTAACTTCTGCGCCAGAAGAGCACGGTTTCGTGAACGCG
AGCGATCCTTGTTTGGACATCAACCGCTCATCGTCTGTCGATTACATGTACACCCACGCATTGCGCTCTGAGTGTGC
GGCGTCTGGTGCTGAGAAGTTTGTATTCTGGGATGTCACGCATCCAACAACAGCAACTCACCGCTATGTTGCAGAG
AAAATGCTAGAAAGTAGCAACAACTTAGCAGAGTACCGTTTCTAA

>S106
ATGATGAAAAAAACAATCACACTATTAACTGCATTACTCCCGCTTGCTTCTGCAGTTGCCGAAGAGCCAACCTTAT
CACCAGAAATGGTTTCAGCGTCTGAAGTGATCAGCACGCAAGAAAACCAAACCTATACCTATGTTCGCTGTTGGTA
TCGCACCAGCTACTCGAAAGATGATCCGGCGACCGATTGGGAATGGGCAAAAAACGAAGATGGTAGCTACTTCAC
CATTGACGGCTACTGGTGGAGCTCCGTTTCATTTAAAAACATGTTCTACACCAACACGTCGCAAAACGTTATCCGT
CAGCGTTGTGAAGCAACATTAGATTTGGCGAACGAGAACGCAGACATTACGTTCTTCGCCGCTGACAATCGCTTCT
CATACAACCACACGATCTGGAGCAACGACGCAGCAATGCAGCCAGATCAAATCAACAAAGTGGTTGCACTCGGTG
ACAGCTTGTCTGATACAGGCAACATCTTTAACGCATCACAATGGCGCTTCCCCAACCCGAACAGCTGGTTCTTAGG
TCACTTCTCCAACGGTTTTGTTTGGACAGAATACATTGCCAAAGCGAAGAACCTTCCGCTCTACAACTGGGCAGTT
GGCGGCGCGGCTGGTGAGAACCAATACATCGCGCTAACAGGGGTTGGTGAGCAAGTTTCTTCGTACTTAACCTACG
CAAAACTGGCGAAGAACTACAAACCAGCAAACACCTTGTTTACGCTTGAGTTTGGTTTGAATGACTTCATGAACTA
CAACCGTGGCGTTCCAGAAGTGAAAGCAGATTATGCAGAAGCACTGATTCGTTTGACGGACGCAGGTGCGAAGAA
CTTCATGTTGATGACACTGCCAGACGCGACGAAAGCGCCTCAGTTTAAGTACTCAACACAAGAAGAGATCGACAA
AATTCGTGCGAAAGTGCTTGAGATGAACGAGTTCATCAAGGCACAAGCGATGTACTACAAAGCGCAAGGTTACAA
CATCACGTTGTTTGATACTCACGCCTTGTTCGAGACGCTAACTTCTGCGCCAGAAGAGCACGGTTTCGTGAACGCG
AGCGATCCTTGTTTGGACATCAACCGCTCATCGTCTGTCGATTACATGTACACCCACGCATTGCGCTCTGAGTGTGC
GGCGTCTGGTGCTGAGAAGTTTGTATTCTGGGATGTCACGCATCCAACAACAGCAACTCACCGCTATGTTGCAGAG
AAAATGCTAGAAAGTAGCAACAACTTAGCAGAGTACCGTTTCTAA

>S105
ATGATGAAAAAAACAATCACACTATTAACTGCATTACTCCCGCTTGCTTCTGCAGTTGCCGAAGAGCCAA
CCTTATCACCAGAAATGGTTTCAGCGTCTGAAGTGATCAGCACGCAAGAAAACCAAACCTATACCTATGT
TCGCTGTTGGTATCGCACCAGCTACTCGAAAGATGATCCGGCGACCGATTGGGAATGGGCAAAAAACGAA
GATGGTAGCTACTTCACCATTGACGGCTACTGGTGGAGCTCCGTTTCATTTAAAAACATGTTCTACACCA
ACACGTCGCAAAACGTTATCCGTCAGCGTTGTGAAGCCACATTAGATTTGGCGAACGAGAACGCAGACAT
TACGTTCTTCGCCGCTGACAATCGCTTCTCATACAACCACACGATCTGGAGCAACGACGCAGCAATGCAG
CCAGATCAAATCAACAAAGTGGTTGCACTCGGTGACAGCTTGTCTGATACAGGCAACATCTTTAACGCAT
CACAATGGCGCTTCCCTAACCCGAACAGCTGGTTCTTAGGTCACTTCTCCAACGGTTTTGTGTGGACAGA
ATACATTGCCAAAGCGAAGAACCTTCCGCTCTACAACTGGGCAGTTGGCGGCGCGGCTGGTGAGAACCAA
TACATCGCGCTAACAGGGGTTGGTGATCAAGTTTCTTCGTACTTAACCTACGCAAAACTGGCGAAGAACT
ACAAACCAGCAAACACCTTGTTTACGCTTGAGTTTGGTTTGAATGACTTCATGAACTACAACCGTGGCGT
TCCAGAAGTGAAAGCGGATTATGCAGAAGCACTGATTCGTTTGACGGACGCAGGTGCGAAGAACTTCATG
TTGATGACACTGCCAGACGCGACGAAAGCGCCTCAGTTTAAGTACTCAACACAAGAAGAGATCGACAAAA
TTCGTGCGAAAGTGCTTGAGATGAACGAGTTCATCAAGGCACAAGCGATGTACTACAAAGCGCAAGGTTA
CAACATCACGTTGTTTGATACTCACGCCTTGTTCGAGACGCTAACTTCTGCACCCGAAGAGCACGGTTTC
GTGAACGCGAGTGATCCTTGTTTGGACATCAACCGCTCATCGTCTGTCGATTACATGTACACCCACGCAT
TGCGCTCTGAGTGTGCGGCGTCTGGTGCTGAGAAGTTTGTGTTCTGGGATGTCACGCACCCAACAACAGC
AACTCACCGCTATGTTGCAGAGAAAATGCTAGAAAGTAGCAACAACTTAGCAGAGTACCGTTTCTAA

>S104
ATGATGAAAAAAACAATCACACTATTAACTGCATTACTCCCGCTTGCTTCTGCAGTTGCCGAAGAGCCAACCTTAT
CACCAGAAATGGTTTCAGCGTCTGAAGTGATCAGCACGCAAGAAAACCAAACCTATACCTATGTTCGCTGTTGGTA

TCGCACCAGCTACTCGAAAGATGATCCGGCGACCGGTTGGGAATGGGCAAAAAACGAAGATGGTAGCTACTTCAC
CATTGACGGCTACTGGTGGAGCTCCGTTTCATTTAAAAACATGTTCTACACCAACACGTCGCAAAACGTTATCCGT
CAGCGTTGTGAAGCAACATTAGATTTGGCGAACGAGAACGCAGACATTACGTTCTTCGCCGCTGACAATCGCTTCT
CATACAACCACACGATCTGGAGCAACGACGCAGCAATGCAGCCAGATCAAATCAACAAAGTGGTTGCACTCGGTG
ACAGCTTGTCTGATACAGGCAACATCTTTAACGCATCACAATGGCGCTTCCCTAACCCGAACAGCTGGTTCTTAGG
TCACTTCTCGAACGGTTTTGTGTGGACAGAATACATTGCCAAAGCGAAGAACCTTCCGCTCTACAACTGGGCAGTT
GGCGGCGCGGCTGGTGAGAACCAATACATCGCGCTAACAGGGGTTGGTGATCAAGTTTCTTCGTACTTAACCTACG
CAAAACTGGCGAAGAACTACAAACCAGCAAACACCTTGTTTACGCTTGAGTTTGGTTTGAATGACTTCATGAACTA
CAACCGTGGCGTTCCAGAAGTGAAAGCGGATTATGCAGAAGCACTGATTCGTTTGACGGACGCAGGTGCGAAGAA
CTTCATGTTGATGACACTGCCAGACGCGACGAAAGCGCCTCAGTTTAAGTACTCAACACAAGAAGAGATCGACAA
AATTCGTGCGAAAGTGCTTGAGATGAACGAGTTCATCAAGGCACAAGCGATGTACTACAAAGCGCAAGGTTACAA
CATCACGTTGTTTGATACTCACGCCTTGTTCGAGACGCTAACTTCTGCGCCAGAAGAGCACGGTTTCGTGAACGCG
AGCGATCCTTGTTTGGACATCAACCGCTCATCGTCTGTCGACTACATGTACACCCACGCATTGCGCTCTGAGTGTGC
GGCGTCTGGTGCTGAGAAGTTTGTATTCTGGGATGTCACGCACCCAACAACAGCAACTCACCGCTATGTTGCAGAG
AAAATGCTAGAAAGTAGCAACAACTTAGCCGAGTACCGTTTCTAA

>S101
ATGATGAAAAAAACAATCACACTATTAACTGCATTACTCCCGCTTGCTTCTGCAGTTGCCGAAGAGCCAACCTTAT
CACCAGAAATGGTTTCAGCGTCTGAAGTGATCAGCACGCAAGAAAACCAAACCTATACCTATGTTCGCTGTTGGTA
TCGCACCAGCTACTCGAAAGATGATCCGGCGACCGATTGGGAATGGGCAAAAAACGAAGATGGTAGCTACTTCAC
CATTGACGGCTACTGGTGGAGCTCCGTTTCATTTAAAAACATGTTCTACACCAACACGTCGCAAAACGTTATCCGT
CAGCGTTGTGAAGCAACATTAGATTTGGCGAACGAGAACGCAGACATTACGTTCTTCGCCGCTGACAATCGCTTCT
CATACAACCACACGATCTGGAGCAACGACGCAGCAATGCAGCCAGATCAAATCAACAAAGTGGTTGCACTCGGTG
ACAGCTTGTCTGATACAGGCAACATCTTTAACGCATCACAATGGCGCTTCCCTAACCCGAACAGCTGGTTCTTAGG
TCACTTCTCCAACGGTTTTGTGTGGACAGAATACATTGCCAAAGCGAAGAACCTTCCGCTCTACAACTGGGCAGTT
GGCGGTGCGGCTGGTGAGAACCAATACATCGCGCTAACAGGGGTTGGTGATCAAGTTTCTTCGTACTTAACCTACG
CAAAACTGGCGAAGAACTACAAACCAGCAAACACCTTGTTTACGCTTGAGTTTGGTTTGAATGACTTCATGAACTA
CAACCGTGGCGTTCCAGAAGTGAAAGCGGATTATGCAGAAGCACTGATTCGTTTGACGGACGCAGGTGCGAAGAA
CTTCATGTTGATGACACTGCCAGATGCGACGAAAGCGCCTCAGTTTAAGTACTCAACACAAGAAGAGATCGACAA
AATTCGTGCGAAAGTGCTTGAGATGAACGAGTTCATCAAGGCACAAGCGATGTACTACAAAGCGCAAGGTTACAA
CATCACGTTGTTTGATACTCACGCCTTGTTCGAGACGCTAACTTCTGCGCCAGAAGAGCACGGTTTCGTGAACGCG
AGCGATCCTTGTTTGGACATCAACCGCTCATCGTCTGTCGATTACATGTACACCCACGCATTGCGCTCTGAGTGTGC
AGCGTCTGGTGCTGAGAAGTTTGTGTTCTGGGATGTCACGCACCCAACAACAGCAACTCACCGCTATGTTGCAGAG
AAAATGCTAGAAAGTAGCAACAACTTAGAAGAGTTTCGCTTTTAA

>S100
ATGATGAAAAAAACAATCACACTATTAACTGCATTACTCCCGCTTGCTTCTGCAGTTGCCGAAGAGCCAACCTTAT
CACCAGAAATGGTTTCAGCGTCTGAAGTGATCAGCACGCAAGAAAACCAAACCTATACCTATGTTCGCTGTTGGTA
TCGCACCAGCTACTCGAAAGATGATCCGGCGACCGATTGGGAATGGGCAAAAAACGAAGATGGTAGCTACTTCAC
CATTGACGGCTACTGGTGGAGCTCCGTTTCATTTAAAAACATGTTCTACACCAACACGTCGCAAAACGTTATCCGT
CAGCGTTGTGAAGCAACATTAGATTTGGCGAACGAGAACGCAGATATTACGTTCTTCGCCGCTGACAATCGCTTCT
CATACAACCACACGATCTGGAGCAACGACGCAGCAATGCAGCCAGATCAAATCAACAAAGTGGTTGCACTCGGTG
ACAGCTTGTCTGATACAGGCAACATCTTTAACGCATCACAATGGCGCTTCCCTAACCCGAACAGCTGGTTCTTAGG
TCACTTCTCCAACGGTTTTGTGTGGACAGAATACATTGCCAAAGCGAAGAACCTTCCGCTCTACAACTGGGCAGTT
GGCGGCGCGGCTGGTGAGAACCAATACATCGCGCTAACAGGGGTTGGTGATCAAGTTTCTTCGTACTTAACCTACG
CAAAACAGGCGAAGAACTACAAACCAGCAAACACCTTGTTTACGCTTGAGTTTGGTTTGAATGACTTCATGAACTA
CAACCGTGGCGTTCCAGAAGTGAAAGCGGATTATGCAGAAGCACTGATTCGTTTGACGGACGCAGGTGCGAAGAA
CTTCATGTTGATGACACTGCCAGACGCGACGAAAGCGCCTCAGTTTAAGTACTCAACACAAGAAGAGATCGACAA
AATTCGTGCGAAAGTGCTTGAGATGAACGAGTTCATCAAGGCACAAGCGATGTACTACAAAGCGCAAGGTTACAA
CATCACGTTGTTTGATACTCACGCCTTGTTCGAGACGCTAACTTCTGCGCCAGAAGAGCACGGTTTCGTGAACGCG
AGCGATCCTTGTTTGGACATCAACCGCTCATCGTCTGTCGATTACATGTACACCCACGCATTGCGCTCTGAGTGTGC

AGCGTCTGGTGCTGAGAAGTTTGTGTTCTGGGATGTGACTCACCCAACTACAGCCACGCATCGTTATGTTGCTGAA
AAAATGTTGGAAAGCAGCAATAACTTAGAAGAGTTTCGCTTTTAA

>S098
ATGATGAAAAAAACAATCACACTATTAACTGCATTACTCCCGCTTGCTTCTGCAGTTGCCGAAGAGCCAACCTTAT
CACCAGAAATGGTTTCAGCGTCTGAAGTGATCAGCACGCAAGAAAACCAAACCTATACCTATGTTCGCTGTTGGTA
TCGCACCAGCTACTCGAAAGATGATCCGGCGACCGATTGGGAATGGGCAAAAAACGAAGATGGTAGCTACTTCAC
CATTGACGGCTACTGGTGGAGCTCCGTTTCATTTAAAAACATGTTCTACACCAACACGTCGCAAAACGTTATCCGT
CAGCGTTGTGAAGCCACATTAGATTTGGCGAACGAGAACGCAGACATTACGTTCTTCGCCGCTGACAATCGCTTCT
CATACAACCACACGATCTGGAGCAACGACGCAGCAATGCAGCCAGATCAAATCAACAAAGTGGTTGCTCTCGGTG
ACAGCTTGTCTGATACAGGCAACATCTTTAACGCATCACAATGGCGCTTCCCTAACCCGAACAGCTGGTTCTTAGG
TCACTTCTCCAACGGTTTTGTGTGGACAGAATACATTGCCAAAGCGAAGAACCTTCCGCTCTACAACTGGGCAGTT
GGCGGTGCGGCTGGTGAGAACCAATACATCGCGCTAACAGGGGTTGGTGATCAAGTTTCTTCGTACTTAACCTACG
CAAAACTGGCGAAGAACTACAAACCAGCAAACACCTTGTTTACGCTTGAGTTTGGTTTGAATGACTTCATGAACTA
CAACCGTGGCGTTCCAGAAGTGAAAGCGGATTATGCAGAAGCACTGATTCGTTTGACGGACGCAGGTGCGAAGAA
CTTCATGTTGATGACACTGCCAGATGCGACGAAAGCGCCTCAGTTTAAGTACTCAACACAAGAAGAGATCGACAA
AATTCGTGCGAAAGTGCTTGAGATGAACGAGTTCATCAAGGCACAAGCGATGTACTACAAAGCGCAAGGTTACAA
CATCACGTTGTTTGATACTCACGCCTTGTTCGAGACGCTAACTTCTGCGCCAGAAGAGCACGGTTTCGTGAACGCG
AGCGATCCTTGTTTGGACATCAACCGCTCATCGTCTGTCGATTACATGTACACCCACGCATTGCGCTCTGAGTGTGC
AGCGTCTGGTGCTGAGAAGTTTGTGTTCTGGGATGTCACGCACCCAACAACAGCAACTCACCGCTATGTTGCAGAG
AAAATGCTAGAAAGTAGCAACAACTTAGCAGAGTACCGTTTCTAA

>S097
ATGATGAAAAAAACAATCACACTATTAACTGCATTACTCCCGCTTGCTTCTGCAGTTGCCGAAGAGCCAACCTTAT
CACCAGAAATGGTTTCAGCGTCTGAAGTGATCAGCACGCAAGAAAACCAAACCTATACCTATGTTCGCTGTTGGTA
TCGCACCAGCTACTCGAAAGATGATCCGGCGACCGATTGGGAATGGGCAAAAAACGAAGATGGTAGCTACTTCAC
CATTGACGGCTACTGGTGGAGCTCCGTTTCATTTAAAAACATGTTCTACACCAACACGTCGCAAAACGTTATCCGT
CAGCGTTGTGAAGCAACATTAGATTTGGCGAACGAGAACGCAGACATTACGTTCTTCGCCGCTGACAATCGCTTCT
CATACAACCACACGATCTGGAGCAACGACGCAGCAATGCAGCCAGATCAAATCAACAAAGTGGTTGCACTCGGTG
ACAGCTTGTCTGATACAGGCAACATCTTTAACGCATCACAATGGCGCTTCCCTAACCCGAACAGCTGGTTCTTAGG
TCACTTCTCCAACGGTTTTGTGTGGACAGAATACATTGCCAAAGCGAAGAACCTTCCGCTCTACAACTGGGCAGTT
GGCGGCGCGGCTGGTGAGAACCAATACATCGCGCTAACAGGGGTTGGTGATCAAGTTTCTTCGTACTTAACCTACG
CAAAACTGGCGAAGAACTACAAACCAGCAAACACCTTGTTTACGCTTGAGTTTGGTTTGAATGACTTCATGAACTA
CAACCGTGGCGTTCCAGAAGTGAAAGCGGATTATGCAGAAGCACTGATTCGTTTGACGGACGCAGGTGCGAAGAA
CTTCATGTTGATGACACTGCCAGACGCGACGAAAGCGCCTCAGTTTAAGTACTCAACACAAGAAGAGATCGACAA
AATTCGTGCGAAAGTGCTTGAGATGAACGAGTTCATCAAGGCACAAGCGATGTACTACAAAGCGCAAGGTTACAA
CATCACGTTGTTTGATACTCACGCCTTGTTCGAGACGCTAACTTCTGCGCCAGAAGAGCACGGTTTCGTGAACGCG
AGCGATCCTTGTTTGGACATCAACCGCTCATCGTCTGTCGATTACATGTACACCCACGCATTGCGCTCTGAGTGTGC
AGCGTCTGGTGCTGAGAAGTTTGTGTTCTGGGATGTCACGCATCCAACAACAGCAACTCACCGCTATGTTGCAGAG
AAAATGCTAGAAAGTAGCAACAACTTAGCCGAGTACCGTTTCTAA

>S095
ATGATGAAAAAAACAATCACACTATTAACTGCATTACTCCCGCTTGCTTCTGCAGTTGCCGAAGAGCCAACCTTAT
CACCAGAAATGGTTTCAGCGTCTGAAGTGATCAGCACGCAAGAAAACCAAACCTATACCTATGTTCGCTGTTGGTA
TCGCACCAGCTACTCGAAAGATGATCCAGCGACCGATTGGGAATGGGCAAAAAACGAAGATGGTAGCTACTTCAC
CATTGACGGCTACTGGTGGAGCTCCGTTTCATTTAAAAACATGTTCTACACCAACACGTCGCAAAACGTTATCCGT
CAGCGTTGTGAAGCAACATTAGATTTGGCGAACGAGAACGCAGACATTACGTTCTTCGCCGCTGACAATCGCTTCT
CATACAACCACACGATCTGGAGCAACGACGCAGCAATGCAGCCAGATCAAATCAACAAAGTGGTTGCACTCGGTG
ACAGCTTGTCTGATACAGGCAACATCTTTAACGCATCACAATGGCGCTTCCCTAACCCGAACAGCTGGTTCTTAGG
TCACTTCTCCAACGGTTTTGTTTGGACAGAATACATTGCCAAAGCGAAGAACCTTCCGCTCTACAACTGGGCAGTT
GGCGGCGCGGCTGGTGAGAACCAATACATCGCGCTAACAGGGGTTGGTGAGCAAGTTTCTTCGTACTTAACCTACG

CAAAACTGGCAAAGAACTACAAACCAGCAAACACCTTGTTTACGCTTGAGTTTGGTTTGAATGACTTCATGAACTA
CAACCGTGGCGTTCCAGAAGTGAAAGCGGATTATGCAGAAGCACTGATTCGTTTGACGGACGCAGGTGCGAAGAA
CTTCATGTTGATGACACTGCCAGATGCGACGAAAGCGCCTCAGTTTAAGTACTCAACACAAGAAGAGATCGACAA
AATTCGTGCGAAAGTGCTTGAGATGAACGAGTTCATCAAGGCACAAGCGATGTACTACAAAGCGCAAGGTTACAA
CATCACGTTGTTTGATACTCACGCCTTGTTCGAGACGCTAACTTCTGCGCCAGAAGAGCACGGTTTCGTGAACGCG
AGCGATCCTTGTTTGGACATCAACCGCTCATCGTCTGTCGATTACATGTACACCCACGCATTGCGCTCTGAGTGTGC
AGCGTCTGGTGCTGAGAAGTTTGTGTTCTGGGATGTCACGCATCCAACAACAGCAACTCACCGCTATGTTGCAGAG
AAAATGCTAGAAAGTAGCAACAACTTAGCCGAGTACCGTTTCTAA

>S094
ATGATGAAAAAAACAATCACACTATTAACTGCATTACTCCCGCTTGCTTCTGCAGTTGCCGAAGAGCCAACCTTAT
CACCAGAAATGGTTTCAGCGTCTGAAGTGATCAGCACGCAAGAAAACCAAACCTATACCTATGTTCGCTGTTGGTA
TCGCACCAGCTACTCGAAAGATGATCCAGCGACCGATTGGGAATGGGCAAAAAACGAAGATGGTAGCTACTTCAC
CATTGACGGCTACTGGTGGAGCTCCGTTTCATTTAAAAACATGTTCTACACCAACACGTCGCAAAACGTTATCCGT
CAGCGTTGTGAAGCAACATTAGATTTGGCGAACGAGAACGCAGACATTACGTTCTTCGCCGCTGACAATCGCTTCT
CATACAACCACACGATCTGGAGCAACGACGCAGCAATGCAGCCAGATCAAATCAACAAAGTGGTTGCACTCGGTG
ACAGCTTGTCTGATACAGGCAACATCTTTAACGCATCACAATGGCGCTTCCCTAACCCGAACAGCTGGTTCTTAGG
TCACTTCTCCAACGGTTTTGTTTGGACAGAATACATTGCCAAAGCGAAGAACCTTCCGCTCTACAACTGGGCAGTT
GGCGGCGCGGCTGGTGAGAACCAATACATCGCGCTAACAGGGGTTGGTGAGCAAGTTTCTTCGTACTTAACCTACG
CAAAACTGGCGAAGAACTACAAACCAGCAAACACCTTGTTTACGCTTGAGTTTGGTTTGAATGACTTCATGAACTA
CAACCGTGGCGTTCCAGAAGTGAAAGCGGATTATGCAGAAGCACTGATTCGTTTGACGGACGCAGGTGCGAAGAA
CTTCATGTTGATGACACTGCCAGATGCGACGAAAGCGCCTCAGTTTAAGTACTCAACACAAGAAGAGATCGACAA
AATTCGTGCGAAAGTGCTTGAGATGAACGAGTTCATCAAGGCACAAGCGATGTACTACAAAGCGCAAGGTTACAA
CATCACGTTGTTTGATACTCACGCCTTGTTCGAGACGCTAACTTCTGCGCCAGAAGAGCACGGTTTCGTGAACGCG
AGCGATCCTTGTTTGGACATCAACCGCTCATCGTCTGTCGATTACATGTACACCCACGCATTGCGCTCTGAGTGTGC
AGCGTCTGGTGCTGAGAAGTTTGTGTTCTGGGATGTCACGCATCCAACAACAGCAACTCACCGCTATGTTGCAGAG
AAAATGCTAGAAAGTAGCAACAACTTAGCCGAGTACCGTTTCTAA

>S093
ATGATGAAAAAAACAATCACACTATTAACTGCATTACTCCCGCTTGCTTCTGCAGTTGCCGAAGAGCCAACCTTAT
CACCAGAAATGGTTTCAGCGTCTGAAGTGATCAGCACGCAAGAAAACCAAACCTATACCTATGTTCGCTGTTGGTA
TCGCACCAGCTACTCGAAAGATGATCCAGCGACCGATTGGGAATGGGCAAAAAACGAAGATGGTAGCTACTTCAC
CATTGACGGCTACTGGTGGAGCTCCGTTTCATTTAAAAACATGTTCTACACCAACACGTCGCAAAACGTTATCCGT
CAGCGTTGTGAAGCAACATTAGATTTGGCGAACGAGAACGCAGACATTACGTTCTTCGCCGCTGACAATCGCTTCT
CATACAACCACACGATCTGGAGCAACGACGCAGCAATGCAGCCAGATCAAATCAACAAAGTGGTTGCACTCGGTG
ACAGCTTGTCTGATACAGGCAACATCTTTAACGCATCACAATGGCGCTTCCCTAACCCGAACAGCTGGTTCTTAGG
TCACTTCTCCAACGGTTTTGTTTGGACAGAATACATTGCCAAAGCGAAGAACCTTCCGCTCTACAACTGGGCAGTT
GGCGGCGCGGCTGGTGAGAACCAATACATCGCGCTAACAGGGGTTGGTGAGCAAGTTTCTTCGTACTTAACCTACG
CAAAACTGGCGAAGAACTACAAACCAGCAAACACCTTGTTTACGCTTGAGTTTGGTTTGAATGACTTCATGAACTA
CAACCGTGGCGTTCCAGAAGTGAAAGCGGATTATGCAGAAGCACTGATTCGTTTGACGGACGCAGGTGCGAAGAA
CTTCATGTTGATGACACTGCCAGATGCGACGAAAGCGCCTCAGTTTAAGTACTCAACACAAGAAGAGATCGACAA
AATTCGTGCGAAAGTGCTTGAGATGAACGAGTTCATCAAGGCACAAGCGATGTACTACAAAGCGCAAGGTTACAA
CATCACGTTGTTTGATACTCACGCCTTGTTCGAGACGCTAACTTCTGCGCCAGAAGAGCACGGTTTCGTGAACGCG
AGCGATCCTTGTTTGGACATCAACCGCTCATCGTCTGTCGATTACATGTACACCCACGCATTGCGCTCTGAGTGTGC
AGCGTCTGGTGCTGAGAAGTTTGTGTTCTGGGATGTCACGCATCCAACAACAGCAACTCACCGCTATGTTGCAGAG
AAAATGCTAGAAAGTAGCAACAACTTAGCCGAGTACCGTTTCTAA

>S091
ATGATGAAAAAAACAATCACACTATTAACTGCATTACTCCCGCTTGCTTCTGCAGTTGCCGAAGAGCCAA
CCTTATCACCAGAAATGGTTTCAGCGTCTGAAGTGATCAGCACGCAAGAAAACCAAACCTATACCTATGT
TCGCTGTTGGTATCGCACCAGCTACTCGAAAGATGATCCAGCGACCGATTGGGAATGGGCAAAAAACGAA

GATGGTAGCTACTTCACCATTGACGGCTACTGGTGGAGCTCCGTTTCATTTAAAAACATGTTCTACACCA
ACACGTCGCAAAACGTTATCCGTCAGCGTTGTGAAGCAACATTAGATTTGGCGAACGAGAACGCAGACAT
TACGTTCTTCGCCGCTGACAATCGCTTCTCATACAACCACACGATCTGGAGCAACGACGCAGCAATGCAG
CCAGATCAAATCAACAAAGTGGTTGCACTCGGTGACAGCTTGTCTGATACAGGCAACATCTTTAACGCAT
CACAATGGCGCTTCCCTAACCCGAACAGCTGGTTCTTAGGTCACTTCTCCAACGGTTTTGTTTGGACAGA
ATACATTGCCAAAGCGAAGAACCTTCCGCTCTACAACTGGGCAGTTGGCGGCGCGGCTGGTGAGAACCAA
TACATCGCGCTAACAGGGGTTGGTGAGCAAGTTTCTTCGTACTTAACCTACGCAAAACTGGCGAAGAACT
ACAAACCAGCAAACACCTTGTTTACGCTTGAGTTTGGTTTGAATGACTTCATGAACTACAACCGTGGCGT
TCCAGAAGTGAAAGCGGATTATGCAGAAGCACTGATTCGTTTGACGGACGCAGGTGCGAAGAACTTCATG
TTGATGACACTGCCAGATGCGACGAAAGCGCCTCAGTTTAAGTACTCAACACAAGAAGAGATCGACAAAA
TTCGTGCGAAAGTGCTTGAGATGAACGAGTTCATCAAGGCACAAGCGATGTACTACAAAGCGCAAGGTTA
CAACATCACGTTGTTTGATACTCACGCCTTGTTCGAGACGCTAACTTCTGCGCCAGAAGAGCACGGTTTC
GTGAACGCGAGCGATCCTTGTTTGGACATCAACCGCTCATCGTCTGTCGATTACATGTACACCCACGCAT
TGCGCTCTGAGTGTGCAGCGTCTGGTGCTGAGAAGTTTGTGTTCTGGGATGTCACGCATCCAACAACAGC
AACTCACCGCTATGTTGCAGAGAAAATGCTAGAAAGTAGCAACAACTTAGCCGAGTACCGTTTCTAA

>S090
ATGATGAAAAAAACAATCACACTATTAACTGCATTACTCCCGCTTGCTTCTGCAGTTGCCGAAGAGCCAACCTTAT
CACCAGAAATGGTTTCAGCGTCTGAAGTGATCAGCACGCAAGAAAACCAAACCTATACCTATGTTCGCTGTTGGTA
TCGCACCAGCTACTCGAAAGATGATCCAGCGACCGATTGGGAATGGGCAAAAAACGAAGATGGTAGCTACTTCAC
CATTGACGGCTACTGGTGGAGCTCCGTTTCATTTAAAAACATGTTCTACACCAACACGTCGCAAAACGTTATCCGT
CAGCGTTGTGAAGCAACATTAGATTTGGCGAACGAGAACGCAGACATTACGTTCTTCGCCGCTGACAATCGCTTCT
CATACAACCACACGATCTGGAGCAACGACGCAGCAATGCAGCCAGATCAAATCAACAAAGTGGTTGCACTCGGTG
ACAGCTTGTCTGATACAGGCAACATCTTTAACGCATCACAATGGCGCTTCCCTAACCCGAACAGCTGGTTCTTAGG
TCACTTCTCCAACGGTTTTGTTTGGACAGAATACATTGCCAAAGCGAAGAACCTTCCGCTCTACAACTGGGCAGTT
GGCGGCGCGGCTGGTGAGAACCAATACATCGCGCTAACAGGGGTTGGTGAGCAAGTTTCTTCGTACTTAACCTACG
CAAAACTGGCGAAGAACTACAAACCAGCAAACACCTTGTTTACGCTTGAGTTTGGTTTGAATGACTTCATGAACTA
CAACCGTGGCGTTCCAGAAGTGAAAGCGGATTATGCAGAAGCACTGATTCGTTTGACGGACGCAGGTGCGAAGAA
CTTCATGTTGATGACACTGCCAGATGCGACGAAAGCGCCTCAGTTTAAGTACTCAACACAAGAAGAGATCGACAA
AATTCGTGCGAAAGTGCTTGAGATGAACGAGTTCATCAAGGCACAAGCGATGTACTACAAAGCGCAAGGTTACAA
CATCACGTTGTTTGATACTCACGCCTTGTTCGAGACGCTAACTTCTGCGCCAGAAGAGCACGGTTTCGTGAACGCG
AGCGATCCTTGTTTGGACATCAACCGCTCATCGTCTGTCGATTACATGTACACCCACGCATTGCGCTCTGAGTGTGC
AGCGTCTGGTGCTGAGAAGTTTGTGTTCTGGGATGTCACGCATCCAACAACAGCAACTCACCGCTATGTTGCAGAG
AAAATGCTAGAAAGTAGCAACAACTTAGCCGAGTACCGTTTCTAA

>S088
ATGATGAAAAAAACAATCACACTATTAACTGCATTACTCCCGCTTGCTTCTGCAGTTGCCGAAGAGCCAACCTTAT
CACCAGAAATGGTTTCAGCGTCTGAAGTGATCAGCACGCAAGAAAACCAAACCTATACCTATGTTCGCTGTTGGTA
TCGCACCAGCTACTCGAAAGATGATCCAGCGACCGATTGGGAATGGGCAAAAAACGAAGATGGTAGCTACTTCAC
CATTGACGGCTACTGGTGGAGCTCCGTTTCATTTAAAAACATGTTCTACACCAACACGTCGCAAAACGTTATCCGT
CAGCGTTGTGAAGCAACATTAGATTTGGCGAACGAGAACGCAGACATTACGTTCTTCGCCGCTGACAATCGCTTCT
CATACAACCACACGATCTGGAGCAACGACGCAGCAATGCAGCCAGATCAAATCAACAAAGTGGTTGCACTCGGTG
ACAGCTTGTCTGATACAGGCAACATCTTTAACGCATCACAATGGCGCTTCCCTAACCCGAACAGCTGGTTCTTAGG
TCACTTCTCCAACGGTTTTGTTTGGACAGAATACATTGCCAAAGCGAAGAACCTTCCGCTCTACAACTGGGCAGTT
GGCGGCGCGGCTGGTGAGAACCAATACATCGCGCTAACAGGGGTTGGTGAGCAAGTTTCTTCGTACTTAACCTACG
CAAAACTGGCGAAGAACTACAAACCAGCAAACACCTTGTTTACGCTTGAGTTTGGTTTGAATGACTTCATGAACTA
CAACCGTGGCGTTCCAGAAGTGAAAGCGGATTATGCAGAAGCACTGATTCGTTTGACGGACGCAGGTGCGAAGAA
CTTCATGTTGATGACACTGCCAGATGCGACGAAAGCGCCTCAGTTTAAGTACTCAACACAAGAAGAGATCGACAA
AATTCGTGCGAAAGTGCTTGAGATGAACGAGTTCATCAAGGCACAAGCGATGTACTACAAAGCGCAAGGTTACAA
CATCACGTTGTTTGATACTCACGCCTTGTTCGAGACGCTAACTTCTGCGCCAGAAGAGCACGGTTTCGTGAACGCG
AGCGATCCTTGTTTGGACATCAACCGCTCATCGTCTGTCGATTACATGTACACCCACGCATTGCGCTCTGAGTGTGC

AGCGTCTGGTGCTGAGAAGTTTGTGTTCTGGGATGTCACGCATCCAACAACAGCAACTCACCGCTATGTTGCAGAG
AAAATGCTAGAAAGTAGCAACAACTTAGCCGAGTACCGTTTCTAA

>S087
ATGATGAAAAAAACAATCACACTATTAACTGCATTACTCCCGCTTGCTTCTGCAGTTGCCGAAGAGCCAA
CCTTATCACCAGAAATGGTTTCAGCGTCTGAAGTGATCAGCACGCAAGAAAACCAAACCTATACCTATGT
TCGCTGTTGGTATCGCACCAGCTACTCGAAAGATGATCCAGCGACCGATTGGGAATGGGCAAAAAACGAA
GATGGTAGCTACTTCACCATTGACGGCTACTGGTGGAGCTCCGTTTCATTTAAAAACATGTTCTACACCA
ACACGTCGCAAAACGTTATCCGTCAGCGTTGTGAAGCAACATTAGATTTGGCGAACGAGAACGCAGACAT
TACGTTCTTCGCCGCTGACAATCGCTTCTCATACAACCACACGATCTGGAGCAACGACGCAGCAATGCAG
CCAGATCAAATCAACAAAGTGGTTGCACTCGGTGACAGCTTGTCTGATACAGGCAACATCTTTAACGCAT
CACAATGGCGCTTCCCTAACCCGAACAGCTGGTTCTTAGGTCACTTCTCCAACGGTTTTGTTTGGACAGA
ATACATTGCCAAAGCGAAGAACCTTCCGCTCTACAACTGGGCAGTTGGCGGCGCGGCTGGTGAGAACCAA
TACATCGCGCTAACAGGGGTTGGTGAGCAAGTTTCTTCGTACTTAACCTACGCAAAACTGGCGAAGAACT
ACAAACCAGCAAACACCTTGTTTACGCTTGAGTTTGGTTTGAATGACTTCATGAACTACAACCGTGGCGT
TCCAGAAGTGAAAGCGGATTATGCAGAAGCACTGATTCGTTTGACGGACGCAGGTGCGAAGAACTTCATG
TTGATGACACTGCCAGATGCGACGAAAGCGCCTCAGTTTAAGTACTCAACACAAGAAGAGATCGACAAAA
TTCGTGCGAAAGTGCTTGAGATGAACGAGTTCATCAAGGCACAAGCGATGTACTACAAAGCGCAAGGTTA
CAACATCACGTTGTTTGATACTCACGCCTTGTTCGAGACGCTAACTTCTGCGCCAGAAGAGCACGGTTTC
GTGAACGCGAGCGATCCTTGTTTGGACATCAACCGCTCATCGTCTGTCGATTACATGTACACCCACGCAT
TGCGCTCTGAGTGTGCAGCGTCTGGTGCTGAGAAGTTTGTGTTCTGGGATGTCACGCATCCAACAACAGC
AACTCACCGCTATGTTGCAGAGAAAATGCTAGAAAGTAGCAACAACTTAGCCGAGTACCGTTTCTAA

>S086
ATGATGAAAAAAACAATCACACTATTAACTGCATTACTCCCGCTTGCTTCTGCAGTTGCCGAAGAGCCAACCTTAT
CACCAGAAATGGTTTCAGCGTCTGAAGTGATCAGCACGCAAGAAAACCAAACCTATACCTATGTTCGCTGTTGGTA
TCGCACCAGCTACTCGAAAGATGATCCAGCGACCGATTGGGAATGGGCAAAAAACGAAGATGGTAGCTACTTCAC
CATTGACGGCTACTGGTGGAGCTCCGTTTCATTTAAAAACATGTTCTACACCAACACGTCGCAAAACGTTATCCGT
CAGCGTTGTGAAGCAACATTAGATTTGGCGAACGAGAACGCAGACATTACGTTCTTCGCCGCTGACAATCGCTTCT
CATACAACCACACGATCTGGAGCAACGACGCAGCAATGCAGCCAGATCAAATCAACAAAGTGGTTGCACTCGGTG
ACAGCTTGTCTGATACAGGCAACATCTTTAACGCATCACAATGGCGCTTCCCTAACCCGAACAGCTGGTTCTTAGG
TCACTTCTCCAACGGTTTTGTTTGGACAGAATACATTGCCAAAGCGAAGAACCTTCCGCTCTACAACTGGGCAGTT
GGCGGCGCGGCTGGTGAGAACCAATACATCGCGCTAACAGGGGTTGGTGAGCAAGTTTCTTCGTACTTAACCTACG
CAAAACTGGCGAAGAACTACAAACCAGCAAACACCTTGTTTACGCTTGAGTTTGGTTTGAATGACTTCATGAACTA
CAACCGTGGCGTTCCAGAAGTGAAAGCGGATTATGCAGAAGCACTGATTCGTTTGACGGACGCAGGTGCGAAGAA
CTTCATGTTGATGACACTGCCAGATGCGACGAAAGCGCCTCAGTTTAAGTACTCAACACAAGAAGAGATCGACAA
AATTCGTGCGAAAGTGCTTGAGATGAACGAGTTCATCAAGGCACAAGCGATGTACTACAAAGCGCAAGGTTACAA
CATCACGTTGTTTGATACTCACGCCTTGTTCGAGACGCTAACTTCTGCGCCAGAAGAGCACGGTTTCGTGAACGCG
AGCGATCCTTGTTTGGACATCAACCGCTCATCGTCTGTCGATTACATGTACACCCACGCATTGCGCTCTGAGTGTGC
AGCGTCTGGTGCTGAGAAGTTTGTGTTCTGGGATGTCACGCATCCAACAACAGCAACTCACCGCTATGTTGCAGAG
AAAATGCTAGAAAGTAGCAACAACTTAGCCGAGTACCGTTTCTAA

>S083
ATGATGAAAAAAACAATCACACTATTAACTGCATTACTCCCGCTTGCTTCTGCAGTTGCCGAAGAGCCAACCTTAT
CACCAGAAATGGTTTCAGCGTCTGAAGTGATCAGCACGCAAGAAAACCAAACCTATACCTATGTTCGCTGTTGGTA
TCGCACCAGCTACTCGAAAGATGATCCAGCGACCGATTGGGAATGGGCAAAAAACGAAGATGGTAGCTACTTCAC
CATTGACGGCTACTGGTGGAGCTCCGTTTCATTTAAAAACATGTTCTACACCAACACGTCGCAAAACGTTATCCGT
CAGCGTTGTGAAGCAACATTAGATTTGGCGAACGAGAACGCAGACATTACGTTCTTCGCCGCTGACAATCGCTTCT
CATACAACCACACGATCTGGAGCAACGACGCAGCAATGCAGCCAGATCAAATCAACAAAGTGGTTGCACTCGGTG
ACAGCTTGTCTGATACAGGCAACATCTTTAACGCATCACAATGGCGCTTCCCTAACCCGAACAGCTGGTTCTTAGG
TCACTTCTCCAACGGTTTTGTTTGGACAGAATACATTGCCAAAGCGAAGAACCTTCCGCTCTACAACTGGGCAGTT

GGCGGCGCGGCTGGTGAGAACCAATACATCGCGCTAACAGGGGTTGGTGAGCAAGTTTCTTCGTACTTAACCTACG
CAAAACTGGCGAAGAACTACAAACCAGCAAACACCTTGTTTACGCTTGAGTTTGGTTTGAATGACTTCATGAACTA
CAACCGTGGCGTTCCAGAAGTGAAAGCGGATTATGCAGAAGCACTGATTCGTTTGACGGACGCAGGTGCGAAGAA
CTTCATGTTGATGACACTGCCAGATGCGACGAAAGCGCCTCAGTTTAAGTACTCAACACAAGAAGAGATCGACAA
AATTCGTGCGAAAGTGCTTGAGATGAACGAGTTCATCAAGGCACAAGCGATGTACTACAAAGCGCAAGGTTACAA
CATCACGTTGTTTGATACTCACGCCTTGTTCGAGACGCTAACTTCTGCGCCAGAAGAGCACGGTTTCGTGAACGCG
AGCGATCCTTGTTTGGACATCAACCGCTCATCGTCTGTCGATTACATGTACACCCACGCATTGCGCTCTGAGTGTGC
AGCGTCTGGTGCTGAGAAGTTTGTGTTCTGGGATGTCACGCATCCAACAACAGCAACTCACCGCTATGTTGCAGAG
AAAATGCTAGAAAGTAGCAACAACTTAGCCGAGTACCGTTTCTAA

>S082
ATGATGAAAAAAACAATCACACTATTAACTGCATTACTCCCGCTTGCTTCTGCAGTTGCCGAAGAGCCAACCTTAT
CACCAGAAATGGTTTCAGCGTCTGAAGTGATCAGCACGCAAGAAAACCAAACCTATACCTATGTTCGCTGTTGGTA
TCGCACCAGCTACTCGAAAGATGATCCAGCGACCGATTGGGAATGGGCAAAAAACGAAGATGGTAGCTACTTCAC
CATTGACGGCTACTGGTGGAGCTCCGTTTCATTTAAAAACATGTTCTACACCAACACGTCGCAAAACGTTATCCGT
CAGCGTTGTGAAGCAACATTAGATTTGGCGAACGAGAACGCAGACATTACGTTCTTCGCCGCTGACAATCGCTTCT
CATACAACCACACGATCTGGAGCAACGACGCAGCAATGCAGCCAGATCAAATCAACAAAGTGGTTGCACTCGGTG
ACAGCTTGTCTGATACAGGCAACATCTTTAACGCATCACAATGGCGCTTCCCTAACCCGAACAGCTGGTTCTTAGG
TCACTTCTCCAACGGTTTTGTTTGGACAGAATACATTGCCAAAGCGAAGAACCTTCCGCTCTACAACTGGGCAGTT
GGCGGCGCGGCTGGTGAGAACCAATACATCGCGCTAACAGGGGTTGGTGAGCAAGTTTCTTCGTACTTAACCTACG
CAAAACTGGCGAAGAACTACAAACCAGCAAACACCTTGTTTACGCTTGAGTTTGGTTTGAATGACTTCATGAACTA
CAACCGTGGCGTTCCAGAAGTGAAAGCGGATTATGCAGAAGCACTGATTCGTTTGACGGACGCAGGTGCGAAGAA
CTTCATGTTGATGACACTGCCAGATGCGACGAAAGCGCCTCAGTTTAAGTACTCAACACAAGAAGAGATCGACAA
AATTCGTGCGAAAGTGCTTGAGATGAACGAGTTCATCAAGGCACAAGCGATGTACTACAAAGCGCAAGGTTACAA
CATCACGTTGTTTGATACTCACGCCTTGTTCGAGACGCTAACTTCTGCGCCAGAAGAGCACGGTTTCGTGAACGCG
AGCGATCCTTGTTTGGACATCAACCGCTCATCGTCTGTCGATTACATGTACACCCACGCATTGCGCTCTGAGTGTGC
AGCGTCTGGTGCTGAGAAGTTTGTGTTCTGGGATGTCACGCATCCAACAACAGCAACTCACCGCTATGTTGCAGAG
AAAATGCTAGAAAGTAGCAACAACTTAGCCGAGTACCGTTTCTAA

>S081
ATGATGAAAAAAACAATCACACTATTAACTGCATTACTCCCGCTTGCTTCTGCAGTTGCCGAAGAGCCAA
CCTTATCACCAGAAATGGTTTCAGCGTCTGAAGTGATCAGCACGCAAGAAAACCAAACCTATACCTATGT
TCGCTGTTGGTATCGCACCAGCTACTCGAAAGATGATCCAGCGACCGATTGGGAATGGGCAAAAAACGAA
GATGGTAGCTACTTCACCATTGACGGCTACTGGTGGAGCTCCGTTTCATTTAAAAACATGTTCTACACCA
ACACGTCGCAAAACGTTATCCGTCAGCGTTGTGAAGCAACATTAGATTTGGCGAACGAGAACGCAGACAT
TACGTTCTTCGCCGCTGACAATCGCTTCTCATACAACCACACGATCTGGAGCAACGACGCAGCAATGCAG
CCAGATCAAATCAACAAAGTGGTTGCACTCGGTGACAGCTTGTCTGATACAGGCAACATCTTTAACGCAT
CACAATGGCGCTTCCCTAACCCGAACAGCTGGTTCTTAGGTCACTTCTCCAACGGTTTTGTTTGGACAGA
ATACATTGCCAAAGCGAAGAACCTTCCGCTCTACAACTGGGCAGTTGGCGGCGCGGCTGGTGAGAACCAA
TACATCGCGCTAACAGGGGTTGGTGAGCAAGTTTCTTCGTACTTAACCTACGCAAAACTGGCGAAGAACT
ACAAACCAGCAAACACCTTGTTTACGCTTGAGTTTGGTTTGAATGACTTCATGAACTACAACCGTGGCGT
TCCAGAAGTGAAAGCGGATTATGCAGAAGCACTGATTCGTTTGACGGACGCAGGTGCGAAGAACTTCATG
TTGATGACACTGCCAGATGCGACGAAAGCGCCTCAGTTTAAGTACTCAACACAAGAAGAGATCGACAAAA
TTCGTGCGAAAGTGCTTGAGATGAACGAGTTCATCAAGGCACAAGCGATGTACTACAAAGCGCAAGGTTA
CAACATCACGTTGTTTGATACTCACGCCTTGTTCGAGACGCTAACTTCTGCGCCAGAAGAGCACGGTTTC
GTGAACGCGAGCGATCCTTGTTTGGACATCAACCGCTCATCGTCTGTCGATTACATGTACACCCACGCAT
TGCGCTCTGAGTGTGCAGCGTCTGGTGCTGAGAAGTTTGTGTTCTGGGATGTCACGCATCCAACAACAGC
AACTCACCGCTATGTTGCAGAGAAAATGCTAGAAAGTAGCAACAACTTAGCCGAGTACCGTTTCTAA

>S079
ATGATGAAAAAAACAATCACACTATTAACTGCATTACTCCCGCTTGCTTCTGCAGTTGCCGAAGAGCCAA

CCTTATCACCAGAAATGGTTTCAGCGTCTGAAGTGATCAGCACGCAAGAAAACCAAACCTATACCTATGT
TCGCTGTTGGTATCGCACCAGCTACTCGAAAGATGATCCAGCGACCGATTGGGAATGGGCAAAAAACGAA
GATGGTAGCTACTTCACCATTGACGGCTACTGGTGGAGCTCCGTTTCATTTAAAAACATGTTCTACACCA
ACACGTCGCAAAACGTTATCCGTCAGCGTTGTGAAGCAACATTAGATTTGGCGAACGAGAACGCAGACAT
TACGTTCTTCGCCGCTGACAATCGCTTCTCATACAACCACACGATCTGGAGCAACGACGCAGCAATGCAG
CCAGATCAAATCAACAAAGTGGTTGCACTCGGTGACAGCTTGTCTGATACAGGCAACATCTTTAACGCAT
CACAATGGCGCTTCCCTAACCCGAACAGCTGGTTCTTAGGTCACTTCTCCAACGGTTTTGTTTGGACAGA
ATACATTGCCAAAGCGAAGAACCTTCCGCTCTACAACTGGGCAGTTGGCGGCGCGGCTGGTGAGAACCAA
TACATCGCGCTAACAGGGGTTGGTGAGCAAGTTTCTTCGTACTTAACCTACGCAAAACTGGCGAAGAACT
ACAAACCAGCAAACACCTTGTTTACGCTTGAGTTTGGTTTGAATGACTTCATGAACTACAACCGTGGCGT
TCCAGAAGTGAAAGCGGATTATGCAGAAGCACTGATTCGTTTGACGGACGCAGGTGCGAAGAACTTCATG
TTGATGACACTGCCAGATGCGACGAAAGCGCCTCAGTTTAAGTACTCAACACAAGAAGAGATCGACAAAA
TTCGTGCGAAAGTGCTTGAGATGAACGAGTTCATCAAGGCACAAGCGATGTACTACAAAGCGCAAGGTTA
CAACATCACGTTGTTTGATACTCACGCCTTGTTCGAGACGCTAACTTCTGCGCCAGAAGAGCACGGTTTC
GTGAACGCGAGCGATCCTTGTTTGGACATCAACCGCTCATCGTCTGTCGATTACATGTACACCCACGCAT
TGCGCTCTGAGTGTGCAGCGTCTGGTGCTGAGAAGTTTGTGTTCTGGGATGTCACGCATCCAACAACAGC
AACTCACCGCTATGTTGCAGAGAAAATGCTAGAAAGTAGCAACAACTTAGCCGAGTACCGTTTCTAA

>S078
ATGATGAAAAAAACAATCACACTATTAACTGCATTACTCCCGCTTGCTTCTGCAGTTGCCGAAGAGCCAACCTTAT
CACCAGAAATGGTTTCAGCGTCTGAAGTGATCAGCACGCAAGAAAACCAAACCTATACCTATGTTCGCTGTTGGTA
TCGCACCAGCTACTCGAAAGATGATCCAGCGACCGATTGGGAATGGGCAAAAAACGAAGATGGTAGCTACTTCAC
CATTGACGGCTACTGGTGGAGCTCCGTTTCATTTAAAAACATGTTCTACACCAACACGTCGCAAAACGTTATCCGT
CAGCGTTGTGAAGCAACATTAGATTTGGCGAACGAGAACGCAGACATTACGTTCTTCGCCGCTGACAATCGCTTCT
CATACAACCACACGATCTGGAGCAACGACGCAGCAATGCAGCCAGATCAAATCAACAAAGTGGTTGCACTCGGTG
ACAGCTTGTCTGATACAGGCAACATCTTTAACGCATCACAATGGCGCTTCCCTAACCCGAACAGCTGGTTCTTAGG
TCACTTCTCCAACGGTTTTGTTTGGACAGAATACATTGCCAAAGCGAAGAACCTTCCGCTCTACAACTGGGCAGTT
GGCGGCGCGGCTGGTGAGAACCAATACATCGCGCTAACAGGGGTTGGTGAGCAAGTTTCTTCGTACTTAACCTACG
CAAAACTGGCGAAGAACTACAAACCAGCAAACACCTTGTTTACGCTTGAGTTTGGTTTGAATGACTTCATGAACTA
CAACCGTGGCGTTCCAGAAGTGAAAGCGGATTATGCAGAAGCACTGATTCGTTTGACGGACGCAGGTGCGAAGAA
CTTCATGTTGATGACACTGCCAGATGCGACGAAAGCGCCTCAGTTTAAGTACTCAACACAAGAAGAGATCGACAA
AATTCGTGCGAAAGTGCTTGAGATGAACGAGTTCATCAAGGCACAAGCGATGTACTACAAAGCGCAAGGTTACAA
CATCACGTTGTTTGATACTCACGCCTTGTTCGAGACGCTAACTTCTGCGCCAGAAGAGCACGGTTTCGTGAACGCG
AGCGATCCTTGTTTGGACATCAACCGCTCATCGTCTGTCGATTACATGTACACCCACGCATTGCGCTCTGAGTGTGC
AGCGTCTGGTGCTGAGAAGTTTGTGTTCTGGGATGTCACGCATCCAACAACAGCAACTCACCGCTATGTTGCAGAG
AAAATGCTAGAAAGTAGCAACAACTTAGCCGAGTACCGTTTCTAA

>S077
ATGATGAAAAAAACAATCACACTATTAACTGCATTACTCCCGCTTGCTTCTGCAGTTGCCGAAGAGCCAA
CCTTATCACCAGAAATGGTTTCAGCGTCTGAAGTGATCAGCACGCAAGAAAACCAAACCTATACCTATGT
TCGCTGTTGGTATCGCACCAGCTACTCGAAAGATGATCCAGCGACCGATTGGGAATGGGCAAAAAACGAA
GATGGTAGCTACTTCACCATTGACGGCTACTGGTGGAGCTCCGTTTCATTTAAAAACATGTTCTACACCA
ACACGTCGCAAAACGTTATCCGTCAGCGTTGTGAAGCAACATTAGATTTGGCGAACGAGAACGCAGACAT
TACGTTCTTCGCCGCTGACAATCGCTTCTCATACAACCACACGATCTGGAGCAACGACGCAGCAATGCAG
CCAGATCAAATCAACAAAGTGGTTGCACTCGGTGACAGCTTGTCTGATACAGGCAACATCTTTAACGCAT
CACAATGGCGCTTCCCTAACCCGAACAGCTGGTTCTTAGGTCACTTCTCCAACGGTTTTGTTTGGACAGA
ATACATTGCCAAAGCGAAGAACCTTCCGCTCTACAACTGGGCAGTTGGCGGCGCGGCTGGTGAGAACCAA
TACATCGCGCTAACAGGGGTTGGTGAGCAAGTTTCTTCGTACTTAACCTACGCAAAACTGGCGAAGAACT
ACAAACCAGCAAACACCTTGTTTACGCTTGAGTTTGGTTTGAATGACTTCATGAACTACAACCGTGGCGT
TCCAGAAGTGAAAGCGGATTATGCAGAAGCACTGATTCGTTTGACGGACGCAGGTGCGAAGAACTTCATG
TTGATGACACTGCCAGATGCGACGAAAGCGCCTCAGTTTAAGTACTCAACACAAGAAGAGATCGACAAAA

TTCGTGCGAAAGTGCTTGAGATGAACGAGTTCATCAAGGCACAAGCGATGTACTACAAAGCGCAAGGTTA
CAACATCACGTTGTTTGATACTCACGCCTTGTTCGAGACGCTAACTTCTGCGCCAGAAGAGCACGGTTTC
GTGAACGCGAGCGATCCTTGTTTGGACATCAACCGCTCATCGTCTGTCGATTACATGTACACCCACGCAT
TGCGCTCTGAGTGTGCAGCGTCTGGTGCTGAGAAGTTTGTGTTCTGGGATGTCACGCATCCAACAACAGC
AACTCACCGCTATGTTGCAGAGAAAATGCTAGAAAGTAGCAACAACTTAGCCGAGTACCGTTTCTAA

>S076
ATGATGAAAAAAACAATCACACTATTAACTGCATTACTCCCGCTTGCTTCTGCAGTTGCCGAAGAGCCAACCTTAT
CACCAGAAATGGTTTCAGCGTCTGAAGTGATCAGCACGCAAGAAAACCAAACCTATACCTATGTTCGCTGTTGGTA
TCGCACCAGCTACTCGAAAGATGATCCAGCGACCGATTGGGAATGGGCAAAAAACGAAGATGGTAGCTACTTCAC
CATTGACGGCTACTGGTGGAGCTCCGTTTCATTTAAAAACATGTTCTACACCAACACGTCGCAAAACGTTATCCGT
CAGCGTTGTGAAGCAACATTAGATTTGGCGAACGAGAACGCAGACATTACGTTCTTCGCCGCTGACAATCGCTTCT
CATACAACCACACGATCTGGAGCAACGACGCAGCAATGCAGCCAGATCAAATCAACAAAGTGGTTGCACTCGGTG
ACAGCTTGTCTGATACAGGCAACATCTTTAACGCATCACAATGGCGCTTCCCTAACCCGAACAGCTGGTTCTTAGG
TCACTTCTCCAACGGTTTTGTTTGGACAGAATACATTGCCAAAGCGAAGAACCTTCCGCTCTACAACTGGGCAGTT
GGCGGCGCGGCTGGTGAGAACCAATACATCGCGCTAACAGGGGTTGGTGAGCAAGTTTCTTCGTACTTAACCTACG
CAAAACTGGCGAAGAACTACAAACCAGCAAACACCTTGTTTACGCTTGAGTTTGGTTTGAATGACTTCATGAACTA
CAACCGTGGCGTTCCAGAAGTGAAAGCGGATTATGCAGAAGCACTGATTCGTTTGACGGACGCAGGTGCGAAGAA
CTTCATGTTGATGACACTGCCAGATGCGACGAAAGCGCCTCAGTTTAAGTACTCAACACAAGAAGAGATCGACAA
AATTCGTGCGAAAGTGCTTGAGATGAACGAGTTCATCAAGGCACAAGCGATGTACTACAAAGCGCAAGGTTACAA
CATCACGTTGTTTGATACTCACGCCTTGTTCGAGACGCTAACTTCTGCGCCAGAAGAGCACGGTTTCGTGAACGCG
AGCGATCCTTGTTTGGACATCAACCGCTCATCGTCTGTCGATTACATGTACACCCACGCATTGCGCTCTGAGTGTGC
AGCGTCTGGTGCTGAGAAGTTTGTGTTCTGGGATGTCACGCATCCAACAACAGCAACTCACCGCTATGTTGCAGAG
AAAATGCTAGAAAGTAGCAACAACTTAGCCGAGTACCGTTTCTAA

>S075
ATGATGAAAAAAACAATCACACTATTAACTGCATTACTCCCGCTTGCTTCTGCAGTTGCCGAAGAGCCAACCTTAT
CACCAGAAATGGTTTCAGCGTCTGAAGTGATCAGCACGCAAGAAAACCAAACCTATACCTATGTTCGCTGTTGGTA
TCGCACCAGCTACTCGAAAGATGATCCAGCGACCGATTGGGAATGGGCAAAAAACGAAGATGGTAGCTACTTCAC
CATTGACGGCTACTGGTGGAGCTCCGTTTCATTTAAAAACATGTTCTACACCAACACGTCGCAAAACGTTATCCGT
CAGCGTTGTGAAGCAACATTAGATTTGGCGAACGAGAACGCAGACATTACGTTCTTCGCCGCTGACAATCGCTTCT
CATACAACCACACGATCTGGAGCAACGACGCAGCAATGCAGCCAGATCAAATCAACAAAGTGGTTGCACTCGGTG
ACAGCTTGTCTGATACAGGCAACATCTTTAACGCATCACAATGGCGCTTCCCTAACCCGAACAGCTGGTTCTTAGG
TCACTTCTCCAACGGTTTTGTTTGGACAGAATACATTGCCAAAGCGAAGAACCTTCCGCTCTACAACTGGGCAGTT
GGCGGCGCGGCTGGTGAGAACCAATACATCGCGCTAACAGGGGTTGGTGAGCAAGTTTCTTCGTACTTAACCTACG
CAAAACTGGCGAAGAACTACAAACCAGCAAACACCTTGTTTACGCTTGAGTTTGGTTTGAATGACTTCATGAACTA
CAACCGTGGCGTTCCAGAAGTGAAAGCGGATTATGCAGAAGCACTGATTCGTTTGACGGACGCAGGTGCGAAGAA
CTTCATGTTGATGACACTGCCAGATGCGACGAAAGCGCCTCAGTTTAAGTACTCAACACAAGAAGAGATCGACAA
AATTCGTGCGAAAGTGCTTGAGATGAACGAGTTCATCAAGGCACAAGCGATGTACTACAAAGCGCAAGGTTACAA
CATCACGTTGTTTGATACTCACGCCTTGTTCGAGACGCTAACTTCTGCGCCAGAAGAGCACGGTTTCGTGAACGCG
AGCGATCCTTGTTTGGACATCAACCGCTCATCGTCTGTCGATTACATGTACACCCACGCATTGCGCTCTGAGTGTGC
AGCGTCTGGTGCTGAGAAGTTTGTGTTCTGGGATGTCACGCATCCAACAACAGCAACTCACCGCTATGTTGCAGAG
AAAATGCTAGAAAGTAGCAACAACTTAGCCGAGTACCGTTTCTAA

>S074
ATGATGAAAAAAACAATCACACTATTAACTGCATTACTCCCGCTTGCTTCTGCAGTTGCCGAAGAGCCAACCTTAT
CACCAGAAATGGTTTCAGCGTCTGAAGTGATCAGCACGCAAGAAAACCAAACCTATACCTATGTTCGCTGTTGGTA
TCGCACCAGCTACTCGAAAGATGATCCAGCGACCGATTGGGAATGGGCAAAAAACGAAGATGGTAGCTACTTCAC
CATTGACGGCTACTGGTGGAGCTCCGTTTCATTTAAAAACATGTTCTACACCAACACGTCGCAAAACGTTATCCGT
CAGCGTTGTGAAGCAACATTAGATTTGGCGAACGAGAACGCAGACATTACGTTCTTCGCCGCTGACAATCGCTTCT
CATACAACCACACGATCTGGAGCAACGACGCAGCAATGCAGCCAGATCAAATCAACAAAGTGGTTGCACTCGGTG

ACAGCTTGTCTGATACAGGCAACATCTTTAACGCATCACAATGGCGCTTCCCTAACCCGAACAGCTGGTTCTTAGG
TCACTTCTCCAACGGTTTTGTTTGGACAGAATACATTGCCAAAGCGAAGAACCTTCCGCTCTACAACTGGGCAGTT
GGCGGCGCGGCTGGTGAGAACCAATACATCGCGCTAACAGGGGTTGGTGAGCAAGTTTCTTCGTACTTAACCTACG
CAAAACTGGCGAAGAACTACAAACCAGCAAACACCTTGTTTACGCTTGAGTTTGGTTTGAATGACTTCATGAACTA
CAACCGTGGCGTTCCAGAAGTGAAAGCGGATTATGCAGAAGCACTGATTCGTTTGACGGACGCAGGTGCGAAGAA
CTTCATGTTGATGACACTGCCAGATGCGACGAAAGCGCCTCAGTTTAAGTACTCAACACAAGAAGAGATCGACAA
AATTCGTGCGAAAGTGCTTGAGATGAACGAGTTCATCAAGGCACAAGCGATGTACTACAAAGCGCAAGGTTACAA
CATCACGTTGTTTGATACTCACGCCTTGTTCGAGACGCTAACTTCTGCGCCAGAAGAGCACGGTTTCGTGAACGCG
AGCGATCCTTGTTTGGACATCAACCGCTCATCGTCTGTCGATTACATGTACACCCACGCATTGCGCTCTGAGTGTGC
AGCGTCTGGTGCTGAGAAGTTTGTGTTCTGGGATGTCACGCATCCAACAACAGCAACTCACCGCTATGTTGCAGAG
AAAATGCTAGAAAGTAGCAACAACTTAGCCGAGTACCGTTTCTAA

>S073
ATGATGAAAAAAACAATCACACTATTAACTGCATTACTCCCGCTTGCTTCTGCAGTTGCCGAAGAGCCAA
CCTTATCACCAGAAATGGTTTCAGCGTCTGAAGTGATCAGCACGCAAGAAAACCAAACCTATACCTATGT
TCGCTGTTGGTATCGCACCAGCTACTCGAAAGATGATCCGGCGACCGATTGGGAATGGGCAAAAAACGAA
GATGGTAGCTACTTCACCATTGACGGCTACTGGTGGAGCTCCGTTTCATTTAAAAACATGTTCTACACCA
ACACGTCGCAAAACGTTATCCGTCAGCGTTGTGAAGCCACATTAGATTTGGCGAACGAGAACGCAGACAT
TACGTTCTTCGCCGCTGACAATCGCTTCTCATACAACCACACGATCTGGAGCAACGACGCAGCAATGCAG
CCAGATCAAATCAACAAAGTGGTTGCACTCGGTGACAGCTTGTCTGATACAGGCAACATCTTTAACGCAT
CACAATGGCGCTTCCCTAACCCGAACAGCTGGTTCTTAGGTCACTTCTCCAACGGTTTTGTGTGGACAGA
ATACATTGCCAAAGCGAAGAACCTTCCGCTCTACAACTGGGCAGTTGGCGGCGCGGCTGGTGAGAACCAA
TACATCGCGCTAACAGGGGTTGGTGATCAAGTTTCTTCGTACTTAACCTACGCAAAACTGGCGAAGAACT
ACAAACCAGCAAACACCTTGTTTACGCTTGAGTTTGGTTTGAATGACTTCATGAACTACAACCGTGGCGT
TCCAGAAGTGAAAGCAGATTATGCAGAAGCACTGATTCGTTTGACGGACGCAGGTGCAAAGAACTTCATG
TTGATGACACTGCCAGACGCGACGAAAGCGCCTCAGTTTAAGTACTCAACACAAGAAGAGATCGACAAAA
TTCGTGCGAAAGTGCTTGAGATGAACGAGTTCATCAAGGCACAAGCGATGTACTACAAAGCGCAAGGTTA
CAACATCACGTTGTTTGATACTCACGCCTTGTTCGAGACGCTAACTTCTGCGCCAGAAGAGCACGGTTTC
GTGAACGCGAGCGATCCTTGTTTGGACATCAACCGTTCATCGTCTGTCGATTACATGTACACCCACGCAT
TGCGCTCTGAGTGTGCGGCGTCTGGTGCTGAGAAGTTTGTGTTCTGGGATGTCACGCATCCAACAACAGC
AACTCACCGCTATGTTGCAGAGAAAATGCTAGAAAGTAGCAACAACTTAGCCGAGTACCGTTTCTAA

>S072
ATGATGAAAAAAACAATCACACTATTAACTGCATTACTCCCGCTTGCTTCTGCAGTTGCCGAAGAGCCAA
CCTTATCACCAGAAATGGTTTCAGCGTCTGAAGTGATCAGCACGCAAGAAAACCAAACCTATACCTATGT
TCGCTGTTGGTATCGCACCAGCTACTCGAAAGATGATCCAGCGACCGATTGGGAATGGGCAAAAAACGAA
GATGGTAGCTACTTCACCATTGACGGCTACTGGTGGAGCTCCGTTTCATTTAAAAACATGTTCTACACCA
ACACGTCGCAAAACGTTATCCGTCAGCGTTGTGAAGCAACATTAGATTTGGCGAACGAGAACGCAGACAT
TACGTTCTTCGCCGCTGACAATCGCTTCTCATACAACCACACGATCTGGAGCAACGACGCAGCAATGCAG
CCAGATCAAATCAACAAAGTGGTTGCACTCGGTGACAGCTTGTCTGATACAGGCAACATCTTTAACGCAT
CACAATGGCGCTTCCCTAACCCGAACAGCTGGTTCTTAGGTCACTTCTCCAACGGTTTTGTTTGGACAGA
ATACATTGCCAAAGCGAAGAACCTTCCGCTCTACAACTGGGCAGTTGGCGGCGCGGCTGGTGAGAACCAA
TACATCGCGCTAACAGGGGTTGGTGAGCAAGTTTCTTCGTACTTAACCTACGCAAAACTGGCGAAGAACT
ACAAACCAGCAAACACCTTGTTTACGCTTGAGTTTGGTTTGAATGACTTCATGAACTACAACCGTGGCGT
TCCAGAAGTGAAAGCGGATTATGCAGAAGCACTGATTCGTTTGACGGACGCAGGTGCGAAGAACTTCATG
TTGATGACACTGCCAGATGCGACGAAAGCGCCTCAGTTTAAGTACTCAACACAAGAAGAGATCGACAAAA
TTCGTGCGAAAGTGCTTGAGATGAACGAGTTCATCAAGGCACAAGCGATGTACTACAAAGCGCAAGGTTA
CAACATCACGTTGTTTGATACTCACGCCTTGTTCGAGACGCTAACTTCTGCGCCAGAAGAGCACGGTTTC
GTGAACGCGAGCGATCCTTGTTTGGACATCAACCGCTCATCGTCTGTCGATTACATGTACACCCACGCAT
TGCGCTCTGAGTGTGCAGCGTCTGGTGCTGAGAAGTTTGTGTTCTGGGATGTCACGCATCCAACAACAGC
AACTCACCGCTATGTTGCAGAGAAAATGCTAGAAAGTAGCAACAACTTAGCCGAGTACCGTTTCTAA

>S071
ATGATGAAAAAAACAATCACACTATTAACTGCATTACTCCCGCTTGCTTCTGCAGTTGCCGAAGAGCCAA
CCTTATCACCAGAAATGGTTTCAGCGTCTGAAGTGATCAGCACGCAAGAAAACCAAACCTATACCTATGT
TCGCTGTTGGTATCGCACCAGCTACTCGAAAGATGATCCAGCGACCGATTGGGAATGGGCAAAAAACGAA
GATGGTAGCTACTTCACCATTGACGGCTACTGGTGGAGCTCCGTTTCATTTAAAAACATGTTCTACACCA
ACACGTCGCAAAACGTTATCCGTCAGCGTTGTGAAGCAACATTAGATTTGGCGAACGAGAACGCAGACAT
TACGTTCTTCGCCGCTGACAATCGCTTCTCATACAACCACACGATCTGGAGCAACGACGCAGCAATGCAG
CCAGATCAAATCAACAAAGTGGTTGCACTCGGTGACAGCTTGTCTGATACAGGCAACATCTTTAACGCAT
CACAATGGCGCTTCCCTAACCCGAACAGCTGGTTCTTAGGTCACTTCTCCAACGGTTTTGTTTGGACAGA
ATACATTGCCAAAGCGAAGAACCTTCCGCTCTACAACTGGGCAGTTGGCGGCGCGGCTGGTGAGAACCAA
TACATCGCGCTAACAGGGGTTGGTGAGCAAGTTTCTTCGTACTTAACCTACGCAAAACTGGCGAAGAACT
ACAAACCAGCAAACACCTTGTTTACGCTTGAGTTTGGTTTGAATGACTTCATGAACTACAACCGTGGCGT
TCCAGAAGTGAAAGCGGATTATGCAGAAGCACTGATTCGTTTGACGGACGCAGGTGCGAAGAACTTCATG
TTGATGACACTGCCAGATGCGACGAAAGCGCCTCAGTTTAAGTACTCAACACAAGAAGAGATCGACAAAA
TTCGTGCGAAAGTGCTTGAGATGAACGAGTTCATCAAGGCACAAGCGATGTACTACAAAGCGCAAGGTTA
CAACATCACGTTGTTTGATACTCACGCCTTGTTCGAGACGCTAACTTCTGCGCCAGAAGAGCACGGTTTC
GTGAACGCGAGCGATCCTTGTTTGGACATCAACCGCTCATCGTCTGTCGATTACATGTACACCCACGCAT
TGCGCTCTGAGTGTGCAGCGTCTGGTGCTGAGAAGTTTGTGTTCTGGGATGTCACGCATCCAACAACAGC
AACTCACCGCTATGTTGCAGAGAAAATGCTAGAAAGTAGCAACAACTTAGCCGAGTACCGTTTCTAA

>S070
ATGATGAAAAAAACAATCACACTATTAACTGCATTACTCCCGCTTGCTTCTGCAGTTGCCGAAGAGCCAACCTTAT
CACCAGAAATGGTTTCAGCGTCTGAAGTGATCAGCACGCAAGAAAACCAAACCTATACCTATGTTCGCTGTTGGTA
TCGCACCAGCTACTCGAAAGATGATCCAGCGACCGATTGGGAATGGGCAAAAAACGAAGATGGTAGCTACTTCAC
CATTGACGGCTACTGGTGGAGCTCCGTTTCATTTAAAAACATGTTCTACACCAACACGTCGCAAAACGTTATCCGT
CAGCGTTGTGAAGCAACATTAGATTTGGCGAACGAGAACGCAGACATTACGTTCTTCGCCGCTGACAATCGCTTCT
CATACAACCACACGATCTGGAGCAACGACGCAGCAATGCAGCCAGATCAAATCAACAAAGTGGTTGCACTCGGTG
ACAGCTTGTCTGATACAGGCAACATCTTTAACGCATCACAATGGCGCTTCCCTAACCCGAACAGCTGGTTCTTAGG
TCACTTCTCCAACGGTTTTGTTTGGACAGAATACATTGCCAAAGCGAAGAACCTTCCGCTCTACAACTGGGCAGTT
GGCGGCGCGGCTGGTGAGAACCAATACATCGCGCTAACAGGGGTTGGTGAGCAAGTTTCTTCGTACTTAACCTACG
CAAAACTGGCGAAGAACTACAAACCAGCAAACACCTTGTTTACGCTTGAGTTTGGTTTGAATGACTTCATGAACTA
CAACCGTGGCGTTCCAGAAGTGAAAGCGGATTATGCAGAAGCACTGATTCGTTTGACGGACGCAGGTGCGAAGAA
CTTCATGTTGATGACACTGCCAGATGCGACGAAAGCGCCTCAGTTTAAGTACTCAACACAAGAAGAGATCGACAA
AATTCGTGCGAAAGTGCTTGAGATGAACGAGTTCATCAAGGCACAAGCGATGTACTACAAAGCGCAAGGTTACAA
CATCACGTTGTTTGATACTCACGCCTTGTTCGAGACGCTAACTTCTGCGCCAGAAGAGCACGGTTTCGTGAACGCG
AGCGATCCTTGTTTGGACATCAACCGCTCATCGTCTGTCGATTACATGTACACCCACGCATTGCGCTCTGAGTGTGC
AGCGTCTGGTGCTGAGAAGTTTGTGTTCTGGGATGTCACGCATCCAACAACAGCAACTCACCGCTATGTTGCAGAG
AAAATGCTAGAAAGTAGCAACAACTTAGCCGAGTACCGTTTCTAA

>S069
ATGATGAAAAAAACAATCACACTATTAACTGCATTACTCCCGCTTGCTTCTGCAGTTGCCGAAGAGCCAA
CCTTATCACCAGAAATGGTTTCAGCGTCTGAAGTGATCAGCACGCAAGAAAACCAAACCTATACCTATGT
TCGCTGTTGGTATCGCACCAGCTACTCGAAAGATGATCCAGCGACCGATTGGGAATGGGCAAAAAACGAA
GATGGTAGCTACTTCACCATTGACGGCTACTGGTGGAGCTCCGTTTCATTTAAAAACATGTTCTACACCA
ACACGTCGCAAAACGTTATCCGTCAGCGTTGTGAAGCAACATTAGATTTGGCGAACGAGAACGCAGACAT
TACGTTCTTCGCCGCTGACAATCGCTTCTCATACAACCACACGATCTGGAGCAACGACGCAGCAATGCAG
CCAGATCAAATCAACAAAGTGGTTGCACTCGGTGACAGCTTGTCTGATACAGGCAACATCTTTAACGCAT
CACAATGGCGCTTCCCTAACCCGAACAGCTGGTTCTTAGGTCACTTCTCCAACGGTTTTGTTTGGACAGA
ATACATTGCCAAAGCGAAGAACCTTCCGCTCTACAACTGGGCAGTTGGCGGCGCGGCTGGTGAGAACCAA
TACATCGCGCTAACAGGGGTTGGTGAGCAAGTTTCTTCGTACTTAACCTACGCAAAACTGGCGAAGAACT

ACAAACCAGCAAACACCTTGTTTACGCTTGAGTTTGGTTTGAATGACTTCATGAACTACAACCGTGGCGT
TCCAGAAGTGAAAGCGGATTATGCAGAAGCACTGATTCGTTTGACGGACGCAGGTGCGAAGAACTTCATG
TTGATGACACTGCCAGATGCGACGAAAGCGCCTCAGTTTAAGTACTCAACACAAGAAGAGATCGACAAAA
TTCGTGCGAAAGTGCTTGAGATGAACGAGTTCATCAAGGCACAAGCGATGTACTACAAAGCGCAAGGTTA
CAACATCACGTTGTTTGATACTCACGCCTTGTTCGAGACGCTAACTTCTGCGCCAGAAGAGCACGGTTTC
GTGAACGCGAGCGATCCTTGTTTGGACATCAACCGCTCATCGTCTGTCGATTACATGTACACCCACGCAT
TGCGCTCTGAGTGTGCAGCGTCTGGTGCTGAGAAGTTTGTGTTCTGGGATGTCACGCATCCAACAACAGC
AACTCACCGCTATGTTGCAGAGAAAATGCTAGAAAGTAGCAACAACTTAGCCGAGTACCGTTTCTAA

>S068
ATGATGAAAAAAACAATCACACTATTAACTGCATTACTCCCGCTTGCTTCTGCAGTTGCCGAAGAGCCAA
CCTTATCACCAGAAATGGTTTCAGCGTCTGAAGTGATCAGCACGCAAGAAAACCAAACCTATACCTATGT
TCGCTGTTGGTATCGCACCAGCTACTCGAAAGATGATCCAGCGACCGATTGGGAATGGGCAAAAAACGAA
GATGGTAGCTACTTCACCATTGACGGCTACTGGTGGAGCTCCGTTTCATTTAAAAACATGTTCTACACCA
ACACGTCGCAAAACGTTATCCGTCAGCGTTGTGAAGCAACATTAGATTTGGCGAACGAGAACGCAGACAT
TACGTTCTTCGCCGCTGACAATCGCTTCTCATACAACCACACGATCTGGAGCAACGACGCAGCAATGCAG
CCAGATCAAATCAACAAAGTGGTTGCACTCGGTGACAGCTTGTCTGATACAGGCAACATCTTTAACGCAT
CACAATGGCGCTTCCCTAACCCGAACAGCTGGTTCTTAGGTCACTTCTCCAACGGTTTTGTTTGGACAGA
ATACATTGCCAAAGCGAAGAACCTTCCGCTCTACAACTGGGCAGTTGGCGGCGCGGCTGGTGAGAACCAA
TACATCGCGCTAACAGGGGTTGGTGAGCAAGTTTCTTCGTACTTAACCTACGCAAAACTGGCGAAGAACT
ACAAACCAGCAAACACCTTGTTTACGCTTGAGTTTGGTTTGAATGACTTCATGAACTACAACCGTGGCGT
TCCAGAAGTGAAAGCGGATTATGCAGAAGCACTGATTCGTTTGACGGACGCAGGTGCGAAGAACTTCATG
TTGATGACACTGCCAGATGCGACGAAAGCGCCTCAGTTTAAGTACTCAACACAAGAAGAGATCGACAAAA
TTCGTGCGAAAGTGCTTGAGATGAACGAGTTCATCAAGGCACAAGCGATGTACTACAAAGCGCAAGGTTA
CAACATCACGTTGTTTGATACTCACGCCTTGTTCGAGACGCTAACTTCTGCGCCAGAAGAGCACGGTTTC
GTGAACGCGAGCGATCCTTGTTTGGACATCAACCGCTCATCGTCTGTCGATTACATGTACACCCACGCAT
TGCGCTCTGAGTGTGCAGCGTCTGGTGCTGAGAAGTTTGTGTTCTGGGATGTCACGCATCCAACAACAGC
AACTCACCGCTATGTTGCAGAGAAAATGCTAGAAAGTAGCAACAACTTAGCCGAGTACCGTTTCTAA

>S067
ATGATGAAAAAAACAATCACACTATTAACTGCATTACTCCCGCTTGCTTCTGCAGTTGCCGAAGAGCCAACCTTAT
CACCAGAAATGGTTTCAGCGTCTGAAGTGATCAGCACGCAAGAAAACCAAACCTATACCTATGTTCGCTGTTGGTA
TCGCACCAGCTACTCGAAAGATGATCCAGCGACCGATTGGGAATGGGCAAAAAACGAAGATGGTAGCTACTTCAC
CATTGACGGCTACTGGTGGAGCTCCGTTTCATTTAAAAACATGTTCTACACCAACACGTCGCAAAACGTTATCCGT
CAGCGTTGTGAAGCAACATTAGATTTGGCGAACGAGAACGCAGACATTACGTTCTTCGCCGCTGACAATCGCTTCT
CATACAACCACACGATCTGGAGCAACGACGCAGCAATGCAGCCAGATCAAATCAACAAAGTGGTTGCACTCGGTG
ACAGCTTGTCTGATACAGGCAACATCTTTAACGCATCACAATGGCGCTTCCCTAACCCGAACAGCTGGTTCTTAGG
TCACTTCTCCAACGGTTTTGTTTGGACAGAATACATTGCCAAAGCGAAGAACCTTCCGCTCTACAACTGGGCAGTT
GGCGGCGCGGCTGGTGAGAACCAATACATCGCGCTAACAGGGGTTGGTGAGCAAGTTTCTTCGTACTTAACCTACG
CAAAACTGGCGAAGAACTACAAACCAGCAAACACCTTGTTTACGCTTGAGTTTGGTTTGAATGACTTCATGAACTA
CAACCGTGGCGTTCCAGAAGTGAAAGCGGATTATGCAGAAGCACTGATTCGTTTGACGGACGCAGGTGCGAAGAA
CTTCATGTTGATGACACTGCCAGATGCGACGAAAGCGCCTCAGTTTAAGTACTCAACACAAGAAGAGATCGACAA
AATTCGTGCGAAAGTGCTTGAGATGAACGAGTTCATCAAGGCACAAGCGATGTACTACAAAGCGCAAGGTTACAA
CATCACGTTGTTTGATACTCACGCCTTGTTCGAGACGCTAACTTCTGCGCCAGAAGAGCACGGTTTCGTGAACGCG
AGCGATCCTTGTTTGGACATCAACCGCTCATCGTCTGTCGATTACATGTACACCCACGCATTGCGCTCTGAGTGTGC
AGCGTCTGGTGCTGAGAAGTTTGTGTTCTGGGATGTCACGCATCCAACAACAGCAACTCACCGCTATGTTGCAGAG
AAAATGCTAGAAAGTAGCAACAACTTAGCCGAGTACCGTTTCTAA

>S066
ATGATGAAAAAAACAATCACACTATTAACTGCATTACTCCCGCTTGCTTCTGCAGTTGCCGAAGAGCCAACCTTAT
CACCAGAAATGGTTTCAGCGTCTGAAGTGATCAGCACGCAAGAAAACCAAACCTATACCTATGTTCGCTGTTGGTA

TCGCACCAGCTACTCGAAAGATGATCCAGCGACCGATTGGGAATGGGCAAAAAACGAAGATGGTAGCTACTTCAC
CATTGACGGCTACTGGTGGAGCTCCGTTTCATTTAAAAACATGTTCTACACCAACACGTCGCAAAACGTTATCCGT
CAGCGTTGTGAAGCAACATTAGATTTGGCGAACGAGAACGCAGACATTACGTTCTTCGCCGCTGACAATCGCTTCT
CATACAACCACACGATCTGGAGCAACGACGCAGCAATGCAGCCAGATCAAATCAACAAAGTGGTTGCACTCGGTG
ACAGCTTGTCTGATACAGGCAACATCTTTAACGCATCACAATGGCGCTTCCCTAACCCGAACAGCTGGTTCTTAGG
TCACTTCTCCAACGGTTTTGTTTGGACAGAATACATTGCCAAAGCGAAGAACCTTCCGCTCTACAACTGGGCAGTT
GGCGGCGCGGCTGGTGAGAACCAATACATCGCGCTAACAGGGGTTGGTGAGCAAGTTTCTTCGTACTTAACCTACG
CAAAACTGGCGAAGAACTACAAACCAGCAAACACCTTGTTTACGCTTGAGTTTGGTTTGAATGACTTCATGAACTA
CAACCGTGGCGTTCCAGAAGTGAAAGCGGATTATGCAGAAGCACTGATTCGTTTGACGGACGCAGGTGCGAAGAA
CTTCATGTTGATGACACTGCCAGATGCGACGAAAGCGCCTCAGTTTAAGTACTCAACACAAGAAGAGATCGACAA
AATTCGTGCGAAAGTGCTTGAGATGAACGAGTTCATCAAGGCACAAGCGATGTACTACAAAGCGCAAGGTTACAA
CATCACGTTGTTTGATACTCACGCCTTGTTCGAGACGCTAACTTCTGCGCCAGAAGAGCACGGTTTCGTGAACGCG
AGCGATCCTTGTTTGGACATCAACCGCTCATCGTCTGTCGATTACATGTACACCCACGCATTGCGCTCTGAGTGTGC
AGCGTCTGGTGCTGAGAAGTTTGTGTTCTGGGATGTCACGCATCCAACAACAGCAACTCACCGCTATGTTGCAGAG
AAAATGCTAGAAAGTAGCAACAACTTAGCCGAGTACCGTTTCTAA

>S065
ATGATGAAAAAAACAATCACACTATTAACTGCATTACTCCCGCTTGCTTCTGCAGTTGCCGAAGAGCCAA
CCTTATCACCAGAAATGGTTTCAGCGTCTGAAGTGATCAGCACGCAAGAAAACCAAACCTATACCTATGT
TCGCTGTTGGTATCGCACCAGCTACTCGAAAGATGATCCAGCGACCGATTGGGAATGGGCAAAAAACGAA
GATGGTAGCTACTTCACCATTGACGGCTACTGGTGGAGCTCCGTTTCATTTAAAAACATGTTCTACACCA
ACACGTCGCAAAACGTTATCCGTCAGCGTTGTGAAGCAACATTAGATTTGGCGAACGAGAACGCAGACAT
TACGTTCTTCGCCGCTGACAATCGCTTCTCATACAACCACACGATCTGGAGCAACGACGCAGCAATGCAG
CCAGATCAAATCAACAAAGTGGTTGCACTCGGTGACAGCTTGTCTGATACAGGCAACATCTTTAACGCAT
CACAATGGCGCTTCCCTAACCCGAACAGCTGGTTCTTAGGTCACTTCTCCAACGGTTTTGTTTGGACAGA
ATACATTGCCAAAGCGAAGAACCTTCCGCTCTACAACTGGGCAGTTGGCGGCGCGGCTGGTGAGAACCAA
TACATCGCGCTAACAGGGGTTGGTGAGCAAGTTTCTTCGTACTTAACCTACGCAAAACTGGCGAAGAACT
ACAAACCAGCAAACACCTTGTTTACGCTTGAGTTTGGTTTGAATGACTTCATGAACTACAACCGTGGCGT
TCCAGAAGTGAAAGCGGATTATGCAGAAGCACTGATTCGTTTGACGGACGCAGGTGCGAAGAACTTCATG
TTGATGACACTGCCAGATGCGACGAAAGCGCCTCAGTTTAAGTACTCAACACAAGAAGAGATCGACAAAA
TTCGTGCGAAAGTGCTTGAGATGAACGAGTTCATCAAGGCACAAGCGATGTACTACAAAGCGCAAGGTTA
CAACATCACGTTGTTTGATACTCACGCCTTGTTCGAGACGCTAACTTCTGCGCCAGAAGAGCACGGTTTC
GTGAACGCGAGCGATCCTTGTTTGGACATCAACCGCTCATCGTCTGTCGATTACATGTACACCCACGCAT
TGCGCTCTGAGTGTGCAGCGTCTGGTGCTGAGAAGTTTGTGTTCTGGGATGTCACGCATCCAACAACAGC
AACTCACCGCTATGTTGCAGAGAAAATGCTAGAAAGTAGCAACAACTTAGCCGAGTACCGTTTCTAA

>S064
ATGATGAAAAAAACAATCACACTATTAACTGCATTACTCCCGCTTGCTTCTGCAGTTGCCGAAGAGCCAACCTTAT
CACCAGAAATGGTTTCAGCGTCTGAAGTGATCAGCACGCAAGAAAACCAAACCTATACCTATGTTCGCTGTTGGTA
TCGCACCAGCTACTCGAAAGATGATCCAGCGACCGATTGGGAATGGGCAAAAAACGAAGATGGTAGCTACTTCAC
CATTGACGGCTACTGGTGGAGCTCCGTTTCATTTAAAAACATGTTCTACACCAACACGTCGCAAAACGTTATCCGT
CAGCGTTGTGAAGCAACATTAGATTTGGCGAACGAGAACGCAGACATTACGTTCTTCGCCGCTGACAATCGCTTCT
CATACAACCACACGATCTGGAGCAACGACGCAGCAATGCAGCCAGATCAAATCAACAAAGTGGTTGCACTCGGTG
ACAGCTTGTCTGATACAGGCAACATCTTTAACGCATCACAATGGCGCTTCCCTAACCCGAACAGCTGGTTCTTAGG
TCACTTCTCCAACGGTTTTGTTTGGACAGAATACATTGCCAAAGCGAAGAACCTTCCGCTCTACAACTGGGCAGTT
GGCGGCGCGGCTGGTGAGAACCAATACATCGCGCTAACAGGGGTTGGTGAGCAAGTTTCTTCGTACTTAACCTACG
CAAAACTGGCGAAGAACTACAAACCAGCAAACACCTTGTTTACGCTTGAGTTTGGTTTGAATGACTTCATGAACTA
CAACCGTGGCGTTCCAGAAGTGAAAGCGGATTATGCAGAAGCACTGATTCGTTTGACGGACGCAGGTGCGAAGAA
CTTCATGTTGATGACACTGCCAGATGCGACGAAAGCGCCTCAGTTTAAGTACTCAACACAAGAAGAGATCGACAA
AATTCGTGCGAAAGTGCTTGAGATGAACGAGTTCATCAAGGCACAAGCGATGTACTACAAAGCGCAAGGTTACAA
CATCACGTTGTTTGATACTCACGCCTTGTTCGAGACGCTAACTTCTGCGCCAGAAGAGCACGGTTTCGTGAACGCG

AGCGATCCTTGTTTGGACATCAACCGCTCATCGTCTGTCGATTACATGTACACCCACGCATTGCGCTCTGAGTGTGC
AGCGTCTGGTGCTGAGAAGTTTGTGTTCTGGGATGTCACGCATCCAACAACAGCAACTCACCGCTATGTTGCAGAG
AAAATGCTAGAAAGTAGCAACAACTTAGCCGAGTACCGTTTCTAA

>S063
ATGATGAAAAAAACAATCACACTATTAACTGCATTACTCCCGCTTGCTTCTGCAGTTGCCGAAGAGCCAACCTTAT
CACCAGAAATGGTTTCAGCGTCTGAAGTGATCAGCACGCAAGAAAACCAAACCTATACCTATGTTCGCTGTTGGTA
TCGCACCAGCTACTCGAAAGATGATCCAGCGACCGATTGGGAATGGGCAAAAAACGAAGATGGTAGCTACTTCAC
CATTGACGGCTACTGGTGGAGCTCCGTTTCATTTAAAAACATGTTCTACACCAACACGTCGCAAAACGTTATCCGT
CAGCGTTGTGAAGCAACATTAGATTTGGCGAACGAGAACGCAGACATTACGTTCTTCGCCGCTGACAATCGCTTCT
CATACAACCACACGATCTGGAGCAACGACGCAGCAATGCAGCCAGATCAAATCAACAAAGTGGTTGCACTCGGTG
ACAGCTTGTCTGATACAGGCAACATCTTTAACGCATCACAATGGCGCTTCCCTAACCCGAACAGCTGGTTCTTAGG
TCACTTCTCCAACGGTTTTGTTTGGACAGAATACATTGCCAAAGCGAAGAACCTTCCGCTCTACAACTGGGCAGTT
GGCGGCGCGGCTGGTGAGAACCAATACATCGCGCTAACAGGGGTTGGTGAGCAAGTTTCTTCGTACTTAACCTACG
CAAAACTGGCGAAGAACTACAAACCAGCAAACACCTTGTTTACGCTTGAGTTTGGTTTGAATGACTTCATGAACTA
CAACCGTGGCGTTCCAGAAGTGAAAGCGGATTATGCAGAAGCACTGATTCGTTTGACGGACGCAGGTGCGAAGAA
CTTCATGTTGATGACACTGCCAGATGCGACGAAAGCGCCTCAGTTTAAGTACTCAACACAAGAAGAGATCGACAA
AATTCGTGCGAAAGTGCTTGAGATGAACGAGTTCATCAAGGCACAAGCGATGTACTACAAAGCGCAAGGTTACAA
CATCACGTTGTTTGATACTCACGCCTTGTTCGAGACGCTAACTTCTGCGCCAGAAGAGCACGGTTTCGTGAACGCG
AGCGATCCTTGTTTGGACATCAACCGCTCATCGTCTGTCGATTACATGTACACCCACGCATTGCGCTCTGAGTGTGC
AGCGTCTGGTGCTGAGAAGTTTGTGTTCTGGGATGTCACGCATCCAACAACAGCAACTCACCGCTATGTTGCAGAG
AAAATGCTAGAAAGTAGCAACAACTTAGCCGAGTACCGTTTCTAA

>S062
ATGATGAAAAAAACAATCACACTATTAACTGCATTACTCCCGCTTGCTTCTGCAGTTGCCGAAGAGCCAA
CCTTATCACCAGAAATGGTTTCAGCGTCTGAAGTGATCAGCACGCAAGAAAACCAAACCTATACCTATGT
TCGCTGTTGGTATCGCACCAGCTACTCGAAAGATGATCCAGCGACCGATTGGGAATGGGCAAAAAACGAA
GATGGTAGCTACTTCACCATTGACGGCTACTGGTGGAGCTCCGTTTCATTTAAAAACATGTTCTACACCA
ACACGTCGCAAAACGTTATCCGTCAGCGTTGTGAAGCAACATTAGATTTGGCGAACGAGAACGCAGACAT
TACGTTCTTCGCCGCTGACAATCGCTTCTCATACAACCACACGATCTGGAGCAACGACGCAGCAATGCAG
CCAGATCAAATCAACAAAGTGGTTGCACTCGGTGACAGCTTGTCTGATACAGGCAACATCTTTAACGCAT
CACAATGGCGCTTCCCTAACCCGAACAGCTGGTTCTTAGGTCACTTCTCCAACGGTTTTGTTTGGACAGA
ATACATTGCCAAAGCGAAGAACCTTCCGCTCTACAACTGGGCAGTTGGCGGCGCGGCTGGTGAGAACCAA
TACATCGCGCTAACAGGGGTTGGTGAGCAAGTTTCTTCGTACTTAACCTACGCAAAACTGGCGAAGAACT
ACAAACCAGCAAACACCTTGTTTACGCTTGAGTTTGGTTTGAATGACTTCATGAACTACAACCGTGGCGT
TCCAGAAGTGAAAGCGGATTATGCAGAAGCACTGATTCGTTTGACGGACGCAGGTGCGAAGAACTTCATG
TTGATGACACTGCCAGATGCGACGAAAGCGCCTCAGTTTAAGTACTCAACACAAGAAGAGATCGACAAAA
TTCGTGCGAAAGTGCTTGAGATGAACGAGTTCATCAAGGCACAAGCGATGTACTACAAAGCGCAAGGTTA
CAACATCACGTTGTTTGATACTCACGCCTTGTTCGAGACGCTAACTTCTGCGCCAGAAGAGCACGGTTTC
GTGAACGCGAGCGATCCTTGTTTGGACATCAACCGCTCATCGTCTGTCGATTACATGTACACCCACGCAT
TGCGCTCTGAGTGTGCAGCGTCTGGTGCTGAGAAGTTTGTGTTCTGGGATGTCACGCATCCAACAACAGC
AACTCACCGCTATGTTGCAGAGAAAATGCTAGAAAGTAGCAACAACTTAGCCGAGTACCGTTTCTAA

>S060
ATGATGAAAAAAACAATCACACTATTAACTGCATTACTCCCGCTTGCTTCTGCAGTTGCCGAAGAGCCAA
CCTTATCACCAGAAATGGTTTCAGCGTCTGAAGTGATCAGCACGCAAGAAAACCAAACCTATACCTATGT
TCGCTGTTGGTATCGCACCAGCTACTCGAAAGATGATCCGGCGACCGATTGGGAATGGGCAAAAAACGAA
GATGGTAGCTACTTCACCATTGACGGCTACTGGTGGAGCTCCGTTTCATTTAAAAACATGTTCTACACCA
ACACGTCGCAAAACGTTATCCGTCAGCGTTGTGAAGCAACATTAGATTTGGCGAACGAGAACGCAGACAT
TACGTTCTTCGCCGCTGACAATCGCTTCTCATACAACCACACGATCTGGAGCAACGACGCAGCAATGCAG
CCAGATCAAATCAACAAAGTGGTTGCACTCGGTGACAGCTTGTCTGATACAGGCAACATCTTTAACGCAT

CACAATGGCGCTTCCCTAACCCGAACAGCTGGTTCTTAGGTCACTTCTCCAACGGTTTTGTGTGGACAGA
ATACATTGCCAAAGCGAAGAACCTTCCGCTCTACAACTGGGCAGTTGGCGGCGCGGCTGGTGAGAACCAA
TACATCGCGCTAACAGGGGTTGGTGATCAAGTTTCTTCGTACTTAACCTACGCAAAACTGGCGAAGAACT
ACAAACCAGCAAACACCTTGTTTACGCTTGAGTTTGGTTTGAATGACTTCATGAACTACAACCGTGGCGT
TCCAGAAGTGAAAGCGGATTATGCAGAAGCACTGATTCGTTTGACGGACGCAGGTGCGAAGAACTTCATG
TTGATGACACTGCCAGACGCGACGAAAGCGCCTCAGTTTAAGTACTCAACACAAGAAGAGATCGACAAAA
TTCGTGCGAAAGTGCTTGAGATGAACGAGTTCATCAAGGCACAAGCGATGTACTACAAAGCGCAAGGTTA
CAACATCACGTTGTTTGATACTCACGCCTTGTTCGAGACGCTAACTTCTGCGCCAGAAGAGCACGGTTTC
GTGAACGCGAGCGATCCTTGTTTGGACATCAACCGCTCATCGTCTGTCGACTACATGTACACCCACGCAT
TGCGCTCTGAGTGTGCGGCGTCCGGTGCTGAGAAATTTGTGTTCTGGGATGTCACGCACCCAACAACAGC
AACTCACCGCTATGTTGCAGAGAAAATGCTAGAAAGTAGCAACAACTTAGCCGAGTACCGTTTCTAA

>S058
ATGATGAAAAAAACAATCACACTATTAACTGCATTACTCCCGCTTGCTTCTGCAGTTGCCGAAGAGCCAA
CCTTATCACCAGAAATGGTTTCAGCGTCTGAAGTGATCAGCACGCAAGAAAACCAAACCTATACCTATGT
TCGCTGTTGGTATCGCACCAGCTACTCGAAAGATGATCCGGCGACCGATTGGGAATGGGCAAAAAACGAA
GATGGTAGCTACTTCACCATTGACGGCTACTGGTGGAGCTCCGTTTCATTTAAAAACATGTTCTACACCA
ACACGTCGCAAAACGTTATCCGTCAGCGTTGTGAAGCCACATTAGATTTGGCGAACGAGAACGCAGACAT
TACGTTCTTCGCCGCTGACAATCGCTTCTCATACAACCACACGATCTGGAGCAACGACGCAGCAATGCAG
CCAGATCAAATCAACAAAGTGGTTGCACTCGGTGACAGCTTGTCTGATACAGGCAACATCTTTAACGCAT
CACAATGGCGCTTCCCTAACCCGAACAGCTGGTTCTTAGGTCACTTCTCCAACGGTTTTGTGTGGACAGA
ATACATTGCCAAAGCGAAGAACCTTCCGCTCTACAACTGGGCAGTTGGCGGCGCGGCTGGTGAGAACCAA
TACATCGCGCTAACAGGGGTTGGTGATCAAGTTTCTTCGTACTTAACCTACGCAAAACTGGCGAAGAACT
ACAAACCAGCAAACACCTTGTTTACGCTTGAGTTTGGTTTGAATGACTTCATGAACTACAACCGTGGCGT
TCCAGAAGTGAAAGCGGATTATGCAGAAGCACTGATTCGTTTGACGGACGCAGGTGCGAAGAACTTCATG
TTGATGACACTGCCAGACGCGACGAAAGCGCCTCAGTTTAAGTACTCAACACAAGAAGAGATCGACAAAA
TTCGTGCGAAAGTGCTTGAGATGAACGAGTTCATCAAGGCACAAGCGATGTACTACAAAGCGCAAGGTTA
CAACATCACGTTGTTTGATACTCACGCCTTGTTCGAGACGCTAACTTCTGCGCCAGAAGAGCACGGTTTC
GTGAACGCGAGTGATCCTTGTTTGGACATCAACCGCTCATCGTCTGTCGATTACATGTACACCCACGCAT
TGCGCTCTGAGTGTGCAGCGTCTGGTGCTGAGAAGTTTGTATTCTGGGATGTCACGCACCCAACAACAGC
AACTCACCGCTATGTTGCAGAGAAAATGCTAGAAAGTAGCAACAACTTAGCCGAGTACCGTTTCTAA

>S057
ATGATGAAAAAAACAATCACACTATTAACTGCATTACTCCCGCTTGCTTCTGCAGTTGCCGAAGAGCCAACCTTAT
CACCAGAAATGGTTTCAGCGTCTGAAGTGATCAGCACGCAAGAAAACCAAACCTATACCTATGTTCGCTGTTGGTA
TCGCACCAGCTACTCGAAAGATGATCCGGCGACCGATTGGGAATGGGCAAAAAACGAAGATGGTAGCTACTTCAC
CATTGACGGCTACTGGTGGAGCTCCGTTTCATTTAAAAACATGTTCTACACCAACACGTCGCAAAACGTTATCCGT
CAGCGTTGTGAAGCAACATTAGATTTGGCGAACGAGAACGCAGACATTACGTTCTTCGCCGCTGACAATCGCTTCT
CATACAACCACACGATCTGGAGCAACGACGCAGCAATGCAGCCAGATCAAATCAACAAAGTGGTTGCACTCGGTG
ACAGCTTGTCTGATACAGGCAACATCTTTAACGCATCACAATGGCGCTTCCCTAACCCGAACAGCTGGTTCTTAGG
TCACTTCTCCAACGGTTTTGTGTGGACAGAATACATTGCCAAAGCGAAGAACCTTCCGCTCTACAACTGGGCAGTT
GGCGGCGCGGCTGGTGAGAACCAATACATCGCACTAACAGGGGTTGGTGATCAAGTTTCTTCGTACTTAACCTACG
CAAAACTGGCGAAGAACTACAAACCAGCAAACACCTTGTTTACGCTTGAGTTTGGTTTGAATGACTTCATGAACTA
CAACCGTGGCGTTCCAGAAGTGAAAGCAGATTATGCAGAAGCACTGATTCGTTTGACGGACGCAGGTGCGAAGAA
CTTCATGTTGATGACACTGCCAGATGCGACGAAAGCGCCTCAGTTTAAGTACTCAACACAAGAAGAGATCGACAA
AATTCGTGCGAAAGTGCTTGAGATGAACGAGTTCATCAAGGCACAAGCGATGTACTACAAAGCGCAAGGTTACAA
CATCACGTTGTTTGATACTCACGCCTTGTTCGAGACGCTAACTTCTGCGCCAGAAGAGCACGGTTTCGTGAACGCG
AGTGATCCTTGTTTGGACATCAACCGCTCATCGTCTGTCGATTACATGTACACCCACGCATTGCGCTCTGAGTGTGC
AGCGTCTGGTGCTGAGAAGTTTGTGTTCTGGGATGTCACGCACCCAACAACAGCAACTCACCGCTATGTTGCAGAG
AAAATGCTAGAAAGTAGCAACAACTTAGCAGAGTACCGTTTCTAA

>S056
ATGATGAAAAAAACAATCACACTATTAACTGCATTACTCCCGCTTGCTTCTGCAGTTGCCGAAGAGCCAACCTTAT
CACCAGAAATGGTTTCAGCGTCTGAAGTGATCAGCACGCAAGAAAACCAAACCTATACCTATGTTCGCTGTTGGTA
TCGCACCAGCTACTCGAAAGATGATCCGGCGACCGATTGGGAATGGGCAAAAAACGAAGATGGTAGCTACTTCAC
CATTGACGGCTACTGGTGGAGCTCCGTTTCATTTAAAAACATGTTCTACACCAACACGTCGCAAAACGTTATCCGT
CAGCGTTGTGAAGCAACATTAGATTTGGCGAACGAGAACGCAGACATTACGTTCTTCGCCGCTGACAATCGCTTCT
CATACAACCACACGATCTGGAGCAACGACGCAGCAATGCAGCCAGATCAAATCAACAAAGTGGTTGCACTCGGTG
ACAGCTTGTCTGATACAGGCAACATCTTTAACGCATCACAATGGCGCTTCCCTAACCCGAACAGCTGGTTCTTAGG
TCACTTCTCCAACGGTTTTGTGTGGACAGAATACATTGCCAAAGCGAAGAACCTTCCGCTCTACAACTGGGCAGTT
GGCGGCGCGGCTGGTGAGAACCAATACATCGCACTAACAGGGGTTGGTGATCAAGTTTCTTCGTACTTAACCTACG
CAAAACTGGCGAAGAACTACAAACCAGCAAACACCTTGTTTACGCTTGAGTTTGGTTTGAATGACTTCATGAACTA
CAACCGTGGCGTTCCAGAAGTGAAAGCAGATTATGCAGAAGCACTGATTCGTTTGACGGACGCAGGTGCGAAGAA
CTTCATGTTGATGACACTGCCAGATGCGACGAAAGCGCCTCAGTTTAAGTACTCAACACAAGAAGAGATCGACAA
AATTCGTGCGAAAGTGCTTGAGATGAACGAGTTCATCAAGGCACAAGCGATGTACTACAAAGCGCAAGGTTACAA
CATCACGTTGTTTGATACTCACGCCTTGTTCGAGACGCTAACTTCTGCGCCAGAAGAGCACGGTTTCGTGAACGCG
AGTGATCCTTGTTTGGACATCAACCGCTCATCGTCTGTCGATTACATGTACACCCACGCATTGCGCTCTGAGTGTGC
AGCGTCTGGTGCTGAGAAGTTTGTGTTCTGGGATGTCACGCACCCAACAACAGCAACTCACCGCTATGTTGCAGAG
AAAATGCTAGAAAGTAGCAACAACTTAGCAGAGTACCGTTTCTAA

>S055
ATGATGAAAAAAACAATCACACTATTAACTGCATTACTCCCGCTTGCTTCTGCAGTTGCCGAAGAGCCAA
CCTTATCACCAGAAATGGTTTCAGCGTCTGAAGTGATCAGCACGCAAGAAAACCAAACCTATACCTATGT
TCGCTGTTGGTATCGCACCAGCTACTCGAAAGATGATCCGGCGACCGATTGGGAATGGGCAAAAAACGAA
GATGGTAGCTACTTCACCATTGACGGCTACTGGTGGAGCTCCGTTTCATTTAAAAACATGTTCTACACCA
ACACGTCGCAAAACGTTATCCGTCAGCGTTGTGAAGCAACATTAGATTTGGCGAACGAGAACGCAGACAT
TACGTTCTTCGCCGCTGACAATCGCTTCTCATACAACCACACGATCTGGAGCAACGACGCAGCAATGCAG
CCAGATCAAATCAACAAAGTGGTTGCACTCGGTGACAGCTTGTCTGATACAGGCAACATCTTTAACGCAT
CACAATGGCGCTTCCCTAACCCGAACAGCTGGTTCTTAGGTCACTTCTCCAACGGTTTTGTGTGGACAGA
ATACATTGCCAAAGCGAAGAACCTTCCGCTCTACAACTGGGCAGTTGGCGGCGCGGCTGGTGAGAACCAA
TACATCGCACTAACAGGGGTTGGTGATCAAGTTTCTTCGTACTTAACCTACGCAAAACTGGCGAAGAACT
ACAAACCAGCAAACACCTTGTTTACGCTTGAGTTTGGTTTGAATGACTTCATGAACTACAACCGTGGCGT
TCCAGAAGTGAAAGCAGATTATGCAGAAGCACTGATTCGTTTGACGGACGCAGGTGCGAAGAACTTCATG
TTGATGACACTGCCAGATGCGACGAAAGCGCCTCAGTTTAAGTACTCAACACAAGAAGAGATCGACAAAA
TTCGTGCGAAAGTGCTTGAGATGAACGAGTTCATCAAGGCACAAGCGATGTACTACAAAGCGCAAGGTTA
CAACATCACGTTGTTTGATACTCACGCCTTGTTCGAGACGCTAACTTCTGCGCCAGAAGAGCACGGTTTC
GTGAACGCGAGTGATCCTTGTTTGGACATCAACCGCTCATCGTCTGTCGATTACATGTACACCCACGCAT
TGCGCTCTGAGTGTGCAGCGTCTGGTGCTGAGAAGTTTGTGTTCTGGGATGTCACGCACCCAACAACAGC
AACTCACCGCTATGTTGCAGAGAAAATGCTAGAAAGTAGCAACAACTTAGCAGAGTACCGTTTCTAA

>S054
ATGATGAAAAAAACAATCACACTATTAACTGCATTACTCCCGCTTGCTTCTGCAGTTGCCGAAGAGCCAA
CCTTATCACCAGAAATGGTTTCAGCGTCTGAAGTGATCAGCACGCAAGAAAACCAAACCTATACCTATGT
TCGCTGTTGGTATCGCACCAGCTACTCGAAAGATGATCCGGCGACCGATTGGGAATGGGCAAAAAACGAA
GATGGTAGCTACTTCACCATTGACGGCTACTGGTGGAGCTCCGTTTCATTTAAAAACATGTTCTACACCA
ACACGTCGCAAAACGTTATCCGTCAGCGTTGTGAAGCAACATTAGATTTGGCGAACGAGAACGCAGACAT
TACGTTCTTCGCCGCTGACAATCGCTTCTCATACAACCACACGATCTGGAGCAACGACGCAGCAATGCAG
CCAGATCAAATCAACAAAGTGGTTGCACTCGGTGACAGCTTGTCTGATACAGGCAACATCTTTAACGCAT
CACAATGGCGCTTCCCTAACCCGAACAGCTGGTTCTTAGGTCACTTCTCCAACGGTTTTGTGTGGACAGA
ATACATTGCCAAAGCGAAGAACCTTCCGCTCTACAACTGGGCAGTTGGCGGCGCGGCTGGTGAGAACCAA
TACATCGCACTAACAGGGGTTGGTGATCAAGTTTCTTCGTACTTAACCTACGCAAAACTGGCGAAGAACT
ACAAACCAGCAAACACCTTGTTTACGCTTGAGTTTGGTTTGAATGACTTCATGAACTACAACCGTGGCGT

TCCAGAAGTGAAAGCAGATTATGCAGAAGCACTGATTCGTTTGACGGACGCAGGTGCGAAGAACTTCATG
TTGATGACACTGCCAGATGCGACGAAAGCGCCTCAGTTTAAGTACTCAACACAAGAAGAGATCGACAAAA
TTCGTGCGAAAGTGCTTGAGATGAACGAGTTCATCAAGGCACAAGCGATGTACTACAAAGCGCAAGGTTA
CAACATCACGTTGTTTGATACTCACGCCTTGTTCGAGACGCTAACTTCTGCGCCAGAAGAGCACGGTTTC
GTGAACGCGAGTGATCCTTGTTTGGACATCAACCGCTCATCGTCTGTCGATTACATGTACACCCACGCAT
TGCGCTCTGAGTGTGCAGCGTCTGGTGCTGAGAAGTTTGTGTTCTGGGATGTCACGCACCCAACAACAGC
AACTCACCGCTATGTTGCAGAGAAAATGCTAGAAAGTAGCAACAACTTAGCAGAGTACCGTTTCTAA

>S053
ATGATGAAAAAAACAATCACACTATTAACTGCATTACTCCCGCTTGCTTCTGCAGTTGCCGAAGAGCCAA
CCTTATCACCAGAAATGGTTTCAGCGTCTGAAGTGATCAGCACGCAAGAAAACCAAACCTATACCTATGT
TCGCTGTTGGTATCGCACCAGCTACTCGAAAGATGATCCGGCGACCGATTGGGAATGGGCAAAAAACGAA
GATGGTAGCTACTTCACCATTGACGGCTACTGGTGGAGCTCCGTTTCATTTAAAAACATGTTCTACACCA
ACACGTCGCAAAACGTTATCCGTCAGCGTTGTGAAGCAACATTAGATTTGGCGAACGAGAACGCAGACAT
TACGTTCTTCGCCGCTGACAATCGCTTCTCATACAACCACACGATCTGGAGCAACGACGCAGCAATGCAG
CCAGATCAAATCAACAAAGTGGTTGCACTCGGTGACAGCTTGTCTGATACAGGCAACATCTTTAACGCAT
CACAATGGCGCTTCCCTAACCCGAACAGCTGGTTCTTAGGTCACTTCTCCAACGGTTTTGTGTGGACAGA
ATACATTGCCAAAGCGAAGAACCTTCCGCTCTACAACTGGGCAGTTGGCGGCGCGGCTGGTGAGAACCAA
TACATCGCGCTAACAGGGGTTGGTGATCAAGTTTCTTCGTACTTAACCTACGCAAAACTGGCGAAGAACT
ACAAACCAGCAAACACCTTGTTTACGCTTGAGTTTGGTTTGAATGACTTCATGAACTACAACCGTGGCGT
TCCAGAAGTGAAAGCGGATTATGCAGAAGCACTGATTCGTTTGACGGACGCAGGTGCGAAGAACTTCATG
TTGATGACACTGCCAGACGCGACGAAAGCGCCTCAGTTTAAGTACTCAACACAAGAAGAGATCGACAAAA
TTCGTGCGAAAGTGCTTGAGATGAACGAGTTCATCAAGGCACAAGCGATGTACTACAAAGCGCAAGGTTA
CAACATCACGTTGTTTGATACTCACGCCTTGTTCGAGACGCTAACTTCTGCGCCAGAAGAGCACGGTTTC
GTGAACGCGAGCGATCCTTGTTTGGACATCAACCGCTCATCGTCTGTCGATTACATGTACACCCACGCAT
TGCGCTCTGAGTGTGCAGCGTCTGGTGCTGAGAAGTTTGTGTTCTGGGATGTCACGCATCCAACAACAGC
AACTCACCGCTATGTTGCAGAGAAAATGCTAGAAAGTAGCAACAACTTAGCCGAGTACCGTTTCTAA

>S052
ATGATGAAAAAAACAATCACACTATTAACTGCATTACTCCCGCTTGCTTCTGCAGTTGCCGAAGAGCCAACCTTAT
CACCAGAAATGGTTTCAGCGTCTGAAGTGATCAGCACGCAAGAAAACCAAACCTATACCTATGTTCGCTGTTGGTA
TCGCACCAGCTACTCGAAAGATGATCCGGCGACCGATTGGGAATGGGCAAAAAACGAAGATGGTAGCTACTTCAC
CATTGACGGCTACTGGTGGAGCTCCGTTTCATTTAAAAACATGTTCTACACCAACACGTCGCAAAACGTTATCCAT
CAGCGTTGTGAAGCAACATTAGATTTGGCGAACGAGAACGCAGACATTACGTTCTTCGCCGCTGACAATCGCTTCT
CATACAACCACACGATCTGGAGCAACGACGCAACAATGCAGCCAGATCAAATCAACAAAGTGGTTGCACTCGGTG
ACAGCTTGTCTGATACAGGCAACATCTTTAACGCATCACAATGGCGCTTCCCTAACCCGAACAGCTGGTTCTTAGG
TCACTTCTCCAACGGTTTTGTGTGGACAGAATACATTGCCAAAGCGAAGAACCTTCCGCTCTACAACTGGGCAGTT
GGCGGCGCGGCTGGTGAGAACCAATACATCGCGCTAACAGGGGTTGGTGATCAAGTTTCTTCGTACTTAACCTACG
CAAAACTGGCGAAGAACTACAAACCAGCAAACACCTTGTTTACGCTTGAGTTTGGTTTGAATGACTTCATGAACTA
CAACCGTGGCGTTCCAGAAGTGAAAGCGGATTATGCAGAAGCACTGATTCGTTTGACGGACGCAGGTGCGAAGAA
CTTCATGTTGATGACACTGCCAGACGCGACGAAAGCGCCTCAGTTTAAGTACTCAACACAAGAAGAGATCGACAA
AATTCGTGCGAAAGTGCTTGAGATGAACGAGTTCATCAAGGCACAAGCGATGTACTACAAAGCGCAAGGTTACAA
TATCACGTTGTTTGATACTCACGCCTTGTTCGAGACGCTAACTTCTGCGCCAGAAGAGCACGGTTTCGTGAACGCG
AGCGATCCTTGTTTGGACATCAACCGCTCATCGTCTGTCGACTACATGTACACCCACGCATTGCGCTCTGAGTGTGC
GGCGTCTGGTGCTGAGAAGTTTGTGTTCTGGGATGTGACTCACCCAACTACAGCCACGCATCGTTATGTTGCTGAA
AAAATGTTGGAAAGCAGCAATAACTTAGAAGAGTTTCGCTTTTAA

>S049
ATGATGAAAAAAACAATCACACTATTAACTGCATTACTCCCGCTTGCTTCTGCAGTTGCCGAAGAGCCAA
CCTTATCACCAGAAATGGTTTCAGCGTCTGAAGTGATCAGCACGCAAGAAAACCAAACCTATACCTATGT
TCGCTGTTGGTATCGCACCAGCTACTCGAAAGATGATCCGGCGACCGATTGGGAATGGGCAAAAAACGAA

GATGGTAGCTACTTCACCATTGACGGCTACTGGTGGAGCTCCGTTTCATTTAAAAACATGTTCTACACCA
ACACGTCGCAAAACGTTATCCGTCAGCGTTGTGAAGCAACATTAGATTTGGCGAACGAGAACGCAGACAT
TACGTTCTTCGCCGCTGACAATCGCTTCTCATACAACCACACGATCTGGAGCAACGACGCAGCAATGCAG
CCAGATCAAATCAACAAAGTGGTTGCACTCGGTGACAGCTTGTCTGATACAGGCAACATCTTTAACGCAT
CACAATGGCGCTTCCCTAACCCGAACAGCTGGTTCTTAGGTCACTTCTCCAACGGTTTTGTGTGGACAGA
ATACATTGCCAAAGCGAAGAACCTTCCGCTCTACAACTGGGCAGTTGGCGGCGCGGCTGGTGAGAACCAA
TACATCGCGCTAACAGGGGTTGGTGATCAAGTTTCTTCGTACTTAACCTACGCAAAACTGGCGAAGAACT
ACAAACCAGCAAACACCTTGTTTACGCTTGAGTTTGGTTTGAATGACTTCATGAACTACAACCGTGGCGT
TCCAGAAGTGAAAGCGGATTATGCAGAAGCACTGATTCGTTTGACGGACGCAGGTGCGAAGAACTTCATG
TTGATGACACTGCCAGACGCGACGAAAGCGCCTCAGTTTAAGTACTCAACACAAGAAGAGATCGACAAAA
TTCGTGCGAAAGTGCTTGAGATGAACGAGTTCATCAAGGCACAAGCGATGTACTACAAAGCGCAAGGTTA
CAACATCACGTTGTTTGATACTCACGCCTTGTTCGAGACGCTAACTTCTGCGCCAGAAGAGCACGGTTTC
GTGAACGCGAGCGATCCTTGTTTGGACATCAACCGCTCATCGTCTGTCGATTACATGTACACCCACGCAT
TGCGCTCTGAGTGTGCTGCGTCTGGTGCTGAGAAGTTTGTGTTCTGGGATGTCACGCATCCAACAACAGC
AACTCACCGCTATGTTGCAGAGAAAATGCTAGAAAGTAGCAACAACTTAGCAGAGTACCGTTTCTAA

>S048
ATGATGAAAAAAACAATCACACTATTAACTGCATTACTCCCGCTTGCTTCTGCAGTTGCCGAAGAGCCAA
CCTTATCACCAGAAATGGTTTCAGCGTCTGAAGTGATCAGCACGCAAGAAAACCAAACCTATACCTATGT
TCGCTGTTGGTATCGCACCAGCTACTCGAAAGATGATCCGGCGACCGATTGGGAATGGGCAAAAAACGAA
GATGGTAGCTACTTCACCATTGACGGCTACTGGTGGAGCTCCGTTTCATTTAAAAACATGTTCTACACCA
ACACGTCGCAAAACGTTATCCGTCAGCGTTGTGAAGAAACATTAGATTTGGCGAACGAGAACGCAGACAT
TACGTTCTTCGCCGCTGACAATCGCTTCTCATACAACCACACGATCTGGAGCAACGACGCAGCAATGCAG
CCAGATCAAATCAACAAAGTGGTTGCACTCGGTGACAGCTTGTCTGATACAGGCAACATCTTTAACGCAT
CACAATGGCGCTTCCCTAACCCGAACAGCTGGTTCTTAGGTCACTTCTCCAACGGTTTTGTGTGGACAGA
ATACATTGCCAAAGCGAAGAACCTTCCGCTCTACAACTGGGCAGTTGGCGGCGCGGCTGGTGAGAACCAA
TACATCGCGCTAACAGGGGTTGGTGATCAAGTTTCTTCGTACTTAACCTACGCAAAACTGGCGAAGAACT
ACAAACCAGCAAACACCTTGTTTACGCTTGAGTTTGGTTTGAATGACTTCATGAACTACAACCGTGGCGT
TCCAGAAGTGAAAGCGGATTATGCAGAAGCACTGATTCGTTTGACGGACGCAGGTGCGAAGAACTTCATG
TTGATGACACTGCCAGATGCGACGAAAGCGCCTCAGTTTAAGTACTCAACACAAGAAGAGATCGACAAAA
TTCGTGCGAAAGTGCTTGAGATGAACGAGTTCATCAAGGCACAAGCGATGTACTACAAAGCGCAAGGTTA
CAACATCACGTTGTTTGATACTCACGCCTTGTTCGAAACGCTAACTTCTGCGCCAGAAGAGCACGGTTTC
GTGAACGCGAGCGATCCTTGTTTGGACATCAACCGCTCATCGTCTGTCGATTACATGTACACCCACGCAT
TGCGCTCTGAGTGTGCAGCGTCTGGTGCTGAGAAATTTGTGTTCTGGGATGTCACGCATCCAACAACAGC
AACTCACCGCTATGTTGCAGAGAAAATGCTAGAAAGTAGCAACAACTTAGCCGAGTACCGTTTCTAA

>S047
ATGATGAAAAAAACAATCACACTATTAACTGCATTACTCCCGCTTGCTTCTGCAGTTGCCGAAGAGCCAACCTTAT
CACCAGAAATGGTTTCAGCGTCTGAAGTGATCAGCACGCAAGAAAACCAAACCTATACCTATGTTCGCTGTTGGTA
TCGCACCAGCTACTCGAAAGATGATCCGGCGACCGATTGGGAATGGGCAAAAAACGAAGATGGTAGCTACTTCAC
CATTGACGGCTACTGGTGGAGCTCCGTTTCATTTAAAAACATGTTCTACACCAACACGTCGCAAAATGTTATCCGT
CAGCGTTGTGAAGCAACATTAGATTTGGCGAACGAGAACGCAGACATTACGTTCTTCGCCGCTGACAATCGCTTCT
CATACAACCACACGATCTGGAGCAACGACGCAGCAATGCAGCCAGATCAAATCAACAAAGTGGTTGCACTCGGTG
ACAGCTTGTCTGATACAGGCAACATCTTTAATGCGTCACAATGGCGCTTCCCTAACCCGAACAGCTGGTTCTTAGG
TCACTTCTCCAACGGTTTTGTGTGGACAGAATACATTGCCAAAGCGAAGAACCTTCCGCTCTACAACTGGGCAGTT
GGCGGTGCGGCTGGTGAGAACCAATACATCGCGCTAACAGGGGTTGGTGAGCAAGTTTCTTCGTACTTAACCTACG
CAAAACTGGCGAAGAACTACAAACCAGCAAACACCTTGTTTACGCTTGAGTTTGGTTTGAATGACTTCATGAACTA
CAACCGTGGCGTTCCAGAAGTGAAAGCGGATTATGCAGAAGCACTGATTCGTTTGACGGACGCAGGTGCGAAGAA
CTTCATGTTGATGACACTGCCAGACGCGACGAAAGCGCCTCAGTTTAAGTACTCAACACAAGAAGAGATCGACAA
AATTCGTGCGAAAGTGCTTGAGATGAACGAGTTCATCAAGGCACAAGCGATGTACTACAAAGCGCAAGGTTACAA
CATCACGTTGTTTGATACTCACGCCTTGTTCGAGACGCTAACTTCTGCGCCAGAAGAGCACGGTTTCGTGAACGCG

AGCGATCCTTGTTTGGACATCAACCGCTCATCGTCTGTCGACTACATGTACACCCACGCATTGCGCTCTGAGTGTGC
GGCGTCTGGTGCTGAGAAGTTTGTATTCTGGGATGTCACGCACCCAACAACAGCAACTCACCGCTATGTTGCAGAG
AAAATGCTAGAAAGTAGCAACAACTTAGCCGAGTACCGTTTCTAA

>S046
ATGATGAAAAAAACAATCACACTATTAACTGCATTACTCCCGCTTGCTTCTGCAGTTGCCGAAGAGCCAA
CCTTATCACCAGAAATGGTTTCAGCGTCTGAAGTGATCAGCACGCAAGAAAACCAAACCTATACCTATGT
TCGCTGTTGGTATCGCACCAGCTACTCGAAAGATGATCCGGCGACCGATTGGGAATGGGCAAAAAACGAA
GATGGTAGCTACTTCACCATTGACGGCTACTGGTGGAGCTCCGTTTCATTTAAAAACATGTTCTACACCA
ACACGTCGCAAAACGTTATCCGTCAGCGTTGTGAAGCAACATTAGATTTGGCGAACGAGAACGCAGACAT
TACGTTCTTCGCCGCTGACAATCGCTTCTCATACAACCACACGATCTGGAGCAACGACGCAGCAATGCAG
CCAGATCAAATCAACAAAGTGGTTGCACTCGGTGACAGCTTGTCTGATACAGGCAACATCTTTAACGCAT
CACAATGGCGCTTCCCTAACCCGAACAGCTGGTTCTTAGGTCACTTCTCCAACGGTTTTGTGTGGACAGA
ATACATTGCCAAAGCGAAGAACCTTCCGCTCTACAACTGGGCAGTTGGCGGCGCGGCTGGTGAGAACCAA
TACATCGCGCTAACAGGGGTTGGTGATCAAGTTTCTTCGTACTTAACCTACGCAAAACTGGCGAAGAACT
ACAAACCAGCAAACACCTTGTTTACGCTTGAGTTTGGTTTGAATGACTTCATGAACTACAACCGTGGCGT
TCCAGAAGTGAAAGCGGATTATGCAGAAGCACTGATTCGTTTGACGGACGCAGGTGCGAAGAACTTCATG
TTGATGACACTGCCAGACGCGACGAAAGCGCCTCAGTTTAAGTACTCAACACAAGAAGAGATCGACAAAA
TTCGTGCGAAAGTGCTTGAGATGAACGAGTTCATCAAGGCACAAGCGATGTACTACAAAGCGCAAGGTTA
CAACATCACGTTGTTTGATACTCACGCCTTGTTCGAGACGCTAACTTCTGCGCCAGAAGAGCACGGTTTC
GTGAACGCGAGCGATCCTTGTTTGGACATCAACCGCTCATCGTCTGTCGATTACATGTACACCCACGCAT
TGCGCTCTGAGTGTGCAGCGTCTGGTGCTGAGAAGTTTGTGTTCTGGGATGTCACGCATCCAACAACTGC
AACTCACCGCTATGTTGCAGAGAAAATGCTAGAAAGTAGCAACAACTTAGCCGAGTACCGTTTCTAA

>S045
ATGATGAAAAAAACAATCACACTATTAACTGCATTACTCCCGCTTGCTTCTGCAGTTGCCGAAGAGCCAACCTTAT
CACCAGAAATGGTTTCAGCGTCTGAAGTGATCAGCACGCAAGAAAACCAAACCTATACCTATGTTCGCTGTTGGTA
TCGCACCAGCTACTCGAAAGATGATCCGGCGACCGATTGGGAATGGGCAAAAAACGAAGATGGTAGCTACTTCAC
CATTGACGGCTACTGGTGGAGCTCCGTTTCATTTAAAAACATGTTCTACACCAACACGTCGCAAAACGTTATCCGT
CAGCGTTGTGAAGCAACATTAGATTTGGCGAACGAGAACGCAGACATTACGTTCTTCGCCGCTGACAATCGCTTCT
CATACAACCACACGATCTGGAGCAACGACGCAGCAATGCAGCCAGATCAAATCAACAAAGTGGTTGCACTCGGTG
ACAGCTTGTCTGATACAGGCAACATCTTTAACGCATCACAATGGCGCTTCCCTAACCCGAACAGCTGGTTCTTAGG
TCACTTCTCCAACGGTTTTGTGTGGACAGAATACATTGCCAAAGCGAAGAACCTTCCGCTCTACAACTGGGCAGTT
GGCGGCGCGGCTGGTGAGAACCAATACATCGCGCTAACAGGGGTTGGTGATCAAGTTTCTTCGTACTTAACCTACG
CAAAACTGGCGAAGAACTACAAACCAGCAAACACCTTGTTTACGCTTGAGTTTGGTTTGAATGACTTCATGAACTA
CAACCGTGGCGTTCCAGAAGTGAAAGCGGATTATGCAGAAGCACTGATTCGTTTGACGGACGCAGGTGCGAAGAA
CTTCATGTTGATGACACTGCCAGACGCGACGAAAGCGCCTCAGTTTAAGTACTCAACACAAGAAGAGATCGACAA
AATTCGTGCGAAAGTGCTTGAGATGAACGAGTTCATCAAGGCACAAGCGATGTACTACAAAGCGCAAGGTTACAA
CATCACGTTGTTTGATACTCACGCCTTGTTCGAGACGCTAACTTCTGCGCCAGAAGAGCACGGTTTCGTGAACGCG
AGCGATCCTTGTTTGGACATCAACCGCTCATCGTCTGTCGATTACATGTACACCCACGCATTGCGCTCTGAGTGTGC
AGCGTCTGGTGCTGAGAAGTTTGTGTTCTGGGATGTCACGCATCCAACAACAGCAACTCACCGCTATGTTGCAGAG
AAAATGCTAGAAAGTAGCAACAACTTAGCCGAGTACCGTTTCTAA

>S044
ATGATGAAAAAAACAATCACACTATTAACTGCATTACTCCCGCTTGCTTCTGCAGTTGCCGAAGAGCCAA
CCTTATCACCAGAAATGGTTTCAGCGTCTGAAGTGATCAGCACGCAAGAAAACCAAACCTATACCTATGT
TCGCTGTTGGTATCGCACCAGCTACTCGAAAGATGATCCGGCGACCGATTGGGAATGGGCAAAAAACGAA
GATGGTAGCTACTTCACCATTGACGGCTACTGGTGGAGCTCCGTTTCATTTAAAAACATGTTCTACACCA
ACACGTCGCAAAACGTTATCCGTCAGCGTTGTGAAGCAACATTAGATTTGGCGAACGAGAACGCAGACAT
TACGTTCTTCGCCGCTGACAATCGCTTCTCATACAACCACACGATCTGGAGCAACGACGCAGCAATGCAG
CCAGATCAAATCAACAAAGTGGTTGCACTCGGTGACAGCTTGTCTGATACAGGCAACATCTTTAACGCAT

CACAATGGCGCTTCCCTAACCCGAACAGCTGGTTCTTAGGTCACTTCTCCAACGGTTTTGTGTGGACAGA
ATACATTGCCAAAGCGAAGAACCTTCCGCTCTACAACTGGGCAGTTGGCGGCGCGGCTGGTGAGAACCAA
TACATCGCGCTAACAGGGGTTGGTGATCAAGTTTCTTCGTACTTAACCTACGCAAAACTGGCGAAGAACT
ACAAACCAGCAAACACCTTGTTTACGCTTGAGTTTGGTTTGAATGACTTCATGAACTACAACCGTGGCGT
TCCAGAAGTGAAAGCGGATTATGCAGAAGCACTGATTCGTTTGACGGACGCAGGTGCGAAGAACTTCATG
TTGATGACACTGCCAGACGCGACGAAAGCGCCTCAGTTTAAGTACTCAACACAAGAAGAGATCGACAAAA
TTCGTGCGAAAGTGCTTGAGATGAACGAGTTCATCAAGGCACAAGCGATGTACTACAAAGCGCAAGGTTA
CAACATCACGTTGTTTGATACTCACGCCTTGTTCGAGACGCTAACTTCTGCGCCAGAAGAGCACGGTTTC
GTGAACGCGAGCGATCCTTGTTTGGACATCAACCGCTCATCGTCTGTCGATTACATGTACACCCACGCAT
TGCGCTCTGAGTGTGCAGCGTCTGGTGCTGAGAAGTTTGTGTTCTGGGATGTCACGCATCCAACAACAGC
AACTCACCGCTATGTTGCAGAGAAAATGCTAGAAAGTAGCAACAACTTAGCCGAGTACCGTTTCTAA

>S043
ATGATGAAAAAAACAATCACACTATTAACTGCATTACTCCCGCTTGCTTCTGCAGTTGCCGAAGAGCCAACCTTAT
CACCAGAAATGGTTTCAGCGTCTGAAGTGATCAGCACGCAAGAAAACCAAACCTATACCTATGTTCGCTGTTGGTA
TCGCACCAGCTACTCGAAAGATGATCCGGCGACCGATTGGGAATGGGCAAAAAACGAAGATGGTAGCTACTTCAC
CATTGACGGCTACTGGTGGAGCTCCGTTTCATTTAAAAACATGTTCTACACCAACACGTCGCAAAACGTTATCCGT
CAGCGTTGTGAAGCCACATTAGATTTGGCGAACGAGAACGCAGACATTACGTTCTTCGCCGCTGACAATCGCTTCT
CATACAACCACACGATCTGGAGCAACGACGCAGCAATGCAGCCAGATCAAATCAACAAAGTGGTTGCACTCGGTG
ACAGCTTGTCTGATACAGGCAACATCTTTAACGCATCACAATGGCGCTTCCCTAACCCGAACAGCTGGTTCTTAGG
TCACTTCTCCAACGGTTTTGTGTGGACAGAATACATTGCCAAAGCGAAGAACCTTCCGCTCTACAACTGGGCAGTT
GGCGGCGCGGCTGGTGAGAACCAATACATCGCGCTAACAGGGGTTGGTGATCAAGTTTCTTCGTACTTAACCTACG
CAAAACTGGCGAAGAACTACAAACCAGCAAACACCTTGTTTACGCTTGAGTTTGGTTTGAATGACTTCATGAACTA
CAACCGTGGCGTTCCAGAAGTGAAAGCAGATTATGCAGAAGCACTGATTCGTTTGACGGACGCAGGTGCGAAGAA
CTTCATGTTGATGACACTGCCAGACGCGACGAAAGCGCCTCAGTTTAAGTACTCAACACAAGAAGAGATCGACAA
AATTCGTGCGAAAGTGCTTGAGATGAACGAGTTCATCAAGGCACAAGCGATGTACTACAAAGCGCAAGGTTACAA
CATCACGTTGTTTGATACTCACGCCTTGTTCGAGACGCTAACTTCTGCGCCCGAAAAGCACGGTTTCGTGAACGCG
AGTGATCCTTGTTTGGACATCAACCGCTCATCGTCTGTCGATTACATGTACACCCACGCATTGCGCTCTGAGTGTGC
GGCGTCTGGTGCTGAGAAGTTTGTGTTCTGGGATGTCACGCACCCAACAACAGCAACTCACCGCTATGTTGCAGAG
AAAATGCTAGAAAGTAGCAACAACTTAGCCGAGTACCGTTTCTAA

>S042
ATGATGAAAAAAACAATCACACTATTAACTGCATTACTCCCGCTTGCTTCTGCAGTTGCCGAAGAGCCAACCTTAT
CACCAGAAATGGTTTCAGCGTCTGAAGTGATCAGCACGCAAGAAAACCAAACCTATACCTATGTTCGCTGTTGGTA
TCGCACCAGCTACTCGAAAGATGATCCGGCGACCGATTGGGAATGGGCAAAAAACGAAGATGGTAGCTACTTCAC
CATTGACGGCTACTGGTGGAGCTCCGTTTCATTTAAAAACATGTTCTACACCAACACGTCGCAAAACGTTATCCGT
CAGCGTTGTGAAGCAACATTAGATTTGGCGAACGAGAACGCAGACATTACGTTCTTCGCCGCTGACAATCGCTTCT
CATACAACCACACGATCTGGAGCAACGACGCAGCAATGCAGCCAGATCAAATCAACAAAGTGGTTGCTCTCGGTG
ACAGCTTGTCTGATACAGGCAACATCTTTAACGCATCACAATGGCGCTTCCCTAACCCGAACAGCTGGTTCTTAGG
TCACTTCTCCAACGGTTTTGTGTGGACAGAATACATTGCCAAAGCGAAGAACCTTCCGCTCTACAACTGGGCAGTT
GGCGGTGCGGCTGGTGAGAACCAATACATCGCGCTAACAGGGGTTGGTGAGCAAGTTTCTTCGTACTTAACCTACG
CAAAACTGGCGAAGAACTACAAACCAGCAAACACCTTGTTTACGCTTGAGTTTGGTTTGAATGACTTCATGAACTA
CAACCGTGGAGTTCCAGAAGTGAAAGCGGATTATGCAGAAGCACTGATTCGTTTGACGGACGCAGGTGCGAAGAA
CTTCATGTTGATGACACTGCCAGACGCGACGAAAGCGCCTCAGTTTAAGTACTCAACACAAGAAGAGATCGACAA
AATTCGTGCGAAAGTGCTTGAGATGAACGAGTTCATCAAGGCACAAGCGATGTACTACAAAGCGCAAGGTTACAA
CATCACGTTGTTTGATACTCACGCCTTGTTCGAGACGCTAACTTCTGCGCCAGAAGAGCACGGTTTCGTGAACGCG
AGTGATCCTTGTTTGGACATCAACCGCTCATCGTCTGTCGATTACATGTACACCCACGCATTGCGCTCTGAGTGTGC
GGCGTCCGGTGCTGAGAAATTTGTGTTCTGGGATGTCACGCACCCAACAACAGCAACTCACCGCTATGTTGCAGAG
AAAATGCTAGAAAGTAGCAACAACTTAGCCGAGTACCGTTTCTAA

>S041

ATGATGAAAAAAACAATCACACTATTAACTGCATTACTCCCGCTTGCTTCTGCAGTTGCCGAAGAGCCAACCTTAT
CACCAGAAATGGTTTCAGCGTCTGAAGTGATCAGCACGCAAGAAAACCAAACCTATACCTATGTTCGCTGTTGGTA
TCGCACCAGCTACTCGAAAGATGATCCGGCGACCGATTGGGAATGGGCAAAAAACGAAGATGGTAGCTACTTCAC
CATTGACGGCTACTGGTGGAGCTCCGTTTCATTTAAAAACATGTTCTACACCAACACGTCGCAAAACGTTATCCGT
CAGCGTTGTGAAGCAACATTAGATTTGGCGAACGAGAACGCAGACATTACGTTCTTCGCCGCTGACAATCGCTTCT
CATACAACCACACGATCTGGAGCAACGACGCAGCAATGCAGCCAGATCAAATCAACAAAGTGGTTGCTCTCGGTG
ACAGCTTGTCTGATACAGGCAACATCTTTAACGCATCACAATGGCGCTTCCCTAACCCGAACAGCTGGTTCTTAGG
TCACTTCTCCAACGGTTTTGTGTGGACAGAATACATTGCCAAAGCGAAGAACCTTCCGCTCTACAACTGGGCAGTT
GGCGGTGCGGCTGGTGAGAACCAATACATCGCGCTAACAGGGGTTGGTGAGCAAGTTTCTTCGTACTTAACCTACG
CAAAACTGGCGAAGAACTACAAACCAGCAAACACCTTGTTTACGCTTGAGTTTGGTTTGAATGACTTCATGAACTA
CAACCGTGGAGTTCCAGAAGTGAAAGCGGATTATGCAGAAGCACTGATTCGTTTGACGGACGCAGGTGCGAAGAA
CTTCATGTTGATGACACTGCCAGACGCGACGAAAGCGCCTCAGTTTAAGTACTCAACACAAGAAGAGATCGACAA
AATTCGTGCGAAAGTGCTTGAGATGAACGAGTTCATCAAGGCACAAGCGATGTACTACAAAGCGCAAGGTTACAA
CATCACGTTGTTTGATACTCACGCCTTGTTCGAGACGCTAACTTCTGCGCCAGAAGAGCACGGTTTCGTGAACGCG
AGTGATCCTTGTTTGGACATCAACCGCTCATCGTCTGTCGATTACATGTACACCCACGCATTGCGCTCTGAGTGTGC
GGCGTCCGGTGCTGAGAAATTTGTGTTCTGGGATGTCACGCACCCAACAACAGCAACTCACCGCTATGTTGCAGAG
AAAATGCTAGAAAGTAGCAACAACTTAGCCGAGTACCGTTTCTAA

>S040
ATGATGAAAAAAACAATCACACTATTAACTGCATTACTCCCGCTTGCTTCTGCAGTTGCCGAAGAGCCAA
CCTTATCACCAGAAATGGTTTCAGCGTCTGAAGTGATCAGCACGCAAGAAAACCAAACCTATACCTATGT
TCGCTGTTGGTATCGCACCAGCTACTCGAAAGATGATCCGGCGACCGATTGGGAATGGGCAAAAAACGAA
GATGGTAGCTACTTCACCATTGACGGCTACTGGTGGAGCTCCGTTTCATTTAAAAACATGTTCTACACCA
ACACGTCGCAAAACGTTATCCGTCAGCGTTGTGAAGCAACATTAGATTTAGCGAACGAGAACGCAGACAT
TACGTTCTTCGCCGCTGACAATCGCTTCTCATACAACCACACGATCTGGAGCAACGACGCAGCAATGCAG
CCAGATCAAATCAACAAAGTGGTTGCACTCGGTGACAGCTTGTCTGATACAGGCAACATCTTTAACGCAT
CACAATGGCGCTTCCCTAACCCGAACAGCTGGTTCTTAGGTCACTTCTCCAACGGTTTTGTGTGGACAGA
ATACATTGCCAAAGCGAAGAACCTTCCGCTCTACAACTGGGCAGTTGGCGGCGCGGCTGGTGAGAACCAA
TACATCGCGCTAACAGGGGTTGGTGATCAAGTTTCTTCGTACTTAACCTACGCAAAACTGGCGAAGAACT
ACAAACCAGCAAACACCTTGTTTACGCTTGAGTTTGGTTTGAATGACTTCATGAACTACAACCGTGGCGT
TCCAGAAGTGAAAGCGGATTATGCAGAAGCACTGATTCGTTTGACGGACGCAGGTGCGAAGAACTTCATG
TTGATGACACTGCCAGACGCGACGAAAGCGCCTCAGTTTAAGTACTCAACACAAGAAGAGATCGACAAAA
TTCGTGCGAAAGTGCTTGAGATGAACGAGTTCATCAAGGCACAAGCGATGTACTACAAAGCGCAAGGTTA
CAACATCACGTTGTTTGATACTCACGCCTTGTTCGAGACGCTAACTTCTGCGCCAGAAGAGCACGGTTTC
GTGAACGCGAGTGATCCTTGTTTGGACATCAACCGCTCATCGTCTGTCGATTACATGTACACCCACGCAT
TGCGCTCTGAGTGTGCGGCGTCTGGTGCTGAGAAATTTGTGTTCTGGGATGTCACGCACCCAACAACAGC
AACTCACCGCTATGTTGCAGAGAAAATGCTAGAAAGTAGCAACAACTTAGCCGAGTACCGTTTCTAA

>S039
ATGATGAAAAAAACAATCACACTATTAACTGCATTACTCCCGCTTGCTTCTGCAGTTGCCGAAGAGCCAACCTTAT
CACCAGAAATGGTTTCAGCGTCTGAAGTGATCAGCACGCAAGAAAACCAAACCTATACCTATGTTCGCTGTTGGTA
TCGCACCAGCTACTCGAAAGATGATCCGGCGACCGATTGGGAATGGGCAAAAAACGAAGATGGTAGCTACTTCAC
CATTGACGGCTACTGGTGGAGCTCCGTTTCATTTAAAAACATGTTCTACACCAACACGTCGCAAAACGTTATCCGT
CAGCGTTGTGAAGCAACATTAGATTTGGCGAACGAGAACGCAGACATTACGTTCTTCGCCGCTGACAATCGCTTCT
CATACAACCACACGATCTGGAGCAACGACGCAGCAATGCAGCCAGATCAAATCAACAAAGTGGTTGCACTCGGTG
ACAGCTTGTCTGATACAGGCAACATCTTTAACGCATCACAATGGCGCTTCCCTAACCCGAACAGCTGGTTCTTAGG
TCACTTCTCCAACGGTTTTGTTTGGACAGAATACATTGCCAAAGCGAAGAACCTTCCGCTCTACAACTGGGCAGTT
GGCGGCGCGGCTGGTGAGAACCAATACATCGCGCTAACAGGGGTTGGTGAGCAAGTTTCTTCGTACTTAACCTACG
CAAAACTGGCGAAGAACTACAAACCAGCAAACACCTTGTTTACGCTTGAGTTTGGTTTAAATGACTTCATGAACTA
CAACCGTGGCGTTCCAGAAGTGAAAGCGGATTATGCAGAAGCACTGATTCGTTTGACGGACGCAGGTGCGAAGAA
CTTCATGTTGATGACACTGCCAGACGCGACGAAAGCGCCTCAGTTTAAGTACTCAACACAAGAAGAGATCGACAA

AATTCGTGCGAAAGTGCTTGAGATGAACGAGTTCATCAAGGCACAAGCGATGTACTACAAAGCGCAAGGTTACAA
CATCACGTTGTTTGATACTCACGCCTTGTTCGAGACGCTAACTTCTGCGCCCGAAGAGCACGGTTTCGTGAACGCG
AGTGATCCTTGTTTGGACATCAACCGCTCATCGTCTGTCGATTACATGTACACCCACGCATTGCGCTCTGAGTGTGC
GGCGTCTGGTGCTGAGAAGTTTGTGTTCTGGGATGTCACGCATCCAACAACAGCAACTCACCGCTATGTTGCAGAG
AAAATGCTAGAAAGTAGCAACAACTTAGCAGAGTACCGTTTCTAA

>S038
ATGATGAAAAAAACAATCACACTATTAACTGCATTACTCCCGCTTGCTTCTGCAGTTGCCGAAGAGCCAA
CCTTATCACCAGAAATGGTTTCAGCGTCTGAAGTGATCAGCACGCAAGAAAACCAAACCTATACCTATGT
TCGCTGTTGGTATCGCACCAGCTACTCGAAAGATGATCCGGCGACCGATTGGGAATGGGCAAAAAACGAA
GATGGTAGCTACTTCACCATTGACGGCTACTGGTGGAGCTCCGTTTCACTTAAAAACATGTTCTACACCA
ACACGTCGCAAAACGTTATCCGTCAGCGTTGTGAAGCAACATTAGATTTGGCGAACGAGAACGCAGACAT
TACGTTCTTCGCCGCTGACAATCGCTTCTCATACAACCACACGATCTGGAGCAACGACGCAGCAATGCAG
CCAGATCAAATCAACAAAGTGGTTGCACTCGGTGACAGCTTGTCTGATACAGGCAACATCTTTAACGCAT
CACAATGGCGCTTCCCTAACCCGAACAGCTGGTTCTTAGGTCACTTCTCCAACGGTTTTGTGTGGACAGA
ATACATTGCCAAAGCGAAGAACCTTCCGCTCTACAACTGGGCAGTTGGCGGCGCGGCTGGTGAGAACCAA
TACATCGCGCTAACAGGGGTTGGTGATCAAGTTTCTTCGTACTTAACCTACGCAACACTGGCGAAGAACT
ACAAACCAGCAAACACCTTGTTTACGCTTGAGTTTGGTTTGAATGACTTCATGAACTACAACCGTGGCGT
TCCAGAAGTGAAAGCAGATTATGCAGAAGCACTGATTCGTTTGACGGACGCAGGTGCGAAGAACTTCATG
TTGATGACACTGCCAGACGCGACGAAAGCGCCTCAGTTTAAGTACTCAACACAAGAAGAGATCGACAAAA
TTCGTGCGAAAGTGCTTGAGATGAACGAGTTCATCAAGGCACAAGCGATGTACTACAAAGCGCAAGGTTA
CAACATCACGTTGTTTGATACTCACGCCTTGTTCGAGACGCTAACTTCTGCGCCAGAAGAGCACGGTTTC
GTGAACGCGAGCGATCCTTGTTTGGACATCAACCGCTCATCGTCTGTCGATTACATGTACACCCACGCAT
TGCGCTCTGAGTGTGCAGCGTCTGGTGCTGAGAAGTTTGTGTTCTGGGATGTCACGCATCCAACAACAGC
AACTCACCGCTATGTTGCAGAGAAAATGCTAGAAAGTAGCAACAACTTAGCCGAGTACCGTTTCTAA

>S037
ATGATGAAAAAAACAATCACACTATTAACTGCATTACTCCCGCTTGCTTCTGCAGTTGCCGAAGAGCCAACCTTAT
CACCAGAAATGGTTTCAGCGTCTGAAGTGATCAGCACGCAAGAAAACCAAACCTATACCTATGTTCGCTGTTGGTA
TCGCACCAGCTACTCGAAAGATGATCCGGCGACCGATTGGGAATGGGCAAAAAACGAAGATGGTAGCTACTTCAC
CATTGACGGCTACTGGTGGAGCTCCGTTTCACTTAAAAACATGTTCTACACCAACACGTCGCAAAACGTTATCCGT
CAGCGTTGTGAAGCAACATTAGATTTGGCGAACGAGAACGCAGACATTACGTTCTTCGCCGCTGACAATCGCTTCT
CATACAACCACACGATCTGGAGCAACGACGCAGCAATGCAGCCAGATCAAATCAACAAAGTGGTTGCACTCGGTG
ACAGCTTGTCTGATACAGGCAACATCTTTAACGCATCACAATGGCGCTTCCCTAACCCGAACAGCTGGTTCTTAGG
TCACTTCTCCAACGGTTTTGTGTGGACAGAATACATTGCCAAAGCGAAGAACCTTCCGCTCTACAACTGGGCAGTT
GGCGGCGCGGCTGGTGAGAACCAATACATCGCGCTAACAGGGGTTGGTGATCAAGTTTCTTCGTACTTAACCTACG
CAAAACTGGCGAAGAACTACAAACCAGCAAACACCTTGTTTACGCTTGAGTTTGGTTTGAATGACTTCATGAACTA
CAACCGTGGCGTTCCAGAAGTGAAAGCAGATTATGCAGAAGCACTGATTCGTTTGACGGACGCAGGTGCGAAGAA
CTTCATGTTGATGACACTGCCAGACGCGACGAAAGCGCCTCAGTTTAAGTACTCAACACAAGAAGAGATCGACAA
AATTCGTGCGAAAGTGCTTGAGATGAACGAGTTCATCAAGGCACAAGCGATGTACTACAAAGCGCAAGGTTACAA
CATCACGTTGTTTGATACTCACGCCTTGTTCGAGACGCTAACTTCTGCGCCAGAAGAGCACGGTTTCGTGAACGCG
AGCGATCCTTGTTTGGACATCAACCGCTCATCGTCTGTCGATTACATGTACACCCACGCATTGCGCTCTGAGTGTGC
AGCGTCTGGTGCTGAGAAGTTTGTGTTCTGGGATGTCACGCATCCAACAACAGCAACTCACCGCTATGTTGCAGAG
AAAATGCTAGAAAGTAGCAACAACTTAGCCGAGTACCGTTTCTAA

>S036
ATGATGAAAAAAACAATCACACTATTAACTGCATTACTCCCGCTTGCTTCTGCAGTTGCCGAAGAGCCAA
CCTTATCACCAGAAATGGTTTCAGCGTCTGAAGTGATCAGCACGCAAGAAAACCAAACCTATACCTATGT
TCGCTGTTGGTATCGCACCAGCTACTCGAAAGATGATCCGGCGACCGATTGGGAATGGGCAAAAAACGAA
GATGGTAGCTACTTCACCATTGACGGCTACTGGTGGAGCTCCGTTTCATTTAAAAACATGTTCTACACCA
ACACGTCGCAAAACGTTATCCGTCAGCGTTGTGAAGCAACATTAGATTTGGCGAACGAGAACGCAGACAT

TACGTTCTTCGCCGCTGACAATCGCTTCTCATACAACCACACGATCTGGAGCAACGACGCAGCAATGCAG
CCAGATCAAATCAACAAAGTGGTTGCACTCGGTGACAGCTTGTCTGATACAGGCAACATCTTTAACGCAT
CACAATGGCGCTTCCCTAACCCGAACAGCTGGTTCTTAGGTCACTTCTCCAACGGTTTTGTGTGGACAGA
ATACATTGCCAAAGCGAAGAACCTTCCGCTCTACAACTGGGCAGTTGGCGGCGCGGCTGGTGAGAACCAA
TACATCGCGCTAACAGGGGTTGGTGATCAAGTTTCTTCGTACTTAACCTACGCAAAACTGGCGAAGAACT
ACAAACCAGCAAACACCTTGTTTACGCTTGAGTTTGGTTTGAATGACTTCATGAACTACAACCGTGGCGT
TCCAGAAGTGAAAGCGGATTATGCAGAAGCACTGATTCGTTTGACGGACGCAGGTGCGAAGAACTTCATG
TTGATGACACTGCCAGACGCGACGAAAGCGCCTCAGTTTAAGTACTCAACACAAGAAGAGATCGACAAAA
TTCGTGCGAAAGTGCTTGAGATGAACGAGTTCATCAAGGCACAAGCGATGTACTACAAAGCGCAAGGTTA
CAACATCACGTTGTTTGATACTCACGCCTTGTTCGAGACGCTAACTTCTGCGCCAGAAGAGCACGGTTTC
GTGAACGCGAGCGATCCTTGTTTGGACATCAACCGCTCATCGTCTGTCGATTACATGTACACCCACGCAT
TGCGCTCTGAGTGTGCTGCGTCTGGTGCTGAGAAGTTTGTGTTCTGGGATGTCACGCATCCAACAACAGC
AACTCACCGCTATGTTGCAGAGAAAATGCTAGAAAGTAGCAACAACTTAGCAGAGTACCGTTTCTAA

>S035
ATGATGAAAAAAACAATCACACTATTAACTGCATTACTCCCGCTTGCTTCTGCAGTTGCCGAAGAGCCAACCTTAT
CACCAGAAATGGTTTCAGCGTCTGAAGTGATCAGCACGCAAGAAAACCAAACCTATACCTATGTTCGCTGTTGGTA
TCGCACCAGCTACTCGAAAGATGATCCGGCGACCGATTGGGAATGGGCAAAAAACGAAGATGGTAGCTACTTCAC
CATTGACGGCTACTGGTGGAGCTCCGTTTCATTTAAAAACATGTTCTACACCAACACGTCGCAAAACGTTATCCGT
CAGCGTTGTGAAGCAACATTAGATTTGGCGAACGAGAACGCAGACATTACGTTCTTCGCCGCTGACAATCGCTTCT
CATACAACCACACGATCTGGAGCAACGACGCAGCAATGCAGCCAGATCAAATCAACAAAGTGGTTGCACTCGGTG
ACAGCTTGTCTGATACAGGCAACATCTTTAACGCATCACAATGGCGCTTCCCTAACCCGAACAGCTGGTTCTTAGG
TCACTTCTCCAACGGTTTTGTGTGGACAGAATACATTGCCAAAGCGAAGAACCTTCCGCTCTACAACTGGGCAGTT
GGCGGCGCGGCTGGTGAGAACCAATACATCGCGCTAACAGGGGTTGGTGATCAAGTTTCTTCGTACTTAACCTACG
CAAAACTGGCGAAGAACTACAAACCAGCAAACACCTTGTTTACGCTTGAGTTTGGTTTGAATGACTTCATGAACTA
CAACCGTGGCGTTCCAGAAGTGAAAGCGGATTATGCAGAAGCACTGATTCGTTTGACGGACGCAGGTGCGAAGAA
CTTCATGTTGATGACACTGCCAGACGCGACGAAAGCGCCTCAGTTTAAGTACTCAACACAAGAAGAGATCGACAA
AATTCGTGCGAAAGTGCTTGAGATGAACGAGTTCATCAAGGCACAAGCGATGTACTACAAAGCGCAAGGTTACAA
CATCACGTTGTTTGATACTCACGCCTTGTTCGAGACGCTAACTTCTGCGCCAGAAGAGCACGGTTTCGTGAACGCG
AGCGATCCTTGTTTGGACATCAACCGCTCATCGTCTGTCGATTACATGTACACCCACGCATTGCGCTCTGAGTGTGC
TGCGTCTGGTGCTGAGAAGTTTGTGTTCTGGGATGTCACGCATCCAACAACAGCAACTCACCGCTATGTTGCAGAG
AAAATGCTAGAAAGTAGCAACAACTTAGCAGAGTACCGTTTCTAA

>S034
ATGATGAAAAAAACAATCACACTATTAACTGCATTACTCCCGCTTGCTTCTGCAGTTGCCGAAGAGCCAACCTTAT
CACCAGAAATGGTTTCAGCGTCTGAAGTGATCAGCACGCAAGAAAACCAAACCTATACCTATGTTCGCTGTTGGTA
TCGCACCAGCTACTCGAAAGATGATCCGGCGACCGATTGGGAATGGGCAAAAAACGAAGATGGTAGCTACTTCAC
CATTGACGGCTACTGGTGGAGCTCCGTTTCATTTAAAAACATGTTCTACACCAACACGTCGCAAAACGTTATCCGT
CAGCGTTGTGAAGCAACATTAGATTTGGCGAACGAGAACGCAGACATTACGTTCTTCGCCGCTGACAATCGCTTCT
CATACAACCACACGATCTGGAGCAACGACGCAGCAATGCAGCCAGATCAAATCAACAAAGTGGTTGCACTCGGTG
ACAGCTTGTCTGATACAGGCAACATCTTTAACGCATCACAATGGCGCTTCCCTAACCCGAACAGCTGGTTCTTAGG
TCACTTCTCCAACGGTTTTGTGTGGACAGAATACATTGCCAAAGCGAAGAACCTTCCGCTCTACAACTGGGCAGTT
GGCGGCGCGGCTGGTGAGAACCAATACATCGCGCTAACAGGGGTTGGTGATCAAGTTTCTTCGTACTTAACCTACG
CAAAACTGGCGAAGAACTACAAACCAGCAAACACCTTGTTTACGCTTGAGTTTGGTTTGAATGACTTCATGAACTA
CAACCGTGGCGTTCCAGAAGTGAAAGCGGATTATGCAGAAGCACTGATTCGTTTGACGGACGCAGGTGCGAAGAA
CTTCATGTTGATGACACTGCCAGACGCGACGAAAGCGCCTCAGTTTAAGTACTCAACACAAGAAGAGATCGACAA
AATTCGTGCGAAAGTGCTTGAGATGAACGAGTTCATCAAGGCACAAGCGATGTACTACAAAGCGCAAGGTTACAA
CATCACGTTGTTTGATACTCACGCCTTGTTCGAGACGCTAACTTCTGCGCCAGAAGAGCACGGTTTCGTGAACGCG
AGCGATCCTTGTTTGGACATCAACCGCTCATCGTCTGTCGATTACATGTACACCCACGCATTGCGCTCTGAGTGTGC
TGCGTCTGGTGCTGAGAAGTTTGTGTTCTGGGATGTCACGCATCCAACAACAGCAACTCACCGCTATGTTGCAGAG
AAAATGCTAGAAAGTAGCAACAACTTAGCAGAGTACCGTTTCTAA

>S033
ATGATGAAAAAAACAATCACACTATTAACTGCATTACTCCCGCTTGCTTCTGCAGTTGCCGAAGAGCCAACCTTAT
CACCAGAAATGGTTTCAGCGTCTGAAGTGATCAGCACGCAAGAAAACCAAACCTATACCTATGTTCGCTGTTGGTA
TCGCACCAGCTACTCGAAAGATGATCCGGCGACCGATTGGGAATGGGCAAAAAACGAAGATGGTAGCTACTTCAC
CATTGACGGCTACTGGTGGAGCTCCGTTTCATTTAAAAACATGTTCTACACCAACACGTCGCAAAACGTTATCCGT
CAGCGTTGTGAAGCAACATTAGATTTGGCGAACGAGAACGCAGACATTACGTTCTTCGCCGCTGACAATCGCTTCT
CATACAACCACACGATCTGGAGCAACGACGCAGCAATGCAGCCAGATCAAATCAACAAAGTGGTTGCACTCGGTG
ACAGCTTGTCTGATACAGGCAACATCTTTAACGCATCACAATGGCGCTTCCCTAACCCGAACAGCTGGTTCTTAGG
TCACTTCTCCAACGGTTTTGTGTGGACAGAATACATTGCCAAAGCGAAGAACCTTCCGCTCTACAACTGGGCAGTT
GGCGGCGCGGCTGGTGAGAACCAATACATCGCGCTAACAGGGGTTGGTGATCAAGTTTCTTCGTACTTAACCTACG
CAAAACTGGCGAAGAACTACAAACCAGCAAACACCTTGTTTACGCTTGAGTTTGGTTTGAATGACTTCATGAACTA
CAACCGTGGCGTTCCAGAAGTGAAAGCGGATTATGCAGAAGCACTGATTCGTTTGACGGACGCAGGTGCGAAGAA
CTTCATGTTGATGACACTGCCAGACGCGACGAAAGCGCCTCAGTTTAAGTACTCAACACAAGAAGAGATCGACAA
AATTCGTGCGAAAGTGCTTGAGATGAACGAGTTCATCAAGGCACAAGCGATGTACTACAAAGCGCAAGGTTACAA
CATCACGTTGTTTGATACTCACGCCTTGTTCGAGACGCTAACTTCTGCGCCAGAAGAGCACGGTTTCGTGAACGCG
AGCGATCCTTGTTTGGACATCAACCGCTCATCGTCTGTCGATTACATGTACACCCACGCATTGCGCTCTGAGTGTGC
TGCGTCTGGTGCTGAGAAGTTTGTGTTCTGGGATGTCACGCATCCAACAACAGCAACTCACCGCTATGTTGCAGAG
AAAATGCTAGAAAGTAGCAACAACTTAGCAGAGTACCGTTTCTAA

>S032
ATGATGAAAAAAACAATCACACTATTAACTGCATTACTCCCGCTTGCTTCTGCAGTTGCCGAAGAGCCAACCTTAT
CACCAGAAATGGTTTCAGCGTCTGAAGTGATCAGCACGCAAGAAAACCAAACCTATACCTATGTTCGCTGTTGGTA
TCGCACCAGCTACTCGAAAGATGATCCGGCGACCGATTGGGAATGGGCAAAAAACGAAGATGGTAGCTACTTCAC
CATTGACGGCTACTGGTGGAGCTCCGTTTCATTTAAAAACATGTTCTACACCAACACGTCGCAAAACGTTATCCGT
CAGCGTTGTGAAGCAACATTAGATTTGGCGAACGAGAACGCAGACATTACGTTCTTCGCCGCTGACAATCGCTTCT
CATACAACCACACGATCTGGAGCAACGACGCAGCAATGCAGCCAGATCAAATCAACAAAGTGGTTGCACTCGGTG
ACAGCTTGTCTGATACAGGCAACATCTTTAACGCATCACAATGGCGCTTCCCTAACCCGAACAGCTGGTTCTTAGG
TCACTTCTCCAACGGTTTTGTGTGGACAGAATACATTGCCAAAGCGAAGAACCTTCCGCTCTACAACTGGGCAGTT
GGCGGCGCGGCTGGTGAGAACCAATACATCGCGCTAACAGGGGTTGGTGATCAAGTTTCTTCGTACTTAACCTACG
CAAAACTGGCGAAGAACTACAAACCAGCAAACACCTTGTTTACGCTTGAGTTTGGTTTGAATGACTTCATGAACTA
CAACCGTGGCGTTCCAGAAGTGAAAGCGGATTATGCAGAAGCACTGATTCGTTTGACGGACGCAGGTGCGAAGAA
CTTCATGTTGATGACACTGCCAGACGCGACGAAAGCGCCTCAGTTTAAGTACTCAACACAAGAAGAGATCGACAA
AATTCGTGCGAAAGTGCTTGAGATGAACGAGTTCATCAAGGCACAAGCGATGTACTACAAAGCGCAAGGTTACAA
CATCACGTTGTTTGATACTCACGCCTTGTTCGAGACGCTAACTTCTGCGCCAGAAGAGCACGGTTTCGTGAACGCG
AGCGATCCTTGTTTGGACATCAACCGCTCATCGTCTGTCGATTACATGTACACCCACGCATTGCGCTCTGAGTGTGC
TGCGTCTGGTGCTGAGAAGTTTGTGTTCTGGGATGTCACGCATCCAACAACAGCAACTCACCGCTATGTTGCAGAG
AAAATGCTAGAAAGTAGCAACAACTTAGCAGAGTACCGTTTCTAA

>S031
ATGATGAAAAAAACAATCACACTATTAACTGCATTACTCCCGCTTGCTTCTGCAGTTGCCGAAGAGCCAACCTTAT
CACCAGAAATGGTTTCAGCGTCTGAAGTGATCAGCACGCAAGAAAACCAAACCTATACCTATGTTCGCTGTTGGTA
TCGCACCAGCTACTCGAAAGATGATCCGGCGACCGATTGGGAATGGGCAAAAAACGAAGATGGTAGCTACTTCAC
CATTGACGGCTACTGGTGGAGCTCCGTTTCATTTAAAAACATGTTCTACACCAACACGTCGCAAAACGTTATCCGT
CAGCGTTGTGAAGCCACATTAGATTTGGCGAACGAGAACGCAGACATTACGTTCTTCGCCGCTGACAATCGCTTCT
CATACAACCACACGATCTGGAGCAACGACGCAGCAATGCAGCCAGATCAAATCAACAAAGTGGTTGCACTCGGTG
ACAGCTTGTCTGATACAGGCAACATCTTTAACGCATCACAATGGCGCTTCCCTAACCCGAACAGCTGGTTCTTAGG
TCACTTCTCCAACGGTTTTGTGTGGACAGAATACATTGCCAAAGCGAAGAACCTTCCGCTCTACAACTGGGCAGTT
GGCGGCGCGGCTGGTGAGAACCAATACATCGCGCTAACAGGGGTTGGTGAGCAAGTTTCTTCGTACTTAACCTACG
CAAAACTGGCGAAGAACTACAAACCAGCAAACACCTTGTTTACGCTTGAGTTTGGTTTGAATGACTTCATGAACTA
CAACCGTGGCGTTCCAGAAGTGAAAGCAGATTATGCAGAAGCACTGATTCGTTTGACGGACGCAGGTGCGAAGAA

CTTCATGTTGATGACACTGCCAGACGCGACGAAAGCGCCTCAGTTTAAGTACTCAACACAAGAAGAGATCGACAA
AATTCGTGCGAAAGTGCTTGAGATGAACGAGTTCATCAAGGCTCAAGCGATGTACTACAAAGCGCAAGGTTACAA
CATCACGTTGTTTGATACTCACGCCTTGTTCGAGACGCTAACTTCTGCGCCAGAAGAGCACGGTTTCGTGAACGCG
AGCGATCCTTGTTTGGACATCAACCGCTCATCGTCTGTCGACTACATGTACACCCACGCATTGCGCTCTGAGTGTGC
GGCGTCTGGTGCTGAGAAGTTTGTGTTCTGGGATGTCACGCACCCAACAACAGCAACTCACCGCTATGTTGCAGAG
AAAATGCTAGAAAGTAGCAACAACTTAGCCGAGTACCGTTTCTAA

>S030
ATGATGAAAAAAACAATCACACTATTAACTGCATTACTCCCGCTTGCTTCTGCAGTTGCCGAAGAGCCAA
CCTTATCACCAGAAATGGTTTCAGCGTCTGAAGTGATCAGCACGCAAGAAAACCAAACCTATACCTATGT
TCGCTGTTGGTATCGCACCAGCTACTCGAAAGATGATCCGGCGACCGATTGGGAATGGGCAAAAAACGAA
GATGGTAGCTACTTCACCATTGACGGCTACTGGTGGAGCTCCGTTTCATTTAAAAACATGTTCTACACCA
ACACGTCGCAAAACGTTATCCGTCAGCGTTGTGAAGCCACATTAGATTTGGCGAACGAGAACGCAGACAT
TACGTTCTTCGCCGCTGACAATCGCTTCTCATACAACCACACGATCTGGAGCAACGACGCAGCAATGCAG
CCAGATCAAATCAACAAAGTGGTTGCACTCGGTGACAGCTTGTCTGATACAGGCAACATCTTTAACGCAT
CACAATGGCGCTTCCCTAACCCGAACAGCTGGTTCTTAGGTCACTTCTCCAACGGTTTTGTGTGGACAGA
ATACATTGCCAAAGCGAAGAACCTTCCGCTCTACAACTGGGCAGTTGGCGGCGCGGCTGGTGAGAACCAA
TACATCGCGCTAACAGGGGTTGGTGAGCAAGTTTCTTCGTACTTAACCTACGCAAAACTGGCGAAGAACT
ACAAACCAGCAAACACCTTGTTTACGCTTGAGTTTGGTTTGAATGACTTCATGAACTACAACCGTGGCGT
TCCAGAAGTGAAAGCAGATTATGCAGAAGCACTGATTCGTTTGACGGACGCAGGTGCGAAGAACTTCATG
TTGATGACACTGCCAGACGCGACGAAAGCGCCTCAGTTTAAGTACTCAACACAAGAAGAGATCGACAAAA
TTCGTGCGAAAGTGCTTGAGATGAACGAGTTCATCAAGGCTCAAGCGATGTACTACAAAGCGCAAGGTTA
CAACATCACGTTGTTTGATACTCACGCCTTGTTCGAGACGCTAACTTCTGCGCCAGAAGAGCACGGTTTC
GTGAACGCGAGCGATCCTTGTTTGGACATCAACCGCTCATCGTCTGTCGACTACATGTACACCCACGCAT
TGCGCTCTGAGTGTGCGGCGTCTGGTGCTGAGAAGTTTGTGTTCTGGGATGTCACGCACCCAACAACAGC
AACTCACCGCTATGTTGCAGAGAAAATGCTAGAAAGTAGCAACAACTTAGCCGAGTACCGTTTCTAA

>S029
ATGATGAAAAAAACAATCACACTATTAACTGCATTACTCCCGCTTGCTTCTGCAGTTGCCGAAGAGCCAACCTTAT
CACCAGAAATGGTTTCAGCGTCTGAAGTGATCAGCACGCAAGAAAACCAAACCTATACCTATGTTCGCTGTTGGTA
TCGCACCAGCTACTCGAAAGATGATCCGGCGACCGATTGGGAATGGGCAAAAAACGAAGATGGTAGCTACTTCAC
CATTGACGGCTACTGGTGGAGCTCCGTTTCATTTAAAAACATGTTCTACACCAACACGTCGCAAAACGTTATCCGT
CAGCGTTGTGAAGCAACATTAGATTTGGCGAACGAGAACGCAGACATTACGTTCTTCGCCGCTGACAATCGCTTCT
CATACAACCACACGATCTGGAGCAACGACGCAGCAATGCAGCCAGATCAAATCAACAAAGTGGTTGCACTCGGTG
ACAGCTTGTCTGATACAGGCAACATCTTTAACGCATCACAATGGCGCTTCCCTAACCCGAACAGCTGGTTCTTAGG
TCACTTCTCCAACGGTTTTGTGTGGACAGAATACATTGCCAAAGCGAAGAACCTTCCGCTCTACAACTGGGCAGTT
GGCGGCGCGGCTGGTGAGAACCAATACATCGCGCTAACAGGGGTTGGTGATCAAGTTTCTTCGTACTTAACCTACG
CAAAACTGGCGAAGAACTACAAACCAGCAAACACCTTGTTTACGCTTGAGTTTGGTTTGAATGACTTCATGAACTA
CAACCGTGGCGTTCCAGAAGTGAAAGCGGATTATGCAGAAGCACTGATTCGCTTGACGGACGCAGGTGCGAAGAA
CTTCATGTTGATGACACTGCCAGACGCGACGAAAGCGCCTCAGTTTAAGTACTCAACACAAGAAGAGATCGACAA
AATTCGTGCGAAAGTGCTTGAGATGAACGAGTTCATCAAGGCACAAGCGATGTACTACAAAGCGCAAGGTTACAA
CATCACGTTGTTTGATACTCACGCCTTGTTCGAGACGCTAACTTCTGCGCCAGAAGAGCACGGTTTCGTGAACGCG
AGCGATCCTTGTTTGGACATCAACCGCTCATCGTCTGTCGACTACATGTACACCCACGCATTGCGCTCTGAGTGTGC
GGCGTCTGGTGCTGAGAAGTTTGTGTTCTGGGATGTCACGCACCCAACAACAGCAACTCACCGCTATGTTGCAGAG
AAAATGCTAGAAAGTAGCAACAACTTAGCCGAGTACCGTTTCTAA

>S028
ATGATGAAAAAAACAATCACACTATTAACTGCATTACTCCCGCTTGCTTCTGCAGTTGCCGAAGAGCCAA
CCTTATCACCAGAAATGGTTTCAGCGTCTGAAGTGATCAGCACGCAAGAAAACCAAACCTATACCTATGT
TCGCTGTTGGTATCGCACCAGCTACTCGAAAGATGATCCGGCGACCGATTGGGAATGGGCAAAAAACGAA
GATGGTAGCTACTTCACCATTGACGGCTACTGGTGGAGCTCCGTTTCATTTAAAAACATGTTCTACACCA

ACACGTCGCAAAACGTTATCCGTCAGCGTTGTGAAGCCACATTAGATTTGGCGAACGAGAACGCAGACAT
TACGTTCTTCGCCGCTGACAATCGCTTCTCATACAACCACACGATCTGGAGCAACGACGCAGCAATGCAG
CCAGATCAAATCAACAAAGTGGTTGCACTCGGTGACAGCTTGTCTGATACAGGCAACATCTTTAACGCAT
CACAATGGCGCTTCCCTAACCCGAACAGCTGGTTCTTAGGTCACTTCTCCAACGGTTTTGTGTGGACAGA
ATACATTGCCAAAGCGAAGAACCTTCCGCTCTACAACTGGGCAGTTGGCGGCGCGGCTGGTGAGAACCAA
TACATCGCGCTAACAGGGGTTGGTGATCAAGTTTCTTCGTACTTAACCTACGCAAAACTGGCGAAGAACT
ACAAACCAGCAAACACCTTGTTTACGCTTGAGTTTGGTTTGAATGACTTCATGAACTACAACCGTGGCGT
TCCAGAAGTGAAAGCAGATTATGCAGAAGCACTGATTCGTTTGACGGACGCAGGTGCAAAGAACTTCATG
TTGATGACACTGCCAGACGCGACGAAAGCGCCTCAGTTTAAGTACTCAACACAAGAAGAGATCGACAAAA
TTCGTGCGAAAGTGCTTGAGATGAACGAGTTCATCAAGGCACAAGCGATGTACTACAAAGCGCAAGGTTA
CAACATCACGTTGTTTGATACTCACGCCTTGTTCGAGACGCTAACTTCTGCGCCAGAAGAGCACGGTTTC
GTGAACGCGAGCGATCCTTGTTTGGACATCAACCGTTCATCGTCTGTCGATTACATGTACACCCACGCAT
TGCGCTCTGAGTGTGCGGCGTCTGGTGCTGAGAAGTTTGTGTTCTGGGATGTCACGCATCCAACAACAGC
AACTCACCGCTATGTTGCAGAGAAAATGCTAGAAAGTAGCAACAACTTAGCCGAGTACCGTTTCTAA

>S027
ATGATGAAAAAAACAATCACACTATTAACTGCATTACTCCCGCTTGCTTCTGCAGTTGCCGAAGAGCCAA
CCTTATCACCAGAAATGGTTTCAGCGTCTGAAGTGATCAGCACGCAAGAAAACCAAACCTATACCTATGT
TCGCTGTTGGTATCGCACCAGCTACTCGAAAGATGATCCGGCGACCGATTGGGAATGGGCAAAAAACGAA
GATGGTAGCTACTTCACCATTGACGGCTACTGGTGGAGCTCCGTTTCATTTAAAAACATGTTCTACACCA
ACACGTCGCAAAACGTTATCCGTCAGCGTTGTGAAGCAACATTAGATTTGGCGAACGAGAACGCAGACAT
TACGTTCTTCGCCGCTGACAATCGCTTCTCATACAACCACACGATCTGGAGCAACGACGCAGCAATGCAG
CCAGATCAAATCAACAAAGTGGTTGCACTCGGTGACAGCTTGTCTGATACAGGCAACATCTTTAACGCAT
CACAATGGCGCTTCCCTAACCCGAATAGCTGGTTCTTAGGTCACTTCTCCAACGGTTTTGTGTGGACAGA
ATACATTGCCAAAGCGAAGAACCTTCCACTCTACAACTGGGCAGTTGGCGGCGCGGCTGGTGAGAACCAA
TACATCGCGCTAACAGGGGTTGGTGATCAAGTTTCTTCGTACTTAACCTACGCAAAACTGGCGAAGAACT
ACAAACCAGCAAACACCTTGTTTACGCTTGAGTTTGGTTTGAATGACTTCATGAACTACAACCGTGGCGT
TCCAGAAGTGAAAGCGGATTATGCAGAAGCACTGATTCGTTTGACGGACGCAGGTGCGAAGAACTTCATG
TTGATGACACTGCCAGATGCGACGAAAGCGCCTCAGTTTAAGTACTCAACACAAGAAGAGATCGACAAAA
TTCGTGCGAAAGTGCTTGAGATGAACGAGTTCATCAAGGCACAAGCGATGTACTACAAAGCGCAAGGTTA
CAACATCACGTTGTTTGATACTCACGCCTTGTTCGAGACGCTAACTTCTGCGCCAGAAGAGCACGGTTTC
GTGAACGCGAGTGATCCTTGTTTGGACATCAACCGCTCATCGTCTGTCGATTACATGTACACCCACGCAT
TGCGCTCTGAGTGTGCGGCGTCCGGTGCTGAGAAATTTGTGTTCTGGGATGTCACGCACCCAACAACAGC
AACTCACCGCTATGTTGCAGAGAAAATGCTAGAAAGTAGCAACAACTTAGCAGAGTACCGTTTCTAA

>S026
ATGATGAAAAAAACAATCACACTATTAACTGCATTACTCCCGCTTGCTTCTGCAGTTGCCGAAGAGCCAACCTTAT
CACCAGAAATGGTTTCAGCGTCTGAAGTGATCAGCACGCAAGAAAACCAAACCTATACCTATGTTCGCTGTTGGTA
TCGCACCAGCTACTCGAAAGATGATCCGGCGACCGATTGGGAATGGGCAAAAAACGAAGATGGTAGCTACTTCAC
CATTGACGGCTACTGGTGGAGCTCCGTTTCATTTAAAAACATGTTCTACACCAACACGTCGCAAAACGTTATCCGT
CAGCGTTGTGAAGCAACATTAGATTTGGCGAACGAGAACGCAGACATTACGTTCTTCGCCGCTGACAATCGCTTCT
CATACAACCACACGATCTGGAGCAACGACGCAGCAATGCAGCCAGATCAAATCAACAAAGTGGTTGCACTCGGTG
ACAGCTTGTCTGATACAGGCAACATCTTTAACGCATCACAATGGCGCTTCCCTAACCCGAATAGCTGGTTCTTAGG
TCACTTCTCCAACGGTTTTGTGTGGACAGAATACATTGCCAAAGCGAAGAACCTTCCACTCTACAACTGGGCAGTT
GGCGGCGCGGCTGGTGAGAACCAATACATCGCGCTAACAGGGGTTGGTGATCAAGTTTCTTCGTACTTAACCTACG
CAAAACTGGCGAAGAACTACAAACCAGCAAACACCTTGTTTACGCTTGAGTTTGGTTTGAATGACTTCATGAACTA
CAACCGTGGCGTTCCAGAAGTGAAAGCGGATTATGCAGAAGCACTGATTCGTTTGACGGACGCAGGTGCGAAGAA
CTTCATGTTGATGACACTGCCAGATGCGACGAAAGCGCCTCAGTTTAAGTACTCAACACAAGAAGAGATCGACAA
AATTCGTGCGAAAGTGCTTGAGATGAACGAGTTCATCAAGGCACAAGCGATGTACTACAAAGCGCAAGGTTACAA
CATCACGTTGTTTGATACTCACGCCTTGTTCGAGACGCTAACTTCTGCGCCAGAAGAGCACGGTTTCGTGAACGCG
AGTGATCCTTGTTTGGACATCAACCGCTCATCGTCTGTCGATTACATGTACACCCACGCATTGCGCTCTGAGTGTGC

GGCGTCCGGTGCTGAGAAATTTGTGTTCTGGGATGTCACGCACCCAACAACAGCAACTCACCGCTATGTTGCAGAG
AAAATGCTAGAAAGTAGCAACAACTTAGCAGAGTACCGTTTCTAA

>S025
ATGATGAAAAAAACAATCACACTATTAACTGCATTACTCCCGCTTGCTTCTGCAGTTGCCGAAGAGCCAACCTTAT
CACCAGAAATGGTTTCAGCGTCGGAAGTGATCAGCACGCAAGAAAACCAAACCTATACCTATGTTCGCTGTTGGTA
TCGCACCAGCTACTCGAAAGATGATCCGGCGACCGATTGGGAATGGGCAAAAAACGAAGATGGTAGCTACTTCAC
CATTGACGGTTACTGGTGGAGCTCCGTTTCATTTAAAAACATGTTCTACACCAACACGTCGCAAAACGTTATCCGT
CAGCGTTGTGAAGCCACATTAGATTTGGCGAACGAGAACGCAGACATTACGTTCTTCGCCGCTGACAATCGCTTCT
CATACAACCACACGATCTGGAGCAACGACGCAGCAATGCAGCCAGATCAAATCAACAAAGTGGTTGCACTCGGTG
ACAGCTTGTCTGATACAGGCAACATCTTTAACGCATCACAATGGCGCTTCCCTAACCCGAACAGCTGGTTCTTAGG
TCACTTCTCCAACGGTTTTGTGTGGACAGAATACATTGCCAAAGCGAAGAACCTTCCGCTCTACAACTGGGCAGTT
GGCGGCGCGGCTGGTGAGAACCAATACATCGCGCTAACAGGGGTTGGTGAGCAAGTTTCTTCGTACTTAACCTATG
CAAAACTGGCGAAGAACTACAAACCAGCAAACACCTTGTTTACGCTTGAGTTTGGTTTGAATGACTTCATGAACTA
CAACCGTGGCGTTCCAGAAGTGAAAGCAGATTATGCAGAAGCACTGATTCGTTTGACGGACGCAGGTGCGAAGAA
CTTCATGTTGATGACACTGCCAGACGCGACGAAAGCGCCTCAGTTTAAGTACTCAACACAAGAAGAGATCGACAA
AATTCGTGCGAAAGTGCTTGAGATGAACGAGTTCATCAAGGCACAAGCGATGTACTACAAAGCGCAAGGTTACAA
CATCACGTTGTTTGATACTCACGCCTTGTTCGAGACGCTAACTTCTGCGCCCGAAGAGCACGGTTTCGTGAACGCG
AGTGATCCTTGTTTGGACATCAACCGCTCATCGTCTGTCGATTACATGTACACCCACGCATTGCGCTCTGAGTGTGC
AGCGTCTGGTGCTGAGAAGTTTGTGTTCTGGGATGTCACGCATCCAACAACAGCAACTCACCGCTATGTTGCAGAG
AAAATGCTAGAAAGTAGCAACAACTTAGCCGAGTACCGTTTCTAA

>S024
ATGATGAAAAAAACAATCACACTATTAACTGCATTACTCCCGCTTGCTTCTGCAGTTGCCGAAGAGCCAACCTTAT
CACCAGAAATGGTTTCAGCGTCTGAAGTGATCAGCACGCAAGAAAACCAAACCTATACCTATGTTCGCTGTTGGTA
TCGCACCAGCTACTCGAAAGATGATCCGGCGACCGATTGGGAATGGGCAAAAAACGAAGATGGTAGCTACTTCAC
CATTGACGGCTACTGGTGGAGCTCCGTTTCATTTAAAAACATGTTCTACACCAACACGTCGCAAAACGTTATCCGT
CAGCGTTGTGAAGCAACATTAGATTTGGCGAACGAGAACGCAGACATTACGTTCTTCGCCGCTGACAATCGCTTCT
CATACAACCACACGATCTGGAGCAACGACGCAGCAATGCAGCCAGATCAAATCAACAAAGTGGTTGCACTCGGTG
ACAGCTTGTCTGATACAGGCAACATCTTTAACGCATCACAATGGCGCTTCCCTAACCCGAACAGCTGGTTCTTAGG
TCACTTCTCCAACGGTTTTGTGTGGACAGAATACATTGCCAAAGCGAAGAACCTTCCGCTCTACAACTGGGCAGTT
GGCGGCGCGGCTGGTGAGAACCAATATATCGCGCTAACAGGGGTTGGTGATCAAGTTTCTTCGTACTTAACCTACG
CAAAACTGGCGAAGAACTACAAACCAGCAAACACCTTGTTTACGCTTGAGTTTGGTTTGAATGACTTCATGAACTA
CAACCGTGGCGTTCCAGAAGTGAAAGCAGATTATGCAGAAGCACTGATTCGTTTGACGGACGCAGGTGCGAAGAA
CTTCATGTTGATGACACTGCCAGACGCGACGAAAGCGCCTCAGTTTAAGTACTCAACACAAGAAGAGATCGACAA
AATTCGTGCGAAAGTGCTTGAGATGAACGAGTTCATCAAGGCACAAGCGATGTACTACAAAGCGCAAGGTTACAA
CATCACGTTGTTTGATACTCACGCCTTGTTCGAGACGCTAACTTCTGCGCCCGAAGAGCACGGTTTCGTGAACGCG
AGTGATCCTTGTTTGGACATCAACCGCTCATCGTCTATCGATTACATGTACACCCACGCATTGCGCTCTGAGTGTGC
GGCGTCTGGTGCTGAGAAGTTTGTGTTCTGGGATGTCACGCACCCAACAACAGCAACTCACCGCTATGTTGCAGAG
AAAATGCTAGAAAGTAGCAACAACTTAGCCGAGTACCGTTTCTAA

>S023
ATGATGAAAAAAACAATCACACTATTAACTGCATTACTCCCGCTTGCTTCTGCAGTTGCCGAAGAGCCAACCTTAT
CACCAGAAATGGTTTCAGCGTCTGAAGTGATCAGCACGCAAGAAAACCAAACCTATACCTATGTTCGCTGTTGGTA
TCGCACCAGCTACTCGAAAGATGATCCGGCGACCGATTGGGAATGGGCAAAAAACGAAGATGGTAGCTACTTCAC
CATTGACGGCTACTGGTGGAGCTCCGTTTCATTTAAAAACATGTTCTACACCAACACGTCGCAAAACGTTATCCGT
CAGCGTTGTGAAGCAACATTAGATTTGGCGAACGAGAACGCAGACATTACGTTCTTCGCCGCTGACAATCGCTTCT
CATACAACCACACGATCTGGAGCAACGATGCAGCAATGCAGCCAGATCAAATCAACAAAGTGGTTGCACTCGGTG
ACAGCTTGTCTGATACAGGCAACATCTTTAACGCATCACAATGGCGCTTCCCTAACCCGAACAGCTGGTTCTTAGG
TCACTTCTCCAACGGTTTTGTGTGGACAGAATACATTGCCAAAGCGAAGAACCTTCCGCTCTACAACTGGGCAGTT
GGCGGCGCGGCTGGTGAGAACCAATACATCGCGCTAACAGGGGTTGGTGATCAAGTTTCTTCGTACTTAACCTACG

CAAAACTGGCGAAGAACTACAAACCAGCAAACACCTTGTTTACGCTTGAGTTTGGTTTGAATGACTTCATGAACTA
CAACCGTGGCGTTCCAGAAGTGAAAGCGGATTATGCAGAAGCACTGATTCGTTTGACGGACGCAGGTGCGAAGAA
CTTCATGTTGATGACACTGCCAGACGCGACGAAAGCGCCTCAGTTTAAGTACTCAACACAAGAAGAGATCGACAA
AATTCGTGCGAAAGTGCTTGAGATGAACGAGTTCATCAAGGCACAAGCGATGTACTACAAAGCGCAAGGTTACAA
CATCACGTTGTTTGATACTCACGCCTTGTTCGAGACGCTAACTTCTGCGCCAGAAGAGCACGGTTTCGTGAACGCG
AGCGATCCTTGTTTGGACATCAACCGCTCATCGTCTGTCGATTACATGTACACCCACGCATTGCGCTCTGAGTGTGC
AGCGTCTGGTGCTGAGAAGTTTGTATTCTGGGATGTCACGCACCCAACAACAGCAACTCACCGCTATGTTGCAGAG
AAAATGCTAGAAAGTAGCAACAACTTAGCCGAGTACCGTTTCTAA

>S022
ATGATGAAAAAAACAATCACACTATTAACTGCATTACTCCCGCTTGCTTCTGCAGTTGCCGAAGAGCCAACCTTAT
CACCAGAAATGGTTTCAGCGTCTGAAGTGATCAGCACGCAAGAAAACCAAACCTATACCTATGTTCGCTGTTGGTA
TCGCACCAGCTACTCGAAAGATGATCCGGCGACCGATTGGGAATGGGCAAAAAACGAAGATGGTAGCTACTTCAC
CATTGACGGCTACTGGTGGAGCTCCGTTTCATTTAAAAACATGTTCTACACCAACACGTCGCAAAACGTTATCCGT
CAGCGTTGTGAAGCAACATTAGATTTGGCGAACGAGAACGCAGACATTACGTTCTTCGCCGCTGACAATCGCTTCT
CATACAACCACACGATCTGGAGCAACGACGCAGCAATGCAGCCAGATCAAATCAACAAAGTGGTTGCACTCGGTG
ACAGCTTGTCTGATACAGGCAACATCTTTAACGCATCACAATGGCGCTTCCCTAACCCGAACAGCTGGTTCTTAGG
TCACTTCTCCAACGGTTTTGTGTGGACAGAATACATTGCCAAAGCGAAGAACCTTCCGCTCTACAACTGGGCAGTT
GGCGGCGCGGCTGGTGAGAACCAATACATCGCGCTAACAGGGGTTGGTGATCAAGTTTCTTCGTACTTAACCTACG
CAAAACTGGCGAAGAACTACAAACCAGCAAACACCTTGTTTACGCTTGAGTTTGGTTTGAATGACTTCATGAACTA
CAACCGTGGCGTTCCAGAAGTGAAAGCGGATTATGCAGAAGCACTGATTCGTTTGACGGACGCAGGTGCGAAGAA
CTTCATGTTGATGACACTGCCAGACGCGACGAAAGCGCCTCAGTTTAAGTACTCAACACAAGAAGAGATCGACAA
AATTCGTGCGAAAGTGCTTGAGATGAACGAGTTCATCAAGGCACAAGCGATGTACTACAAAGCGCAAGGTTACAA
CATCACGTTGTTTGATACTCACGCCTTGTTCGAGACGCTAACTTCTGCGCCAGAAGAGCACGGTTTCGTGAACGCG
AGCGATCCTTGTTTGGACATCAACCGCTCATCGTCTGTCGATTACATGTACACCCACGCATTGCGCTCTGAGTGTGC
TGCGTCTGGTGCTGAGAAGTTTGTGTTCTGGGATGTCACGCATCCAACAACAGCAACTCACCGCTATGTTGCAGAG
AAAATGCTAGAAAGTAGCAACAACTTAGCAGAGTACCGTTTCTAA

>S021
ATGATGAAAAAAACAATCACACTATTAACTGCATTACTCCCGCTTGCTTCTGCAGTTGCCGAAGAGCCAA
CCTTATCACCAGAAATGGTTTCAGTGTCTGAAGTGATCAGCACGCAAGAAAACCAAACCTATACCTATGT
TCGCTGTTGGTATCGCACCAGCTACTCGAAAGATGATCCGGCGACCGATTGGGAATGGGCAAAAAACGAA
GATGGTAGCTACTTCACCATTGACGGCTACTGGTGGAGCTCCGTTTCATTTAAAAACATGTTCTACACCA
ACACGTCGCAAAACGTTATCCGTCAGCGTTGTGAAGCAACATTAGATTTGGCGAACGAGAACGCAGACAT
TACGTTCTTCGCCGCTGACAATCGCTTCTCATACAACCACACGATCTGGAGCAACGACGCAGCAATGCAG
CCAGATCAAATCAACAAAGTGGTTGCACTCGGTGACAGCTTGTCTGATACAGGCAACATCTTTAACGCAT
CACAATGGCGCTTCCCTAACCCGAACAGCTGGTTCTTAGGTCACTTCTCCAACGGTTTTGTGTGGACAGA
ATACATTGCCAAAGCGAAGAACCTTCCGCTCTACAACTGGGCAGTTGGCGGCGCGGCTGGTGAGAACCAA
TACATCGCGCTAACAGGGGTTGGTGAGCAAGTTTCTTCGTACTTAACCTACGCAAAACTGGCGAAGAACT
ACAAACCAGCAAACACCTTGTTTACGCTTGAGTTTGGTTTGAATGACTTCATGAACTACAACCGTGGCGT
TCCAGAAGTGAAAGCGGATTATGCAGAAGCACTGATTCGTTTGACGGACGCAGGTGCGAAGAACTTCATG
TTGATGACACTGCCAGATGCGACGAAAGCGCCTCAGTTTAAGTACTCAACACAAGAAGAGATCGACAAAA
TTCGTGCGAAAGTGCTTGAGATGAACGAGTTCATCAAGGCACAAGCGATGTACTACAAAGCGCAAGGTTA
CAACATCACGTTGTTTGATACTCACGCCTTGTTCGAGACGCTAACTTCTGCGCCAGAAGAGCACGGTTTC
GTGAACGCGAGCGATCCTTGTTTGGACATCAACCGCTCATCGTCTGTCGATTACATGTACACCCACGCAT
TGCGCTCTGAGTGTGCAGCGTCTGGTGCTGAGAAGTTTGTGTTCTGGGATGTCACGCATCCAACAACAGC
AACTCACCGCTATGTTGCAGAGAAAATGCTAGAAAGTAGCAACAACTTAGCCGAGTACCGTTTCTAA

>S020
ATGATGAAAAAAACAATCACACTATTAACTGCATTACTCCCGCTTGCTTCTGCAGTTGCCGAAGAGCCAA
CCTTATCACCAGAAATGGTTTCAGCGTCTGAAGTGATCAGCACGCAAGAAAACCAAACCTATACCTATGT

TCGCTGTTGGTATCGCACCAGCTACTCGAAAGATGATCCGGCGACCGATTGGGAATGGGCAAAAAACGAA
GATGGTAGCTACTTCACCATTGACGGCTACTGGTGGAGCTCCGTTTCATTTAAAAACATGTTCTACACCA
ACACGTCGCAAAACGTTATCCGTCAGCGTTGTGAAGCAACATTAGATTTGGCGAACGAGAACGCAGACAT
TACGTTCTTCGCCGCTGACAATCGCTTCTCATACAACCACACGATCTGGAGCAACGACGCAGCAATGCAG
CCAGATCAAATCAACAAAGTGGTTGCACTCGGTGACAGCTTGTCTGATACAGGCAACATCTTTAACGCAT
CACAATGGCGCTTCCCTAACCCGAACAGCTGGTTCTTAGGTCACTTCTCCAACGGTTTTGTGTGGACAGA
ATACATTGCCAAAGCGAAGAACCTTCCGCTCTACAACTGGGCAGTTGGCGGCGCGGCTGGTGAGAACCAA
TACATCGCGCTAACAGGGGTTGGTGAGCAAGTTTCTTCGTACTTAACCTACGCAAAACTGGCGAAGAACT
ACAAACCAGCAAACACCTTGTTTACGCTTGAGTTTGGTTTGAATGACTTCATGAACTACAACCGTGGCGT
TCCAGAAGTGAAAGCGGATTATGCAGAAGCACTGATTCGTTTGACGGACGCAGGTGCGAAGAACTTCATG
TTGATGACACTGCCAGATGCGACGAAAGCGCCTCAGTTTAAGTACTCAACACAAGAAGAGATCGACAAAA
TTCGTGCGAAAGTGCTTGAGATGAACGAGTTCATCAAGGCACAAGCGATGTACTACAAAGCGCAAGGTTA
CAACATCACGTTGTTTGATACTCACGCCTTGTTCGAGACGCTAACTTCTGCGCCAGAAGAGCACGGTTTC
GTGAACGCGAGCGATCCTTGTTTGGACATCAACCGCTCATCGTCTGTCGATTACATGTACACCCACGCAT
TGCGCTCTGAGTGTGCAGCGTCTGGTGCTGAGAAGTTTGTGTTCTGGGATGTCACGCATCCAACAACAGC
AACTCACCGCTATGTTGCAGAGAAAATGCTAGAAAGTAGCAACAACTTAGCCGAGTACCGTTTCTAA

>S019
ATGATGAAAAAAACAATCACACTATTAACTGCATTACTCCCGCTTGCTTCTGCAGTTGCCGAAGAGCCAA
CCTTATCACCAGAAATGGTTTCAGCGTCTGAAGTGATCAGCACGCAAGAAAACCAAACCTATACCTATGT
TCGCTGTTGGTATCGCACCAGCTACTCGAAAGATGATCCGGCGACCGATTGGGAATGGGCAAAAAACGAA
GATGGTAGCTACTTCACCATTGACGGCTACTGGTGGAGCTCCGTTTCATTTAAAAACATGTTCTACACCA
ACACGTCGCAAAACGCTATCCGTCAGCGTTGTGAAGCAACATTAGATTTGGCGAACGAGAACGCAGACAT
TACGTTCTTCGCCGCTGACAATCGCTTCTCATACAACCACACGATCTGGAGCAACGACGCAGCAATGCAG
CCAGATCAAATCAACAAAGTGGTTGCACTCGGTGACAGCTTGTCTGATACAGGCAACATCTTTAACGCAT
CACAATGGCGCTTCCCTAACCCGAACAGCTGGTTCTTAGGTCACTTCTCCAACGGTTTTGTGTGGACAGA
ATACATTGCCAAAGCGAAGAACCTTCCGCTCTACAACTGGGCAGTTGGCGGCGCGGCTGGTGAGAACCAA
TACATCGCGCTAACAGGGGTTGGTGATCAAGTTTCTTCGTACTTAACCTACGCAAAACTGGCGAAGAACT
ACAAACCAGCAAACACCTTGTTTACGCTTGAGTTTGGTTTGAATGACTTCATGAACTACAACCGTGGCGT
TCCAGAAGTGAAAGCGGATTATGCAGAAGCACTGATTCGTTTGACGGACGCAGGTGCGAAGAACTTCATG
TTGATGACACTGCCAGATGCGACGAAAGCGCCTCAGTTTAAGTACTCAACACAAGAAGAGATCGACAAAA
TTCGTGCGAAAGTGCTTGAGATGAACGAGTTCATCAAGGCACAAGCGATGTACTACAAAGCGCAAGGTTA
CAACATCACGTTGTTTGATACTCACGCCTTGTTCGAGACGCTAACTTCTGCGCCCGAAGAGCACGGTTTC
GTGAACGCGAGTGATCCTTGTTTGGACATCAACCGCTCATCGGCTGTCGATTACATGTACACCCACGCAT
TGCGCTCTGAGTGTGCGGCGTCTGGTGCTGAGAAATTTGTGTTCTGGGATGTCACGCACCCAACAACAGC
AACTCACCGCTATGTTGCAGAGAAAATGCTAGAAAGTAGCAACAACTTAGCCGAGTACCGTTTCTAA

>S018
ATGATGAAAAAAACAATCACACTATTAACTGCATTACTCCCGCTTGCTTCTGCAGTTGCCGAAGAGCCAACCTTAT
CACCAGAAATGGTTTCAGCGTCTGAAGTGATCAGCACGCAAGAAAACCAAACCTATACCTATGTTCGCTGTTGGTA
TCGCACCAGCTACTCGAAAGATGATCCGGCGACCGATTGGGAATGGGCAAAAAACGAAGATGGTAGCTACTTCAC
CATTGACGGCTACTGGTGGAGCTCCGTTTCATTTAAAAACATGTTCTACACCAACACGTCGCAAAACGTTATCCGT
CAGCGTTGTGAAGCAACATTAGATTTGGCGAACGAGAACGCAGACATTACGTTCTTCGCCGCTGACAATCGCTTCT
CATACAACCACACGATCTGGAGCAACGACGCAGCAATGCAGCCAGATCAAATCAACAAAGTGGTTGCACTCGGTG
ACAGCTTGTCTGATACAGGCAACATCTTTAACGCATCACAATGGCGCTTCCCTAACCCGAACAGCTGGTTCTTAGG
TCACTTCTCCAACGGTTTTGTGTGGACAGAATACATTGCCAAAGCGAAGAACCTTCCGCTCTACAACTGGGCAGTT
GGCGGCGCGGCTGGTGAGAACCAATACATCGCGCTAACAGGGGTTGGTGATCAAGTTTCTTCGTACTTAACCTACG
CAAAACTGGCGAAGAACTACAAACCAGCAAACACCTTGTTTACGCTTGAGTTTGGTTTGAATGACTTCATGAACTA
CAACCGTGGCGTTCCAGAAGTGAAAGCGGATTATGCAGAAGCACTGATTCGTTTGACGGACGCAGGTGCGAAGAA
CTTCATGTTGATGACACTGCCAGACGCGACGAAAGCGCCTCAGTTTAAGTACTCAACACAAGAAGAGATCGACAA
AATTCGTGCGAAAGTGCTTGAGATGAACGAGTTCATCAAGGCACAAGCGATGTACTACAAAGCGCAAGGTTACAA

CATCACGTTGTTTGATACTCACGCCTTGTTCGAGACGCTAACTTCTGCGCCCGAAGAGCACGGTTTCGTGAACGCG
AGTGATCCTTGTTTGGACATCAACCGCTCATCGTCTGTCGATTACATGTACACCCACGCATTGCGCTCTGAGTGTGC
GGCGTCTGGTGCTGAGAAGTTTGTGTTCTGGGATGTCACGCACCCAACAACAGCAACTCACCGCTATGTTGCAGAG
AAAATGCTAGAAAGTAGCAACAACTTAGCCGAGTACCGTTTCTAA

>S017
ATGATGAAAAAAACAATCACACTATTAACTGCATTACTCCCGCTTGCTTCTGCAGTTGCCGAAGAGCCAACCTTAT
CACCAGAAATGGTTTCAGCGTCGGAAGTGATCAGCACGCAAGAAAACCAAACCTATACCTATGTTCGCTGTTGGTA
TCGCACCAGCTACTCGAAAGATGATCCGGCGACCGATTGGGAATGGGCAAAAAACGAAGATGGTAGCTACTTCAC
CATTGACGGTTACTGGTGGAGCTCCGTTTCATTTAAAAACATGTTCTACACCAACACGTCGCAAAACGTTATCCGT
CAGCGTTGTGAAGCCACATTAGATTTGGCGAACGAGAACGCAGACATTACGTTCTTCGCCGCTGACAATCGCTTCT
CATACAACCACACGATCTGGAGCAACGACGCAGCAATGCAGCCAGATCAAATCAACAAAGTGGTTGCACTCGGTG
ACAGCTTGTCTGATACAGGCAACATCTTTAACGCATCACAATGGCGCTTCCCTAACCCGAACAGCTGGTTCTTAGG
TCACTTCTCCAACGGTTTTGTGTGGACAGAATACATTGCCAAAGCGAAGAACCTTCCGCTCTACAACTGGGCAGTT
GGCGGCGCGGCTGGTGAGAACCAATACATCGCGCTAACAGGGGTTGGTGAGCAAGTTTCTTCGTACTTAACCTATG
CAAAACTGGCGAAGAACTACAAACCAGCAAACACCTTGTTTACGCTTGAGTTTGGTTTGAATGACTTCATGAACTA
CAACCGTGGCGTTCCAGAAGTGAAAGCAGATTATGCAGAAGCACTGATTCGTTTGACGGACGCAGGTGCGAAGAA
CTTCATGTTGATGACACTGCCAGACGCGACGAAAGCGCCTCAGTTTAAGTACTCAACACAAGAAGAGATCGACAA
AATTCGTGCGAAAGTGCTTGAGATGAACGAGTTCATCAAGGCACAAGCGATGTACTACAAAGCGCAAGGTTACAA
CATCACGTTGTTTGATACTCACGCCTTGTTCGAGACGCTAACTTCTGCGCCCGAAGAGCACGGTTTCGTGAACGCG
AGTGATCCTTGTTTGGACATCAACCGCTCATCGTCTGTCGATTACATGTACACCCACGCATTGCGCTCTGAGTGTGC
AGCGTCTGGTGCTGAGAAGTTTGTGTTCTGGGATGTCACGCATCCAACAACAGCAACTCACCGCTATGTTGCAGAG
AAAATGCTAGAAAGTAGCAACAACTTAGCCGAGTACCGTTTCTAA

>S016
ATGATGAAAAAAACAATCACACTATTAACTGCATTACTCCCGCTTGCTTCTGCAGTTGCCGAAGAGCCAACCTTAT
CACCAGAAATGGTTTCAGCGTCTGAAGTGATCAGCACGCAAGAAAACCAAACCTATACCTATGTTCGCTGTTGGTA
TCGCACCAGCTACTCGAAAGATGATCCGGCGACCGATTGGGAATGGGCAAAAAACGAAGATGGTAGCTACTTCAC
CATTGACGGCTACTGGTGGAGCTCCGTTTCATTTAAAAACATGTTCTACACCAACACGTCGCAAAACGTTATCCGT
CAGCGTTGTGAAGCAACATTAGATTTGGCGAACGAGAACGCAGACATTACGTTCTTCGCCGCTGACAATCGCTTCT
CATACAACCACACGATCTGGAGCAACGACGCAGCAATGCAGCCAGATCAAATCAACAAAGTGGTTGCACTCGGTG
ACAGCTTGTCTGATACAGGCAACATCTTTAACGCATCACAATGGCGCTTCCCTAACCCGAACAGCTGGTTCTTAGG
TCACTTCTCCAACGGTTTTGTGTGGACAGAATACATTGCCAAAGCGAAGAACCTTCCGCTCTACAACTGGGCAGTT
GGCGGCGCGGCTGGTGAGAACCAATACATCGCGCTAACAGGGGTTGGTGATCAAGTTTCTTCGTACTTAACCTACG
CAAAACTGGCGAAGAACTACAAACCAGCAAACACCTTGTTTACGCTTGAGTTTGGTTTGAATGACTTCATGAACTA
CAACCGTGGCGTTCCAGAAGTGAAAGCGGATTATGCAGAAGCACTGATTCGTTTGACGGACGCAGGTGCGAAGAA
CTTCATGTTGATGACACTGCCAGACGCGACGAAAGCGCCTCAGTTTAAGTACTCAACACAAGAAGAGATCGACAA
AATTCGTGCGAAAGTGCTTGAGATGAACGAGTTCATCAAGGCACAAGCGATGTACTACAAAGCGCAAGGTTACAA
CATCACGTTGTTTGATACTCACGCCTTGTTCGAGACGCTAACTTCTGCGCCCGAAGAGCACGGTTTCGTGAACGCG
AGTGATCCTTGTTTGGACATCAACCGCTCATCGTCTGTCGATTACATGTACACCCACGCATTGCGCTCTGAGTGTGC
GGCGTCTGGTGCTGAGAAGTTTGTGTTCTGGGATGTCACGCACCCAACAACAGCAACTCACCGCTATGTTGCAGAG
AAAATGCTAGAAAGTAGCAACAACTTAGCCGAGTACCGTTTCTAA

>S015
ATGATGAAAAAAACAATCACACTATTAACTGCATTACTCCCGCTTGCTTCTGCAGTTGCCGAAGAGCCAA
CCTTATCACCAGAAATGGTTTCAGCGTCTGAAGTGATCAGCACGCAAGAAAACCAAACCTATACCTATGT
TCGCTGTTGGTATCGCACCAGCTACTCGAAAGATGATCCGGCGACCGATTGGGAATGGGCAAAAAACGAA
GATGGTAGCTACTTCACCATTGACGGCTACTGGTGGAGCTCCGTTTCATTTAAAAACATGTTCTACACCA
ACACGTCGCAAAACGTTATCCGTCAGCGTTGTGAAGCAACATTAGATTTGGCGAACGAGAACGCAGACAT
TACGTTCTTCGCCGCTGACAATCGCTTCTCATACAACCACACGATCTGGAGCAACGACGCAGCAATGCAG

CCAGATCAAATCAACAAAGTGGTTGCACTCGGTGACAGCTTGTCTGATACAGGCAACATCTTTAACGCAT
CACAATGGCGCTTCCCTAACCCGAACAGCTGGTTCTTAGGTCACTTCTCCAACGGTTTTGTGTGGACAGA
ATACATTGCCAAAGCGAAGAACCTTCCGCTCTACAACTGGGCAGTTGGCGGCGCGGCTGGTGAGAATCAA
TACATCGCGCTAACAGGGGTTGGTGATCAAGTTTCTTCGTACTTAACCTACGCAAAACTGGCGAAGAACT
ACAAACCAGCAAACACCTTGTTTACGCTTGAGTTTGGTTTGAATGACTTCATGAACTACAACCGTGGCGT
TCCAGAAGTGAAAGCAGATTATGCAGAAGCACTGATTCGTTTGACGGACGCAGGTGCGAAGAACTTCATG
TTGATGACACTGCCAGATGCGACGAAAGCGCCTCAGTTTAAGTACTCAACACAAGAAGAGATCGACAAAA
TTCGTGCGAAAGTGCTTGAGATGAACGAGTTCATCAAGGCACAAGCGATGTACTACAAAGCGCAAGGTTA
CAACATCACGTTGTTTGATACTCACGCCTTGTTCGAGACGCTAACTTCTGCGCCAGAAGAGCACGGTTTC
GTGAACGCGAGTGATCCTTGTTTGGACATCAACCGCTCATCGTCTGTCGATTACATGTACACCCACGCAT
TGCGCTCTGAGTGTGCGGCGTCTGGTGCTGAGAAGTTTGTGTTCTGGGATGTCACGCATCCAACAACAGC
AACTCACCGCTATGTTGCAGAGAAAATGCTAGAAAGTAGCAACAACTTAGCCGAGTACCGTTTCTAA

>S014
ATGATGAAAAAAACAATCACACTATTAACTGCATTACTCCCGCTTGCTTCTGCAGTTGCCGAAGAGCCAA
CCTTATCACCAGAAATGGTTTCAGCGTCTGAAGTGATCAGCACGCAAGAAAACCAAACCTATACCTATGT
TCGCTGTTGGTATCGCACCAGCTACTCGAAAGATGATCCGGCGACCGATTGGGAATGGGCAAAAAACGAA
GATGGTAGCTACTTCACCATTGACGGCTACTGGTGGAGCTCCGTTTCATTTAAAAACATGTTCTACACCA
ACACGTCGCAAAACGTTATCCGTCAGCGTTGTGAAGCAACATTAGATTTGGCGAACGAGAACGCAGACAT
TACGTTCTTCGCCGCTGACAATCGCTTCTCATACAACCACACGATCTGGAGCAACGACGCAGCAATGCAG
CCAGATCAAATCAACAAAGTGGTTGCACTCGGTGACAGCTTGTCTGATACAGGCAACATCTTTAACGCAT
CACAATGGCGCTTCCCTAACCCGAACAGCTGGTTCTTAGGTCACTTCTCCAACGGTTTTGTGTGGACAGA
ATACATTGCCAAAGCGAAGAACCTTCCGCTCTACAACTGGGCAGTTGGCGGCGCGGCTGGTGAGAATCAA
TACATCGCGCTAACAGGGGTTGGTGATCAAGTTTCTTCGTACTTAACCTACGCAAAACTGGCGAAGAACT
ACAAACCAGCAAACACCTTGTTTACGCTTGAGTTTGGTTTGAATGACTTCATGAACTACAACCGTGGCGT
TCCAGAAGTGAAAGCAGATTATGCAGAAGCACTGATTCGTTTGACGGACGCAGGTGCGAAGAACTTCATG
TTGATGACACTGCCAGATGCGACGAAAGCGCCTCAGTTTAAGTACTCAACACAAGAAGAGATCGACAAAA
TTCGTGCGAAAGTGCTTGAGATGAACGAGTTCATCAAGGCACAAGCGATGTACTACAAAGCGCAAGGTTA
CAACATCACGTTGTTTGATACTCACGCCTTGTTCGAGACGCTAACTTCTGCGCCAGAAGAGCACGGTTTC
GTGAACGCGAGTGATCCTTGTTTGGACATCAACCGCTCATCGTCTGTCGATTACATGTACACCCACGCAT
TGCGCTCTGAGTGTGCGGCGTCTGGTGCTGAGAAGTTTGTGTTCTGGGATGTCACGCATCCAACAACAGC
AACTCACCGCTATGTTGCAGAGAAAATGCTAGAAAGTAGCAACAACTTAGCCGAGTACCGTTTCTAA

>S013
ATGATGAAAAAAACAATCACACTATTAACTGCATTACTCCCGCTTGCTTCTGCAGTTGCCGAAGAGCCAACCTTAT
CACCAGAAATGGTTTCAGCGTCTGAAGTGATCAGCACGCAAGAAAACCAAACCTATACCTATGTTCGCTGTTGGTA
TCGCACCAGCTACTCGAAAGATGATCCAGCGACCGATTGGGAATGGGCAAAAAACGAAGATGGTAGCTACTTCAC
CATTGACGGCTACTGGTGGAGCTCCGTTTCATTTAAAAACATGTTCTACACCAACACGTCGCAAAACGTTATCCGT
CAGCGTTGTGAAGCAACATTAGATTTGGCGAACGAGAACGCAGACATTACGTTCTTCGCCGCTGACAATCGCTTCT
CATACAACCACACGATCTGGAGCAACGACGCAGCAATGCAGCCAGATCAAATCAACAAAGTGGTTGCACTCGGTG
ACAGCTTGTCTGATACAGGCAACATCTTTAACGCATCACAATGGCGCTTCCCTAACCCGAACAGCTGGTTCTTAGG
TCACTTCTCCAACGGTTTTGTTTGGACAGAATACATTGCCAAAGCGAAGAACCTTCCGCTCTACAACTGGGCAGTT
GGCGGCGCGGCTGGTGAGAACCAATACATCGCGCTAACAGGGGTTGGTGAGCAAGTTTCTTCGTACTTAACCTACG
CAAAACTGGCAAAGAACTACAAACCAGCAAACACCTTGTTTACGCTTGAGTTTGGTTTGAATGACTTCATGAACTA
CAACCGTGGCGTTCCAGAAGTGAAAGCGGATTATGCAGAAGCACTGATTCGTTTGACGGACGCAGGTGCGAAGAA
CTTCATGTTGATGACACTGCCAGATGCGACGAAAGCGCCTCAGTTTAAGTACTCAACACAAGAAGAGATCGACAA
AATTCGTGCGAAAGTGCTTGAGATGAACGAGTTCATCAAGGCACAAGCGATGTACTACAAAGCGCAAGGTTACAA
CATCACGTTGTTTGATACTCACGCCTTGTTCGAGACGCTAACTTCTGCGCCAGAAGAGCACGGTTTCGTGAACGCG
AGCGATCCTTGTTTGGACATCAACCGCTCATCGTCTGTCGATTACATGTACACCCACGCATTGCGCTCTGAGTGTGC
AGCGTCTGGTGCTGAGAAGTTTGTGTTCTGGGATGTCACGCATCCAACAACAGCAACTCACCGCTATGTTGCAGAG
AAAATGCTAGAAAGTAGCAACAACTTAGCCGAGTACCGTTTCTAA

>S012
ATGATGAAAAAAACAATCACACTATTAACTGCATTACTCCCGCTTGCTTCTGCAGTTGCCGAAGAGCCAA
CCTTATCACCAGAAATGGTTTCAGCGTCTGAAGTGATCAGCACGCAAGAAAACCAAACCTATACCTATGT
TCGCTGTTGGTATCGCACCAGCTACTCGAAAGATGATCCAGCGACCGATTGGGAATGGGCAAAAAACGAA
GATGGTAGCTACTTCACCATTGACGGCTACTGGTGGAGCTCCGTTTCATTTAAAAACATGTTCTACACCA
ACACGTCGCAAAACGTTATCCGTCAGCGTTGTGAAGCAACATTAGATTTGGCGAACGAGAACGCAGACAT
TACGTTCTTCGCCGCTGACAATCGCTTCTCATACAACCACACGATCTGGAGCAACGACGCAGCAATGCAG
CCAGATCAAATCAACAAAGTGGTTGCACTCGGTGACAGCTTGTCTGATACAGGCAACATCTTTAACGCAT
CACAATGGCGCTTCCCTAACCCGAACAGCTGGTTCTTAGGTCACTTCTCCAACGGTTTTGTTTGGACAGA
ATACATTGCCAAAGCGAAGAACCTTCCGCTCTACAACTGGGCAGTTGGCGGCGCGGCTGGTGAGAACCAA
TACATCGCGCTAACAGGGGTTGGTGAGCAAGTTTCTTCGTACTTAACCTACGCAAAACTGGCAAAGAACT
ACAAACCAGCAAACACCTTGTTTACGCTTGAGTTTGGTTTGAATGACTTCATGAACTACAACCGTGGCGT
TCCAGAAGTGAAAGCGGATTATGCAGAAGCACTGATTCGTTTGACGGACGCAGGTGCGAAGAACTTCATG
TTGATGACACTGCCAGATGCGACGAAAGCGCCTCAGTTTAAGTACTCAACACAAGAAGAGATCGACAAAA
TTCGTGCGAAAGTGCTTGAGATGAACGAGTTCATCAAGGCACAAGCGATGTACTACAAAGCGCAAGGTTA
CAACATCACGTTGTTTGATACTCACGCCTTGTTCGAGACGCTAACTTCTGCGCCAGAAGAGCACGGTTTC
GTGAACGCGAGCGATCCTTGTTTGGACATCAACCGCTCATCGTCTGTCGATTACATGTACACCCACGCAT
TGCGCTCTGAGTGTGCAGCGTCTGGTGCTGAGAAGTTTGTGTTCTGGGATGTCACGCATCCAACAACAGC
AACTCACCGCTATGTTGCAGAGAAAATGCTAGAAAGTAGCAACAACTTAGCCGAGTACCGTTTCTAA

>S011
ATGATGAAAAAAACAATCACACTATTAACTGCATTACTCCCGCTTGCTTCTGCAGTTGCCGAAGAGCCAACCTTAT
CACCAGAAATGGTTTCAGCGTCTGAAGTGATCAGCACGCAAGAAAACCAAACCTATACCTATGTTCGCTGTTGGTA
TCGCACCAGCTACTCGAAAGATGATCCAGCGACCGATTGGGAATGGGCAAAAAACGAAGATGGTAGCTACTTCAC
CATTGACGGCTACTGGTGGAGCTCCGTTTCATTTAAAAACATGTTCTACACCAACACGTCGCAAAACGTTATCCGT
CAGCGTTGTGAAGCAACATTAGATTTGGCGAACGAGAACGCAGACATTACGTTCTTCGCCGCTGACAATCGCTTCT
CATACAACCACACGATCTGGAGCAACGACGCAGCAATGCAGCCAGATCAAATCAACAAAGTGGTTGCACTCGGTG
ACAGCTTGTCTGATACAGGCAACATCTTTAACGCATCACAATGGCGCTTCCCTAACCCGAACAGCTGGTTCTTAGG
TCACTTCTCCAACGGTTTTGTTTGGACAGAATACATTGCCAAAGCGAAGAACCTTCCGCTCTACAACTGGGCAGTT
GGCGGCGCGGCTGGTGAGAACCAATACATCGCGCTAACAGGGGTTGGTGAGCAAGTTTCTTCGTACTTAACCTACG
CAAAACTGGCAAAGAACTACAAACCAGCAAACACCTTGTTTACGCTTGAGTTTGGTTTGAATGACTTCATGAACTA
CAACCGTGGCGTTCCAGAAGTGAAAGCGGATTATGCAGAAGCACTGATTCGTTTGACGGACGCAGGTGCGAAGAA
CTTCATGTTGATGACACTGCCAGATGCGACGAAAGCGCCTCAGTTTAAGTACTCAACACAAGAAGAGATCGACAA
AATTCGTGCGAAAGTGCTTGAGATGAACGAGTTCATCAAGGCACAAGCGATGTACTACAAAGCGCAAGGTTACAA
CATCACGTTGTTTGATACTCACGCCTTGTTCGAGACGCTAACTTCTGCGCCAGAAGAGCACGGTTTCGTGAACGCG
AGCGATCCTTGTTTGGACATCAACCGCTCATCGTCTGTCGATTACATGTACACCCACGCATTGCGCTCTGAGTGTGC
AGCGTCTGGTGCTGAGAAGTTTGTGTTCTGGGATGTCACGCATCCAACAACAGCAACTCACCGCTATGTTGCAGAG
AAAATGCTAGAAAGTAGCAACAACTTAGCCGAGTACCGTTTCTAA

>S009
ATGATGAAAAAAACAATCACACTATTAACTGCATTACTCCCGCTTGCTTCTGCAGTTGCCGAAGAGCCAA
CCTTATCACCAGAAATGGTTTCAGCGTCTGAAGTGATCAGCACGCAAGAAAACCAAACCTATACCTATGT
TCGCTGTTGGTATCGCACCAGCTACTCGAAAGATGATCCAGCGACCGATTGGGAATGGGCAAAAAACGAA
GATGGTAGCTACTTCACCATTGACGGCTACTGGTGGAGCTCCGTTTCATTTAAAAACATGTTCTACACCA
ACACGTCGCAAAACGTTATCCGTCAGCGTTGTGAAGCAACATTAGATTTGGCGAACGAGAACGCAGACAT
TACGTTCTTCGCCGCTGACAATCGCTTCTCATACAACCACACGATCTGGAGCAACGACGCAGCAATGCAG
CCAGATCAAATCAACAAAGTGGTTGCACTCGGTGACAGCTTGTCTGATACAGGCAACATCTTTAACGCAT
CACAATGGCGCTTCCCTAACCCGAACAGCTGGTTCTTAGGTCACTTCTCCAACGGTTTTGTTTGGACAGA
ATACATTGCCAAAGCGAAGAACCTTCCGCTCTACAACTGGGCAGTTGGCGGCGCGGCTGGTGAGAACCAA
TACATCGCGCTAACAGGGGTTGGTGAGCAAGTTTCTTCGTACTTAACCTACGCAAAACTGGCAAAGAACT

ACAAACCAGCAAACACCTTGTTTACGCTTGAGTTTGGTTTGAATGACTTCATGAACTACAACCGTGGCGT
TCCAGAAGTGAAAGCGGATTATGCAGAAGCACTGATTCGTTTGACGGACGCAGGTGCGAAGAACTTCATG
TTGATGACACTGCCAGATGCGACGAAAGCGCCTCAGTTTAAGTACTCAACACAAGAAGAGATCGACAAAA
TTCGTGCGAAAGTGCTTGAGATGAACGAGTTCATCAAGGCACAAGCGATGTACTACAAAGCGCAAGGTTA
CAACATCACGTTGTTTGATACTCACGCCTTGTTCGAGACGCTAACTTCTGCGCCAGAAGAGCACGGTTTC
GTGAACGCGAGCGATCCTTGTTTGGACATCAACCGCTCATCGTCTGTCGATTACATGTACACCCACGCAT
TGCGCTCTGAGTGTGCAGCGTCTGGTGCTGAGAAGTTTGTGTTCTGGGATGTCACGCATCCAACAACAGC
AACTCACCGCTATGTTGCAGAGAAAATGCTAGAAAGTAGCAACAACTTAGCCGAGTACCGTTTCTAA

>S008
ATGATGAAAAAAACAATCACACTATTAACTGCATTACTCCCGCTTGCTTCTGCAGTTGCCGAAGAGCCAA
CCTTATCACCAGAAATGGTTTCAGCGTCTGAAGTGATCAGCACGCAAGAAAACCAAACCTATACCTATGT
TCGCTGTTGGTATCGCACCAGCTACTCGAAAGATGATCCAGCGACCGATTGGGAATGGGCAAAAAACGAA
GATGGTAGCTACTTCACCATTGACGGCTACTGGTGGAGCTCCGTTTCATTTAAAAACATGTTCTACACCA
ACACGTCGCAAAACGTTATCCGTCAGCGTTGTGAAGCAACATTAGATTTGGCGAACGAGAACGCAGACAT
TACGTTCTTCGCCGCTGACAATCGCTTCTCATACAACCACACGATCTGGAGCAACGACGCAGCAATGCAG
CCAGATCAAATCAACAAAGTGGTTGCACTCGGTGACAGCTTGTCTGATACAGGCAACATCTTTAACGCAT
CACAATGGCGCTTCCCTAACCCGAACAGCTGGTTCTTAGGTCACTTCTCCAACGGTTTTGTTTGGACAGA
ATACATTGCCAAAGCGAAGAACCTTCCGCTCTACAACTGGGCAGTTGGCGGCGCGGCTGGTGAGAACCAA
TACATCGCGCTAACAGGGGTTGGTGAGCAAGTTTCTTCGTACTTAACCTACGCAAAACTGGCAAAGAACT
ACAAACCAGCAAACACCTTGTTTACGCTTGAGTTTGGTTTGAATGACTTCATGAACTACAACCGTGGCGT
TCCAGAAGTGAAAGCGGATTATGCAGAAGCACTGATTCGTTTGACGGACGCAGGTGCGAAGAACTTCATG
TTGATGACACTGCCAGATGCGACGAAAGCGCCTCAGTTTAAGTACTCAACACAAGAAGAGATCGACAAAA
TTCGTGCGAAAGTGCTTGAGATGAACGAGTTCATCAAGGCACAAGCGATGTACTACAAAGCGCAAGGTTA
CAACATCACGTTGTTTGATACTCACGCCTTGTTCGAGACGCTAACTTCTGCGCCAGAAGAGCACGGTTTC
GTGAACGCGAGCGATCCTTGTTTGGACATCAACCGCTCATCGTCTGTCGATTACATGTACACCCACGCAT
TGCGCTCTGAGTGTGCAGCGTCTGGTGCTGAGAAGTTTGTGTTCTGGGATGTCACGCATCCAACAACAGC
AACTCACCGCTATGTTGCAGAGAAAATGCTAGAAAGTAGCAACAACTTAGCCGAGTACCGTTTCTAA

>S005
ATGATGAAAAAAACAATCACACTATTAACTGCATTACTCCCGCTTGCTTCTGCAGTTGCCGAAGAGCCAA
CCTTATCACCAGAAATGGTTTCAGCGTCTGAAGTGATCAGCACGCAAGAAAACCAAACCTATACCTATGT
TCGCTGTTGGTATCGCACCAGCTACTCGAAAGATGATCCAGCGACCGATTGGGAATGGGCAAAAAACGAA
GATGGTAGCTACTTCACCATTGACGGCTACTGGTGGAGCTCCGTTTCATTTAAAAACATGTTCTACACCA
ACACGTCGCAAAACGTTATCCGTCAGCGTTGTGAAGCAACATTAGATTTGGCGAACGAGAACGCAGACAT
TACGTTCTTCGCCGCTGACAATCGCTTCTCATACAACCACACGATCTGGAGCAACGACGCAGCAATGCAG
CCAGATCAAATCAACAAAGTGGTTGCACTCGGTGACAGCTTGTCTGATACAGGCAACATCTTTAACGCAT
CACAATGGCGCTTCCCTAACCCGAACAGCTGGTTCTTAGGTCACTTCTCCAACGGTTTTGTTTGGACAGA
ATACATTGCCAAAGCGAAGAACCTTCCGCTCTACAACTGGGCAGTTGGCGGCGCGGCTGGTGAGAACCAA
TACATCGCGCTAACAGGGGTTGGTGAGCAAGTTTCTTCGTACTTAACCTACGCAAAACTGGCAAAGAACT
ACAAACCAGCAAACACCTTGTTTACGCTTGAGTTTGGTTTGAATGACTTCATGAACTACAACCGTGGCGT
TCCAGAAGTGAAAGCGGATTATGCAGAAGCACTGATTCGTTTGACGGACGCAGGTGCGAAGAACTTCATG
TTGATGACACTGCCAGATGCGACGAAAGCGCCTCAGTTTAAGTACTCAACACAAGAAGAGATCGACAAAA
TTCGTGCGAAAGTGCTTGAGATGAACGAGTTCATCAAGGCACAAGCGATGTACTACAAAGCGCAAGGTTA
CAACATCACGTTGTTTGATACTCACGCCTTGTTCGAGACGCTAACTTCTGCGCCAGAAGAGCACGGTTTC
GTGAACGCGAGCGATCCTTGTTTGGACATCAACCGCTCATCGTCTGTCGATTACATGTACACCCACGCAT
TGCGCTCTGAGTGTGCAGCGTCTGGTGCTGAGAAGTTTGTGTTCTGGGATGTCACGCATCCAACAACAGC
AACTCACCGCTATGTTGCAGAGAAAATGCTAGAAAGTAGCAACAACTTAGCCGAGTACCGTTTCTAA

>S004

ATGATGAAAAAAACAATCACACTATTAACTGCATTACTCCCGCTTGCTTCTGCAGTTGCCGAAGAGCCAACCTTAT
CACCAGAAATGGTTTCAGCGTCTGAAGTGATCAGCACGCAAGAAAACCAAACCTATACCTATGTTCGCTGTTGGTA
TCGCACCAGCTACTCGAAAGATGATCCAGCGACCGATTGGGAATGGGCAAAAAACGAAGATGGTAGCTACTTCAC
CATTGACGGCTACTGGTGGAGCTCCGTTTCATTTAAAAACATGTTCTACACCAACACGTCGCAAAACGTTATCCGT
CAGCGTTGTGAAGCAACATTAGATTTGGCGAACGAGAACGCAGACATTACGTTCTTCGCCGCTGACAATCGCTTCT
CATACAACCACACGATCTGGAGCAACGACGCAGCAATGCAGCCAGATCAAATCAACAAAGTGGTTGCACTCGGTG
ACAGCTTGTCTGATACAGGCAACATCTTTAACGCATCACAATGGCGCTTCCCTAACCCGAACAGCTGGTTCTTAGG
TCACTTCTCCAACGGTTTTGTTTGGACAGAATACATTGCCAAAGCGAAGAACCTTCCGCTCTACAACTGGGCAGTT
GGCGGCGCGGCTGGTGAGAACCAATACATCGCGCTAACAGGGGTTGGTGAGCAAGTTTCTTCGTACTTAACCTACG
CAAAACTGGCAAAGAACTACAAACCAGCAAACACCTTGTTTACGCTTGAGTTTGGTTTGAATGACTTCATGAACTA
CAACCGTGGCGTTCCAGAAGTGAAAGCGGATTATGCAGAAGCACTGATTCGTTTGACGGACGCAGGTGCGAAGAA
CTTCATGTTGATGACACTGCCAGATGCGACGAAAGCGCCTCAGTTTAAGTACTCAACACAAGAAGAGATCGACAA
AATTCGTGCGAAAGTGCTTGAGATGAACGAGTTCATCAAGGCACAAGCGATGTACTACAAAGCGCAAGGTTACAA
CATCACGTTGTTTGATACTCACGCCTTGTTCGAGACGCTAACTTCTGCGCCAGAAGAGCACGGTTTCGTGAACGCG
AGCGATCCTTGTTTGGACATCAACCGCTCATCGTCTGTCGATTACATGTACACCCACGCATTGCGCTCTGAGTGTGC
AGCGTCTGGTGCTGAGAAGTTTGTGTTCTGGGATGTCACGCATCCAACAACAGCAACTCACCGCTATGTTGCAGAG
AAAATGCTAGAAAGTAGCAACAACTTAGCCGAGTACCGTTTCTAA

>S003
ATGATGAAAAAAACAATCACACTATTAACTGCATTACTCCCGCTTGCTTCTGCAGTTGCCGAAGAGCCAACCTTAT
CACCAGAAATGGTTTCAGCGTCTGAAGTGATCAGCACGCAAGAAAACCAAACCTATACCTATGTTCGCTGTTGGTA
TCGCACCAGCTACTCGAAAGATGATCCAGCGACCGATTGGGAATGGGCAAAAAACGAAGATGGTAGCTACTTCAC
CATTGACGGCTACTGGTGGAGCTCCGTTTCATTTAAAAACATGTTCTACACCAACACGTCGCAAAACGTTATCCGT
CAGCGTTGTGAAGCAACATTAGATTTGGCGAACGAGAACGCAGACATTACGTTCTTCGCCGCTGACAATCGCTTCT
CATACAACCACACGATCTGGAGCAACGACGCAGCAATGCAGCCAGATCAAATCAACAAAGTGGTTGCACTCGGTG
ACAGCTTGTCTGATACAGGCAACATCTTTAACGCATCACAATGGCGCTTCCCTAACCCGAACAGCTGGTTCTTAGG
TCACTTCTCCAACGGTTTTGTTTGGACAGAATACATTGCCAAAGCGAAGAACCTTCCGCTCTACAACTGGGCAGTT
GGCGGCGCGGCTGGTGAGAACCAATACATCGCGCTAACAGGGGTTGGTGAGCAAGTTTCTTCGTACTTAACCTACG
CAAAACTGGCAAAGAACTACAAACCAGCAAACACCTTGTTTACGCTTGAGTTTGGTTTGAATGACTTCATGAACTA
CAACCGTGGCGTTCCAGAAGTGAAAGCGGATTATGCAGAAGCACTGATTCGTTTGACGGACGCAGGTGCGAAGAA
CTTCATGTTGATGACACTGCCAGATGCGACGAAAGCGCCTCAGTTTAAGTACTCAACACAAGAAGAGATCGACAA
AATTCGTGCGAAAGTGCTTGAGATGAACGAGTTCATCAAGGCACAAGCGATGTACTACAAAGCGCAAGGTTACAA
CATCACGTTGTTTGATACTCACGCCTTGTTCGAGACGCTAACTTCTGCGCCAGAAGAGCACGGTTTCGTGAACGCG
AGCGATCCTTGTTTGGACATCAACCGCTCATCGTCTGTCGATTACATGTACACCCACGCATTGCGCTCTGAGTGTGC
AGCGTCTGGTGCTGAGAAGTTTGTGTTCTGGGATGTCACGCATCCAACAACAGCAACTCACCGCTATGTTGCAGAG
AAAATGCTAGAAAGTAGCAACAACTTAGCCGAGTACCGTTTCTAA

>S002
ATGATGAAAAAAACAATCACACTATTAACTGCATTACTCCCGCTTGCTTCTGCAGTTGCCGAAGAGCCAACCTTAT
CACCAGAAATGGTTTCAGCGTCTGAAGTGATCAGCACGCAAGAAAACCAAACCTATACCTATGTTCGCTGTTGGTA
TCGCACCAGCTACTCGAAAGATGATCCGGCGACCGATTGGGAATGGGCAAAAAACGAAGATGGTAGCTACTTCAC
CATTGACGGCTACTGGTGGAGCTCCGTTTCATTTAAAAACATGTTCTACACCAACACGTCGCAAAACGTTATCCGT
CAGCGTTGTGAAGCAACATTAGATTTGGCGAACGAGAACGCAGACATTACGTTCTTCGCCGCTGACAATCGCTTCT
CATACAACCACACGATCTGGAGCAACGACGCAGCAATGCAGCCAGATCAAATCAACAAAGTGGTTGCACTCGGTG
ACAGCTTGTCTGATACAGGCAACATCTTTAACGCATCACAATGGCGCTTCCCTAACCCGAACAGCTGGTTCTTAGG
TCACTTCTCCAACGGTTTTGTGTGGACAGAATACATTGCCAAAGCGAAGAACCTTCCGCTCTACAACTGGGCAGTT
GGCGGCGCGGCTGGTGAGAACCAATACATCGCGCTAACAGGGGTTGGTGATCAAGTTTCTTCGTACTTAACCTACG
CAAAACTGGCGAAGAACTACAAACCAGCAAACACCTTGTTTACGCTTGAGTTTGGTTTGAATGACTTCATGAACTA
CAACCGTGGCGTTCCAGAAGTGAAAGCAGATTATGCAGAAGCACTGATTCGTTTGACGGACGCAGGTGCGAAGAA
CTTCATGTTGATGACACTGCCAGACGCGACGAAAGCGCCTCAGTTTAAGTACTCAACACAAGAAGAGATCGACAA
AATTCGTGCGAAAGTGCTTGAGATGAACGAGTTCATCAAGGCACAAGCGATGTACTACAAAGCGCAAGGTTACAA

CATCACGTTGTTTGATACTCACGCCTTGTTCGAGACGCTAACTTCTGCGCCAGAAGAGCACGGTTTCGTGAACGCG
AGTGATCCTTGTTTGGACATCAACCGCTCATCGTCTGTCGATTACATGTACACCCACGCATTGCGCTCTGAGTGTGC
GGCGTCTGGTGCTGAGAAGTTTGTGTTCTGGGATGTCACGCACCCAACAACAGCAACTCACCGCTATGTTGCAGAG
AAAATGCTAGAAAGTAGCAACAACTTAGCCGAGTACCGTTTCTAA

>S001
ATGATGAAAAAAACAATCACACTATTAACTGCATTACTCCCGCTTGCTTCTGCAGTTGCCGAAGAGCCAACCTTAT
CACCAGAAATGGTTTCAGCGTCTGAAGTGATCAGCACGCAAGAAAACCAAACCTATACCTATGTTCGCTGTTGGTA
TCGCACCAGCTACTCGAAAGATGATCCGGCGACCGATTGGGAATGGGCAAAAAACGAAGATGGTAGCTACTTCAC
CATTGACGGCTACTGGTGGAGCTCCGTTTCATTTAAAAACATGTTCTACACCAACACGTCGCAAAACGTTATCCGT
CAGCGTTGTGAAGCAACATTAGATTTGGCGAACGAGAACGCAGACATTACGTTCTTCGCCGCTGACAATCGCTTCT
CATACAACCACACGATCTGGAGCAACGACGCAGCAATGCAGCCAGATCAAATCAACAAAGTGGTTGCACTCGGTG
ACAGCTTGTCTGATACAGGCAACATCTTTAACGCATCACAATGGCGCTTCCCTAACCCGAACAGCTGGTTCTTAGG
TCACTTCTCCAACGGTTTTGTTTGGACAGAATACATTGCCAAAGCGAAGAACCTTCCGCTCTACAACTGGGCAGTT
GGCGGCGCGGCTGGTGAGAACCAATACATCGCGCTAACAGGGGTTGGTGAGCAAGTTTCTTCGTACTTAACCTACG
CAAAACTGGCGAAGAACTACAAACCAGCAAACACCTTGTTTACGCTTGAGTTTGGTTTAAATGACTTCATGAACTA
CAACCGTGGCGTTCCAGAAGTGAAAGCGGATTATGCAGAAGCACTGATTCGTTTGACGGACGCAGGTGCGAAGAA
CTTCATGTTGATGACACTGCCAGACGCGACGAAAGCGCCTCAGTTTAAGTACTCAACACAAGAAGAGATCGACAA
AATTCGTGCGAAAGTGCTTGAGATGAACGAGTTCATCAAGGCACAAGCGATGTACTACAAAGCGCAAGGTTACAA
CATCACGTTGTTTGATACTCACGCCTTGTTCGAGACGCTAACTTCTGCGCCAGAAGAGCACGGTTTCGTGAACGCG
AGTGATCCTTGTTTGGACATCAACCGCTCATCGTCTGTCGATTACATGTACACCCACGCATTGCGCTCTGAGTGTGC
GGCGTCCGGTGCTGAGAAATTTGTGTTCTGGGATGTCACGCACCCAACAACAGCAACTCACCGCTATGTTGCAGAG
AAAATGCTAGAAAGTAGCAACAACTTAGCCGAGTACCGTTTCTAA

>S096
ATGATGAAAAAAACAATCACACTATTAACTGCATTACTCCCGCTTGCTTCTGCAGTTGCCGAAGAGCCAACCTTAT
CACCAGAAATGGTTTCAGCGTCTGAAGTGATCAGCACGCAAGAAAACCAAACCTATACCTATGTTCGCTGTTGGTA
TCGCACCAGCTACTCGAAAGATGATCCGGCGACCGATTGGGAATGGGCAAAAAACGAAGATGGTAGCTACTTCAC
CATTGACGGCTACTGGTGGAGCTCCGTTTCATTTAAAAACATGTTCTACACCAACACGTCGCAAAACGTTATCCGT
CAGCGTTGTGAAGCAACATTAGATTTGGCGAACGAGAACGCAGACATTACGTTCTTCGCCGCTGACAATCGCTTCT
CATACAACCACACGATCTGGAGCAACGACGCAGCAATGCAGCCAGATCAAATCAACAAAGTGGTTGCACTCGGTG
ACAGCTTGTCTGATACAGGCAACATCTTTAACGCATCACAATGGCGCTTCCCTAACCCGAACAGCTGGTTCTTAGG
TCACTTCTCCAACGGTTTTGTGTGGACAGAATACATTGCCAAAGCGAAGAACCTTCCGCTCTACAACTGGGCAGTT
GGCGGCGCGGCTGGTGAGAACCAATACATCGCGCTAACAGGGGTTGGTGATCAAGTTTCTTCGTACTTAACCTACG
CAAAACTGGCGAAGAACTACAAACCAGCAAACACCTTGTTTACGCTTGAGTTTGGTTTGAATGACTTCATGAACTA
CAACCGTGGCGTTCCAGAAGTGAAAGCGGATTATGCAGAAGCACTGATTCGTTTGACGGACGCAGGTGCGAAGAA
CTTCATGTTGATGACACTGCCAGACGCGACGAAAGCGCCTCAGTTTAAGTACTCAACACAAGAAGAGATCGACAA
AATTCGTGCGAAAGTGCTTGAGATGAACGAGTTCATCAAGGCACAAGCGATGTACTACAAAGCGCAAGGTTACAA
CATCACGTTGTTTGATACTCACGCCTTGTTCGAGACGCTAACTTCTGCGCCAGAAGAGCACGGTTTCGTGAACGCG
AGCGATCCTTGTTTGGACATCAACCGCTCATCGTCTGTCGATTACATGTACACCCACGCATTGCGCTCTGAGTGTGC
AGCGTCTGGTGCTGAGAAGTTTGTGTTCTGGGATGTCACGCATCCAACAACAGCAACTCACCGCTATGTTGCAGAG
AAAATGCTAGGAAGTAGCAACAACTTAGCAGAGTACCGTTTCTAA

>S092
ATGATGAAAAAAACAATCACACTATTAACTGCATTACTCCCGCTTGCTTCTGCAGTTGCCGAAGAGCCAACCTTAT
CACCAGAAATGGTTTCAGCGTCTGAAGTGATCAGCACGCAAGAAAACCAAACCTATACCTATGTTCGCTGTTGGTA
TCGCACCAGCTACTCGAAAGATGATCCAGCGACCGATTGGGAATGGGCAAAAAACGAAGATGGTAGCTACTTCAC
CATTGACGGCTACTGGTGGAGCTCCGTTTCATTTAAAAACATGTTCTACACCAACACGTCGCAAAACGTTATCCGT
CAGCGTTGTGAAGCAACATTAGATTTGGCGAACGAGAACGCAGACATTACGTTCTTCGCCGCTGACAATCGCTTCT
CATACAACCACACGATCTGGAGCAACGACGCAGCAATGCAGCCAGATCAAATCAACAAAGTGGTTGCACTCGGTG
ACAGCTTGTCTGATACAGGCAACATCTTTAACGCATCACAATGGCGCTTCCCTAACCCGAACAGCTGGTTCTTAGG

TCACTTCTCCAACGGTTTTGTTTGGACAGAATACATTGCCAAAGCGAAGAACCTTCCGCTCTACAACTGGGCAGTT
GGCGGCGCGGCTGGTGAGAACCAATACATCGCGCTAACAGGGGTTGGTGAGCAAGTTTCTTCGTACTTAACCTACG
CAAAACTGGCGAAGAACTACAAACCAGCAAACACCTTGTTTACGCTTGAGTTTGGTTTGAATGACTTCATGAACTA
CAACCGTGGCGTTCCAGAAGTGAAAGCGGATTATGCAGAAGCACTGATTCGTTTGACGGACGCAGGTGCGAAGAA
CTTCATGTTGATGACACTGCCAGATGCGACGAAAGCGCCTCAGTTTAAGTACTCAACACAAGAAGAGATCGACAA
AATTCGTGCGAAAGTGCTTGAGATGAACGAGTTCATCAAGGCACAAGCGATGTACTACAAAGCGCAAGGTTACAA
CATCACGTTGTTTGATACTCACGCCTTGTTCGAGACGCTAACTTCTGCGCCAGAAGAGCACGGTTTCGTGAACGCG
AGCGATCCTTGTTTGGACATCAACCGCTCATCGTCTGTCGATTACATGTACACCCACGCATTGCGCTCTGAGTGTGC
AGCGTCTGGTGCTGAGAAGTTTGTGTTCTGGGATGTCACGCATCCAACAACAGCAACTCACCGCTATGTTGCAGAG
AAAATGCTAGAAAGTAGCAACAACTTAGCCGAGTACCGTTTCTAA

>VIP4-0439
ATGATGAAAAAAACAATCACACTATTAACTGCATTACTCCCGCTTGCTTCTGCAGTTGCCGAAGAGCCAA
CCTTATCACCAGAAATGGTTTCAGCGTCTGAAGTGATCAGTACGCAAGAAAACCAAACCTATACCTATGT
TCGCTGTTGGTATCGCACCAGCTACTCGAAAGATGATCCAGCGACCGATTGGGAATGGGCAAAAAACGAA
GATGGTAGCTACTTCACCATTGACGGCTACTGGTGGAGCTCCGTTTCATTTAAAAACATGTTCTACACCA
ACACGTCGCAAAACGTTATCCGTCAGCGTTGTGAAGCAACATTAGATTTGGCGAACGAGAACGCAGACAT
TACGTTCTTCGCCGCTGACAATCGCTTCTCATACAACCACACGATCTGGAGCAACGACGCAGCAATGCAG
CCAGATCAAATCAACAAAGTGGTTGCACTCGGTGACAGCTTGTCTGATACAGGCAACATCTTTAACGCAT
CACAATGGCGCTTCCCTAACCCGAACAGCTGGTTCTTAGGTCACTTCTCCAACGGTTTTGTTTGGACAGA
ATACATTGCCAAAGCGAAGAACCTTCCGCTCTACAACTGGGCAGTTGGCGGCGCGGCTGGTGAGAACCAA
TACATCGCGCTAACAGGGGTTGGTGAGCAAGTTTCTTCGTACTTAACCTACGCAAAACTGGCGAAGAACT
ACAAACCAGCAAACACCTTGTTTACGCTTGAGTTTGGTTTGAATGACTTCATGAACTACAACCGTGGCGT
TCCAGAAGTGAAAGCGGATTATGCAGAAGCACTGATTCGTTTGACGGACGCAGGTGCGAAGAACTTCATG
TTGATGACACTGCCAGATGCGACGAAAGCGCCTCAGTTTAAGTACTCAACACAAGAAGAGATCGACAAAA
TTCGTGCGAAAGTGCTTGAGATGAACGAGTTCATCAAGGCACAAGCGATGTACTACAAAGCGCAAGGTTA
CAACATCACGTTGTTTGATACTCACGCCTTGTTCGAGACGCTAACTTCTGCGCCAGAAGAGCACGGTTTC
GTGAACGCGAGCGATCCTTGTTTGGACATCAACCGCTCATCGTCTGTCGATTACATGTACACCCACGCAT
TGCGCTCTGAGTGTGCAGCGTCTGGTGCTGAGAAGTTTGTGTTCTGGGATGTCACGCATCCAACAACAGC
AACTCACCGCTATGTTGCAGAGAAAATGCTAGAAAGTAGCAACAACTTAGCCGAGTACCGTTTCTAA

>VIP4-0445
ATGATGAAAAAAACAATCACACTATTAACTGCATTACTCCCGCTTGCTTCTGCAGTTGCCGAAGAGCCAA
CCTTATCACCAGAAATGGTTTCAGCGTCTGAAGTGATCAGTACGCAAGAAAACCAAACCTATACCTATGT
TCGCTGTTGGTATCGCACCAGCTACTCGAAAGATGATCCAGCGACCGATTGGGAATGGGCAAAAAACGAA
GATGGTAGCTACTTCACCATTGACGGCTACTGGTGGAGCTCCGTTTCATTTAAAAACATGTTCTACACCA
ACACGTCGCAAAACGTTATCCGTCAGCGTTGTGAAGCAACATTAGATTTGGCGAACGAGAACGCAGACAT
TACGTTCTTCGCCGCTGACAATCGCTTCTCATACAACCACACGATCTGGAGCAACGACGCAGCAATGCAG
CCAGATCAAATCAACAAAGTGGTTGCACTCGGTGACAGCTTGTCTGATACAGGCAACATCTTTAACGCAT
CACAATGGCGCTTCCCTAACCCGAACAGCTGGTTCTTAGGTCACTTCTCCAACGGTTTTGTTTGGACAGA
ATACATTGCCAAAGCGAAGAACCTTCCGCTCTACAACTGGGCAGTTGGCGGCGCGGCTGGTGAGAACCAA
TACATCGCGCTAACAGGGGTTGGTGAGCAAGTTTCTTCGTACTTAACCTACGCAAAACTGGCGAAGAACT
ACAAACCAGCAAACACCTTGTTTACGCTTGAGTTTGGTTTGAATGACTTCATGAACTACAACCGTGGCGT
TCCAGAAGTGAAAGCGGATTATGCAGAAGCACTGATTCGTTTGACGGACGCAGGTGCGAAGAACTTCATG
TTGATGACACTGCCAGATGCGACGAAAGCGCCTCAGTTTAAGTACTCAACACAAGAAGAGATCGACAAAA
TTCGTGCGAAAGTGCTTGAGATGAACGAGTTCATCAAGGCACAAGCGATGTACTACAAAGCGCAAGGTTA
CAACATCACGTTGTTTGATACTCACGCCTTGTTCGAGACGCTAACTTCTGCGCCAGAAGAGCACGGTTTC
GTGAACGCGAGCGATCCTTGTTTGGACATCAACCGCTCATCGTCTGTCGATTACATGTACACCCACGCAT
TGCGCTCTGAGTGTGCAGCGTCTGGTGCTGAGAAGTTTGTGTTCTGGGATGTCACGCATCCAACAACAGC
AACTCACCGCTATGTTGCAGAGAAAATGCTAGAAAGTAGCAACAACTTAGCCGAGTACCGTTTCTAA

>VIP4-0407
ATGATGAAAAAAACAATCACACTATTAACTGCATTACTCCCGCTTGCTTCTGCAGTTGCCGAAGAGCCAA
CCTTATCACCAGAAATGGTTTCAGCGTCTGAAGTGATCAGCACGCAAGAAAACCAAACCTATACCTATGT
TCGCTGTTGGTATCGCACCAGCTACTCGAAAGATGATCCAGCGACCGATTGGGAATGGGCAAAAAACGAA
GATGGTAGCTACTTCACCATTGACGGCTACTGGTGGAGCTCCGTTTCATTTAAAAACATGTTCTACACCA
ACACGTCGCAAAACGTTATCCGTCAGCGTTGTGAAGCAACATTAGATTTGGCGAACGAGAACGCAGACAT
TACGTTCTTCGCCGCTGACAATCGCTTCTCATACAACCACACGATCTGGAGCAACGACGCAGCAATGCAG
CCAGATCAAATCAACAAAGTGGTTGCACTCGGTGACAGCTTGTCTGATACAGGCAACATCTTTAACGCAT
CACAATGGCGCTTCCCTAACCCGAACAGCTGGTTCTTAGGTCACTTCTCCAACGGTTTTGTTTGGACAGA
ATACATTGCCAAAGCGAAGAACCTTCCGCTCTACAACTGGGCAGTTGGCGGCGCGGCTGGTGAGAACCAA
TACATCGCGCTAACAGGGGTTGGTGAGCAAGTTTCTTCGTACTTAACCTACGCAAAACTGGCGAAGAACT
ACAAACCAGCAAACACCTTGTTTACGCTTGAGTTTGGTTTGAATGACTTCATGAACTACAACCGTGGCGT
TCCAGAAGTGAAAGCGGATTATGCAGAAGCACTGATTCGTTTGACGGACGCAGGTGCGAAGAACTTCATG
TTGATGACACTGCCAGATGCGACGAAAGCGCCTCAGTTTAAGTACTCAACACAAGAAGAGATCGACAAAA
TTCGTGCGAAAGTGCTTGAGATGAACGAGTTCATCAAGGCACAAGCGATGTACTACAAAGCGCAAGGTTA
CAACATCACGTTGTTTGATACTCACGCCTTGTTCGAGACGCTAACTTCTGCGCCAGAAGAGCACGGTTTC
GTGAACGCGAGCGATCCTTGTTTGGACATCAACCGCTCATCGTCTGTCGATTACATGTACACCCACGCAT
TGCGCTCTGAGTGTGCAGCGTCTGGTGCTGAGAAGTTTGTGTTCTGGGATGTCACGCATCCAACAACAGC
AACTCACCGCTATGTTGCAGAGAAAATGCTAGAAAGTAGCAACAACTTAGCCGAGTACCGTTTCTAA

>VIP4-0434
ATGATGAAAAAAACAATCACACTATTAACTGCATTACTCCCGCTTGCTTCTGCAGTTGCCGAAGAGCCAACCTTAT
CACCAGAAATGGTTTCAGCGTCTGAAGTGATCAGCACGCAAGAAAACCAAACCTATACCTATGTTCGCTGTTGGTA
TCGCACCAGCTACTCGAAAGATGATCCGGCGACCGATTGGGAATGGGCAAAAAACGAAGATGGTAGCTACTTCAC
CATTGACGGCTACTGGTGGAGCTCCGTTTCATTTAAAAACATGTTCTACACCAACACGTCGCAAAACGTTATCCGT
CAGCGTTGTGAAGCCACATTAGATTTGGCGAACGAGAACGCAGACATTACGTTCTTCGCCGCTGACAATCGCTTCT
CATACAACCACACGATCTGGAGCAACGACGCAGCAATGCAGCCAGATCAAATCAACAAAGTGGTTGCACTCGGTG
ACAGCTTGTCTGATACAGGCAACATCTTTAACGCATCACAATGGCGCTTCCCTAACCCGAACAGCTGGTTCTTAGG
TCACTTCTCCAACGGTTTTGTGTGGACAGAATACATTGCCAAAGCGAAGAACCTTCCGCTCTACAACTGGGCAGTT
GGCGGCGCGGCTGGTGAGAACCAATACATCGCGCTAACAGGGGTTGGTGATCAAGTTTCTTCGTACTTAACCTACG
CAAAACTGGCGAAGAACTACAAACCAGCAAACACCTTGTTTACGCTTGAGTTTGGTTTGAATGACTTCATGAACTA
CAACCGTGGCGTTCCAGAAGTGAAAGCAGATTATGCAGAAGCACTGATTCGTTTGACGGACGCAGGTGCAAAGAA
CTTCATGTTGATGACACTGCCAGACGCGACGAAAGCGCCTCAGTTTAAGTACTCAACACAAGAAGAGATCGACAA
AATTCGTGCGAAAGTGCTTGAGATGAACGAGTTCATCAAGGCACAAGCGATGTACTACAAAGCGCAAGGTTACAA
CATCACGTTGTTTGATACTCACGCCTTGTTCGAGACGCTAACTTCTGCGCCAGAAGAGCACGGTTTCGTGAACGCG
AGCGATCCTTGTTTGGACATCAACCGTTCATCGTCTGTCGATTACATGTACACCCACGCATTGCGCTCTGAGTGTGC
GGCGTCTGGTGCTGAGAAGTTTGTGTTCTGGGATGTCACGCATCCAACAACAGCAACTCACCGCTATGTTGCAGAG
AAAATGCTAGAAAGTAGCAACAACTTAGCCGAGTACCGTTTCTAA

>VIP4-0430
ATGATGAAAAAAACAATCACACTATTAACTGCATTACTCCCGCTTGCTTCTGCAGTTGCCGAAGAGCCAA
CCTTATCACCAGAAATGGTTTCAGCGTCTGAAGTGATCAGCACGCAAGAAAACCAAACCTATACCTATGT
TCGCTGTTGGTATCGCACCAGCTACTCGAAAGATGATCCGGCGACCGATTGGGAATGGGCAAAAAACGAA
GATGGTAGCTACTTCACCATTGACGGCTACTGGTGGAGCTCCGTTTCATTTAAAAACATGTTCTACACCA
ACACGTCGCAAAACGTTATCCGTCAGCGTTGTGAAGCAACATTAGATTTGGCGAACGAGAACGCAGACAT
TACGTTCTTCGCCGCTGACAATCGCTTCTCATACAACCACACGATCTGGAGCAACGACGCAGCAATGCAG
CCAGATCAAATCAACAAAGTGGTTGCACTCGGTGACAGCTTGTCTGATACAGGCAACATCTTTAACGCAT
CACAATGGCGCTTCCCTAACCCGAACAGCTGGTTCTTAGGTCACTTCTCCAACGGTTTTGTGTGGACAGA
ATACATTGCCAAAGCGAAGAACCTTCCGCTCTACAACTGGGCAGTTGGCGGCGCGGCTGGTGAGAACCAA
TACATCGCGCTAACAGGGGTTGGTGATCAAGTTTCTTCGTACTTAACCTACGCAAAACTGGCGAAGAACT
ACAAACCAGCAAACACCTTGTTTACGCTTGAGTTTGGTTTGAATGACTTCATGAACTACAACCGTGGCGT

TCCAGAAGTGAAAGCAGATTATGCAGAAGCACTGATTCGTTTGACGGACGCAGGTGCGAAGAACTTCATG
TTGATGACACTGCCAGACGCGACGAAAGCGCCTCAGTTTAAGTACTCAACACAAGAAGAGATCGACAAAA
TTCGTGCGAAAGTGCTTGAGATGAACGAGTTCATCAAGGCACAAGCGATGTACTACAAAGCGCAAGGTTA
CAACATCACGTTGTTTGATACTCACGCCTTGTTCGAGACGCTAACTTCTGCGCCAGAAGAGCACGGTTTC
GTGAACGCGAGTGATCCTTGTTTGGACATCAACCGCTCATCGTCTGTCGATTACATGTACACCCACGCAT
TGCGCTCTGAGTGTGCGGCGTCTGGTGCTGAGAAATTTGTGTTCTGGGATGTCACGCACCCAACAACAGC
AACTCACCGCTATGTTGCAGAGAAAATGCTAGAAAGTAGCAACAACTTAGCCGAGTACCGTTTCTAA

>VIP4-0444
ATGATGAAAAAAACAATCACACTATTAACTGCATTACTCCCGCTTGCTTCTGCAGTTGCCGAAGAGCCAA
CCTTATCACCAGAAATGGTTTCAGCGTCTGAAGTGATCAGCACGCAAGAAAACCAAACCTATACCTATGT
TCGCTGTTGGTATCGCACCAGCTACTCGAAAGATGATCCGGCGACCGATTGGGAATGGGCAAAAAACGAA
GATGGTAGCTACTTCGCCATTGACGGCTACTGGTGGAGCTCCGTTTCATTTAAAAACATGTTCTACACCA
ACACGTCGCAAAACGTTATCCGTCAGCGTTGTGAAGCAACATTAGATTTGGCGAACGAGAACGCAGACAT
TACGTTCTTCGCCGCTGACAATCGCTTCTCATACAACCACACGATCTGGAGCAACGACGCAGCAATGCAG
CCAGATCAAATCAACAAAGTGGTTGCACTCGGTGACAGCTTGTCTGATACAGGCAACATCTTTAACGCAT
CACAATGGCGCTTCCCTAACCCGAACAGCTGGTTCTTAGGTCACTTCTCCAACGGTTTTGTGTGGACAGA
ATACATTGCCAAAGCGAAGAACCTTCCGCTCTACAACTGGTCAGTTGGCGGCGCGGCTGGTGAGAACCAA
TACATCGCGCTAACAGGGGTTGGTGATCAAGTTTCTTCGTACTTAACCTACGCAAAACTGGCGAAGAACT
ACAAACCAGCAAACACCTTGTTTACGCTTGAGTTTGGTTTGAATGACTTCATGAACTACAACCGTGGCGT
TCCAGAAGTGAAAGCAGATTATGCAGAAGCACTGATTCGTTTGACGGACGCAGGTGCGAAGAACTTCATG
TTGATGACACTGCCAGACGCGACGAAAGCGCCTCAGTTTAAGTACTCAACACAAGAAGAGATCGACAAAA
TTCGTGCGAAAGTGCTTGAGATGAACGAGTTCATCAAGGCACAAGCGATGTACTACAAAGCGCAAGGTTA
CAACATCACGTTGTTTGATACTCACGCCTTGTTCGAGACGCTAACTTCTGCGCCAGAAGAGCACGGTTTC
GTGAACGCGAGCGATCCTTGTTTGGACATCAACCGCTCATCGTCTGTCGATTACATGTACACCCACGCAT
TGCGCTCTGAGTGTGCGGCGTCTGGTGCTGAGAAGTTTGTGTTCTGGGATGTCACGCACCCAACAACAGC
AACTCACCGCTATGTTGCAGAGAAAATGCTAGAAAGTAGCAACAACTTAGCCGAGTACCGTTTCTAA

>VIP4-0395
ATGATGAAAAAAACAATCACACTATTAACTGCATTACTCCCGCTTGCTTCTGCAGTTGCCGAAGAGCCAA
CCTTATCACCAGAAATGGTTTCAGCGTCTGAAGTGATCAGCACGCAAGAAAACCAAACCTATACCTATGT
TCGCTGTTGGTATCGCACCAGCTACTCGAAAGATGATCCAGCGACCGATTGGGAATGGGCAAAAAACGAA
GATGGTAGCTACTTCACCATTGACGGCTACTGGTGGAGCTCCGTTTCATTTAAAAACATGTTCTACACCA
ACACGTCGCAAAACGTTATCCGTCAGCGTTGTGAAGCAACATTAGATTTGGCGAACGAGAACGCAGACAT
TACGTTCTTCGCCGCTGACAATCGCTTCTCATACAACCACACGATCTGGAGCAACGACGCAGCAATGCAG
CCAGATCAAATCAACAAAGTGGTTGCACTCGGTGACAGCTTGTCTGATACAGGCAACATCTTTAACGCAT
CACAATGGCGCTTCCCTAACCCGAACAGCTGGTTCTTAGGTCACTTCTCCAACGGTTTTGTTTGGACAGA
ATACATTGCCAAAGCGAAGAACCTTCCGCTCTACAACTGGGCAGTTGGCGGCGCGGCTGGTGAGAACCAA
TACATCGCGCTAACAGGGGTTGGTGAGCAAGTTTCTTCGTACTTAACCTACGCAAAACTGGCGAAGAACT
ACAAACCAGCAAACACCTTGTTTACGCTTGAGTTTGGTTTGAATGACTTCATGAACTACAACCGTGGCGT
TCCAGAAGTGAAAGCGGATTATGCAGAAGCACTGATTCGTTTGACGGACGCAGGTGCGAAGAACTTCATG
TTGATGACACTGCCAGATGCGACGAAAGCGCCTCAGTTTAAGTACTCAACACAAGAAGAGATCGACAAAA
TTCGTGCGAAAGTGCTTGAGATGAACGAGTTCATCAAGGCACAAGCGATGTACTACAAAGCGCAAGGTTA
CAACATCACGTTGTTTGATACTCACGCCTTGTTCGAGACGCTAACTTCTGCGCCAGAAGAGCACGGTTTC
GTGAACGCGAGCGATCCTTGTTTGGACATCAACCGCTCATCGTCTGTCGATTACATGTACACCCACGCAT
TGCGCTCTGAGTGTGCAGCGTCTGGTGCTGAGAAGTTTGTGTTCTGGGATGTCACGCATCCAACAACAGC
AACTCACCGCTATGTTGCAGAGAAAATGCTAGAAAGTAGCAACAACTTAGCCGAGTACCGTTTCTAA

>VIP4-0219

ATGATGAAAAAAACAATCACACTATTAACTGCATTACTCCCGCTTGCTTCTGCAGTTGCCGAAGAGCCAA
CCTTATCACCAGAAATGGTTTCAGCGTCTGAAGTGATCAGCACGCAAGAAAACCAAACCTATACCTATGT
TCGCTGTTGGTATCGCACCAGCTACTCGAAAGATGATCCGGCGACCGATTGGGAATGGGCAAAAAACGAA
GATGGTAGCTACTTCACCATTGACGGCTACTGGTGGAGCTCCGTTTCATTTAAAAACATGTTCTACACCA
ACACGTCGCAAAACGTTATCCGTCAGCGTTGTGAAGCAACATTAGATTTGACGAACGAGAACGCAGACAT
TACGTTCTTCGCCGCTGACAATCGCTTCTCATACAACCACACGATCTGGAGCAACGACGCAGCAATGCAG
CCAGATCAAATCAACAAAGTGGTTGCACTCGGTGACAGCTTGTCTGATACAGGCAACATCTTTAACGCAT
CACAATGGCGCTTCCCTAACCCGAACAGCTGGTTCTTAGGTCACTTCTCCAACGGTTTTGTGTGGACAGA
ATACATTGCCAAAGCGAAGAACCTTCCGCTCTACAACTGGGCAGTTGGCGGCGCGGCTGGTGAGAACCAA
TACATCGCGCTAACAGGGGTTGGTGAGCAAGTTTCTTCGTACTTAACCTACGCAAAACTGGCGAAGAACT
ACAAACCAGCAAACACCTTGTTTACGCTTGAGTTTGGTTTGAATGACTTCATGAACTACAACCGTGGCGT
TCCAGAAGTGAAAGCAGATTATGCAGAAGCACTGATTCGTTTGACGGACGCAGGTGCGAAGAACTTCATG
TTGATGACACTGCCAGACGCGACGAAAGCGCCTCAGTTTAAGTACTCAACACAAGAAGAGATCGACAAAA
TTCGTGCGAAAGTGCTTGAGATGAACGAGTTCATCAAGGCACAAGCGATGTACTACAAAGCGCAAGGTTA
CAACATCACGTTGTTTGATACTCACGCCTTGTTCGAGACGCTAACTTCTGCGCCCGAAGAGCACGGTTTC
GTGAACGCGAGTGATCCTTGTTTGGACATCAACCGCTCATCGTCTGTCGATTACATGTACACCCACGCAT
TGCGCTCTGAGTGTGCTGCGTCTGGTGCTGAGAAGTTTGTGTTCTGGGATGTCACGCATCCAACAACAGC
AACTCACCGCTATGTTGCAGAGAAAATGCTAGAAAGTAGCAACAACTTAGCAGAGTACCGTTTCTAA

>VIP4-0447
ATGATGAAAAAAACAATCACACTATTAACTGCATTACTCCCGCTTGCTTCTGCAGTTGCCGAAGAGCCAA
CCTTATCACCAGAAATGGTTTCAGCGTCTGAAGTGATCAGCACGCAAGAAAACCAAACCTATACCTATGT
TCGCTGTTGGTATCGCACCAGCTACTCGAAAGATGATCCGGCGACCGATTGGGAATGGGCAAAAAACGAA
GATGGTAGCTACTTCACCATTGACGGCTACTGGTGGAGCTCCGTTTCATTTAAAAACATGTTCTACACCA
ACACGTCGCAAAACGTTATCCGTCAGCGTTGTGAAGCAACATTAGATTTGGCGAACGAGAACGCAGACAT
TACGTTCTTCGCCGCTGACAATCGCTTCTCATACAACCACACGATCTGGAGCAACGACGCAGCAATGCAG
CCAGATCAAATCAACAAAGTGGTTGCACTCGGTGACAGCTTGTCTGATACCGGCAACATCTTTAACGCAT
CACAATGGCGCTTCCCTAACCCGAACAGCTGGTTCTTAGGTCACTTCTCCAACGGTTTTGTGTGGACAGA
ATACATTGCCAAAGCGAAGAACCTTCCGCTCTACAACTGGGCAGTTGGCGGCGCGGCTGGTGAGAACCAA
TACATCGCGCTAACAGGGGTTGGTGATCAAGTTTCTTCGTACTTAACCTACGCAAAACTGGCGAAGAACT
ACAAACCAGCAAACACCTTGTTTACGCTTGAGTTTGGTTTGAATGACTTCATGAACTACAACCGTGGTGT
TCCAGAAGTGAAAGCGGATTATGCAGAAGCACTGATTCGTTTGACGGACGCAGGTGCGAAGAACTTCATG
TTGATGACACTGCCAGACGCGACGAAAGCGCCTCAGTTTAAGTACTCAACACAAGAAGAGATCGACAAAA
TTCGTGCGAAAGTGCTTGAGATGAACGAGTTCATCAAGGCACAAGCGATGTACTACAAAGCGCAAGGTTA
CAACATCACGTTGTTTGATACTCACGCCTTGTTCGAGACGCTAACTTCTGCGCCAGAAGAGCACGGTTTC
GTGAACGCGAGCGATCCTTGTTTGGACATCAACCGCTCATCGTCTGTCGATTACATGTACACCCACGCAT
TGCGCTCTGAGTGTGCAGCGTCTGGTGCTGAGAAGTTTGTGTTCTGGGATGTCACGCATCCAACAACAGC
AACTCACCGCTATGTTGCAGAGAAAATGCTAGAAAGTAGCAACAACTTAGCCGAGTACCGTTTCTAA

>M0605
ATGATGAAAAAAACAATCACACTATTAACTGCATTACTCCCGCTTGCTTCTGCAGTTGCCGAAGAGCCAA
CCTTATCACCAGAAATGGTTTCAGCGTCTGAAGTGATCAGCACGCAAGAAAACCAAACCTATACCTATGT
TCGCTGTTGGTATCGCACCAGCTACTCGAAAGATGATCCGGCGACCGATTGGGAATGGGCAAAAAACGAA
GATGGTAGCTACTTCACCATTGACGGCTACTGGTGGAGCTCCGTTTCATTTAAAAACATGTTCTACACCA
ACACGTCGCAAAACGTTATCCGTCAGCGTTGTGAAGCAACATTAGATTTGGCGAACGAGAACGCAGACAT
TACGTTCTTCGCCGCTGACAATCGCTTCTCATACAACCACACGATCTGGAGCAACGACGCAGCAATGCAG
CCAGATCAAATCAACAAAGTGGTTGCTCTCGGTGACAGCTTGTCTGATACAGGCAACATCTTTAACGCAT
CACAATGGCGCTTCCCTAACCCGAACAGCTGGTTCTTAGGTCACTTCTCCAACGGTTTTGTGTGGACAGA
ATACATTGCCAAAGCGAAGAACCTTCCGCTCTACAACTGGGCAGTTGGCGGCGCGGCTGGTGAGAACCAA
TACATCGCGCTAACAGGGTTGGTGAGCAAGTTTCTTCGTACTTAACCTACGCAAAACTGGCGAAGAACTA
CAAACCAGCAAACACCTTGTTTACGCTTGAGTTTGGTTTGAATGACTTCATGAACTACAACCGTGGCGTT

CCAGAAGTGAAAGCGGATTATGCAGAAGCACTGATTCGTTTGACGGACGCAGGTGCGAAGAACTTCATGT
TGATGACACTGCCAGACGCGACGAAAGCGCCTCAGTTTAAGTACTCAACACAAGAAGAGAGCGACAAAAT
TCGTGCGAAAGTGCTTGAGATGAACGAGTTCATCAAGGCACAAGCGATGTACTACAAAGCGCAAGGTTAC
AACATCACGTTGTTTGATACTCACGCCTTGTTCGAGACGCTAACTTCTGCGCCAGAAGAGCACGGTTTCG
TGAACGCGAGTGATCCTTGTTTGGACATCAACCGCTCATCGTCTGTCGATTACATGTACACCCACGCATT
GCGCTCTGAGTGTGCAGCGTCTGGTGCTGAGAAGTTTGTGTTCTGGGATGTCACGCATCCAACAACAGCA
ACTCACCGCTATGTTGCAGAGAAAATGCTAGAAAGTAGCAACAACTTAGCAGAGTACCGTTTCTAA

>TUMSAT_DE1_S1
ATGATGAAAAAAACAATCACACTATTAACTGCATTACTCCCGCTTGCTTCTGCAGTTGCCGAAGAGCCAACCTTAT
CACCAGAAATGGTTTCAGCGTCTGAAGTGATCAGCACGCAAGAAAACCAAACCTATACCTATGTTCGCTGTTGGTA
TCGCACCAGCTACTCGAAAGATGATCCGGCGACCGATTGGGAATGGGCAAAAAACGAAGATGGTAGCTACTTCAC
CATTGACGGCTACTGGTGGAGCTCCGTTTCATTTAAAAACATGTTCTACACCAACACGTCGCAAAACGTTATCCGT
CAGCGTTGTGAAGCAACATTAGATTTGGCGAACGAGAACGCAGACATTACGTTCTTCGCCGCTGACAATCGCTTCT
CATACAACCACACGATCTGGAGCAACGACGCAGCAATGCAGCCAGATCAAATCAACAAAGTGGTTGCACTCGGTG
ACAGCTTGTCTGATACAGGCAACATCTTTAACGCATCACAATGGCGCTTCCCTAACCCGAACAGCTGGTTCTTAGG
TCACTTCTCCAACGGTTTTGTGTGGACAGAATACATTGCCAAAGCGAAGAACCTTCCGCTCTACAACTGGGCAGTT
GGCGGCGCGGCTGGTGAGAACCAATACATCGCGCTAACAGGGGTTGGTGATCAAGTTTCTTCGTACTTAACCTACG
CAAAACTGGCGAAGAACTACAAACCAGCAAACACCTTGTTTACGCTTGAGTTTGGTTTGAATGACTTCATGAACTA
CAACCGTGGCGTTCCAGAAGTGAAAGCGGATTATGCAGAAGCACTGATTCGTTTGACGGACGCAGGTGCGAAGAA
CTTCATGTTGATGACACTGCCAGACGCGACGAAAGCGCCTCAGTTTAAGTACTCAACACAAGAAGAGATCGACAA
AATTCGTGCGAAAGTGCTTGAGATGAACGAGTTCATCAAGGCACAAGCGATGTACTACAAAGCGCAAGGTTACAA
CATCACGTTGTTTGATACTCACGCCTTGTTCGAGACGCTAACTTCTGCGCCAGAAGAGCACGGTTTCGTGAACGCG
AGTGATCCTTGTTTGGACATCAACCGCTCATCGTCTGTCGATTACATGTACACCCACGCATTGCGCTCTGAGTGTGC
GGCGTCTGGTGCTGAGAAATTTGTGTTCTGGGATGTCACGCACCCAACAACAGCAACTCACCGCTATGTTGCAGAG
AAAATGCTAGAAAGTAGCAACAACTTAGCCGAGTACCGTTTCTAA

>TUMSAT_DE2_S2
ATGATGAAAAAAACAATCACACTATTAACTGCATTACTCCCGCTTGCTTCTGCAGTTGCCGAAGAGCCAACCTTAT
CACCAGAAATGGTTTCAGCGTCTGAAGTGATCAGCACGCAAGAAAACCAAACCTATACCTATGTTCGCTGTTGGTA
TCGCACCAGCTACTCGAAAGATGATCCGGCGACCGATTGGGAATGGGCAAAAAACGAAGATGGTAGCTACTTCAC
CATTGACGGTTACTGGTGGAGCTCCGTTTCATTTAAAAACATGTTCTACACCAACACGTCGCAAAACGTTATCCGT
CAGCGTTGTGAAGCCACATTAGATTTGGCGAACGAGAACGCAGACATTACGTTCTTCGCCGCTGACAATCGCTTCT
CATACAACCACACGATCTGGAGCAACGACGCAGCAATGCAGCCAGATCAAATCAACAAAGTGGTTGCACTCGGTG
ACAGCTTGTCTGATACAGGCAACATCTTTAACGCATCACAATGGCGCTTCCCTAACCCGAACAGCTGGTTCTTAGG
TCACTTCTCCAACGGTTTTGTGTGGACAGAATACATTGCCAAAGCGAAGAACCTTCCGCTCTACAACTGGGCAGTT
GGCGGCGCGGCTGGTGAGAACCAATACATCGCGCTAACAGGGGTTGGTGAGCAAGTTTCTTCGTACTTAACCTACG
CAAAACTGGCGAAGAACTACAAACCAGCAAACACCTTGTTTACGCTTGAGTTTGGTTTGAATGACTTCATGAACTA
CAACCGTGGCGTTCCAGAAGTGAAAGCAGATTATGCAGAAGCACTGATTCGTTTGACGGACGCAGGTGCGAAGAA
CTTCATGTTGATGACACTGCCAGACGCGACGAAAGCGCCTCAGTTTAAGTACTCAACACAAGAAGAGATCGACAA
AATTCGTGCGAAAGTGCTTGAGATGAACGAGTTCATCAAGGCACAAGCGATGTACTACAAAGCGCAAGGTTACAA
CATCACGTTGTTTGATACTCACGCCTTGTTCGAGACGCTAACTTCTGCGCCCGAAGAGCACGGTTTCGTGAACGCG
AGTGATCCTTGTTTGGACATCAACCGCTCATCGTCTGTCGATTACATGTACACCCACGCATTGCGCTCTGAGTGTGC
GGCGTCTGGTGCTGAGAAGTTTGTGTTCTGGGATGTCACGCACCCAACAACAGCAACTCACCGCTATGTTGCAGAG
AAAATGCTAGAAAGTAGCAACAACTTAGCCGAGTACCGTTTCTAA

>TUMSAT_D06_S3
ATGATGAAAAAAACAATCACACTATTAACTGCATTACTCCCGCTTGCTTCTGCAGTTGCCGAAGAGCCAA
CCTTATCACCAGAAATGGTTTCAGCGTCTGAAGTGATCAGCACGCAAGAAAACCAAACCTATACCTATGT
TCGCTGTTGGTATCGCACCAGCTACTCGAAAGATGATCCGGCGACCGATTGGGAATGGGCAAAAAACGAA
GATGGTAGCTACTTCACCATTGACGGCTACTGGTGGAGCTCCGTTTCATTTAAAAACATGTTCTACACCA

ACACGTCGCAAAACGTTATCCGTCAGCGTTGTGAAGCAACATTAGATTTGGCGAACGAGAACGCAGACAT
TACGTTCTTCGCCGCTGACAATCGCTTCTCATACAACCACACGATCTGGAGCAACGACGCAGCAATGCAG
CCAGATCAAATCAACAAAGTGGTTGCACTCGGTGACAGCTTGTCTGATACAGGCAACATCTTTAACGCAT
CACAATGGCGCTTCCCTAACCCGAACAGCTGGTTCTTAGGTCACTTCTCCAACGGTTTTGTGTGGACAGA
ATACATTGCCAAAGCGAAGAACCTTCCGCTCTACAACTGGGCAGTTGGCGGCGCGGCTGGTGAGAACCAA
TACATCGCGCTAACAGGGGTTGGTGAGCAAGTTTCTTCGTACTTAACCTACGCAAAACTGGCGAAGAACT
ACAAACCAGCAAACACCTTGTTTACGCTTGAGTTTGGTTTGAATGACTTCATGAACTACAACCGTGGCGT
TCCAGAAGTGAAAGCAGATTATGCAGAAGCACTGATTCGTTTGACGGACGCAGGTGCGAAGAACTTCATG
TTGATGACACTGCCAGACGCGACGAAAGCGCCTCAGTTTAAGTACTCAACACAAGAAGAGATCGACAAAA
TTCGTGCGAAAGTGCTTGAGATGAACGAGTTCATCAAGGCACAAGCGATGTACTACAAAGCGCAAGGTTA
CAACATCACGTTGTTTGATACTCACGCCTTGTTCGAGACGCTAACTTCTGCGCCCGAAGAGCACGGTTTC
GTGAACGCGAGTGATCCTTGTTTGGACATCAACCGCTCATCGTCTGTCGATTACATGTACACCCACGCAT
TGCGCTCTGAGTGTGCGGCGTCTGGTGCTGAGAAGTTTGTGTTCTGGGATGTCACGCATCCAACAACAGC
AACTCACCGCTATGTTGCAGAGAAAATGCTAGAAAGTAGCAACAACTTAGCAGAGTACCGTTTCTAA

>TUMSAT_H01_S4
ATGATGAAAAAAACAATCACACTATTAACTGCATTACTCCCGCTTGCTTCTGCAGTTGCCGAAGAGCCAACCTTAT
CACCAGAAATGGTTTCAGCGTCTGAAGTGATCAGCACGCAAGAAAACCAAACCTATACCTATGTTCGCTGTTGGTA
TCGCACCAGCTACTCGAAAGATGATCCGGCGACCGATTGGGAATGGGCAAAAAACGAAGATGGTAGCTACTTCAC
CATTGACGGCTACTGGTGGAGCTCCGTTTCATTTAAAAACATGTTCTACACCAACACGTCGCAAAACGTTATCCGT
CAGCGTTGTGAAGCAACATTAGATTTGGCGAACGAGAACGCAGACATTACGTTCTTCGCCGCTGACAATCGCTTCT
CATACAACCACACGATCTGGAGCAACGACGCAGCAATGCAGCCAGATCAAATCAACAAAGTGGTTGCACTCGGTG
ACAGCTTGTCTGATACAGGCAACATCTTTAACGCATCACAATGGCGCTTCCCTAACCCGAACAGCTGGTTCTTAGG
TCACTTCTCCAACGGTTTTGTGTGGACAGAATACATTGCCAAAGCGAAGAACCTTCCGCTCTACAACTGGGCAGTT
GGCGGCGCGGCTGGTGAGAACCAATACATCGCGCTAACAGGGGTTGGTGATCAAGTTTCTTCGTACTTAACCTACG
CAAAACTGGCGAAGAACTACAAACCAGCAAACACCTTGTTTACGCTTGAGTTTGGTTTGAATGACTTCATGAACTA
CAACCGTGGCGTTCCAGAAGTGAAAGCGGATTATGCAGAAGCACTGATTCGTTTGACGGACGCAGGTGCGAAGAA
CTTCATGTTGATGACACTGCCAGACGCGACGAAAGCGCCTCAGTTTAAGTACTCAACACAAGAAGAGATCGACAA
AATTCGTGCGAAAGTGCTTGAGATGAACGAGTTCATCAAGGCACAAGCGATGTACTACAAAGCGCAAGGTTACAA
CATCACGTTGTTTGATACTCACGCCTTGTTCGAGACGCTAACTTCTGCGCCAGAAGAGCACGGTTTCGTGAACGCG
AGCGATCCTTGTTTGGACATCAACCGCTCATCGTCTGTCGATTACATGTACACCCACGCATTGCGCTCTGAGTGTGC
AGCGTCTGGTGCTGAGAAGTTTGTATTCTGGGATGTCACGCACCCAACAACAGCCACGCATCGTTATGTTGCTGAA
AAAATGTTGGAAAGCAGCAATAACTTAGAAGAGTTTCGCTTTTAA

>TUMSAT_H10_S6
ATGATGAAAAAAACAATCACACTATTAACTGCATTACTCCCGCTTGCTTCTGCAGTTGCCGAAGAGCCAACCTTAT
CACCAGAAATGGTTTCAGCGTCTGAAGTGATCAGCACGCAAGAAAACCAAACCTATACCTATGTTCGCTGTTGGTA
TCGCACCAGCTACTCGAAAGATGATCCGGCGACCGATTGGGAATGGGCAAAAAACGAAGATGGTAGCTACTTCAC
CATTGACGGCTACTGGTGGAGCTCCGTTTCATTTAAAAACATGTTCTACACCAACACGTCGCAAAACGTTATCCGT
CAGCGTTGTGAAGCCACATTAGATTTGGCGAACGAGAACGCAGACATTACGTTCTTCGCCGCTGACAATCGCTTCT
CATACAACCACACGATCTGGAGCAACGACGCAGCAATGCAGCCAGATCAAATCAACAAAGTGGTTGCACTCGGTG
ACAGCTTGTCTGATACAGGCAACATCTTTAACGCATCACAATGGCGCTTCCCTAACCCGAACAGCTGGTTCTTAGG
TCACTTCTCCAACGGTTTTGTGTGGACAGAATACATTGCCAAAGCGAAGAACCTTCCGCTCTACAACTGGGCAGTT
GGCGGCGCGGCTGGTGAGAACCAATACATCGCGCTAACAGGGGTTGGTGAGCAAGTTTCTTCGTACTTAACCTACG
CAAAACTGGCGAAGAACTACAAACCAGCAAACACCTTGTTTACGCTTGAGTTTGGTTTGAATGACTTCATGAACTA
CAACCGTGGCGTTCCAGAAGTGAAAGCAGATTATGCAGAAGCACTGATTCGTTTGACGGACGCAGGTGCGAAGAA
CTTCATGTTGATGACACTGCCAGACGCGACGAAAGCGCCTCAGTTTAAGTACTCAACACAAGAAGAGATCGACAA
AATTCGTGCGAAAGTGCTTGAGATGAACGAGTTCATCAAGGCACAAGCGATGTACTACAAAGCGCAAGGTTACAA
CATCACGTTGTTTGATACTCACGCCTTGTTCGAGACGCTAACTTCTGCGCCAGAAGAGCACGGTTTCGTGAACGCG

AGCGATCCTTGTTTGGACATCAACCGCTCATCGTCTGTCGACTACATGTACACCCACGCATTGCGCTCTGAGTGTGC
GGCGTCTGGTGCTGAGAAGTTTGTGTTCTGGGATGTCACGCATCCAACAACAGCAACTCACCGCTATGTTGCAGAG
AAAATGCTAGAAAGTAGCAACAACTTAGCAGAGTACCGCTTCTAA

>VP49
ATGATGAAAAAAACAATCACACTATTAACTGCATTACTCCCGCTTGCTTCTGCAGTTGCCGAAGAGCCAACCTTAT
CACCAGAAATGGTTTCAGCGTCTGAAGTGATCAGCACGCAAGAAAACCAAACCTATACCTATGTTCGCTGTTGGTA
TCGCACCAGCTACTCGAAAGATGATCCGGCGACCGATTGGGAATGGGCAAAAAACGAAGATGGTAGCTACTTCAC
CATTGACGGCTACTGGTGGAGCTCCGTTTCATTTAAAAACATGTTCTACACCAACACGTCGCAAAACGTTATCCGT
CAGCGTTGTGAAGCCACATTAGATTTGGCGAACGAGAACGCAGACATTACGTTCTTCGCCGCTGACAATCGCTTCT
CATACAACCACACGATCTGGAGCAACGACGCAGCAATGCAGCCAGATCAAATCAACAAAGTGGTTGCACTCGGTG
ACAGCTTGTCTGATACAGGCAACATCTTTAACGCATCACAATGGCGCTTCCCTAACCCGAATAGCTGGTTCTTAGG
TCACTTCTCCAACGGTTTTGTGTGGACAGAATACATTGCCAAAGCGAAGAACCTTCCACTCTACAACTGGGCAGTT
GGCGGCGCGGCTGGTGAGAACCAATACATCGCGCTAACAGGTTGGTGATCAAGTTTCTTCGTACTTAACCTACGCA
AAACTGGCGAAGAACTACAAACCAGCAAACACCTTGTTTACGCTTGAGTTTGGTTTGAATGACTTCATGAACTACA
ACCGTGGCGTTCCAGAAGTGAAAGCAGATTATGCAGAAGCACTGATTCGTTTGACGGACGCAGGTGCGAAGAACT
TCATGTTGATGACACTGCCAGACGCGACGAAAGCGCCTCAGTTTAAGTACTCAACACAAGAAGAGATCGACAAAA
TTCGTGCGAAAGTGCTTGAGATGAACGAGTTCATCAAGGCACAAGCGATGTACTACAAAGCGCAAGGTTACAACA
TAACGTTGTTTGATACTCACGCCTTGTTCGAGACGCTAACTTCTGCGCCCGAAGAGCACGGTTTCGTGAACGCGAG
CGATCCTTGTTTGGACATCAACCGCTCATCGTCTGTCGATTACATGTACACCCACGCATTGCGCTCTGAGTGTGCAG
CGTCTGGTGCTGAGAAGTTTGTGTTCTGGGATGTCACGCACCCAACAACAGCAACTCACCGCTATGTTGCAGAGAA
AATGCTAGAAAGTAGCAACAACTTAGCCGAGTACCGTTTCTAA

>CFSAN001595
ATGATGAAAAAAACAATCACACTATTAACTGCATTACTCCCGCTTGCTTCTGCAGTTGCCGAAGAGCCAACCTTAT
CACCAGAAATGGTTTCAGCGTCTGAAGTGATCAGCACGCAAGAAAACCAAACCTATACCTATGTTCGCTGTTGGTA
TCGCACCAGCTACTCGAAAGATGATCCGGCGACCGATTGGGAATGGGCAAAAAACGAAGATGGTAGCTACTTCAC
CATTGACGGCTACTGGTGGAGCTCCGTTTCATTTAAAAACATGTTCTACACCAACACGTCGCAAAACGTTATCCGT
CAGCGTTGTGAAGCCACATTAGATTTGGCGAACGAGAACGCAGACATTACGTTCTTCGCCGCTGACAATCGCTTCT
CATACAACCACACGATCTGGAGCAACGACGCAGCAATGCAGCCAGATCAAATCAACAAAGTGGTTGCACTCGGTG
ACAGCTTGTCTGATACAGGCAACATCTTTAACGCATCACAATGGCGCTTCCCTAACCCGAACAGCTGGTTCTTAGG
TCACTTCTCCAACGGTTTTGTGTGGACAGAATACATTGCCAAAGCGAAGAACCTTCCGCTCTACAACTGGGCAGTT
GGCGGCGCGGCTGGTGAGAACCAATACATCGCGCTAACAGGGGTTGGTGAGCAAGTTTCTTCGTACTTAACCTACG
CAAAACTGGCGAAGAACTACAAACCAGCAAACACCTTGTTTACGCTTGAGTTTGGTTTGAATGACTTCATGAACTA
CAACCGTGGCGTTCCAGAAGTGAAAGCAGATTATGCAGAAGCACTGATTCGTTTGACGGACGCAGGTGCGAAGAA
CTTCATGTTGATGACACTGCCAGACGCGACGAAAGCGCCTCAGTTTAAGTACTCAACACAAGAAGAGATCGACAA
AATTCGTGCGAAAGTGCTTGAGATGAACGAGTTCATCAAGGCTCAAGCGATGTACTACAAAGCGCAAGGTTACAA
CATCACGTTGTTTGATACTCACGCCTTGTTCGAGACGCTAACTTCTGCGCCAGAAGAGCACGGTTTCGTGAACGCG
AGCGATCCTTGTTTGGACATCAACCGCTCATCGTCTGTCGACTACATGTACACCCACGCATTGCGCTCTGAGTGTGC
GGCGTCTGGTGCTGAGAAGTTTGTGTTCTGGGATGTCACGCACCCAACAACAGCAACTCACCGCTATGTTGCAGAG
AAAATGCTAGAAAGTAGCAACAACTTAGCCGAGTACCGTTTCTAA

>CFSAN001612
ATGATGAAAAAAACAATCACACTATTAACTGCATTACTCCCGCTTGCTTCTGCAGTTGCCGAAGAGCCAACCTTAT
CACCAGAAATGGTTTCAGCGTCTGAAGTGATCAGCACGCAAGAAAACCAAACCTATACCTATGTTCGCTGTTGGTA
TCGCACCAGCTACTCGAAAGATGATCCGGCGACCGATTGGGAATGGGCAAAAAACGAAGATGGTAGCTACTTCAC
CATTGACGGCTACTGGTGGAGCTCCGTTTCACTTAAAAACATGTTCTACACCAACACGTCGCAAAACGTTATCCGT
CAGCGTTGTGAAGCAACATTAGATTTGGCGAACGAGAACGCAGACATTACGTTCTTCGCCGCTGACAATCGCTTCT
CATACAACCACACGATCTGGAGCAACGACGCAGCAATGCAGCCAGATCAAATCAACAAAGTGGTTGCACTCGGTG
ACAGCTTGTCTGATACAGGCAACATCTTTAACGCATCACAATGGCGCTTCCCTAACCCGAACAGCTGGTTCTTAGG
TCACTTCTCCAACGGTTTTGTGTGGACAGAATACATTGCCAAAGCGAAGAACCTTCCGCTCTACAACTGGGCAGTT

GGCGGCGCGGCTGGTGAGAACCAATACATCGCGCTAACAGGGGTTGGTGATCAAGTTTCTTCGTACTTAACCTACG
CAAAACTGGCGAAGAACTACAAACCAGCAAACACCTTGTTTACGCTTGAGTTTGGTTTGAATGACTTCATGAACTA
CAACCGTGGCGTTCCAGAAGTGAAAGCAGATTATGCAGAAGCACTGATTCGTTTGACGGACGCAGGTGCGAAGAA
CTTCATGTTGATGACACTGCCAGACGCGACGAAAGCGCCTCAGTTTAAGTACTCAACACAAGAAGAGATCGACAA
AATTCGTGCGAAAGTGCTTGAGATGAACGAGTTCATCAAGGCACAAGCGATGTACTACAAAGCGCAAGGTTACAA
CATCACGTTGTTTGATACTCACGCCTTGTTCGAGACGCTAACTTCTGCGCCAGAAGAGCACGGTTTCGTGAACGCG
AGCGATCCTTGTTTGGACATCAACCGCTCATCGTCTGTCGATTACATGTACACCCACGCATTGCGCTCTGAGTGTGC
AGCGTCTGGTGCTGAGAAGTTTGTGTTCTGGGATGTCACGCATCCAACAACAGCAACTCACCGCTATGTTGCAGAG
AAAATGCTAGAAAGTAGCAACAACTTAGCCGAGTACCGTTTCTAA

>CFSAN006134
ATGATGAAAAAAACAATCACACTATTAACTGCATTACTCCCGCTTGCTTCTGCAGTTGCCGAAGAGCCAACCTTAT
CACCAGAAATGGTTTCAGCGTCTGAAGTGATCAGCACGCAAGAAAACCAAACCTATACCTATGTTCGCTGTTGGTA
TCGCACCAGCTACTCGAAAGATGATCCGGCGACCGATTGGGAATGGGCAAAAAACGAAGATGGTAGCTACTTCAC
CATTGACGGCTACTGGTGGAGCTCCGTTTCACTTAAAAACATGTTCTACACCAACACGTCGCAAAACGTTATCCGT
CAGCGTTGTGAAGCAACATTAGATTTGGCGAACGAGAACGCAGACATTACGTTCTTCGCCGCTGACAATCGCTTCT
CATACAACCACACGATCTGGAGCAACGACGCAGCAATGCAGCCAGATCAAATCAACAAAGTGGTTGCACTCGGTG
ACAGCTTGTCTGATACAGGCAACATCTTTAACGCATCACAATGGCGCTTCCCTAACCCGAACAGCTGGTTCTTAGG
TCACTTCTCCAACGGTTTTGTGTGGACAGAATACATTGCCAAAGCGAAGAACCTTCCGCTCTACAACTGGGCAGTT
GGCGGCGCGGCTGGTGAGAACCAATACATCGCGCTAACAGGGTTGGTGATCAAGTTTCTTCGTACTTAACCTACGC
AAAACTGGCGAAGAACTACAAACCAGCAAACACCTTGTTTACGCTTGAGTTTGGTTTGAATGACTTCATGAACTAC
AACCGTGGCGTTCCAGAAGTGAAAGCAGATTATGCAGAAGCACTGATTCGTTTGACGGACGCAGGTGCGAAGAAC
TTCATGTTGATGACACTGCCAGACGCGACGAAAGCGCCTCAGTTTAAGTACTCAACACAAGAAGAGATCGACAAA
ATTCGTGCGAAAGTGCTTGAGATGAACGAGTTCATCAAGGCACAAGCGATGTACTACAAAGCGCAAGGTTACAAC
ATCACGTTGTTTGATACTCACGCCTTGTTCGAGACGCTAACTTCTGCGCCAGAAGAGCACGGTTTCGTGAACGCGA
GCGATCCTTGTTTGGACATCAACCGCTCATCGTCTGTCGATTACATGTACACCCACGCATTGCGCTCTGAGTGTGCA
GCGTCTGGTGCTGAGAAGTTTGTGTTCTGGGATGTCACGCATCCAACAACAGCAACTCACCGCTATGTTGCAGAGA
AAATGCTAGAAAGTAGCAACAACTTAGCCGAGTACCGTTTCTAA

>CFSAN006135
ATGATGAAAAAAACAATCACACTATTAACTGCATTACTCCCGCTTGCTTCTGCAGTTGCCGAAGAGCCAA
CCTTATCACCAGAAATGGTTTCAGCGTCTGAAGTGATCAGCACGCAAGAAAACCAAACCTATACCTATGT
TCGCTGTTGGTATCGCACCAGCTACTCGAAAGATGATCCGGCGACCGATTGGGAATGGGCAAAAAACGAA
GATGGTAGCTACTTCACCATTGACGGCTACTGGTGGAGCTCCGTTTCACTTAAAAACATGTTCTACACCA
ACACGTCGCAAAACGTTATCCGTCAGCGTTGTGAAGCAACATTAGATTTGGCGAACGAGAACGCAGACAT
TACGTTCTTCGCCGCTGACAATCGCTTCTCATACAACCACACGATCTGGAGCAACGACGCAGCAATGCAG
CCAGATCAAATCAACAAAGTGGTTGCACTCGGTGACAGCTTGTCTGATACAGGCAACATCTTTAACGCAT
CACAATGGCGCTTCCCTAACCCGAACAGCTGGTTCTTAGGTCACTTCTCCAACGGTTTTGTGTGGACAGA
ATACATTGCCAAAGCGAAGAACCTTCCGCTCTACAACTGGGCAGTTGGCGGCGCGGCTGGTGAGAACCAA
TACATCGCGCTAACAGGGGTTGGTGATCAAGTTTCTTCGTACTTAACCTACGCAAAACTGGCGAAGAACT
ACAAACCAGCAAACACCTTGTTTACGCTTGAGTTTGGTTTGAATGACTTCATGAACTACAACCGTGGCGT
TCCAGAAGTGAAAGCAGATTATGCAGAAGCACTGATTCGTTTGACGGACGCAGGTGCGAAGAACTTCATG
TTGATGACACTGCCAGACGCGACGAAAGCGCCTCAGTTTAAGTACTCAACACAAGAAGAGATCGACAAAA
TTCGTGCGAAAGTGCTTGAGATGAACGAGTTCATCAAGGCACAAGCGATGTACTACAAAGCGCAAGGTTA
CAACATCACGTTGTTTGATACTCACGCCTTGTTCGAGACGCTAACTTCTGCGCCAGAAGAGCACGGTTTC
GTGAACGCGAGCGATCCTTGTTTGGACATCAACCGCTCATCGTCTGTCGATTACATGTACACCCACGCAT
TGCGCTCTGAGTGTGCAGCGTCTGGTGCTGAGAAGTTTGTGTTCTGGGATGTCACGCATCCAACAACAGC
AACTCACCGCTATGTTGCAGAGAAAATGCTAGAAAGTAGCAACAACTTAGCCGAGTACCGTTTCTAA

>CFSAN007436

ATGATGAAAAAAACAATCACACTATTAACTGCATTACTCCCGCTTGCTTCTGCAGTTGCCGAAGAGCCAACCTTAT
CACCAGAAATGGTTTCAGCGTCTGAAGTGATCAGCACGCAAGAAAACCAAACCTATACCTATGTTCGCTGTTGGTA
TCGCACCAGCTACTCGAAAGATGATCCGGCGACCGATTGGGAATGGGCAAAAAACGAAGATGGTAGCTACTTCAC
CATTGACGGCTACTGGTGGAGCTCCGTTTCATTTAAAAACATGTTCTACACCAACACGTCGCAAAACGTTATCCGT
CAGCGTTGTGAAGCAACATTAGATTTGGCGAACGAGAACGCAGACATTACGTTCTTCGCCGCTGACAATCGCTTCT
CATACAACCACACGATCTGGAGCAACGACGCAGCAATGCAGCCAGATCAAATCAACAAAGTGGTTGCACTCGGTG
ACAGCTTGTCTGATACAGGCAACATCTTTAACGCATCACAATGGCGCTTCCCTAACCCGAACAGCTGGTTCTTAGG
TCACTTCTCCAACGGTTTTGTGTGGACAGAATACATTGCCAAAGCGAAGAACCTTCCGCTCTACAACTGGGCAGTT
GGCGGCGCGGCTGGTGAGAACCAATACATCGCGCTAACAGGGGTTGGTGATCAAGTTTCTTCGTACTTAACCTACG
CAAAACTGGCGAAGAACTACAAACCAGCAAACACCTTGTTTACGCTTGAGTTTGGTTTGAATGACTTCATGAACTA
CAACCGTGGCGTTCCAGAAGTGAAAGCGGATTATGCAGAAGCACTGATTCGTTTGACGGACGCAGGTGCGAAGAA
CTTCATGTTGATGACACTGCCAGACGCGACGAAAGCGCCTCAGTTTAAGTACTCAACACAAGAAGAGATCGACAA
AATTCGTGCGAAAGTGCTTGAGATGAACGAGTTCATCAAGGCACAAGCGATGTACTACAAAGCGCAAGGTTACAA
CATCACGTTGTTTGATACTCACGCCTTGTTCGAGACGCTAACTTCTGCGCCAGAAGAGCACGGTTTCGTGAACGCG
AGCGATCCTTGTTTGGACATCAACCGCTCATCGTCTGTCGATTACATGTACACCCACGCATTGCGCTCTGAGTGTGC
AGCGTCTGGTGCTGAGAAGTTTGTGTTCTGGGATGTCACGCATCCAACAACAGCAACTCACCGCTATGTTGCAGAG
AAAATGCTAGAAAGTAGCAACAACTTAGCAGAGTACCGTTTCTAA

>CFSAN007462
ATGATGAAAAAAACAATCACACTATTAACTGCATTACTCCCGCTTGCTTCTGCAGTTGCCGAAGAGCCAACCTTAT
CACCAGAAATGGTTTCAGCGTCTGAAGTGATCAGCACGCAAGAAAACCAAACCTATACCTATGTTCGCTGTTGGTA
TCGCACCAGCTACTCGAAAGATGATCCGGCGACCGATTGGGAATGGGCAAAAACGAAGATGGTAGCTACTTCACC
ATTGACGGCTACTGGTGGAGCTCCGTTTCACTTAAAAACATGTTCTACACCAACACGTCGCAAAACGTTATCCGTC
AGCGTTGTGAAGCAACATTAGATTTGGCGAACGAGAACGCAGACATTACGTTCTTCGCCGCTGACAATCGCTTCTC
ATACAACCACACGATCTGGAGCAACGACGCAGCAATGCAGCCAGATCAAATCAACAAAGTGGTTGCACTCGGTGA
CAGCTTGTCTGATACAGGCAACATCTTTAACGCATCACAATGGCGCTTCCCTAACCCGAACAGCTGGTTCTTAGGT
CACTTCTCCAACGGTTTTGTGTGGACAGAATACATTGCCAAAGCGAAGAACCTTCCGCTCTACAACTGGGCAGTTG
GCGGCGCGGCTGGTGAGAACCAATACATCGCGCTAACAGGGGTTGGTGATCAAGTTTCTTCGTACTTAACCTACGC
AAAACTGGCGAAGAACTACAAACCAGCAAACACCTTGTTTACGCTTGAGTTTGGTTTGAATGACTTCATGAACTAC
AACCGTGGCGTTCCAGAAGTGAAAGCAGATTATGCAGAAGCACTGATTCGTTTGACGGACGCAGGTGCGAAGAAC
TTCATGTTGATGACACTGCCAGACGCGACGAAAGCGCCTCAGTTTAAGTACTCAACACAAGAAGAGATCGACAAA
TTCGTGCGAAAGTGCTTGAGATGAACGAGTTCATCAAGGCACAAGCGATGTACTACAAAGCGCAAGGTTACAACA
TCACGTTGTTTGATACTCACGCCTTGTTCGAGACGCTAACTTCTGCGCCAGAAGAGCACGGTTTCGTGAACGCGAG
CGATCCTTGTTTGGACATCAACCGCTCATCGTCTGTCGATTACATGTACACCCACGCATTGCGCTCTGAGTGTGCAG
CGTCTGGTGCTGAGAAGTTTGTGTTCTGGGATGTCACGCATCCAACAACAGCAACTCACCGCTATGTTGCAGAGAA
AATGCTAGAAAGTAGCAACAACTTAGCCGAGTACCGTTTCTAA

>CFSAN006130
ATGATGAAAAAACAATCACACTATTAACTGCATTACTCCCGCTTGCTTCTGCAGTTGCCGAAGAGCCAACCTTATC
ACCAGAAATGGTTTCAGCGTCTGAAGTGATCAGCACGCAAGAAAACCAAACCTATACCTATGTTCGCTGTTGGTAT
CGCACCAGCTACTCGAAAGATGATCCGGCGACCGATTGGGAATGGGCAAAAAACGAAGATGGTAGCTACTTCACC
ATTGACGGCTACTGGTGGAGCTCCGTTTCACTTAAAAACATGTTCTACACCAACACGTCGCAAAACGTTATCCGTC
AGCGTTGTGAAGCAACATTAGATTTGGCGAACGAGAACGCAGACATTACGTTCTTCGCCGCTGACAATCGCTTCTC
ATACAACCACACGATCTGGAGCAACGACGCAGCAATGCAGCCAGATCAAATCAACAAAGTGGTTGCACTCGGTGA
CAGCTTGTCTGATACAGGCAACATCTTTAACGCATCACAATGGCGCTTCCCTAACCCGAACAGCTGGTTCTTAGGT
CACTTCTCCAACGGTTTTGTGTGGACAGAATACATTGCCAAAGCGAAGAACCTTCCGCTCTACAACTGGGCAGTTG
GCGGCGCGGCTGGTGAGAACCAATACATCGCGCTAACAGGGGTTGGTGATCAAGTTTCTTCGTACTTAACCTACGC
AAAACTGGCGAAGAACTACAAACCAGCAAACACCTTGTTTACGCTTGAGTTTGGTTTGAATGACTTCATGAACTAC
AACCGTGGCGTTCCAGAAGTGAAAGCAGATTATGCAGAAGCACTGATTCGTTTGACGGACGCAGGTGCGAAGAAC
TTCATGTTGATGACACTGCCAGACGCGACGAAAGCGCCTCAGTTTAAGTACTCAACACAAGAAGAGATCGACAAA
TTCGTGCGAAAGTGCTTGAGATGAACGAGTTCATCAAGGCACAAGCGATGTACTACAAAGCGCAAGGTTACAACA

TCACGTTGTTTGATACTCACGCCTTGTTCGAGACGCTAACTTCTGCGCCAGAAGAGCACGGTTTCGTGAACGCGAG
CGATCCTTGTTTGGACATCAACCGCTCATCGTCTGTCGATTACATGTACACCCACGCATTGCGCTCTGAGTGTGCAG
CGTCTGGTGCTGAGAAGTTTGTGTTCTGGGATGTCACGCATCCAACAACAGCAACTCACCGCTATGTTGCAGAGAA
AATGCTAGAAAGTAGCAACAACTTAGCCGAGTACCGTTTCTAA

>CFSAN007441
ATGATGAAAAAAACAATCACACTATTAACTGCATTACTCCCGCTTGCTTCTGCAGTTGCCGAAGAGCCAACCTTAT
CACCAGAAATGGTTTCAGCGTCTGAAGTGATCAGCACGCAAGAAAACCAAACCTATACCTATGTTCGCTGTTGGTA
TCGCACCAGCTACTCGAAAGATGATCCGGCGACCGATTGGGAATGGGCAAAAAACGAAGATGGTAGCTACTTCAC
CATTGACGGCTACTGGTGGAGCTCCGTTTCATTTAAAAACATGTTCTACACCAACACGTCGCAAAACGTTATCCGT
CAGCGTTGTGAAGCAACATTAGATTTGGCGAACGAGAACGCAGACATTACGTTCTTCGCCGCTGACAATCGCTTCT
CATACAACCACACGATCTGGAGCAACGACGCAGCAATGCAGCCAGATCAAATCAACAAAGTGGTTGCACTCGGTG
ACAGCTTGTCTGATACAGGCAACATCTTTAACGCATCACAATGGCGCTTCCCTAACCCGAACAGCTGGTTCTTAGG
TCACTTCTCCAACGGTTTTGTTTGGACAGAATACATTGCCAAAGCGAAGAACCTTCCGCTCTACAACTGGGCAGTT
GGCGGCGCGGCTGGTGAGAACCAATACATCGCGCTAACAGGGGTTGGTGATCAAGTTTCTTCGTACTTAACCTACG
CAAAACTGGCGAAGAACTACAAACCAGCAAACACCTTGTTTACGCTTGAGTTTGGTTTGAATGACTTCATGAACTA
CAACCGTGGCGTTCCAGAAGTGAAAGCGGATTATGCAGAAGCACTGATTCGTTTGACAGACGCAGGTGCGAAGAA
CTTCATGTTGATGACACTGCCAGACGCGACGAAAGCGCCTCAGTTTAAGTACTCAACACAAGAAGAGATCGACAA
AATTCGTGCGAAAGTGCTTGAGATGAACGAGTTCATCAAGGCACAAGCGATGTACTACAAAGCGCAAGGTTACAA
CATCACGTTGTTTGATACTCACGCCTTGTTCGAGACGCTAACTTCTGCGCCAGAAGAGCACGGTTTCGTGAACGCG
AGCGATCCTTGTTTGGACATCAACCGCTCATCGTCTGTCGATTACATGTACACCCACGCATTGCGCTCTGAGTGTGC
AGCGTCTGGTGCTGAGAAGTTTGTGTTCTGGGATGTCACGCACCCAACAACAGCAACTCACCGCTATGTTGCAGAG
AAAATGCTAGAAAGTAGCAACAACTTAGAAGAGTTTCGCTTTTAA

>CFSAN007460
ATGATGAAAAAAACAATCACACTATTAACTGCATTACTCCCGCTTGCTTCTGCAGTTGCCGAAGAGCCAA
CCTTATCACCAGAAATGGTTTCAGCGTCTGAAGTGATCAGCACGCAAGAAAACCAAACCTATACCTATGT
TCGCTGTTGGTATCGCACCAGCTACTCGAAAGATGATCCGGCGACCGATTGGGAATGGGCAAAAAACGAA
GATGGTAGCTACTTCACCATTGACGGCTACTGGTGGAGCTCCGTTTCACTTAAAAACATGTTCTACACCA
ACACGTCGCAAAACGTTATCCGTCAGCGTTGTGAAGCAACATTAGATTTGGCGAACGAGAACGCAGACAT
TACGTTCTTCGCCGCTGACAATCGCTTCTCATACAACCACACGATCTGGAGCAACGACGCAGCAATGCAG
CCAGATCAAATCAACAAAGTGGTTGCACTCGGTGACAGCTTGTCTGATACAGGCAACATCTTTAACGCAT
CACAATGGCGCTTCCCTAACCCGAACAGCTGGTTCTTAGGTCACTTCTCCAACGGTTTTGTGTGGACAGA
ATACATTGCCAAAGCGAAGAACCTTCCGCTCTACAACTGGGCAGTTGGCGGCGCGGCTGGTGAGAACCAA
TACATCGCGCTAACAGGGTTGGTGATCAAGTTTCTTCGTACTTAACCTACGCAAAACTGGCGAAGAACTA
CAAACCAGCAAACACCTTGTTTACGCTTGAGTTTGGTTTGAATGACTTCATGAACTACAACCGTGGCGTT
CCAGAAGTGAAAGCAGATTATGCAGAAGCACTGATTCGTTTGACGGACGCAGGTGCGAAGAACTTCATGT
TGATGACACTGCCAGACGCGACGAAAGCGCCTCAGTTTAAGTACTCAACACAAGAAGAGATCGACAAAAT
TCGTGCGAAAGTGCTTGAGATGAACGAGTTCATCAAGGCACAAGCGATGTACTACAAAGCGCAAGGTTAC
AACATCACGTTGTTTGATACTCACGCCTTGTTCGAGACGCTAACTTCTGCGCCAGAAGAGCACGGTTTCG
TGAACGCGAGCGATCCTTGTTTGGACATCAACCGCTCATCGTCTGTCGATTACATGTACACCCACGCATT
GCGCTCTGAGTGTGCAGCGTCTGGTGCTGAGAAGTTTGTGTTCTGGGATGTCACGCATCCAACAACAGCA
ACTCACCGCTATGTTGCAGAGAAAATGCTAGAAAGTAGCAACAACTTAGCCGAGTACCGTTTCTAA

>CFSAN007455
ATGATGAAAAAAACAATCACACTATTAACTGCATTACTCCCGCTTGCTTCTGCAGTTGCCGAAGAGCCAA
CCTTATCACCAGAAATGGTTTCAGCGTCTGAAGTGATCAGCACGCAAGAAAACCAAACCTATACCTATGT
TCGTTGTTGGTATCGCACCAGCTACTCGAAAGATGATCCGGCGACCGATTGGGAATGGGCAAAAAACGAA
GATGGTAGCTACTTCACCATTGACGGTTACTGGTGGAGCTCCGTTTCATTTAAAAACATGTTCTACACCA
ACACGTCGCAAAACGTTATCCGTCAGCGTTGTGAAGCCACATTAGATTTGGCGAACGAGAACGCAGACAT
TACGTTCTTCGCCGCTGACAATCGCTTCTCATACAACCACACGATCTGGAGCAACGACGCAGCAATGCAG

CCAGATCAAATCAACAAAGTGGTTGCACTCGGTGACAGCTTGTCTGATACAGGCAACATCTTTAACGCAT
CACAATGGCGCTTCCCTAACCCGAACAGCTGGTTCTTAGGTCACTTCTCCAACGGTTTTGTGTGGACAGA
ATACATTGCCAAAGCGAAGAACCTTCCGCTCTACAACTGGGCAGTTGGCGGCGCGGCTGGTGAGAACCAA
TACATCGCGCTAACAGGGGTTGGTGAGCAGGTTTCTTCGTACTTAACCTACGCAAAACTGGCGAAGAACT
ACAAACCAGCAAACACCTTGTTTACGCTTGAGTTTGGTTTGAATGACTTCATGAACTACAACCGTGGCGT
TCCAGAAGTGAAAGCGGATTATGCAGAAGCACTGATTCGTTTGACGGACGCAGGTGCGAAGAACTTCATG
TTGATGACATTGCCAGACGCGACGAAAGCGCCTCAGTTTAAGTACTCAACACAAGAAGAGATCGACAAAA
TTCGTGCGAAAGTGCTTGAGATGAACGAGTTCATCAAGGCACAAGCGATGTACTACAAAGCGCAAGGTTA
CAACATCACGTTGTTTGATACTCACGCCTTGTTCGAGACGCTAACTTCTGCGCCAGAAGAGCACGGTTTC
GTGAACGCGAGTGATCCTTGTTTGGACATCAACCGCTCATCGTCTGTCGATTACATGTACACCCACGCAT
TGCGCTCTGAGTGTGCAGCGTCTGGTGCTGAGAAATTTGTGTTCTGGGATGTCACGCACCCAACAACAGC
AACTCACCGCTATGTTGCAGAGAAAATGCTAGAAAGTAGCAACAACTTAGCCGAGTACCGTTTCTAA

>CFSAN007440
ATGATGAAAAAAACAATCACACTATTAACTGCATTACTCCCGCTTGCTTCTGCAGTTGCCGAAGAGCCAA
CCTTATCACCAGAAATGGTTTCAGCGTCTGAAGTGATCAGCACGCAAGAAAACCAAACCTATACCTATGT
TCGCTGTTGGTATCGCACCAGCTACTCGAAAGATGATCCGGCGACCGATTGGGAATGGGCAAAAAACGAA
GATGGTAGCTACTTCACCATTGACGGCTACTGGTGGAGCTCCGTTTCATTTAAAAACATGTTCTACACCA
ACACGTCGCAAAACGTTATCCGTCAGCGTTGTGAAGCAACATTAGATTTGGCGAACGAGAACGCAGACAT
TACGTTCTTCGCCGCTGACAATCGCTTCTCATACAACCACACGATCTGGAGCAACGACGCAGCAATGCAG
CCAGATCAAATCAACAAAGTGGTTGCACTCGGTGACAGCTTGTCTGATACAGGCAACATCTTTAACGCAT
CACAATGGCGCTTCCCTAACCCGAACAGCTGGTTCTTAGGTCACTTCTCCAACGGTTTTGTGTGGACAGA
ATACATTGCCAAAGCGAAGAACCTTCCGCTCTACAACTGGGCTGTTGGCGGTGCGGCTGGTGAGAACCAA
TACATCGCGCTAACAGGGGTTGGTGATCAAGTTTCTTCGTACTTAACCTACGCAAAACTGGCGAAGAACT
ACAAACCAGCAAACACCTTGTTTACGCTTGAGTTTGGTTTGAATGACTTCATGAACTACAACCGTGGCGT
TCCAGAAGTGAAAGCGGATTATGCAGAAGCACTGATTCGTTTGACGGACGCAGGTGCGAAGAACTTCATG
TTGATGACACTGCCAGACGCGACGAAAGCGCCTCAGTTTAAGTACTCAACACAAGAAGAGATCGACAAAA
TTCGTGCGAAAGTGCTTGAGATGAACGAGTTCATCAAGGCACAAGCGATGTACTACAAAGCGCAAGGTTA
CAACATCACGTTGTTTGATACTCACGCCTTGTTCGAGACGCTAACTTCTGCGCCAGAAGAGCACGGTTTC
GTGAACGCGAGCGATCCTTGTTTGGACATCAACCGCTCATCGTCTGTCGATTACATGTACACCCACGCAT
TGCGCTCTGAGTGTGCAGCGTCTGGTGCTGAGAAGTTTGTGTTCTGGGATGTCACGCACCCAACAACAGC
AACTCACCGCTATGTTGCAGAGAAAATGCTAGAAAGTAGCAACAACTTAGCAGAGTACCGTTTCTAA

>CFSAN007439
ATGATGAAAAAAACAATCACACTATTAACTGCATTACTCCCGCTTGCTTCTGCAGTTGCCGAAGAGCCAA
CCTTATCACCAGAAATGGTTTCAGCGTCTGAAGTGATCAGCACGCAAGAAAACCAAACCTATACCTATGT
TCGCTGTTGGTATCGCACCAGCTACTCGAAAGATGATCCGGCGACCGATTGGGAATGGGCAAAAAACGAA
GATGGTAGCTACTTCACCATTGACGGCTACTGGTGGAGCTCCGTTTCATTTAAAAACATGTTCTACACCA
ACACGTCGCAAAACGTTATCCGTCAGCGTTGTGAAGCAACATTAGATTTGGCGAACGAGAACGCAGACAT
TACGTTCTTCGCCGCTGACAATCGCTTCTCATACAACCACACGATCTGGAGCAACGACGCAGCAATGCAG
CCAGATCAAATCAACAAAGTGGTTGCACTCGGTGACAGCTTGTCTGATACAGGCAACATCTTTAACGCAT
CACAATGGCGCTTCCCTAACCCGAACAGCTGGTTCTTAGGTCACTTCTCCAACGGTTTTGTGTGGACAGA
ATACATTGCCAAAGCGAAGAACCTTCCGCTCTACAACTGGGCTGTTGGCGGTGCGGCTGGTGAGAACCAA
TACATCGCGCTAACAGGGGTTGGTGATCAAGTTTCTTCGTACTTAACCTACGCAAAACTGGCGAAGAACT
ACAAACCAGCAAACACCTTGTTTACGCTTGAGTTTGGTTTGAATGACTTCATGAACTACAACCGTGGCGT
TCCAGAAGTGAAAGCGGATTATGCAGAAGCACTGATTCGTTTGACGGACGCAGGTGCGAAGAACTTCATG
TTGATGACACTGCCAGACGCGACGAAAGCGCCTCAGTTTAAGTACTCAACACAAGAAGAGATCGACAAAA
TTCGTGCGAAAGTGCTTGAGATGAACGAGTTCATCAAGGCACAAGCGATGTACTACAAAGCGCAAGGTTA
CAACATCACGTTGTTTGATACTCACGCCTTGTTCGAGACGCTAACTTCTGCGCCAGAAGAGCACGGTTTC
GTGAACGCGAGCGATCCTTGTTTGGACATCAACCGCTCATCGTCTGTCGATTACATGTACACCCACGCAT
TGCGCTCTGAGTGTGCAGCGTCTGGTGCTGAGAAGTTTGTGTTCTGGGATGTCACGCACCCAACAACAGC

AACTCACCGCTATGTTGCAGAGAAAATGCTAGAAAGTAGCAACAACTTAGCAGAGTACCGTTTCTAA

>CFSAN001611
ATGATGAAAAAAACAATCACACTATTAACTGCATTACTCCCGCTTGCTTCTGCAGTTGCCGAAGAGCCAA
CCTTATCACCAGAAATGGTTTCAGCGTCTGAAGTGATCAGCACGCAAGAAAACCAAACCTATACCTATGT
TCGCTGTTGGTATCGCACCAGCTACTCGAAAGATGATCCGGCGACCGATTGGGAATGGGCAAAAAACGAA
GATGGTAGCTACTTCACCATTGACGGCTACTGGTGGAGCTCCGTTTCACTTAAAAACATGTTCTACACCA
ACACGTCGCAAAACGTTATCCGTCAGCGTTGTGAAGCAACATTAGATTTGGCGAACGAGAACGCAGACAT
TACGTTCTTCGCCGCTGACAATCGCTTCTCATACAACCACACGATCTGGAGCAACGACGCAGCAATGCAG
CCAGATCAAATCAACAAAGTGGTTGCACTCGGTGACAGCTTGTCTGATACAGGCAACATCTTTAACGCAT
CACAATGGCGCTTCCCTAACCCGAACAGCTGGTTCTTAGGTCACTTCTCCAACGGTTTTGTGTGGACAGA
ATACATTGCCAAAGCGAAGAACCTTCCGCTCTACAACTGGGCAGTTGGCGGCGCGGCTGGTGAGAACCAA
TACATCGCGCTAACAGGGGTTGGTGATCAAGTTTCTTCGTACTTAACCTACGCAAAACTGGCGAAGAACT
ACAAACCAGCAAACACCTTGTTTACGCTTGAGTTTGGTTTGAATGACTTCATGAACTACAACCGTGGCGT
TCCAGAAGTGAAAGCAGATTATGCAGAAGCACTGATTCGTTTGACGGACGCAGGTGCGAAGAACTTCATG
TTGATGACACTGCCAGACGCGACGAAAGCGCCTCAGTTTAAGTACTCAACACAAGAAGAGATCGACAAAA
TTCGTGCGAAAGTGCTTGAGATGAACGAGTTCATCAAGGCACAAGCGATGTACTACAAAGCGCAAGGTTA
CAACATCACGTTGTTTGATACTCACGCCTTGTTCGAGACGCTAACTTCTGCGCCAGAAGAGCACGGTTTC
GTGAACGCGAGCGATCCTTGTTTGGACATCAACCGCTCATCGTCTGTCGATTACATGTACACCCACGCAT
TGCGCTCTGAGTGTGCAGCGTCTGGTGCTGAGAAGTTTGTGTTCTGGGATGTCACGCATCCAACAACAGC
AACTCACCGCTATGTTGCAGAGAAAATGCTAGAAAGTAGCAACAACTTAGCCGAGTACCGTTTCTAA

>CFSAN007444
ATGATGAAAAAAACAATCACACTATTAACTGCATTACTCCCGCTTGCTTCTGCAGTTGCCGAAGAGCCAACCTTAT
CACCAGAAATGGTTTCAGCGTCTGAAGTGATCAGCACGCAAGAAAACCAAACCTATACCTATGTTCGCTGTTGGTA
TCGCACCAGCTACTCGAAAGATGATCCGGCGACCGATTGGGAATGGGCAAAAAACGAAGATGGTAGCTACTTCAC
CATTGACGGCTACTGGTGGAGCTCCGTTTCATTTAAAAACATGTTCTACACCAACACGTCGCAAAACGTTATCCGT
CAGCGTTGTGAAGCAACATTAGATTTGGCGAACGAGAACGCAGACATTACGTTCTTCGCCGCTGACAATCGCTTCT
CATACAACCACACGATCTGGAGCAACGACGCAGCAATGCAGCCAGATCAAATCAACAAAGTGGTTGCACTCGGTG
ACAGCTTGTCTGATACAGGCAACATCTTTAACGCATCACAATGGCGCTTCCCTAACCCGAACAGCTGGTTCTTAGG
TCACTTCTCCAACGGTTTTGTGTGGACAGAATACATTGCCAAAGCGAAGAACCTTCCGCTCTACAACTGGGCAGTT
GGCGGCGCGGCTGGTGAGAACCAATACATCGCGCTAACAGGGTTGGTGATCAAGTTTCTTCGTACTTAACCTACGC
AAAACTGGCGAAGAACTACAAACCAGCAAACACCTTGTTTACGCTTGAGTTTGGTTTGAATGACTTCATGAACTAC
AACCGTGGCGTTCCAGAAGTGAAAGCGGATTATGCAGAAGCACTGATTCGTTTGACGGACGCAGGTGCGAAGAAC
TTCATGTTGATGACACTGCCAGACGCGACGAAAGCGCCTCAGTTTAAGTACTCAACACAAGAAGAGATCGACAAA
ATTCGTGCGAAAGTGCTTGAGATGAACGAGTTCATCAAGGCACAAGCGATGTACTACAAAGCGCAAGGTTACAAC
ATCACGTTGTTTGATACTCACGCCTTGTTCGAGACGCTAACTTCTGCGCCAGAAGAGCACGGTTTCGTGAACGCGA
GTGATCCTTGTTTGGACATCAACCGCTCATCGTCTATCGATTACATGTACACCCACGCATTGCGCTCTGAGTGTGCG
GCGTCTGGTGCTGAGAAATTTGTGTTCTGGGATGTCACGCACCCAACAACAGCAACTCACCGCTATGTTGCAGAGA
AAATGCTAGAAAGTAGCAACAACTTAGCCGAGTACCGTTTCTAA

>CFSAN007457
ATGATGAAAAAAACAATCACACTATTAACTGCATTACTCCCGCTTGCTTCTGCAGTTGCCGAAGAGCCAA
CCTTATCACCAGAAATGGTTTCAGCGTCTGAAGTGATCAGCACGCAAGAAAACCAAACCTATACCTATGT
TCGCTGTTGGTATCGCACCAGCTACTCGAAAGATGATCCGGCGACCGGTTGGGAATGGGCAAAAAACGAA
GATGGTAGCTACTTCACCATTGACGGCTACTGGTGGAGCTCCGTTTCATTTAAAAACATGTTCTACACCA
ACACGTCGCAAAACGTTATCCGTCAGCGTTGTGAAGCAACATTAGATTTGGCGAACGAGAACGCAGACAT
TACGTTCTTCGCCGCTGACAATCGCTTCTCATACAACCACACGATCTGGAGCAACGACGCAGCAATGCAG
CCAGATCAAATCAACAAAGTGGTTGCACTCGGTGACAGCTTGTCTGATACAGGCAACATCTTTAACGCAT
CACAATGGCGCTTCCCTAACCCGAACAGCTGGTTCTTAGGTCACTTCTCGAACGGTTTTGTGTGGACAGA
ATACATTGCCAAAGCGAAGAACCTTCCGCTCTACAACTGGGCAGTTGGCGGCGCGGCTGGTGAGAACCAA

TACATCGCGCTAACAGGTTGGTGATCAAGTTTCTTCGTACTTAACCTACGCAAAACTGGCGAAGAACTAC
AAACCAGCAAACACCTTGTTTACGCTTGAGTTTGGTTTGAATGACTTCATGAACTACAACCGTGGCGTTC
CAGAAGTGAAAGCGGATTATGCAGAAGCACTGATTCGTTTGACGGACGCAGGTGCGAAGAACTTCATGTT
GATGACACTGCCAGACGCGACGAAAGCGCCTCAGTTTAAGTACTCAACACAAGAAGAGATCGACAAAATT
CGTGCGAAAGTGCTTGAGATGAACGAGTTCATCAAGGCACAAGCGATGTACTACAAAGCGCAAGGTTACA
ACATCACGTTGTTTGATACTCACGCCTTGTTCGAGACGCTAACTTCTGCGCCAGAAGAGCACGGTTTCGT
GAACGCGAGCGATCCTTGTTTGGACATCAACCGCTCATCGTCTGTCGACTACATGTACACCCACGCATTG
CGCTCTGAGTGTGCGGCGTCTGGTGCTGAGAAGTTTGTATTCTGGGATGTCACGCACCCAACAACAGCAA
CTCACCGCTATGTTGCAGAGAAAATGCTAGAAAGTAGCAACAACTTAGCCGAGTACCGTTTCTAA

>CFSAN007445
ATGATGAAAAAAACAATCACACTATTAACTGCATTACTCCCGCTTGCTTCTGCAGTTGCCGAAGAGCCA
ACCTTATCACCAGAAATGGTTTCAGCGTCTGAAGTGATCAGCACGCAAGAAAACCAAACCTATACCTATG
TTCGCTGTTGGTATCGCACCAGCTACTCGAAAGATGATCCGGCGACCGATTGGGAATGGGCAAAAAACGA
AGATGGTAGCTACTTCACCATTGACGGCTACTGGTGGAGCTCCGTTTCATTTAAAAACATGTTCTACACC
AACACGTCGCAAAACGTTATCCGTCAGCGTTGTGAAGCAACATTAGATTTGGCGAACGAGAACGCAGACA
TTACGTTCTTCGCCGCTGACAATCGCTTCTCATACAACCACACGATCTGGAGCAACGACGCAGCAATGCA
GCCAGATCAAATCAACAAAGTGGTTGCACTCGGTGACAGCTTGTCTGATACAGGCAACATCTTTAACGCA
TCACAATGGCGCTTCCCTAACCCGAACAGCTGGTTCTTAGGTCACTTCTCCAACGGTTTTGTGTGGACAG
AATACATTGCCAAAGCGAAGAACCTTCCGCTCTACAACTGGGCAGTTGGCGGTGCGGCTGGTGAGAACCA
ATACATCGCGCTAACAGGGGTTGGTGATCAAGTTTCTTCGTACTTAACCTACGCAAAACTGGCGAAGAAC
TACAAACCAGCAAACACCTTGTTTACGCTTGAGTTTGGTTTGAATGACTTCATGAACTACAACCGTGGCG
TTCCAGAAGTGAAAGCGGATTATGCAGAAGCACTGATTCGTTTGACGGACGCAGGTGCGAAGAACTTCAT
GTTGATGACACTGCCAGATGCGACGAAAGCGCCTCAGTTTAAGTACTCAACACAAGAAGAGATCGACAAA
ATTCGTGCGAAAGTGCTTGAGATGAACGAGTTCATCAAGGCACAAGCGATGTACTACAAAGCGCAAGGTT
ACAACATCACGTTGTTTGATACTCACGCCTTGTTCGAGACGCTAACTTCTGCGCCAGAAGAGCACGGTTT
CGTGAACGCGAGCGATCCTTGTTTGGACATCAACCGCTCATCGTCTGTCGATTACATGTACACCCACGCA
TTGCGCTCTGAGTGTGCAGCGTCTGGTGCTGAGAAGTTTGTGTTCTGGGATGTCACGCACCCAACAACAG
CAACTCACCGCTATGTTGCAGAGAAAATGCTAGAAAGTAGCAACAACTTAGAAGAGTTTCGCTTTTA

>CFSAN007438
ATGATGAAAAAAACAATCACACTATTAACTGCATTACTCCCGCTTGCTTCTGCAGTTGCCGAAGAGCCAACCTTAT
CACCAGAAATGGTTTCAGCGTCTGAAGTGATCAGCACGCAAGAAAACCAAACCTATACCTATGTTCGCTGTTGGTA
TCGCACCAGCTACTCGAAAGATGATCCGGCGACCGATTGGGAATGGGCAAAAAACGAAGATGGTAGCTACTTCAC
CATTGACGGCTACTGGTGGAGCTCCGTTTCATTTAAAAACATGTTCTACACCAACACGTCGCAAAACGTTATCCGT
CAGCGTTGTGAAGCCACATTAGATTTGGCGAACGAGAACGCAGACATTACGTTCTTCGCCGCTGACAATCGCTTCT
CATACAACCACACGATCTGGAGCAACGACGCAGCAATGCAGCCAGATCAAATCAACAAAGTGGTTGCACTCGGTG
ACAGCTTGTCTGATACAGGCAACATCTTTAACGCATCACAATGGCGCTTCCCTAACCCGAACAGCTGGTTCTTAGG
TCACTTCTCCAACGGTTTTGTGTGGACAGAATACATTGCCAAAGCGAAGAACCTTCCGCTCTACAACTGGGCAGTT
GGCGGCGCGGCTGGTGAGAACCAATACATCGCGCTAACAGGGGTTGGTGATCAAGTTTCTTCGTACTTAACCTACG
CAAAACTGGCGAAGAACTACAAACCAGCAAACACCTTGTTTACGCTTGAGTTTGGTTTGAATGACTTCATGAACTA
CAACCGTGGCGTTCCAGAAGTGAAAGCAGATTATGCAGAAGCACTGATTCGTTTGACGGACGCAGGTGCGAAGAA
CTTCATGTTGATGACACTGCCAGACGCGACGAAAGCGCCTCAGTTTAAGTACTCAACACAAGAAGAGATCGACAA
AATTCGTGCGAAAGTGCTTGAGATGAACGAGTTCATCAAGGCACAAGCGATGTACTACAAAGCGCAAGGTTACAA
CATCACGTTGTTTGATACTCACGCCTTGTTCGAGACGCTAACTTCTGCGCCCGAAGAGCACGGTTTCGTGAACGCG
AGCGATCCTTGTTTGGACATCAACCGCTCATCGTCTGTCGATTACATGTACACCCACGCATTGCGCTCTGAGTGTGC
AGCGTCTGGTGCTGAGAAGTTTGTGTTCTGGGATGTCACGCATCCAACAACAGCAACTCACCGCTATGTTGCAGAG
AAAATGCTAGAAAGTAGCAACAACTTAGCCGAGTACCGTTTCTAA

>CFSAN007437

ATGATGAAAAAAACAATCACACTATTAACTGCATTACTCCCGCTTGCTTCTGCAGTTGCCGAAGAGCCAACCTTAT
CACCAGAAATGGTTTCAGCGTCTGAAGTGATCAGCACACAAGAAAACCAAACCTATACCTATGTTCGCTGTTGGTA
TCGCACCAGCTACTCGAAAGATGATCCGGCGACCGATTGGGAATGGGCAAAAAACGAAGATGGTAGCTACTTCAC
CATTGACGGTTACTGGTGGAGCTCCGTTTCATTTAAAAACATGTTCTACACCAACACGTCGCAAAACGTTATCCGT
CAGCGTTGTGAAGCCACATTAGATTTGGCGAACGAGAACGCAGACATTACGTTCTTCGCCGCTGACAATCGCTTCT
CATACAACCACACGATCTGGAGCAACGACGCAGCAATGCAGCCAGATCAAATCAACAAAGTGGTTGCTCTCGGTG
ACAGCTTGTCTGATACAGGCAACATCTTTAACGCATCACAATGGCGCTTCCCTAACCCGAACAGCTGGTTCTTAGG
TCACTTCTCCAACGGTTTTGTGTGGACAGAATACATTGCCAAAGCGAAGAACCTTCCGCTCTACAACTGGGCAGTT
GGCGGCGCGGCTGGTGAGAACCAATACATCGCGCTAACAGGGGTTGGTGAGCAAGTTTCTTCGTACTTAACCTACG
CAAAACTGGCGAAGAACTACAAACCAGCAAACACCTTGTTTACGCTTGAGTTTGGTTTGAATGACTTCATGAACTA
CAACCGTGGCGTTCCAGAAGTGAAAGCGGATTATGCAGAAGCACTGATTCGTTTGACGGACGCAGGTGCGAAGAA
CTTCATGTTGATGACACTGCCAGACGCGACGAAAGCGCCTCAGTTTAAATACTCAACACAAGAAGAGATCGACAA
AATTCGTGCGAAAGTGCTTGAGATGAACGAGTTCATCAAGGCACAAGCGATGTACTACAAAGCGCAAGGTTACAA
CATAACGTTGTTTGATACTCACGCCTTGTTCGAGACGCTAACTTCTGCGCCCGAAGAGCACGGTTTCGTGAACGCG
AGCGATCCTTGTTTGGACATCAACCGCTCATCGTCTGTCGATTACATGTACACCCACGCATTGCGCTCTGAGTGTGC
AGCGTCTGGTGCTGAGAAGTTTGTGTTCTGGGATGTCACGCATCCAACAACAGCAACTCACCGCTATGTTGCAGAG
AAAATGCTAGAAAGTAGCAACAACTTAGCCGAGTACCGTTTCTAA

>CFSAN007459
ATGATGAAAAAAACAATCACACTATTAACTGCATTACTCCCGCTTGCTTCTGCAGTTGCCGAAGAGCCAACCTTAT
CACCAGAAATGGTTTCAGCGTCTGAAGTGATCAGCACGCAAGAAAACCAAACCTATACCTATGTTCGCTGTTGGTA
TCGCACCAGCTACTCGAAAGATGATCCGGCGACCGATTGGGAATGGGCAAAAAACGAAGATGGTAGCTACTTCAC
CATTGACGGCTACTGGTGGAGCTCCGTTTCACTTAAAAACATGTTCTACACCAACACGTCGCAAAACGTTATCCGT
CAGCGTTGTGAAGCAACATTAGATTTGGCGAACGAGAACGCAGACATTACGTTCTTCGCCGCTGACAATCGCTTCT
CATACAACCACACGATCTGGAGCAACGACGCAGCAATGCAGCCAGATCAAATCAACAAAGTGGTTGCACTCGGTG
ACAGCTTGTCTGATACAGGCAACATCTTTAACGCATCACAATGGCGCTTCCCTAACCCGAACAGCTGGTTCTTAGG
TCACTTCTCCAACGGTTTTGTGTGGACAGAATACATTGCCAAAGCGAAGAACCTTCCGCTCTACAACTGGGCAGTT
GGCGGCGCGGCTGGTGAGAACCAATACATCGCGCTAACAGGGGTTGGTGATCAAGTTTCTTCGTACTTAACCTACG
CAAAACTGGCGAAGAACTACAAACCAGCAAACACCTTGTTTACGCTTGAGTTTGGTTTGAATGACTTCATGAACTA
CAACCGTGGCGTTCCAGAAGTGAAAGCAGATTATGCAGAAGCACTGATTCGTTTGACGGACGCAGGTGCGAAGAA
CTTCATGTTGATGACACTGCCAGACGCGACGAAAGCGCCTCAGTTTAAGTACTCAACACAAGAAGAGATCGACAA
AATTCGTGCGAAAGTGCTTGAGATGAACGAGTTCATCAAGGCACAAGCGATGTACTACAAAGCGCAAGGTTACAA
CATCACGTTGTTTGATACTCACGCCTTGTTCGAGACGCTAACTTCTGCGCCAGAAGAGCACGGTTTCGTGAACGCG
AGCGATCCTTGTTTGGACATCAACCGCTCATCGTCTGTCGATTACATGTACACCCACGCATTGCGCTCTGAGTGTGC
AGCGTCTGGTGCTGAGAAGTTTGTGTTCTGGGATGTCACGCATCCAACAACAGCAACTCACCGCTATGTTGCAGAG
AAAATGCTAGAAAGTAGCAACAACTTAGCCGAGTACCGTTTCTAA

>CFSAN007461
ATGATGAAAAAAACAATCACACTATTAACTGCATTACTCCCGCTTGCTTCTGCAGTTGCCGAAGAGCCAACCTTATC
ACCAGAAATGGTTTCAGCGTCTGAAGTGATCAGCACGCAAGAAAACCAAACCTATACCTATGTTCGCTGTTGGTAT
CGCACCAGCTACTCGAAAGATGATCCGGCGACCGATTGGGAATGGGCAAAAAACGAAGATGGTAGCTACTTCACC
ATTGACGGCTACTGGTGGAGCTCCGTTTCACTTAAAAACATGTTCTACACCAACACGTCGCAAAACGTTATCCGTC
AGCGTTGTGAAGCAACATTAGATTTGGCGAACGAGAACGCAGACATTACGTTCTTCGCCGCTGACAATCGCTTCTC
ATACAACCACACGATCTGGAGCAACGACGCAGCAATGCAGCCAGATCAAATCAACAAAGTGGTTGCACTCGGTGA
CAGCTTGTCTGATACAGGCAACATCTTTAACGCATCACAATGGCGCTTCCCTAACCCGAACAGCTGGTTCTTAGGT
CACTTCTCCAACGGTTTTGTGTGGACAGAATACATTGCCAAAGCGAAGAACCTTCCGCTCTACAACTGGGCAGTTG
GCGGCGCGGCTGGTGAGAACCAATACATCGCGCTAACAGGGGTTGGTGATCAAGTTTCTTCGTACTTAACCTACGC
AAAACTGGCGAAGAACTACAAACCAGCAAACACCTTGTTTACGCTTGAGTTTGGTTTGAATGACTTCATGAACTAC
AACCGTGGCGTTCCAGAAGTGAAAGCAGATTATGCAGAAGCACTGATTCGTTTGACGGACGCAGGTGCGAAGAAC
TTCATGTTGATGACACTGCCAGACGCGACGAAAGCGCCTCAGTTTAAGTACTCAACACAAGAAGAGATCGACAAA
ATTCGTGCGAAAGTGCTTGAGATGAACGAGTTCATCAAGGCACAAGCGATGTACTACAAAGCGCAAGGTTACAAC

ATCACGTTGTTTGATACTCACGCCTTGTTCGAGACGCTAACTTCTGCGCCAGAAGAGCACGGTTTCGTGAACGCGA
GCGATCCTTGTTTGGACATCAACCGCTCATCGTCTGTCGATTACATGTACACCCACGCATTGCGCTCTGAGTGTGCA
GCGTCTGGTGCTGAGAAGTTTGTGTTCTGGGATGTCACGCATCCAACAACAGCAACTCACCGCTATGTTGCAGAGA
AAATGCTAGAAAGTAGCAACAACTTAGCCGAGTACCGTTTCTAA

>CFSAN006129
ATGATGAAAAAAACAATCACACTATTAACTGCATTACTCCCGCTTGCTTCTGCAGTTGCCGAAGAGCCAACCTTAT
CACCAGAAATGGTTTCAGCGTCTGAAGTGATCAGCACGCAAGAAAACCAAACCTATACCTATGTTCGCTGTTGGTA
TCGCACCAGCTACTCGAAAGATGATCCGGCGACCGATTGGGAATGGGCAAAAAACGAAGATGGTAGCTACTTCAC
CATTGACGGCTACTGGTGGAGCTCCGTTTCACTTAAAAACATGTTCTACACCAACACGTCGCAAAACGTTATCCGT
CAGCGTTGTGAAGCAACATTAGATTTGGCGAACGAGAACGCAGACATTACGTTCTTCGCCGCTGACAATCGCTTCT
CATACAACCACACGATCTGGAGCAACGACGCAGCAATGCAGCCAGATCAAATCAACAAAGTGGTTGCACTCGGTG
ACAGCTTGTCTGATACAGGCAACATCTTTAACGCATCACAATGGCGCTTCCCTAACCCGAACAGCTGGTTCTTAGG
TCACTTCTCCAACGGTTTTGTGTGGACAGAATACATTGCCAAAGCGAAGAACCTTCCGCTCTACAACTGGGCAGTT
GGCGGCGCGGCTGGTGAGAACCAATACATCGCGCTAACAGGGGTTGGTGATCAAGTTTCTTCGTACTTAACCTACG
CAAAACTGGCGAAGAACTACAAACCAGCAAACACCTTGTTTACGCTTGAGTTTGGTTTGAATGACTTCATGAACTA
CAACCGTGGCGTTCCAGAAGTGAAAGCAGATTATGCAGAAGCACTGATTCGTTTGACGGACGCAGGTGCGAAGAA
CTTCATGTTGATGACACTGCCAGACGCGACGAAAGCGCCTCAGTTTAAGTACTCAACACAAGAAGAGATCGACAA
AATTCGTGCGAAAGTGCTTGAGATGAACGAGTTCATCAAGGCACAAGCGATGTACTACAAAGCGCAAGGTTACAA
CATCACGTTGTTTGATACTCACGCCTTGTTCGAGACGCTAACTTCTGCGCCAGAAGAGCACGGTTTCGTGAACGCG
AGCGATCCTTGTTTGGACATCAACCGCTCATCGTCTGTCGATTACATGTACACCCACGCATTGCGCTCTGAGTGTGC
AGCGTCTGGTGCTGAGAAGTTTGTGTTCTGGGATGTCACGCATCCAACAACAGCAACTCACCGCTATGTTGCAGAG
AAAATGCTAGAAAGTAGCAACAACTTAGCCGAGTACCGTTTCTAA

>CFSAN001617
ATGATGAAAAAAACAATCACACTATTAACTGCATTACTCCCGCTTGCTTCTGCAGTTGCCGAAGAGCCAACCTTAT
CACCAGAAATGGTTTCAGCGTCTGAAGTGATCAGCACGCAAGAAAACCAAACCTATACCTATGTTCGCTGTTGGTA
TCGCACCAGCTACTCGAAAGATGATCCGGCGACCGGTTGGGAATGGGCAAAAAACGAAGATGGTAGCTACTTCAC
CATTGACGGCTACTGGTGGAGCTCCGTTTCATTTAAAAACATGTTCTACACCAACACGTCGCAAAACGTTATCCGT
CAGCGTTGTGAAGCAACATTAGATTTGGCGAACGAGAACGCAGACATTACGTTCTTCGCCGCTGACAATCGCTTCT
CATACAACCACACGATCTGGAGCAACGACGCAGCAATGCAGCCAGATCAAATCAACAAAGTGGTTGCACTCGGTG
ACAGCTTGTCTGATACAGGCAACATCTTTAACGCATCACAATGGCGCTTCCCTAACCCGAACAGCTGGTTCTTAGG
TCACTTCTCGAACGGTTTTGTGTGGACAGAATACATTGCCAAAGCGAAGAACCTTCCGCTCTACAACTGGGCAGTT
GGCGGCGCGGCTGGTGAGAACCAATACATCGCGCTAACAGGGTTGGTGATCAAGTTTCTTCGTACTTAACCTACGC
AAAACTGGCGAAGAACTACAAACCAGCAAACACCTTGTTTACGCTTGAGTTTGGTTTGAATGACTTCATGAACTAC
AACCGTGGCGTTCCAGAAGTGAAAGCGGATTATGCAGAAGCACTGATTCGTTTGACGGACGCAGGTGCGAAGAAC
TTCATGTTGATGACACTGCCAGACGCGACGAAAGCGCCTCAGTTTAAGTACTCAACACAAGAAGAGATCGACAAA
ATTCGTGCGAAAGTGCTTGAGATGAACGAGTTCATCAAGGCACAAGCGATGTACTACAAAGCGCAAGGTTACAAC
ATCACGTTGTTTGATACTCACGCCTTGTTCGAGACGCTAACTTCTGCGCCAGAAGAGCACGGTTTCGTGAACGCGA
GCGATCCTTGTTTGGACATCAACCGCTCATCGTCTGTCGACTACATGTACACCCACGCATTGCGCTCTGAGTGTGCG
GCGTCTGGTGCTGAGAAGTTTGTATTCTGGGATGTCACGCACCCAACAACAGCAACTCACCGCTATGTTGCAGAGA
AAATGCTAGAAAGTAGCAACAACTTAGCCGAGTACCGTTTCTAA

>CFSAN012493
ATGATGAAAAAAACAATCACACTATTAACTGCATTACTCCCGCTTGCTTCTGCAGTTGCCGAAGAGCCAA
CCTTATCACCAGAAATGGTTTCAGCGTCTGAAGTGATCAGCACGCAAGAAAACCAAACCTATACCTATGT
TCGCTGTTGGTATCGCACCAGCTACTCGAAAGATGATCCGGCGACCGATTGGGAATGGGCAAAAAACGAA
GATGGTAGCTACTTCACCATTGACGGCTACTGGTGGAGCTCCGTTTCATTTAAAAACATGTTCTACACCA
ACACGTCGCAAAACGTTATCCGTCAGCGTTGTGAAGCAACATTAGATTTGACGAACGAGAACGCAGACAT
TACGTTCTTCGCCGCTGACAATCGCTTCTCATACAACCACACGATCTGGAGCAACGACGCAGCAATGCAG
CCAGATCAAATCAACAAAGTGGTTGCACTCGGTGACAGCTTGTCTGATACAGGCAACATCTTTAACGCAT

CACAATGGCGCTTCCCTAACCCGAACAGCTGGTTCTTAGGTCACTTCTCCAACGGTTTTGTGTGGACAGA
ATACATTGCCAAAGCGAAGAACCTTCCGCTCTACAACTGGGCAGTTGGCGGCGCGGCTGGTGAGAACCAA
TACATCGCGCTAACAGGGTTGGTGAGCAAGTTTCTTCGTACTTAACCTACGCAAAACTGGCGAAGAACTA
CAAACCAGCAAACACCTTGTTTACGCTTGAGTTTGGTTTGAATGACTTCATGAACTACAACCGTGGCGTT
CCAGAAGTGAAAGCAGATTATGCAGAAGCACTGATTCGTTTGACGGACGCAGGTGCGAAGAACTTCATGT
TGATGACACTGCCAGACGCGACGAAAGCGCCTCAGTTTAAGTACTCAACACAAGAAGAGATCGACAAAAT
TCGTGCGAAAGTGCTTGAGATGAACGAGTTCATCAAGGCACAAGCGATGTACTACAAAGCGCAAGGTTAC
AACATCACGTTGTTTGATACTCACGCCTTGTTCGAGACGCTAACTTCTGCGCCCGAAGAGCACGGTTTCG
TGAACGCGAGTGATCCTTGTTTGGACATCAACCGCTCATCGTCTGTCGATTACATGTACACCCACGCATT
GCGCTCTGAGTGTGCTGCGTCTGGTGCTGAGAAGTTTGTGTTCTGGGATGTCACGCATCCAACAACAGCA
ACTCACCGCTATGTTGCAGAGAAAATGCTAGAAAGTAGCAACAACTTAGCAGAGTACCGTTTCTAA

>CFSAN007456
ATGATGAAAAAAACAATCACACTATTAACTGCATTACTCCCGCTTGCTTCTGCAGTTGCCGAAGAGCCAA
CCTTATCACCAGAAATGGTTTCAGCGTCTGAAGTGATCAGCACGCAAGAAAACCAAACCTATACCTATGT
TCGCTGTTGGTATCGCACCAGCTACTCGAAAGATGATCCGGCGACCGATTGGGAATGGGCAAAAAACGAA
GATGGTAGCTACTTCACCATTGACGGCTACTGGTGGAGCTCCGTTTCATTTAAAAACATGTTCTACACCA
ACACGTCGCAAAACGTTATCCGTCAGCGTTGTGAAGCAACATTAGATTTGGCGAACGAGAACGCAGACAT
TACGTTCTTCGCCGCTGACAATCGCTTCTCATACAACCACACGATCTGGAGCAACGACGCAGCAATGCAG
CCAGATCAAATCAACAAAGTGGTTGCACTCGGTGACAGCTTGTCTGATACAGGCAACATCTTTAACGCAT
CACAATGGCGCTTCCCTAACCCGAACAGCTGGTTCTTAGGTCACTTCTCCAACGGTTTTGTGTGGACAGA
ATACATTGCCAAAGCGAAGAACCTTCCGCTCTACAACTGGGCAGTTGGCGGCGCGGCTGGTGAGAACCAA
TACATCGCGCTAACAGGGGTTGGTGATCAAGTTTCTTCGTACTTAACCTACGCAAAACTGGCGAAGAACT
ACAAACCAGCAAACACCTTGTTTACGCTTGAGTTTGGTTTGAATGACTTCATGAACTACAACCGTGGCGT
TCCAGAAGTGAAAGCGGATTATGCAGAAGCACTGATTCGCTTGACGGACGCAGGTGCGAAGAACTTCATG
TTGATGACACTGCCAGACGCGACGAAAGCGCCTCAGTTTAAGTACTCAACACAAGAAGAGATCGACAAAA
TTCGTGCGAAAGTGCTTGAGATGAACGAGTTCATCAAGGCACAAGCGATGTACTACAAAGCGCAAGGTTA
CAACATCACGTTGTTTGATACTCACGCCTTGTTCGAGACGCTAACTTCTGCGCCAGAAGAGCACGGTTTC
GTGAACGCGAGCGATCCTTGTTTGGACATCAACCGCTCATCGTCTGTCGACTACATGTACACCCACGCAT
TGCGCTCTGAGTGTGCGGCGTCTGGTGCTGAGAAGTTTGTGTTCTGGGATGTCACGCACCCAACAACAGC
AACTCACCGCTATGTTGCAGAGAAAATGCTAGAAAGTAGCAACAACTTAGCCGAGTACCGTTTCTAA

>CFSAN006131
ATGATGAAAAAAACAATCACACTATTAACTGCATTACTCCCGCTTGCTTCTGCAGTTGCCGAAGAGCCAACCTTAT
CACCAGAAATGGTTTCAGCGTCTGAAGTGATCAGCACGCAAGAAAACCAAACCTATACCTATGTTCGCTGTTGGTA
TCGCACCAGCTACTCGAAAGATGATCCGGCGACCGATTGGGAATGGGCAAAAAACGAAGATGGTAGCTACTTCAC
CATTGACGGCTACTGGTGGAGCTCCGTTTCACTTAAAAACATGTTCTACACCAACACGTCGCAAAACGTTATCCGT
CAGCGTTGTGAAGCAACATTAGATTTGGCGAACGAGAACGCAGACATTACGTTCTTCGCCGCTGACAATCGCTTCT
CATACAACCACACGATCTGGAGCAACGACGCAGCAATGCAGCCAGATCAAATCAACAAAGTGGTTGCACTCGGTG
ACAGCTTGTCTGATACAGGCAACATCTTTAACGCATCACAATGGCGCTTCCCTAACCCGAACAGCTGGTTCTTAGG
TCACTTCTCCAACGGTTTTGTGTGGACAGAATACATTGCCAAAGCGAAGAACCTTCCGCTCTACAACTGGGCAGTT
GGCGGCGCGGCTGGTGAGAACCAATACATCGCGCTAACAGGGGTTGGTGATCAAGTTTCTTCGTACTTAACCTACG
CAAAACTGGCGAAGAACTACAAACCAGCAAACACCTTGTTTACGCTTGAGTTTGGTTTGAATGACTTCATGAACTA
CAACCGTGGCGTTCCAGAAGTGAAAGCAGATTATGCAGAAGCACTGATTCGTTTGACGGACGCAGGTGCGAAGAA
CTTCATGTTGATGACACTGCCAGACGCGACGAAAGCGCCTCAGTTTAAGTACTCAACACAAGAAGAGATCGACAA
AATTCGTGCGAAAGTGCTTGAGATGAACGAGTTCATCAAGGCACAAGCGATGTACTACAAAGCGCAAGGTTACAA
CATCACGTTGTTTGATACTCACGCCTTGTTCGAGACGCTAACTTCTGCGCCAGAAGAGCACGGTTTCGTGAACGCG
AGCGATCCTTGTTTGGACATCAACCGCTCATCGTCTGTCGATTACATGTACACCCACGCATTGCGCTCTGAGTGTGC

AGCGTCTGGTGCTGAGAAGTTTGTGTTCTGGGATGTCACGCATCCAACAACAGCAACTCACCGCTATGTTGCAGAG
AAAATGCTAGAAAGTAGCAACAACTTAGCCGAGTACCGTTTCTAA

>CFSAN012494
ATGATGAAAAAACAATCACACTATTAACTGCATTACTCCCGCTTGCTTCTGCAGTTGCCGAAGAGCCAACCTTATC
ACCAGAAATGGTTTCAGCGTCTGAAGTGATCAGCACGCAAGAAAACCAAACCTATACCTATGTTCGCTGTTGGTAT
CGCACCAGCTACTCGAAAGATGATCCGGCGACCGATTGGGAATGGGCAAAAACGAAGATGGTAGCTACTTCACCA
TTGACGGCTACTGGTGGAGCTCCGTTTCATTTAAAAACATGTTCTACACCAACACGTCGCAAAACGTTATCCGTCA
GCGTTGTGAAGCAACATTAGATTTGACGAACGAGAACGCAGACATTACGTTCTTCGCCGCTGACAATCGCTTCTCA
TACAACCACACGATCTGGAGCAACGACGCAGCAATGCAGCCAGATCAAATCAACAAAGTGGTTGCACTCGGTGAC
AGCTTGTCTGATACAGGCAACATCTTTAACGCATCACAATGGCGCTTCCCTAACCCGAACAGCTGGTTCTTAGGTC
ACTTCTCCAACGGTTTTGTGTGGACAGAATACATTGCCAAAGCGAAGAACCTTCCGCTCTACAACTGGGCAGTTGG
CGGCGCGGCTGGTGAGAACCAATACATCGCGCTAACAGGGGTTGGTGAGCAAGTTTCTTCGTACTTAACCTACGCA
AAACTGGCGAAGAACTACAAACCAGCAAACACCTTGTTTACGCTTGAGTTTGGTTTGAATGACTTCATGAACTACA
ACCGTGGCGTTCCAGAAGTGAAAGCAGATTATGCAGAAGCACTGATTCGTTTGACGGACGCAGGTGCGAAGAACT
TCATGTTGATGACACTGCCAGACGCGACGAAAGCGCCTCAGTTTAAGTACTCAACACAAGAAGAGATCGACAAAT
TCGTGCGAAAGTGCTTGAGATGAACGAGTTCATCAAGGCACAAGCGATGTACTACAAAGCGCAAGGTTACAACAT
CACGTTGTTTGATACTCACGCCTTGTTCGAGACGCTAACTTCTGCGCCCGAAGAGCACGGTTTCGTGAACGCGAGT
GATCCTTGTTTGGACATCAACCGCTCATCGTCTGTCGATTACATGTACACCCACGCATTGCGCTCTGAGTGTGCTGC
GTCTGGTGCTGAGAAGTTTGTGTTCTGGGATGTCACGCATCCAACAACAGCAACTCACCGCTATGTTGCAGAGAAA
ATGCTAGAAAGTAGCAACAACTTAGCAGAGTACCGTTTCTAA

>CFSAN007458
ATGATGAAAAAAACAATCACACTATTAACTGCATTACTCCCGCTTGCTTCTGCAGTTGCCGAAGAGCCAA
CCTTATCACCAGAAATGGTTTCAGCGTCTGAAGTGATCAGCACGCAAGAAAACCAAACCTATACCTATGT
TCGCTGTTGGTATCGCACCAGCTACTCGAAAGATGATCCGGCGACCGATTGGGAATGGGCAAAAAACGAA
GATGGTAGCTACTTCACCATTGACGGCTACTGGTGGAGCTCCGTTTCATTTAAAAACATGTTCTACACCA
ACACGTCGCAAAACGTTATCCGTCAGCGTTGTGAAGCAACATTAGATTTGGCGAACGAGAACGCAGATAT
TACGTTCTTCGCCGCTGACAATCGCTTCTCATACAACCACACGATCTGGAGCAACGACGCAGCAATGCAG
CCAGATCAAATCAACAAAGTGGTTGCACTCGGTGACAGCTTGTCTGATACAGGCAACATCTTTAACGCAT
CACAATGGCGCTTCCCTAACCCGAACAGCTGGTTCTTAGGTCACTTCTCCAACGGTTTTGTGTGGACAGA
ATACATTGCCAAAGCGAAGAACCTTCCGCTCTACAACTGGGCAGTTGGCGGCGCGGCTGGTGAGAACCAA
TACATCGCGCTAACAGGGGTTGGTGATCAAGTTTCTTCGTACTTAACCTACGCAAAACAGGCGAAGAACT
ACAAACCAGCAAACACCTTGTTTACGCTTGAGTTTGGTTTGAATGACTTCATGAACTACAACCGTGGCGT
TCCAGAAGTGAAAGCGGATTATGCAGAAGCACTGATTCGTTTGACGGACGCAGGTGCGAAGAACTTCATG
TTGATGACACTGCCAGACGCGACGAAAGCGCCTCAGTTTAAGTACTCAACACAAGAAGAGATCGACAAAA
TTCGTGCGAAAGTGCTTGAGATGAACGAGTTCATCAAGGCACAAGCGATGTACTACAAAGCGCAAGGTTA
CAACATCACGTTGTTTGATACTCACGCCTTGTTCGAGACGCTAACTTCTGCGCCAGAAGAGCACGGTTTC
GTGAACGCGAGCGATCCTTGTTTGGACATCAACCGCTCATCGTCTGTCGATTACATGTACACCCACGCAT
TGCGCTCTGAGTGTGCAGCGTCTGGTGCTGAGAAGTTTGTGTTCTGGGATGTGACTCACCCAACTACAGC
CACGCATCGTTATGTTGCTGAAAAAATGTTGGAAAGCAGCAATAACTTAGAAGAGTTTCGCTTTTAA

>CFSAN007429
ATGATGAAAAAAAACAATCACACTATTAACTGCATTACTCCCGCTTGCTTCTGCAGTTGCCGAAGAGCCA
ACCTTATCACCAGAAATGGTTTCAGCGTCTGAAGTGATCAGCACGCAAGAAAACCAAACCTATACCTATG
TTCGCTGTTGGTATCGCACCAGCTACTCGAAAGATGATCCGGCGACCGATTGGGAATGGGCAAAAAACGA
AGATGGTAGCTACTTCACCATTGACGGCTACTGGTGGAGCTCCGTTTCATTTAAAAACATGTTCTACACC
AACACGTCGCAAAACGTTATCCGTCAGCGTTGTGAAGCAACATTAGATTTGGCGAACGAGAACGCAGACA

TTACGTTCTTCGCCGCTGACAATCGCTTCTCATACAACCACACGATCTGGAGCAACGACGCAGCAATGCA
GCCAGATCAAATCAACAAAGTGGTTGCACTCGGTGACAGCTTGTCTGATACAGGCAACATCTTTAACGCA
TCACAATGGCGCTTCCCTAACCCGAACAGCTGGTTCTTAGGTCACTTCTCCAACGGTTTTGTGTGGACAG
AATACATTGCCAAAGCGAAGAACCTTCCGCTCTACAACTGGGCAGTTGGCGGCGCGGCTGGTGAGAACCA
ATACATCGCGCTAACAGGGTTGGTGATCAAGTTTCTTCGTACTTAACCTACGCAAAACTGGCGAAGAACT
ACAAACCAGCAAACACCTTGTTTACGCTTGAGTTTGGTTTGAATGACTTCATGAACTACAACCGTGGCGT
TCCAGAAGTGAAAGCGGATTATGCAGAAGCACTGATTCGTTTGACGGACGCAGGTGCGAAGAACTTCATG
TTGATGACACTGCCAGACGCGACGAAAGCGCCTCAGTTTAAGTACTCAACACAAGAAGAGATCGACAAAA
TTCGTGCGAAAGTGCTTGAGATGAACGAGTTCATCAAGGCACAAGCGATGTACTACAAAGCGCAAGGTTA
CAACATCACGTTGTTTGATACTCACGCCTTGTTCGAGACGCTAACTTCTGCGCCAGAAGAGCACGGTTTC
GTGAACGCGAGCGATCCTTGTTTGGACATCAACCGCTCATCGTCTGTCGATTACATGTACACCCACGCAT
TGCGCTCTGAGTGTGCAGCGTCTGGTGCTGAGAAGTTTGTGTTCTGGGATGTCACGCATCCAACAACAGC
AACTCACCGCTATGTTGCAGAGAAAATGCTAGAAAGTAGCAACAACTTAGCAGAGTACCGTTTCTAA

>CFSAN007430
ATGATGAAAAAAACAATCACACTATTAACTGCATTACTCCCGCTTGCTTCTGCAGTTGCCGAAGAGCCAACCTTAT
CACCAGAAATGGTTTCAGCGTCTGAAGTGATCAGCACGCAAGAAAACCAAACCTATACCTATGTTCGCTGTTGGTA
TCGCACCAGCTACTCGAAAGATGATCCGGCGACCGATTGGGAATGGGCAAAAAACGAAGATGGTAGCTACTTCAC
CATTGACGGCTACTGGTGGAGCTCCGTTTCATTTAAAAACATGTTCTACACCAACACGTCGCAAAACGTTATCCGT
CAGCGTTGTGAAGCAACATTAGATTTGGCGAACGAGAACGCAGACATTACGTTCTTCGCCGCTGACAATCGCTTCT
CATACAACCACACGATCTGGAGCAACGACGCAGCAATGCAGCCAGATCAAATCAACAAAGTGGTTGCACTCGGTG
ACAGCTTGTCTGATACAGGCAACATCTTTAACGCATCACAATGGCGCTTCCCTAACCCGAACAGCTGGTTCTTAGG
TCACTTCTCCAACGGTTTTGTGTGGACAGAATACATTGCCAAAGCGAAGAACCTTCCGCTCTACAACTGGGCAGTT
GGCGGCGCGGCTGGTGAGAACCAATACATCGCGCTAACAGGGTTGGTGATCAAGTTTCTTCGTACTTAACCTACGC
AAAACTGGCGAAGAACTACAAACCAGCAAACACCTTGTTTACGCTTGAGTTTGGTTTGAATGACTTCATGAACTAC
AACCGTGGCGTTCCAGAAGTGAAAGCGGATTATGCAGAAGCACTGATTCGTTTGACGGACGCAGGTGCGAAGAAC
TTCATGTTGATGACACTGCCAGACGCGACGAAAGCGCCTCAGTTTAAGTACTCAACACAAGAAGAGATCGACAAA
ATTCGTGCGAAAGTGCTTGAGATGAACGAGTTCATCAAGGCACAAGCGATGTACTACAAAGCGCAAGGTTACAAC
ATCACGTTGTTTGATACTCACGCCTTGTTCGAGACGCTAACTTCTGCGCCAGAAGAGCACGGTTTCGTGAACGCGA
GCGATCCTTGTTTGGACATCAACCGCTCATCGTCTGTCGATTACATGTACACCCACGCATTGCGCTCTGAGTGTGCA
GCGTCTGGTGCTGAGAAGTTTGTGTTCTGGGATGTCACGCATCCAACAACAGCAACTCACCGCTATGTTGCAGAGA
AAATGCTAGAAAGTAGCAACAACTTAGCAGAGTACCGTTTCTAA

>CFSAN007432
ATGATGAAAAAAACAATCACACTATTAACTGCATTACTCCCGCTTGCTTCTGCAGTTGCCGAAGAGCCAA
CCTTATCACCAGAAATGGTTTCAGCGTCTGAAGTGATCAGCACGCAAGAAAACCAAACCTATACCTATGT
TCGCTGTTGGTATCGCACCAGCTACTCGAAAGATGATCCGGCGACCGATTGGGAATGGGCAAAAAACGAA
GATGGTAGCTACTTCACCATTGACGGCTACTGGTGGAGCTCCGTTTCATTTAAAAACATGTTCTACACCA
ACACGTCGCAAAACGTTATCCGTCAGCGTTGTGAAGCAACATTAGATTTGGCGAACGAGAACGCAGACAT
TACGTTCTTCGCCGCTGACAATCGCTTCTCATACAACCACACGATCTGGAGCAACGACGCAGCAATGCAG
CCAGATCAAATCAACAAAGTGGTTGCACTCGGTGACAGCTTGTCTGATACAGGCAACATCTTTAACGCAT
CACAATGGCGCTTCCCTAACCCGAACAGCTGGTTCTTAGGTCACTTCTCCAACGGTTTTGTGTGGACAGA
ATACATTGCCAAAGCGAAGAACCTTCCGCTCTACAACTGGGCAGTTGGCGGCGCGGCTGGTGAGAACCAA
TACATCGCGCTAACAGGGGTTGGTGATCAAGTTTCTTCGTACTTAACCTACGCAAAACTGGCGAAGAACT
ACAAACCAGCAAACACCTTGTTTACGCTTGAGTTTGGTTTGAATGACTTCATGAACTACAACCGTGGCGT
TCCAGAAGTGAAAGCGGATTATGCAGAAGCACTGATTCGTTTGACGGACGCAGGTGCGAAGAACTTCATG
TTGATGACACTGCCAGACGCGACGAAAGCGCCTCAGTTTAAGTACTCAACACAAGAAGAGATCGACAAAA
TTCGTGCGAAAGTGCTTGAGATGAACGAGTTCATCAAGGCACAAGCGATGTACTACAAAGCGCAAGGTTA
CAACATCACGTTGTTTGATACTCACGCCTTGTTCGAGACGCTAACTTCTGCGCCAGAAGAGCACGGTTTC
GTGAACGCGAGCGATCCTTGTTTGGACATCAACCGCTCATCGTCTGTCGATTACATGTACACCCACGCAT
TGCGCTCTGAGTGTGCAGCGTCTGGTGCTGAGAAGTTTGTGTTCTGGGATGTCACGCATCCAACAACAGC

AACTCACCGCTATGTTGCAGAGAAAATGCTAGAAAGTAGCAACAACTTAGCAGAGTACCGTTTCTAA

>CFSAN007433
ATGATGAAAAAACAATCACACTATTAACTGCATTACTCCCGCTTGCTTCTGCAGTTGCCGAAGAGCCAACCTTATC
ACCAGAAATGGTTTCAGCGTCTGAAGTGATCAGCACGCAAGAAAACCAAACCTATACCTATGTTCGCTGTTGGTAT
CGCACCAGCTACTCGAAAGATGATCCGGCGACCGATTGGGAATGGGCAAAAAACGAAGATGGTAGCTACTTCACC
ATTGACGGCTACTGGTGGAGCTCCGTTTCATTTAAAAACATGTTCTACACCAACACGTCGCAAAACGTTATCCGTC
AGCGTTGTGAAGCAACATTAGATTTGGCGAACGAGAACGCAGACATTACGTTCTTCGCCGCTGACAATCGCTTCTC
ATACAACCACACGATCTGGAGCAACGACGCAGCAATGCAGCCAGATCAAATCAACAAAGTGGTTGCACTCGGTGA
CAGCTTGTCTGATACAGGCAACATCTTTAACGCATCACAATGGCGCTTCCCTAACCCGAACAGCTGGTTCTTAGGT
CACTTCTCCAACGGTTTTGTGTGGACAGAATACATTGCCAAAGCGAAGAACCTTCCGCTCTACAACTGGGCAGTTG
GCGGCGCGGCTGGTGAGAACCAATACATCGCGCTAACAGGGTTGGTGATCAAGTTTCTTCGTACTTAACCTACGCA
AAACTGGCGAAGAACTACAAACCAGCAAACACCTTGTTTACGCTTGAGTTTGGTTTGAATGACTTCATGAACTACA
ACCGTGGCGTTCCAGAAGTGAAAGCGGATTATGCAGAAGCACTGATTCGTTTGACGGACGCAGGTGCGAAGAACT
TCATGTTGATGACACTGCCAGACGCGACGAAAGCGCCTCAGTTTAAGTACTCAACACAAGAAGAGATCGACAAAA
TTCGTGCGAAAGTGCTTGAGATGAACGAGTTCATCAAGGCACAAGCGATGTACTACAAAGCGCAAGGTTACAACA
TCACGTTGTTTGATACTCACGCCTTGTTCGAGACGCTAACTTCTGCGCCAGAAGAGCACGGTTTCGTGAACGCGAG
CGATCCTTGTTTGGACATCAACCGCTCATCGTCTGTCGATTACATGTACACCCACGCATTGCGCTCTGAGTGTGCAG
CGTCTGGTGCTGAGAAGTTTGTGTTCTGGGATGTCACGCATCCAACAACAGCAACTCACCGCTATGTTGCAGAGAA
AATGCTAGAAAGTAGCAACAACTTAGCAGAGTACCGTTTCTAA

>CFSAN007434
ATGATGAAAAAACAATCACACTATTAACTGCATTACTCCCGCTTGCTTCTGCAGTTGCCGAAGAGCCAACCTTATC
ACCAGAAATGGTTTCAGCGTCTGAAGTGATCAGCACGCAAGAAAACCAAACCTATACCTATGTTCGCTGTTGGTAT
CGCACCAGCTACTCGAAAGATGATCCGGCGACCGATTGGGAATGGGCAAAAAACGAAGATGGTAGCTACTTCACC
ATTGACGGCTACTGGTGGAGCTCCGTTTCATTTAAAAACATGTTCTACACCAACACGTCGCAAAACGTTATCCGTC
AGCGTTGTGAAGCAACATTAGATTTGGCGAACGAGAACGCAGACATTACGTTCTTCGCCGCTGACAATCGCTTCTC
ATACAACCACACGATCTGGAGCAACGACGCAGCAATGCAGCCAGATCAAATCAACAAAGTGGTTGCACTCGGTGA
CAGCTTGTCTGATACAGGCAACATCTTTAACGCATCACAATGGCGCTTCCCTAACCCGAACAGCTGGTTCTTAGGT
CACTTCTCCAACGGTTTTGTGTGGACAGAATACATTGCCAAAGCGAAGAACCTTCCGCTCTACAACTGGGCAGTTG
GCGGCGCGGCTGGTGAGAACCAATACATCGCGCTAACAGGGGTTGGTGATCAAGTTTCTTCGTACTTAACCTACGC
AAAACTGGCGAAGAACTACAAACCAGCAAACACCTTGTTTACGCTTGAGTTTGGTTTGAATGACTTCATGAACTAC
AACCGTGGCGTTCCAGAAGTGAAAGCGGATTATGCAGAAGCACTGATTCGTTTGACGGACGCAGGTGCGAAGAAC
TTCATGTTGATGACACTGCCAGACGCGACGAAAGCGCCTCAGTTTAAGTACTCAACACAAGAAGAGATCGACAAA
ATTCGTGCGAAAGTGCTTGAGATGAACGAGTTCATCAAGGCACAAGCGATGTACTACAAAGCGCAAGGTTACAAC
ATCACGTTGTTTGATACTCACGCCTTGTTCGAGACGCTAACTTCTGCGCCAGAAGAGCACGGTTTCGTGAACGCGA
GCGATCCTTGTTTGGACATCAACCGCTCATCGTCTGTCGATTACATGTACACCCACGCATTGCGCTCTGAGTGTGCA
GCGTCTGGTGCTGAGAAGTTTGTGTTCTGGGATGTCACGCATCCAACAACAGCAACTCACCGCTATGTTGCAGAGA
AAATGCTAGAAAGTAGCAACAACTTAGCAGAGTACCGTTTCTAA

>CFSAN007435
ATGATGAAAAAAAACAATCACACTATTAACTGCATTACTCCCGCTTGCTTCTGCAGTTGCCGAAGAGCCAA
CCTTATCACCAGAAATGGTTTCAGCGTCTGAAGTGATCAGCACGCAAGAAAACCAAACCTATACCTATGT
TCGCTGTTGGTATCGCACCAGCTACTCGAAAGATGATCCGGCGACCGATTGGGAATGGGCAAAAAACGAA
GATGGTAGCTACTTCACCATTGACGGCTACTGGTGGAGCTCCGTTTCATTTAAAAACATGTTCTACACCA
ACACGTCGCAAAACGTTATCCGTCAGCGTTGTGAAGCAACATTAGATTTGGCGAACGAGAACGCAGACAT
TACGTTCTTCGCCGCTGACAATCGCTTCTCATACAACCACACGATCTGGAGCAACGACGCAGCAATGCAG
CCAGATCAAATCAACAAAGTGGTTGCACTCGGTGACAGCTTGTCTGATACAGGCAACATCTTTAACGCAT
CACAATGGCGCTTCCCTAACCCGAACAGCTGGTTCTTAGGTCACTTCTCCAACGGTTTTGTGTGGACAGA
ATACATTGCCAAAGCGAAGAACCTTCCGCTCTACAACTGGGCAGTTGGCGGCGCGGCTGGTGAGAACCAA
TACATCGCGCTAACAGGGGTTGGTGATCAAGTTTCTTCGTACTTAACCTACGCAAAACTGGCGAAGAACT

ACAAACCAGCAAACACCTTGTTTACGCTTGAGTTTGGTTTGAATGACTTCATGAACTACAACCGTGGCGT
TCCAGAAGTGAAAGCGGATTATGCAGAAGCACTGATTCGTTTGACGGACGCAGGTGCGAAGAACTTCATG
TTGATGACACTGCCAGACGCGACGAAAGCGCCTCAGTTTAAGTACTCAACACAAGAAGAGATCGACAAAA
TTCGTGCGAAAGTGCTTGAGATGAACGAGTTCATCAAGGCACAAGCGATGTACTACAAAGCGCAAGGTTA
CAACATCACGTTGTTTGATACTCACGCCTTGTTCGAGACGCTAACTTCTGCGCCAGAAGAGCACGGTTTC
GTGAACGCGAGCGATCCTTGTTTGGACATCAACCGCTCATCGTCTGTCGATTACATGTACACCCACGCAT
TGCGCTCTGAGTGTGCAGCGTCTGGTGCTGAGAAGTTTGTGTTCTGGGATGTCACGCATCCAACAACAGC
AACTCACCGCTATGTTGCAGAGAAAATGCTAGAAAGTAGCAACAACTTAGCAGAGTACCGTTTCTAA

>CFSAN001618
ATGATGAAAAAAACAATCACACTATTAACTGCATTACTCCCGCTTGCTTCTGCAGTTGCCGAAGAGCCAACCTTAT
CACCAGAAATGGTTTCAGCGTCTGAAGTGATCAGCACGCAAGAAAACCAAACCTATACCTATGTTCGCTGTTGGTA
TCGCACCAGCTACTCGAAAGATGATCCGGCGACCGATTGGGAATGGGCAAAAAACGAAGATGGTAGCTACTTCAC
CATTGACGGCTACTGGTGGAGCTCCGTTTCACTTAAAAACATGTTCTACACCAACACGTCGCAAAACGTTATCCGT
CAGCGTTGTGAAGCAACATTAGATTTGGCGAACGAGAACGCAGACATTACGTTCTTCGCCGCTGACAATCGCTTCT
CATACAACCACACGATCTGGAGCAACGACGCAGCAATGCAGCCAGATCAAATCAACAAAGTGGTTGCACTCGGTG
ACAGCTTGTCTGATACAGGCAACATCTTTAACGCATCACAATGGCGCTTCCCTAACCCGAACAGCTGGTTCTTAGG
TCACTTCTCCAACGGTTTTGTGTGGACAGAATACATTGCCAAAGCGAAGAACCTTCCGCTCTACAACTGGGCAGTT
GGCGGCGCGGCTGGTGAGAACCAATACATCGCGCTAACAGGGGTTGGTGATCAAGTTTCTTCGTACTTAACCTACG
CAAAACTGGCGAAGAACTACAAACCAGCAAACACCTTGTTTACGCTTGAGTTTGGTTTGAATGACTTCATGAACTA
CAACCGTGGCGTTCCAGAAGTGAAAGCAGATTATGCAGAAGCACTGATTCGTTTGACGGACGCAGGTGCGAAGAA
CTTCATGTTGATGACACTGCCAGACGCGACGAAAGCGCCTCAGTTTAAGTACTCAACACAAGAAGAGATCGACAA
AATTCGTGCGAAAGTGCTTGAGATGAACGAGTTCATCAAGGCACAAGCGATGTACTACAAAGCGCAAGGTTACAA
CATCACGTTGTTTGATACTCACGCCTTGTTCGAGACGCTAACTTCTGCGCCAGAAGAGCACGGTTTCGTGAACGCG
AGCGATCCTTGTTTGGACATCAACCGCTCATCGTCTGTCGATTACATGTACACCCACGCATTGCGCTCTGAGTGTGC
AGCGTCTGGTGCTGAGAAGTTTGTGTTCTGGGATGTCACGCATCCAACAACAGCAACTCACCGCTATGTTGCAGAG
AAAATGCTAGAAAGTAGCAACAACTTAGCCGAGTACCGTTTCTAA

>CFSAN001619
ATGATGAAAAAAACAATCACACTATTAACTGCATTACTCCCGCTTGCTTCTGCAGTTGCCGAAGAGCCAACCTTAT
CACCAGAAATGGTTTCAGCGTCTGAAGTGATCAGCACGCAAGAAAACCAAACCTATACCTATGTTCGCTGTTGGTA
TCGCACCAGCTACTCGAAAGATGATCCGGCGACCGATTGGGAATGGGCAAAAAACGAAGATGGTAGCTACTTCAC
CATTGACGGCTACTGGTGGAGCTCCGTTTCACTTAAAAACATGTTCTACACCAACACGTCGCAAAACGTTATCCGT
CAGCGTTGTGAAGCAACATTAGATTTGGCGAACGAGAACGCAGACATTACGTTCTTCGCCGCTGACAATCGCTTCT
CATACAACCACACGATCTGGAGCAACGACGCAGCAATGCAGCCAGATCAAATCAACAAAGTGGTTGCACTCGGTG
ACAGCTTGTCTGATACAGGCAACATCTTTAACGCATCACAATGGCGCTTCCCTAACCCGAACAGCTGGTTCTTAGG
TCACTTCTCCAACGGTTTTGTGTGGACAGAATACATTGCCAAAGCGAAGAACCTTCCGCTCTACAACTGGGCAGTT
GGCGGCGCGGCTGGTGAGAACCAATACATCGCGCTAACAGGGGTTGGTGATCAAGTTTCTTCGTACTTAACCTACG
CAAAACTGGCGAAGAACTACAAACCAGCAAACACCTTGTTTACGCTTGAGTTTGGTTTGAATGACTTCATGAACTA
CAACCGTGGCGTTCCAGAAGTGAAAGCAGATTATGCAGAAGCACTGATTCGTTTGACGGACGCAGGTGCGAAGAA
CTTCATGTTGATGACACTGCCAGACGCGACGAAAGCGCCTCAGTTTAAGTACTCAACACAAGAAGAGATCGACAA
AATTCGTGCGAAAGTGCTTGAGATGAACGAGTTCATCAAGGCACAAGCGATGTACTACAAAGCGCAAGGTTACAA
CATCACGTTGTTTGATACTCACGCCTTGTTCGAGACGCTAACTTCTGCGCCAGAAGAGCACGGTTTCGTGAACGCG
AGCGATCCTTGTTTGGACATCAACCGCTCATCGTCTGTCGATTACATGTACACCCACGCATTGCGCTCTGAGTGTGC
AGCGTCTGGTGCTGAGAAGTTTGTGTTCTGGGATGTCACGCATCCAACAACAGCAACTCACCGCTATGTTGCAGAG
AAAATGCTAGAAAGTAGCAACAACTTAGCCGAGTACCGTTTCTAA

>CFSAN001620
ATGATGAAAAAAACAATCACACTATTAACTGCATTACTCCCGCTTGCTTCTGCAGTTGCCGAAGAGCCAA
CCTTATCACCAGAAATGGTTTCAGCGTCTGAAGTGATCAGCACGCAAGAAAACCAAACCTATACCTATGT
TCGCTGTTGGTATCGCACCAGCTACTCGAAAGATGATCCGGCGACCGATTGGGAATGGGCAAAAAACGAA

GATGGTAGCTACTTCACCATTGACGGCTACTGGTGGAGCTCCGTTTCACTTAAAAACATGTTCTACACCA
ACACGTCGCAAAACGTTATCCGTCAGCGTTGTGAAGCAACATTAGATTTGGCGAACGAGAACGCAGACAT
TACGTTCTTCGCCGCTGACAATCGCTTCTCATACAACCACACGATCTGGAGCAACGACGCAGCAATGCAG
CCAGATCAAATCAACAAAGTGGTTGCACTCGGTGACAGCTTGTCTGATACAGGCAACATCTTTAACGCAT
CACAATGGCGCTTCCCTAACCCGAACAGCTGGTTCTTAGGTCACTTCTCCAACGGTTTTGTGTGGACAGA
ATACATTGCCAAAGCGAAGAACCTTCCGCTCTACAACTGGGCAGTTGGCGGCGCGGCTGGTGAGAACCAA
TACATCGCGCTAACAGGGGTTGGTGATCAAGTTTCTTCGTACTTAACCTACGCAAAACTGGCGAAGAACT
ACAAACCAGCAAACACCTTGTTTACGCTTGAGTTTGGTTTGAATGACTTCATGAACTACAACCGTGGCGT
TCCAGAAGTGAAAGCAGATTATGCAGAAGCACTGATTCGTTTGACGGACGCAGGTGCGAAGAACTTCATG
TTGATGACACTGCCAGACGCGACGAAAGCGCCTCAGTTTAAGTACTCAACACAAGAAGAGATCGACAAAA
TTCGTGCGAAAGTGCTTGAGATGAACGAGTTCATCAAGGCACAAGCGATGTACTACAAAGCGCAAGGTTA
CAACATCACGTTGTTTGATACTCACGCCTTGTTCGAGACGCTAACTTCTGCGCCAGAAGAGCACGGTTTC
GTGAACGCGAGCGATCCTTGTTTGGACATCAACCGCTCATCGTCTGTCGATTACATGTACACCCACGCAT
TGCGCTCTGAGTGTGCAGCGTCTGGTGCTGAGAAGTTTGTGTTCTGGGATGTCACGCATCCAACAACAGC
AACTCACCGCTATGTTGCAGAGAAAATGCTAGAAAGTAGCAACAACTTAGCCGAGTACCGTTTCTAA

>K1203
ATGATGAAAAAAACAATCACACTATTAACTGCATTACTCCCGCTTGCTTCTGCAGTTGCCGAAGAGCCAA
CCTTATCACCAGAAATGGTTTCAGCGTCTGAAGTGATCAGCACGCAAGAAAACCAAACCTATACCTATGT
TCGCTGTTGGTATCGCACCAGCTACTCGAAAGATGATCCGGCGACCGATTGGGAATGGGCAAAAAACGAA
GATGGTAGCTACTTCACCATTGACGGCTACTGGTGGAGCTCCGTTTCACTTAAAAACATGTTCTACACCA
ACACGTCGCAAAACGTTATCCGTCAGCGTTGTGAAGCAACATTAGATTTGGCGAACGAGAACGCAGACAT
TACGTTCTTCGCCGCTGACAATCGCTTCTCATACAACCACACGATCTGGAGCAACGACGCAGCAATGCAG
CCAGATCAAATCAACAAAGTGGTTGCACTCGGTGACAGCTTGTCTGATACAGGCAACATCTTTAACGCAT
CACAATGGCGCTTCCCTAACCCGAACAGCTGGTTCTTAGGTCACTTCTCCAACGGTTTTGTGTGGACAGA
ATACATTGCCAAAGCGAAGAACCTTCCGCTCTACAACTGGGCAGTTGGCGGCGCGGCTGGTGAGAACCAA
TACATCGCGCTAACAGGGGTTGGTGATCAAGTTTCTTCGTACTTAACCTACGCAACACTGGCGAAGAACT
ACAAACCAGCAAACACCTTGTTTACGCTTGAGTTTGGTTTGAATGACTTCATGAACTACAACCGTGGCGT
TCCAGAAGTGAAAGCAGATTATGCAGAAGCACTGATTCGTTTGACGGACGCAGGTGCGAAGAACTTCATG
TTGATGACACTGCCAGACGCGACGAAAGCGCCTCAGTTTAAGTACTCAACACAAGAAGAGATCGACAAAA
TTCGTGCGAAAGTGCTTGAGATGAACGAGTTCATCAAGGCACAAGCGATGTACTACAAAGCGCAAGGTTA
CAACATCACGTTGTTTGATACTCACGCCTTGTTCGAGACGCTAACTTCTGCGCCAGAAGAGCACGGTTTC
GTGAACGCGAGCGATCCTTGTTTGGACATCAACCGCTCATCGTCTGTCGATTACATGTACACCCACGCAT
TGCGCTCTGAGTGTGCAGCGTCTGGTGCTGAGAAGTTTGTGTTCTGGGATGTCACGCATCCAACAACAGC
AACTCACCGCTATGTTGCAGAGAAAATGCTAGAAAGTAGCAACAACTTAGCCGAGTACCGTTTCTAA

>98-513-F52
ATGATGAAAAAAACAATCACACTATTAACTGCATTACTCCCGCTTGCTTCTGCAGTTGCCGAAGAGCCAACCTTAT
CACCAGAAATGGTTTCAGCGTCTGAAGTGATCAGCACGCAAGAAAACCAAACCTATACCTATGTTCGCTGTTGGTA
TCGCACCAGCTACTCGAAAGATGATCCGGCGACCGATTGGGAATGGGCAAAAAACGAAGATGGTAGCTACTTCAC
CATTGACGGCTACTGGTGGAGCTCCGTTTCATTTAAAAACATGTTCTACACCAACACGTCGCAAAACGTTATCCGT
CAGCGTTGTGAAGCAACATTAGATTTGGCGAACGAGAACGCAGATATTACGTTCTTCGCCGCTGACAATCGCTTCT
CATACAACCACACGATCTGGAGCAACGACGCAGCAATGCAGCCAGATCAAATCAACAAAGTGGTTGCACTCGGTG
ACAGCTTGTCTGATACAGGCAACATCTTTAACGCATCACAATGGCGCTTCCCTAACCCGAACAGCTGGTTCTTAGG
TCACTTCTCCAACGGTTTTGTGTGGACAGAATACATTGCCAAAGCGAAGAACCTTCCGCTCTACAACTGGGCAGTT
GGCGGCGCGGCTGGTGAGAACCAATACATCGCGCTAACAGGGGTTGGTGATCAAGTTTCTTCGTACTTAACCTACG
CAAAACAGGCGAAGAACTACAAACCAGCAAACACCTTGTTTACGCTTGAGTTTGGTTTGAATGACTTCATGAACTA
CAACCGTGGCGTTCCAGAAGTGAAAGCGGATTATGCAGAAGCACTGATTCGTTTGACGGACGCAGGTGCGAAGAA
CTTCATGTTGATGACACTGCCAGACGCGACGAAAGCGCCTCAGTTTAAGTACTCAACACAAGAAGAGATCGACAA
AATTCGTGCGAAAGTGCTTGAGATGAACGAGTTCATCAAGGCACAAGCGATGTACTACAAAGCGCAAGGTTACAA
CATCACGTTGTTTGATACTCACGCCTTGTTCGAGACGCTAACTTCTGCGCCAGAAGAGCACGGTTTCGTGAACGCG

AGCGATCCTTGTTTGGACATCAACCGCTCATCGTCTGTCGATTACATGTACACCCACGCATTGCGCTCTGAGTGTGC
AGCGTCTGGTGCTGAGAAGTTTGTGTTCTGGGATGTGACTCACCCAACTACAGCCACGCATCGTTATGTTGCTGAA
AAAATGTTGGAAAGCAGCAATAACTTAGAAGAGTTTCGCTTTTAA

>CFSAN012491
ATGATGAAAAAAAACAATCACACTATTAACTGCATTACTCCCGCTTGCTTCTGCAGTTGCCGAAGAGCCA
ACCTTATCACCAGAAATGGTTTCAGCGTCTGAAGTGATCAGCACGCAAGAAAACCAAACCTATACCTATG
TTCGCTGTTGGTATCGCACCAGCTACTCGAAAGATGATCCGGCGACCGATTGGGAATGGGCAAAAAACGA
AGATGGTAGCTACTTCACCATTGACGGCTACTGGTGGAGCTCCGTTTCATTTAAAAACATGTTCTACACC
AACACGTCGCAAAACGTTATCCGTCAGCGTTGTGAAGCAACATTAGATTTGACGAACGAGAACGCAGACA
TTACGTTCTTCGCCGCTGACAATCGCTTCTCATACAACCACACGATCTGGAGCAACGACGCAGCAATGCA
GCCAGATCAAATCAACAAAGTGGTTGCACTCGGTGACAGCTTGTCTGATACAGGCAACATCTTTAACGCA
TCACAATGGCGCTTCCCTAACCCGAACAGCTGGTTCTTAGGTCACTTCTCCAACGGTTTTGTGTGGACAG
AATACATTGCCAAAGCGAAGAACCTTCCGCTCTACAACTGGGCAGTTGGCGGCGCGGCTGGTGAGAACCA
ATACATCGCGCTAACAGGGGTTGGTGAGCAAGTTTCTTCGTACTTAACCTACGCAAAACTGGCGAAGAAC
TACAAACCAGCAAACACCTTGTTTACGCTTGAGTTTGGTTTGAATGACTTCATGAACTACAACCGTGGCG
TTCCAGAAGTGAAAGCAGATTATGCAGAAGCACTGATTCGTTTGACGGACGCAGGTGCGAAGAACTTCAT
GTTGATGACACTGCCAGACGCGACGAAAGCGCCTCAGTTTAAGTACTCAACACAAGAAGAGATCGACAAA
ATTCGTGCGAAAGTGCTTGAGATGAACGAGTTCATCAAGGCACAAGCGATGTACTACAAAGCGCAAGGTT
ACAACATCACGTTGTTTGATACTCACGCCTTGTTCGAGACGCTAACTTCTGCGCCCGAAGAGCACGGTTT
CGTGAACGCGAGTGATCCTTGTTTGGACATCAACCGCTCATCGTCTGTCGATTACATGTACACCCACGCA
TTGCGCTCTGAGTGTGCTGCGTCTGGTGCTGAGAAGTTTGTGTTCTGGGATGTCACGCATCCAACAACAG
CAACTCACCGCTATGTTGCAGAGAAAATGCTAGAAAGTAGCAACAACTTAGCAGAGTACCGTTTCTAA

>CFSAN012492
ATGATGAAAAAAAACAATCACACTATTAACTGCATTACTCCCGCTTGCTTCTGCAGTTGCCGAAGAGCCAACCTTA
TCACCAGAAATGGTTTCAGCGTCTGAAGTGATCAGCACGCAAGAAAACCAAACCTATACCTATGTTCGCTGTTGGT
ATCGCACCAGCTACTCGAAAGATGATCCGGCGACCGATTGGGAATGGGCAAAAAACGAAGATGGTAGCTACTTCA
CCATTGACGGCTACTGGTGGAGCTCCGTTTCATTTAAAAACATGTTCTACACCAACACGTCGCAAAACGTTATCCG
TCAGCGTTGTGAAGCAACATTAGATTTGACGAACGAGAACGCAGACATTACGTTCTTCGCCGCTGACAATCGCTTC
TCATACAACCACACGATCTGGAGCAACGACGCAGCAATGCAGCCAGATCAAATCAACAAAGTGGTTGCACTCGGT
GACAGCTTGTCTGATACAGGCAACATCTTTAACGCATCACAATGGCGCTTCCCTAACCCGAACAGCTGGTTCTTAG
GTCACTTCTCCAACGGTTTTGTGTGGACAGAATACATTGCCAAAGCGAAGAACCTTCCGCTCTACAACTGGGCAGT
TGGCGGCGCGGCTGGTGAGAACCAATACATCGCGCTAACAGGGGTTGGTGAGCAAGTTTCTTCGTACTTAACCTAC
GCAAAACTGGCGAAGAACTACAAACCAGCAAACACCTTGTTTACGCTTGAGTTTGGTTTGAATGACTTCATGAACT
ACAACCGTGGCGTTCCAGAAGTGAAAGCAGATTATGCAGAAGCACTGATTCGTTTGACGGACGCAGGTGCGAAGA
ACTTCATGTTGATGACACTGCCAGACGCGACGAAAGCGCCTCAGTTTAAGTACTCAACACAAGAAGAGATCGACA
AAATTCGTGCGAAAGTGCTTGAGATGAACGAGTTCATCAAGGCACAAGCGATGTACTACAAAGCGCAAGGTTACA
ACATCACGTTGTTTGATACTCACGCCTTGTTCGAGACGCTAACTTCTGCGCCCGAAGAGCACGGTTTCGTGAACGC
GAGTGATCCTTGTTTGGACATCAACCGCTCATCGTCTGTCGATTACATGTACACCCACGCATTGCGCTCTGAGTGTG
CTGCGTCTGGTGCTGAGAAGTTTGTGTTCTGGGATGTCACGCATCCAACAACAGCAACTCACCGCTATGTTGCAGA
GAAAATGCTAGAAAGTAGCAACAACTTAGCAGAGTACCGTTTCTAA

>CFSAN007446
ATGATGAAAAAAACAATCACACTATTAACTGCATTACTCCCGCTTGCTTCTGCAGTTGCCGAAGAGCCAA
CCTTATCACCAGAAATGGTTTCAGCGTCTGAAGTGATCAGCACGCAAGAAAACCAAACCTATACCTATGT
TCGCTGTTGGTATCGCACCAGCTACTCGAAAGATGATCCGGCGACCGATTGGGAATGGGCAAAAAACGAA
GATGGTAGCTACTTCACCATTGACGGCTACTGGTGGAGCTCCGTTTCATTTAAAAACATGTTCTACACCA
ACACGTCGCAAAACGTTATCCGTCAGCGTTGTGAAGCAACATTAGATTTGGCGAACGAGAACGCAGACAT
TACGTTCTTCGCCGCTGACAATCGCTTCTCATACAACCACACGATCTGGAGCAACGACGCAGCAATGCAG

CCAGATCAAATCAACAAAGTGGTTGCACTCGGTGACAGCTTGTCTGATACAGGCAACATCTTTAACGCAT
CACAATGGCGCTTCCCTAACCCGAACAGCTGGTTCTTAGGTCACTTCTCCAACGGTTTTGTGTGGACAGA
ATACATTGCCAAAGCGAAGAACCTTCCGCTCTACAACTGGGCTGTTGGCGGCGCGGCTGGTGAGAACCAA
TACATCGCGCTAACAGGGGTTGGTGATCAAGTTTCTTCGTACTTAACCTACGCAAAACTGGCGAAGAACT
ACAAACCAGCAAACACCTTGTTTACGCTTGAGTTTGGTTTGAATGACTTCATGAACTACAACCGTGGCGT
TCCAGAAGTGAAAGCAGATTATGCAGAAGCACTGATTCGTTTGACGGACGCAGGTGCGAAGAACTTCATG
TTGATGACACTGCCAGACGCGACGAAAGCGCCTCAGTTTAAGTACTCAACACAAGAAGAGATCGACAAAA
TTCGTGCGAAAGTGCTTGAGATGAACGAGTTCATCAAGGCACAAGCGATGTACTACAAAGCGCAAGGTTA
CAACATCACGTTGTTTGATACTCACGCCTTGTTCGAGACGCTAACTTCTGCGCCAGAAGAGCACGGTTTC
GTGAACGCGAGCGATCCTTGTTTGGACATCAACCGCTCATCGTCTGTCGACTACATGTACACCCACGCAT
TGCGCTCTGAGTGTGCGGCGTCTGGTGCTGAGAAGTTTGTATTCTGGGATGTCACGCACCCAACAACAGC
AACTCACCGCTATGTTGCAGAGAAAATGCTAGAAAGTAGCAACAACTTAGCCGAGTACCGTTTCTAA

>CFSAN007448
ATGATGAAAAAAACAATCACACTATTAACTGCATTACTCCCGCTTGCTTCTGCAGTTGCCGAAGAGCCAACCTTAT
CACCAGAAATGGTTTCAGCGTCTGAAGTGATCAGCACGCAAGAAAACCAAACCTATACCTATGTTCGCTGTTGGTA
TCGCACCAGCTACTCGAAAGATGATCCGGCGACCGATTGGGAATGGGCAAAAAACGAAGATGGTAGCTACTTCAC
CATTGACGGCTACTGGTGGAGCTCCGTTTCATTTAAAAAACATGTTCTACACCAACACGTCGCAAAACGTTATCCG
TCAGCGTTGTGAAGCAACATTAGATTTGGCGAACGAGAACGCAGACATTACGTTCTTCGCCGCTGACAATCGCTTC
TCATACAACCACACGATCTGGAGCAACGACGCAGCAATGCAGCCAGATCAAATCAACAAAGTGGTTGCACTCGGT
GACAGCTTGTCTGATACCGGCAACATCTTTAACGCATCACAATGGCGCTTCCCTAACCCGAACAGCTGGTTCTTAG
GTCACTTCTCCAACGGTTTTGTGTGGACAGAATACATTGCCAAAGCGAAGAACCTTCCGCTCTACAACTGGGCAGT
TGGCGGCGCGGCTGGTGAGAACCAATACATCGCGCTAACAGGGGTTGGTGATCAAGTTTCTTCGTACTTAACCTAC
GCAAAACTGGCGAAGAACTACAAACCAGCAAACACCTTGTTTACGCTTGAGTTTGGTTTGAATGACTTCATGAACT
ACAACCGTGGCGTTCCAGAAGTGAAAGCGGATTATGCAGAAGCACTGATTCGTTTGACGGACGCAGGTGCGAAGA
ACTTCATGTTGATGACACTGCCAGACGCGACGAAAGCGCCTCAGTTTAAGTACTCAACACAAGAAGAGATCGACA
AAATTCGTGCGAAAGTGCTTGAGATGAACGAGTTCATCAAGGCACAAGCGATGTACTACAAAGCGCAAGGTTACA
ACATCACGTTGTTTGATACTCACGCCTTGTTCGAGACGCTAACTTCTGCGCCAGAAGAGCACGGTTTCGTGAACGC
GAGTGATCCTTGTTTGGACATCAACCGCTCATCGTCTGTCGATTACATGTACACCCACGCATTGCGCTCTGAGTGTG
CAGCGTCTGGTGCTGAGAAATTTGTGTTCTGGGATGTCACGCATCCAACAACAGCAACTCACCGCTATGTTGCAGA
GAAAATGCTAGAAAGTAGCAACAACTTAGCAGAGTACCGTTTCTAA

>CFSAN007449
ATGATGAAAAAAAACAATCACACTATTAACTGCATTACTCCCGCTTGCTTCTGCAGTTGCCGAAGAGCCAACCTTA
TCACCAGAAATGGTTTCAGCGTCTGAAGTGATCAGCACGCAAGAAAACCAAACCTATACCTATGTTCGCTGTTGGT
ATCGCACCAGCTACTCGAAAGATGATCCAGCGACCGATTGGGAATGGGCAAAAAACGAAGATGGTAGCTACTTCA
CCATTGACGGCTACTGGTGGAGCTCCGTTTCATTTAAAAACATGTTCTACACCAACACGTCGCAAAACGTTATCCG
TCAGCGTTGTGAAGCAACATTAGATTTGGCGAACGAGAACGCAGACATTACGTTCTTCGCCGCTGACAATCGCTTC
TCATACAACCACACGATCTGGAGCAACGACGCAGCAATGCAGCCAGATCAAATCAACAAAGTGGTTGCACTCGGT
GACAGCTTGTCTGATACAGGCAACATCTTTAACGCATCACAATGGCGCTTCCCTAACCCGAACAGCTGGTTCTTAG
GTCACTTCTCCAACGGTTTTGTTTGGACAGAATACATTGCCAAAGCGAAGAACCTTCCGCTCTACAACTGGGCAGT
TGGCGGCGCGGCTGGTGAGAACCAATACATCGCGCTAACAGGGGTTGGTGAGCAAGTTTCTTCGTACTTAACCTAC
GCAAAACTGGCGAAGAACTACAAACCAGCAAACACCTTGTTTACGCTTGAGTTTGGTTTGAATGACTTCATGAACT
ACAACCGTGGCGTTCCAGAAGTGAAAGCGGATTATGCAGAAGCACTGATTCGTTTGACGGACGCAGGTGCGAAGA
ACTTCATGTTGATGACACTGCCAGATGCGACGAAAGCGCCTCAGTTTAAGTACTCAACACAAGAAGAGATCGACA
AAATTCGTGCGAAAGTGCTTGAGATGAACGAGTTCATCAAGGCACAAGCGATGTACTACAAAGCGCAAGGTTACA
ACATCACGTTGTTTGATACTCACGCCTTGTTCGAGACGCTAACTTCTGCGCCAGAAGAGCACGGTTTCGTGAACGC
GAGCGATCCTTGTTTGGACATCAACCGCTCATCGTCTGTCGATTACATGTACACCCACGCATTGCGCTCTGAGTGTG

CAGCGTCTGGTGCTGAGAAGTTTGTGTTCTGGGATGTCACGCATCCAACAACAGCAACTCACCGCTATGTTGCAGA
GAAAATGCTAGAAAGTAGCAACAACTTAGCCGAGTACCGTTTCTAA

>CFSAN007450
ATGATGAAAAAAACAATCACACTATTAACTGCATTACTCCCGCTTGCTTCTGCAGTTGCCGAAGAGCCAACCTTAT
CACCAGAAATGGTTTCAGCGTCTGAAGTGATCAGCACGCAAGAAAACCAAACCTATACCTATGTTCGCTGTTGGTA
TCGCACCAGCTACTCGAAAGATGATCCAGCGACCGATTGGGAATGGGCAAAAAACGAAGATGGTAGCTACTTCAC
CATTGACGGCTACTGGTGGAGCTCCGTTTCATTTAAAAACATGTTCTACACCAACACGTCGCAAAACGTTATCCGT
CAGCGTTGTGAAGCAACATTAGATTTGGCGAACGAGAACGCAGACATTACGTTCTTCGCCGCTGACAATCGCTTCT
CATACAACCACACGATCTGGAGCAACGACGCAGCAATGCAGCCAGATCAAATCAACAAAGTGGTTGCACTCGGTG
ACAGCTTGTCTGATACAGGCAACATCTTTAACGCATCACAATGGCGCTTCCCTAACCCGAACAGCTGGTTCTTAGG
TCACTTCTCCAACGGTTTTGTTTGGACAGAATACATTGCCAAAGCGAAGAACCTTCCGCTCTACAACTGGGCAGTT
GGCGGCGCGGCTGGTGAGAACCAATACATCGCGCTAACAGGGGTTGGTGAGCAAGTTTCTTCGTACTTAACCTACG
CAAAACTGGCGAAGAACTACAAACCAGCAAACACCTTGTTTACGCTTGAGTTTGGTTTGAATGACTTCATGAACTA
CAACCGTGGCGTTCCAGAAGTGAAAGCGGATTATGCAGAAGCACTGATTCGTTTGACGGACGCAGGTGCGAAGAA
CTTCATGTTGATGACACTGCCAGATGCGACGAAAGCGCCTCAGTTTAAGTACTCAACACAAGAAGAGATCGACAA
AATTCGTGCGAAAGTGCTTGAGATGAACGAGTTCATCAAGGCACAAGCGATGTACTACAAAGCGCAAGGTTACAA
CATCACGTTGTTTGATACTCACGCCTTGTTCGAGACGCTAACTTCTGCGCCAGAAGAGCACGGTTTCGTGAACGCG
AGCGATCCTTGTTTGGACATCAACCGCTCATCGTCTGTCGATTACATGTACACCCACGCATTGCGCTCTGAGTGTGC
AGCGTCTGGTGCTGAGAAGTTTGTGTTCTGGGATGTCACGCATCCAACAACAGCAACTCACCGCTATGTTGCAGAG
AAAATGCTAGAAAGTAGCAACAACTTAGCCGAGTACCGTTTCTAA

>CFSAN007452
ATGATGAAAAAAACAATCACACTATTAACTGCATTACTCCCGCTTGCTTCTGCAGTTGCCGAAGAGCCAA
CCTTATCACCAGAAATGGTTTCAGCGTCTGAAGTGATCAGCACGCAAGAAAACCAAACCTATACCTATGT
TCGCTGTTGGTATCGCACCAGCTACTCGAAAGATGATCCGGCGACCGATTGGGAATGGGCAAAAAACGAA
GATGGTAGCTACTTCACCATTGACGGCTACTGGTGGAGCTCCGTTTCATTTAAAAACATGTTCTACACCA
ACACGTCGCAAAACGTTATCCGTCAGCGTTGTGAAGCAACATTAGATTTGACGAACGAGAACGCAGACAT
TACGTTCTTCGCCGCTGACAATCGCTTCTCATACAACCACACGATCTGGAGCAACGACGCAGCAATGCAG
CCAGATCAAATCAACAAAGTGGTTGCACTCGGTGACAGCTTGTCTGATACAGGCAACATCTTTAACGCAT
CACAATGGCGCTTCCCTAACCCGAACAGCTGGTTCTTAGGTCACTTCTCCAACGGTTTTGTGTGGACAGA
ATACATTGCCAAAGCGAAGAACCTTCCGCTCTACAACTGGGCAGTTGGCGGCGCGGCTGGTGAGAACCAA
TACATCGCGCTAACAGGGGTTGGTGAGCAAGTTTCTTCGTACTTAACCTACGCAAAACTGGCGAAGAACT
ACAAACCAGCAAACACCTTGTTTACGCTTGAGTTTGGTTTGAATGACTTCATGAACTACAACCGTGGCGT
TCCAGAAGTGAAAGCAGATTATGCAGAAGCACTGATTCGTTTGACGGACGCAGGTGCGAAGAACTTCATG
TTGATGACACTGCCAGACGCGACGAAAGCGCCTCAGTTTAAGTACTCAACACAAGAAGAGATCGACAAAA
TTCGTGCGAAAGTGCTTGAGATGAACGAGTTCATCAAGGCACAAGCGATGTACTACAAAGCGCAAGGTTA
CAACATCACGTTGTTTGATACTCACGCCTTGTTCGAGACGCTAACTTCTGCGCCCGAAGAGCACGGTTTC
GTGAACGCGAGTGATCCTTGTTTGGACATCAACCGCTCATCGTCTGTCGATTACATGTACACCCACGCAT
TGCGCTCTGAGTGTGCTGCGTCTGGTGCTGAGAAGTTTGTGTTCTGGGATGTCACGCATCCAACAACAGC
AACTCACCGCTATGTTGCAGAGAAAATGCTAGAAAGTAGCAACAACTTAGCAGAGTACCGTTTCTAA

>CFSAN007451
ATGATGAAAAAAACAATCACACTATTAACTGCATTACTCCCGCTTGCTTCTGCAGTTGCCGAAGAGCCAA
CCTTATCACCAGAAATGGTTTCAGCGTCTGAAGTGATCAGCACGCAAGAAAACCAAACCTATACCTATGT
TCGCTGTTGGTATCGCACCAGCTACTCGAAAGATGATCCAGCGACCGATTGGGAATGGGCAAAAAACGAA
GATGGTAGCTACTTCACCATTGACGGCTACTGGTGGAGCTCCGTTTCATTTAAAAACATGTTCTACACCA
ACACGTCGCAAAACGTTATCCGTCAGCGTTGTGAAGCAACATTAGATTTGGCGAACGAGAACGCAGACAT
TACGTTCTTCGCCGCTGACAATCGCTTCTCATACAACCACACGATCTGGAGCAACGACGCAGCAATGCAG
CCAGATCAAATCAACAAAGTGGTTGCACTCGGTGACAGCTTGTCTGATACAGGCAACATCTTTAACGCAT
CACAATGGCGCTTCCCTAACCCGAACAGCTGGTTCTTAGGTCACTTCTCCAACGGTTTTGTTTGGACAGA

ATACATTGCCAAAGCGAAGAACCTTCCGCTCTACAACTGGGCAGTTGGCGGCGCGGCTGGTGAGAACCAA
TACATCGCGCTAACAGGGTTGGTGAGCAAGTTTCTTCGTACTTAACCTACGCAAAACTGGCGAAGAACTA
CAAACCAGCAAACACCTTGTTTACGCTTGAGTTTGGTTTGAATGACTTCATGAACTACAACCGTGGCGTT
CCAGAAGTGAAAGCGGATTATGCAGAAGCACTGATTCGTTTGACGGACGCAGGTGCGAAGAACTTCATGT
TGATGACACTGCCAGATGCGACGAAAGCGCCTCAGTTTAAGTACTCAACACAAGAAGAGATCGACAAAAT
TCGTGCGAAAGTGCTTGAGATGAACGAGTTCATCAAGGCACAAGCGATGTACTACAAAGCGCAAGGTTAC
AACATCACGTTGTTTGATACTCACGCCTTGTTCGAGACGCTAACTTCTGCGCCAGAAGAGCACGGTTTCG
TGAACGCGAGCGATCCTTGTTTGGACATCAACCGCTCATCGTCTGTCGATTACATGTACACCCACGCATT
GCGCTCTGAGTGTGCAGCGTCTGGTGCTGAGAAGTTTGTGTTCTGGGATGTCACGCATCCAACAACAGCA
ACTCACCGCTATGTTGCAGAGAAAATGCTAGAAAGTAGCAACAACTTAGCCGAGTACCGTTTCTAA

>CFSAN007453
ATGATGAAAAAAACAATCACACTATTAACTGCATTACTCCCGCTTGCTTCTGCAGTTGCCGAAGAGCCAACCTTAT
CACCAGAAATGGTTTCAGCGTCTGAAGTGATCAGCACGCAAGAAAACCAAACCTATACCTATGTTCGCTGTTGGTA
TCGCACCAGCTACTCGAAAGATGATCCGGCGACCGATTGGGAATGGGCAAAAAACGAAGATGGTAGCTACTTCAC
CATTGACGGCTACTGGTGGAGCTCCGTTTCATTTAAAAACATGTTCTACACCAACACGTCGCAAAACGTTATCCGT
CAGCGTTGTGAAGCAACATTAGATTTGACGAACGAGAACGCAGACATTACGTTCTTCGCCGCTGACAATCGCTTCT
CATACAACCACACGATCTGGAGCAACGACGCAGCAATGCAGCCAGATCAAATCAACAAAGTGGTTGCACTCGGTG
ACAGCTTGTCTGATACAGGCAACATCTTTAACGCATCACAATGGCGCTTCCCTAACCCGAACAGCTGGTTCTTAGG
TCACTTCTCCAACGGTTTTGTGTGGACAGAATACATTGCCAAAGCGAAGAACCTTCCGCTCTACAACTGGGCAGTT
GGCGGCGCGGCTGGTGAGAACCAATACATCGCGCTAACAGGGGTTGGTGAGCAAGTTTCTTCGTACTTAACCTACG
CAAAACTGGCGAAGAACTACAAACCAGCAAACACCTTGTTTACGCTTGAGTTTGGTTTGAATGACTTCATGAACTA
CAACCGTGGCGTTCCAGAAGTGAAAGCAGATTATGCAGAAGCACTGATTCGTTTGACGGACGCAGGTGCGAAGAA
CTTCATGTTGATGACACTGCCAGACGCGACGAAAGCGCCTCAGTTTAAGTACTCAACACAAGAAGAGATCGACAA
AATTCGTGCGAAAGTGCTTGAGATGAACGAGTTCATCAAGGCACAAGCGATGTACTACAAAGCGCAAGGTTACAA
CATCACGTTGTTTGATACTCACGCCTTGTTCGAGACGCTAACTTCTGCGCCCGAAGAGCACGGTTTCGTGAACGCG
AGTGATCCTTGTTTGGACATCAACCGCTCATCGTCTGTCGATTACATGTACACCCACGCATTGCGCTCTGAGTGTGC
TGCGTCTGGTGCTGAGAAGTTTGTGTTCTGGGATGTCACGCATCCAACAACAGCAACTCACCGCTATGTTGCAGAG
AAAATGCTAGAAAGTAGCAACAACTTAGCAGAGTACCGTTTCTAA

>CFSAN006132
ATGATGAAAAAAACAATCACACTATTAACTGCATTACTCCCGCTTGCTTCTGCAGTTGCCGAAGAGCCAACCTTAT
CACCAGAAATGGTTTCAGCGTCTGAAGTGATCAGCACGCAAGAAAACCAAACCTATACCTATGTTCGCTGTTGGTA
TCGCACCAGCTACTCGAAAGATGATCCGGCGACCGATTGGGAATGGGCAAAAAACGAAGATGGTAGCTACTTCAC
CATTGACGGCTACTGGTGGAGCTCCGTTTCACTTAAAAACATGTTCTACACCAACACGTCGCAAAACGTTATCCGT
CAGCGTTGTGAAGCAACATTAGATTTGGCGAACGAGAACGCAGACATTACGTTCTTCGCCGCTGACAATCGCTTCT
CATACAACCACACGATCTGGAGCAACGACGCAGCAATGCAGCCAGATCAAATCAACAAAGTGGTTGCACTCGGTG
ACAGCTTGTCTGATACAGGCAACATCTTTAACGCATCACAATGGCGCTTCCCTAACCCGAACAGCTGGTTCTTAGG
TCACTTCTCCAACGGTTTTGTGTGGACAGAATACATTGCCAAAGCGAAGAACCTTCCGCTCTACAACTGGGCAGTT
GGCGGCGCGGCTGGTGAGAACCAATACATCGCGCTAACAGGTTGGTGATCAAGTTTCTTCGTACTTAACCTACGCA
AAACTGGCGAAGAACTACAAACCAGCAAACACCTTGTTTACGCTTGAGTTTGGTTTGAATGACTTCATGAACTACA
ACCGTGGCGTTCCAGAAGTGAAAGCAGATTATGCAGAAGCACTGATTCGTTTGACGGACGCAGGTGCGAAGAACT
TCATGTTGATGACACTGCCAGACGCGACGAAAGCGCCTCAGTTTAAGTACTCAACACAAGAAGAGATCGACAAAT
TCGTGCGAAAGTGCTTGAGATGAACGAGTTCATCAAGGCACAAGCGATGTACTACAAAGCGCAAGGTTACAACAT
CACGTTGTTTGATACTCACGCCTTGTTCGAGACGCTAACTTCTGCGCCAGAAGAGCACGGTTTCGTGAACGCGAGC
GATCCTTGTTTGGACATCAACCGCTCATCGTCTGTCGATTACATGTACACCCACGCATTGCGCTCTGAGTGTGCAGC
GTCTGGTGCTGAGAAGTTTGTGTTCTGGGATGTCACGCATCCAACAACAGCAACTCACCGCTATGTTGCAGAGAAA
ATGCTAGAAAGTAGCAACAACTTAGCCGAGTACCGTTTCTAA

>CFSAN006133
ATGATGAAAAAAACAATCACACTATTAACTGCATTACTCCCGCTTGCTTCTGCAGTTGCCGAAGAGCCAA

CCTTATCACCAGAAATGGTTTCAGCGTCTGAAGTGATCAGCACGCAAGAAAACCAAACCTATACCTATGT
TCGCTGTTGGTATCGCACCAGCTACTCGAAAGATGATCCGGCGACCGATTGGGAATGGGCAAAAAACGAA
GATGGTAGCTACTTCACCATTGACGGCTACTGGTGGAGCTCCGTTTCACTTAAAAACATGTTCTACACCA
ACACGTCGCAAAACGTTATCCGTCAGCGTTGTGAAGCAACATTAGATTTGGCGAACGAGAACGCAGACAT
TACGTTCTTCGCCGCTGACAATCGCTTCTCATACAACCACACGATCTGGAGCAACGACGCAGCAATGCAG
CCAGATCAAATCAACAAAGTGGTTGCACTCGGTGACAGCTTGTCTGATACAGGCAACATCTTTAACGCAT
CACAATGGCGCTTCCCTAACCCGAACAGCTGGTTCTTAGGTCACTTCTCCAACGGTTTTGTGTGGACAGA
ATACATTGCCAAAGCGAAGAACCTTCCGCTCTACAACTGGGCAGTTGGCGGCGCGGCTGGTGAGAACCAA
TACATCGCGCTAACAGGGTTGGTGATCAAGTTTCTTCGTACTTAACCTACGCAAAACTGGCGAAGAACTA
CAAACCAGCAAACACCTTGTTTACGCTTGAGTTTGGTTTGAATGACTTCATGAACTACAACCGTGGCGTT
CCAGAAGTGAAAGCAGATTATGCAGAAGCACTGATTCGTTTGACGGACGCAGGTGCGAAGAACTTCATGT
TGATGACACTGCCAGACGCGACGAAAGCGCCTCAGTTTAAGTACTCAACACAAGAAGAGATCGACAAAAT
TCGTGCGAAAGTGCTTGAGATGAACGAGTTCATCAAGGCACAAGCGATGTACTACAAAGCGCAAGGTTAC
AACATCACGTTGTTTGATACTCACGCCTTGTTCGAGACGCTAACTTCTGCGCCAGAAGAGCACGGTTTCG
TGAACGCGAGCGATCCTTGTTTGGACATCAACCGCTCATCGTCTGTCGATTACATGTACACCCACGCATT
GCGCTCTGAGTGTGCAGCGTCTGGTGCTGAGAAGTTTGTGTTCTGGGATGTCACGCATCCAACAACAGCA
ACTCACCGCTATGTTGCAGAGAAAATGCTAGAAAGTAGCAACAACTTAGCCGAGTACCGTTTCTAA

>CFSAN001614
ATGATGAAAAAAACAATCACACTATTAACTGCATTACTCCCGCTTGCTTCTGCAGTTGCCGAAGAGCCAACCTTAT
CACCAGAAATGGTTTCAGCGTCTGAAGTGATCAGCACGCAAGAAAACCAAACCTATACCTATGTTCGCTGTTGGTA
TCGCACCAGCTACTCGAAAGATGATCCGGCGACCGATTGGGAATGGGCAAAAAACGAAGATGGTAGCTACTTCAC
CATTGACGGCTACTGGTGGAGCTCCGTTTCACTTAAAAACATGTTCTACACCAACACGTCGCAAAACGTTATCCGT
CAGCGTTGTGAAGCAACATTAGATTTGGCGAACGAGAACGCAGACATTACGTTCTTCGCCGCTGACAATCGCTTCT
CATACAACCACACGATCTGGAGCAACGACGCAGCAATGCAGCCAGATCAAATCAACAAAGTGGTTGCACTCGGTG
ACAGCTTGTCTGATACAGGCAACATCTTTAACGCATCACAATGGCGCTTCCCTAACCCGAACAGCTGGTTCTTAGG
TCACTTCTCCAACGGTTTTGTGTGGACAGAATACATTGCCAAAGCGAAGAACCTTCCGCTCTACAACTGGGCAGTT
GGCGGCGCGGCTGGTGAGAACCAATACATCGCGCTAACAGGGGTTGGTGATCAAGTTTCTTCGTACTTAACCTACG
CAACACTGGCGAAGAACTACAAACCAGCAAACACCTTGTTTACGCTTGAGTTTGGTTTGAATGACTTCATGAACTA
CAACCGTGGCGTTCCAGAAGTGAAAGCAGATTATGCAGAAGCACTGATTCGTTTGACGGACGCAGGTGCGAAGAA
CTTCATGTTGATGACACTGCCAGACGCGACGAAAGCGCCTCAGTTTAAGTACTCAACACAAGAAGAGATCGACAA
AATTCGTGCGAAAGTGCTTGAGATGAACGAGTTCATCAAGGCACAAGCGATGTACTACAAAGCGCAAGGTTACAA
CATCACGTTGTTTGATACTCACGCCTTGTTCGAGACGCTAACTTCTGCGCCAGAAGAGCACGGTTTCGTGAACGCG
AGCGATCCTTGTTTGGACATCAACCGCTCATCGTCTGTCGATTACATGTACACCCACGCATTGCGCTCTGAGTGTGC
AGCGTCTGGTGCTGAGAAGTTTGTGTTCTGGGATGTCACGCATCCAACAACAGCAACTCACCGCTATGTTGCAGAG
AAAATGCTAGAAAGTAGCAACAACTTAGCCGAGTACCGTTTCTAA

>CFSAN007454
ATGATGAAAAAAACAATCACACTATTAACTGCATTACTCCCGCTTGCTTCTGCAGTTGCCGAAGAGCCAA
CCTTATCACCAGAAATGGTTTCAGCGTCTGAAGTGATCAGCACGCAAGAAAACCAAACCTATACCTATGT
TCGCTGTTGGTATCGCACCAGCTACTCGAAAGATGATCCGGCGACCGATTGGGAATGGGCAAAAAACGAA
GATGGTAGCTACTTCACCATTGACGGCTACTGGTGGAGCTCCGTTTCATTTAAAAACATGTTCTACACCA
ACACGTCGCAAAACGTTATCCGTCAGCGTTGTGAAGCCACATTAGATTTGGCGAACGAGAACGCAGACAT
TACGTTCTTCGCCGCTGACAATCGCTTCTCATACAACCACACGATCTGGAGCAACGACGCAGCAATGCAG
CCAGATCAAATCAACAAAGTGGTTGCACTCGGTGACAGCTTGTCTGATACAGGCAACATCTTTAACGCAT
CACAATGGCGCTTCCCTAACCCGAACAGCTGGTTCTTAGGTCACTTCTCCAACGGTTTTGTGTGGACAGA
ATACATTGCCAAAGCGAAGAACCTTCCGCTCTACAACTGGGCCGTTGGCGGCGCGGCTGGTGAGAACCAA
TACATCGCGCTAACAGGGGTTGGTGATCAAGTTTCTTCGTACTTAACCTACGCAAAACTGGCGAAGAACT
ACAAACCAGCAAACACCTTGTTTACGCTTGAGTTTGGTTTGAATGACTTCATGAACTACAACCGTGGCGT
TCCAGAAGTGAAAGCAGATTATGCAGAAGCACTGATTCGTTTGACGGACGCAGGTGCGAAGAACTTCATG
TTGATGACATTGCCAGACGCGACGAAAGCGCCTCAGTTTAAGTACTCAACACAAGAAGAGATCGACAAAA

TTCGTGCGAAAGTGCTTGAGATGAACGAGTTCATCAAGGCACAAGCGATGTACTACAAAGCGCAAGGTTA
CAACATCACGTTGTTTGATACTCACGCCTTGTTCGAGACGCTAACTTCTGCGCCAGAAGAGCACGGTTTC
GTGAACGCGAGCGATCCTTGTTTGGACATCAACCGCTCATCGTCTGTCGATTACATGTACACCCACGCAT
TGCGCTCTGAGTGTGCAGCGTCTGGTGCTGAGAAGTTTGTGTTCTGGGATGTCACGCACCCAACAACAGC
AACTCACCGCTATGTTGCAGAGAAAATGCTAGAAAGTAGCAACAACTTAGCCGAGTACCGTTTCTAA

>13-028/A2
ATGATGAAAAAAACAATCACACTATTAACTGCATTACTCCCGCTTGCTTCTGCAGTTGCCGAAGAGCCAA
CCTTATCACCAGAAATGGTTTCAGCGTCTGAAGTGATCAGCACGCAAGAAAACCAAACCTATACCTATGT
TCGCTGTTGGTATCGCACCAGCTACTCGAAAGATGATCCGGCGACCGATTGGGAATGGGCAAAAAACGAA
GATGGTAGCTACTTCACCATTGACGGCTACTGGTGGAGCTCCGTTTCATTTAAAAACATGTTCTACACCA
ACACGTCGCAAAACGTTATCCGTCAGCGTTGTGAAGCAACATTAGATTTGGCGAACGAGAACGCAGACAT
TACGTTCTTCGCCGCTGACAATCGCTTCTCATACAACCACACGATCTGGAGCAACGATGCAGCAATGCAG
CCAGATCAAATCAACAAAGTGGTTGCACTCGGTGACAGCTTGTCTGATACAGGCAACATCTTTAACGCAT
CACAATGGCGCTTCCCTAACCCGAACAGCTGGTTCTTAGGTCACTTCTCCAACGGTTTTGTGTGGACAGA
ATACATTGCCAAAGCGAAGAACCTTCCGCTCTACAACTGGGCAGTTGGCGGCGCGGCTGGTGAGAACCAA
TACATCGCGCTAACAGGGGTTGGTGATCAAGTTTCTTCGTACTTAACCTACGCAAAACTGGCGAAGAACT
ACAAACCAGCAAACACCTTGTTTACGCTTGAGTTTGGTTTGAATGACTTCATGAACTACAACCGTGGCGT
TCCAGAAGTGAAAGCGGATTATGCAGAAGCACTGATTCGTTTGACGGACGCAGGTGCGAAGAACTTCATG
TTGATGACACTGCCAGACGCGACGAAAGCGCCTCAGTTTAAGTACTCAACACAAGAAGAGATCGACAAAA
TTCGTGCGAAAGTGCTTGAGATGAACGAGTTCATCAAGGCACAAGCGATGTACTACAAAGCGCAAGGTTA
CAACATCACGTTGTTTGATACTCACGCCTTGTTCGAGACGCTAACTTCTGCGCCCGAAGAGCACGGTTTC
GTGAACGCGAGTGATCCTTGTTTGGACATCAACCGCTCATCGTCTGTCGATTACATGTACACCCACGCAT
TGCGCTCTGAGTGTGCGGCGTCTGGTGCTGAGAAGTTTGTGTTCTGGGATGTCACGCATCCAACAACAGC
AACTCACCGCTATGTTGCAGAGAAAATGCTAGAAAGTAGCAACAACTTAGCAGAGTACCGTTTCTAA

>FIM-S1708+
ATGATGAAAAAAACAATCACACTATTAACTGCATTACTCCCGCTTGCTTCTGCAGTTGCCGAAGAGCCAA
CCTTATCACCAGAAATGGTTTCAGCGTCTGAAGTGATCAGCACGCAAGAAAACCAAACCTATACCTATGT
TCGCTGTTGGTATCGCACCAGCTACTCGAAAGATGATCCGGCGACCGATTGGGAATGGGCAAAAAACGAA
GATGGTAGCTACTTCACCATTGACGGCTACTGGTGGAGCTCCGTTTCATTTAAAAACATGTTCTACACCA
ACACGTCGCAAAACGTTATCCGTCAGCGTTGTGAAGCAACATTAGATTTGGCGAACGAGAACGCAGACAT
TACGTTCTTCGCCGCTGACAATCGCTTCTCATACAACCACACGATCTGGAGCAACGACGCAGCAATGCAG
CCAGATCAAATCAACAAAGTGGTTGCACTCGGTGACAGCTTGTCTGATACAGGCAACATCTTTAACGCAT
CACAATGGCGCTTCCCTAACCCGAACAGCTGGTTCTTAGGTCACTTCTCCAACGGTTTTGTGTGGACAGA
ATACATTGCCAAAGCGAAGAACCTTCCGCTCTACAACTGGGCAGTTGGCGGCGCGGCTGGTGAGAACCAA
TACATCGCGCTAACAGGGGTTGGTGATCAAGTTTCTTCGTACTTAACCTACGCAAAACTGGCGAAGAACT
ACAAACCAGCAAACACCTTGTTTACGCTTGAGTTTGGTTTGAATGACTTCATGAACTACAACCGTGGCGT
TCCAGAAGTGAAAGCGGATTATGCAGAAGCACTGATTCGTTTGACGGACGCAGGTGCGAAGAACTTCATG
TTGATGACACTGCCAGACGCGACGAAAGCGCCTCAGTTTAAGTACTCAACACAAGAAGAGATCGACAAAA
TTCGTGCGAAAGTGCTTGAGATGAACGAGTTCATCAAGGCACAAGCGATGTACTACAAAGCGCAAGGTTA
CAACATCACGTTGTTTGATACTCACGCCTTGTTCGAGACGCTAACTTCTGCGCCAGAAGAGCACGGTTTC
GTGAACGCGAGTGATCCTTGTTTGGACATCAACCGCTCATCGTCTGTCGATTACATGTACACCCACGCAT
TGCGCTCTGAGTGTGCGGCGTCTGGTGCTGAGAAATTTGTGTTCTGGGATGTCACGCACCCAACAACAGC
AACTCACCGCTATGTTGCAGAGAAAATGCTAGAAAGTAGCAACAACTTAGCCGAGTACCGTTTCTAA

>FIM-S1392-
ATGATGAAAAAAACAATCACACTATTAACTGCATTACTCCCGCTTGCTTCTGCAGTTGCCGAAGAGCCAA
CCTTATCACCAGAAATGGTTTCAGCGTCTGAAGTGATCAGCACGCAAGAAAACCAAACCTATACCTATGT
TCGCTGTTGGTATCGCACCAGCTACTCGAAAGATGATCCGGCGACCGATTGGGAATGGGCAAAAAACGAA
GATGGTAGCTACTTCACCATTGACGGCTACTGGTGGAGCTCCGTTTCATTTAAAAACATGTTCTACACCA

ACACGTCGCAAAACGTTATCCGTCAGCGTTGTGAAGCCACATTAGATTTGGCGAACGAGAACGCAGACAT
TACGTTCTTCGCCGCTGACAATCGCTTCTCATACAACCACACGATCTGGAGCAACGACGCAGCAATGCAG
CCAGATCAAATCAACAAAGTGGTTGCACTCGGTGACAGCTTGTCTGATACAGGCAACATCTTTAACGCAT
CACAATGGCGCTTCCCTAACCCGAACAGCTGGTTCTTAGGTCACTTCTCCAACGGTTTTGTGTGGACAGA
ATACATTGCCAAAGCGAAGAACCTTCCGCTCTACAACTGGGCAGTTGGCGGCGCGGCTGGTGAGAACCAA
TACATCGCGCTAACAGGGGTTGGTGATCAAGTTTCTTCGTACTTAACCTACGCAAAACTGGCGAAGAACT
ACAAACCAGCAAACACCTTGTTTACGCTTGAGTTTGGTTTGAATGACTTCATGAACTACAACCGTGGCGT
TCCAGAAGTGAAAGCAGATTATGCAGAAGCACTGATTCGTTTGACGGACGCAGGTGCGAAGAACTTCATG
TTGATGACACTGCCAGACGCGACGAAAGCGCCTCAGTTTAAGTACTCAACACAAGAAGAGATCGACAAAA
TTCGTGCGAAAGTGCTTGAGATGAACGAGTTCATCAAGGCACAAGCGATGTACTACAAAGCGCAAGGTTA
CAACATCACGTTGTTTGATACTCACGCCTTGTTCGAGACGCTAACTTCTGCGCCAGAAGAGCACGGTTTC
GTGAACGCGAGCGATCCTTGTTTGGACATCAACCGCTCATCGTCTGTCGACTACATGTACACCCACGCAT
TGCGCTCTGAGTGTGCGGCGTCTGGTGCTGAGAAGTTTGTATTCTGGGATGTCACGCACCCAACAACAGC
AACTCACCGCTATGTTGCAGAGAAAATGCTAGAAAGTAGCAACAACTTAGCCGAGTACCGTTTCTAA

>NCKU_TV_5HP
ATGATGAAAAAAACAATCACACTATTAACTGCATTACTCCCGCTTGCTTCTGCAGTTGCCGAAGAGCCAA
CCTTATCACCAGAAATGGTTTCAGCGTCTGAAGTGATCAGCACGCAAGAAAACCAAACCTATACCTATGT
TCGCTGTTGGTATCGCACCAGCTACTCGAAAGATGATCCGGCGACCGATTGGGAATGGGCAAAAAACGAA
GATGGTAGCTACTTCACCATTGACGGTTACTGGTGGAGCTCCGTTTCATTTAAAAACATGTTCTACACCA
ACACGTCGCAAAACGTTATCCGTCAGCGTTGTGAAGCCACATTAGATTTGGCGAACGAGAACGCAGACAT
TACGTTCTTCGCCGCTGACAATCGCTTCTCATACAACCACACGATCTGGAGCAACGACGCAGCAATGCAG
CCAGATCAAATCAACAAAGTGGTTGCACTCGGTGACAGCTTGTCTGATACAGGCAACATCTTTAACGCAT
CACAATGGCGCTTCCCTAACCCGAACAGCTGGTTCTTAGGTCACTTCTCCAACGGTTTTGTGTGGACAGA
ATACATTGCCAAAGCGAAGAACCTTCCGCTCTACAACTGGGCAGTTGGCGGCGCGGCTGGTGAGAACCAA
TACATCGCGCTAACAGGGGTTGGTGAGCAAGTTTCTTCGTACTTAACCTACGCAAAACTGGCGAAGAACT
ACAAACCAGCAAACACCTTGTTTACGCTTGAGTTTGGTTTGAATGACTTCATGAACTACAACCGTGGCGT
TCCAGAAGTGAAAGCAGATTATGCAGAAGCACTGATTCGTTTGACGGACGCAGGTGCGAAGAACTTCATG
TTGATGACACTGCCAGACGCGACGAAAGCGCCTCAGTTTAAGTACTCAACACAAGAAGAGATCGACAAAA
TTCGTGCGAAAGTGCTTGAGATGAACGAGTTCATCAAGGCACAAGCGATGTACTACAAAGCGCAAGGTTA
CAACATCACGTTGTTTGATACTCACGCCTTGTTCGAGACGCTAACTTCTGCGCCCGAAGAGCACGGTTTC
GTGAACGCGAGTGATCCTTGTTTGGACATCAACCGCTCATCGTCTGTCGATTACATGTACACCCACGCAT
TGCGCTCTGAGTGTGCGGCGTCTGGTGCTGAGAAGTTTGTGTTCTGGGATGTCACGCACCCAACAACAGC
AACTCACCGCTATGTTGCAGAGAAAATGCTAGAAAGTAGCAACAACTTAGCCGAGTACCGTTTCTAA

>NCKU_CV_CHN
ATGATGAAAAAAACAATCACACTATTAACTGCATTACTCCCGCTTGCTTCTGCAGTTGCCGAAGAGCCAACCTTAT
CACCAGAAATGGTTTCAGCGTCTGAAGTGATCAGCACGCAAGAAAACCAAACCTATACCTATGTTCGCTGTTGGTA
TCGCACCAGCTACTCGAAAGATGATCCGGCGACCGATTGGGAATGGGCAAAAAACGAAGATGGTAGCTACTTCAC
CATTGACGGCTACTGGTGGAGCTCCGTTTCATTTAAAAACATGTTCTACACCAACACGTCGCAAAACGTTATCCGT
CAGCGTTGTGAAGCAACATTAGATTTGGCGAACGAGAACGCAGACATTACGTTCTTCGCCGCTGACAATCGCTTCT
CATACAACCACACGATCTGGAGCAACGACGCAGCAATGCAGCCAGATCAAATCAACAAAGTGGTTGCACTCGGTG
ACAGCTTGTCTGATACAGGCAACATCTTTAACGCATCACAATGGCGCTTCCCTAACCCGAACAGCTGGTTCTTAGG
TCACTTCTCCAACGGTTTTGTGTGGACAGAATACATTGCCAAAGCGAAGAACCTTCCGCTCTACAACTGGGCAGTT
GGCGGCGCGGCTGGTGAGAACCAATACATCGCGCTAACAGGGGTTGGTGATCAAGTTTCTTCGTACTTAACCTACG
CAAAACTGGCGAAGAACTACAAACCAGCAAACACCTTGTTTACGCTTGAGTTTGGTTTGAATGACTTCATGAACTA
CAACCGTGGCGTTCCAGAAGTGAAAGCGGATTATGCAGAAGCACTGATTCGTTTGACGGACGCAGGTGCGAAGAA
CTTCATGTTGATGACACTGCCAGACGCGACGAAAGCGCCTCAGTTTAAGTACTCAACACAAGAAGAGATCGACAA
AATTCGTGCGAAAGTGCTTGAGATGAACGAGTTCATCAAGGCACAAGCGATGTACTACAAAGCGCAAGGTTACAA
CATCACGTTGTTTGATACTCACGCCTTGTTCGAGACGCTAACTTCTGCGCCAGAAGAGCACGGTTTCGTGAACGCG
AGTGATCCTTGTTTGGACATCAACCGCTCATCGTCTGTCGATTACATGTACACCCACGCATTGCGCTCTGAGTGTGC

GGCGTCTGGTGCTGAGAAATTTGTGTTCTGGGATGTCACGCACCCAACAACAGCAACTCACCGCTATGTTGCAGAG
AAAATGCTAGAAAGTAGCAACAACTTAGCCGAGTACCGTTTCTAA

>NCKU_TV_3HP
ATGATGAAAAAAACAATCACACTATTAACTGCATTACTCCCGCTTGCTTCTGCAGTTGCCGAAGAGCCAACCTTAT
CACCAGAAATGGTTTCAGCGTCTGAAGTGATCAGCACGCAAGAAAACCAAACCTATACCTATGTTCGCTGTTGGTA
TCGCACCAGCTACTCGAAAGATGATCCGGCGACCGATTGGGAATGGGCAAAAAACGAAGATGGTAGCTACTTCAC
CATTGACGGTTACTGGTGGAGCTCCGTTTCATTTAAAAACATGTTCTACACCAACACGTCGCAAAACGTTATCCGT
CAGCGTTGTGAAGCCACATTAGATTTGGCGAACGAGAACGCAGACATTACGTTCTTCGCCGCTGACAATCGCTTCT
CATACAACCACACGATCTGGAGCAACGACGCAGCAATGCAGCCAGATCAAATCAACAAAGTGGTTGCACTCGGTG
ACAGCTTGTCTGATACAGGCAACATCTTTAACGCATCACAATGGCGCTTCCCTAACCCGAACAGCTGGTTCTTAGG
TCACTTCTCCAACGGTTTTGTGTGGACAGAATACATTGCCAAAGCGAAGAACCTTCCGCTCTACAACTGGGCAGTT
GGCGGCGCGGCTGGTGAGAACCAATACATCGCGCTAACAGGGGTTGGTGAGCAAGTTTCTTCGTACTTAACCTACG
CAAAACTGGCGAAGAACTACAAACCAGCAAACACCTTGTTTACGCTTGAGTTTGGTTTGAATGACTTCATGAACTA
CAACCGTGGCGTTCCAGAAGTGAAAGCAGATTATGCAGAAGCACTGATTCGTTTGACGGACGCAGGTGCGAAGAA
CTTCATGTTGATGACACTGCCAGACGCGACGAAAGCGCCTCAGTTTAAGTACTCAACACAAGAAGAGATCGACAA
AATTCGTGCGAAAGTGCTTGAGATGAACGAGTTCATCAAGGCACAAGCGATGTACTACAAAGCGCAAGGTTACAA
CATCACGTTGTTTGATACTCACGCCTTGTTCGAGACGCTAACTTCTGCGCCCGAAGAGCACGGTTTCGTGAACGCG
AGTGATCCTTGTTTGGACATCAACCGCTCATCGTCTGTCGATTACATGTACACCCACGCATTGCGCTCTGAGTGTGC
GGCGTCTGGTGCTGAGAAGTTTGTGTTCTGGGATGTCACGCACCCAACAACAGCAACTCACCGCTATGTTGCAGAG
AAAATGCTAGAAAGTAGCAACAACTTAGCCGAGTACCGTTTCTAA

>NCKU_TN_S02
ATGATGAAAAAAACAATCACACTATTAACTGCATTACTCCCGCTTGCTTCTGCAGTTGCCGAAGAGCCAA
CCTTATCACCAGAAATGGTTTCAGCGTCTGAAGTGATCAGCACGCAAGAAAACCAAACCTATACCTATGT
TCGCTGTTGGTATCGCACCAGCTACTCGAAAGATGATCCGGCGACCGATTGGGAATGGGCAAAAAACGAA
GATGGTAGCTACTTCACCATTGACGGCTACTGGTGGAGCTCCGTTTCATTTAAAAACATGTTCTACACCA
ACACGTCGCAAAACGTTATCCGTCAGCGTTGTGAAGCAACATTAGATTTGGCGAACGAGAACGCAGACAT
TACGTTCTTCGCCGCTGACAATCGCTTCTCATACAACCACACGATCTGGAGCAACGACGCAGCAATGCAG
CCAGATCAAATCAACAAAGTGGTTGCACTCGGTGACAGCTTGTCTGATACAGGCAACATCTTTAACGCAT
CACAATGGCGCTTCCCTAACCCGAACAGCTGGTTCTTAGGTCACTTCTCCAACGGTTTTGTGTGGACAGA
ATACATTGCCAAAGCGAAGAACCTTCCGCTCTACAACTGGGCAGTTGGCGGCGCGGCTGGTGAGAACCAA
TATATCGCGCTAACAGGGGTTGGTGATCAAGTTTCTTCGTACTTAACCTACGCAAAACTGGCGAAGAACT
ACAAACCAGCAAACACCTTGTTTACGCTTGAGTTTGGTTTGAATGACTTCATGAACTACAACCGTGGCGT
TCCAGAAGTGAAAGCAGATTATGCAGAAGCACTGATTCGTTTGACGGACGCAGGTGCGAAGAACTTCATG
TTGATGACACTGCCAGACGCGACGAAAGCGCCTCAGTTTAAGTACTCAACACAAGAAGAGATCGACAAAA
TTCGTGCGAAAGTGCTTGAGATGAACGAGTTCATCAAGGCACAAGCGATGTACTACAAAGCGCAAGGTTA
CAACATCACGTTGTTTGATACTCACGCCTTGTTCGAGACGCTAACTTCTGCGCCCGAAGAGCACGGTTTC
GTGAACGCGAGTGATCCTTGTTTGGACATCAACCGCTCATCGTCTATCGATTACATGTACACCCACGCAT
TGCGCTCTGAGTGTGCGGCGTCTGGTGCTGAGAAGTTTGTGTTCTGGGATGTCACGCACCCAACAACAGC
AACTCACCGCTATGTTGCAGAGAAAATGCTAGAAAGTAGCAACAACTTAGCCGAGTACCGTTTCTAA

>13-028/A3
ATGATGAAAAAAACAATCACACTATTAACTGCATTACTCCCGCTTGCTTCTGCAGTTGCCGAAGAGCCAA
CCTTATCACCAGAAATGGTTTCAGCGTCTGAAGTGATCAGCACGCAAGAAAACCAAACCTATACCTATGT
TCGCTGTTGGTATCGCACCAGCTACTCGAAAGATGATCCGGCGACCGATTGGGAATGGGCAAAAAACGAA
GATGGTAGCTACTTCACCATTGACGGCTACTGGTGGAGCTCCGTTTCATTTAAAAACATGTTCTACACCA
ACACGTCGCAAAATGTTATCCGTCAGCGTTGTGAAGCAACATTAGATTTGGCGAACGAGAACGCAGACAT
TACGTTCTTCGCCGCTGACAATCGCTTCTCATACAACCACACGATCTGGAGCAACGACGCAGCAATGCAG
CCAGATCAAATCAACAAAGTGGTTGCACTCGGTGACAGCTTGTCTGATACAGGCAACATCTTTAACGCAT
CACAATGGCGCTTCCCTAACCCGAACAGCTGGTTCTTAGGTCACTTCTCCAACGGTTTTGTTTGGACAGA

ATACATTGCCAAAGCGAAGAACCTTCCGCTCTACAACTGGGCAGTTGGCGGCGCGGCTGGTGAGAACCAA
TACATCGCGCTAACAGGGGTTGGTGAGCAAGTTTCTTCGTACTTAACCTACGCAAAACTGGCGAAGAACT
ACAAACCAGCAAACACCTTGTTTACGCTTGAGTTTGGTTTGAATGACTTCATGAACTACAACCGTGGCGT
TCCAGAAGTGAAAGCAGATTATGCAGAAGCACTGATTCGTTTGACGGACGCAGGTGCGAAGAACTTCATG
TTGATGACACTGCCAGACGCGACGAAAGCGCCTCAGTTTAAGTACTCAACACAAGAAGAGATCGACAAAA
TTCGTGCGAAAGTGCTTGAGATGAACGAGTTCATCAAGGCACAAGCGATGTACTACAAAGCGCAAGGTTA
CAACATCACGTTGTTTGATACTCACGCCTTGTTCGAGACGCTAACTTCTGCGCCCGAAGAGCACGGTTTC
GTGAACGCGAGTGATCCTTGTTTGGACATCAACCGCTCATCGTCTGTCGATTACATGTACACCCACGCAT
TGCGCTCTGAGTGTGCGGCGTCTGGTGCTGAGAAGTTTGTGTTCTGGGATGTCACGCACCCAACAACAGC
AACTCACCGCTATGTTGCAGAGAAAATGCTAGAAAGTAGCAACAACTTAGCCGAGTACCGTTTCTAA

>VPA-67
ATGATGAAAAAAACAATCACACTATTAACTGCATTACTCCCGCTTGCTTCTGCAGTTGCCGAAGAGCCAACCTTAT
CACCAGAAATGGTTTCAGCGTCTGAAGTGATCAGCACGCAAGAAAACCAAACCTATACCTATGTTCGCTGTTGGTA
TCGCACCAGCTACTCGAAAGATGATCCGGCGACCGATTGGGAATGGGCAAAAAACGAAGATGGTAGCTACTTCAC
CATTGACGGCTACTGGTGGAGCTCCGTTTCATTTAAAAACATGTTCTACACCAACACGTCGCAAAACGTTATCCGT
CAGCGTTGTGAAGCAACATTAGATTTGGCGAACGAGAACGCAGACATTACGTTCTTCGCCGCTGACAATCGCTTCT
CATACAACCACACGATCTGGAGCAACGACGCAGCAATGCAGCCAGATCAAATCAACAAAGTGGTTGCACTCGGTG
ACAGCTTGTCTGATACCGGCAACATCTTTAACGCATCACAATGGCGCTTCCCTAACCCGAACAGCTGGTTCTTAGG
TCACTTCTCCAACGGTTTTGTGTGGACAGAATACATTGCCAAAGCGAAGAACCTTCCGCTCTACAACTGGGCAGTT
GGCGGCGCGGCTGGTGAGAACCAATACATCGCGCTAACAGGTTGGTGATCAAGTTTCTTCGTACTTAACCTACGCA
AAACTGGCGAAGAACTACAAACCAGCAAACACCTTGTTTACGCTTGAGTTTGGTTTGAATGACTTCATGAACTACA
ACCGTGGCGTTCCAGAAGTGAAAGCGGATTATGCAGAAGCACTGATTCGTTTGACGGACGCAGGTGCGAAGAACT
TCATGTTGATGACACTGCCAGACGCGACGAAAGCGCCTCAGTTTAAGTACTCAACACAAGAAGAGATCGACAAAA
TTCGTGCGAAAGTGCTTGAGATGAACGAGTTCATCAAGGCACAAGCGATGTACTACAAAGCGCAAGGTTACAACA
TCACGTTGTTTGATACTCACGCCTTGTTCGAGACGCTAACTTCTGCGCCAGAAGAGCACGGTTTCGTGAACGCGAG
CGATCCTTGTTTGGACATCAACCGCTCATCGTCTGTCGATTACATGTACACCCACGCATTGCGCTCTGAGTGTGCAG
CGTCTGGTGCTGAGAAGTTTGTGTTCTGGGATGTCACGCATCCAACAACAGCAACTCACCGCTATGTTGCAGAGAA
AATGCTAGAAAGTAGCAACAACTTAGCCGAGTACCGTTTCTAA

>NBRC 12711
ATGATGAAAAAAACAATCACACTATTAACTGCATTACTCCCGCTTGCTTCTGCAGTTGCCGAAGAGCCAA
CCTTATCACCAGAAATGGTTTCAGCGTCTGAAGTGATCAGCACGCAAGAAAACCAAACCTATACCTATGT
TCGCTGTTGGTATCGCACCAGCTACTCGAAAGATGATCCGGCGACCGATTGGGAATGGGCAAAAAACGAA
GATGGTAGCTACTTCACCATTGACGGCTACTGGTGGAGCTCCGTTTCATTTAAAAACATGTTCTACACCA
ACACGTCGCAAAACGTTATCCGTCAGCGTTGTGAAGCAACATTAGATTTGGCGAACGAGAACGCAGACAT
TACGTTCTTCGCCGCTGACAATCGCTTCTCATACAACCACACGATCTGGAGCAACGACGCAGCAATGCAG
CCAGATCAAATCAACAAAGTGGTTGCACTCGGTGACAGCTTGTCTGATACAGGCAACATATTTAACGCAT
CACAATGGCGCTTCCCTAACCCGAACAGCTGGTTCTTAGGTCACTTCTCCAACGGTTTTGTGTGGACAGA
ATACATTGCCAAAGCGAAGAACCTTCCGCTCTACAACTGGGCAGTTGGCGGCGCGGCTGGTGAGAACCAA
TACATCGCGCTAACAGGGGTTGGTGATCAAGTTTCTTCGTACTTAACCTACGCAAAACTGGCGAAGAACT
ACAAACCAGCAAACACCTTGTTTACGCTTGAGTTTGGTTTGAATGACTTCATGAACTACAACCGTGGCGT
TCCAGAAGTGAAAGCGGATTATGCAGAAGCACTGATTCGTTTGACGGACGCAGGTGCGAAGAACTTCATG
TTGATGACACTGCCAGATGCGACGAAAGCGCCTCAGTTTAAGTACTCAACACAAGAAGAGATCGACAAAA
TTCGTGCGAAAGTGCTTGAGATGAACGAGTTCATCAAGGCACAAGCGATGTACTACAAAGCGCAAGGTTA
CAACATCACGTTGTTTGATACTCACGCCTTGTTCGAGACGCTAACTTCTGCGCCAGAAGAGCACGGTTTC
GTGAACGCGAGTGATCCTTGTTTGGACATCAACCGCTCATCGTCTGTCGATTACATGTACACCCACGCAT
TGCGCTCTGAGTGTGCAGCATCTGGTGCTGAGAAGTTTGTGTTCTGGGATGTCACGCATCCAACAACAGC
AACTCACCGCTATGTTGCAGAGAAAATGCTAGAAAGTAGCAACAACTTAGCAGAGTACCGTTTCTAA

>846

ATGATGAAAAAAACAATCACACTATTAACTGCATTACTCCCGCTTGCTTCTGCAGTTGCCGAAGAGCCAACCTTAT
CACCAGAAATGGTTTCAGCGTCTGAAGTGATCAGCACGCAAGAAAACCAAACCTATACCTATGTTCGCTGTTGGTA
TCGCACCAGCTACTCGAAAGATGATCCGGCGACCGATTGGGAATGGGCAAAAAACGAAGATGGTAGCTACTTCAC
CATTGACGGCTACTGGTGGAGCTCCGTTTCACTTAAAAACATGTTCTACACCAACACGTCGCAAAACGTTATCCGT
CAGCGTTGTGAAGCAACATTAGATTTGGCGAACGAGAACGCAGACATTACGTTCTTCGCCGCTGACAATCGCTTCT
CATACAACCACACGATCTGGAGCAACGACGCAGCAATGCAGCCAGATCAAATCAACAAAGTGGTTGCACTCGGTG
ACAGCTTGTCTGATACAGGCAACATCTTTAACGCATCACAATGGCGCTTCCCTAACCCGAACAGCTGGTTCTTAGG
TCACTTCTCCAACGGTTTTGTGTGGACAGAATACATTGCCAAAGCGAAGAACCTTCCGCTCTACAACTGGGCAGTT
GGCGGCGCGGCTGGTGAGAACCAATACATCGCGCTAACAGGGGTTGGTGATCAAGTTTCTTCGTACTTAACCTACG
CAAAACTGGCGAAGAACTACAAACCAGCAAACACCTTGTTTACGCTTGAGTTTGGTTTGAATGACTTCATGAACTA
CAACCGTGGCGTTCCAGAAGTGAAAGCAGATTATGCAGAAGCACTGATTCGTTTGACGGACGCAGGTGCGAAGAA
CTTCATGTTGATGACACTGCCAGACGCGACGAAAGCGCCTCAGTTTAAGTACTCAACACAAGAAGAGATCGACAA
AATTCGTGCGAAAGTGCTTGAGATGAACGAGTTCATCAAGGCACAAGCGATGTACTACAAAGCGCAAGGTTACAA
CATCACGTTGTTTGATACTCACGCCTTGTTCGAGACGCTAACTTCTGCGCCAGAAGAGCACGGTTTCGTGAACGCG
AGCGATCCTTGTTTGGACATCAACCGCTCATCGTCTGTCGATTACATGTACACCCACGCATTGCGCTCTGAGTGTGC
AGCGTCTGGTGCTGAGAAGTTTGTGTTCTGGGATGTCACGCATCCAACAACAGCAACTCACCGCTATGTTGCAGAG
AAAATGCTAGAAAGTAGCAACAACTTAGCCGAGTACCGTTTCTAA

>VP551
ATGATGAAAAAAACAATCACACTATTAACTGCATTACTCCCGCTTGCTTCTGCAGTTGCCGAAGAGCCAACCTTAT
CACCAGAAATGGTTTCAGCGTCTGAAGTGATCAGCACGCAAGAAAACCAAACCTATACCTATGTTCGCTGTTGGTA
TCGCACCAGCTACTCGAAAGATGATCCAGCGACCGATTGGGAATGGGCAAAAAACGAAGATGGTAGCTACTTCAC
CATTGACGGCTACTGGTGGAGCTCCGTTTCATTTAAAAACATGTTCTACACCAACACGTCGCAAAACGTTATCCGT
CAGCGTTGTGAAGCAACATTAGATTTGGCGAACGAGAACGCAGACATTACGTTCTTCGCCGCTGACAATCGCTTCT
CATACAACCACACGATCTGGAGCAACGACGCAGCAATGCAGCCAGATCAAATCAACAAAGTGGTTGCACTCGGTG
ACAGCTTGTCTGATACAGGCAACATCTTTAACGCATCACAATGGCGCTTCCCTAACCCGAACAGCTGGTTCTTAGG
TCACTTCTCCAACGGTTTTGTTTGGACAGAATACATTGCCAAAGCGAAGAACCTTCCGCTCTACAACTGGGCAGTT
GGCGGCGCGGCTGGTGAGAACCAATACATCGCGCTAACAGGGGTTGGTGAGCAAGTTTCTTCGTACTTAACCTACG
CAAAACTGGCGAAGAACTACAAACCAGCAAACACCTTGTTTACGCTTGAGTTTGGTTTGAATGACTTCATGAACTA
CAACCGTGGCGTTCCAGAAGTGAAAGCGGATTATGCAGAAGCACTGATTCGTTTGACGGACGCAGGTGCGAAGAA
CTTCATGTTGATGACACTGCCAGATGCGACGAAAGCGCCTCAGTTTAAGTACTCAACACAAGAAGAGATCGACAA
AATTCGTGCGAAAGTGCTTGAGATGAACGAGTTCATCAAGGCACAAGCGATGTACTACAAAGCGCAAGGTTACAA
CATCACGTTGTTTGATACTCACGCCTTGTTCGAGACGCTAACTTCTGCGCCAGAAGAGCACGGTTTCGTGAACGCG
AGCGATCCTTGTTTGGACATCAACCGCTCATCGTCTGTCGATTACATGTACACCCACGCATTGCGCTCTGAGTGTGC
AGCGTCTGGTGCTGAGAAGTTTGTGTTCTGGGATGTCACGCATCCAACAACAGCAACTCACCGCTATGTTGCAGAG
AAAATGCTAGAAAGTAGCAACAACTTAGCCGAGTACCGTTTCTAA

>97-10290
ATGATGAAAAAAACAATCACACTATTAACTGCATTACTCCCGCTTGCTTCTGCAGTTGCCGAAGAGCCAACCTTAT
CACCAGAAATGGTTTCAGCGTCTGAAGTGATCAGCACGCAAGAAAACCAAACCTATACCTATGTTCGCTGTTGGTA
TCGCACCAGCTACTCGAAAGATGATCCGGCGACCGATTGGGAATGGGCAAAAAACGAAGATGGTAGCTACTTCAC
CATTGACGGCTACTGGTGGAGCTCCGTTTCACTTAAAAACATGTTCTACACCAACACGTCGCAAAACGTTATCCGT
CAGCGTTGTGAAGCAACATTAGATTTGGCGAACGAGAACGCAGACATTACGTTCTTCGCCGCTGACAATCGCTTCT
CATACAACCACACGATCTGGAGCAACGACGCAGCAATGCAGCCAGATCAAATCAACAAAGTGGTTGCACTCGGTG
ACAGCTTGTCTGATACAGGCAACATCTTTAACGCATCACAATGGCGCTTCCCTAACCCGAACAGCTGGTTCTTAGG
TCACTTCTCCAACGGTTTTGTGTGGACAGAATACATTGCCAAAGCGAAGAACCTTCCGCTCTACAACTGGGCAGTT
GGCGGCGCGGCTGGTGAGAACCAATACATCGCGCTAACAGGGGTTGGTGATCAAGTTTCTTCGTACTTAACCTACG
CAAAACTGGCGAAGAACTACAAACCAGCAAACACCTTGTTTACGCTTGAGTTTGGTTTGAATGACTTCATGAACTA
CAACCGTGGCGTTCCAGAAGTGAAAGCAGATTATGCAGAAGCACTGATTCGTTTGACGGACGCAGGTGCGAAGAA
CTTCATGTTGATGACACTGCCAGACGCGACGAAAGCGCCTCAGTTTAAGTACTCAACACAAGAAGAGATCGACAA
AATTCGTGCGAAAGTGCTTGAGATGAACGAGTTCATCAAGGCACAAGCGATGTACTACAAAGCGCAAGGTTACAA

CATCACGTTGTTTGATACTCACGCCTTGTTCGAGACGCTAACTTCTGCGCCAGAAGAGCACGGTTTCGTGAACGCG
AGCGATCCTTGTTTGGACATCAACCGCTCATCGTCTGTCGATTACATGTACACCCACGCATTGCGCTCTGAGTGTGC
AGCGTCTGGTGCTGAGAAGTTTGTGTTCTGGGATGTCACGCATCCAACAACAGCAACTCACCGCTATGTTGCAGAG
AAAATGCTAGAAAGTAGCAACAACTTAGCCGAGTACCGTTTCTAA

>930
ATGATGAAAAAAACAATCACACTATTAACTGCATTACTCCCGCTTGCTTCTGCAGTTGCCGAAGAGCCAACCTTAT
CACCAGAAATGGTTTCAGCGTCTGAAGTGATCAGCACGCAAGAAAACCAAACCTATACCTATGTTCGCTGTTGGTA
TCGCACCAGCTACTCGAAAGATGATCCAGCGACCGATTGGGAATGGGCAAAAAACGAAGATGGTAGCTACTTCAC
CATTGACGGCTACTGGTGGAGCTCCGTTTCATTTAAAAACATGTTCTACACCAACACGTCGCAAAACGTTATCCGT
CAGCGTTGTGAAGCAACATTAGATTTGGCGAACGAGAACGCAGACATTACGTTCTTCGCCGCTGACAATCGCTTCT
CATACAACCACACGATCTGGAGCAACGACGCAGCAATGCAGCCAGATCAAATCAACAAAGTGGTTGCACTCGGTG
ACAGCTTGTCTGATACAGGCAACATCTTTAACGCATCACAATGGCGCTTCCCTAACCCGAACAGCTGGTTCTTAGG
TCACTTCTCCAACGGTTTTGTTTGGACAGAATACATTGCCAAAGCGAAGAACCTTCCGCTCTACAACTGGGCAGTT
GGCGGCGCGGCTGGTGAGAACCAATACATCGCGCTAACAGGGGTTGGTGAGCAAGTTTCTTCGTACTTAACCTACG
CAAAACTGGCGAAGAACTACAAACCAGCAAACACCTTGTTTACGCTTGAGTTTGGTTTGAATGACTTCATGAACTA
CAACCGTGGCGTTCCAGAAGTGAAAGCGGATTATGCAGAAGCACTGATTCGTTTGACGGACGCAGGTGCGAAGAA
CTTCATGTTGATGACACTGCCAGATGCGACGAAAGCGCCTCAGTTTAAGTACTCAACACAAGAAGAGATCGACAA
AATTCGTGCGAAAGTGCTTGAGATGAACGAGTTCATCAAGGCACAAGCGATGTACTACAAAGCGCAAGGTTACAA
CATCACGTTGTTTGATACTCACGCCTTGTTCGAGACGCTAACTTCTGCGCCAGAAGAGCACGGTTTCGTGAACGCG
AGCGATCCTTGTTTGGACATCAACCGCTCATCGTCTGTCGATTACATGTACACCCACGCATTGCGCTCTGAGTGTGC
AGCGTCTGGTGCTGAGAAGTTTGTGTTCTGGGATGTCACGCATCCAACAACAGCAACTCACCGCTATGTTGCAGAG
AAAATGCTAGAAAGTAGCAACAACTTAGCCGAGTACCGTTTCTAA

>863
ATGATGAAAAAAACAATCACACTATTAACTGCATTACTCCCGCTTGCTTCTGCAGTTGCCGAAGAGCCAACCTTAT
CACCAGAAATGGTTTCAGCGTCTGAAGTGATCAGCACGCAAGAAAACCAAACCTATACCTATGTTCGCTGTTGGTA
TCGCACCAGCTACTCGAAAGATGATCCAGCGACCGATTGGGAATGGGCAAAAAACGAAGATGGTAGCTACTTCAC
CATTGACGGCTACTGGTGGAGCTCCGTTTCATTTAAAAACATGTTCTACACCAACACGTCGCAAAACGTTATCCGT
CAGCGTTGTGAAGCAACATTAGATTTGGCGAACGAGAACGCAGACATTACGTTCTTCGCCGCTGACAATCGCTTCT
CATACAACCACACGATCTGGAGCAACGACGCAGCAATGCAGCCAGATCAAATCAACAAAGTGGTTGCACTCGGTG
ACAGCTTGTCTGATACAGGCAACATCTTTAACGCATCACAATGGCGCTTCCCTAACCCGAACAGCTGGTTCTTAGG
TCACTTCTCCAACGGTTTTGTTTGGACAGAATACATTGCCAAAGCGAAGAACCTTCCGCTCTACAACTGGGCAGTT
GGCGGCGCGGCTGGTGAGAACCAATACATCGCGCTAACAGGGGTTGGTGAGCAAGTTTCTTCGTACTTAACCTACG
CAAAACTGGCGAAGAACTACAAACCAGCAAACACCTTGTTTACGCTTGAGTTTGGTTTGAATGACTTCATGAACTA
CAACCGTGGCGTTCCAGAAGTGAAAGCGGATTATGCAGAAGCACTGATTCGTTTGACGGACGCAGGTGCGAAGAA
CTTCATGTTGATGACACTGCCAGATGCGACGAAAGCGCCTCAGTTTAAGTACTCAACACAAGAAGAGATCGACAA
AATTCGTGCGAAAGTGCTTGAGATGAACGAGTTCATCAAGGCACAAGCGATGTACTACAAAGCGCAAGGTTACAA
CATCACGTTGTTTGATACTCACGCCTTGTTCGAGACGCTAACTTCTGCGCCAGAAGAGCACGGTTTCGTGAACGCG
AGCGATCCTTGTTTGGACATCAACCGCTCATCGTCTGTCGATTACATGTACACCCACGCATTGCGCTCTGAGTGTGC
AGCGTCTGGTGCTGAGAAGTTTGTGTTCTGGGATGTCACGCATCCAACAACAGCAACTCACCGCTATGTTGCAGAG
AAAATGCTAGAAAGTAGCAACAACTTAGCCGAGTACCGTTTCTAA

>3324
ATGATGAAAAAAACAATCACACTATTAACTGCATTACTCCCGCTTGCTTCTGCAGTTGCCGAAGAGCCAACCTTAT
CACCAGAAATGGTTTCAGCGTCTGAAGTGATCAGCACGCAAGAAAACCAAACCTATACCTATGTTCGCTGTTGGTA
TCGCACCAGCTACTCGAAAGATGATCCGGCGACCGATTGGGAATGGGCAAAAAACGAAGATGGTAGCTACTTCAC
CATTGACGGCTACTGGTGGAGCTCCGTTTCACTTAAAAACATGTTCTACACCAACACGTCGCAAAACGTTATCCGT
CAGCGTTGTGAAGCAACATTAGATTTGGCGAACGAGAACGCAGACATTACGTTCTTCGCCGCTGACAATCGCTTCT
CATACAACCACACGATCTGGAGCAACGACGCAGCAATGCAGCCAGATCAAATCAACAAAGTGGTTGCACTCGGTG
ACAGCTTGTCTGATACAGGCAACATCTTTAACGCATCACAATGGCGCTTCCCTAACCCGAACAGCTGGTTCTTAGG

TCACTTCTCCAACGGTTTTGTGTGGACAGAATACATTGCCAAAGCGAAGAACCTTCCGCTCTACAACTGGGCAGTT
GGCGGCGCGGCTGGTGAGAACCAATACATCGCGCTAACAGGGGTTGGTGATCAAGTTTCTTCGTACTTAACCTACG
CAAAACTGGCGAAGAACTACAAACCAGCAAACACCTTGTTTACGCTTGAGTTTGGTTTGAATGACTTCATGAACTA
CAACCGTGGCGTTCCAGAAGTGAAAGCAGATTATGCAGAAGCACTGATTCGTTTGACGGACGCAGGTGCGAAGAA
CTTCATGTTGATGACACTGCCAGACGCGACGAAAGCGCCTCAGTTTAAGTACTCAACACAAGAAGAGATCGACAA
AATTCGTGCGAAAGTGCTTGAGATGAACGAGTTCATCAAGGCACAAGCGATGTACTACAAAGCGCAAGGTTACAA
CATCACGTTGTTTGATACTCACGCCTTGTTCGAGACGCTAACTTCTGCGCCAGAAGAGCACGGTTTCGTGAACGCG
AGCGATCCTTGTTTGGACATCAACCGCTCATCGTCTGTCGATTACATGTACACCCACGCATTGCGCTCTGAGTGTGC
AGCGTCTGGTGCTGAGAAGTTTGTGTTCTGGGATGTCACGCATCCAACAACAGCAACTCACCGCTATGTTGCAGAG
AAAATGCTAGAAAGTAGCAACAACTTAGCCGAGTACCGTTTCTAA

>12315
ATGATGAAAAAAACAATCACACTATTAACTGCATTACTCCCGCTTGCTTCTGCAGTTGCCGAAGAGCCAACCTTAT
CACCAGAAATGGTTTCAGCGTCTGAAGTGATCAGCACGCAAGAAAACCAAACCTATACCTATGTTCGCTGTTGGTA
TCGCACCAGCTACTCGAAAGATGATCCGGCGACCGATTGGGAATGGGCAAAAAACGAAGATGGTAGCTACTTCAC
CATTGACGGCTACTGGTGGAGCTCCGTTTCACTTAAAAACATGTTCTACACCAACACGTCGCAAAACGTTATCCGT
CAGCGTTGTGAAGCAACATTAGATTTGGCGAACGAGAACGCAGACATTACGTTCTTCGCCGCTGACAATCGCTTCT
CATACAACCACACGATCTGGAGCAACGACGCAGCAATGCAGCCAGATCAAATCAACAAAGTGGTTGCACTCGGTG
ACAGCTTGTCTGATACAGGCAACATCTTTAACGCATCACAATGGCGCTTCCCTAACCCGAACAGCTGGTTCTTAGG
TCACTTCTCCAACGGTTTTGTGTGGACAGAATACATTGCCAAAGCGAAGAACCTTCCGCTCTACAACTGGGCAGTT
GGCGGCGCGGCTGGTGAGAACCAATACATCGCGCTAACAGGGGTTGGTGATCAAGTTTCTTCGTACTTAACCTACG
CAAAACTGGCGAAGAACTACAAACCAGCAAACACCTTGTTTACGCTTGAGTTTGGTTTGAATGACTTCATGAACTA
CAACCGTGGCGTTCCAGAAGTGAAAGCAGATTATGCAGAAGCACTGATTCGTTTGACGGACGCAGGTGCGAAGAA
CTTCATGTTGATGACACTGCCAGACGCGACGAAAGCGCCTCAGTTTAAGTACTCAACACAAGAAGAGATCGACAA
AATTCGTGCGAAAGTGCTTGAGATGAACGAGTTCATCAAGGCACAAGCGATGTACTACAAAGCGCAAGGTTACAA
CATCACGTTGTTTGATACTCACGCCTTGTTCGAGACGCTAACTTCTGCGCCAGAAGAGCACGGTTTCGTGAACGCG
AGCGATCCTTGTTTGGACATCAACCGCTCATCGTCTGTCGATTACATGTACACCCACGCATTGCGCTCTGAGTGTGC
AGCGTCTGGTGCTGAGAAGTTTGTGTTCTGGGATGTCACGCATCCAACAACAGCAACTCACCGCTATGTTGCAGAG
AAAATGCTAGAAAGTAGCAACAACTTAGCCGAGTACCGTTTCTAA

>EN9701173
ATGATGAAAAAAACAATCACACTATTAACTGCATTACTCCCGCTTGCTTCTGCAGTTGCCGAAGAGCCAACCTTAT
CACCAGAAATGGTTTCAGCGTCTGAAGTGATCAGCACGCAAGAAAACCAAACCTATACCTATGTTCGCTGTTGGTA
TCGCACCAGCTACTCGAAAGATGATCCGGCGACCGATTGGGAATGGGCAAAAAACGAAGATGGTAGCTACTTCAC
CATTGACGGCTACTGGTGGAGCTCCGTTTCACTTAAAAACATGTTCTACACCAACACGTCGCAAAACGTTATCCGT
CAGCGTTGTGAAGCAACATTAGATTTGGCGAACGAGAACGCAGACATTACGTTCTTCGCCGCTGACAATCGCTTCT
CATACAACCACACGATCTGGAGCAACGACGCAGCAATGCAGCCAGATCAAATCAACAAAGTGGTTGCACTCGGTG
ACAGCTTGTCTGATACAGGCAACATCTTTAACGCATCACAATGGCGCTTCCCTAACCCGAACAGCTGGTTCTTAGG
TCACTTCTCCAACGGTTTTGTGTGGACAGAATACATTGCCAAAGCGAAGAACCTTCCGCTCTACAACTGGGCAGTT
GGCGGCGCGGCTGGTGAGAACCAATACATCGCGCTAACAGGGGTTGGTGATCAAGTTTCTTCGTACTTAACCTACG
CAAAACTGGCGAAGAACTACAAACCAGCAAACACCTTGTTTACGCTTGAGTTTGGTTTGAATGACTTCATGAACTA
CAACCGTGGCGTTCCAGAAGTGAAAGCAGATTATGCAGAAGCACTGATTCGTTTGACGGACGCAGGTGCGAAGAA
CTTCATGTTGATGACACTGCCAGACGCGACGAAAGCGCCTCAGTTTAAGTACTCAACACAAGAAGAGATCGACAA
AATTCGTGCGAAAGTGCTTGAGATGAACGAGTTCATCAAGGCACAAGCGATGTACTACAAAGCGCAAGGTTACAA
CATCACGTTGTTTGATACTCACGCCTTGTTCGAGACGCTAACTTCTGCGCCAGAAGAGCACGGTTTCGTGAACGCG
AGCGATCCTTGTTTGGACATCAACCGCTCATCGTCTGTCGATTACATGTACACCCACGCATTGCGCTCTGAGTGTGC
AGCGTCTGGTGCTGAGAAGTTTGTGTTCTGGGATGTCACGCATCCAACAACAGCAACTCACCGCTATGTTGCAGAG
AAAATGCTAGAAAGTAGCAACAACTTAGCCGAGTACCGTTTCTAA

>EN9901310

ATGATGAAAAAAACAATCACACTATTAACTGCATTACTCCCGCTTGCTTCTGCAGTTGCCGAAGAGCCAACCTTAT
CACCAGAAATGGTTTCAGCGTCTGAAGTGATCAGCACGCAAGAAAACCAAACCTATACCTATGTTCGCTGTTGGTA
TCGCACCAGCTACTCGAAAGATGATCCGGCGACCGATTGGGAATGGGCAAAAAACGAAGATGGTAGCTACTTCAC
CATTGACGGCTACTGGTGGAGCTCCGTTTCACTTAAAAACATGTTCTACACCAACACGTCGCAAAACGTTATCCGT
CAGCGTTGTGAAGCAACATTAGATTTGGCGAACGAGAACGCAGACATTACGTTCTTCGCCGCTGACAATCGCTTCT
CATACAACCACACGATCTGGAGCAACGACGCAGCAATGCAGCCAGATCAAATCAACAAAGTGGTTGCACTCGGTG
ACAGCTTGTCTGATACAGGCAACATCTTTAACGCATCACAATGGCGCTTCCCTAACCCGAACAGCTGGTTCTTAGG
TCACTTCTCCAACGGTTTTGTGTGGACAGAATACATTGCCAAAGCGAAGAACCTTCCGCTCTACAACTGGGCAGTT
GGCGGCGCGGCTGGTGAGAACCAATACATCGCGCTAACAGGGGTTGGTGATCAAGTTTCTTCGTACTTAACCTACG
CAAAACTGGCGAAGAACTACAAACCAGCAAACACCTTGTTTACGCTTGAGTTTGGTTTGAATGACTTCATGAACTA
CAACCGTGGCGTTCCAGAAGTGAAAGCAGATTATGCAGAAGCACTGATTCGTTTGACGGACGCAGGTGCGAAGAA
CTTCATGTTGATGACACTGCCAGACGCGACGAAAGCGCCTCAGTTTAAGTACTCAACACAAGAAGAGATCGACAA
AATTCGTGCGAAAGTGCTTGAGATGAACGAGTTCATCAAGGCACAAGCGATGTACTACAAAGCGCAAGGTTACAA
CATCACGTTGTTTGATACTCACGCCTTGTTCGAGACGCTAACTTCTGCGCCAGAAGAGCACGGTTTCGTGAACGCG
AGCGATCCTTGTTTGGACATCAACCGCTCATCGTCTGTCGATTACATGTACACCCACGCATTGCGCTCTGAGTGTGC
AGCGTCTGGTGCTGAGAAGTTTGTGTTCTGGGATGTCACGCATCCAACAACAGCAACTCACCGCTATGTTGCAGAG
AAAATGCTAGAAAGTAGCAACAACTTAGCCGAGTACCGTTTCTAA

>3631
ATGATGAAAAAAACAATCACACTATTAACTGCATTACTCCCGCTTGCTTCTGCAGTTGCCGAAGAGCCAA
CCTTATCACCAGAAATGGTTTCAGCGTCTGAAGTGATCAGCACGCAAGAAAACCAAACCTATACCTATGT
TCGCTGTTGGTATCGCACCAGCTACTCGAAAGATGATCCGGCGACCGATTGGGAATGGGCAAAAAACGAA
GATGGTAGCTACTTCACCATTGACGGCTACTGGTGGAGCTCCGTTTCATTTAAAAACATGTTCTACACCA
ACACGTCGCAAAACGTTATCCGTCAGCGTTGTGAAGCCACATTAGATTTGGCGAACGAGAACGCAGACAT
TACGTTCTTCGCCGCTGACAATCGCTTCTCATACAACCACACGATCTGGAGCAACGACGCAGCAATGCAG
CCAGATCAAATCAACAAAGTGGTTGCACTCGGTGACAGCTTGTCTGATACAGGCAACATCTTTAACGCAT
CACAATGGCGCTTCCCTAACCCGAACAGCTGGTTCTTAGGTCACTTCTCCAACGGTTTTGTGTGGACAGA
ATACATTGCCAAAGCGAAGAACCTTCCGCTCTACAACTGGGCAGTTGGCGGCGCGGCTGGTGAGAACCAA
TACATCGCGCTAACAGGGGTTGGTGATCAAGTTTCTTCGTACTTAACCTACGCAAAACTGGCGAAGAACT
ACAAACCAGCAAACACCTTGTTTACGCTTGAGTTTGGTTTGAATGACTTCATGAACTACAACCGTGGCGT
TCCAGAAGTGAAAGCAGATTATGCAGAAGCACTGATTCGTTTGACGGACGCAGGTGCGAAGAACTTCATG
TTGATGACACTGCCAGACGCGACGAAAGCGCCTCAGTTTAAGTACTCAACACAAGAAGAGATCGACAAAA
TTCGTGCGAAAGTGCTTGAGATGAACGAGTTCATCAAGGCACAAGCGATGTACTACAAAGCGCAAGGTTA
CAACATCACGTTGTTTGATACTCACGCCTTGTTCGAGACGCTAACTTCTGCGCCCGAAAAGCACGGTTTC
GTGAACGCGAGTGATCCTTGTTTGGACATCAACCGCTCATCGTCTGTCGATTACATGTACACCCACGCAT
TGCGCTCTGAGTGTGCGGCGTCTGGTGCTGAGAAGTTTGTGTTCTGGGATGTCACGCACCCAACAACAGC
AACTCACCGCTATGTTGCAGAGAAAATGCTAGAAAGTAGCAACAACTTAGCCGAGTACCGTTTCTAA

>VP766
ATGATGAAAAAAACAATCACACTATTAACTGCATTACTCCCGCTTGCTTCTGCAGTTGCCGAAGAGCCAA
CCTTATCACCAGAAATGGTTTCAGCGTCTGAAGTGATCAGCACGCAAGAAAACCAAACCTATACCTATGT
TCGCTGTTGGTATCGCACCAGCTACTCGAAAGATGATCCGGCGACCGATTGGGAATGGGCAAAAAACGAA
GATGGTAGCTACTTCACCATTGACGGCTACTGGTGGAGCTCCGTTTCATTTAAAAACATGTTCTACACCA
ACACGTCGCAAAACGTTATCCGTCAGCGTTGTGAAGCCACATTAGATTTGGCGAACGAGAACGCAGACAT
TACGTTCTTCGCCGCTGACAATCGCTTCTCATACAACCACACGATCTGGAGCAACGACGCAGCAATGCAG
CCAGATCAAATCAACAAAGTGGTTGCACTCGGTGACAGCTTGTCTGATACAGGCAACATCTTTAACGCAT
CACAATGGCGCTTCCCTAACCCGAACAGCTGGTTCTTAGGTCACTTCTCCAACGGTTTTGTGTGGACAGA
ATACATTGCCAAAGCGAAGAACCTTCCGCTCTACAACTGGGCAGTTGGCGGCGCGGCTGGTGAGAACCAA
TACATCGCGCTAACAGGGGTTGGTGATCAAGTTTCTTCGTACTTAACCTACGCAAAACTGGCGAAGAACT
ACAAACCAGCAAACACCTTGTTTACGCTTGAGTTTGGTTTGAATGACTTCATGAACTACAACCGTGGCGT
TCCAGAAGTGAAAGCAGATTATGCAGAAGCACTGATTCGTTTGACGGACGCAGGTGCGAAGAACTTCATG

TTGATGACACTGCCAGACGCGACGAAAGCGCCTCAGTTTAAGTACTCAACACAAGAAGAGATCGACAAAA
TTCGTGCGAAAGTGCTTGAGATGAACGAGTTCATCAAGGCACAAGCGATGTACTACAAAGCGCAAGGTTA
CAACATCACGTTGTTTGATACTCACGCCTTGTTCGAGACGCTAACTTCTGCGCCCGAAAAGCACGGTTTC
GTGAACGCGAGTGATCCTTGTTTGGACATCAACCGCTCATCGTCTGTCGATTACATGTACACCCACGCAT
TGCGCTCTGAGTGTGCGGCGTCTGGTGCTGAGAAGTTTGTGTTCTGGGATGTCACGCACCCAACAACAGC
AACTCACCGCTATGTTGCAGAGAAAATGCTAGAAAGTAGCAACAACTTAGCCGAGTACCGTTTCTAA

>3355
ATGATGAAAAAAACAATCACACTATTAACTGCATTACTCCCGCTTGCTTCTGCAGTTGCCGAAGAGCCAACCTTAT
CACCAGAAATGGTTTCAGCGTCTGAAGTGTTCAGCACGCAAGAAAACCAAACCTATACCTATGTTCGCTGTTGGTA
TCGCACCAGCTACTCGAAAGATGACCCGGCGACCGATTGGGAATGGGCAAAAAACGAAGATGGTAGCTACTTCAC
CATTGACGGCTACTGGTGGAGCTCCGTTTCATTTAAAAACATGTTCTACACCAACACGTCGCAAAACGTTATCCGT
CAGCGTTGTGAAGCAACATTAGATTTGGCGAACGAGAACGCAGACATTACGTTCTTCGCCGCTGACAATCGCTTCT
CATACAACCACACGATCTGGAGCAACGACGCAGCAATGCAGCCAGATCAAATCAACAAAGTGGTTGCACTCGGTG
ACAGCTTGTCTGATACAGGCAACATCTTTAACGCATCACAATGGCGCTTCCCTAACCCGAACAGCTGGTTCTTAGG
TCACTTCTCCAACGGTTTTGTGTGGACAGAATACATTGCCAAAGCGAAGAACCTTCCGCTCTACAACTGGGCAGTT
GGCGGCGCGGCTGGTGAGAACCAATACATCGCGCTAACAGGGGTTGGTGATCAAGTTTCTTCGTACTTAACCTACG
CAAAACTGGCGAAGAACTACAAACCAGCAAACACCTTGTTTACGCTTGAGTTTGGTTTGAATGACTTCATGAACTA
CAACCGTGGCGTTCCAGAAGTGAAAGCGGATTATGCAGAAGCACTGATTCGTTTGACGGACGCAGGTGCGAAGAA
CTTCATGTTGATGACACTGCCAGACGCGACGAAAGCGCCTCAGTTTAAGTACTCAACACAAGAAGAGATCGACAA
AATTCGTGCGAAAGTGCTTGAGATGAACGAGTTCATCAAGGCACAAGCGATGTACTACAAAGCGCAAGGTTACAA
CATCACGTTGTTTGATACTCACGCCTTGTTCGAGACGCTAACTTCTGCGCCAGAAGAGCACGGTTTCGTGAACGCG
AGCGATCCTTGTTTGGACATCAACCGCTCATCGTCTGTCGATTACATGTACACCCACGCATTGCGCTCTGAGTGTGC
GGCGTCCGGTGCTGAGAAATTTGTGTTCTGGGATGTCACGCACCCAACAACAGCAACTCACCGCTATGTTGCAGAG
AAAATGCTAGAAAGTAGCAACAACTTAGCCGAGTACCGTTTCTAA

>49
ATGATGAAAAAAACAATCACACTATTAACTGCATTACTCCCGCTTGCTTCTGCAGTTGCCGAAGAGCCAACCTTAT
CACCAGAAATGGTTTCAGCGTCTGAAGTGATCAGCACGCAAGAAAACCAAACCTATACCTATGTTCGCTGTTGGTA
TCGCACCAGCTACTCGAAAGATGATCCGGCGACCGATTGGGAATGGGCAAAAAACGAAGATGGTAGCTACTTCAC
CATTGACGGCTACTGGTGGAGCTCCGTTTCATTTAAAAACATGTTCTACACCAACACGTCGCAAAACGTTATCCGT
CAGCGTTGTGAAGCCACATTAGATTTGGCGAACGAGAACGCAGACATTACGTTCTTCGCCGCTGACAATCGCTTCT
CATACAACCACACGATCTGGAGCAACGACGCAGCAATGCAGCCAGATCAAATCAACAAAGTGGTTGCACTCGGTG
ACAGCTTGTCTGATACAGGCAACATCTTTAACGCATCACAATGGCGCTTCCCTAACCCGAATAGCTGGTTCTTAGG
TCACTTCTCCAACGGTTTTGTGTGGACAGAATACATTGCCAAAGCGAAGAACCTTCCACTCTACAACTGGGCAGTT
GGCGGCGCGGCTGGTGAGAACCAATACATCGCGCTAACAGGTTGGTGATCAAGTTTCTTCGTACTTAACCTACGCA
AAACTGGCGAAGAACTACAAACCAGCAAACACCTTGTTTACGCTTGAGTTTGGTTTGAATGACTTCATGAACTACA
ACCGTGGCGTTCCAGAAGTGAAAGCAGATTATGCAGAAGCACTGATTCGTTTGACGGACGCAGGTGCGAAGAACT
TCATGTTGATGACACTGCCAGACGCGACGAAAGCGCCTCAGTTTAAGTACTCAACACAAGAAGAGATCGACAAAA
TTCGTGCGAAAGTGCTTGAGATGAACGAGTTCATCAAGGCACAAGCGATGTACTACAAAGCGCAAGGTTACAACA
TAACGTTGTTTGATACTCACGCCTTGTTCGAGACGCTAACTTCTGCGCCCGAAGAGCACGGTTTCGTGAACGCGAG
CGATCCTTGTTTGGACATCAACCGCTCATCGTCTGTCGATTACATGTACACCCACGCATTGCGCTCTGAGTGTGCAG
CGTCTGGTGCTGAGAAGTTTGTGTTCTGGGATGTCACGCACCCAACAACAGCAACTCACCGCTATGTTGCAGAGAA
AATGCTAGAAAGTAGCAACAACTTAGCCGAGTACCGTTTCTAA

>901128
ATGATGAAAAAAACAATCACACTATTAACTGCATTACTCCCGCTTGCTTCTGCAGTTGCCGAAGAGCCAACCTTAT
CACCAGAAATGGTTTCAGCGTCTGAAGTGATCAGCACGCAAGAAAACCAAACCTATACCTATGTTCGCTGTTGGTA
TCGCACCAGCTACTCGAAAGATGATCCGGCGACCGATTGGGAATGGGCAAAAAACGAAGATGGTAGCTACTTCAC
CATTGACGGCTACTGGTGGAGCTCCGTTTCATTTAAAAACATGTTCTACACCAACACGTCGCAAAACGTTATCCGT
CAGCGTTGTGAAGCAACATTAGATTTGGCGAACGAGAACGCAGACATTACGTTCTTCGCCGCTGACAATCGCTTCT

CATACAACCACACGATCTGGAGCAACGACGCAGCAATGCAGCCAGATCAAATCAACAAAGTGGTTGCACTCGGTG
ACAGCTTGTCTGATACAGGCAACATCTTTAACGCATCACAATGGCGCTTCCCTAACCCGAACAGCTGGTTCTTAGG
TCACTTCTCCAACGGTTTTGTGTGGACAGAATACATTGCCAAAGCGAAGAACCTTCCGCTCTACAACTGGGCAGTT
GGCGGCGCGGCTGGTGAGAACCAATACATCGCGCTAACAGGGGTTGGTGATCAAGTTTCTTCGTACTTAACCTACG
CAAAACTGGCGAAGAACTACAAACCAGCAAACACCTTGTTTACGCTTGAGTTTGGTTTGAATGACTTCATGAACTA
CAACCGTGGCGTTCCAGAAGTGAAAGCGGATTATGCAGAAGCACTGATTCGTTTGACGGACGCAGGTGCGAAGAA
CTTCATGTTGATGACACTGCCAGACGCGACGAAAGCGCCTCAGTTTAAGTACTCAACACAAGAAGAGATCGACAA
AATTCGTGCGAAAGTGCTTGAGATGAACGAGTTCATCAAGGCACAAGCGATGTACTACAAAGCGCAAGGTTACAA
CATCACGTTGTTTGATACTCACGCCTTGTTCGAGACGCTAACTTCTGCGCCAGAAGAGCACGGTTTCGTGAACGCG
AGCGATCCTTGTTTGGACATCAACCGCTCATCGTCTGTCGATTACATGTACACCCACGCATTGCGTTCTGAGTGTGC
AGCGTCTGGTGCTGAGAAGTTTGTGTTCTGGGATGTCACGCATCCAACAACAGCAACTCACCGCTATGTTGCAGAG
AAAATGCTAGAAAGTAGCAACAACTTAGCAGAGTACCGTTTCTAA

>EN2910
ATGATGAAAAAAACAATCACACTATTAACTGCATTACTCCCGCTTGCTTCTGCAGTTGCCGAAGAGCCAACCTTAT
CACCAGAAATGGTTTCAGCGTCTGAAGTGATCAGCACGCAAGAAAACCAAACCTATACCTATGTTCGCTGTTGGTA
TCGCACCAGCTACTCGAAAGATGATCCGGCGACCGATTGGGAATGGGCAAAAAACGAAGATGGTAGCTACTTCAC
CATTGACGGCTACTGGTGGAGCTCCGTTTCACTTAAAAACATGTTCTACACCAACACGTCGCAAAACGTTATCCGT
CAGCGTTGTGAAGCAACATTAGATTTGGCGAACGAGAACGCAGACATTACGTTCTTCGCCGCTGACAATCGCTTCT
CATACAACCACACGATCTGGAGCAACGACGCAGCAATGCAGCCAGATCAAATCAACAAAGTGGTTGCACTCGGTG
ACAGCTTGTCTGATACAGGCAACATCTTTAACGCATCACAATGGCGCTTCCCTAACCCGAACAGCTGGTTCTTAGG
TCACTTCTCCAACGGTTTTGTGTGGACAGAATACATTGCCAAAGCGAAGAACCTTCCGCTCTACAACTGGGCAGTT
GGCGGCGCGGCTGGTGAGAACCAATACATCGCGCTAACAGGGGTTGGTGATCAAGTTTCTTCGTACTTAACCTACG
CAAAACTGGCGAAGAACTACAAACCAGCAAACACCTTGTTTACGCTTGAGTTTGGTTTGAATGACTTCATGAACTA
CAACCGTGGCGTTCCAGAAGTGAAAGCAGATTATGCAGAAGCACTGATTCGTTTGACGGACGCAGGTGCGAAGAA
CTTCATGTTGATGACACTGCCAGACGCGACGAAAGCGCCTCAGTTTAAGTACTCAACACAAGAAGAGATCGACAA
AATTCGTGCGAAAGTGCTTGAGATGAACGAGTTCATCAAGGCACAAGCGATGTACTACAAAGCGCAAGGTTACAA
CATCACGTTGTTTGATACTCACGCCTTGTTCGAGACGCTAACTTCTGCGCCAGAAGAGCACGGTTTCGTGAACGCG
AGCGATCCTTGTTTGGACATCAACCGCTCATCGTCTGTCGATTACATGTACACCCACGCATTGCGCTCTGAGTGTGC
AGCGTCTGGTGCTGAGAAGTTTGTGTTCTGGGATGTCACGCATCCAACAACAGCAACTCACCGCTATGTTGCAGAG
AAAATGCTAGAAAGTAGCAACAACTTAGCCGAGTACCGTTTCTAA

>EN9701072
ATGATGAAAAAAACAATCACACTATTAACTGCATTACTCCCGCTTGCTTCTGCAGTTGCCGAAGAGCCAACCTTAT
CACCAGAAATGGTTTCAGCGTCTGAAGTGATCAGCACGCAAGAAAACCAAACCTATACCTATGTTCGCTGTTGGTA
TCGCACCAGCTACTCGAAAGATGATCCGGCGACCGATTGGGAATGGGCAAAAAACGAAGATGGTAGCTACTTCAC
CATTGACGGCTACTGGTGGAGCTCCGTTTCATTTAAAAACATGTTCTACACCAACACGTCGCAAAACGTTATCCGT
CAGCGTTGTGAAGCAACATTAGATTTGGCGAACGAGAACGCAGACATTACGTTCTTCGCCGCTGACAATCGCTTCT
CATACAACCACACGATCTGGAGCAACGACGCAGCAATGCAGCCAGATCAAATCAACAAAGTGGTTGCACTCGGTG
ACAGCTTGTCTGATACAGGCAACATCTTTAACGCATCACAATGGCGCTTCCCTAACCCGAACAGCTGGTTCTTAGG
TCACTTCTCCAACGGTTTTGTGTGGACAGAATACATTGCCAAAGCGAAGAACCTTCCGCTCTACAACTGGGCAGTT
GGCGGTGCGGCTGGTGAGAACCAATACATCGCGCTAACAGGGGTTGGTGATCAAGTTTCTTCGTACTTAACCTACG
CAAAACTGGCGAAGAACTACAAACCAGCAAACACCTTGTTTACGCTTGAGTTTGGTTTGAATGACTTCATGAACTA
CAACCGTGGCGTTCCAGAAGTGAAAGCGGATTATGCAGAAGCACTGATTCGTTTGACGGACGCAGGTGCGAAGAA
CTTCATGTTGATGACACTGCCAGATGCGACGAAAGCGCCTCAGTTTAAGTACTCAACACAAGAAGAGATCGACAA
AATTCGTGCGAAAGTGCTTGAGATGAACGAGTTCATCAAGGCACAAGCGATGTACTACAAAGCGCAAGGTTACAA
CATCACGTTGTTTGATACTCACGCCTTGTTCGAGACGCTAACTTCTGCGCCAGAAGAGCACGGTTTCGTGAACGCG
AGCGATCCTTGTTTGGACATCAACCGCTCATCGTCTGTCGATTACATGTACACCCACGCATTGCGCTCTGAGTGTGC
AGCGTCTGGTGCTGAGAAGTTTGTGTTCTGGGATGTCACGCACCCAACAACAGCAACTCACCGCTATGTTGCAGAG
AAAATGCTAGAAAGTAGCAACAACTTAGAAGAGTTTCGCTTTTAA

>EN9701121
ATGATGAAAAAAACAATCACACTATTAACTGCATTACTCCCGCTTGCTTCTGCAGTTGCCGAAGAGCCAACCTTAT
CACCAGAAATGGTTTCAGCGTCTGAAGTGATCAGCACGCAAGAAAACCAAACCTATACCTATGTTCGCTGTTGGTA
TCGCACCAGCTACTCGAAAGATGATCCGGCGACCGGTTGGGAATGGGCAAAAAACGAAGATGGTAGCTACTTCAC
CATTGACGGCTACTGGTGGAGCTCCGTTTCATTTAAAAACATGTTCTACACCAACACGTCGCAAAACGTTATCCGT
CAGCGTTGTGAAGCAACATTAGATTTGGCGAACGAGAACGCAGACATTACGTTCTTCGCCGCTGACAATCGCTTCT
CATACAACCACACGATCTGGAGCAACGACGCAGCAATGCAGCCAGATCAAATCAACAAAGTGGTTGCACTCGGTG
ACAGCTTGTCTGATACAGGCAACATCTTTAACGCATCACAATGGCGCTTCCCTAACCCGAACAGCTGGTTCTTAGG
TCACTTCTCGAACGGTTTTGTGTGGACAGAATACATTGCCAAAGCGAAGAACCTTCCGCTCTACAACTGGGCAGTT
GGCGGCGCGGCTGGTGAGAACCAATACATCGCGCTAACAGGGGTTGGTGATCAAGTTTCTTCGTACTTAACCTACG
CAAAACTGGCGAAGAACTACAAACCAGCAAACACCTTGTTTACGCTTGAGTTTGGTTTGAATGACTTCATGAACTA
CAACCGTGGCGTTCCAGAAGTGAAAGCGGATTATGCAGAAGCACTGATTCGTTTGACGGACGCAGGTGCGAAGAA
CTTCATGTTGATGACACTGCCAGACGCGACGAAAGCGCCTCAGTTTAAGTACTCAACACAAGAAGAGATCGACAA
AATTCGTGCGAAAGTGCTTGAGATGAACGAGTTCATCAAGGCACAAGCGATGTACTACAAAGCGCAAGGTTACAA
CATCACGTTGTTTGATACTCACGCCTTGTTCGAGACGCTAACTTCTGCGCCAGAAGAGCACGGTTTCGTGAACGCG
AGCGATCCTTGTTTGGACATCAACCGCTCATCGTCTGTCGACTACATGTACACCCACGCATTGCGCTCTGAGTGTGC
GGCGTCTGGTGCTGAGAAGTTTGTATTCTGGGATGTCACGCACCCAACAACAGCAACTCACCGCTATGTTGCAGAG
AAAATGCTAGAAAGTAGCAACAACTTAGCCGAGTACCGTTTCTAA

>3644
ATGATGAAAAAAACAATCACACTATTAACTGCATTACTCCCGCTTGCTTCTGCAGTTGCCGAAGAGCCAA
CCTTATCACCAGAAATGGTTTCAGCGTCTGAAGTGATCAGCACGCAAGAAAACCAAACCTATACCTATGT
TCGCTGTTGGTATCGCACCAGCTACTCGAAAGATGATCCGGCGACCGATTGGGAATGGGCAAAAAACGAA
GATGGTAGCTACTTCACCATTGACGGCTACTGGTGGAGCTCCGTTTCATTTAAAAACATGTTCTACACCA
ACACGTCGCAAAACGTTATCCGTCAGCGTTGTGAAGCAACATTAGATTTGGCGAACGAGAACGCAGACAT
TACGTTCTTCGCCGCTGACAATCGCTTCTCATACAACCACACGATCTGGAGCAACGACGCAGCAATGCAG
CCAGATCAAATCAACAAAGTGGTTGCACTCGGTGACAGCTTGTCTGATACAGGCAACATCTTTAACGCAT
CACAATGGCGCTTCCCTAACCCGAACAGCTGGTTCTTAGGTCACTTCTCCAACGGTTTTGTGTGGACAGA
ATACATTGCCAAAGCGAAGAACCTTCCGCTCTACAACTGGGCAGTTGGCGGTGCGGCTGGTGAGAACCAA
TACATCGCGCTAACAGGGGTTGGTGATCAAGTTTCTTCGTACTTAACCTACGCAAAACTGGCGAAGAACT
ACAAACCAGCAAACACCTTGTTTACGCTTGAGTTTGGTTTGAATGACTTCATGAACTACAACCGTGGCGT
TCCAGAAGTGAAAGCGGATTATGCAGAAGCACTGATTCGTTTGACGGACGCAGGTGCGAAGAACTTCATG
TTGATGACACTGCCAGATGCGACGAAAGCGCCTCAGTTTAAGTACTCAACACAAGAAGAGATCGACAAAA
TTCGTGCGAAAGTGCTTGAGATGAACGAGTTCATCAAGGCACAAGCGATGTACTACAAAGCGCAAGGTTA
CAACATCACGTTGTTTGATACTCACGCCTTGTTCGAGACGCTAACTTCTGCGCCAGAAGAGCACGGTTTC
GTGAACGCGAGCGATCCTTGTTTGGACATCAACCGCTCATCGTCTGTCGATTACATGTACACCCACGCAT
TGCGCTCTGAGTGTGCAGCGTCTGGTGCTGAGAAGTTTGTGTTCTGGGATGTCACGCACCCAACAACAGC
AACTCACCGCTATGTTGCAGAGAAAATGCTAGAAAGTAGCAACAACTTAGAAGAGTTTCGCTTTTAA

>3646
ATGATGAAAAAAACAATCACACTATTAACTGCATTACTCCCGCTTGCTTCTGCAGTTGCCGAAGAGCCAA
CCTTATCACCAGAAATGGTTTCAGCGTCTGAAGTGATCAGCACGCAAGAAAACCAAACCTATACCTATGT
TCGCTGTTGGTATCGCACCAGCTACTCGAAAGATGATCCGGCGACCGATTGGGAATGGGCAAAAAACGAA
GATGGTAGCTACTTCACCATTGACGGCTACTGGTGGAGCTCCGTTTCATTTAAAAACATGTTCTACACCA
ACACGTCGCAAAACGTTATCCGTCAGCGTTGTGAAGCCACATTAGATTTGGCGAACGAGAACGCAGACAT
TACGTTCTTCGCCGCTGACAATCGCTTCTCATACAACCACACGATCTGGAGCAACGACGCAGCAATGCAG
CCAGATCAAATCAACAAAGTGGTTGCACTCGGTGACAGCTTGTCTGATACAGGCAACATCTTTAACGCAT
CACAATGGCGCTTCCCTAACCCGAACAGCTGGTTCTTAGGTCACTTCTCCAACGGTTTTGTGTGGACAGA
ATACATTGCCAAAGCGAAGAACCTTCCGCTCTACAACTGGGCAGTTGGCGGCGCGGCTGGTGAGAACCAA
TACATCGCGCTAACAGGGGTTGGTGATCAAGTTTCTTCGTACTTAACCTACGCAAAACTGGCGAAGAACT

ACAAACCAGCAAACACCTTGTTTACGCTTGAGTTTGGTTTGAATGACTTCATGAACTACAACCGTGGCGT
TCCAGAAGTGAAAGCAGATTATGCAGAAGCACTGATTCGTTTGACGGACGCAGGTGCGAAGAACTTCATG
TTGATGACACTGCCAGACGCGACGAAAGCGCCTCAGTTTAAGTACTCAACACAAGAAGAGATCGACAAAA
TTCGTGCGAAAGTGCTTGAGATGAACGAGTTCATCAAGGCACAAGCGATGTACTACAAAGCGCAAGGTTA
CAACATCACGTTGTTTGATACTCACGCCTTGTTCGAGACGCTAACTTCTGCGCCCGAAAAGCACGGTTTC
GTGAACGCGAGTGATCCTTGTTTGGACATCAACCGCTCATCGTCTGTCGATTACATGTACACCCACGCAT
TGCGCTCTGAGTGTGCGGCGTCTGGTGCTGAGAAGTTTGTGTTCTGGGATGTCACGCACCCAACAACAGC
AACTCACCGCTATGTTGCAGAGAAAATGCTAGAAAGTAGCAACAACTTAGCCGAGTACCGTTTCTAA

>10-4298
ATGATGAAAAAAACAATCACACTATTAACTGCATTACTCCCGCTTGCTTCTGCAGTTGCCGAAGAGCCAA
CCTTATCACCAGAAATGGTTTCAGCGTCTGAAGTGATCAGCACGCAAGAAAACCAAACCTATACCTATGT
TCGCTGTTGGTATCGCACCAGCTACTCGAAAGATGATCCGGCGACCGATTGGGAATGGGCAAAAAACGAA
GATGGTAGCTACTTCACCATTGACGGCTACTGGTGGAGCTCCGTTTCACTTAAAAACATGTTCTACACCA
ACACGTCGCAAAACGTTATCCGTCAGCGTTGTGAAGCAACATTAGATTTGGCGAACGAGAACGCAGACAT
TACGTTCTTCGCCGCTGACAATCGCTTCTCATACAACCACACGATCTGGAGCAACGACGCAGCAATGCAG
CCAGATCAAATCAACAAAGTGGTTGCACTCGGTGACAGCTTGTCTGATACAGGCAACATCTTTAACGCAT
CACAATGGCGCTTCCCTAACCCGAACAGCTGGTTCTTAGGTCACTTCTCCAACGGTTTTGTGTGGACAGA
ATACATTGCCAAAGCGAAGAACCTTCCGCTCTACAACTGGGCAGTTGGCGGCGCGGCTGGTGAGAACCAA
TACATCGCGCTAACAGGGGTTGGTGATCAAGTTTCTTCGTACTTAACCTACGCAAAACTGGCGAAGAACT
ACAAACCAGCAAACACCTTGTTTACGCTTGAGTTTGGTTTGAATGACTTCATGAACTACAACCGTGGCGT
TCCAGAAGTGAAAGCAGATTATGCAGAAGCACTGATTCGTTTGACGGACGCAGGTGCGAAGAACTTCATG
TTGATGACACTGCCAGACGCGACGAAAGCGCCTCAGTTTAAGTACTCAACACAAGAAGAGATCGACAAAA
TTCGTGCGAAAGTGCTTGAGATGAACGAGTTCATCAAGGCACAAGCGATGTACTACAAAGCGCAAGGTTA
CAACATCACGTTGTTTGATACTCACGCCTTGTTCGAGACGCTAACTTCTGCGCCAGAAGAGCACGGTTTC
GTGAACGCGAGCGATCCTTGTTTGGACATCAACCGCTCATCGTCTGTCGATTACATGTACACCCACGCAT
TGCGCTCTGAGTGTGCAGCGTCTGGTGCTGAGAAGTTTGTGTTCTGGGATGTCACGCATCCAACAACAGC
AACTCACCGCTATGTTGCAGAGAAAATGCTAGAAAGTAGCAACAACTTAGCCGAGTACCGTTTCTAA

>10-4303
ATGATGAAAAAAACAATCACACTATTAACTGCATTACTCCCGCTTGCTTCTGCAGTTGCCGAAGAGCCAACCTTAT
CACCAGAAATGGTTTCAGCGTCTGAAGTGATCAGCACGCAAGAAAACCAAACCTATACCTATGTTCGCTGTTGGTA
TCGCACCAGCTACTCGAAAGATGATCCGGCGACCGATTGGGAATGGGCAAAAAACGAAGATGGTAGCTACTTCAC
CATTGACGGCTACTGGTGGAGCTCCGTTTCACTTAAAAACATGTTCTACACCAACACGTCGCAAAACGTTATCCGT
CAGCGTTGTGAAGCAACATTAGATTTGGCGAACGAGAACGCAGACATTACGTTCTTCGCCGCTGACAATCGCTTCT
CATACAACCACACGATCTGGAGCAACGACGCAGCAATGCAGCCAGATCAAATCAACAAAGTGGTTGCACTCGGTG
ACAGCTTGTCTGATACAGGCAACATCTTTAACGCATCACAATGGCGCTTCCCTAACCCGAACAGCTGGTTCTTAGG
TCACTTCTCCAACGGTTTTGTGTGGACAGAATACATTGCCAAAGCGAAGAACCTTCCGCTCTACAACTGGGCAGTT
GGCGGCGCGGCTGGTGAGAACCAATACATCGCGCTAACAGGGGTTGGTGATCAAGTTTCTTCGTACTTAACCTACG
CAAAACTGGCGAAGAACTACAAACCAGCAAACACCTTGTTTACGCTTGAGTTTGGTTTGAATGACTTCATGAACTA
CAACCGTGGCGTTCCAGAAGTGAAAGCAGATTATGCAGAAGCACTGATTCGTTTGACGGACGCAGGTGCGAAGAA
CTTCATGTTGATGACACTGCCAGACGCGACGAAAGCGCCTCAGTTTAAGTACTCAACACAAGAAGAGATCGACAA
AATTCGTGCGAAAGTGCTTGAGATGAACGAGTTCATCAAGGCACAAGCGATGTACTACAAAGCGCAAGGTTACAA
CATCACGTTGTTTGATACTCACGCCTTGTTCGAGACGCTAACTTCTGCGCCAGAAGAGCACGGTTTCGTGAACGCG
AGCGATCCTTGTTTGGACATCAACCGCTCATCGTCTGTCGATTACATGTACACCCACGCATTGCGCTCTGAGTGTGC
AGCGTCTGGTGCTGAGAAGTTTGTGTTCTGGGATGTCACGCATCCAACAACAGCAACTCACCGCTATGTTGCAGAG
AAAATGCTAGAAAGTAGCAACAACTTAGCCGAGTACCGTTTCTAA

>10-7197

ATGATGAAAAAAACAATCACACTATTAACTGCATTACTCCCGCTTGCTTCTGCAGTTGCCGAAGAGCCAACCTTAT
CACCAGAAATGGTTTCAGCGTCTGAAGTGATCAGCACGCAAGAAAACCAAACCTATACCTATGTTCGCTGTTGGTA
TCGCACCAGCTACTCGAAAGATGATCCGGCGACCGATTGGGAATGGGCAAAAAACGAAGATGGTAGCTACTTCAC
CATTGACGGCTACTGGTGGAGCTCCGTTTCACTTAAAAACATGTTCTACACCAACACGTCGCAAAACGTTATCCGT
CAGCGTTGTGAAGCAACATTAGATTTGGCGAACGAGAACGCAGACATTACGTTCTTCGCCGCTGACAATCGCTTCT
CATACAACCACACGATCTGGAGCAACGACGCAGCAATGCAGCCAGATCAAATCAACAAAGTGGTTGCACTCGGTG
ACAGCTTGTCTGATACAGGCAACATCTTTAACGCATCACAATGGCGCTTCCCTAACCCGAACAGCTGGTTCTTAGG
TCACTTCTCCAACGGTTTTGTGTGGACAGAATACATTGCCAAAGCGAAGAACCTTCCGCTCTACAACTGGGCAGTT
GGCGGCGCGGCTGGTGAGAACCAATACATCGCGCTAACAGGGGTTGGTGATCAAGTTTCTTCGTACTTAACCTACG
CAAAACTGGCGAAGAACTACAAACCAGCAAACACCTTGTTTACGCTTGAGTTTGGTTTGAATGACTTCATGAACTA
CAACCGTGGCGTTCCAGAAGTGAAAGCAGATTATGCAGAAGCACTGATTCGTTTGACGGACGCAGGTGCGAAGAA
CTTCATGTTGATGACACTGCCAGACGCGACGAAAGCGCCTCAGTTTAAGTACTCAACACAAGAAGAGATCGACAA
AATTCGTGCGAAAGTGCTTGAGATGAACGAGTTCATCAAGGCACAAGCGATGTACTACAAAGCGCAAGGTTACAA
CATCACGTTGTTTGATACTCACGCCTTGTTCGAGACGCTAACTTCTGCGCCAGAAGAGCACGGTTTCGTGAACGCG
AGCGATCCTTGTTTGGACATCAACCGCTCATCGTCTGTCGATTACATGTACACCCACGCATTGCGCTCTGAGTGTGC
AGCGTCTGGTGCTGAGAAGTTTGTGTTCTGGGATGTCACGCATCCAACAACAGCAACTCACCGCTATGTTGCAGAG
AAAATGCTAGAAAGTAGCAACAACTTAGCCGAGTACCGTTTCTAA

>10-4293
ATGATGAAAAAAACAATCACACTATTAACTGCATTACTCCCGCTTGCTTCTGCAGTTGCCGAAGAGCCAACCTTAT
CACCAGAAATGGTTTCAGCGTCTGAAGTGATCAGCACGCAAGAAAACCAAACCTATACCTATGTTCGCTGTTGGTA
TCGCACCAGCTACTCGAAAGATGATCCGGCGACCGATTGGGAATGGGCAAAAAACGAAGATGGTAGCTACTTCAC
CATTGACGGCTACTGGTGGAGCTCCGTTTCACTTAAAAACATGTTCTACACCAACACGTCGCAAAACGTTATCCGT
CAGCGTTGTGAAGCAACATTAGATTTGGCGAACGAGAACGCAGACATTACGTTCTTCGCCGCTGACAATCGCTTCT
CATACAACCACACGATCTGGAGCAACGACGCAGCAATGCAGCCAGATCAAATCAACAAAGTGGTTGCACTCGGTG
ACAGCTTGTCTGATACAGGCAACATCTTTAACGCATCACAATGGCGCTTCCCTAACCCGAACAGCTGGTTCTTAGG
TCACTTCTCCAACGGTTTTGTGTGGACAGAATACATTGCCAAAGCGAAGAACCTTCCGCTCTACAACTGGGCAGTT
GGCGGCGCGGCTGGTGAGAACCAATACATCGCGCTAACAGGGGTTGGTGATCAAGTTTCTTCGTACTTAACCTACG
CAAAACTGGCGAAGAACTACAAACCAGCAAACACCTTGTTTACGCTTGAGTTTGGTTTGAATGACTTCATGAACTA
CAACCGTGGCGTTCCAGAAGTGAAAGCAGATTATGCAGAAGCACTGATTCGTTTGACGGACGCAGGTGCGAAGAA
CTTCATGTTGATGACACTGCCAGACGCGACGAAAGCGCCTCAGTTTAAGTACTCAACACAAGAAGAGATCGACAA
AATTCGTGCGAAAGTGCTTGAGATGAACGAGTTCATCAAGGCACAAGCGATGTACTACAAAGCGCAAGGTTACAA
CATCACGTTGTTTGATACTCACGCCTTGTTCGAGACGCTAACTTCTGCGCCAGAAGAGCACGGTTTCGTGAACGCG
AGCGATCCTTGTTTGGACATCAACCGCTCATCGTCTGTCGATTACATGTACACCCACGCATTGCGCTCTGAGTGTGC
AGCGTCTGGTGCTGAGAAGTTTGTGTTCTGGGATGTCACGCATCCAACAACAGCAACTCACCGCTATGTTGCAGAG
AAAATGCTAGAAAGTAGCAACAACTTAGCCGAGTACCGTTTCTAA

>10-4288
ATGATGAAAAAAACAATCACACTATTAACTGCATTACTCCCGCTTGCTTCTGCAGTTGCCGAAGAGCCAACCTTAT
CACCAGAAATGGTTTCAGCGTCTGAAGTGATCAGCACGCAAGAAAACCAAACCTATACCTATGTTCGCTGTTGGTA
TCGCACCAGCTACTCGAAAGATGATCCGGCGACCGATTGGGAATGGGCAAAAAACGAAGATGGTAGCTACTTCAC
CATTGACGGCTACTGGTGGAGCTCCGTTTCACTTAAAAACATGTTCTACACCAACACGTCGCAAAACGTTATCCGT
CAGCGTTGTGAAGCAACATTAGATTTGGCGAACGAGAACGCAGACATTACGTTCTTCGCCGCTGACAATCGCTTCT
CATACAACCACACGATCTGGAGCAACGACGCAGCAATGCAGCCAGATCAAATCAACAAAGTGGTTGCACTCGGTG
ACAGCTTGTCTGATACAGGCAACATCTTTAACGCATCACAATGGCGCTTCCCTAACCCGAACAGCTGGTTCTTAGG
TCACTTCTCCAACGGTTTTGTGTGGACAGAATACATTGCCAAAGCGAAGAACCTTCCGCTCTACAACTGGGCAGTT
GGCGGCGCGGCTGGTGAGAACCAATACATCGCGCTAACAGGGGTTGGTGATCAAGTTTCTTCGTACTTAACCTACG
CAAAACTGGCGAAGAACTACAAACCAGCAAACACCTTGTTTACGCTTGAGTTTGGTTTGAATGACTTCATGAACTA
CAACCGTGGCGTTCCAGAAGTGAAAGCAGATTATGCAGAAGCACTGATTCGTTTGACGGACGCAGGTGCGAAGAA
CTTCATGTTGATGACACTGCCAGACGCGACGAAAGCGCCTCAGTTTAAGTACTCAACACAAGAAGAGATCGACAA
AATTCGTGCGAAAGTGCTTGAGATGAACGAGTTCATCAAGGCACAAGCGATGTACTACAAAGCGCAAGGTTACAA

CATCACGTTGTTTGATACTCACGCCTTGTTCGAGACGCTAACTTCTGCGCCAGAAGAGCACGGTTTCGTGAACGCG
AGCGATCCTTGTTTGGACATCAACCGCTCATCGTCTGTCGATTACATGTACACCCACGCATTGCGCTCTGAGTGTGC
AGCGTCTGGTGCTGAGAAGTTTGTGTTCTGGGATGTCACGCATCCAACAACAGCAACTCACCGCTATGTTGCAGAG
AAAATGCTAGAAAGTAGCAACAACTTAGCCGAGTACCGTTTCTAA

>10-4247
ATGATGAAAAAAACAATCACACTATTAACTGCATTACTCCCGCTTGCTTCTGCAGTTGCCGAAGAGCCAACCTTAT
CACCAGAAATGGTTTCAGCGTCTGAAGTGATCAGCACGCAAGAAAACCAAACCTATACCTATGTTCGCTGTTGGTA
TCGCACCAGCTACTCGAAAGATGATCCGGCGACCGATTGGGAATGGGCAAAAAACGAAGATGGTAGCTACTTCAC
CATTGACGGCTACTGGTGGAGCTCCGTTTCACTTAAAAACATGTTCTACACCAACACGTCGCAAAACGTTATCCGT
CAGCGTTGTGAAGCAACATTAGATTTGGCGAACGAGAACGCAGACATTACGTTCTTCGCCGCTGACAATCGCTTCT
CATACAACCACACGATCTGGAGCAACGACGCAGCAATGCAGCCAGATCAAATCAACAAAGTGGTTGCACTCGGTG
ACAGCTTGTCTGATACAGGCAACATCTTTAACGCATCACAATGGCGCTTCCCTAACCCGAACAGCTGGTTCTTAGG
TCACTTCTCCAACGGTTTTGTGTGGACAGAATACATTGCCAAAGCGAAGAACCTTCCGCTCTACAACTGGGCAGTT
GGCGGCGCGGCTGGTGAGAACCAATACATCGCGCTAACAGGGGTTGGTGATCAAGTTTCTTCGTACTTAACCTACG
CAAAACTGGCGAAGAACTACAAACCAGCAAACACCTTGTTTACGCTTGAGTTTGGTTTGAATGACTTCATGAACTA
CAACCGTGGCGTTCCAGAAGTGAAAGCAGATTATGCAGAAGCACTGATTCGTTTGACGGACGCAGGTGCGAAGAA
CTTCATGTTGATGACACTGCCAGACGCGACGAAAGCGCCTCAGTTTAAGTACTCAACACAAGAAGAGATCGACAA
AATTCGTGCGAAAGTGCTTGAGATGAACGAGTTCATCAAGGCACAAGCGATGTACTACAAAGCGCAAGGTTACAA
CATCACGTTGTTTGATACTCACGCCTTGTTCGAGACGCTAACTTCTGCGCCAGAAGAGCACGGTTTCGTGAACGCG
AGCGATCCTTGTTTGGACATCAACCGCTCATCGTCTGTCGATTACATGTACACCCACGCATTGCGCTCTGAGTGTGC
AGCGTCTGGTGCTGAGAAGTTTGTGTTCTGGGATGTCACGCATCCAACAACAGCAACTCACCGCTATGTTGCAGAG
AAAATGCTAGAAAGTAGCAACAACTTAGCCGAGTACCGTTTCTAA

>10-4248
ATGATGAAAAAAACAATCACACTATTAACTGCATTACTCCCGCTTGCTTCTGCAGTTGCCGAAGAGCCAA
CCTTATCACCAGAAATGGTTTCAGCGTCTGAAGTGATCAGCACGCAAGAAAACCAAACCTATACCTATGT
TCGCTGTTGGTATCGCACCAGCTACTCGAAAGATGATCCGGCGACCGATTGGGAATGGGCAAAAAACGAA
GATGGTAGCTACTTCACCATTGACGGCTACTGGTGGAGCTCCGTTTCACTTAAAAACATGTTCTACACCA
ACACGTCGCAAAACGTTATCCGTCAGCGTTGTGAAGCAACATTAGATTTGGCGAACGAGAACGCAGACAT
TACGTTCTTCGCCGCTGACAATCGCTTCTCATACAACCACACGATCTGGAGCAACGACGCAGCAATGCAG
CCAGATCAAATCAACAAAGTGGTTGCACTCGGTGACAGCTTGTCTGATACAGGCAACATCTTTAACGCAT
CACAATGGCGCTTCCCTAACCCGAACAGCTGGTTCTTAGGTCACTTCTCCAACGGTTTTGTGTGGACAGA
ATACATTGCCAAAGCGAAGAACCTTCCGCTCTACAACTGGGCAGTTGGCGGCGCGGCTGGTGAGAACCAA
TACATCGCGCTAACAGGGGTTGGTGATCAAGTTTCTTCGTACTTAACCTACGCAAAACTGGCGAAGAACT
ACAAACCAGCAAACACCTTGTTTACGCTTGAGTTTGGTTTGAATGACTTCATGAACTACAACCGTGGCGT
TCCAGAAGTGAAAGCAGATTATGCAGAAGCACTGATTCGTTTGACGGACGCAGGTGCGAAGAACTTCATG
TTGATGACACTGCCAGACGCGACGAAAGCGCCTCAGTTTAAGTACTCAACACAAGAAGAGATCGACAAAA
TTCGTGCGAAAGTGCTTGAGATGAACGAGTTCATCAAGGCACAAGCGATGTACTACAAAGCGCAAGGTTA
CAACATCACGTTGTTTGATACTCACGCCTTGTTCGAGACGCTAACTTCTGCGCCAGAAGAGCACGGTTTC
GTGAACGCGAGCGATCCTTGTTTGGACATCAACCGCTCATCGTCTGTCGATTACATGTACACCCACGCAT
TGCGCTCTGAGTGTGCAGCGTCTGGTGCTGAGAAGTTTGTGTTCTGGGATGTCACGCATCCAACAACAGC
AACTCACCGCTATGTTGCAGAGAAAATGCTAGAAAGTAGCAACAACTTAGCCGAGTACCGTTTCTAA

>10-4246
ATGATGAAAAAAACAATCACACTATTAACTGCATTACTCCCGCTTGCTTCTGCAGTTGCCGAAGAGCCAACCTTAT
CACCAGAAATGGTTTCAGCGTCTGAAGTGATCAGCACGCAAGAAAACCAAACCTATACCTATGTTCGCTGTTGGTA
TCGCACCAGCTACTCGAAAGATGATCCGGCGACCGATTGGGAATGGGCAAAAAACGAAGATGGTAGCTACTTCAC
CATTGACGGCTACTGGTGGAGCTCCGTTTCACTTAAAAACATGTTCTACACCAACACGTCGCAAAACGTTATCCGT
CAGCGTTGTGAAGCAACATTAGATTTGGCGAACGAGAACGCAGACATTACGTTCTTCGCCGCTGACAATCGCTTCT
CATACAACCACACGATCTGGAGCAACGACGCAGCAATGCAGCCAGATCAAATCAACAAAGTGGTTGCACTCGGTG

ACAGCTTGTCTGATACAGGCAACATCTTTAACGCATCACAATGGCGCTTCCCTAACCCGAACAGCTGGTTCTTAGG
TCACTTCTCCAACGGTTTTGTGTGGACAGAATACATTGCCAAAGCGAAGAACCTTCCGCTCTACAACTGGGCAGTT
GGCGGCGCGGCTGGTGAGAACCAATACATCGCGCTAACAGGGGTTGGTGATCAAGTTTCTTCGTACTTAACCTACG
CAAAACTGGCGAAGAACTACAAACCAGCAAACACCTTGTTTACGCTTGAGTTTGGTTTGAATGACTTCATGAACTA
CAACCGTGGCGTTCCAGAAGTGAAAGCAGATTATGCAGAAGCACTGATTCGTTTGACGGACGCAGGTGCGAAGAA
CTTCATGTTGATGACACTGCCAGACGCGACGAAAGCGCCTCAGTTTAAGTACTCAACACAAGAAGAGATCGACAA
AATTCGTGCGAAAGTGCTTGAGATGAACGAGTTCATCAAGGCACAAGCGATGTACTACAAAGCGCAAGGTTACAA
CATCACGTTGTTTGATACTCACGCCTTGTTCGAGACGCTAACTTCTGCGCCAGAAGAGCACGGTTTCGTGAACGCG
AGCGATCCTTGTTTGGACATCAACCGCTCATCGTCTGTCGATTACATGTACACCCACGCATTGCGCTCTGAGTGTGC
AGCGTCTGGTGCTGAGAAGTTTGTGTTCTGGGATGTCACGCATCCAACAACAGCAACTCACCGCTATGTTGCAGAG
AAAATGCTAGAAAGTAGCAACAACTTAGCCGAGTACCGTTTCTAA

>10-4245
ATGATGAAAAAAACAATCACACTATTAACTGCATTACTCCCGCTTGCTTCTGCAGTTGCCGAAGAGCCAACCTTAT
CACCAGAAATGGTTTCAGCGTCTGAAGTGATCAGCACGCAAGAAAACCAAACCTATACCTATGTTCGCTGTTGGTA
TCGCACCAGCTACTCGAAAGATGATCCGGCGACCGATTGGGAATGGGCAAAAAACGAAGATGGTAGCTACTTCAC
CATTGACGGCTACTGGTGGAGCTCCGTTTCACTTAAAAACATGTTCTACACCAACACGTCGCAAAACGTTATCCGT
CAGCGTTGTGAAGCAACATTAGATTTGGCGAACGAGAACGCAGACATTACGTTCTTCGCCGCTGACAATCGCTTCT
CATACAACCACACGATCTGGAGCAACGACGCAGCAATGCAGCCAGATCAAATCAACAAAGTGGTTGCACTCGGTG
ACAGCTTGTCTGATACAGGCAACATCTTTAACGCATCACAATGGCGCTTCCCTAACCCGAACAGCTGGTTCTTAGG
TCACTTCTCCAACGGTTTTGTGTGGACAGAATACATTGCCAAAGCGAAGAACCTTCCGCTCTACAACTGGGCAGTT
GGCGGCGCGGCTGGTGAGAACCAATACATCGCGCTAACAGGGGTTGGTGATCAAGTTTCTTCGTACTTAACCTACG
CAAAACTGGCGAAGAACTACAAACCAGCAAACACCTTGTTTACGCTTGAGTTTGGTTTGAATGACTTCATGAACTA
CAACCGTGGCGTTCCAGAAGTGAAAGCAGATTATGCAGAAGCACTGATTCGTTTGACGGACGCAGGTGCGAAGAA
CTTCATGTTGATGACACTGCCAGACGCGACGAAAGCGCCTCAGTTTAAGTACTCAACACAAGAAGAGATCGACAA
AATTCGTGCGAAAGTGCTTGAGATGAACGAGTTCATCAAGGCACAAGCGATGTACTACAAAGCGCAAGGTTACAA
CATCACGTTGTTTGATACTCACGCCTTGTTCGAGACGCTAACTTCTGCGCCAGAAGAGCACGGTTTCGTGAACGCG
AGCGATCCTTGTTTGGACATCAACCGCTCATCGTCTGTCGATTACATGTACACCCACGCATTGCGCTCTGAGTGTGC
AGCGTCTGGTGCTGAGAAGTTTGTGTTCTGGGATGTCACGCATCCAACAACAGCAACTCACCGCTATGTTGCAGAG
AAAATGCTAGAAAGTAGCAACAACTTAGCCGAGTACCGTTTCTAA

>10-4274
ATGATGAAAAAAACAATCACACTATTAACTGCATTACTCCCGCTTGCTTCTGCAGTTGCCGAAGAGCCAACCTTAT
CACCAGAAATGGTTTCAGCGTCTGAAGTGATCAGCACGCAAGAAAACCAAACCTATACCTATGTTCGCTGTTGGTA
TCGCACCAGCTACTCGAAAGATGATCCGGCGACCGATTGGGAATGGGCAAAAAACGAAGATGGTAGCTACTTCAC
CATTGACGGCTACTGGTGGAGCTCCGTTTCACTTAAAAACATGTTCTACACCAACACGTCGCAAAACGTTATCCGT
CAGCGTTGTGAAGCAACATTAGATTTGGCGAACGAGAACGCAGACATTACGTTCTTCGCCGCTGACAATCGCTTCT
CATACAACCACACGATCTGGAGCAACGACGCAGCAATGCAGCCAGATCAAATCAACAAAGTGGTTGCACTCGGTG
ACAGCTTGTCTGATACAGGCAACATCTTTAACGCATCACAATGGCGCTTCCCTAACCCGAACAGCTGGTTCTTAGG
TCACTTCTCCAACGGTTTTGTGTGGACAGAATACATTGCCAAAGCGAAGAACCTTCCGCTCTACAACTGGGCAGTT
GGCGGCGCGGCTGGTGAGAACCAATACATCGCGCTAACAGGGGTTGGTGATCAAGTTTCTTCGTACTTAACCTACG
CAAAACTGGCGAAGAACTACAAACCAGCAAACACCTTGTTTACGCTTGAGTTTGGTTTGAATGACTTCATGAACTA
CAACCGTGGCGTTCCAGAAGTGAAAGCAGATTATGCAGAAGCACTGATTCGTTTGACGGACGCAGGTGCGAAGAA
CTTCATGTTGATGACACTGCCAGACGCGACGAAAGCGCCTCAGTTTAAGTACTCAACACAAGAAGAGATCGACAA
AATTCGTGCGAAAGTGCTTGAGATGAACGAGTTCATCAAGGCACAAGCGATGTACTACAAAGCGCAAGGTTACAA
CATCACGTTGTTTGATACTCACGCCTTGTTCGAGACGCTAACTTCTGCGCCAGAAGAGCACGGTTTCGTGAACGCG
AGCGATCCTTGTTTGGACATCAACCGCTCATCGTCTGTCGATTACATGTACACCCACGCATTGCGCTCTGAGTGTGC
AGCGTCTGGTGCTGAGAAGTTTGTGTTCTGGGATGTCACGCATCCAACAACAGCAACTCACCGCTATGTTGCAGAG
AAAATGCTAGAAAGTAGCAACAACTTAGCCGAGTACCGTTTCTAA

>10-4242

ATGATGAAAAAAACAATCACACTATTAACTGCATTACTCCCGCTTGCTTCTGCAGTTGCCGAAGAGCCAACCTTAT
CACCAGAAATGGTTTCAGCGTCTGAAGTGATCAGCACGCAAGAAAACCAAACCTATACCTATGTTCGCTGTTGGTA
TCGCACCAGCTACTCGAAAGATGATCCGGCGACCGATTGGGAATGGGCAAAAAACGAAGATGGTAGCTACTTCAC
CATTGACGGCTACTGGTGGAGCTCCGTTTCACTTAAAAACATGTTCTACACCAACACGTCGCAAAACGTTATCCGT
CAGCGTTGTGAAGCAACATTAGATTTGGCGAACGAGAACGCAGACATTACGTTCTTCGCCGCTGACAATCGCTTCT
CATACAACCACACGATCTGGAGCAACGACGCAGCAATGCAGCCAGATCAAATCAACAAAGTGGTTGCACTCGGTG
ACAGCTTGTCTGATACAGGCAACATCTTTAACGCATCACAATGGCGCTTCCCTAACCCGAACAGCTGGTTCTTAGG
TCACTTCTCCAACGGTTTTGTGTGGACAGAATACATTGCCAAAGCGAAGAACCTTCCGCTCTACAACTGGGCAGTT
GGCGGCGCGGCTGGTGAGAACCAATACATCGCGCTAACAGGGGTTGGTGATCAAGTTTCTTCGTACTTAACCTACG
CAAAACTGGCGAAGAACTACAAACCAGCAAACACCTTGTTTACGCTTGAGTTTGGTTTGAATGACTTCATGAACTA
CAACCGTGGCGTTCCAGAAGTGAAAGCAGATTATGCAGAAGCACTGATTCGTTTGACGGACGCAGGTGCGAAGAA
CTTCATGTTGATGACACTGCCAGACGCGACGAAAGCGCCTCAGTTTAAGTACTCAACACAAGAAGAGATCGACAA
AATTCGTGCGAAAGTGCTTGAGATGAACGAGTTCATCAAGGCACAAGCGATGTACTACAAAGCGCAAGGTTACAA
CATCACGTTGTTTGATACTCACGCCTTGTTCGAGACGCTAACTTCTGCGCCAGAAGAGCACGGTTTCGTGAACGCG
AGCGATCCTTGTTTGGACATCAACCGCTCATCGTCTGTCGATTACATGTACACCCACGCATTGCGCTCTGAGTGTGC
AGCGTCTGGTGCTGAGAAGTTTGTGTTCTGGGATGTCACGCATCCAACAACAGCAACTCACCGCTATGTTGCAGAG
AAAATGCTAGAAAGTAGCAACAACTTAGCCGAGTACCGTTTCTAA

>09-3216
ATGATGAAAAAAACAATCACACTATTAACTGCATTACTCCCGCTTGCTTCTGCAGTTGCCGAAGAGCCAACCTTAT
CACCAGAAATGGTTTCAGCGTCTGAAGTGATCAGCACGCAAGAAAACCAAACCTATACCTATGTTCGCTGTTGGTA
TCGCACCAGCTACTCGAAAGATGATCCGGCGACCGATTGGGAATGGGCAAAAAACGAAGATGGTAGCTACTTCAC
CATTGACGGCTACTGGTGGAGCTCCGTTTCACTTAAAAACATGTTCTACACCAACACGTCGCAAAACGTTATCCGT
CAGCGTTGTGAAGCAACATTAGATTTGGCGAACGAGAACGCAGACATTACGTTCTTCGCCGCTGACAATCGCTTCT
CATACAACCACACGATCTGGAGCAACGACGCAGCAATGCAGCCAGATCAAATCAACAAAGTGGTTGCACTCGGTG
ACAGCTTGTCTGATACAGGCAACATCTTTAACGCATCACAATGGCGCTTCCCTAACCCGAACAGCTGGTTCTTAGG
TCACTTCTCCAACGGTTTTGTGTGGACAGAATACATTGCCAAAGCGAAGAACCTTCCGCTCTACAACTGGGCAGTT
GGCGGCGCGGCTGGTGAGAACCAATACATCGCGCTAACAGGGGTTGGTGATCAAGTTTCTTCGTACTTAACCTACG
CAAAACTGGCGAAGAACTACAAACCAGCAAACACCTTGTTTACGCTTGAGTTTGGTTTGAATGACTTCATGAACTA
CAACCGTGGCGTTCCAGAAGTGAAAGCAGATTATGCAGAAGCACTGATTCGTTTGACGGACGCAGGTGCGAAGAA
CTTCATGTTGATGACACTGCCAGACGCGACGAAAGCGCCTCAGTTTAAGTACTCAACACAAGAAGAGATCGACAA
AATTCGTGCGAAAGTGCTTGAGATGAACGAGTTCATCAAGGCACAAGCGATGTACTACAAAGCGCAAGGTTACAA
CATCACGTTGTTTGATACTCACGCCTTGTTCGAGACGCTAACTTCTGCGCCAGAAGAGCACGGTTTCGTGAACGCG
AGCGATCCTTGTTTGGACATCAACCGCTCATCGTCTGTCGATTACATGTACACCCACGCATTGCGCTCTGAGTGTGC
AGCGTCTGGTGCTGAGAAGTTTGTGTTCTGGGATGTCACGCATCCAACAACAGCAACTCACCGCTATGTTGCAGAG
AAAATGCTAGAAAGTAGCAACAACTTAGCCGAGTACCGTTTCTAA

>10-4241
ATGATGAAAAAAACAATCACACTATTAACTGCATTACTCCCGCTTGCTTCTGCAGTTGCCGAAGAGCCAACCTTAT
CACCAGAAATGGTTTCAGCGTCTGAAGTGATCAGCACGCAAGAAAACCAAACCTATACCTATGTTCGCTGTTGGTA
TCGCACCAGCTACTCGAAAGATGATCCGGCGACCGATTGGGAATGGGCAAAAAACGAAGATGGTAGCTACTTCAC
CATTGACGGCTACTGGTGGAGCTCCGTTTCACTTAAAAACATGTTCTACACCAACACGTCGCAAAACGTTATCCGT
CAGCGTTGTGAAGCAACATTAGATTTGGCGAACGAGAACGCAGACATTACGTTCTTCGCCGCTGACAATCGCTTCT
CATACAACCACACGATCTGGAGCAACGACGCAGCAATGCAGCCAGATCAAATCAACAAAGTGGTTGCACTCGGTG
ACAGCTTGTCTGATACAGGCAACATCTTTAACGCATCACAATGGCGCTTCCCTAACCCGAACAGCTGGTTCTTAGG
TCACTTCTCCAACGGTTTTGTGTGGACAGAATACATTGCCAAAGCGAAGAACCTTCCGCTCTACAACTGGGCAGTT
GGCGGCGCGGCTGGTGAGAACCAATACATCGCGCTAACAGGGGTTGGTGATCAAGTTTCTTCGTACTTAACCTACG
CAAAACTGGCGAAGAACTACAAACCAGCAAACACCTTGTTTACGCTTGAGTTTGGTTTGAATGACTTCATGAACTA
CAACCGTGGCGTTCCAGAAGTGAAAGCAGATTATGCAGAAGCACTGATTCGTTTGACGGACGCAGGTGCGAAGAA
CTTCATGTTGATGACACTGCCAGACGCGACGAAAGCGCCTCAGTTTAAGTACTCAACACAAGAAGAGATCGACAA
AATTCGTGCGAAAGTGCTTGAGATGAACGAGTTCATCAAGGCACAAGCGATGTACTACAAAGCGCAAGGTTACAA

CATCACGTTGTTTGATACTCACGCCTTGTTCGAGACGCTAACTTCTGCGCCAGAAGAGCACGGTTTCGTGAACGCG
AGCGATCCTTGTTTGGACATCAACCGCTCATCGTCTGTCGATTACATGTACACCCACGCATTGCGCTCTGAGTGTGC
AGCGTCTGGTGCTGAGAAGTTTGTGTTCTGGGATGTCACGCATCCAACAACAGCAACTCACCGCTATGTTGCAGAG
AAAATGCTAGAAAGTAGCAACAACTTAGCCGAGTACCGTTTCTAA

>04-1290
ATGATGAAAAAAACAATCACACTATTAACTGCATTACTCCCGCTTGCTTCTGCAGTTGCCGAAGAGCCAACCTTAT
CACCAGAAATGGTTTCAGCGTCTGAAGTGATCAGCACGCAAGAAAACCAAACCTATACCTATGTTCGCTGTTGGTA
TCGCACCAGCTACTCGAAAGATGATCCGGCGACCGATTGGGAATGGGCAAAAAACGAAGATGGTAGCTACTTCAC
CATTGACGGCTACTGGTGGAGCTCCGTTTCACTTAAAAACATGTTCTACACCAACACGTCGCAAAACGTTATCCGT
CAGCGTTGTGAAGCAACATTAGATTTGGCGAACGAGAACGCAGACATTACGTTCTTCGCCGCTGACAATCGCTTCT
CATACAACCACACGATCTGGAGCAACGACGCAGCAATGCAGCCAGATCAAATCAACAAAGTGGTTGCACTCGGTG
ACAGCTTGTCTGATACAGGCAACATCTTTAACGCATCACAATGGCGCTTCCCTAACCCGAACAGCTGGTTCTTAGG
TCACTTCTCCAACGGTTTTGTGTGGACAGAATACATTGCCAAAGCGAAGAACCTTCCGCTCTACAACTGGGCAGTT
GGCGGCGCGGCTGGTGAGAACCAATACATCGCGCTAACAGGGGTTGGTGATCAAGTTTCTTCGTACTTAACCTACG
CAAAACTGGCGAAGAACTACAAACCAGCAAACACCTTGTTTACGCTTGAGTTTGGTTTGAATGACTTCATGAACTA
CAACCGTGGCGTTCCAGAAGTGAAAGCAGATTATGCAGAAGCACTGATTCGTTTGACGGACGCAGGTGCGAAGAA
CTTCATGTTGATGACACTGCCAGACGCGACGAAAGCGCCTCAGTTTAAGTACTCAACACAAGAAGAGATCGACAA
AATTCGTGCGAAAGTGCTTGAGATGAACGAGTTCATCAAGGCACAAGCGATGTACTACAAAGCGCAAGGTTACAA
CATCACGTTGTTTGATACTCACGCCTTGTTCGAGACGCTAACTTCTGCGCCAGAAGAGCACGGTTTCGTGAACGCG
AGCGATCCTTGTTTGGACATCAACCGCTCATCGTCTGTCGATTACATGTACACCCACGCATTGCGCTCTGAGTGTGC
AGCGTCTGGTGCTGAGAAGTTTGTGTTCTGGGATGTCACGCATCCAACAACAGCAACTCACCGCTATGTTGCAGAG
AAAATGCTAGAAAGTAGCAACAACTTAGCCGAGTACCGTTTCTAA

>04-2549
ATGATGAAAAAAACAATCACACTATTAACTGCATTACTCCCGCTTGCTTCTGCAGTTGCCGAAGAGCCAACCTTAT
CACCAGAAATGGTTTCAGCGTCTGAAGTGATCAGCACGCAAGAAAACCAAACCTATACCTATGTTCGCTGTTGGTA
TCGCACCAGCTACTCGAAAGATGATCCAGCGACCGATTGGGAATGGGCAAAAAACGAAGATGGTAGCTACTTCAC
CATTGACGGCTACTGGTGGAGCTCCGTTTCATTTAAAAACATGTTCTACACCAACACGTCGCAAAACGTTATCCGT
CAGCGTTGTGAAGCAACATTAGATTTGGCGAACGAGAACGCAGACATTACGTTCTTCGCCGCTGACAATCGCTTCT
CATACAACCACACGATCTGGAGCAACGACGCAGCAATGCAGCCAGATCAAATCAACAAAGTGGTTGCACTCGGTG
ACAGCTTGTCTGATACAGGCAACATCTTTAACGCATCACAATGGCGCTTCCCTAACCCGAACAGCTGGTTCTTAGG
TCACTTCTCCAACGGTTTTGTTTGGACAGAATACATTGCCAAAGCGAAGAACCTTCCGCTCTACAACTGGGCAGTT
GGCGGCGCGGCTGGTGAGAACCAATACATCGCGCTAACAGGGGTTGGTGAGCAAGTTTCTTCGTACTTAACCTACG
CAAAACTGGCGAAGAACTACAAACCAGCAAACACCTTGTTTACGCTTGAGTTTGGTTTGAATGACTTCATGAACTA
CAACCGTGGCGTTCCAGAAGTGAAAGCGGATTATGCAGAAGCACTGATTCGTTTGACGGACGCAGGTGCGAAGAA
CTTCATGTTGATGACACTGCCAGATGCGACGAAAGCGCCTCAGTTTAAGTACTCAACACAAGAAGAGATCGACAA
AATTCGTGCGAAAGTGCTTGAGATGAACGAGTTCATCAAGGCACAAGCGATGTACTACAAAGCGCAAGGTTACAA
CATCACGTTGTTTGATACTCACGCCTTGTTCGAGACGCTAACTTCTGCGCCAGAAGAGCACGGTTTCGTGAACGCG
AGCGATCCTTGTTTGGACATCAACCGCTCATCGTCTGTCGATTACATGTACACCCACGCATTGCGCTCTGAGTGTGC
AGCGTCTGGTGCTGAGAAGTTTGTGTTCTGGGATGTCACGCATCCAACAACAGCAACTCACCGCTATGTTGCAGAG
AAAATGCTAGAAAGTAGCAACAACTTAGCCGAGTACCGTTTCTAA

>SG176
ATGATGAAAAAAACAATCACACTATTAACTGCATTACTCCCGCTTGCTTCTGCAGTTGCCGAAGAGCCAA
CCTTATCACCAGAAATGGTTTCAGCGTCTGAAGTGATCAGCACGCAAGAAAACCAAACCTATACCTATGT
TCGCTGTTGGTATCGCACCAGCTACTCGAAAGATGATCCGGCGACCGATTGGGAATGGGCAAAAAACGAA
GATGGTAGCTACTTCACCATTGACGGCTACTGGTGGAGCTCCGTTTCATTTAAAAACATGTTCTACACCA
ACACGTCGCAAAACGTTATCCGTCAGCATTGTGAAGTAACATTAGATTTGGCGAACGAGAACGCAGACAT
TACGTTCTTCGCCGCTGACAATCGCTTCTCATACAACCACACGATCTGGAGCAACGACGCAGCAATGCAG
CCAGATCAAATCAACAAAGTGGTTGCACTCGGTGACAGCTTGTCTGATACAGGCAACATCTTTAACGCAT

CACAATGGCGCTTCCCTAACCCGAACAGCTGGTTCTTAGGTCACTTCTCCAACGGTTTTGTGTGGACAGA
ATACATTGCCAAAGCGAAGAACCTTCCGCTCTACAACTGGGCAGTTGGCGGCGCGGCTGGTGAGAACCAA
TACATCGCGCTAACAGGGGTTGGTGATCAAGTTTCTTCGTACTTAACCTACGCAAAACTGGCGAAGAACT
ACAAACCAGCAAACACCTTGTTTACGCTTGAGTTTGGTTTGAATGACTTCATGAACTACAACCGTGGCGT
TCCAGAAGTGAAAGCAGATTATGCAGAAGCACTGATTCGTTTGACGGACGCAGGTGCGAAGAACTTCATG
TTGATGACACTGCCAGACGCGACGAAAGCGCCTCAGTTTAAGTACTCAACACAAGAAGAGATCGACAAAA
TTCGTGCGAAAGTGCTTGAGATGAACGAGTTCATCAAGGCACAAGCGATGTACTACAAAGCGCAAGGTTA
CAACATCACGTTGTTTGATACTCACGCCTTGTTCGAGACGCTAACTTCTGCGCCAGAAGAGCACGGTTTC
GTGAACGCGAGTGATCCTTGTTTGGACATCAACCGCTCATCGTCTGTCGATTACATGTACACCCACGCAT
TGCGCTCTGAGTGTGCGGCGTCTGGTGCTGAGAAGTTTGTGTTCTGGGATGTCACGCACCCAACAACAGC
AACTCACCGCTATGTTGCAGAGAAAATGCTAGAAAGTAGCAACAACTTAGCCGAGTACCGTTTCTAA

>K1461
ATGATGAAAAAAACAATCACACTATTAACTGCATTACTCCCGCTTGCTTCTGCAGTTGCCGAAGAGCCAA
CCTTATCACCAGAAATGGTTTCAGCGTCTGAAGTGATCAGCACGCAAGAAAACCAAACCTATACCTATGT
TCGCTGTTGGTATCGCACCAGCTACTCGAAAGATGATCCGGCGACCGATTGGGAATGGGCAAAAAACGAA
GATGGTAGCTACTTCACCATTGACGGCTACTGGTGGAGCTCCGTTTCACTTAAAAACATGTTCTACACCA
ACACGTCGCAAAACGTTATCCGTCAGCGTTGTGAAGCAACATTAGATTTGGCGAACGAGAACGCAGACAT
TACGTTCTTCGCCGCTGACAATCGCTTCTCATACAACCACACGATCTGGAGCAACGACGCAGCAATGCAG
CCAGATCAAATCAACAAAGTGGTTGCACTCGGTGACAGCTTGTCTGATACAGGCAACATCTTTAACGCAT
CACAATGGCGCTTCCCTAACCCGAACAGCTGGTTCTTAGGTCACTTCTCCAACGGTTTTGTGTGGACAGA
ATACATTGCCAAAGCGAAGAACCTTCCGCTCTACAACTGGGCAGTTGGCGGCGCGGCTGGTGAGAACCAA
TACATCGCGCTAACAGGGGTTGGTGATCAAGTTTCTTCGTACTTAACCTACGCAAAACTGGCGAAGAACT
ACAAACCAGCAAACACCTTGTTTACGCTTGAGTTTGGTTTGAATGACTTCATGAACTACAACCGTGGCGT
TCCAGAAGTGAAAGCAGATTATGCAGAAGCACTGATTCGTTTGACGGACGCAGGTGCGAAGAACTTCATG
TTGATGACACTGCCAGACGCGACGAAAGCGCCTCAGTTTAAGTACTCAACACAAGAAGAGATCGACAAAA
TTCGTGCGAAAGTGCTTGAGATGAACGAGTTCATCAAGGCACAAGCGATGTACTACAAAGCGCAAGGTTA
CAACATCACGTTGTTTGATACTCACGCCTTGTTCGAGACGCTAACTTCTGCGCCAGAAGAGCACGGTTTC
GTGAACGCGAGCGATCCTTGTTTGGACATCAACCGCTCATCGTCTGTCGATTACATGTACACCCACGCAT
TGCGCTCTGAGTGTGCAGCGTCTGGTGCTGAGAAGTTTGTGTTCTGGGATGTCACGCATCCAACAACAGC
AACTCACCGCTATGTTGCAGAGAAAATGCTAGAAAGTAGCAACAACTTAGCCGAGTACCGTTTCTAA

>K1275
ATGATGAAAAAAACAATCACACTATTAACTGCATTACTCCCACTTGCTTCTGCAGTTGCCGAAGAGCCAACCTTAT
CACCAGAAATGGTTTCAGCGTCTGAAGTGATCAGCACGCAAGAAAACCAAACCTATACCTATGTTCGCTGTTGGTA
TCGTACCAGCTACTCGAAAGATGATCCGGCGACCGATTGGGAATGGGCAAAAAACGAAGATGGTAGCTACTTCAC
CATTGACGGCTACTGGTGGAGCTCCGTTTCATTTAAAAACATGTTCTACACCAACACGTCGCAAAACGTTATCCGT
CAGCGTTGTGAAGCAACATTAGATTTGGCGAACGAGAACGCAGACATTACGTTCTTCGCCGCTGACAATCGCTTCT
CATACAACCACACGATCTGGAGCAACGACGCAGCAATGCAGCCAGATCAAATCAACAAAGTGGTTGCACTCGGTG
ACAGCTTGTCTGATACAGGCAACATCTTTAACGCATCACAATGGCGCTTCCCGAACCCGAACAGCTGGTTCTTAGG
TCACTTCTCCAACGGTTTTGTGTGGACAGAATACATTGCCAAAGCGAAGAACCTTCCGCTCTACAACTGGGCAGTT
GGCGGCGCGGCTGGTGAGAACCAATACATCGCGCTAACAGGGGTTGGTGATCAAGTTTCTTCGTACTTAACCTACG
CAAAACTGGCGAAGAACTACAAACCAGCAAACACCTTGTTTACGCTTGAGTTTGGTTTGAATGACTTCATGAACTA
CAACCGTGGCGTTCCAGAAGTGAAAGCGGATTATGCAGAAGCACTGATTCGTTTGACGGACGCAGGTGCGAAGAA
CTTCATGTTGATGACACTGCCAGACGCGACGAAAGCGCCTCAGTTTAAGTACTCAACACAAGAAGAGATCGACAA
AATTAGTGCGAAAGTGCTTGAGATGAACGAGTTCATCAAGGCACAAGCGATGTACTACAAAGCGCAAGGTTACAA
CATCACGTTGTTTGATACTCACGCCTTGTTCGAGACGCTAACTTCTGCGCCAGAAGAGCACGGTTTCGTGAACGCG
AGCGATCCTTGTTTGGACATCAACCGCTCATCGTCTGTCGATTACATGTACACCCACGCATTGCGTTCTGAGTGTGC
AGCGTCTGGTGCTGAGAAGTTTGTGTTCTGGGATGTCACGCATCCAACAACAGCAACTCACCGCTATGTTGCAGAG
AAAATGCTAGAAAGTAGCAACAACTTAGCCGAGTACCGTTTCTAA

>AF91
ATGATGAAAAAAACAATCACACTATTAACTGCATTACTCCCGCTTGCTTCTGCAGTTGCCGAAGAGCCAA
CCTTATCACCAGAAATGGTTTCAGCGTCTGAAGTGATCAGCACGCAAGAAAACCAAACCTATACCTATGT
TCGCTGTTGGTATCGCACCAGCTACTCGAAAGATGACCCGGCGACCGATTGGGAATGGGCAAAAAACGAA
GATGGTAGCTACTTCACCATTGACGGCTACTGGTGGAGCTCCGTTTCATTTAAAAACATGTTCTACACCA
ACACGTCGCAAAACGTTATCCGTCAGCGTTGTGAAGCAACATTAGATTTGGCGAACGAGAACGCAGACAT
TACGTTCTTCGCCGCTGACAATCGCTTCTCATACAACCACACGATCTGGAGCAACGACGCAGCAATGCAG
CCAGATCAAATCAACAAAGTGGTTGCACTCGGTGACAGCTTGTCTGATACAGGCAACATCTTTAACGCAT
CACAATGGCGCTTCCCTAACCCGAACAGCTGGTTCTTAGGTCACTTCTCCAACGGTTTTGTTTGGACAGA
ATACATTGCCAAAGCGAAGAACCTTCCGCTCTACAACTGGGCAGTTGGCGGCGCGGCTGGTGAGAACCAA
TACATCGCGCTAACAGGGGTTGGTGAGCAAGTTTCTTCGTACTTAACCTACGCAAAACTGGCGAAGAACT
ACAAACCAGCAAACACCTTGTTTACGCTTGAGTTTGGTTTGAATGACTTCATGAACTACAACCGTGGCGT
TCCAGAAGTGAAAGCGGATTATGCAGAAGCACTGATTCGTTTGACGGACGCAGGTGCGAAGAACTTCATG
TTGATGACACTGCCAGATGCGACGAAAGCGCCTCAGTTTAAGTACTCAACACAAGAAGAGATCGACAAAA
TTCGTGCGAAAGTGCTTGAGATGAACGAGTTCATCAAGGCACAAGCGATGTACTACAAAGCGCAAGGTTA
CAACATCACGTTGTTTGATACTCACGCCTTGTTCGAGACGCTAACTTCTGCGCCAGAAGAGCACGGTTTC
GTGAACGCGAGCGATCCTTGTTTGGACATCAACCGCTCATCGTCTGTCGATTACATGTACACCCACGCAT
TGCGCTCTGAGTGTGCAGCGTCTGGTGCTGAGAAGTTTGTGTTCTGGGATGTCACGCATCCAACAACAGC
AACTCACCGCTATGTTGCAGAGAAAATGCTAGAAAGTAGCAACAACTTAGCCGAGTACCGTTTCTAA

>22702
ATGATGAAAAAAACAATCACACTATTAACTGCATTACTCCCGCTTGCTTCTGCAGTTGCCGAAGAGCCAACCTTAT
CACCAGAAATGGTTTCAGCGTCTGAAGTGATCAGCACGCAAGAAAACCAAACCTATACCTATGTTCGCTGTTGGTA
TCGCACCAGCTACTCGAAAGATGATCCGGCGACCGATTGGGAATGGGCAAAAAACGAAGATGGTAGCTACTTCAC
CATTGACGGCTACTGGTGGAGCTCCGTTTCATTTAAAAACATGTTCTACACCAACACGTCGCAAAACGTTATCCGT
CAGCGTTGTGAAGCAACATTAGATTTGGCGAACGAGAACGCAGACATTACGTTCTTCGCCGCTGACAATCGCTTCT
CATACAACCACACGATCTGGAGCAACGACGCAGCAATGCAGCCAGATCAAATCAACAAAGTGGTTGCACTCGGTG
ACAGCTTGTCTGATACAGGCAACATCTTTAACGCATCACAATGGCGCTTCCCTAACCCGAACAGCTGGTTCTTAGG
TCACTTCTCCAACGGTTTTGTTTGGACAGAATACATTGCCAAAGCGAAGAACCTTCCGCTCTACAACTGGGCAGTT
GGCGGCGCGGCTGGTGAGAACCAATACATCGCGCTAACAGGGGTTGGTGATCAAGTTTCTTCGTACTTAACCTACG
CAAAACTGGCGAAGAACTACAAACCAGCAAACACCTTGTTTACGCTTGAGTTTGGTTTGAATGACTTCATGAACTA
CAACCGTGGCGTTCCAGAAGTGAAAGCGGATTATGCAGAAGCACTGATTCGTTTGACGGACGCAGGTGCGAAGAA
CTTCATGTTGATGACACTGCCAGATGCGACGAAAGCGCCTCAGTTTAAGTACTCAACACAAGAAGAGATCGACAA
AATTCGTGCGAAAGTGCTTGAGATGAACGAGTTCATCAAGGCACAAGCGATGTACTACAAAGCGCAAGGTTACAA
CATCACGTTGTTTGATACTCACGCCTTGTTCGAGACGCTAACTTCTGCGCCAGAAGAGCACGGTTTCGTGAACGCG
AGCGATCCTTGTTTGGACATCAACCGCTCATCGTCTGTCGATTACATGTACACCCACGCATTGCGCTCTGAGTGTGC
GGCGTCTGGTGCTGAGAAGTTTGTGTTCTGGGATGTCACGCACCCAACAACAGCAACTCACCGCTATGTTGCAGAG
AAAATGCTAGAAAGTAGCAACAACTTAGCCGAGTACCGTTTCTAA

>J-C2-34
ATGATGAAAAAAACAATCACACTATTAACTGCATTACTCCCGCTTGCTTCTGCAGTTGCCGAAGAGCCAA
CCTTATCACCAGAAATGGTTTCAGCGTCTGAAGTGATCAGCACGCAAGAAAACCAAACCTATACCTATGT
TCGCTGTTGGTATCGCACCAGCTACTCGAAAGATGATCCGGCGACCGATTGGGAATGGGCAAAAAACGAA
GATGGTAGCTACTTCACCATTGACGGCTACTGGTGGAGCTCCGTTTCATTTAAAAACATGTTCTACACCA
ACACGTCGCAAAACGTTATCCGTCAGCGTTGTGAAGCAACATTAGATTTGGCGAACGAGAACGCAGACAT
TACGTTCTTCGCCGCTGACAATCGCTTCTCATACAACCACACGATCTGGAGCAACGACGCAGCAATGCAG
CCAGATCAAATCAACAAAGTGGTTGCACTCGGTGACAGCTTGTCTGATACAGGCAACATCTTTAACGCAT
CACAATGGCGCTTCCCTAACCCTAACAGCTGGTTCTTAGGTCACTTCTCCAACGGTTTTGTGTGGACAGA
ATACATTGCCAAAGCGAAGAACCTTCCGCTCTACAACTGGGCAGTTGGCGGCGCGGCTGGTGAGAACCAA
TACATCGCGCTAACAGGGGTTGGTGAGCAAGTTTCTTCGTACTTAACCTACGCAAAACTGGCGAAGAACT
ACAAACCAGCAAACACCTTGTTTACGCTTGAGTTTGGTTTGAATGACTTCATGAACTACAACCGTGGCGT

TCCAGAAGTGAAAGCGGACTATGCAGAAGCACTGATTCGTTTGACGGACGCAGGTGCGAAGAACTTCATG
TTGATGACACTGCCAGACGCGACGAAAGCGCCTCAGTTTAAGTACTCAACACAAGAAGAGATCGACAAAA
TTCGTGCGAAAGTGCTTGAGATGAACGAGTTCATCAAGGCACAAGCGATGTACTACAAAGCGCAAGGTTA
CAACATCACGTTGTTTGATACTCACGCCTTGTTCGAGACGCTAACTTCTGCGCCAGAAGAGCACGGTTTC
GTGAACGCGAGCGATCCTTGTTTGGACATCAACCGCTCATCGTCTGTCGACTACATGTACACCCACGCAT
TGCGCTCTGAGTGTGCGGCGTCTGGTGCTGAGAAGTTTGTATTCTGGGATGTCACGCACCCAACAACAGC
AACTCACCGCTATGTTGCAGAGAAAATGCTAGAAAGTAGCAACAACTTAGCCGAGTACCGTTTCTAA

>07-2965
ATGATGAAAAAAACAATCACACTATTAACTGCATTACTCCCGCTTGCTTCTGCAGTTGCCGAAGAGCCAACCTTAT
CACCAGAAATGGTTTCAGTGTCTGAAGTGATCAGCACGCAAGAAAACCAAACCTATACCTATGTTCGCTGTTGGTA
TCGCACCAGCTACTCGAAAGATGATCCGGCGACCGATTGGGAATGGGCAAAAAACGAAGATGGTAGCTACTTCAC
CATTGACGGCTACTGGTGGAGCTCCGTTTCATTTAAAAACATGTTCTACACCAACACGTCGCAAAACGTTATCCGT
CAGCGTTGTGAAGCAACATTAGATTTGGCGAACGAGAACGCAGACATTACGTTCTTCGCCGCTGACAATCGCTTCT
CATACAACCACACGATCTGGAGCAACGACGCAGCAATGCAGCCAGATCAAATCAACAAAGTGGTTGCACTCGGTG
ACAGCTTGTCTGATACAGGCAACATCTTTAACGCATCACAATGGCGCTTCCCTAACCCGAACAGCTGGTTCTTAGG
TCACTTCTCCAACGGTTTTGTGTGGACAGAATACATTGCCAAAGCGAAGAACCTTCCGCTCTACAACTGGGCAGTT
GGCGGCGCGGCTGGTGAGAACCAATACATCGCGCTAACAGGGGTTGGTGAGCAAGTTTCTTCGTACTTAACCTACG
CAAAACTGGCGAAGAACTACAAACCAGCAAACACCTTGTTTACGCTTGAGTTTGGTTTGAATGACTTCATGAACTA
CAACCGTGGCGTTCCAGAAGTGAAAGCGGATTATGCAGAAGCACTGATTCGTTTGACGGACGCAGGTGCGAAGAA
CTTCATGTTGATGACACTGCCAGATGCGACGAAAGCGCCTCAGTTTAAGTACTCAACACAAGAAGAGATCGACAA
AATTCGTGCGAAAGTGCTTGAGATGAACGAGTTCATCAAGGCACAAGCGATGTACTACAAAGCGCAAGGTTACAA
CATCACGTTGTTTGATACTCACGCCTTGTTCGAGACGCTAACTTCTGCGCCAGAAGAGCACGGTTTCGTGAACGCG
AGCGATCCTTGTTTGGACATCAACCGCTCATCGTCTGTCGATTACATGTACACCCACGCATTGCGCTCTGAGTGTGC
AGCGTCTGGTGCTGAGAAGTTTGTGTTCTGGGATGTCACGCATCCAACAACAGCAACTCACCGCTATGTTGCAGAG
AAAATGCTAGAAAGTAGCAACAACTTAGCCGAGTACCGTTTCTAA

>09-4435
ATGATGAAAAAAACAATCACACTATTAACTGCATTACTCCCGCTTGCTTCTGCAGTTGCCGAAGAGCCAA
CCTTATCACCAGAAATGGTTTCAGCGTCTGAAGTGATCAGCACGCAAGAAAACCAAACCTATACCTATGT
TCGCTGTTGGTATCGCACCAGCTACTCGAAAGATGATCCAGCGACCGATTGGGAATGGGCAAAAAACGAA
GATGGTAGCTACTTCACCATTGACGGCTACTGGTGGAGCTCCGTTTCATTTAAAAACATGTTCTACACCA
ACACGTCGCAAAACGTTATCCGTCAGCGTTGTGAAGCAACATTAGATTTGGCGAACGAGAACGCAGACAT
TACGTTCTTCGCCGCTGACAATCGCTTCTCATACAACCACACGATCTGGAGCAACGACGCAGCAATGCAG
CCAGATCAAATCAACAAAGTGGTTGCACTCGGTGACAGCTTGTCTGATACAGGCAACATCTTTAACGCAT
CACAATGGCGCTTCCCTAACCCGAACAGCTGGTTCTTAGGTCACTTCTCCAACGGTTTTGTTTGGACAGA
ATACATTGCCAAAGCGAAGAACCTTCCGCTCTACAACTGGGCAGTTGGCGGCGCGGCTGGTGAGAACCAA
TACATCGCGCTAACAGGGGTTGGTGAGCAAGTTTCTTCGTACTTAACCTACGCAAAACTGGCGAAGAACT
ACAAACCAGCAAACACCTTGTTTACGCTTGAGTTTGGTTTGAATGACTTCATGAACTACAACCGTGGCGT
TCCAGAAGTGAAAGCGGATTATGCAGAAGCACTGATTCGTTTGACGGACGCAGGTGCGAAGAACTTCATG
TTGATGACACTGCCAGATGCGACGAAAGCGCCTCAGTTTAAGTACTCAACACAAGAAGAGATCGACAAAA
TTCGTGCGAAAGTGCTTGAGATGAACGAGTTCATCAAGGCACAAGCGATGTACTACAAAGCGCAAGGTTA
CAACATCACGTTGTTTGATACTCACGCCTTGTTCGAGACGCTAACTTCTGCGCCAGAAGAGCACGGTTTC
GTGAACGCGAGCGATCCTTGTTTGGACATCAACCGCTCATCGTCTGTCGATTACATGTACACCCACGCAT
TGCGCTCTGAGTGTGCAGCGTCTGGTGCTGAGAAGTTTGTGTTCTGGGATGTCACGCATCCAACAACAGC
AACTCACCGCTATGTTGCAGAGAAAATGCTAGAAAGTAGCAACAACTTAGCCGAGTACCGTTTCTAA

>09-4681
ATGATGAAAAAAACAATCACACTATTAACTGCATTACTCCCGCTTGCTTCTGCAGTTGCCGAAGAGCCAACCTTAT
CACCAGAAATGGTTTCAGCGTCTGAAGTGATCAGCACGCAAGAAAACCAAACCTATACCTATGTTCGCTGTTGGTA

TCGCACCAGCTACTCGAAAGATGATCCGGCGACCGATTGGGAATGGGCAAAAAACGAAGATGGTAGCTACTTCAC
CATTGACGGCTACTGGTGGAGCTCCGTTTCATTTAAAAACATGTTCTACACCAACACGTCGCAAAACGTTATCCGT
CAGCGTTGTGAAGCAACATTAGATTTGGCGAACGAGAACGCAGACATTACGTTCTTCGCCGCTGACAATCGCTTCT
CATACAACCACACGATCTGGAGCAACGACGCAGCAATGCAGCCAGATCAAATCAACAAAGTGGTTGCACTCGGTG
ACAGCTTGTCTGATACAGGCAACATCTTTAACGCATCACAATGGCGCTTCCCTAACCCGAACAGCTGGTTCTTAGG
TCACTTCTCGAACGGTTTTGTGTGGACAGAATACATTGCCAAAGCGAAGAACCTTCCGCTCTACAACTGGGCAGTT
GGCGGCGCGGCTGGTGAGAACCAATACATCGCGCTAACAGGGGTTGGTGATCAAGTTTCTTCGTACTTAACCTACG
CAAAACTGGCGAAGAACTACAAACCAGCAAACACCTTGTTTACGCTTGAGTTTGGTTTGAATGACTTCATGAACTA
CAACCGTGGCGTTCCAGAAGTGAAAGCAGATTATGCAGAAGCACTGATTCGTTTGACGGACGCAGGTGCGAAGAA
CTTCATGTTGATGACATTGCCAGACGCGACGAAAGCGCCTCAGTTTAAGTACTCAACACAAGAAGAGATCGACAA
AATTCGTGCGAAAGTGCTTGAGATGAACGAGTTCATCAAGGCACAAGCGATGTACTACAAAGCGCAAGGTTACAA
CATCACGTTGTTTGATACTCACGCCTTGTTCGAGACGCTAACTTCTGCGCCAGAAGAGCACGGTTTCGTGAACGCG
AGTGATCCTTGTTTGGACATCAACCGCTCATCGTCTGTCGATTACATGTACACCCACGCATTGCGCTCTGAGTGTGC
GGCGTCTGGTGCTGAGAAATTTGTGTTCTGGGATGTCACGCACCCAACAACAGCAACTCACCGCTATGTTGCAGAG
AAAATGCTAGAAAGTAGCAACAACTTAGCCGAGTACCGTTTCTAA

>09-4664
ATGATGAAAAAAACAATCACACTATTAACTGCATTACTCCCGCTTGCTTCTGCAGTTGCCGAAGAGCCAACCTTAT
CACCAGAAATGGTTTCAGCGTCTGAAGTGATCAGCACGCAAGAAAACCAAACCTATACCTATGTTCGCTGTTGGTA
TCGCACCAGCTACTCGAAAGATGATCCAGCGACCGATTGGGAATGGGCAAAAAACGAAGATGGTAGCTACTTCAC
CATTGACGGCTACTGGTGGAGCTCCGTTTCATTTAAAAACATGTTCTACACCAACACGTCGCAAAACGTTATCCGT
CAGCGTTGTGAAGCAACATTAGATTTGGCGAACGAGAACGCAGACATTACGTTCTTCGCCGCTGACAATCGCTTCT
CATACAACCACACGATCTGGAGCAACGACGCAGCAATGCAGCCAGATCAAATCAACAAAGTGGTTGCACTCGGTG
ACAGCTTGTCTGATACAGGCAACATCTTTAACGCATCACAATGGCGCTTCCCTAACCCGAACAGCTGGTTCTTAGG
TCACTTCTCCAACGGTTTTGTTTGGACAGAATACATTGCCAAAGCGAAGAACCTTCCGCTCTACAACTGGGCAGTT
GGCGGCGCGGCTGGTGAGAACCAATACATCGCGCTAACAGGGGTTGGTGAGCAAGTTTCTTCGTACTTAACCTACG
CAAAACTGGCGAAGAACTACAAACCAGCAAACACCTTGTTTACGCTTGAGTTTGGTTTGAATGACTTCATGAACTA
CAACCGTGGCGTTCCAGAAGTGAAAGCGGATTATGCAGAAGCACTGATTCGTTTGACGGACGCAGGTGCGAAGAA
CTTCATGTTGATGACACTGCCAGATGCGACGAAAGCGCCTCAGTTTAAGTACTCAACACAAGAAGAGATCGACAA
AATTCGTGCGAAAGTGCTTGAGATGAACGAGTTCATCAAGGCACAAGCGATGTACTACAAAGCGCAAGGTTACAA
CATCACGTTGTTTGATACTCACGCCTTGTTCGAGACGCTAACTTCTGCGCCAGAAGAGCACGGTTTCGTGAACGCG
AGCGATCCTTGTTTGGACATCAACCGCTCATCGTCTGTCGATTACATGTACACCCACGCATTGCGCTCTGAGTGTGC
AGCGTCTGGTGCTGAGAAGTTTGTGTTCTGGGATGTCACGCATCCAACAACAGCAACTCACCGCTATGTTGCAGAG
AAAATGCTAGAAAGTAGCAACAACTTAGCCGAGTACCGTTTCTAA

>07-1339
ATGATGAAAAAAACAATCACACTATTAACTGCATTACTCCCGCTTGCTTCTGCAGTTGCCGAAGAGCCAACCTTAT
CACCAGAAATGGTTTCAGCGTCTGAAGTGATCAGCACGCAAGAAAACCAAACCTATACCTATGTTCGCTGTTGGTA
TCGCACCAGCTACTCGAAAGATGATCCAGCGACCGATTGGGAATGGGCAAAAAACGAAGATGGTAGCTACTTCAC
CATTGACGGCTACTGGTGGAGCTCCGTTTCATTTAAAAACATGTTCTACACCAACACGTCGCAAAACGTTATCCGT
CAGCGTTGTGAAGCAACATTAGATTTGGCGAACGAGAACGCAGACATTACGTTCTTCGCCGCTGACAATCGCTTCT
CATACAACCACACGATCTGGAGCAACGACGCAGCAATGCAGCCAGATCAAATCAACAAAGTGGTTGCACTCGGTG
ACAGCTTGTCTGATACAGGCAACATCTTTAACGCATCACAATGGCGCTTCCCTAACCCGAACAGCTGGTTCTTAGG
TCACTTCTCCAACGGTTTTGTTTGGACAGAATACATTGCCAAAGCGAAGAACCTTCCGCTCTACAACTGGGCAGTT
GGCGGCGCGGCTGGTGAGAACCAATACATCGCGCTAACAGGGGTTGGTGAGCAAGTTTCTTCGTACTTAACCTACG
CAAAACTGGCGAAGAACTACAAACCAGCAAACACCTTGTTTACGCTTGAGTTTGGTTTGAATGACTTCATGAACTA
CAACCGTGGCGTTCCAGAAGTGAAAGCGGATTATGCAGAAGCACTGATTCGTTTGACGGACGCAGGTGCGAAGAA
CTTCATGTTGATGACACTGCCAGATGCGACGAAAGCGCCTCAGTTTAAGTACTCAACACAAGAAGAGATCGACAA
AATTCGTGCGAAAGTGCTTGAGATGAACGAGTTCATCAAGGCACAAGCGATGTACTACAAAGCGCAAGGTTACAA
CATCACGTTGTTTGATACTCACGCCTTGTTCGAGACGCTAACTTCTGCGCCAGAAGAGCACGGTTTCGTGAACGCG
AGCGATCCTTGTTTGGACATCAACCGCTCATCGTCTGTCGATTACATGTACACCCACGCATTGCGCTCTGAGTGTGC

AGCGTCTGGTGCTGAGAAGTTTGTGTTCTGGGATGTCACGCATCCAACAACAGCAACTCACCGCTATGTTGCAGAG
AAAATGCTAGAAAGTAGCAACAACTTAGCCGAGTACCGTTTCTAA

>09-4660
ATGATGAAAAAAACAATCACACTATTAACTGCATTACTCCCGCTTGCTTCTGCAGTTGCCGAAGAGCCAA
CCTTATCACCAGAAATGGTTTCAGCGTCTGAAGTGATCAGCACGCAAGAAAACCAAACCTATACCTATGT
TCGCTGTTGGTATCGCACCAGCTACTCGAAAGATGATCCGGCGACCGATTGGGAATGGGCAAAAAACGAA
GATGGTAGCTACTTCACCATTGACGGCTACTGGTGGAGCTCCGTTTCATTTAAAAACATGTTCTACACCA
ACACGTCGCAAAACGTTATCCGTCAGCGTTGTGAAGCCACATTAGATTTGGCGAACGAGAACGCAGACAT
TACGTTCTTCGCCGCTGACAATCGCTTCTCATACAACCACACGATCTGGAGCAACGACGCAGCAATGCAG
CCAGATCAAATCAACAAAGTGGTTGCACTCGGTGACAGCTTGTCTGATACAGGCAACATCTTTAACGCAT
CACAATGGCGCTTCCCTAACCCGAACAGCTGGTTCTTAGGTCACTTCTCCAACGGTTTTGTGTGGACAGA
ATACATTGCCAAAGCGAAGAACCTTCCGCTCTACAACTGGGCAGTTGGCGGCGCGGCTGGTGAGAACCAA
TACATCGCGCTAACAGGGGTTGGTGATCAAGTTTCTTCGTACTTAACCTACGCAAAACTGGCGAAGAACT
ACAAACCAGCAAACACCTTGTTTACGCTTGAGTTTGGTTTGAATGACTTCATGAACTACAACCGTGGCGT
TCCAGAAGTGAAAGCAGATTATGCAGAAGCACTGATTCGTTTGACGGACGCAGGTGCGAAGAACTTCATG
TTGATGACACTGCCAGACGCGACGAAAGCGCCTCAGTTTAAGTACTCAACACAAGAAGAGATCGACAAAA
TTCGTGCGAAAGTGCTTGAGATGAACGAGTTCATCAAGGCACAAGCGATGTACTACAAAGCGCAAGGTTA
CAACATCACGTTGTTTGATACTCACGCCTTGTTCGAGACGCTAACTTCTGCGCCCGAAAAGCACGGTTTC
GTGAACGCGAGTGATCCTTGTTTGGACATCAACCGCTCATCGTCTGTCGATTACATGTACACCCACGCAT
TGCGCTCTGAGTGTGCGGCGTCTGGTGCTGAGAAGTTTGTGTTCTGGGATGTCACGCACCCAACAACAGC
AACTCACCGCTATGTTGCAGAGAAAATGCTAGAAAGTAGCAACAACTTAGCCGAGTACCGTTTCTAA

>10-4243
ATGATGAAAAAAACAATCACACTATTAACTGCATTACTCCCGCTTGCTTCTGCAGTTGCCGAAGAGCCAACCTTAT
CACCAGAAATGGTTTCAGCGTCTGAAGTGATCAGCACGCAAGAAAACCAAACCTATACCTATGTTCGCTGTTGGTA
TCGCACCAGCTACTCGAAAGATGATCCGGCGACCGATTGGGAATGGGCAAAAAACGAAGATGGTAGCTACTTCAC
CATTGACGGCTACTGGTGGAGCTCCGTTTCATTTAAAAACATGTTCTACACCAACACGTCGCAAAACGTTATCCGT
CAGCGTTGTGAAGCAACATTAGATTTGGCGAACGAGAACGCAGACATTACGTTCTTCGCCGCTGACAATCGCTTCT
CATACAACCACACGATCTGGAGCAACGACGCAGCAATGCAGCCAGATCAAATCAACAAAGTGGTTGCACTCGGTG
ACAGCTTGTCTGATACAGGCAACATCTTTAACGCATCACAATGGCGCTTCCCTAACCCGAACAGCTGGTTCTTAGG
TCACTTCTCCAACGGTTTTGTGTGGACAGAATACATTGCCAAAGCGAAGAACCTTCCGCTCTACAACTGGGCAGTT
GGCGGCGCGGCTGGTGAGAACCAATACATCGCGCTAACAGGGGTTGGTGATCAAGTTTCTTCGTACTTAACCTACG
CAAAACTGGCGAAGAACTACAAACCAGCAAACACCTTGTTTACGCTTGAGTTTGGTTTGAATGACTTCATGAACTA
CAACCGTGGCGTTCCAGAAGTGAAAGCGGATTATGCAGAAGCACTGATTCGTTTGACGGACGCAGGTGCGAAGAA
CTTCATGTTGATGACACTGCCAGACGCGACGAAAGCGCCTCAGTTTAAGTACTCAACACAAGAAGAGATCGACAA
AATTCGTGCGAAAGTGCTTGAGATGAACGAGTTCATCAAGGCACAAGCGATGTACTACAAAGCGCAAGGTTACAA
CATCACGTTGTTTGATACTCACGCCTTGTTCGAGACGCTAACTTCTGCGCCAGAAGAGCACGGTTTCGTGAACGCG
AGCGATCCTTGTTTGGACATCAACCGCTCATCGTCTGTCGATTACATGTACACCCACGCATTGCGCTCTGAGTGTGC
AGCGTCTGGTGCTGAGAAGTTTGTGTTCTGGGATGTCACGCATCCAACAACTGCAACTCACCGCTATGTTGCAGAG
AAAATGCTAGAAAGTAGCAACAACTTAGCCGAGTACCGTTTCTAA

>09-4434
ATGATGAAAAAAACAATCACACTATTAACTGCATTACTCCCGCTTGCTTCTGCAGTTGCCGAAGAGCCAA
CCTTATCACCAGAAATGGTTTCAGCGTCTGAAGTGATCAGCACGCAAGAAAACCAAACCTATACCTATGT
TCGCTGTTGGTATCGCACCAGCTACTCGAAAGATGATCCGGCGACCGATTGGGAATGGGCAAAAAACGAA
GATGGTAGCTACTTCACCATTGACGGCTACTGGTGGAGCTCCGTTTCATTTAAAAACATGTTCTACACCA
ACACGTCGCAAAACGTTATCCGTCAGCGTTGTGAAGCCACATTAGATTTGGCGAACGAGAACGCAGACAT
TACGTTCTTCGCCGCTGACAATCGCTTCTCATACAACCACACGATCTGGAGCAACGACGCAGCAATGCAG
CCAGATCAAATCAACAAAGTGGTTGCACTCGGTGACAGCTTGTCTGATACAGGCAACATCTTTAACGCAT
CACAATGGCGCTTCCCTAACCCGAACAGCTGGTTCTTAGGTCACTTCTCCAACGGTTTTGTGTGGACAGA

ATACATTGCCAAAGCGAAGAACCTTCCGCTCTACAACTGGGCAGTTGGCGGCGCGGCTGGTGAGAACCAA
TACATCGCGCTAACAGGGGTTGGTGATCAAGTTTCTTCGTACTTAACCTACGCAAAACTGGCGAAGAACT
ACAAACCAGCAAACACCTTGTTTACGCTTGAGTTTGGTTTGAATGACTTCATGAACTACAACCGTGGCGT
TCCAGAAGTGAAAGCAGATTATGCAGAAGCACTGATTCGTTTGACGGACGCAGGTGCGAAGAACTTCATG
TTGATGACACTGCCAGACGCGACGAAAGCGCCTCAGTTTAAGTACTCAACACAAGAAGAGATCGACAAAA
TTCGTGCGAAAGTGCTTGAGATGAACGAGTTCATCAAGGCACAAGCGATGTACTACAAAGCGCAAGGTTA
CAACATCACGTTGTTTGATACTCACGCCTTGTTCGAGACGCTAACTTCTGCGCCCGAAAAGCACGGTTTC
GTGAACGCGAGTGATCCTTGTTTGGACATCAACCGCTCATCGTCTGTCGATTACATGTACACCCACGCAT
TGCGCTCTGAGTGTGCGGCGTCTGGTGCTGAGAAGTTTGTGTTCTGGGATGTCACGCACCCAACAACAGC
AACTCACCGCTATGTTGCAGAGAAAATGCTAGAAAGTAGCAACAACTTAGCCGAGTACCGTTTCTAA

>09-3218
ATGATGAAAAAAACAATCACACTATTAACTGCATTACTCCCGCTTGCTTCTGCAGTTGCCGAAGAGCCAA
CCTTATCACCAGAAATGGTTTCAGCGTCTGAAGTGATCAGCACGCAAGAAAACCAAACCTATACCTATGT
TCGCTGTTGGTATCGCACCAGCTACTCGAAAGATGATCCGGCGACCGATTGGGAATGGGCAAAAAACGAA
GATGGTAGCTACTTCACCATTGACGGCTACTGGTGGAGCTCCGTTTCATTTAAAAACATGTTCTACACCA
ACACGTCGCAAAACGTTATCCGTCAGCGTTGTGAAGCCACATTAGATTTGGCGAACGAGAACGCAGACAT
TACGTTCTTCGCCGCTGACAATCGCTTCTCATACAACCACACGATCTGGAGCAACGACGCAGCAATGCAG
CCAGATCAAATCAACAAAGTGGTTGCACTCGGTGACAGCTTGTCTGATACAGGCAACATCTTTAACGCAT
CACAATGGCGCTTCCCTAACCCGAACAGCTGGTTCTTAGGTCACTTCTCCAACGGTTTTGTGTGGACAGA
ATACATTGCCAAAGCGAAGAACCTTCCGCTCTACAACTGGGCAGTTGGCGGCGCGGCTGGTGAGAACCAA
TACATCGCGCTAACAGGGGTTGGTGATCAAGTTTCTTCGTACTTAACCTACGCAAAACTGGCGAAGAACT
ACAAACCAGCAAACACCTTGTTTACGCTTGAGTTTGGTTTGAATGACTTCATGAACTACAACCGTGGCGT
TCCAGAAGTGAAAGCAGATTATGCAGAAGCACTGATTCGTTTGACGGACGCAGGTGCGAAGAACTTCATG
TTGATGACACTGCCAGACGCGACGAAAGCGCCTCAGTTTAAGTACTCAACACAAGAAGAGATCGACAAAA
TTCGTGCGAAAGTGCTTGAGATGAACGAGTTCATCAAGGCACAAGCGATGTACTACAAAGCGCAAGGTTA
CAACATCACGTTGTTTGATACTCACGCCTTGTTCGAGACGCTAACTTCTGCGCCCGAAAAGCACGGTTTC
GTGAACGCGAGTGATCCTTGTTTGGACATCAACCGCTCATCGTCTGTCGATTACATGTACACCCACGCAT
TGCGCTCTGAGTGTGCGGCGTCTGGTGCTGAGAAGTTTGTGTTCTGGGATGTCACGCACCCAACAACAGC
AACTCACCGCTATGTTGCAGAGAAAATGCTAGAAAGTAGCAACAACTTAGCCGAGTACCGTTTCTAA

>10-7205
ATGATGAAAAAAACAATCACACTATTAACTGCATTACTCCCGCTTGCTTCTGCAGTTGCCGAAGAGCCAA
CCTTATCACCAGAAATGGTTTCAGCGTCTGAAGTGATCAGCACGCAAGAAAACCAAACCTATACCTATGT
TCGCTGTTGGTATCGCACCAGCTACTCGAAAGATGATCCGGCGACCGATTGGGAATGGGCAAAAAACGAA
GATGGTAGCTACTTCACCATTGACGGCTACTGGTGGAGCTCCGTTTCATTTAAAAACATGTTCTACACCA
ACACGTCGCAAAACGTTATCCGTCAGCGTTGTGAAGCCACATTAGATTTGGCGAACGAGAACGCAGACAT
TACGTTCTTCGCCGCTGACAATCGCTTCTCATACAACCACACGATCTGGAGCAACGACGCAGCAATGCAG
CCAGATCAAATCAACAAAGTGGTTGCACTCGGTGACAGCTTGTCTGATACAGGCAACATCTTTAACGCAT
CACAATGGCGCTTCCCTAACCCGAACAGCTGGTTCTTAGGTCACTTCTCCAACGGTTTTGTGTGGACAGA
ATACATTGCCAAAGCGAAGAACCTTCCGCTCTACAACTGGGCAGTTGGCGGCGCGGCTGGTGAGAACCAA
TACATCGCGCTAACAGGGGTTGGTGATCAAGTTTCTTCGTACTTAACCTACGCAAAACTGGCGAAGAACT
ACAAACCAGCAAACACCTTGTTTACGCTTGAGTTTGGTTTGAATGACTTCATGAACTACAACCGTGGCGT
TCCAGAAGTGAAAGCAGATTATGCAGAAGCACTGATTCGTTTGACGGACGCAGGTGCGAAGAACTTCATG
TTGATGACACTGCCAGACGCGACGAAAGCGCCTCAGTTTAAGTACTCAACACAAGAAGAGATCGACAAAA
TTCGTGCGAAAGTGCTTGAGATGAACGAGTTCATCAAGGCACAAGCGATGTACTACAAAGCGCAAGGTTA
CAACATCACGTTGTTTGATACTCACGCCTTGTTCGAGACGCTAACTTCTGCGCCCGAAAAGCACGGTTTC
GTGAACGCGAGTGATCCTTGTTTGGACATCAACCGCTCATCGTCTGTCGATTACATGTACACCCACGCAT
TGCGCTCTGAGTGTGCGGCGTCTGGTGCTGAGAAGTTTGTGTTCTGGGATGTCACGCACCCAACAACAGC
AACTCACCGCTATGTTGCAGAGAAAATGCTAGAAAGTAGCAACAACTTAGCCGAGTACCGTTTCTAA

>MAVP-E
ATGATGAAAAAAACAATCACACTATTAACTGCATTACTCCCGCTTGCTTCTGCAGTTGCCGAAGAGCCAACCTTAT
CACCAGAAATGGTTTCAGCGTCTGAAGTGATCAGCACGCAAGAAAACCAAACCTATACCTATGTTCGCTGTTGGTA
TCGCACCAGCTACTCGAAAGATGATCCGGCGACCGATTGGGAATGGGCAAAAAACGAAGATGGTAGCTACTTCAC
CATTGACGGCTACTGGTGGAGCTCCGTTTCATTTAAAAACATGTTCTACACCAACACGTCGCAAAACGTTATCCGT
CAGCGTTGTGAAGCAACATTAGATTTGGCGAACGAGAACGCAGACATTACGTTCTTCGCCGCTGACAATCGCTTCT
CATACAACCACACGATCTGGAGCAACGACGCAGCAATGCAGCCAGATCAAATCAACAAAGTGGTTGCACTCGGTG
ACAGCTTGTCTGATACAGGCAACATCTTTAACGCATCACAATGGCGCTTCCCTAACCCGAACAGCTGGTTCTTAGG
TCACTTCTCCAACGGTTTTGTGTGGACAGAATACATTGCCAAAGCGAAGAACCTTCCGCTCTACAACTGGGCAGTT
GGCGGCGCGGCTGGTGAGAACCAATACATCGCGCTAACAGGGGTTGGTGATCAAGTTTCTTCGTACTTAACCTACG
CAAAACTGGCGAAGAACTACAAACCAGCAAACACCTTGTTTACGCTTGAGTTTGGTTTGAATGACTTCATGAACTA
CAACCGTGGCGTTCCAGAAGTGAAAGCGGATTATGCAGAAGCACTGATTCGTTTGACGGACGCAGGTGCGAAGAA
CTTCATGTTGATGACACTGCCAGACGCGACGAAAGCGCCTCAGTTTAAGTACTCAACACAAGAAGAGATCGACAA
AATTCGTGCGAAAGTGCTTGAGATGAACGAGTTCATCAAGGCACAAGCGATGTACTACAAAGCGCAAGGTTACAA
CATCACGTTGTTTGATACTCACGCCTTGTTCGAGACGCTAACTTCTGCGCCAGAAGAGCACGGTTTCGTGAACGCG
AGCGATCCTTGTTTGGACATCAACCGCTCATCGTCTGTCGATTACATGTACACCCACGCATTGCGCTCTGAGTGTGC
AGCGTCTGGTGCTGAGAAGTTTGTGTTCTGGGATGTCACGCATCCAACAACAGCAACTCACCGCTATGTTGCAGAG
AAAATGCTAGAAAGTAGCAACAACTTAGCAGAGTACCGTTTCTAA

>MAVP-45
ATGATGAAAAAAACAATCACACTATTAACTGCATTACTCCCGCTTGCTTCTGCAGTTGCCGAAGAGCCAACCTTAT
CACCAGAAATGGTTTCAGCGTCTGAAGTGATCAGCACGCAAGAAAACCAAACCTATACCTATGTTCGCTGTTGGTA
TCGCACCAGCTACTCGAAAGATGATCCGGCGACCGATTGGGAATGGGCAAAAAACGAAGATGGTAGCTACTTCAC
CATTGACGGCTACTGGTGGAGCTCCGTTTCACTTAAAAACATGTTCTACACCAACACGTCGCAAAACGTTATCCGT
CAGCGTTGTGAAGCAACATTAGATTTGGCGAACGAGAACGCAGACATTACGTTCTTCGCCGCTGACAATCGCTTCT
CATACAACCACACGATCTGGAGCAACGACGCAGCAATGCAGCCAGATCAAATCAACAAAGTGGTTGCACTCGGTG
ACAGCTTGTCTGATACAGGCAACATCTTTAACGCATCACAATGGCGCTTCCCTAACCCGAACAGCTGGTTCTTAGG
TCACTTCTCCAACGGTTTTGTGTGGACAGAATACATTGCCAAAGCGAAGAACCTTCCGCTCTACAACTGGGCAGTT
GGCGGCGCGGCTGGTGAGAACCAATACATCGCGCTAACAGGGGTTGGTGATCAAGTTTCTTCGTACTTAACCTACG
CAAAACTGGCGAAGAACTACAAACCAGCAAACACCTTGTTTACGCTTGAGTTTGGTTTGAATGACTTCATGAACTA
CAACCGTGGCGTTCCAGAAGTGAAAGCAGATTATGCAGAAGCACTGATTCGTTTGACGGACGCAGGTGCGAAGAA
CTTCATGTTGATGACACTGCCAGACGCGACGAAAGCGCCTCAGTTTAAGTACTCAACACAAGAAGAGATCGACAA
AATTCGTGCGAAAGTGCTTGAGATGAACGAGTTCATCAAGGCACAAGCGATGTACTACAAAGCGCAAGGTTACAA
CATCACGTTGTTTGATACTCACGCCTTGTTCGAGACGCTAACTTCTGCGCCAGAAGAGCACGGTTTCGTGAACGCG
AGCGATCCTTGTTTGGACATCAACCGCTCATCGTCTGTCGATTACATGTACACCCACGCATTGCGCTCTGAGTGTGC
AGCGTCTGGTGCTGAGAAGTTTGTGTTCTGGGATGTCACGCATCCAACAACAGCAACTCACCGCTATGTTGCAGAG
AAAATGCTAGAAAGTAGCAACAACTTAGCCGAGTACCGTTTCTAA

>MAVP-V
ATGATGAAAAAAACAATCACACTATTAACTGCATTACTCCCGCTTGCTTCTGCAGTTGCCGAAGAGCCAACCTTAT
CACCAGAAATGGTTTCAGCGTCTGAAGTGATCAGCACGCAAGAAAACCAAACCTATACCTATGTTCGCTGTTGGTA
TCGCACCAGCTACTCGAAAGATGATCCGGCGACCGATTGGGAATGGGCAAAAAACGAAGATGGTAGCTACTTCAC
CATTGACGGCTACTGGTGGAGCTCCGTTTCACTTAAAAACATGTTCTACACCAACACGTCGCAAAACGTTATCCGT
CAGCGTTGTGAAGCAACATTAGATTTGGCGAACGAGAACGCAGACATTACGTTCTTCGCCGCTGACAATCGCTTCT
CATACAACCACACGATCTGGAGCAACGACGCAGCAATGCAGCCAGATCAAATCAACAAAGTGGTTGCACTCGGTG
ACAGCTTGTCTGATACAGGCAACATCTTTAACGCATCACAATGGCGCTTCCCTAACCCGAACAGCTGGTTCTTAGG
TCACTTCTCCAACGGTTTTGTGTGGACAGAATACATTGCCAAAGCGAAGAACCTTCCGCTCTACAACTGGGCAGTT
GGCGGCGCGGCTGGTGAGAACCAATACATCGCGCTAACAGGGGTTGGTGATCAAGTTTCTTCGTACTTAACCTACG

CAAAACTGGCGAAGAACTACAAACCAGCAAACACCTTGTTTACGCTTGAGTTTGGTTTGAATGACTTCATGAACTA
CAACCGTGGCGTTCCAGAAGTGAAAGCAGATTATGCAGAAGCACTGATTCGTTTGACGGACGCAGGTGCGAAGAA
CTTCATGTTGATGACACTGCCAGACGCGACGAAAGCGCCTCAGTTTAAGTACTCAACACAAGAAGAGATCGACAA
AATTCGTGCGAAAGTGCTTGAGATGAACGAGTTCATCAAGGCACAAGCGATGTACTACAAAGCGCAAGGTTACAA
CATCACGTTGTTTGATACTCACGCCTTGTTCGAGACGCTAACTTCTGCGCCAGAAGAGCACGGTTTCGTGAACGCG
AGCGATCCTTGTTTGGACATCAACCGCTCATCGTCTGTCGATTACATGTACACCCACGCATTGCGCTCTGAGTGTGC
AGCGTCTGGTGCTGAGAAGTTTGTGTTCTGGGATGTCACGCATCCAACAACAGCAACTCACCGCTATGTTGCAGAG
AAAATGCTAGAAAGTAGCAACAACTTAGCCGAGTACCGTTTCTAA

>ATCC 17802
ATGATGAAAAAAACAATCACACTATTAACTGCATTACTCCCGCTTGCTTCTGCAGTTGCCGAAGAGCCAACCTTAT
CACCAGAAATGGTTTCAGCGTCTGAAGTGATCAGCACGCAAGAAAACCAAACCTATACCTATGTTCGCTGTTGGTA
TCGCACCAGCTACTCGAAAGATGATCCGGCGACCGATTGGGAATGGGCAAAAAACGAAGATGGTAGCTACTTCAC
CATTGACGGCTACTGGTGGAGCTCCGTTTCATTTAAAAACATGTTCTACACCAACACGTCGCAAAACGTTATCCGT
CAGCGTTGTGAAGCAACATTAGATTTGGCGAACGAGAACGCAGACATTACGTTCTTCGCCGCTGACAATCGCTTCT
CATACAACCACACGATCTGGAGCAACGACGCAGCAATGCAGCCAGATCAAATCAACAAAGTGGTTGCACTCGGTG
ACAGCTTGTCTGATACAGGCAACATATTTAACGCATCACAATGGCGCTTCCCTAACCCGAACAGCTGGTTCTTAGG
TCACTTCTCCAACGGTTTTGTGTGGACAGAATACATTGCCAAAGCGAAGAACCTTCCGCTCTACAACTGGGCAGTT
GGCGGCGCGGCTGGTGAGAACCAATACATCGCGCTAACAGGGGTTGGTGATCAAGTTTCTTCGTACTTAACCTACG
CAAAACTGGCGAAGAACTACAAACCAGCAAACACCTTGTTTACGCTTGAGTTTGGTTTGAATGACTTCATGAACTA
CAACCGTGGCGTTCCAGAAGTGAAAGCGGATTATGCAGAAGCACTGATTCGTTTGACGGACGCAGGTGCGAAGAA
CTTCATGTTGATGACACTGCCAGATGCGACGAAAGCGCCTCAGTTTAAGTACTCAACACAAGAAGAGATCGACAA
AATTCGTGCGAAAGTGCTTGAGATGAACGAGTTCATCAAGGCACAAGCGATGTACTACAAAGCGCAAGGTTACAA
CATCACGTTGTTTGATACTCACGCCTTGTTCGAGACGCTAACTTCTGCGCCAGAAGAGCACGGTTTCGTGAACGCG
AGTGATCCTTGTTTGGACATCAACCGCTCATCGTCTGTCGATTACATGTACACCCACGCATTGCGCTCTGAGTGTGC
AGCATCTGGTGCTGAGAAGTTTGTGTTCTGGGATGTCACGCATCCAACAACAGCAACTCACCGCTATGTTGCAGAG
AAAATGCTAGAAAGTAGCAACAACTTAGCAGAGTACCGTTTCTAA

>VH3
ATGATGAAAAAAACAATCACACTATTAACTGCATTACTCCCGCTTGCTTCTGCAGTTGCCGAAGAGCCAACCTTAT
CACCAGAAATGGTTTCAGCGTCTGAAGTGATCAGCACGCAAGAAAACCAAACCTATACCTATGTTCGCTGTTGGTA
TCGCACCAGCTACTCGAAAGATGATCCGGCGACCGATTGGGAATGGGCAAAAAACGAAGATGGTAGCTACTTCAC
CATTGACGGCTACTGGTGGAGCTCCGTTTCATTTAAAAACATGTTCTACACCAACACGTCGCAAAACGTTATCCGT
CAGCGTTGTGAAGCCACATTAGATTTGGCGAACGAGAACGCAGACATTACGTTCTTCGCCGCTGACAATCGCTTCT
CATACAACCACACGATCTGGAGCAACGACGCAGCAATGCAGCCAGATCAAATCAACAAAGTGGTTGCACTCGGTG
ACAGCTTGTCTGATACAGGCAACATCTTTAACGCATCACAATGGCGCTTCCCTAACCCGAACAGCTGGTTCTTAGG
TCACTTCTCCAACGGTTTTGTGTGGACAGAATACATTGCCAAAGCGAAGAACCTTCCGCTCTACAACTGGGCAGTT
GGCGGCGCGGCTGGTGAGAACCAATACATCGCGCTAACAGGGGTTGGTGATCAAGTTTCTTCGTACTTAACCTACG
CAAAACTGGCGAAGAACTACAAACCAGCAAACACCTTGTTTACGCTTGAGTTTGGTTTGAATGACTTCATGAACTA
CAACCGTGGCGTTCCAGAAGTGAAAGCAGATTATGCAGAAGCACTGATTCGTTTGACGGACGCAGGTGCGAAGAA
CTTCATGTTGATGACACTGCCAGACGCGACGAAAGCGCCTCAGTTTAAGTACTCAACACAAGAAGAGATCGACAA
AATTCGTGCGAAAGTGCTTGAGATGAACGAGTTCATCAAGGCACAAGCGATGTACTACAAAGCGCAAGGTTACAA
CATCACGTTGTTTGATACTCACGCCTTGTTCGAGACGCTAACTTCTGCGCCCGAAGAGCACGGTTTCGTGAACGCG
AGTGATCCTTGTTTGGACATCAACCGCTCATCGTCTGTCGATTACATGTACACCCACGCATTGCGCTCTGAGTGTGC
GGCGTCTGGTGCTGAGAAGTTTGTGTTCTGGGATGTCACGCACCCAACAACAGCAACTCACCGCTATGTTGCAGAG
AAAATGCTAGAAAGTAGCAACAACTTAGCCGAGTACCGTTTCTAA

>MAVP-26
ATGATGAAAAAAACAATCACACTATTAACTGCATTACTCCCGCTTGCTTCTGCAGTTGCCGAAGAGCCAACCTTAT
CACCAGAAATGGTTTCAGCGTCTGAAGTGATCAGCACGCAAGAAAACCAAACCTATACCTATGTTCGCTGTTGGTA
TCGCACCAGCTACTCGAAAGATGATCCGGCGACCGATTGGGAATGGGCAAAAAACGAAGATGGTAGCTACTTCAC

CATTGACGGCTACTGGTGGAGCTCCGTTTCACTTAAAAACATGTTCTACACCAACACGTCGCAAAACGTTATCCGT
CAGCGTTGTGAAGCAACATTAGATTTGGCGAACGAGAACGCAGACATTACGTTCTTCGCCGCTGACAATCGCTTCT
CATACAACCACACGATCTGGAGCAACGACGCAGCAATGCAGCCAGATCAAATCAACAAAGTGGTTGCACTCGGTG
ACAGCTTGTCTGATACAGGCAACATCTTTAACGCATCACAATGGCGCTTCCCTAACCCGAACAGCTGGTTCTTAGG
TCACTTCTCCAACGGTTTTGTGTGGACAGAATACATTGCCAAAGCGAAGAACCTTCCGCTCTACAACTGGGCAGTT
GGCGGCGCGGCTGGTGAGAACCAATACATCGCGCTAACAGGGGTTGGTGATCAAGTTTCTTCGTACTTAACCTACG
CAAAACTGGCGAAGAACTACAAACCAGCAAACACCTTGTTTACGCTTGAGTTTGGTTTGAATGACTTCATGAACTA
CAACCGTGGCGTTCCAGAAGTGAAAGCAGATTATGCAGAAGCACTGATTCGTTTGACGGACGCAGGTGCGAAGAA
CTTCATGTTGATGACACTGCCAGACGCGACGAAAGCGCCTCAGTTTAAGTACTCAACACAAGAAGAGATCGACAA
AATTCGTGCGAAAGTGCTTGAGATGAACGAGTTCATCAAGGCACAAGCGATGTACTACAAAGCGCAAGGTTACAA
CATCACGTTGTTTGATACTCACGCCTTGTTCGAGACGCTAACTTCTGCGCCAGAAGAGCACGGTTTCGTGAACGCG
AGCGATCCTTGTTTGGACATCAACCGCTCATCGTCTGTCGATTACATGTACACCCACGCATTGCGCTCTGAGTGTGC
AGCGTCTGGTGCTGAGAAGTTTGTGTTCTGGGATGTCACGCATCCAACAACAGCAACTCACCGCTATGTTGCAGAG
AAAATGCTAGAAAGTAGCAACAACTTAGCCGAGTACCGTTTCTAA

>MAVP-36
ATGATGAAAAAAACAATCACACTATTAACTGCATTACTCCCGCTTGCTTCTGCAGTTGCCGAAGAGCCAACCTTAT
CACCAGAAATGGTTTCAGCGTCTGAAGTGATCAGCACGCAAGAAAACCAAACCTATACCTATGTTCGCTGTTGGTA
TCGCACCAGCTACTCGAAAGATGATCCGGCGACCGATTGGGAATGGGCAAAAAACGAAGATGGTAGCTACTTCAC
CATTGACGGCTACTGGTGGAGCTCCGTTTCACTTAAAAACATGTTCTACACCAACACGTCGCAAAACGTTATCCGT
CAGCGTTGTGAAGCAACATTAGATTTGGCGAACGAGAACGCAGACATTACGTTCTTCGCCGCTGACAATCGCTTCT
CATACAACCACACGATCTGGAGCAACGACGCAGCAATGCAGCCAGATCAAATCAACAAAGTGGTTGCACTCGGTG
ACAGCTTGTCTGATACAGGCAACATCTTTAACGCATCACAATGGCGCTTCCCTAACCCGAACAGCTGGTTCTTAGG
TCACTTCTCCAACGGTTTTGTGTGGACAGAATACATTGCCAAAGCGAAGAACCTTCCGCTCTACAACTGGGCAGTT
GGCGGCGCGGCTGGTGAGAACCAATACATCGCGCTAACAGGGGTTGGTGATCAAGTTTCTTCGTACTTAACCTACG
CAAAACTGGCGAAGAACTACAAACCAGCAAACACCTTGTTTACGCTTGAGTTTGGTTTGAATGACTTCATGAACTA
CAACCGTGGCGTTCCAGAAGTGAAAGCAGATTATGCAGAAGCACTGATTCGTTTGACGGACGCAGGTGCGAAGAA
CTTCATGTTGATGACACTGCCAGACGCGACGAAAGCGCCTCAGTTTAAGTACTCAACACAAGAAGAGATCGACAA
AATTCGTGCGAAAGTGCTTGAGATGAACGAGTTCATCAAGGCACAAGCGATGTACTACAAAGCGCAAGGTTACAA
CATCACGTTGTTTGATACTCACGCCTTGTTCGAGACGCTAACTTCTGCGCCAGAAGAGCACGGTTTCGTGAACGCG
AGCGATCCTTGTTTGGACATCAACCGCTCATCGTCTGTCGATTACATGTACACCCACGCATTGCGCTCTGAGTGTGC
AGCGTCTGGTGCTGAGAAGTTTGTGTTCTGGGATGTCACGCATCCAACAACAGCAACTCACCGCTATGTTGCAGAG
AAAATGCTAGAAAGTAGCAACAACTTAGCCGAGTACCGTTTCTAA

>CT4287
ATGATGAAAAAAACAATCACACTATTAACTGCATTACTCCCGCTTGCTTCTGCAGTTGCCGAAGAGCCAACCTTAT
CACCAGAAATGGTTTCAGCGTCTGAAGTGATCAGCACGCAAGAAAACCAAACCTATACCTATGTTCGCTGTTGGTA
TCGCACCAGCTACTCGAAAGATGATCCGGCGACCGATTGGGAATGGGCAAAAAACGAAGATGGTAGCTACTTCAC
CATTGACGGCTACTGGTGGAGCTCCGTTTCATTTAAAAACATGTTCTACACCAACACGTCGCAAAACGTTATCCGT
CAGCGTTGTGAAGCAACATTAGATTTGGCGAACGAGAACGCAGACATTACGTTCTTCGCCGCTGACAATCGCTTCT
CATACAACCACACGATCTGGAGCAACGACGCAGCAATGCAGCCAGATCAAATCAACAAAGTGGTTGCACTCGGTG
ACAGCTTGTCTGATACAGGCAACATCTTTAACGCATCACAATGGCGCTTCCCTAACCCGAACAGCTGGTTCTTAGG
TCACTTCTCCAACGGTTTTGTGTGGACAGAATACATTGCCAAAGCGAAGAACCTTCCGCTCTACAACTGGGCAGTT
GGCGGCGCGGCTGGTGAGAACCAATACATCGCGCTAACAGGGGTTGGTGATCAAGTTTCTTCGTACTTAACCTACG
CAAAACTGGCGAAGAACTACAAACCAGCAAACACCTTGTTTACGCTTGAGTTTGGTTTGAATGACTTCATGAACTA
CAACCGTGGCGTTCCAGAAGTGAAAGCGGATTATGCAGAAGCACTGATTCGTTTGACGGACGCAGGTGCGAAGAA
CTTCATGTTGATGACACTGCCAGACGCGACGAAAGCGCCTCAGTTTAAGTACTCAACACAAGAAGAGATCGACAA
AATTCGTGCGAAAGTGCTTGAGATGAACGAGTTCATCAAGGCACAAGCGATGTACTACAAAGCGCAAGGTTACAA
CATCACGTTGTTTGATACTCACGCCTTGTTCGAGACGCTAACTTCTGCGCCCGAAGAGCACGGTTTCGTGAACGCG
AGCGATCCTTGTTTGGACATCAACCGCTCATCGTCTGTCGACTACATGTACACCCACGCATTGCGCTCTGAGTGTGC

GGCGTCTGGTGCTGAGAAGTTTGTATTCTGGGATGTCACGCACCCAACAACAGCAACTCACCGCTATGTTGCAGAG
AAAATGCTAGAAAGTAGCAACAACTTAGCAGAGTACCGTTTCTAA

>MAVP-M
ATGATGAAAAAAACAATCACACTATTAACTGCATTACTCCCGCTTGCTTCTGCAGTTGCCGAAGAGCCAACCTTAT
CACCAGAAATGGTTTCAGCGTCTGAAGTGATCAGCACGCAAGAAAACCAAACCTATACCTATGTTCGCTGTTGGTA
TCGCACCAGCTACTCGAAAGATGATCCGGCGACCGATTGGGAATGGGCAAAAAACGAAGATGGTAGCTACTTCAC
CATTGACGGCTACTGGTGGAGCTCCGTTTCATTTAAAAACATGTTCTACACCAACACGTCGCAAAACGTTATCCGT
CAGCGTTGTGAAGCAACATTAGATTTGGCGAACGAGAACGCAGACATTACGTTCTTCGCCGCTGACAATCGCTTCT
CATACAACCACACGATCTGGAGCAACGACGCAGCAATGCAGCCAGATCAAATCAACAAAGTGGTTGCACTCGGTG
ACAGCTTGTCTGATACAGGCAACATCTTTAACGCATCACAATGGCGCTTCCCTAACCCGAACAGCTGGTTCTTAGG
TCACTTCTCCAACGGTTTTGTGTGGACAGAATACATTGCCAAAGCGAAGAACCTTCCGCTCTACAACTGGGCAGTT
GGCGGCGCGGCTGGTGAGAACCAATACATCGCGCTAACAGGGGTTGGTGATCAAGTTTCTTCGTACTTAACCTACG
CAAAACTGGCGAAGAACTACAAACCAGCAAACACCTTGTTTACGCTTGAGTTTGGTTTGAATGACTTCATGAACTA
CAACCGTGGCGTTCCAGAAGTGAAAGCAGATTATGCAGAAGCACTGATTCGTTTGACGGACGCAGGTGCGAAGAA
CTTCATGTTGATGACACTGCCAGACGCGACGAAAGCGCCTCAGTTTAAGTACTCAACACAAGAAGAGATCGACAA
AATTCGTGCGAAAGTGCTTGAGATGAACGAGTTCATCAAGGCACAAGCGATGTACTACAAAGCGCAAGGTTACAA
CATCACGTTGTTTGATACTCACGCCTTGTTCGAGACGCTAACTTCTGCGCCCGAAGAGCACGGTTTCGTGAACGCG
AGTGATCCTTGTTTGGACATCAACCGCTCATCGTCTGTCGATTACATGTACACCCACGCATTGCGCTCTGAGTGTGC
GGCGTCTGGTGCTGAGAAGTTTGTGTTCTGGGATGTCACGCACCCAACAACAGCAACTCACCGCTATGTTGCAGAG
AAAATGCTAGAAAGTAGCAACAACTTAGCCGAGTACCGTTTCTAA

>CFSAN001614-2

ATGATGAAAAAAACAATCACACTATTAACTGCATTACTCCCGCTTGCTTCTGCAGTTGCCGAAGAGCCAACCTTAT
CACCAGAAATGGTTTCAGCGTCTGAAGTGATCAGCACGCAAGAAAACCAAACCTATACCTATGTTCGCTGTTGGTA
TCGCACCAGCTACTCGAAAGATGATCCGGCGACCGATTGGGAATGGGCAAAAAACGAAGATGGTAGCTACTTCAC
CATTGACGGCTACTGGTGGAGCTCCGTTTCACTTAAAAACATGTTCTACACCAACACGTCGCAAAACGTTATCCGT
CAGCGTTGTGAAGCAACATTAGATTTGGCGAACGAGAACGCAGACATTACGTTCTTCGCCGCTGACAATCGCTTCT
CATACAACCACACGATCTGGAGCAACGACGCAGCAATGCAGCCAGATCAAATCAACAAAGTGGTTGCACTCGGTG
ACAGCTTGTCTGATACAGGCAACATCTTTAACGCATCACAATGGCGCTTCCCTAACCCGAACAGCTGGTTCTTAGG
TCACTTCTCCAACGGTTTTGTGTGGACAGAATACATTGCCAAAGCGAAGAACCTTCCGCTCTACAACTGGGCAGTT
GGCGGCGCGGCTGGTGAGAACCAATACATCGCGCTAACAGGGTTGGTGATCAAGTTTCTTCGTACTTAACCTACGC
AACACTGGCGAAGAACTACAAACCAGCAAACACCTTGTTTACGCTTGAGTTTGGTTTGAATGACTTCATGAACTAC
AACCGTGGCGTTCCAGAAGTGAAAGCAGATTATGCAGAAGCACTGATTCGTTTGACGGACGCAGGTGCGAAGAAC
TTCATGTTGATGACACTGCCAGACGCGACGAAAGCGCCTCAGTTTAAGTACTCAACACAAGAAGAGATCGACAAA
ATTCGTGCGAAAGTGCTTGAGATGAACGAGTTCATCAAGGCACAAGCGATGTACTACAAAGCGCAAGGTTACAAC
ATCACGTTGTTTGATACTCACGCCTTGTTCGAGACGCTAACTTCTGCGCCAGAAGAGCACGGTTTCGTGAACGCGA
GCGATCCTTGTTTGGACATCAACCGCTCATCGTCTGTCGATTACATGTACACCCACGCATTGCGCTCTGAGTGTGCA
GCGTCTGGTGCTGAGAAGTTTGTGTTCTGGGATGTCACGCATCCAACAACAGCAACTCACCGCTATGTTGCAGAGA
AAATGCTAGAAAGTAGCAACAACTTAGCCGAGTACCGTTTCTAA

>CFSAN001153
ATGATGAAAAAAACAATCACACTATTAACTGCATTACTCCCGCTTGCTTCTGCAGTTGCCGAAGAGCCAA
CCTTATCACCAGAAATGGTTTCAGCGTCTGAAGTGATCAGCACGCAAGAAAACCAAACCTATACCTATGT
TCGCTGTTGGTATCGCACCAGCTACTCGAAAGATGATCCGGCGACCGATTGGGAATGGGCAAAAAACGAA
GATGGTAGCTACTTCACCATTGACGGCTACTGGTGGAGCTCCGTTTCACTTAAAAACATGTTCTACACCA
ACACGTCGCAAAACGTTATCCGTCAGCGTTGTGAAGCAACATTAGATTTGGCGAACGAGAACGCAGACAT

TACGTTCTTCGCCGCTGACAATCGCTTCTCATACAACCACACGATCTGGAGCAACGACGCAGCAATGCAG
CCAGATCAAATCAACAAAGTGGTTGCACTCGGTGACAGCTTGTCTGATACAGGCAACATCTTTAACGCAT
CACAATGGCGCTTCCCTAACCCGAACAGCTGGTTCTTAGGTCACTTCTCCAACGGTTTTGTGTGGACAGA
ATACATTGCCAAAGCGAAGAACCTTCCGCTCTACAACTGGGCAGTTGGCGGCGCGGCTGGTGAGAACCAA
TACATCGCGCTAACAGGGGTTGGTGATCAAGTTTCTTCGTACTTAACCTACGCAAAACTGGCGAAGAACT
ACAAACCAGCAAACACCTTGTTTACGCTTGAGTTTGGTTTGAATGACTTCATGAACTACAACCGTGGCGT
TCCAGAAGTGAAAGCAGATTATGCAGAAGCACTGATTCGTTTGACGGACGCAGGTGCGAAGAACTTCATG
TTGATGACACTGCCAGACGCGACGAAAGCGCCTCAGTTTAAGTACTCAACACAAGAAGAGATCGACAAAA
TTCGTGCGAAAGTGCTTGAGATGAACGAGTTCATCAAGGCACAAGCGATGTACTACAAAGCGCAAGGTTA
CAACATCACGTTGTTTGATACTCACGCCTTGTTCGAGACGCTAACTTCTGCGCCAGAAGAGCACGGTTTC
GTGAACGCGAGCGATCCTTGTTTGGACATCAACCGCTCATCGTCTGTCGATTACATGTACACCCACGCAT
TGCGCTCTGAGTGTGCAGCGTCTGGTGCTGAGAAGTTTGTGTTCTGGGATGTCACGCATCCAACAACAGC
AACTCACCGCTATGTTGCAGAGAAAATGCTAGAAAGTAGCAACAACTTAGCCGAGTACCGTTTCTAA

All the 13 tlh genes from 10 whole genome sequences and 3 nr database of Vibrio alginolyticus:

>NBRC 15630
ATGATGAAAAAAACAATCACACTATTAACTGCATTACTTCCGCTTGCCTCCGCCATTGCA
GAGGAGCCAACTTTATCACCGGCAATGGTTTCTGCGGCTGAAGTGGTTAGCGCGCAAGAA
AATCAAACTTATACGTACGTTCGATGCTGGTATCGTACGAGCCACTCTAAGGACGATGCG
GCTACCGATTGGAAATGGGCAAAAAACCAAGATGGTAGTGACTTCACTATCGATGGTTAT
TGGTGGAGCTCTGTCTCATTTAAAAACATGTTCTACACCAATACCTCACAAAACGTAATT
CGCCAGCGTTGTGAAGAAACACTAGATCTAGCGAACGAGAACGCAGACATTACATTTTTT
GCTGCTGACAATCGTTATTCTTATAACCACACGATCTGGAGCAATGATGCTGCAATGCAA
CCAGATCAAATCAACAAGGTAGTGGCTTTGGGCGATAGCTTGTCTGATACAGGCAACATC
TTTAACGCGTCCCAATGGCGCTTCCCTAACCCGAATAGCTGGTTCTTGGGTCACTTCTCA
AACGGTTTTGTTTGGACGGAATACGTTGCTAAAGCCAAAAACTTACCGCTATACAACTGG
GCAGTTGGTGGCGCAGCAGGTGAAAACCAATACATCGCGCTAACTGGTGTCGGCGAACAA
GTTTCTTCTTACTTAACCTACACAAAGCTGGCGAAGAACTACAACCCTGCTAACACATTG
TTTACGTTAGAGTTTGGTTTAAATGATTTCATGAACTACAACCGTAGCGTGCCGGAAGTA
AAAGCGGATTACGCTGAAGCTCTGATTCGTTTAACAGACGCTGGCGCGAAGAACTTCATG
TTGATGACACTGCCTGATGCAACCAAAGCACCACAGTTCAAATACTCAACACAGGAAGAA
ATTGAAACGATTCGTGCGAAAGTATTGAAGATGAACGAGTTCATCAAAGCACAAGCGATG
TACTACAAAGCCCAAGGCTACAACATCGCATTGTTTGATACACACGCACTGTTTGAGAAG
TTAACCTCGGCACCAGAAGAACACGGTTTCGTAAATGCAAGCGACCCTTGCTTAGACATC
AACCGTTCATCCTCTGTTGACTACATGTACACTCACTCATTACGTTCTGAATGTGCAGCA
TCTGGCGCAGATAAGTTCGTGTTCTGGGATGTGACTCACCCAACCACAGCAACGCATCGT
TATGTGGCTGAAAAAATGTTAGAAAGCAGCAATAACTTAGAAGAGTTTCGCTTTTAA

>12G01
ATGATGAAAAAAACAATCACACTATTAACTGCATTACTTCCGCTTGCCTCCGCCATTGCTGAGGAGCCAA
CTTTATCACCAGCAATGGTTTCTGCGGCTGAAGTGGTTAGCGCGCAAGAAAATCAAACTTATACGTACGT
TCGATGCTGGTATCGTACGAGCCACTCTAAGGACGATGCGGCTACCGATTGGAAATGGGCAAAAAACCAA
GATGGTAGTGACTTCACTATCGATGGCTATTGGTGGAGCTCTGTTTCATTTAAAAACATGTTCTACACCA
ATACCTCACAAAACGTAATTCGTCAGCGTTGTGAAGAAACACTAGATCTAGCGAACGAGAACGCAGACAT
TACATTTTTTGCTGCTGACAATCGTTATTCTTATAACCACACGATCTGGAGCAATGATGCTGCAATGCAA
CCAGATCAGATAAACAAAGTAGTGGCTTTAGGCGATAGCTTGTCTGATACAGGCAACATCTTTAACGCGT
CCCAATGGCGCTTTCCTAACCCGAATAGCTGGTTCTTGGGTCACTTCTCAAACGGTTTTGTTTGGACGGA
ATACGTTGCTAAAGCCAAAAACTTACCGCTATACAACTGGGCAGTTGGTGGCGCAGCAGGTGAAAACCAA
TACATCGCGCTAACTGGTGTCGGCGAACAAGTTTCTTCTTACTTAACCTACACCAAGCTGGCGAAGAACT
ACAACCCTGCTAACACATTGTTTACGTTAGAGTTTGGTTTAAATGATTTCATGAACTACAACCGTAGCGT

GCCGGAAGTAAAAGCGGATTACGCTGAAGCTCTGATTCGTTTAACAGACGCTGGCGCGAAGAACTTCATG
TTGATGACACTGCCTGATGCAACCAAAGCACCACAGTTCAAATACTCAACACAGGAAGAAATTGAAACGA
TTCGTGCGAAAGTATTGAAAATGAACGAGTTCATCAAAGCACAAGCGATGTACTACAAAGCCCAAGGCTA
CAACATCGCATTGTTTGATACACACGCACTGTTTGAGAAGTTAACCTCGGCACCAGAAGAACACGGTTTC
GTAAATGCAAGCGACCCTTGCTTAGACATCAACCGTTCATCCTCTGTTGACTACATGTACACTCACTCAT
TACGTTCTGAATGTGCAGCATCTGGCGTAGATAAGTTCGTGTTCTGGGATGTGACTCACCCAACCACAGC
AACGCATCGTTATGTGGCTGAAAAAATGTTAGAAAGCAGCAATAACTTAGAAGAGTTTCGCTTTTAA

>40B
ATGATGAAAAAAACAATCACACTATTAACTGCATTACTTCCGCTTGCCTCCGCCATTGCAGAGGAGCCAACTTTAT
CACCAGCAATGGTTTCTGCGGCTGAAGTGGTTAGCGCGCAAGAAAATCAAACTTATACGTACGTTCGATGCTGGTA
TCGTACGAGCCACTCTAAGGACGATGCGGCTACCGATTGGAAATGGGCAAAAAACCAAGATGGTAGTGACTTCAC
TATCGATGGTTATTGGTGGAGCTCTGTTTCATTTAAAAACATGTTCTACACCAATACCTCACAAAACGTAATTCGCC
AGCGTTGTGAGGAAACACTAGATCTAGCGAACGAGAACGCAGACATTACATTTTTTGCTGCCGACAATCGTTATTC
TTATAACCACACGATCTGGAGCAATAATGCTGCAATGCAACCAGATCAAATCAACAAGGTGGTGGCTTTGGGCGA
TAGCTTGTCTGATACAGGCAACATCTTTAACGCGTCCCAATGGCGCTTCCCTAACCCTAATAGCTGGTTCTTGGGTC
ACTTCTCAAACGGTTTTGTTTGGACGGAATACGTTGCTAAAGCCAAAAACTTACCGCTATACAACTGGGCAGTTGG
TGGCGCAGCAGGTGAAAACCAATACATCGCGCTAACTGGTGTCGGCGAACAAGTTTCTTCTTACTTAACCTACACA
AAGCTGGCGAAGAACTACAACCCTGCTAACACATTGTTTACGTTAGAGTTTGGTTTAAATGATTTCATGAACTACA
ACCGTAGCGTGCCGGAAGTAAAAGCGGATTACGCTGAAGCTCTGATTCGTTTAACAGACGCTGGCGCGAAGAACT
TCATGTTGATGACACTGCCAGATGCAACCAAAGCACCACAGTTCAAATACTCAACACAGGAAGAAATTGAAACGA
TTCGTGCGAAAGTATTGAAGATGAACGAGTTCATCAAAGCACAAGCGATGTACTACAAAGCCCAAGGCTACAACA
TCGCATTGTTTGATACACACGCACTGTTTGAGAAGTTAACCTCGGCACCAGAAGAACACGGTTTCGTAAATGCAAG
CGACCCTTGCTTAGACATCAACCGTTCATCCTCTGTTGACTACATGTACACTCACTCATTACGTTCTGAATGTGCAG
CATCTGGCGCAGATAAGTTCGTGTTCTGGGATGTGACTCACCCAACCACAGCAACGCATCGTTATGTGGCTGAAAA
AATGTTAGAAAGCAGCAATAACTTAGAAGAGTTTCGCTTTTAA

>E0666
ATGATGAAAAAAACAATCACACTATTAACTGCATTACTTCCGCTTGCCTCTGCCGTTGCAGAGGAGCCAA
CATTATCACCAGAAATGGTTTCTGCGTCTGAAGTGGTCAGCACGCAAGAAAATCAAACCTATACTTACGT
TCGCTGCTGGTATCGTACGAGCCACTCTAAGGATGATGCGGCTACCGACTGGAAATGGGCGAAAAACCAA
GATGGTAGTGACTTCACTATCGACGGTTATTGGTGGAGCTCTGTTTCATTTAAAAACATGTTCTACACCA
ACACTTCACAAAACGTGATTCGCCAGCGCTGTGAAGAAACACTAGATCTGGCGAACGAGAACGCAGACAT
TACGTTTTTTGCTGCTGACAATCGCTATTCTTATAACCACACGATCTGGAGCAATGATGCTGCAATGCAA
CCAGATCAAATCAACAAAGTAGTGGCTTTGGGCGACAGCTTATCTGATACAGGCAACATCTTTAACGCTT
CCCAATGGCGCTTCCCTAACCCAAACAGCTGGTTCTTAGGTCACTTCTCGAACGGTTTTGTTTGGACGGA
ATACGTTGCTAAAGCCAAAAACTTACCGCTTTATAACTGGGCAGTTGGCGGCGCAGCAGGTGAGAACCAA
TACATTGCGCTAACAGGTGTCGGCGAACAAGTTTCTTCTTACTTGACCTACACAAAACTGGCGAAAAACT
ACAACCCAGCAAACACATTGTTTACGTTGGAGTTTGGTTTAAATGATTTCATGAACTACAACCGTAGCGT
GCCGGAAGTAAAAGCGGATTACGCTGAAGCTCTGATTCGTCTAACAGACGCGGGCGCGAAGAACTTCATG
TTGATGACACTGCCAGACGCAACCAAAGCACCACAGTTCAAATACTCAACGCAGGAAGAAATTGAAACGA
TTCGCGCGAAAGTATTGAAAATGAACGAGTTCATCAAAGCACAAGCGATGTACTATAAAGCTCAAGGTTA
CAACATTTCATTGTTTGATACGCACGCGCTGTTTGAGACGTTAACCTCGGCACCAGAAGAACACGGGTTC
GTGAATGCAAGTGATCCTTGTCTGGACATCAACCGCTCATCTTCTGTGGATTACATGTACACCCATTCAT
TACGCTCTGAGTGTGCGGCATCTGGCGCAGATAAGTTTGTGTTCTGGGATGTGACTCACCCAACCACAGC
CACGCATCGTTATGTTGCTGAAAAAATGTTGGAAAGCAGCAATAACTTAGAAGAGTTTCGCTTTTAA

>NBRC 15630
ATGATGAAAAAAACAATCACACTATTAACTGCATTACTTCCGCTTGCCTCCGCCATTGCA
GAGGAGCCAACTTTATCACCGGCAATGGTTTCTGCGGCTGAAGTGGTTAGCGCGCAAGAA

AATCAAACTTATACGTACGTTCGATGCTGGTATCGTACGAGCCACTCTAAGGACGATGCG
GCTACCGATTGGAAATGGGCAAAAAACCAAGATGGTAGTGACTTCACTATCGATGGTTAT
TGGTGGAGCTCTGTCTCATTTAAAAACATGTTCTACACCAATACCTCACAAAACGTAATT
CGCCAGCGTTGTGAAGAAACACTAGATCTAGCGAACGAGAACGCAGACATTACATTTTTT
GCTGCTGACAATCGTTATTCTTATAACCACACGATCTGGAGCAATGATGCTGCAATGCAA
CCAGATCAAATCAACAAGGTAGTGGCTTTGGGCGATAGCTTGTCTGATACAGGCAACATC
TTTAACGCGTCCCAATGGCGCTTCCCTAACCCGAATAGCTGGTTCTTGGGTCACTTCTCA
AACGGTTTTGTTTGGACGGAATACGTTGCTAAAGCCAAAAACTTACCGCTATACAACTGG
GCAGTTGGTGGCGCAGCAGGTGAAAACCAATACATCGCGCTAACTGGTGTCGGCGAACAA
GTTTCTTCTTACTTAACCTACACAAAGCTGGCGAAGAACTACAACCCTGCTAACACATTG
TTTACGTTAGAGTTTGGTTTAAATGATTTCATGAACTACAACCGTAGCGTGCCGGAAGTA
AAAGCGGATTACGCTGAAGCTCTGATTCGTTTAACAGACGCTGGCGCGAAGAACTTCATG
TTGATGACACTGCCTGATGCAACCAAAGCACCACAGTTCAAATACTCAACACAGGAAGAA
ATTGAAACGATTCGTGCGAAAGTATTGAAGATGAACGAGTTCATCAAAGCACAAGCGATG
TACTACAAAGCCCAAGGCTACAACATCGCATTGTTTGATACACACGCACTGTTTGAGAAG
TTAACCTCGGCACCAGAAGAACACGGTTTCGTAAATGCAAGCGACCCTTGCTTAGACATC
AACCGTTCATCCTCTGTTGACTACATGTACACTCACTCATTACGTTCTGAATGTGCAGCA
TCTGGCGCAGATAAGTTCGTGTTCTGGGATGTGACTCACCCAACCACAGCAACGCATCGT
TATGTGGCTGAAAAAATGTTAGAAAGCAGCAATAACTTAGAAGAGTTTCGCTTTTAA

>BSW15
ATGATGAAAAAAACAATCACACTATTAACTGCATTACTTCCGCTTGCCTCCGCCATTGCAGAGGAGCCAA
CTTTATCACCAGCAATGGTTTCTGCGGCTGAAGTGGTTAGCGCGCAAGAAAATCAAACTTATACGTACGT
TCGATGCTGGTATCGTACGAGCCACTCTAAGGACGATGCGGCTACCGATTGGAAATGGGCAAAAAACAAA
GATGGTAGTGACTTCACTATCGATGGTTATTGGTGGAGCTCTGTTTCATTTAAAAACATGTTCTACACCA
ATACCTCACAAAACGTAATTCGCCAGCGTTGTGAAGAAACACTAGATCTAGCGAACGAGAACGCAGACAT
TACATTTTTTGCTGCCGACAATCGTTATTCTTATAACCACACGATCTGGAGCAATGATGCTGCAATGCAA
CCAGATCAGATAAACAAAGTAGTGGCTTTAGGCGATAGCTTGTCTGATACAGGCAACATCTTTAACGCGT
CCCAATGGCGCTTTCCTAACCCGAATAGCTGGTTCTTGGGTCACTTCTCAAACGGTTTTGTTTGGACGGA
ATACGTTGCTAAAGCCAAAAACTTACCGCTATACAACTGGGCAGTTGGTGGCGCAGCAGGTGAAAACCAA
TACATCGCGCTAACTGGTGTCGGCGAACAAGTTTCTTCTTACTTAACCTACACAAAGCTGGCGAAGAACT
ACAACCCTGCTAACACATTGTTTACGTTAGAGTTTGGTTTAAATGATTTCATGAACTACAACCGTAGCGT
GCCGGAAGTAAAAGCGGATTACGCTGAAGCTCTGATTCGTTTAACAGACGCTGGCGCGAAGAACTTCATG
TTGATGACACTGCCAGATGCAACCAAAGCACCACAGTTCAAATACTCAACACAGGAAGAAATTGAAACGA
TTCGTGCGAAAGTATTGAAGATGAACGAGTTCATCAAAGCACAAGCGATGTACTACAAAGCCCAAGGCTA
CAACATCGCATTGTTTGATACACACGCACTGTTTGAGAAGTTAACCTCGGCACCAGAAGAACACGGTTTC
GTAAATGCAAGCGACCCTTGCTTAGACATCAACCGTTCATCCTCTGTTGACTACATGTACACTCACTCAT
TACGTTCTGAATGTGCAGCATCTGGCGCAGATAAGTTCGTGTTCTGGGATGTGACTCACCCAACCACAGC
AACGCATCGTTATGTGGCTGAAAAAATGTTAGAAAGCAGCAATAACTTAGAAGAGTTTCGCTTTTAA

>BSW8
ATGATGAAAAAAACAATCACACTATTAACTGCATTACTTCCGCTTGCCTCCGCCATTGCAGAGGAGCCAACTTTAT
CACCAGCAATGGTTTCTGCGGCTGAAGTGGTTAGCGCGCAAGAAAATCAAACTTATACGTACGTTCGATGCTGGTA
TCGTACGAGCCACTCTAAGGACGATGCGGCTACCGATTGGAAATGGGCAAAAAACAAAGATGGTAGTGACTTCAC
TATCGATGGTTATTGGTGGAGCTCTGTTTCATTTAAAAACATGTTCTACACCAATACCTCACAAAACGTAATTCGCC
AGCGTTGTGAAGAAACACTAGATCTAGCGAACGAGAACGCAGACATTACATTTTTTGCTGCCGACAATCGTTATTC
TTATAACCACACGATCTGGAGCAATGATGCTGCAATGCAACCAGATCAGATAAACAAAGTAGTGGCTTTAGGCGA
TAGCTTGTCTGATACAGGCAACATCTTTAACGCGTCCCAATGGCGCTTTCCTAACCCGAATAGCTGGTTCTTGGGTC
ACTTCTCAAACGGTTTTGTTTGGACGGAATACGTTGCTAAAGCCAAAAACTTACCGCTATACAACTGGGCAGTTGG
TGGCGCAGCAGGTGAAAACCAATACATCGCGCTAACTGGTGTCGGCGAACAAGTTTCTTCTTACTTAACCTACACA
AAGCTGGCGAAGAACTACAACCCTGCTAACACATTGTTTACGTTAGAGTTTGGTTTAAATGATTTCATGAACTACA

ACCGTAGCGTGCCGGAAGTAAAAGCGGATTACGCTGAAGCTCTGATTCGTTTAACAGACGCTGGCGCGAAGAACT
TCATGTTGATGACACTGCCAGATGCAACCAAAGCACCACAGTTCAAATACTCAACACAGGAAGAAATTGAAACGA
TTCGTGCGAAAGTATTGAAGATGAACGAGTTCATCAAAGCACAAGCGATGTACTACAAAGCCCAAGGCTACAACA
TCGCATTGTTTGATACACACGCACTGTTTGAGAAGTTAACCTCGGCACCAGAAGAACACGGTTTCGTAAATGCAAG
CGACCCTTGCTTAGACATCAACCGTTCATCCTCTGTTGACTACATGTACACTCACTCATTACGTTCTGAATGTGCAG
CATCTGGCGCAGATAAGTTCGTGTTCTGGGATGTGACTCACCCAACCACAGCAACGCATCGTTATGTGGCTGAAAA
AATGTTAGAAAGCAGCAATAACTTAGAAGAGTTTCGCTTTTAA

>TS13
ATGATGAAAAAAACAATCACACTATTAACTGCATTACTTCCGCTTGCCTCTGCCGTTGCAGAGGAGCCAA
CATTATCACCAGAAATGGTTTCTGCGTCTGAAGTGGTCAGCACGCAAGAAAATCAAACCTATACTTACGT
TCGCTGCTGGTATCGTACGAGCCACTCTAAGGATGATGCGGCTACCGACTGGAAATGGGCAAAAAACCAA
GATGGTAGTGACTTCACTATCGACGGTTATTGGTGGAGCTCTGTTTCATTTAAAAACATGTTCTACACCA
ACACTTCACAAAACGTGATTCGCCAGCGCTGTGAAGAAACACTAGATCTGGCGAACGAGAACGCAGACAT
TACGTTTTTTGCTGCTGACAATCGCTATTCTTATAACCACACGATCTGGAGCAATGATGCTGCAATGCAA
CCAGATCAAATCAACAAAGTAGTGGCTTTGGGCGACAGCTTATCTGATACAGGCAACATCTTTAACGCTT
CCCAATGGCGCTTCCCTAACCCAAACAGCTGGTTCTTAGGTCACTTCTCGAACGGTTTTGTTTGGACGGA
ATACGTGGCTAAAGCCAAAAACTTACCGCTTTATAACTGGGCAGTTGGCGGCGCAGCAGGTGAGAACCAA
TACATTGCGCTAACAGGTGTCGGCGAACAAGTTTCTTCTTACTTGACCTACACAAAACTGGCGAAAAACT
ACAACCCAGCAAACACATTGTTTACGTTGGAGTTTGGTTTAAATGATTTCATGAACTACAACCGTAGCGT
GCCGGAAGTAAAAGCGGATTACGCTGAAGCTCTGATTCGTCTAACAGACGCGGGCGCGAAGAACTTCATG
TTGATGACACTGCCAGACGCAACCAAAGCACCACAGTTCAAATACTCAACGCAGGAAGAAATTGAAACGA
TTCGCGCGAAAGTATTGAAAATGAACGAGTTCATCAAAGCACAAGCGATGTACTATAAAGCTCAAGGTTA
CAACATTTCATTGTTTGATACGCACGCGCTGTTTGAGACGTTAACCTCGGCACCAGAAGAACACGGGTTC
GTGAATGCAAGTGATCCTTGTCTGGACATCAACCGCTCATCTTCTGTGGATTACATGTACACCCATTCAT
TACGCTCAGAGTGCGCGGCATCTGGCGCAGATAAGTTTGTGTTCTGGGATGTGACTCACCCAACCACAGC
CACGCATCGTTATGTTGCTGAAAAAATGTTGGAAAGCAGCAATAACTTAGAAGAGTTTCGCTTTTAA

>V2
ATGATGAAAAAAACAATCACACTATTAACTGCATTACTTCCGCTTGCCTCTGCCGTTGCAGAGGAGCCAACATTAT
CACCAGAAATGGTTTCTGCGTCTGAAGTGGTCAGCACGCAAGAAAATCAAACCTATACTTACGTTCGCTGCTGGTA
TCGTACGAGCCACTCTAAGGATGATGCGGCTACCGACTGGAAATGGGCGAAAAACCAAGATGGTAGTGACTTCAC
TATCGACGGTTATTGGTGGAGCTCTGTTTCATTTAAAAACATGTTCTACACCAACACTTCACAAAACGTGATTCGCC
AGCGCTGTGAAGAAACACTAGATCTGGCGAACGAGAACGCAGACATTACGTTTTTTGCTGCTGACAATCGCTATTC
TTATAACCACACGATCTGGAGCAATGATGCTGCAATGCAACCGGATCAAATCAACAAAGTAGTGGCTTTGGGCGA
CAGCTTATCTGATACAGGCAACATCTTTAACGCTTCCCAATGGCGCTTCCCTAACCCAAACAGCTGGTTCTTAGGTC
ACTTCTCGAACGGTTTTGTTTGGACGGAATACGTTGCTAAAGCCAAAAACTTACCGCTTTATAACTGGGCAGTTGG
CGGCGCAGCAGGTGAGAACCAATACATTGCGCTAACAGGTGTCGGCGAACAAGTTTCTTCTTACTTGACCTACACA
AAACTGGCGAAAAACTACAACCCAGCAAACACATTGTTTACGTTGGAGTTTGGTTTAAATGATTTCATGAACTACA
ACCGTAGCGTGCCGGAAGTAAAAGCGGATTACGCTGAAGCTCTGATTCGTCTAACAGACGCGGGCGCGAAGAACT
TCATGTTGATGACACTGCCAGACGCAACCAAAGCACCACAGTTCAAATACTCAACGCAGGAAGAAATTGAAACGA
TTCGCGCGAAAGTATTGAAAATGAACGAGTTCATCAAAGCACAAGCGATGTACTATAAAGCTCAAGGTTACAACA
TTTCATTGTTTGATACGCACGCGCTGTTTGAGACGTTAACCTCGGCACCAGAAGAACACGGGTTCGTGAATGCAAG
TGATCCTTGTCTGGACATCAACCGCTCATCTTCTGTGGATTACATGTACACCCATTCATTACGCTCTGAGTGTGCGG
CATCTGGCGCAGATAAGTTTGTGTTCTGGGATGTGACTCACCCAACCACAGCCACGCATCGTTATGTTGCTGAAAA
AATGTTGGGAAGCAGCAATAACTTAGAAGAGTTTCGCTTTTAA

>V1
ATGATGAAAAAAACAATCACACTATTAACTGCATTACTTCCGCTTGCCTCCGCCATTGCAGAGGAGCCAA
CTTTATCACCAGCAATGGTTTCTGCGGCTGAAGTGGTTAGCGCGCAAGAAAATCAAACTTATACGTACGT

TCGATGCTGGTATCGTACGAGCCACTCTAAGGACGATGCGGCTACCGATTGGAAATGGGCAAAAAACCAA
GATGGTAGTGACTTCACTATCGATGGCTATTGGTGGAGCTCTGTTTCATTTAAAAACATGTTCTACACCA
ATACCTCACAAAACGTAATTCGTCAGCGTTGTGAAGAAACACTAGATCTAGCGAACGAGAACGCAGACAT
TACATTTTTTGCTGCTGACAATCGTTATTCTTATAACCACACGATCTGGAGCAATGATGCTGCAATGCAA
CCAGATCAGATAAACAAAGTAGTGGCTTTAGGCGATAGCTTGTCTGATACAGGCAACATCTTTAACGCGT
CCCAATGGCGCTTTCCTAACCCGAATAGCTGGTTCTTGGGTCACTTCTCAAACGGTTTTGTTTGGACGGA
ATACGTTGCTAAAGCCAAAAACTTACCGCTATACAACTGGGCAGTTGGTGGCGCAGCAGGTGAAAACCAA
TACATCGCGCTAACTGGTGTCGGCGAACAAGTTTCTTCTTACTTAACCTACACAAAGCTGGCGAAGAACT
ACAACCCTGCTAACACATTGTTTACGTTAGAGTTTGGTTTAAATGATTTCATGAACTACAACCGTAGCGT
GCCGGAAGTAAAAGCGGATTACGCTGAAGCTCTGATTCGTTTAACAGACGCTGGCGCTAAGAACTTCATG
TTGATGACACTGCCTGATGCAACCAAAGCACCACAGTTCAAATACTCAACACAGGAAGAAATTGAAACGA
TTCGTGCGAAAGTATTGAAAATGAACGAGTTCATCAAAGCACAAGCGATGTACTACAAAGCCCAAGGCTA
CAACATCGCATTGTTTGATACACACACACTGTTTGAGAAGTTAACCTCGGCACCAGAAGAACACGGTTTC
GTAAATGCAAGCGACCCTTGCTTAGACATCAACCGTTCATCCTCTGTTGACTACATGTACACTCACTCAT
TACGTTCTGAATGTGCAGCATCTGGCGCAGATAAGTTCGTGTTCTGGGATGTGACTCACCCAACCACAGC
AACGCATCGTTATGTGGCTGAAAAAATGTTAGAAAGCAGCAATAACTTAGAAGAGTTTCGCTTTTAA
>E0601
ATGAAAAAAACAATCACACTATTAACTGCATTACTTCCGCTTGCCTCCGCCATTGCAGAGGAGCCAACTT
TATCACCAGCAATGGTTTCTGCGGCTGAAGTGGTTAGCGCGCAAGAAAATCAAACTTATACGTACGTTCG
ATGCTGGTATCGTACGAGCCACTCTAAGGACGATGCGGCTACCGATTGGAAATGGGCAAAAAACCAAGAT
GGTAGTGACTTCACTATCGATGGTTATTGGTGGAGCTCTGTTTCATTTAAAAACATGTTCTACACCAATA
CCTCACAAAACGTAATTCGCCAGCGTTGTGAGGAAACACTAGATCTAGCGAACGAGAACGCAGACATTAC
CTTTTTTGCTGCCGACAATCGTTATTCTTATAACCACACGATCTGGAGCAATGATGCTGCAATGCAACCA
GATCAAATCAACAAGGTAGTGGCTTTAGGCGATAGCTTGTCTGATACAGGCAACATCTTTAACGCGTCCC
AATGGCGCTTCCCTAACCCGAATAGCTGGTTCTTGGGTCACTTCTCAAACGGTTTTGTTTGGACGGAATA
CGTTGCTAAAGCCAAAAACTTACCGCTATACAACTGGGCAGTTGGTGGCGCAGCAGGTGAAAACCAATAC
ATCGCGCTAACTGGTGTCGGCGAACAAGTTTCTTCTTACTTAACCTACACAAAGCTGGCGAAGAACTACA
ACCCTGCTAACACATTGTTTACGTTAGAGTTTGGTTTAAATGATTTCATGAACTACAACCGTAGCGTGCC
GGAAGTAAAAGCGGATTACGCTGAAGCTCTGATTCGTTTAACAGACGCTGGCGCGAAGAACTTCATGTTG
ATGACACTGCCTGATGCAACCAAAGCACCACAGTTCAAATACTCAACACAGGAAGAAATTGAAACGATTC
GTGCGAAAGTATTGAAAATGAACGAGTTCATCAAAGCACAAGCGATGTACTACAAAGCCCAAGGCTACAA
CATCGCATTGTTTGATACACACGCACTGTTTGAGAAGTTAACCTCGGCACCAGAAGAACACGGTTTCGTA
AATGCAAGCGACCCTTGCTTAGACATCAACCGTTCATCCTCTGTTGACTACATGTACACTCACTCATTAC
GTTCTGAATGTGCAGCATCTGGCGCAGATAAGTTCGTGTTCTGGGATGTGACTCACCCAACCACAGCAAC
GCATCGTTATGTGGCTGAAAAAATGTTAGAAAGCAGCAATAACTTAGAAGAGTTTCGCTTTTAA

>A056
ATGAAAAAAACAATCACACTATTAACTGCATTACTTCCGCTTGCCTCCGCCATTGCAGAGGAGCCAACTT
TATCACCAGCAATGGTTTCTGCGGCTGAAGTGGTTAGCGCGCAAGAAAATCAAACTTATACGTACGTTCG
ATGCTGGTATCGTACGAGCCACTCTAAGGACGATGCGGCTACCGATTGGAAATGGGCAAAAAACCAAGAT
GGTAGTGACTTCACTATCGATGGCTATTGGTGGAGCTCTGTTTCATTTAAAAACATGTTCTACACCAATA
CCTCACAAAACGTAATTCGTCAGCGTTGTGAAGAAACACTAGATCTAGCGAACGAGAACGCAGACATTAC
ATTTTTTGCTGCTGACAATCGTTATTCTTATAACCACACGATCTGGAGCAATGATGCTGCAATGCAACCA
GATCAGATAAACAAAGTAGTGGCTTTAGGCGATAGCTTGTCTGATACAGGCAACATCTTTAACGCGTCCC
AATGGCGCTTTCCTAACCCGAATAGCTGGTTCTTGGGTCACTTCTCAAACGGTTTTGTTTGGACGGAATA
CGTTGCTAAAGCCAAAAACTTACCGCTATACAACTGGGCAGTTGGTGGCGCAGCAGGTGAAAACCAATAC
ATCGCGCTAACTGGTGTCGGCGAACAAGTTTCTTCTTACTTAACCTACACAAAGCTGGCGAAGAACTACA
ACCCTGCTAACACATTGTTTACGTTAGAGTTTGGTTTAAATGATTTCATGAACTACAACCGTAGCGTGCC
GGAAGTAAAAGCGGATTACGCTGAAGCTCTGATTCGTTTAACAGACGCTGGCGCTAAGAACTTCATGTTG
ATGACACTGCCTGATGCAACCAAAGCACCACAGTTCAAATACTCAACACAGGAAGAAATTGAAACGATTC
GTGCGAAAGTATTGAAAATGAACGAGTTCATCAAAGCACAAGCGATGTACTACAAAGCCCAAGGCTACAA

CATCGCATTGTTTGATACACACGCACTGTTTGAGAAGTTAACCTCGGCACCAGAAGAACACGGTTTCGTA
AATGCAAGCGACCCTTGCTTAGACATCAACCGTTCATCCTCTGTTGACTACATGTACACTCACTCATTAC
GTTCTGAATGTGCAGCATCTGGCGCAGATAAGTTCGTGTTCTGGGATGTGACTCACCCAACCACAGCAAC
GCATCGTTATGTGGCTGAAAAAATGTTAGAAAGCAGCAATAACTTAGAAGAGTTTCGCTTTTAA

>V05
ATGAAAAAAACAATCACACTATTAACTGCATTACTTCCGCTTGCCTCCGCCATTGCAGAGGAGCCAACTT
TATCACCAGCAATGGTTTCTGCGGCTGAAGTGGTTAGCGCGCAAGAAAATCAAACTTATACGTACGTTCG
ATGCTGGTATCGTACGAGCCACTCTAAGGACGATGCGGCTACCGATTGGAAATGGGCAAAAAACCAAGAT
GGTAGTGACTTCACTATCGATGGCTATTGGTGGAGCTCTGTTTCATTTAAAAACATGTTCTACACCAATA
CCTCACAAAACGTAATTCGTCAGCGTTGTGAAGAAACACTAGATCTAGCGAACGAGAACGCAGACATTAC
ATTTTTTGCTGCTGACAATCGTTATTCTTATAACCACACGATCTGGAGCAACGATGCTGCAATGCAGCCA
GATCAGATAAACAAAGTAGTGGCTTTAGGCGATAGCTTGTCTGATACAGGCAACATCTTTAACGCGTCCC
AATGGCGCTTTCCTAACCCGAATAGCTGGTTCTTGGGTCACTTCTCAAACGGTTTTGTTTGGACGGAATA
CGTTGCTAAAGCCAAAAACTTACCGCTATACAACTGGGCAGTTGGTGGCGCAGCAGGTGAAAACCAATAC
ATCGCGCTAACTGGTGTCGGCGAACAAGTTTCTTCTTACTTAACCTACACAAAGCTGGCGAAGAACTACA
ACCCTGCTAACACATTGTTTACGTTAGAGTTTGGTTTAAATGATTTCATGAACTACAACCGTAGCGTGCC
GGAAGTAAAAGCGGATTACGCTGAAGCTCTGATTCGTTTAACAGACGCTGGCGCTAAGAACTTCATGTTG
ATGACACTGCCTGATGCAACCAAAGCACCACAGTTCAAATACTCAACACAGGAAGAAATTGAAACGATTC
GTGCGAAAGTATTGAAAATGAACGAGTTCATCAAAGCACAAGCGATGTACTACAAAGCCCAAGGCTACAA
CATCGCATTGTTTGATACACACGCACTGTTTGAGAAGTTAACCTCGGCACCAGAAGAACACGGTTTCGTA
AATGCAAGCGACCCTTGCTTAGACATCAACCGTTCATCCTCTGTTGACTACATGTACACTCACTCATTAC
GTTCTGAATGTGCAGCATCTGGCGCAGATAAGTTCGTGTTCTGGGATGTGACTCACCCAACCACAGCAAC
GCATCGTTATGTGGCTGAAAAAATGTTAGAAAGCAGCAATAACTTAGAAGAGTTTCGCTTTTAA
